# Modular assembly and property profiling of an expansive, shape-defined library of pentacyclic saturated N-heterocycles

**DOI:** 10.1039/d6sc05613j

**Published:** 2026-09-02

**Authors:** Elia Boschi, Dmitry Mazunin, Alex Mueller, Daniel Zimmerli, David Wechsler, Marc-Olivier Ebert, Christian Bartelmus, Martin Binder, Bjoern Wagner, Christian Weinmann, Carsten Kroll, Wolfgang Haap, Jeffrey W. Bode

**Affiliations:** a Laboratory for Organic Chemistry, Department of Chemistry and Applied Biosciences, ETH Zürich Zürich 8093 Switzerland bode@org.chem.ethz.ch; b F. Hoffmann-La Roche Ltd, Pharma Research and Early Development pRED Innovation Center Basel Grenzacherstrasse 124 Basel CH-4070 Switzerland wolfgang.haap@roche.com

## Abstract

Small-molecule drug discovery is increasingly applied to targets historically classified as “undruggable”, such as non-coding RNAs and biomolecular interactions. These complex interfaces demand ligands with greater three-dimensional complexity and preferred physicochemical profiles that are deficient in typical small molecule chemical libraries housed in large pharmaceutical companies or commercial suppliers. Inspired by the need for novel libraries that might hypothetically address these target classes, we report the design, iterative synthesis, and comprehensive property profiling of a novel chemical library of shape-defined, morpholine-containing pentacycles. Utilizing iterative Stannyl Amine Protocol (iSnAP) reagents, we divergently assembled rigid, 3D-diverse scaffolds that exhibit a chameleonic basic character at physiological pH, elegantly balancing aqueous solubility with membrane permeability. Computational analysis of the 2400-member virtual library confirmed that it occupies a distinct, three-dimensional chemical space. Furthermore, high-throughput experimental profiling of the synthesized compounds revealed promising drug-like properties, including broad aqueous solubility, metabolic stability, low cytochrome P450 inhibition, and tunable membrane permeability. Although no bioactivity has yet been discovered from this library, the compounds offer a robust chemical foundation for future evaluations in ligand screenings targeting emerging therapeutic modalities.

## Abstract 2

Synthesis of 3D-complex, morpholine-containing pentacycles from iSnAP reagents. These shape-defined scaffolds occupy distinct chemical space and leverage chameleonic basicity to balance aqueous solubility with tunable membrane permeability.

## Graphical abstract

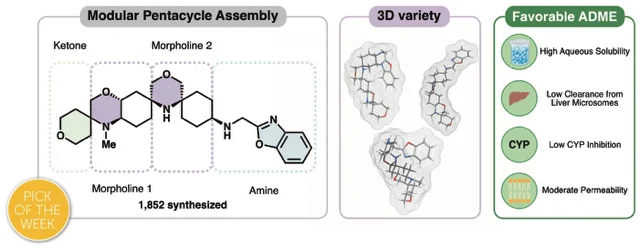

## Introduction

Small molecule drug discovery has expanded significantly beyond traditional enzyme and receptor inhibitors to address targets historically classified as “undruggable”.^1–3^ These target drivers include non-coding RNA, protein–protein, protein–DNA, and protein–RNA interactions, among others.^4–7^ The emergence of these target classes brings new requirements on their ligands, illustrated by the differences in binding interactions between ligands targeting proteins and RNA. While protein–ligand binding is predominantly stabilized by hydrophobic interactions, RNA–ligand binding relies primarily on π-stacking and hydrogen bonding.^8^ RNA binders are designed to be more cationic to engage the negatively charged phosphate backbone.^9–11^ The increased polarity associated with this cationic character typically limits oral bioavailability, as exemplified by the rRNA binding aminoglycoside antibiotics.^12,13^ To potentially meet these target-class-specific ligand requirements while maintaining optimal target selectivity and ADME properties, there is a critical need to design chemical libraries based on novel structural classes.

In our efforts to expand the chemical space of small molecules with suitable ADME properties for engaging difficult-to-drug targets, our group has developed new methodologies including the Stannyl Amine Protocol (SnAP) to access a variety of spirocyclic and fused saturated N-heterocycles.^14–16^ For example, we have prepared substituted morpholines with a p*K*_a_ around physiological pH, resulting in a morpholine nitrogen atom that is present in both cationic and neutral form. The p*K*_a_ can be tuned by the choice and position of substituents, making it a valuable building block for optimizing a ligand's properties and pharmacokinetics.^17–19^ Its saturated character brings the option of shaping its connectivity in 3D-space which is highly desired for increasing target selectivity. Notably, the number of morpholine-containing FDA-approved drugs has doubled from the year 2012 to the present, making it one of the 10 most ubiquitous N-heterocycles and emphasizing this scaffold's importance in drug discovery (Fig. 1A).^20^

**Fig. 1:**
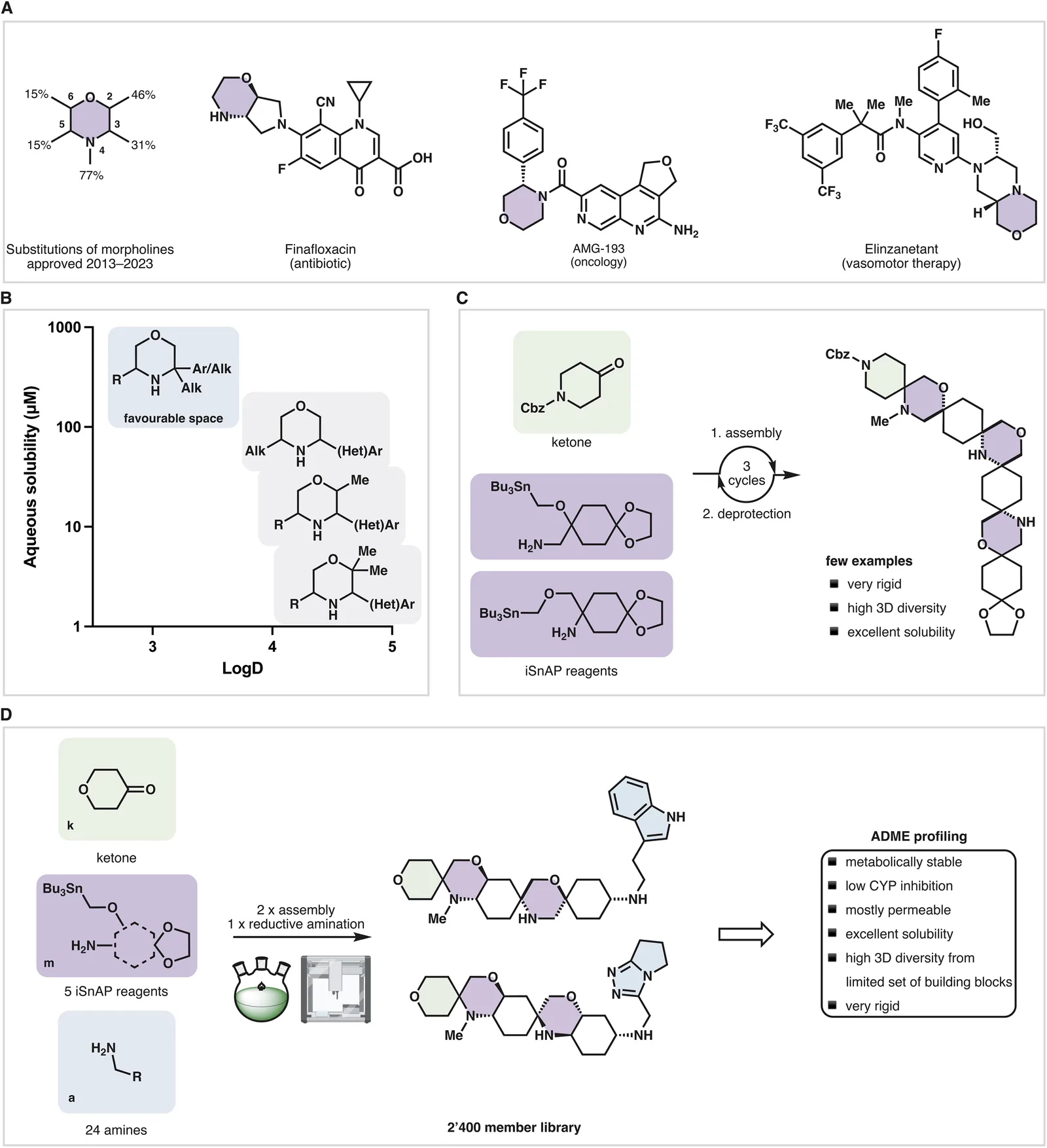
Morpholines in drug discovery and previous observation of morpholine substitution influence on solubility. (A) Morpholine-containing drugs and the relative substitution pattern of FDA approved drugs between 2013 and 2023. A drug molecule can bear multiple substituents on a morpholine ring, for example, at the 3- and 5-positions. (B) Previous findings on the log *D* value and solubility influence of different morpholine substitution patterns. (C) iSnAP reagents for programmable synthesis of multiple morpholine-containing small molecules. (D) This work: semi-automated synthesis of morpholine-containing, diversified pentacycles based on the combination of three building block classes and extensive ADME profiling.

In a previous study, we observed that trisubstituted morpholines surprisingly showed improved water solubility over their disubstituted analogues. The trisubstituted morpholine's average log *D* value of around 3 at physiological conditions suggested a good balance of membrane permeability, metabolic stability and solubility (Fig. 1B).^21^ In another study, we iteratively synthesized a limited number of morpholine-containing oligocycles with diverse three-dimensional shapes (Fig. 1C).^22^ These oligocycles were assembled using iterative SnAP (iSnAP) chemistry from carefully selected bifunctional building blocks constituting an organostannane and a protected ketone handle, which could be unmasked for iterative construction of spirocyclic morpholine chains. This approach yielded tailor-made, rigid 3D-structures with high aqueous solubility—molecules of potential value for drug discovery. While no bioactive compounds have yet been validated and their performance in ligand screenings against these specific targets remains to be determined, to further investigate the potential of this compound class, we wished to fully interrogate their ADME properties.^23^

In this report we disclose a divergent, modular synthesis of morpholine-containing pentacycles, coupled with automated diversification, to assemble a library of 2400 structurally diverse compounds (Fig. 1D). To systematically investigate the physicochemical properties of these scaffolds, we developed a semi-automated approach amenable to future structure–activity relationship (SAR) studies. This strategy utilized bifunctional building blocks that paired iSnAP reagents with a ketal-protected ketone. Following the construction of the initial morpholine core, deprotection revealed a new ketone handle, which then served as the substrate for either a subsequent iterative SnAP reaction or a reductive amination. The building blocks were strategically selected to balance synthetic accessibility with the full exploration of three-dimensional (3D) structural diversity.

## Results and discussion

### Library design and synthesis

We designed a library of rigid, spirocyclic pentacycles featuring fused saturated rings appended to aromatic heterocycles. The SnAP cyclization can be conducted with either aldehyde or ketone substrates, determining the substitution alpha to the heterocyclic nitrogen. We chose cyclic ketones to maximize rigidity through the concomitant formation of spirocycles and increasing the druglike character of the target compounds. The limited set of five iSnAP reagents was selected to maximize the 3D diversity including spirocycles and fused cycles with two chiral centers, while minimizing synthetic overhead. For this systematic study, we chose a single starting ketone, tetrahydropyranone (Fig. 2A). The pentacycles were assembled from a ketone oxacycle k, 5 bifunctional, iSnAP reagents mA–mE which were installed in two iterations and 24 carefully chosen primary amines a1–a24 for further diversification. For the diversification, primary amines were chosen to ensure reliability of the reductive amination. Furthermore, they were selected with the goal of constructing a broad set containing either benzylic or homobenzylic amines including pure carbon, oxygen and nitrogen containing aromatics. Fluorinated aromatics were also included to tune the electronics of the heterocycle and the amine. While the resulting secondary amine of the morpholine within the pentacyclic core offers an additional vector for diversification, functionalization at this position requires substrate-specific optimization due to the high steric constraints of the substituted morpholines, and was therefore not systematically pursued for this library.

**Fig. 2:**
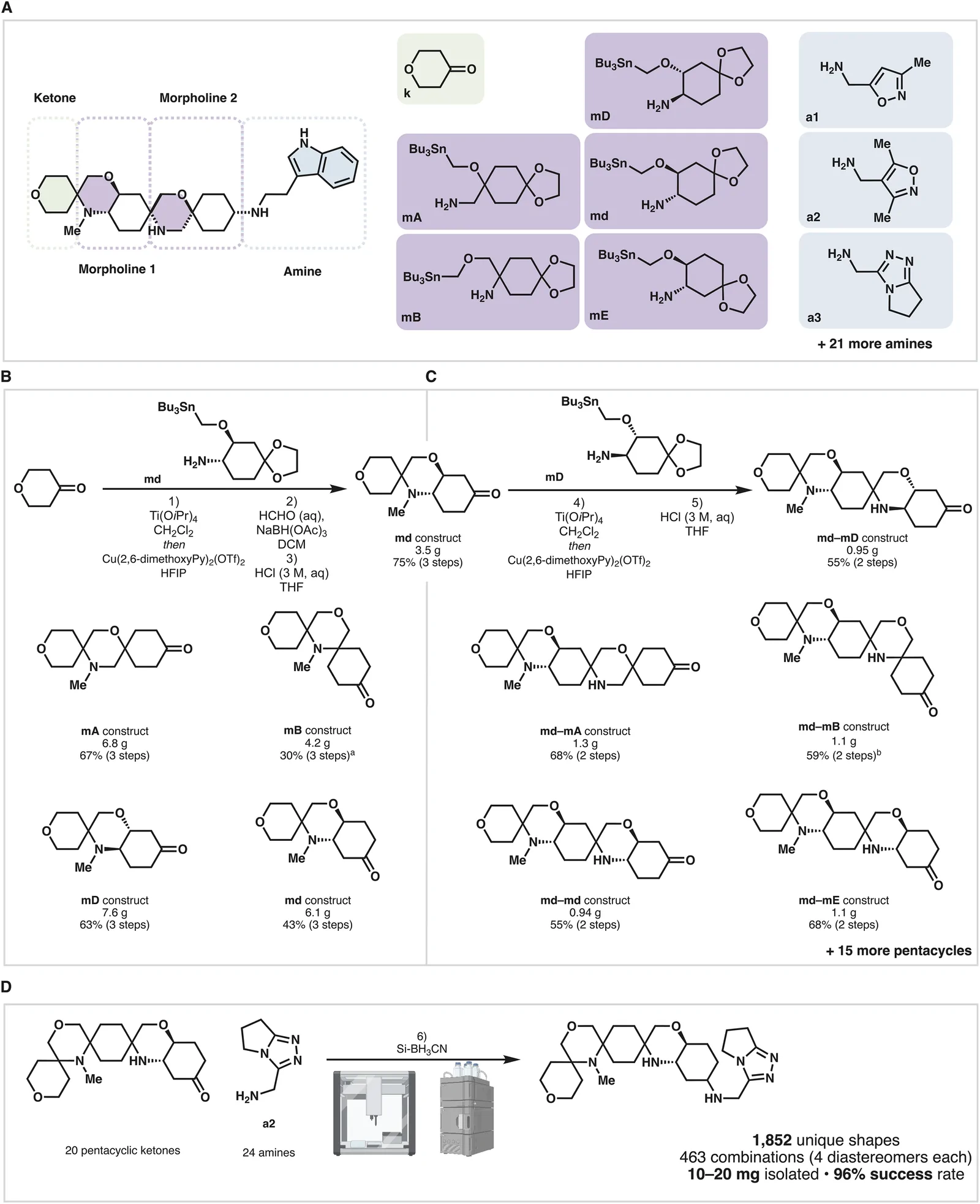
Design and synthesis of diversified morpholine-containing pentacycles. (A) Library design with incorporated building block classes. (B) Construction of tricycles from tetrahydropyranone and diverse, bifunctional iSnAP reagents. ^*a*^Ketimine formation with 4 equiv excess of tetrahydropyranone. (C) Assembly of pentacycles from tricycles and diverse, iSnAP reagents. ^*b*^Ketimine formation neat under high vacuum. (D) Automated diversification of pentacyclic ketones with diverse primary amines.

The ketone k and the bifunctional iSnAP reagents mA to mE delivered the first spirocyclic morpholines as tricycles, which were *N*-methylated and deprotected to the respective ketones (*e.g.*mA construct, Fig. 2B). The tricyclic ketone was coupled with second SnAP reagents mA to mE to form the second morpholine core and the products were deprotected to the respective ketone (*e.g.*mA–mB construct, Fig. 2C). From 25 hypothetical morpholine 1–morpholine 2 combinations, 20 were successfully synthesized on gram scale and isolated by flash column chromatography as mixtures of two diastereomers. The other five pentacycles from the m5 tricycle were not obtained due to an unexpected β-elimination in the second SnAP reaction, enabled by the 1,3-diamine substitution pattern on the pentacycle's central cyclohexane ring (Fig. S1).

The 20 resulting pentacyclic ketones were further elaborated by semi-automated reductive amination on a liquid handler to prepare the final targets (Fig. 2D). The reductive amination was performed with silica supported cyanoborohydride, which was removed simply by sedimentation after the reaction to deliver a crude mixture that was concentrated by nitrogen blowdown and then purified by reverse phase HPLC. From the reductive amination step 463 building block combinations out of 480 attempted provided isolatable material in quantities between 10 and 20 mg each. This corresponded to a 96% success rate on the final step.

### Diastereomer assignment

Each building block combination furnished four diastereomers that, in selected cases, were separated by HPLC or SFC. While we successfully crystallized and assigned one complete set of four diastereomers *via* X-ray crystallography (Fig. 3A), purely experimental assignment methods were not viable for larger numbers. Because routine X-ray analysis was hindered by crystallization bottlenecks and NOE-NMR lacked the required correlations, we explored computationally aided methods. Out of a variety of alternative methods including ion-mobility MS, IR, vibrational circular dichroism (VCD), ^1^H or ^13^C-NMR comparisons with predictions, we chose ^13^C-NMR (Fig. 3).^24–26^ The advantage of ^13^C-NMR over other methods lies in the good signal separation and the multiple datapoints describing the environment of the stereogenic centers, leading to higher confidence in the assignment.

**Fig. 3:**
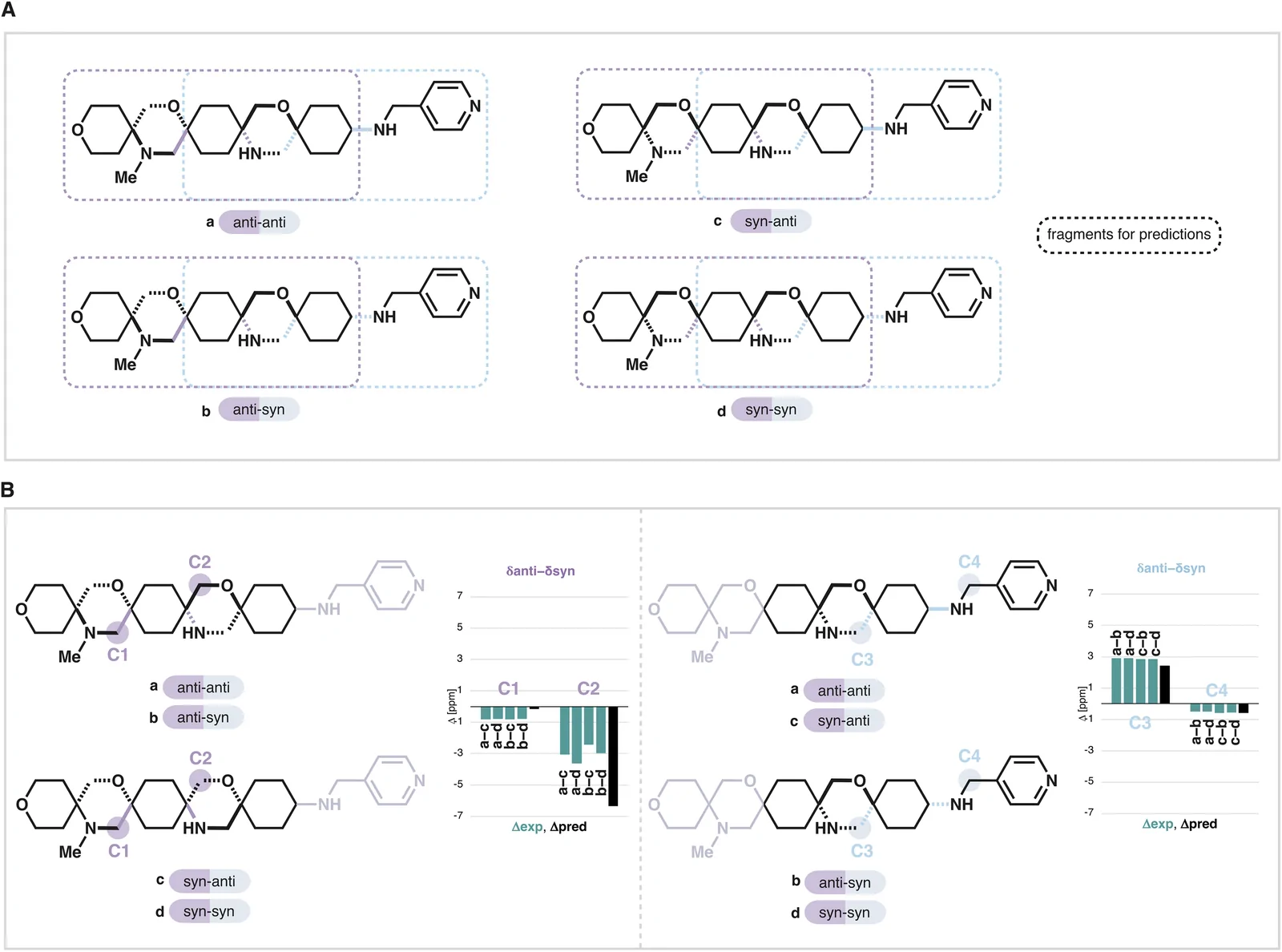
Diastereomer assignment example. Comparison of the experimental NMR *δ*_13C_ differences of two diastereomeric carbons to the corresponding predicted differences. (A) Set of 4 possible and accessed diastereomers successfully assigned by X-ray crystallography. Boxes indicate the fragments used for predictions. (B) NMR *δ*_13C_ differences between diastereomeric carbons rendering the assignment of diastereomers possible. All 4 diastereomer assignments matched their X-ray structures. Additional examples of assignment can be found in Fig. S2.

We assigned the structure of these separated compounds by comparing predicted ^13^C-NMR chemical shift differences between diastereomers with experimental values. Our computational workflow involved generating a conformer ensemble using CREST^27^ (gfn2), followed by conformer filtering, optimization, and ^13^C-NMR shift calculations. We employed CENSO^28^ for conformational analysis and chemical shift computations (optimized on b97-d3/def2-TZVP level, SPE b3lyp-d4/def2-QZVPP and NMR chemical shift pbe0-d4/cc-pVTZ). To mitigate computational error, we compared predicted and experimental ^13^C-NMR shift differences, achieving mean absolute errors (MAE) of 0.5–1.1 ppm for several examples. Crucially, each diastereomer pair displayed a Δ*δ* > 2 ppm for at least one carbon in proximity to the stereogenic center, rendering the prediction accuracy sufficient.

### Virtual library analysis

To characterize and compare compound classes to the available chemical space, the virtual library (VL) was enumerated based on the employed building blocks (Fig. 4A). The assembly of the building blocks enumerated to 1 × 5 × 5 × 24 = 600 different combinations of four diastereomers each, counting 4 × 600 = 2400 unique compounds. A Tanimoto similarity search was performed relative to Roche's chemical space and to several commercial libraries based on the 2D Extended-Connectivity Fingerprints with a radius of 4 (ECFP4), a derivative from Morgan circular fingerprints (Fig. 4B).^29,30^ Fulfilling our objective to access novel chemical space, the library of pentacycles exhibited the targeted 2D dissimilarity, maintaining Tanimoto similarities of <0.7 compared to both the Roche and commercial libraries, a generally accepted threshold of dissimilarity in a medicinal chemistry context.^31,32^ Comparison of the pentacycles to their most similar commercial entities indicated that the similarity came from the utilized commercial amine building blocks. Ranging from 450 to 600 Da, the molecular weight of the VL occupies a size regime relevant for modern drug discovery campaigns (Fig. S3). Moreover, we envisioned that other VL characteristics are very favorable including log *P* of 1–5, topological polar surface area (TPSA) mostly below 90 Å^2^, the mostly low number of hydrogen bond donors of 2, hydrogen bond acceptors between 6 and 10, the high sp^3^ carbon fraction of 0.7–0.93 and low number of rotatable bonds between 3 and 5.

**Fig. 4:**
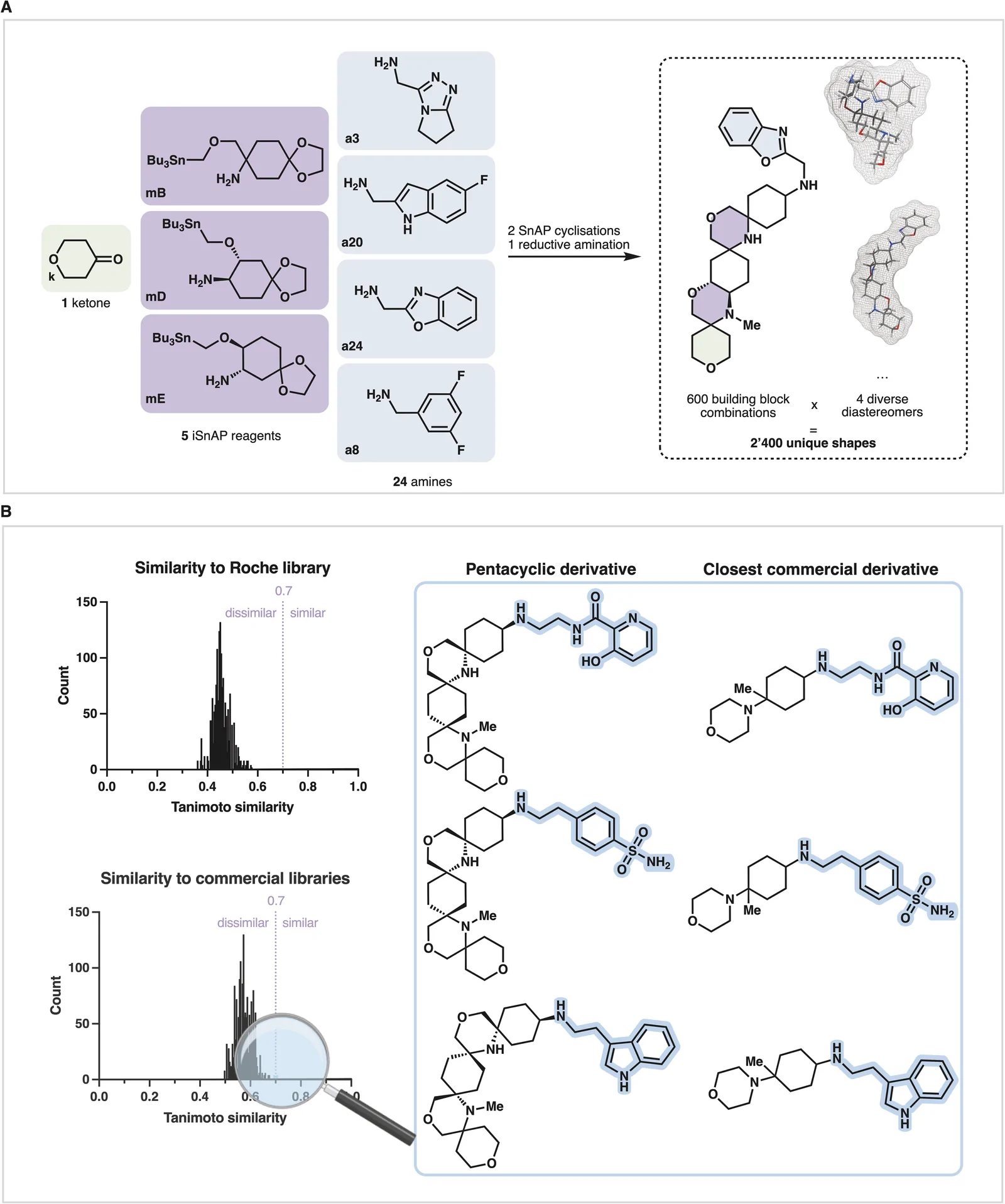
Virtual library enumeration and analysis. (A) Selection of used building blocks to construct the virtual library and example set of 4 diastereomers formed by a building block combination with computed minimal 3D structures (CREST-gfn2-hexane, CENSO-pbe-d4/def2-svp/cpcm cyclohexane). (B) Closest Tanimoto similarities to compounds from the Roche and commercial libraries based on ECFP4 fingerprints and structures of the most similar commercial compounds (4 × 10^9^ compounds available at Enamine, Mcule, WuXi or Emolecules; off-the-shelf and make on demand).

Analysis of the 20 accessible morpholine pentacycles revealed a high degree of rigidity; Boltzmann-weighted conformer ensemble averages of heavy-atom RMSD values dependent on the morpholine 1 and morpholine 2 building blocks ranged from 0.15 Å to 0.55 Å (Fig. 5B). The conformationally locked *trans*-fused cycles mD, md and mE accounted for higher rigidities (lower RMSD) compared to the conformationally more flexible spirocyclic building blocks mA and mB. In terms of 3D diversity, the radius of gyration ranged from 3.8 to 4.8 Å, while the pentacycle length, defined as the distance between the oxacycle oxygen and the primary amine nitrogen, varied between 8 and 16 Å (Fig. 5C). This significant variation, highlighted by a two-fold difference in length, underscores the extensive 3D structural diversity accessible within this limited set of morpholine-containing pentacycles derived from iSnAP reagent building blocks.

**Fig. 5:**
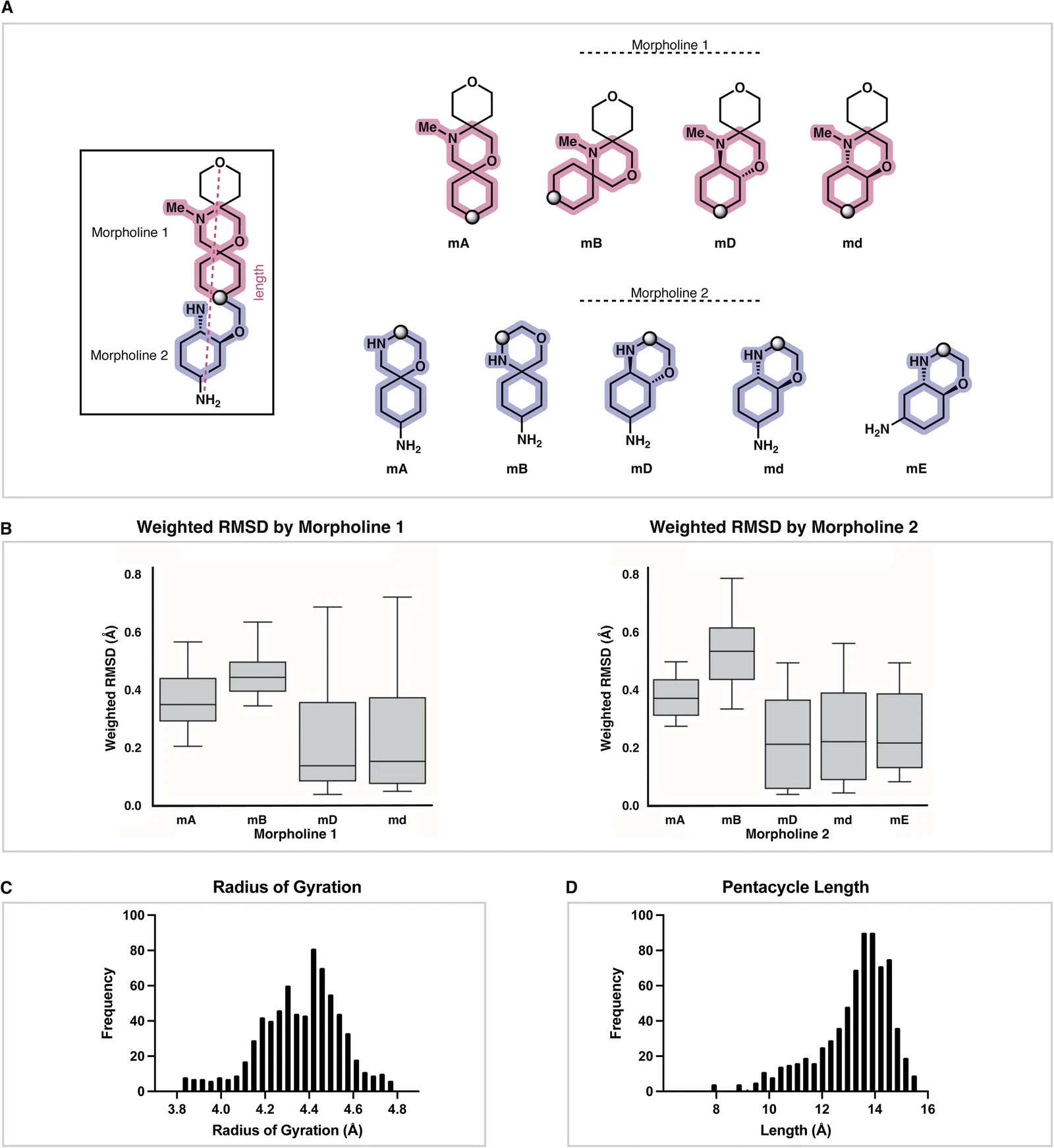
3D diversity and flexibility analysis of the 20 accessed morpholine containing pentacycles. (A) Overview on the used building blocks with different substitution patterns and definition of the analyzed pentacycle length from the oxacycle oxygen to the primary amine. (B) Boltzmann-weighted heavy atom-RMSD of the conformer ensembles by the morpholine 1 and morpholine 2 building blocks. (C and D) Distribution of the radius of gyration of the pentacycles and their length.

### Experimental ADME profiling

Following the construction of the morpholine-containing pentacyclic library, we sought to evaluate the suitability of this compound class for drug discovery applications. To this end, we conducted a comprehensive ADME profiling campaign. The general profiling included solubility, permeability, lipophilicity (log *D*), metabolic stability (intrinsic clearance) in mouse and human liver microsomes, and inhibition of the three major cytochrome P450 (CYP) isoforms involved in drug metabolism. These parameters were selected to maximize the profiling spectrum while considering assay capacities. The morpholine-containing pentacycle library was profiled on unseparated mixtures of diastereomers.

Starting from mixtures of diastereomers, metabolic stability was assessed by measuring clearance in mouse and human liver microsomes. In human microsomes, strikingly the vast majority (69%) of the measured compounds exhibited a favorably low clearance of 10 µL per min per mg protein (lowest possible clearance in this assay setup), a proportion that even increased to 86% in mouse microsomes (Fig. 6A). Conversely, only 14% of compounds were rapidly cleared in human microsomes and 4% in mouse microsomes, with the remaining fraction displaying an intermediate clearance profile. Overall, these findings indicated that this compound class possesses excellent metabolic stability across both species.

**Fig. 6:**
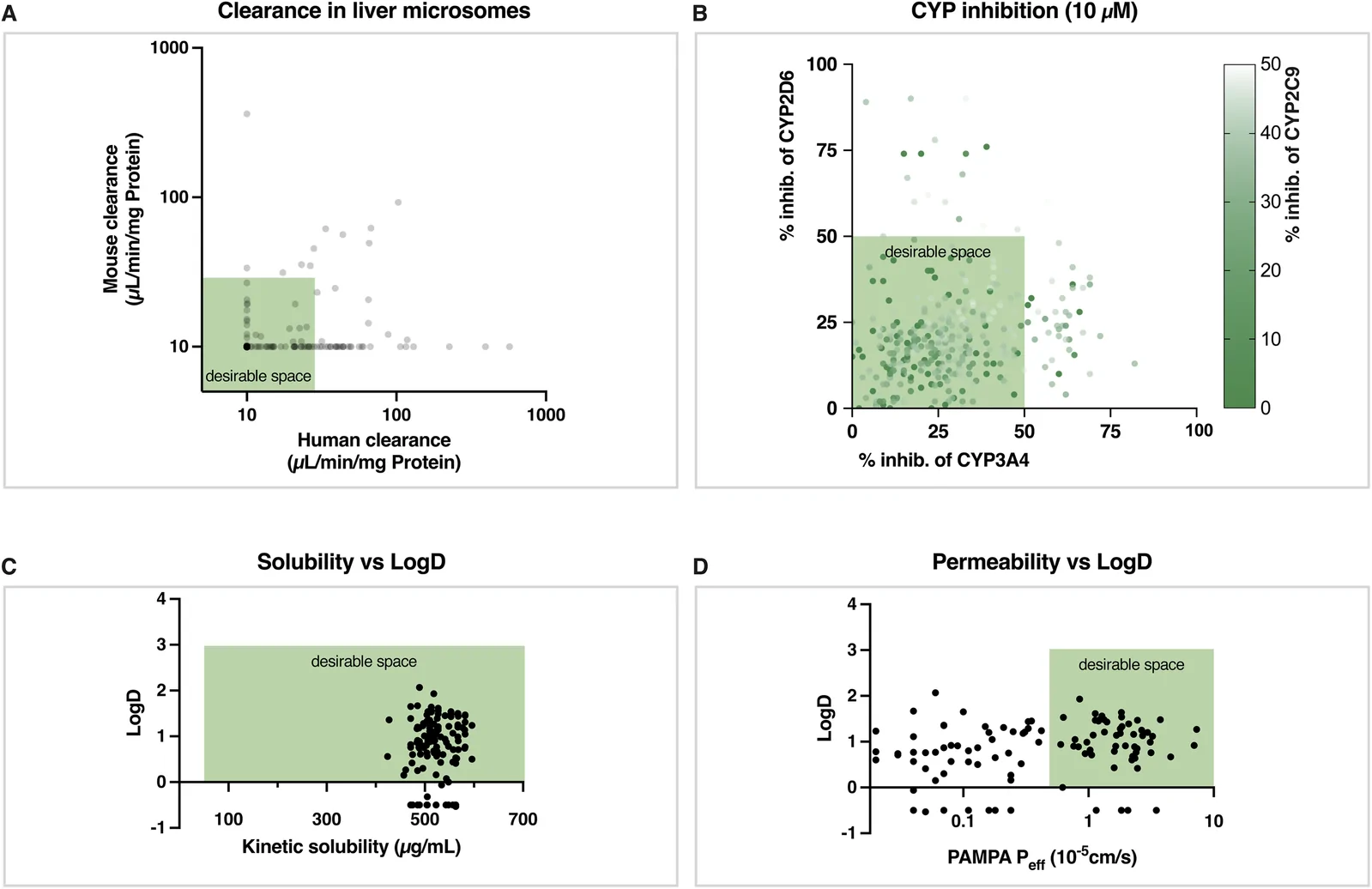
ADME properties of morpholine containing pentacycles. Green area highlights target properties for a chronic 50–100 mg daily oral dose. (A) Clearance of library compounds in liver microsomes: each datapoint consists of one library member as mixture of diastereomers, showing its clearance in human *vs.* mouse microsomes. Dark points indicate overlap of multiple datapoints. (B) CYP inhibition at 10 µM compound concentration of three major CYP isoforms responsible for drug metabolism. Each datapoint consists of one library member as mixture of diastereomers. (C) Kinetic solubility and (D) permeability (PAMPA) of the library compounds compared to their log *D*. Each datapoint consists of one library member as mixture of diastereomers.

The vast majority of the library of pentacycles showed a favorable, low CYP inhibition profile with less than 3% causing moderate (>60%) inhibition of CYP2D6 and CYP2C9 at 10 µM compound concentration, while only 5% of the compounds were strong inhibitors of CYP3A4 (Fig. 6B). This indicates that these pentacycles carry a low risk of causing cytochrome P450-mediated drug–drug interactions.

Slightly more than half of the library exhibited low lipophilicity at pH 7.4, with log *D* values ranging between −1 and 1, while the remaining portion displayed log *D* values between 1 and 2 (Fig. 6C). All compounds were readily water soluble with a kinetic solubility larger than 400 µg mL^−1^ at pH 6.5. Regarding permeability, one-third of the library was classified as passively permeable in the PAMPA assay at pH 7.4 (Fig. 6D), whereas the remaining two-thirds exhibit lower permeabilities. This profile highlights an inherently hydrophilic scaffold with excellent aqueous solubility, albeit with an expected trade-off in passive membrane diffusion. Notably, no correlation was observed between lipophilicity and either kinetic solubility or permeability (Fig. 6C and D), suggesting that the log *D* does not govern absorption in this series, pointing instead toward more complex structural factors.

The observed permeability correlated significantly with the nature of the (hetero)aromatic moiety introduced through the amine building block (Fig. 7A). This modification altered the number of hydrogen bond donors (HBD) in the diversified pentacycles, ranging from 2–4. Specifically, compounds with 4 HBD consistently exhibited low permeability, whereas those with 2–3 HBD displayed a broad distribution ranging from low to high permeability, heavily dependent on the specific (hetero)aromatic system (Fig. 7B). This highlights the classic penalty of excessive hydrogen bond donors on membrane penetration. Despite this strong correlation with the amine building blocks, a significant distribution in effective permeability was observed among pentacycles sharing the same amine (*e.g.*, amines a1, a2, a10, or a15), suggesting that the varying shapes of the morpholine scaffolds also influenced permeability (Fig. 7C). Together, these findings indicate that while limiting HBDs is necessary for absorption, the 3D geometry of the core scaffold can be actively leveraged to fine-tune passive permeability.

**Fig. 7:**
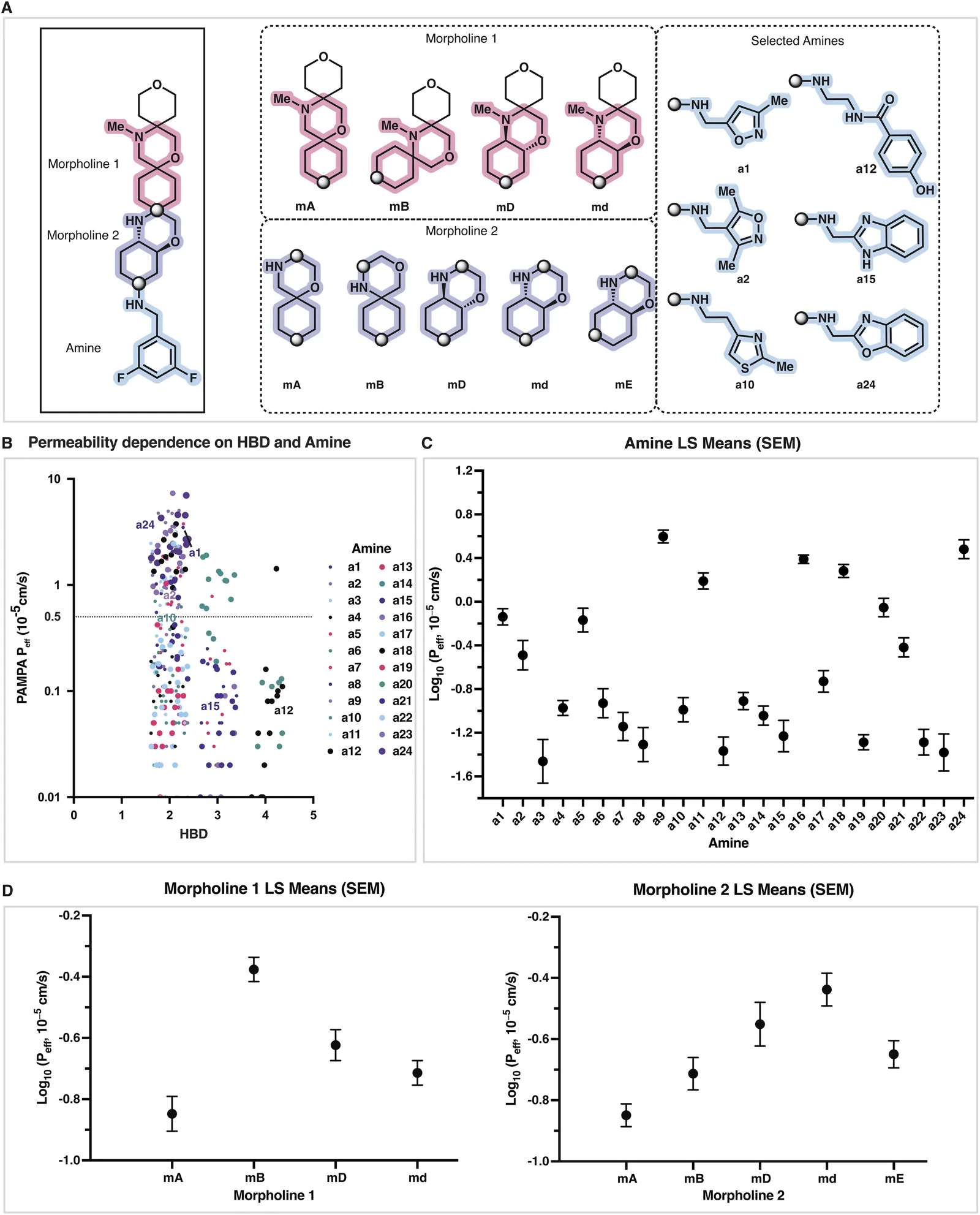
Permeability dependence on building blocks. (A) Overview on the morpholine building blocks and a selection of the 24 amine building blocks. (B) PAMPA dependence on #HBD and amine building blocks with each datapoint consisting of one building block combination. (C) Dependence on amine building blocks illustrated based on generalized regression ridge model. (D) Influence through morpholine building blocks illustrated based on generalized regression ridge model.

To elucidate the specific influence of the first and second morpholine units (morpholine 1 and morpholine 2) on permeability (Fig. 7A), several predictive models were trained with parameter screening on building block combinations labeled with log(*P*_eff_) values. Among various linear regression, random forest, and Graph Neural Network (GNN) algorithms, a linear, Generalized Regression Ridge model performed best on a randomly selected 20% test set. This model successfully explained the permeability variance (*R*^2^ = 0.83 *p* = 0.017, *n* = 50) when comparing predicted *versus* actual log(*P*_eff_) data, indicating a strong effect according to Cohen.^33^

This statistical analysis revealed that while the amine building block exerted the strongest statistical evidence of association with permeability (Wald Chi^2^ = 1646), morpholine 1 (Wald Chi^2^ = 41) showed higher statistical evidence of association than morpholine 2 (Wald Chi^2^ = 18). The morpholine 1–morpholine 2 factor showed a Wald Chi^2^ = 30. Structurally, morpholine mA, which possessed only two substituents α to the nitrogen, generally caused a significant decrease in permeability compared to morpholines mB, mD, md, and mE, which featured 3–4 α-substituents (Fig. 7D).

This trend held true whether mA was introduced at the first or second morpholine position. Specifically, for the morpholine 1 position, mB generally conferred the highest permeability, followed by mD and md with mA showing the lowest values. For the morpholine 2 position, the differentiation in permeability influence among mB, mD, md, and mE was less distinct.

Structurally, this substitution influence on the permeability could be attributed to the increase in steric bulk associated with a higher number of α-substituents. This hydrophobic congestion likely shielded the polar and potentially ionized nitrogen atom, thereby rendering the morpholine motif more lipophilic and facilitating its passage through the lipid bilayer.

To better understand how the morpholine subunits influence permeability, p*K*_a_ values were measured for varying combinations of morpholine 1 and morpholine 2, utilizing a constant amine building block (a5) and analyzing the compounds as mixtures of diastereomers (Fig. 7D). For each molecule, three distinct p*K*_a_ values were determined. Based on the structural contributions of the building blocks and independent verification through NMR experiments, the highest p*K*_a_ value was assigned to the acyclic amine (p*K*_a_ 3), the intermediate value to the less substituted morpholine nitrogen (p*K*_a_ 2), and lowest value to the methylated morpholine nitrogen (p*K*_a_ 1) (Fig. 8).

**Fig. 8:**
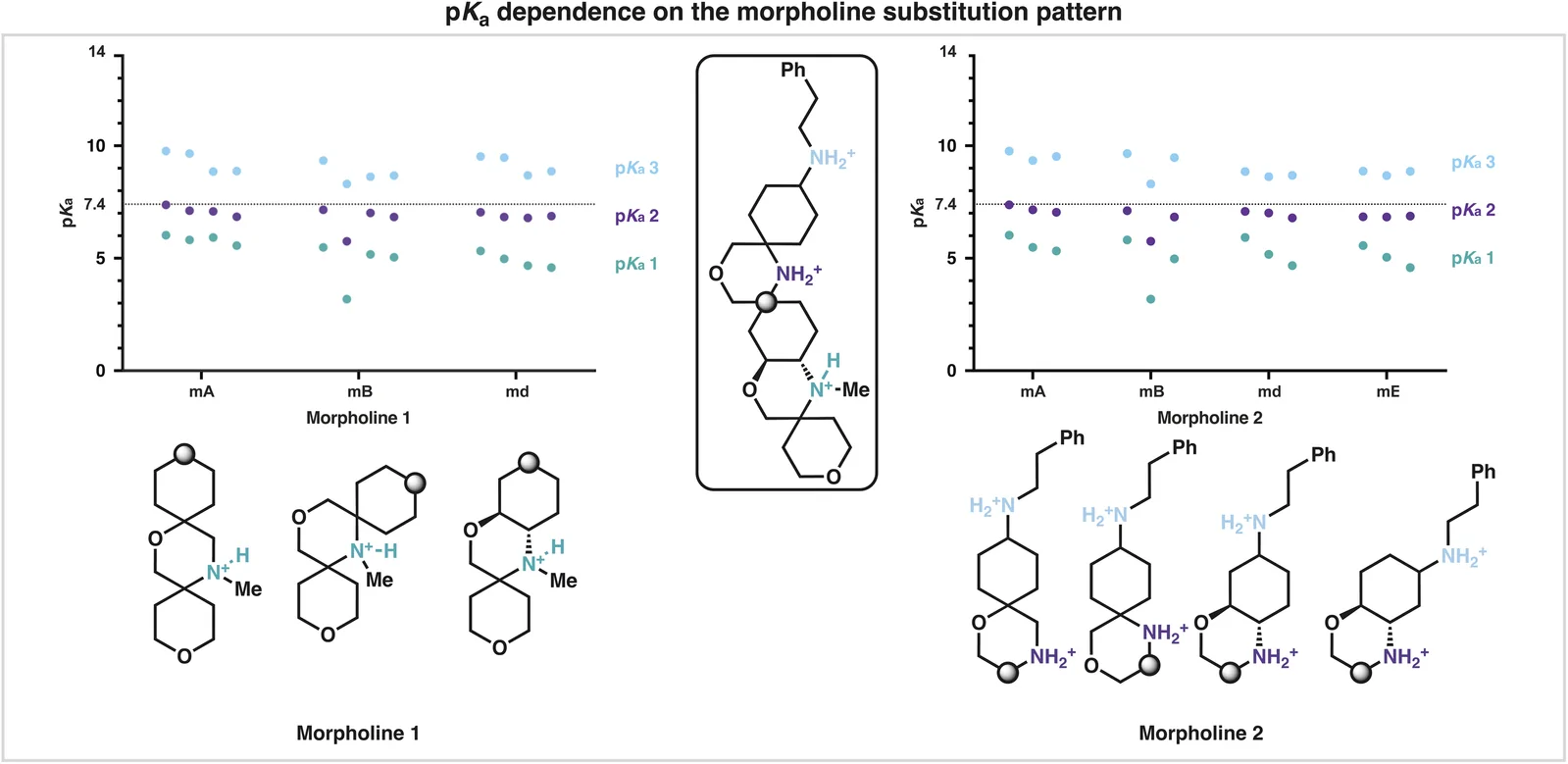
p*K*_a_ values of morpholine containing pentacycles with constant amine building block. The lowest p*K*_a_ 1 was assigned by NMR experiments to the methylated morpholine, p*K*_a_ 2 to the non-methylated morpholine and p*K*_a_ 3 to the terminal amine.

While the lower p*K*_a_ 1 in the pentacycles showed a stronger dependence on morpholine 1 than morpholine 2, the higher p*K*_a_ 3 correlated strongly to the proximal morpholine building block, morpholine 2. For both, p*K*_a_ 1 and 3, a trend was observed from higher p*K*_a_ for the sterically less crowded morpholine mA to lower p*K*_a_-values for the morpholines mB–mE containing 3–4 α-substituents to the nitrogen. This trend is consistent with the permeability dependence on the substitution pattern of spirocyclic morpholines.

In contrast to the lower and higher p*K*_a_ in the pentacycles, the middle p*K*_a_ 2 did not display any significant morpholine building block dependence with the exception of the outlier mB–mB which showed the lowest p*K*_a_ values compared to the other building block combinations. These lowered p*K*_a_-values are likely caused by the high steric congestion resulting from the 4 α-substituents to the morpholine nitrogens and its highly twisted shape. Finally, only minor p*K*_a_ variations were observed between diastereomers of selected building block combinations, indicating that basicity depends predominantly on the overall morpholine substitution pattern rather than the relative spatial orientation of the nitrogen atoms within the scaffold (Fig. S4).

## Conclusions

In conclusion, we have established a modular, semi-automated synthesis for the construction of morpholine-containing pentacycles. With this approach, a library of 1852 pentacycles was prepared from five iSnAP reagents and 24 amines with a 96% success rate in 463 unique building block combinations. In total, demonstrating that expansive structural diversity can be accessed using just a few carefully selected iSnAP reagents. This synthetic platform provided access to a vast array of unique three-dimensional shapes characterized by high aqueous solubility, metabolic stability, low cytochrome P450 inhibition, and tunable membrane permeability. Ultimately, this reliable synthetic methodology, coupled with the elucidated structure–property relationships regarding morpholine substitution patterns, provides a powerful framework to support future medicinal chemistry campaigns in the optimization of hit and lead structures.

## Author contributions

J. W. B., W. H., E. B., D. M., and C. K. conceived the idea and designed the experiments. E. B. synthesized the library, D. Z. and D. W. ran HT purifications and diastereomer separations, C. B., M. B., B. W. and C. W. ran HT characterizations. E. B. and M. O. E. assigned diastereomers by NMR. E. B. and A. M. prepared and analyzed the virtual library. E. B. analyzed the ADME data which was discussed with J. W. B., W. H. and D. M. The manuscript was written by E. B., J. W. B. and W. H.

## Conflicts of interest

There authors have no conflicts of interest to declare.

## Supplementary Material

SC-017-D6SC05613J-s001

SC-017-D6SC05613J-s002

## Data Availability

CCDC 2557952, 2557953, 2557955–2557959 contain the supplementary crystallographic data for this paper.^34*a*–*g*^ The data supporting this article have been included as part of the supplementary information (SI). Supplementary information: additional figures, synthetic procedures, characterization data, and NMR spectra for all new compounds, computational procedures, calculated structures, and X-ray crystallographic data (PDF). NMR and LCMS of the library consisting of 463 building block combinations (S1–S3), library's experimental ADME data (S4), virtual library (S5), and the pentacycle conformer ensembles with shape analysis (S6) are available from Zenodo (doi.org/10.5281/zenodo.20431925). See DOI: https://doi.org/10.1039/d6sc05613j.
