# Supplementary material for: Modular assembly and property profiling of an expansive, shape-defined library of pentacyclic saturated N-heterocycles
Source: Chem Sci. 2026 Sep 2;17(40):19235–46. doi: 10.1039/d6sc05613j (PMC13537041; doi:10.1039/d6sc05613j)

Supplementary Information (SI) for Chemical Science.
This journal is © The Royal Society of Chemistry 2026

Supporting Information

Modular Assembly and Property Profiling of an Expansive, Shape-Defined Library of Pentacyclic Saturated N-Heterocycles

Elia Boschi[1], Dmitry Mazunin[2], Alex Mueller[2], Daniel Zimmerli[2], David Wechsler[2], Marc-Olivier Ebert[1], Christian Bartelmus[2], Martin Binder[2], Bjoern Wagner[2], Christian Weinmann[2], Carsten Kroll[2], Wolfgang Haap[2,], and Jeffrey W. Bode[1,*]*

[1]Laboratory for Organic Chemistry, Department of Chemistry and Applied Biosciences, ETH Zürich,
8093 Zürich, Switzerland; bode@org.chem.ethz.ch

[2]F. Hoffmann-La Roche Ltd. Pharma Research and Early Development pRED Innovation Center Basel, Grenzacherstrasse 124, CH-4070 Basel, Switzerland;
wolfgang.haap@roche.com

TABLE OF CONTENTS

1 Supplementary figures ____ *3*

2 ADME profiling ____ *6*

2.1 Building block structures ____ **7**

2.2 Permeability ____ **8**

2.3 Clearance in liver microsomes ____ **11**

2.3.1 Clearance in human liver microsomes ____ 11

2.3.2 Clearance in mouse liver microsomes ____ 13

2.4 CYP inhibition ____ **15**

2.4.1 HLM CYP3A4 inhibition ____ 15

2.4.2 HLM CYP2D6 inhibition ____ 17

2.4.3 HLM CYP2C9 inhibition ____ 19

2.5 Additional relations between properties ____ **21**

2.5.1 Data distributions ____ 23

2.6 Linear model for permeability dependence on building blocks ____ **26**

2.7 ADME assay descriptions ____ **27**

3 Cheminformatic workflows ____ *28*

3.1 VL enumeration and analysis ____ **28**

3.1.1 VL analysis ____ 29

3.1.2 3D shape analysis of pentacycle cores ____ 29

3.2 ^{13}C NMR predictions for diastereomer assignment ____ **30**

3.2.1 Conformer sampling ____ 30

3.2.2 Conformer sorting, and ^{13}C NMR calculations ____ 30

4 Library synthesis ____ *34*

4.1 General remarks ____ **34**

4.2 Preparation of the copper catalyst $Cu(DMP)_2(OTf)_2$ ____ **36**

4.3 SnAP reagents ____ **36**

4.4 General procedures ____ **36**

4.5 Synthesis of tricycles ____ **38**

4.6 Synthesis of pentacycles ____ **47**

4.7 Automated reductive amination and purification ____ **73**

4.7.1 Separated diastereomers ____ 74

5 X-Ray crystallography data ____ *84*

6 References ____ *91*

6 NMR spectra of intermediates ____ *91*

1 SUPPLEMENTARY FIGURES

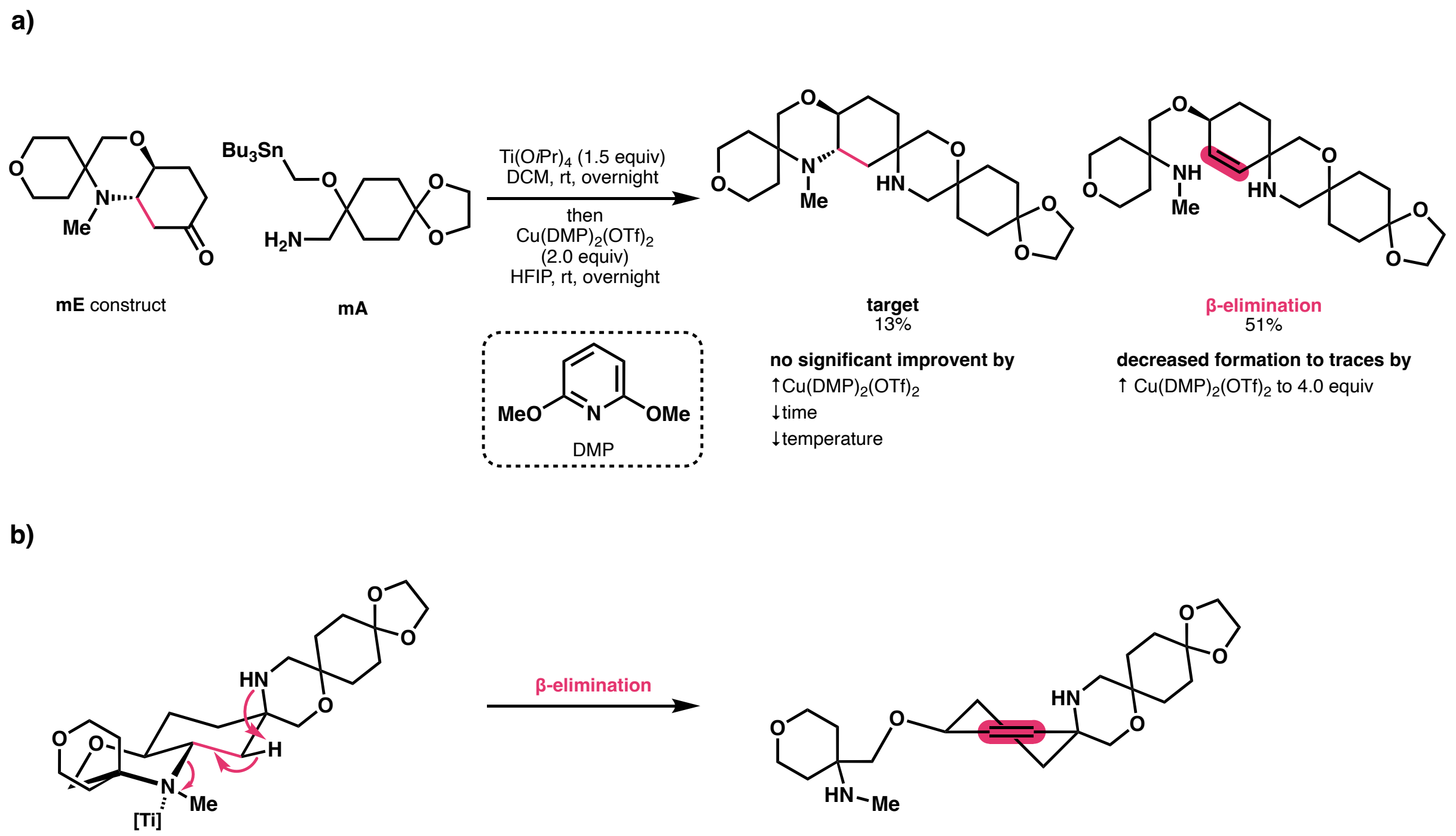

Figure S1: Limitation in forming the pentacycle from the ***mE*** *construct.* ***a)*** *Cyclisation with observed β-elimination byproduct.* ***b)*** *proposed elimination mechanism shown on the example of one of the two diastereomers and role of the Lewis acid.*

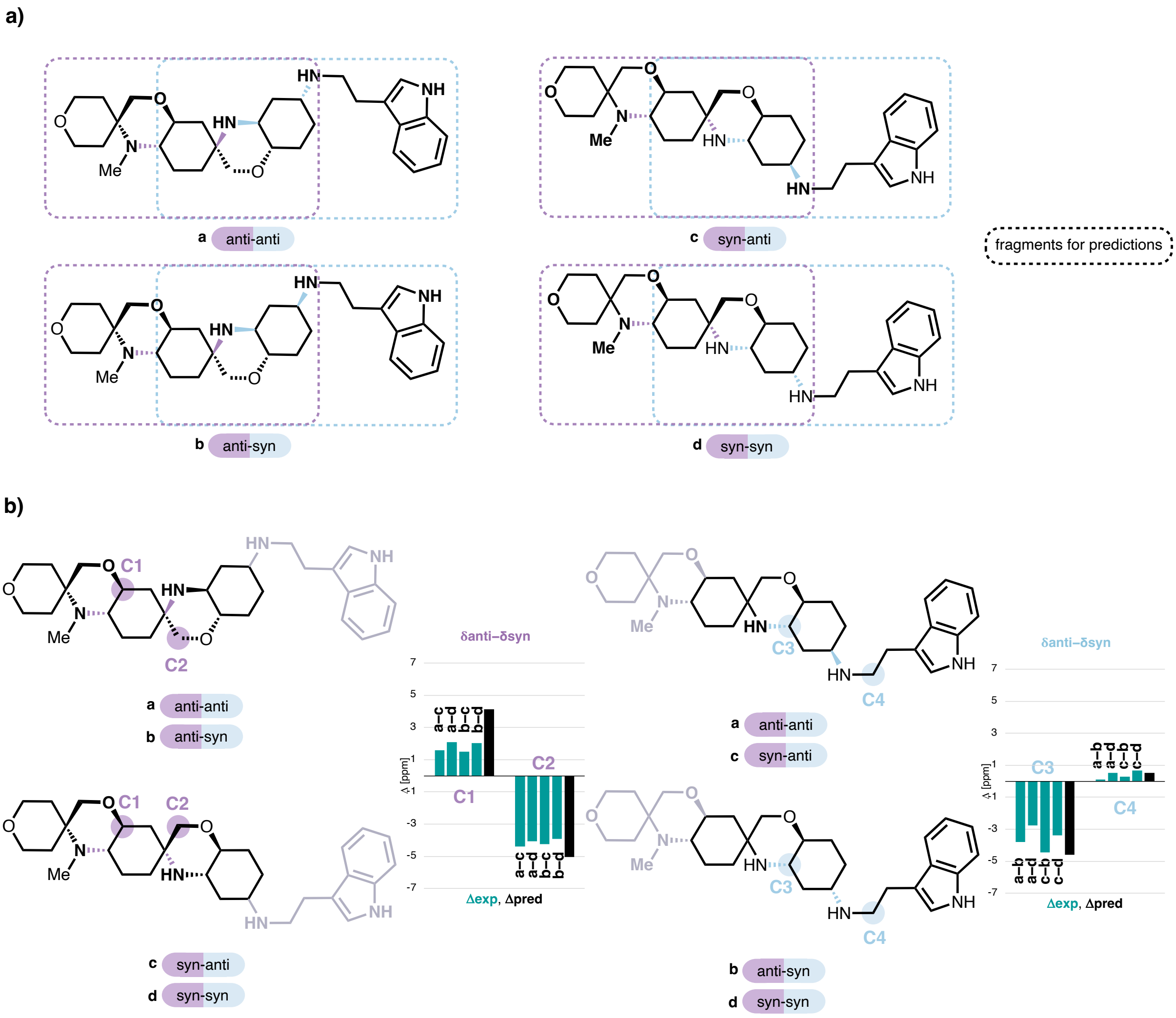

Figure S2: Additional diastereomer assignment example. Comparison of the experimental δ_{13C} differences of two diastereomeric carbons to the corresponding predicted differences. ***a)*** *Example set of 4 possible and accessed diastereomers. Boxes indicate the fragments used for predictions.* ***b)*** *Examples of δ_{13C} differences between diastereomeric carbons rendering the assignment of diastereomers possible. All 4 diastereomer assignments matched their X-ray structures.*

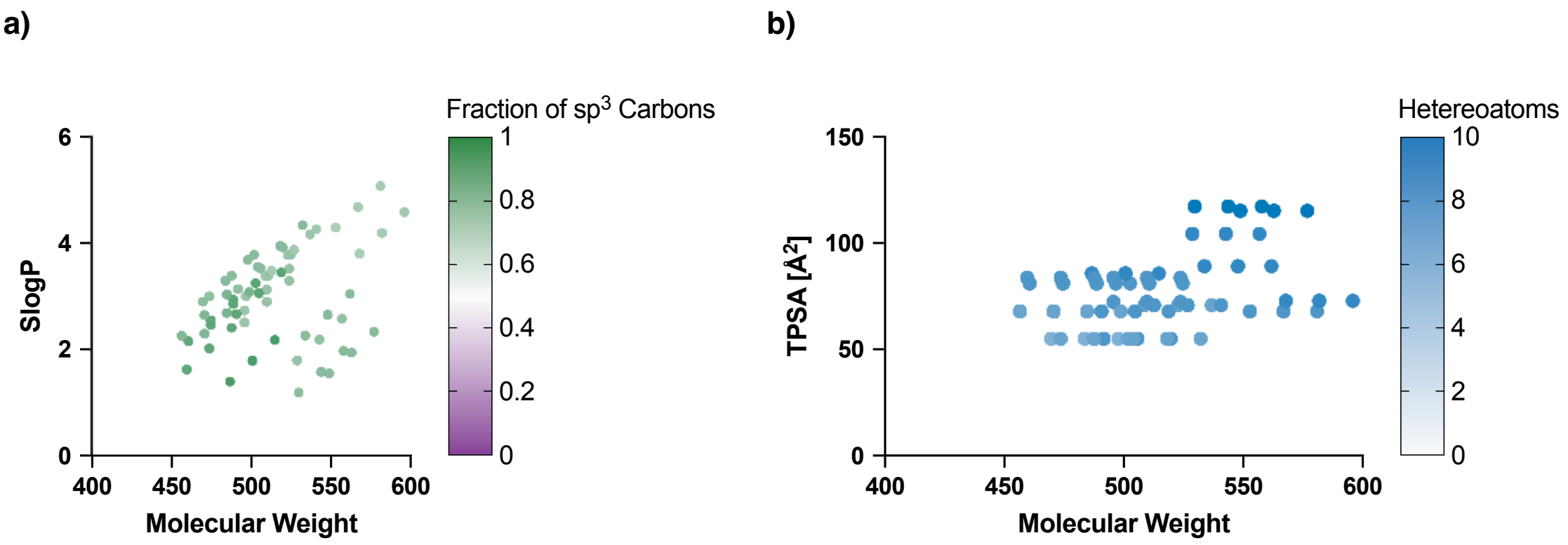

Figure S3: Virtual library characterisitics. ***a)*** *Lipophilicity (SLogP) vs molecular weight with colouring by level of saturation.* ***b)*** *Topological Polar Surface Area (TPSA) vs molecular weight with colouring by number of heteroatoms.*

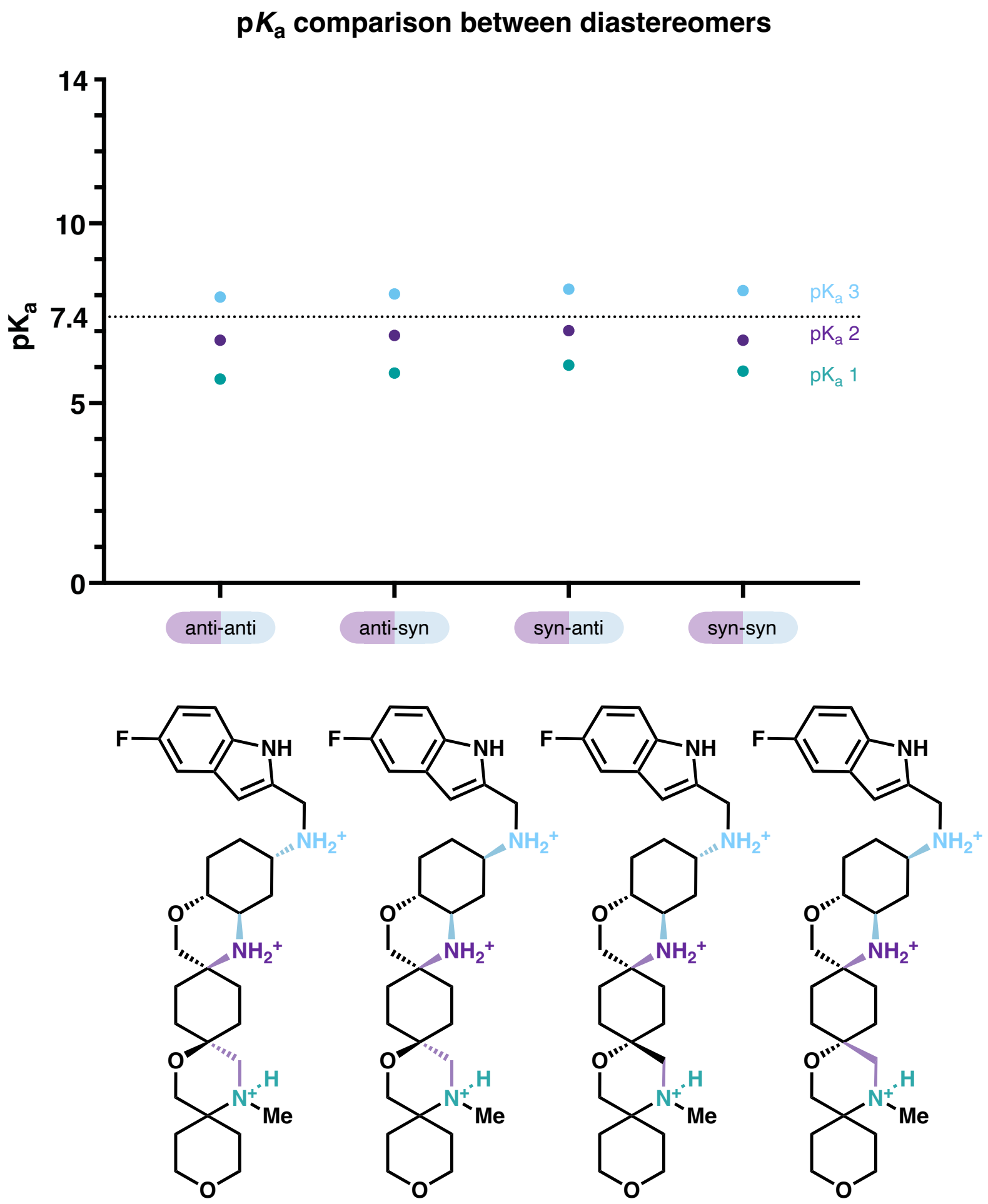

Figure S4: Comparison of pK_a values on an example set of diastereomers.

2 ADME PROFILING

In this section, additional illustrations regarding the building block influence on the ADME properties PAMPA, clearance in human microsomes, clearance in mouse microsomes, CYP3A4 inhibition, CYP2D6 inhibition, CYP2C9 inhibition, LogD and kinetic solubility are shown. Some additional property-to-property relation plots and a dimensionality reduction of selected properties are illustrated. Additionally, the data distributions per property were analyzed and plotted.

2.1 Building block structures

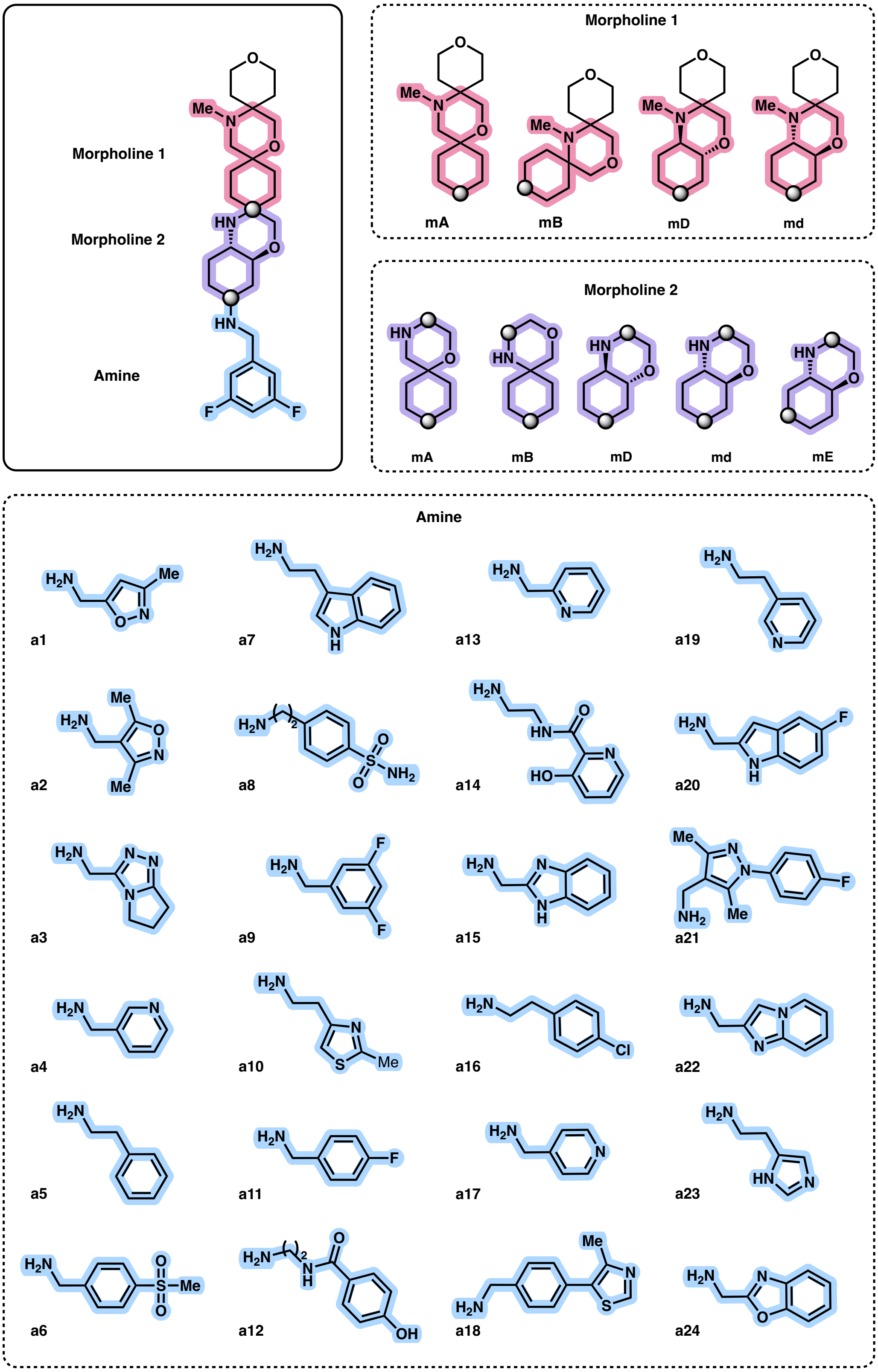

Figure S5: Structures of the incorporated building blocks for Morpholine 1, Morpholine 2 and Amine.

2.2 Permeability

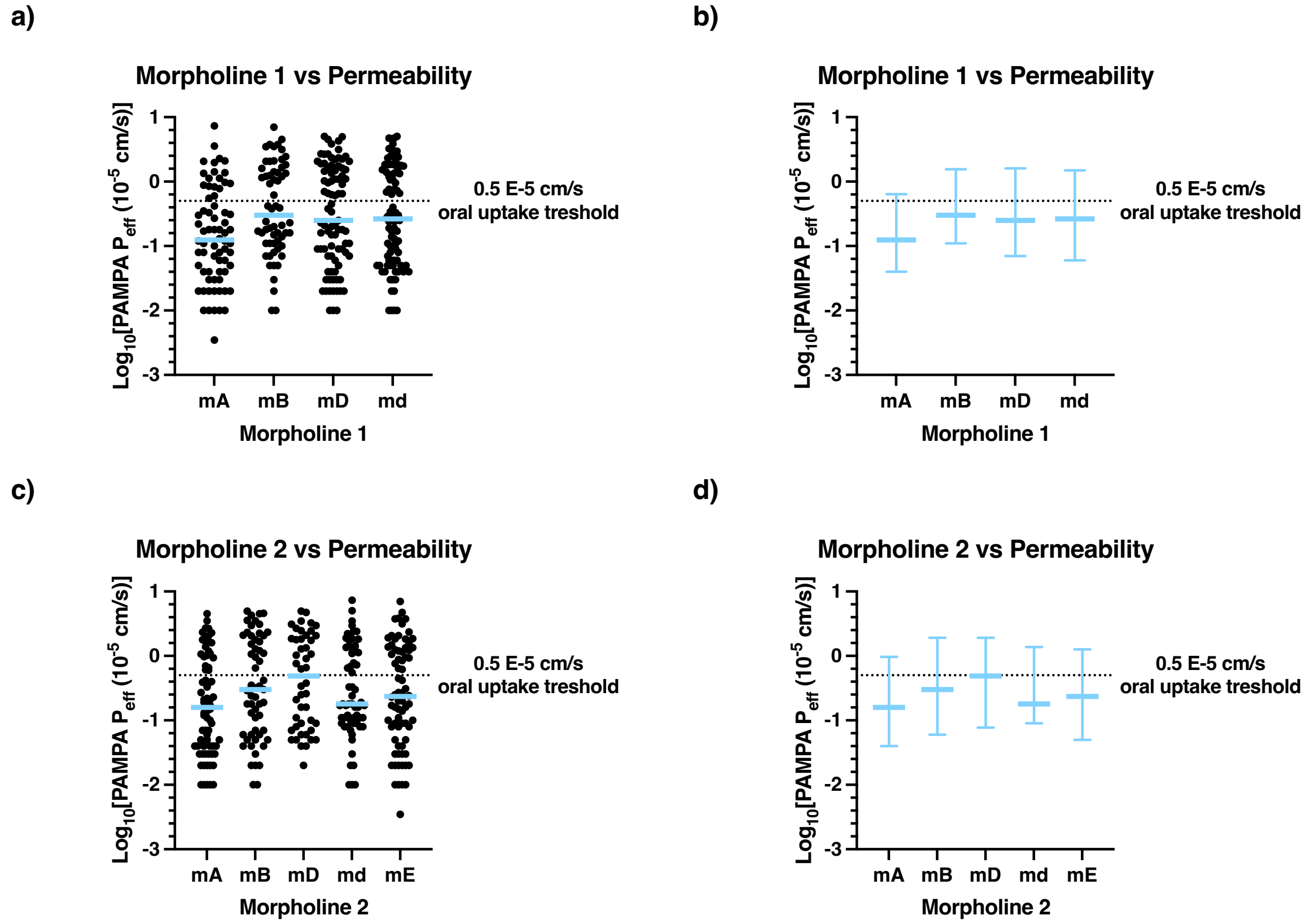

Figure S6: Dependence of permeability on morpholine building block identity. Data points indicate the permeability of diastereomeric mixtures, grouped by Morpholine 1 and Morpholine 2 components. 27 datapoints are omitted (P_{eff} = 0). **a)** *Permeability dependence on the Morpholine 1 position with permeability median per Morpholine 1 building block.* **b)** *Median permeability per Morpholine 1 building block with IQR.* **c)** *Permeability dependence on the Morpholine 2 position with permeability median per Morpholine 1 building block.* **b)** *Median permeability per Morpholine 2 building block with IQR.*

a)

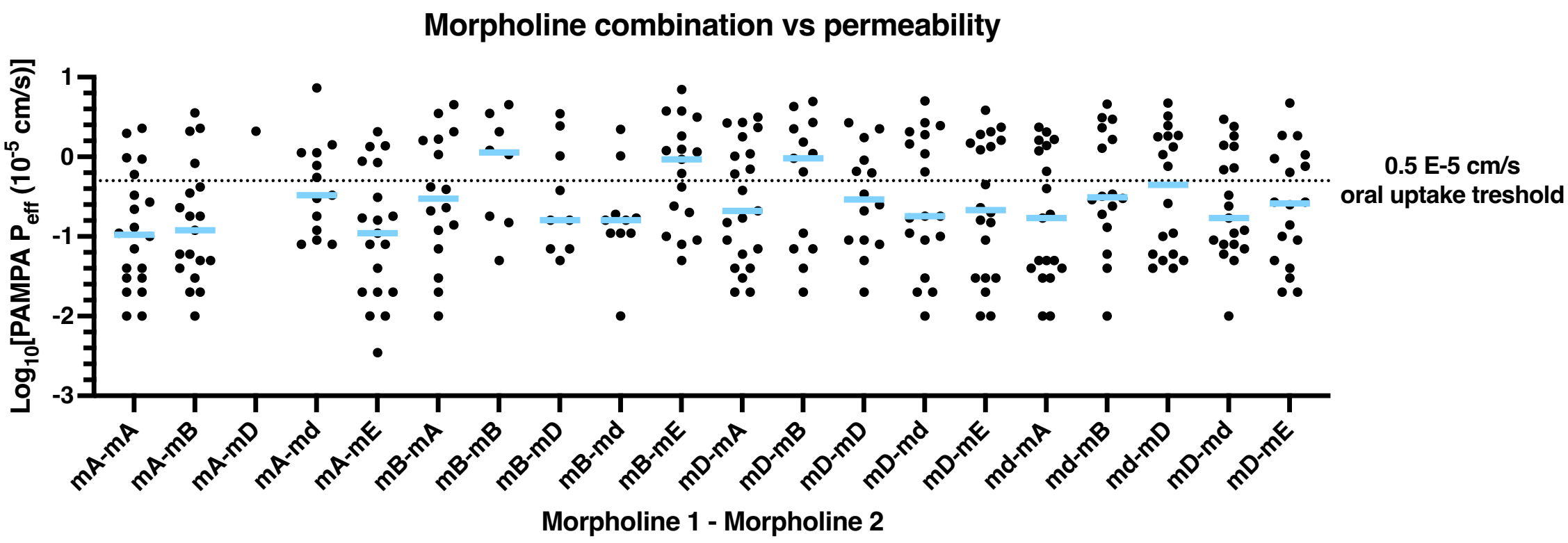

b)

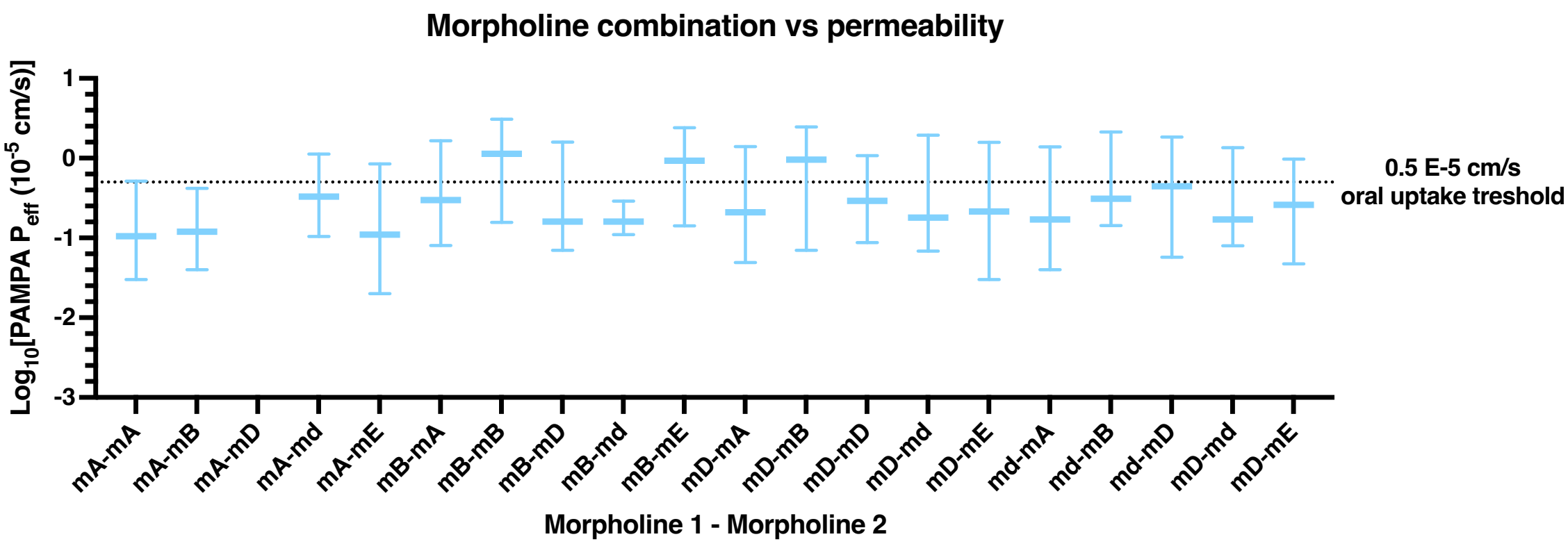

Figure S7: Dependence of permeability on the Morpholine 1 – Morpholine 2 combinations in pentacycles. Data points indicate the permeability of diastereomeric mixtures, grouped by Morpholine 1 – Morpholine 2 combinations. 27 datapoints are omitted ($P_{eff} = 0$). **a)** *Permeability dependence on the Morpholine 1 – Morpholine 2 with permeability median per morpholine building block combination.* **b)** *Median permeability per Morpholine 1 – Morpholine 2 building block combination with IQR.*

a)

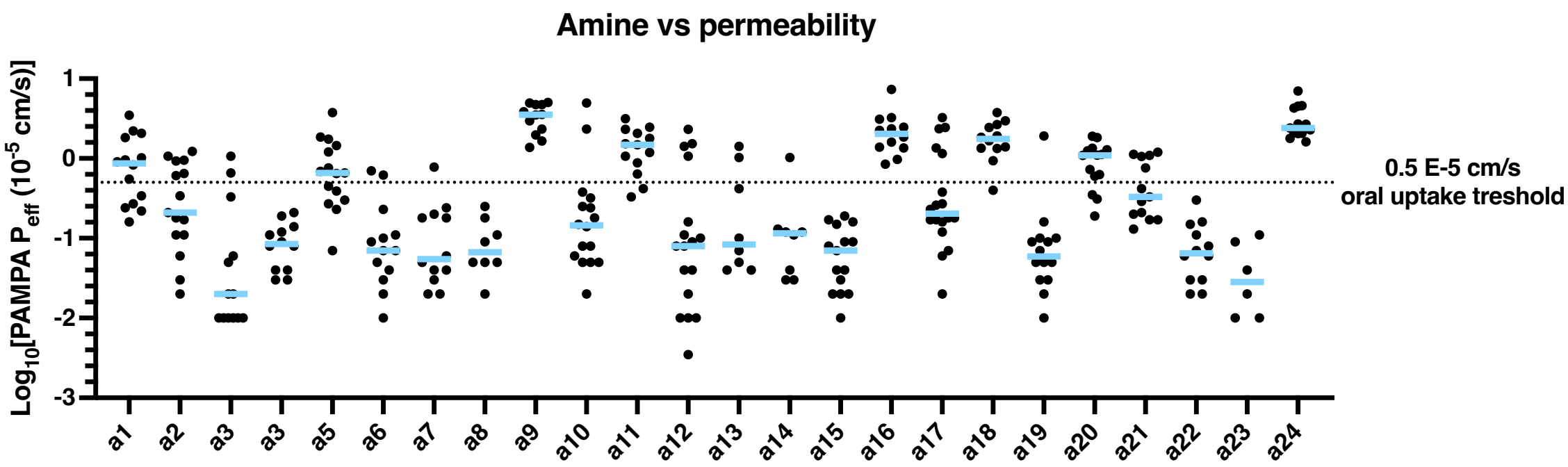

b)

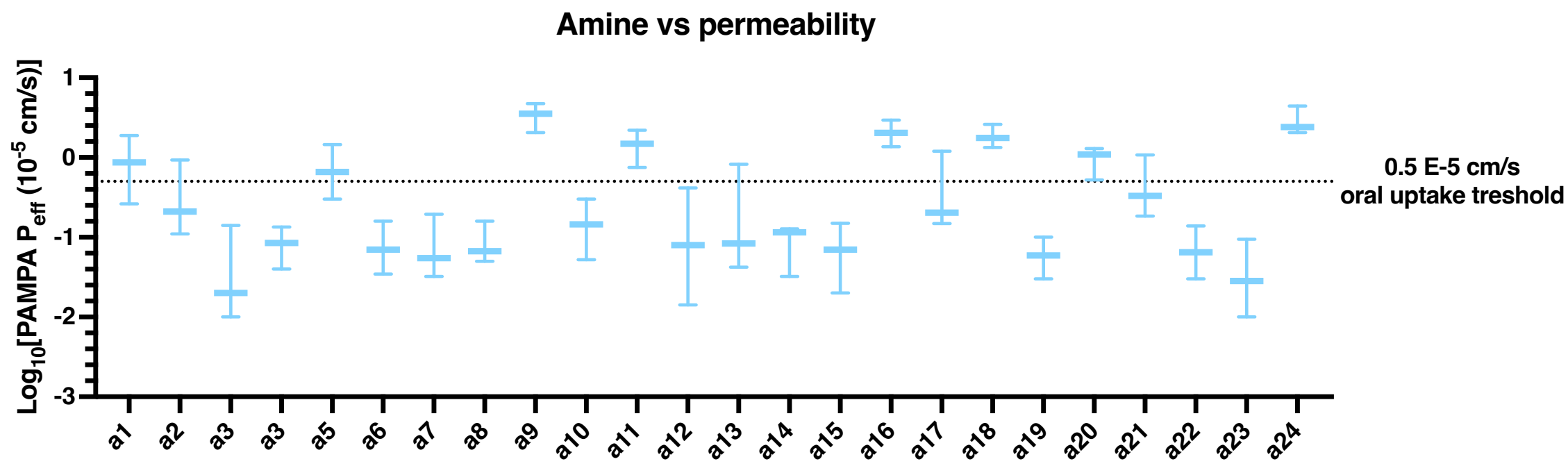

Figure S8: Dependence of permeability on the Amine building blocks. Data points indicate the permeability of diastereomeric mixtures, grouped by Amine building blocks. 27 datapoints are omitted ($P_{eff} = 0$). **a)** *Permeability dependence on the Amine with permeability median per building block.* **b)** *Median permeability per Amine with IQR.*

2.3 Clearance in liver microsomes

2.3.1 Clearance in human liver microsomes

a)

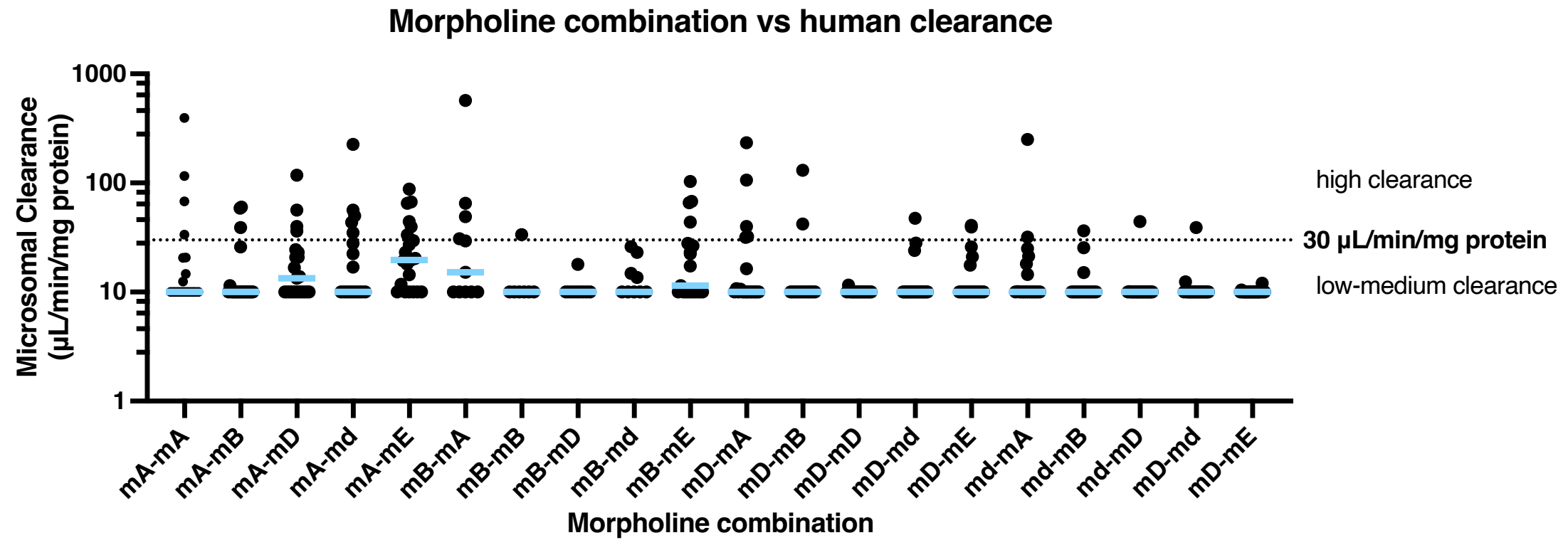

b)

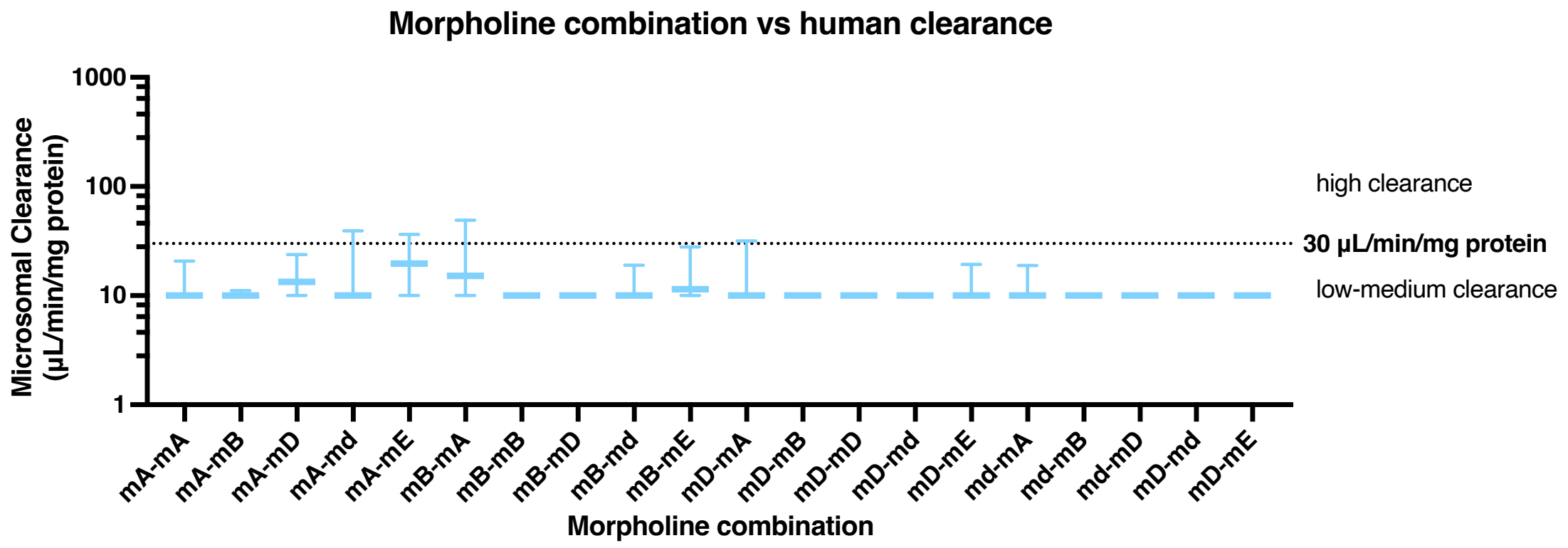

Figure S9: Dependence of clearance in human microsomes on the Morpholine 1 – Morpholine 2 combinations. Data points indicate the clearance of diastereomeric mixtures, grouped by Morpholine 1 – Morpholine 2 combinations. **a)** *Clearance dependence on the Morpholine 1 – Morpholine 2 combination with clearance median per combination.* **b)** *Median clearance per Morpholine 1 – Morpholine 2 combination with IQR.*

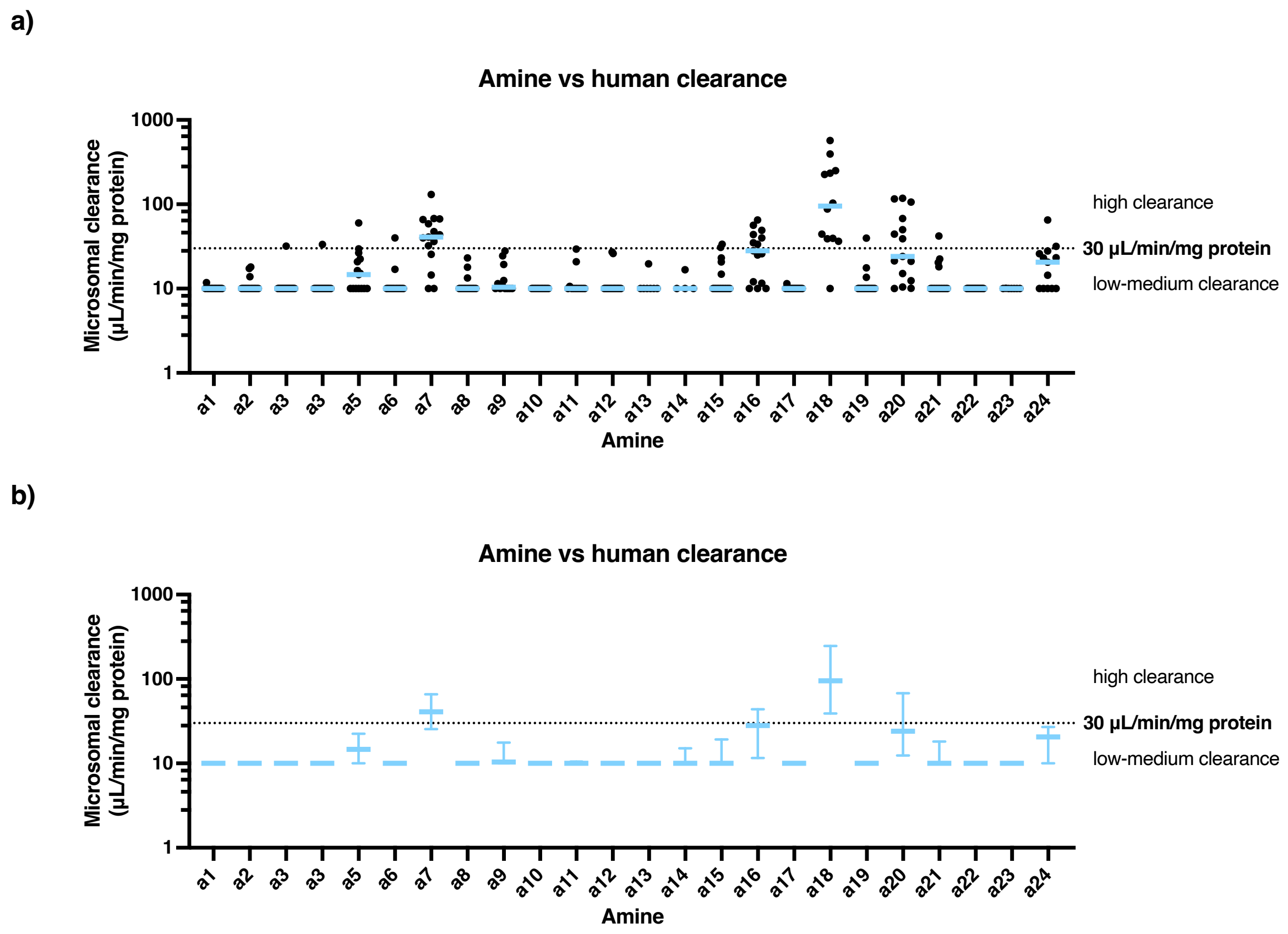

Figure S10: Dependence of clearance in human micrososmes on the Amine building blocks. Data points indicate the clearance of diastereomeric mixtures, grouped by Amine building blocks. **a)** *Clearance dependence on the Amine with clearance median per building block.* **b)** *Median clearance per Amine with IQR.*

2.3.2 Clearance in mouse liver microsomes

a)

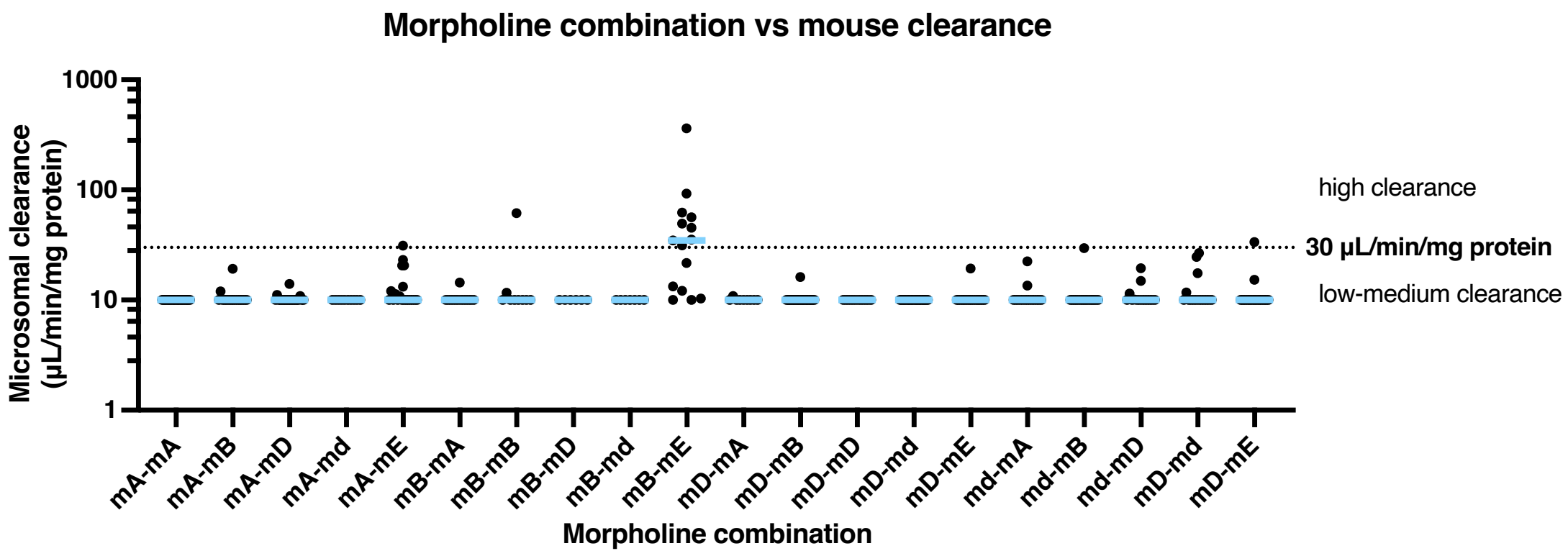

b)

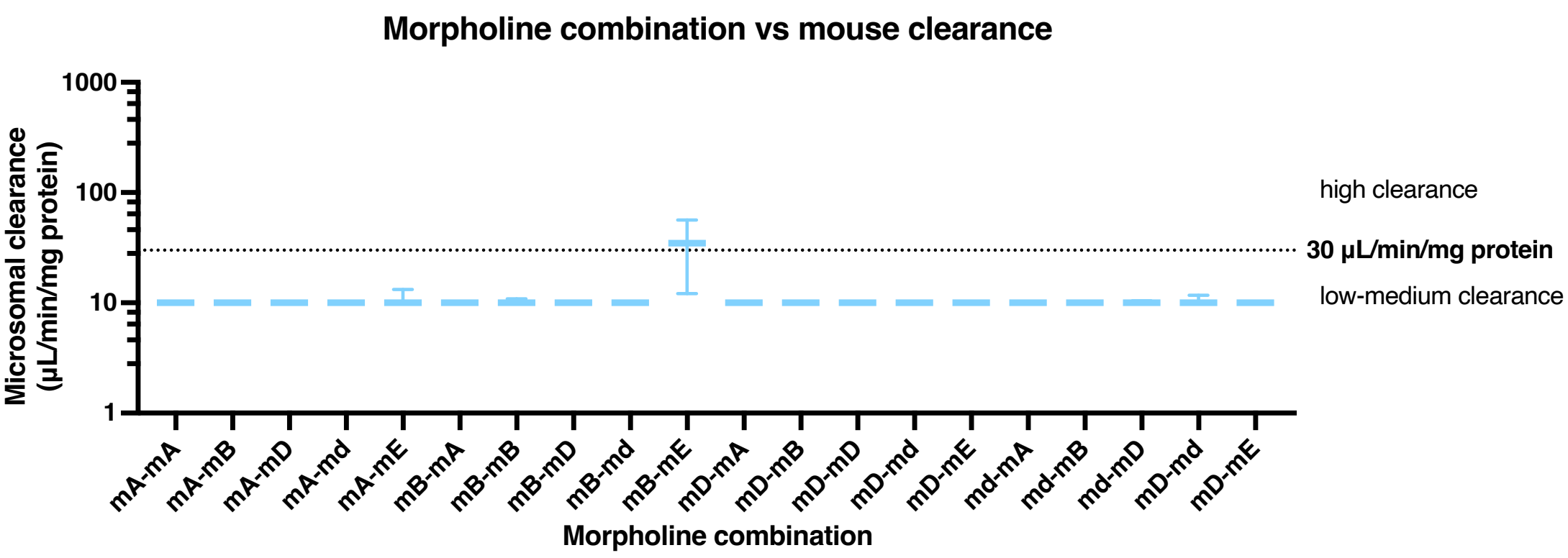

Figure S11: Dependence of clearance in mouse microsomes on the Morpholine 1 – Morpholine 2 combinations. Data points indicate the clearance of diastereomeric mixtures, grouped by Morpholine 1 – Morpholine 2 combinations. **a)** *Clearance dependence on the Morpholine 1 – Morpholine 2 combination with clearance median per combination.* **b)** *Median clearance per Morpholine 1 – Morpholine 2 combination with IQR.*

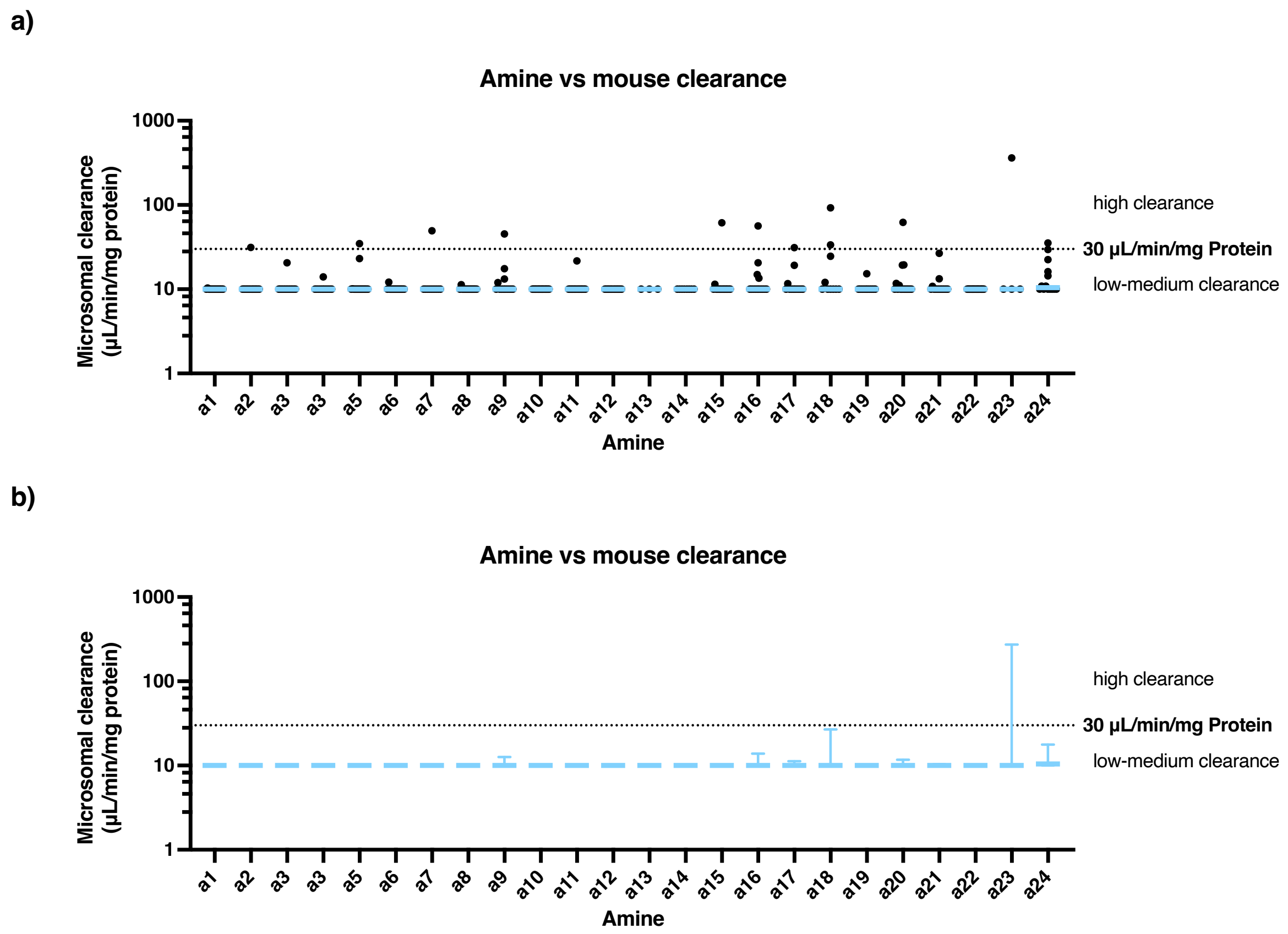

Figure S12: Dependence of clearance in mouse micrososmes on the Amine building blocks. Data points indicate the clearance of diastereomeric mixtures, grouped by Amine building blocks. **a)** *Clearance dependence on the Amine with clearance median per building block.* **b)** *Median clearance per Amine with IQR.*

2.4 CYP inhibition

2.4.1 HLM CYP3A4 inhibition

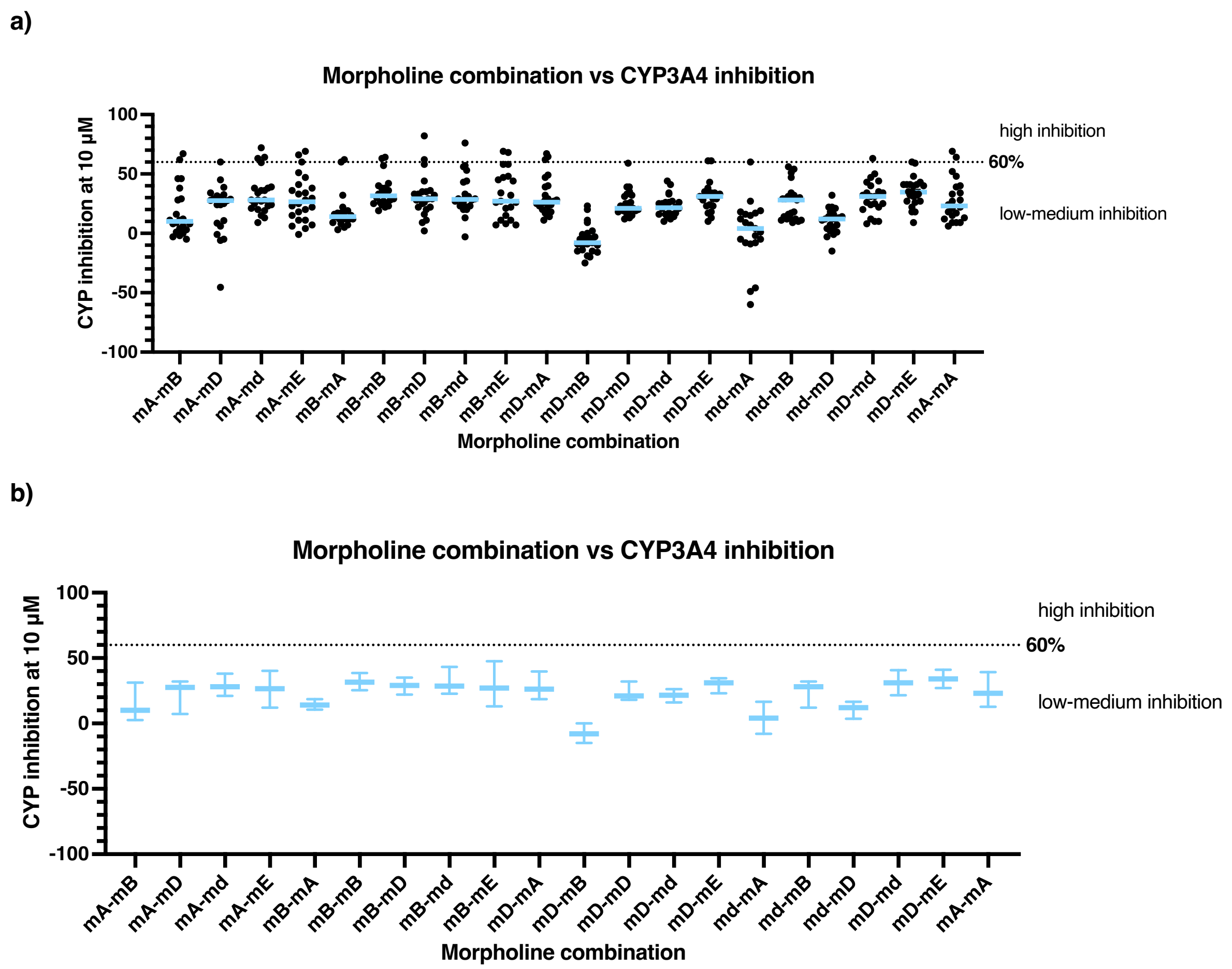

Figure S1: Dependence of HLM CYP3A4 inhibition on the Morpholine 1 – Morpholine 2 combinations. Data points indicate the %inhibition of diastereomeric mixtures, grouped by Morpholine 1 – Morpholine 2 combinations. **a)** *Inhibition dependence on the Morpholine 1 – Morpholine 2 combination with inhibition median per combination.* **b)** *Median inhibition per Morpholine 1 – Morpholine 2 combination with IQR.*

a)

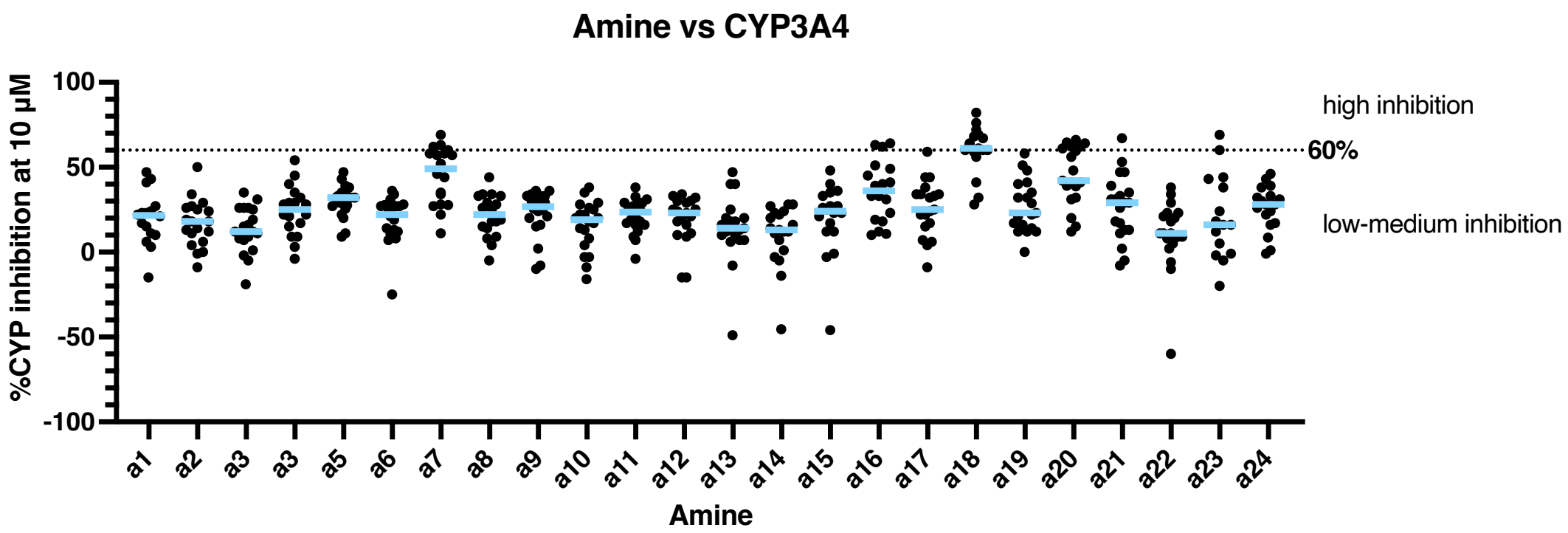

b)

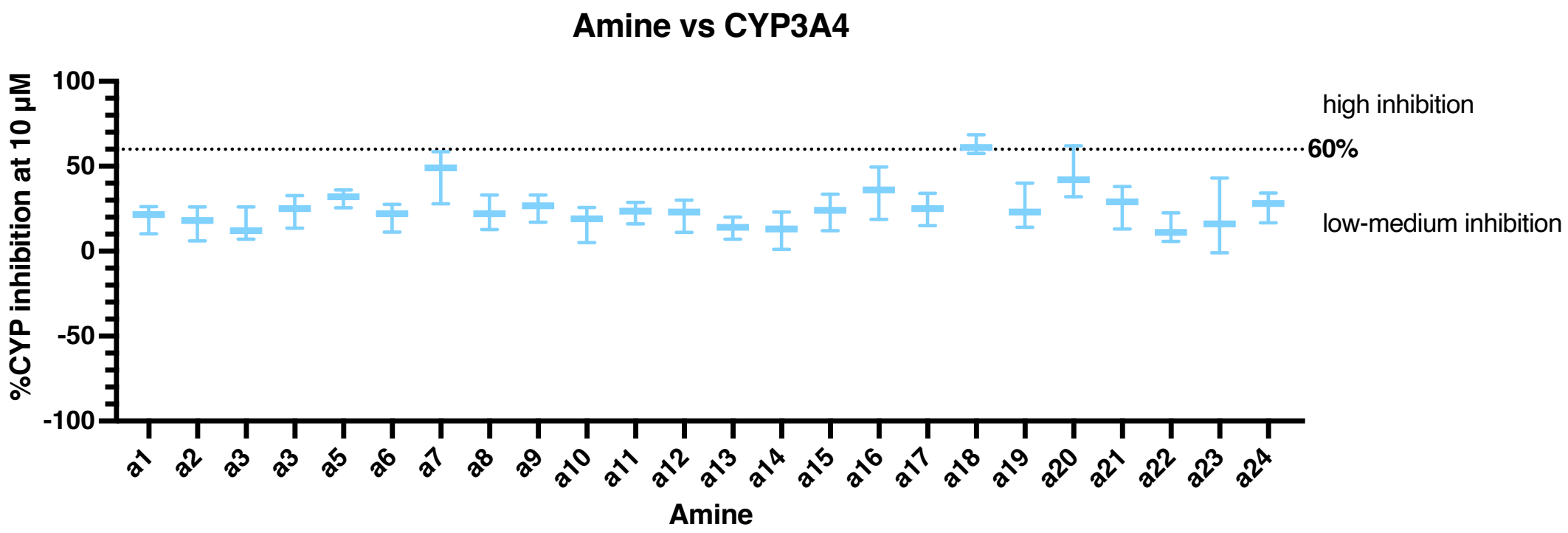

Figure S2: Dependence of HLM CYP3A4 inhibition on the Amine building blocks. Data points indicate the %inhibition of diastereomeric mixtures, grouped by Amine building blocks. **a)** *Inhibition dependence on the Amine with inhibition median per building block.* **b)** *Median inhibition per Amine with IQR.*

2.4.2 HLM CYP2D6 inhibition

a)

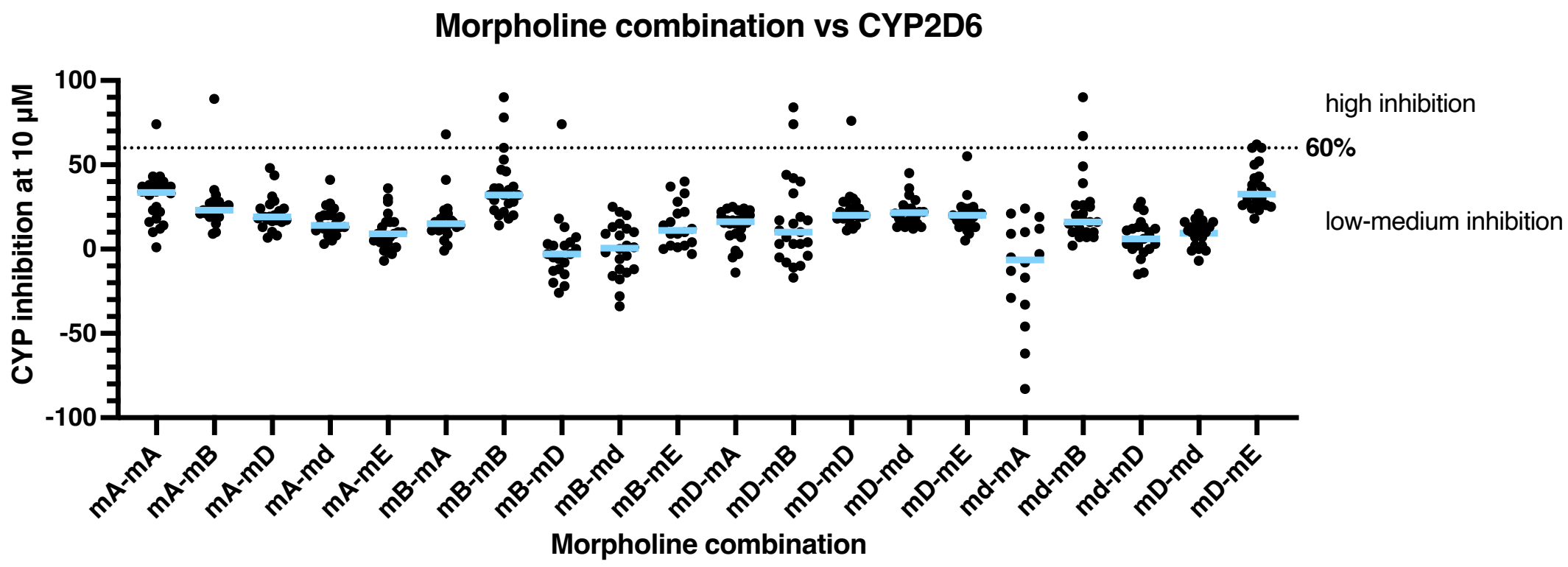

b)

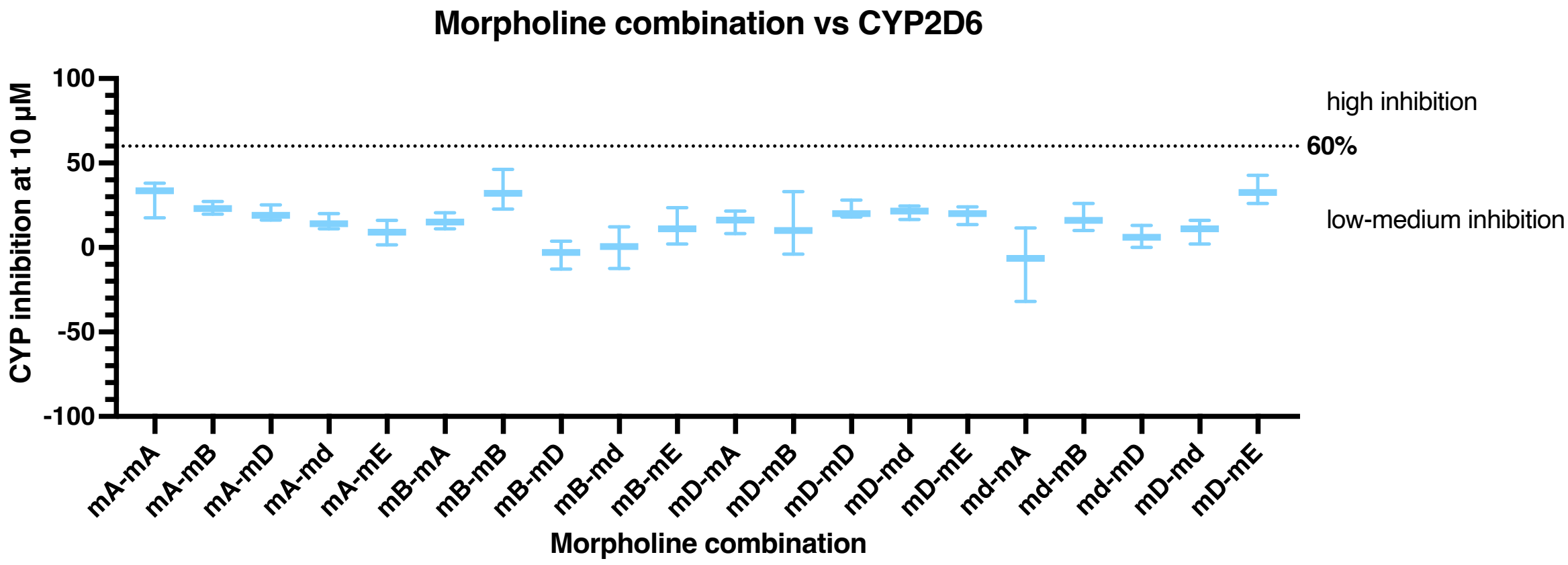

Figure S3: Dependence of HLM CYP2D6 inhibition on the Morpholine 1 – Morpholine 2 combinations. Data points indicate the %inhibition of diastereomeric mixtures, grouped by Morpholine 1 – Morpholine 2 combinations. **a)** *Inhibition dependence on the Morpholine 1 – Morpholine 2 combination with inhibition median per combination.* **b)** *Median inhibition per Morpholine 1 – Morpholine 2 combination with IQR.*

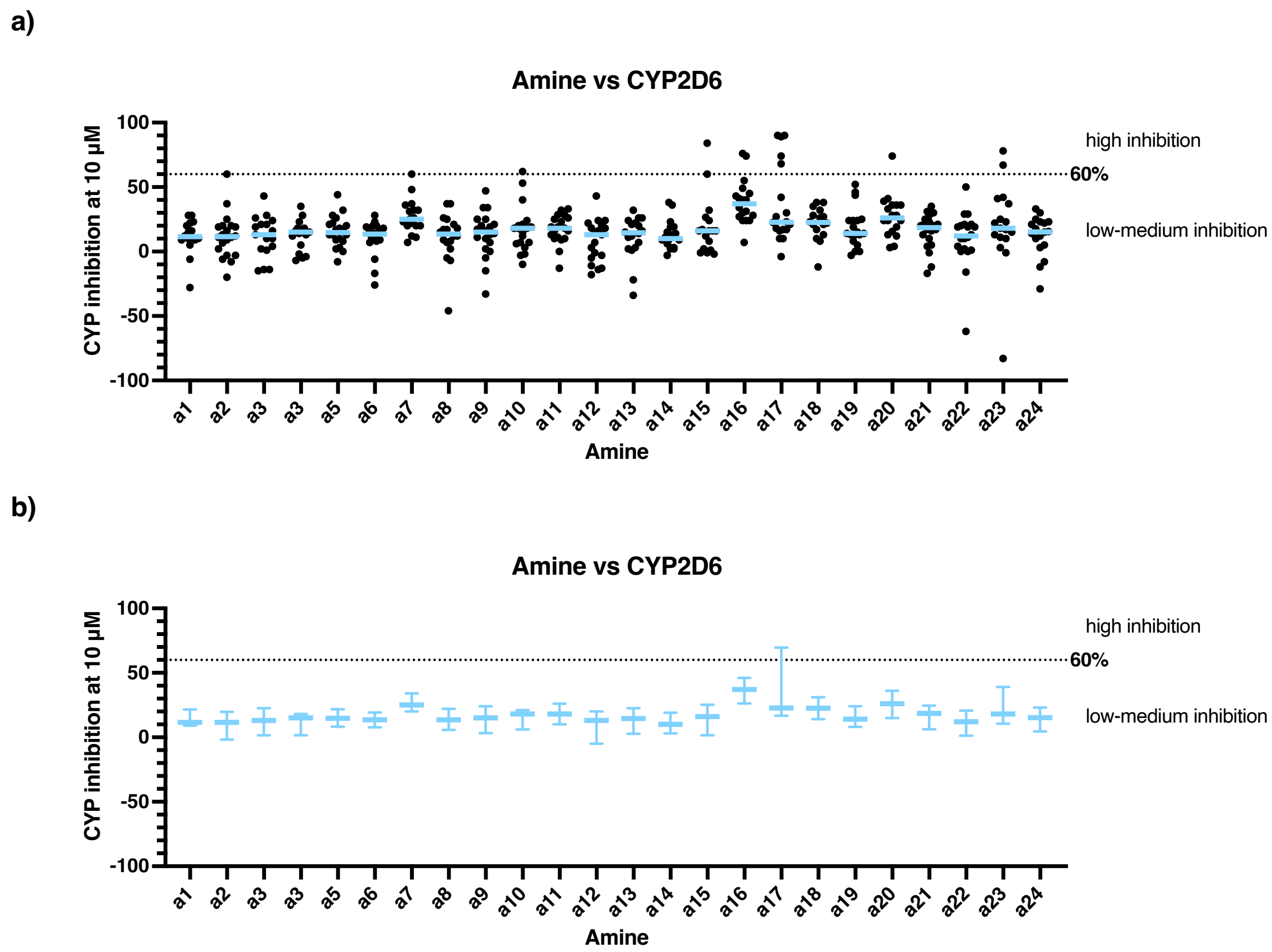

Figure S4: Dependence of HLM CYP2D6 inhibition on the Amine building blocks. Data points indicate the %inhibition of diastereomeric mixtures, grouped by Amine building blocks. **a)** *Inhibition dependence on the Amine with inhibition median per building block.* **b)** *Median inhibition per Amine with IQR.*

2.4.3 HLM CYP2C9 inhibition

a)

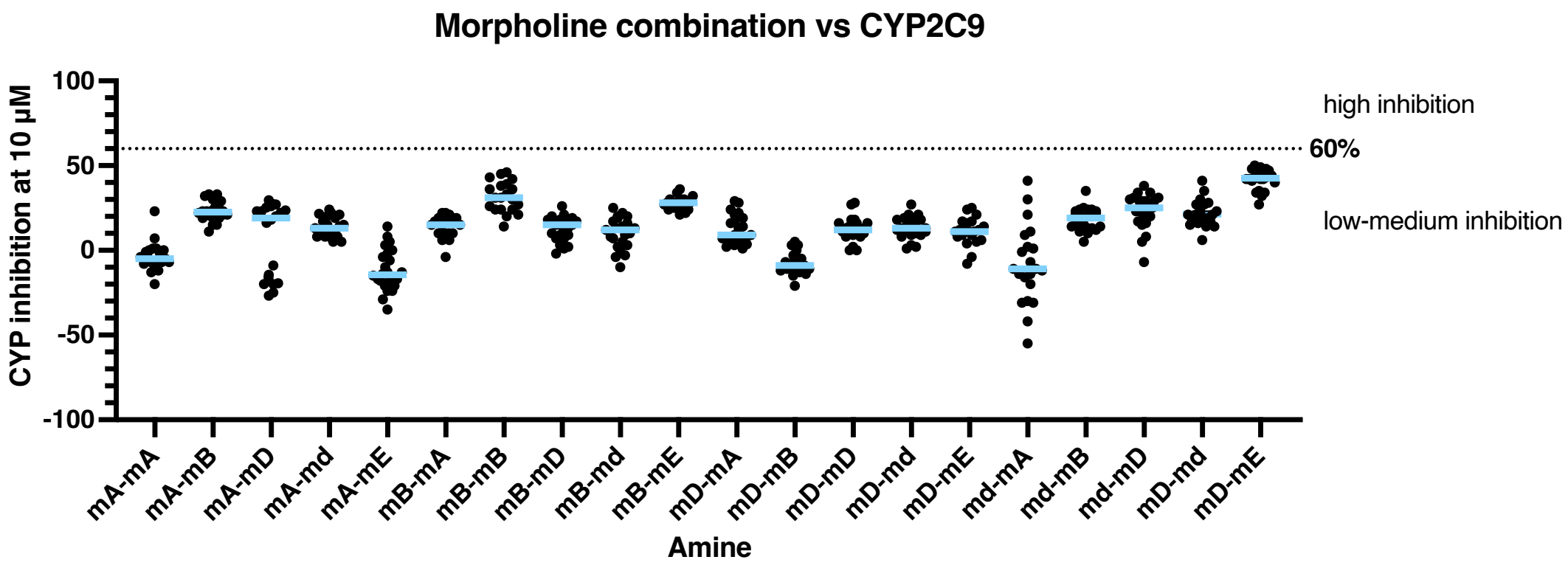

b)

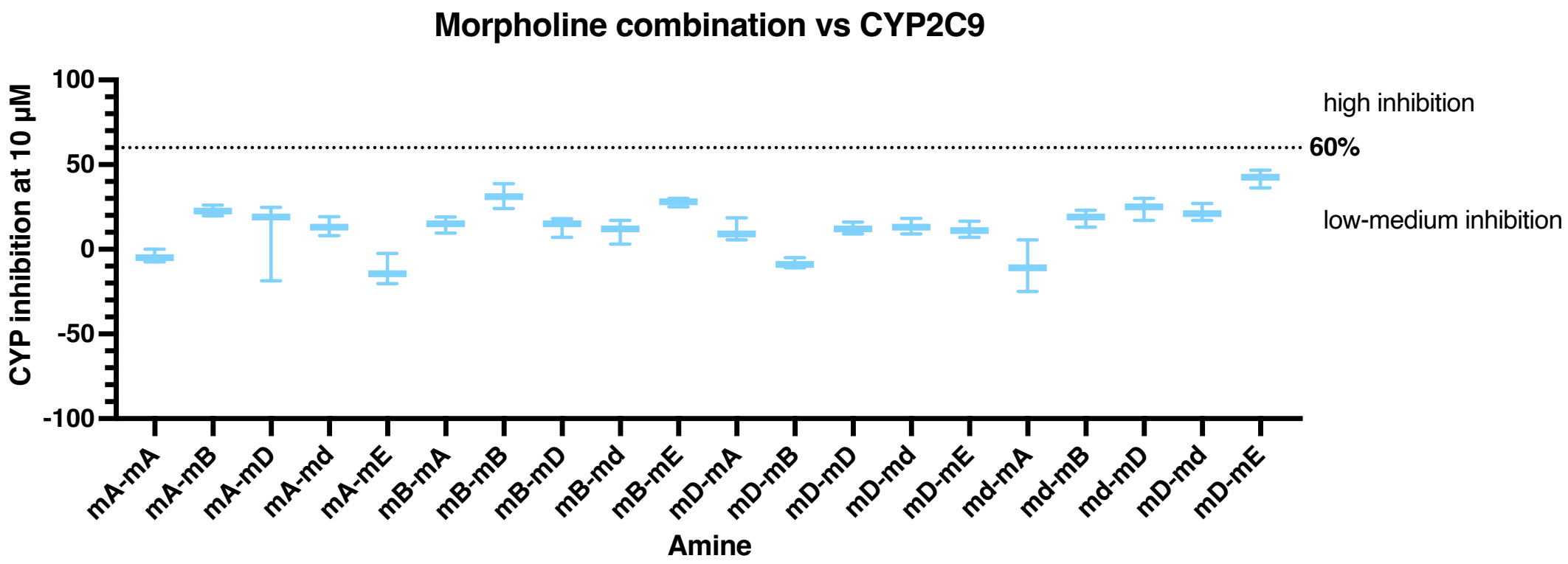

Figure S5: Dependence of HLM CYP2C9 inhibition on the Morpholine 1 – Morpholine 2 combinations. Data points indicate the %inhibition of diastereomeric mixtures, grouped by Morpholine 1 – Morpholine 2 combinations. **a)** *Inhibition dependence on the Morpholine 1 – Morpholine 2 combination with inhibition median per combination.* **b)** *Median inhibition per Morpholine 1 – Morpholine 2 combination with IQR.*

a)

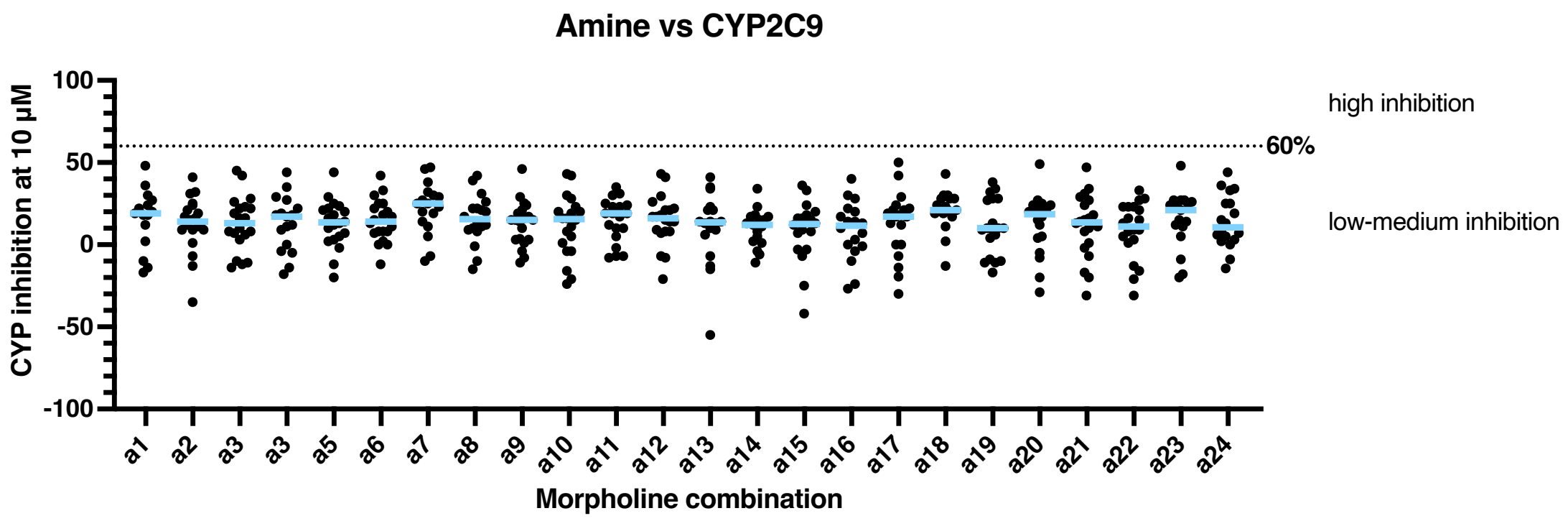

b)

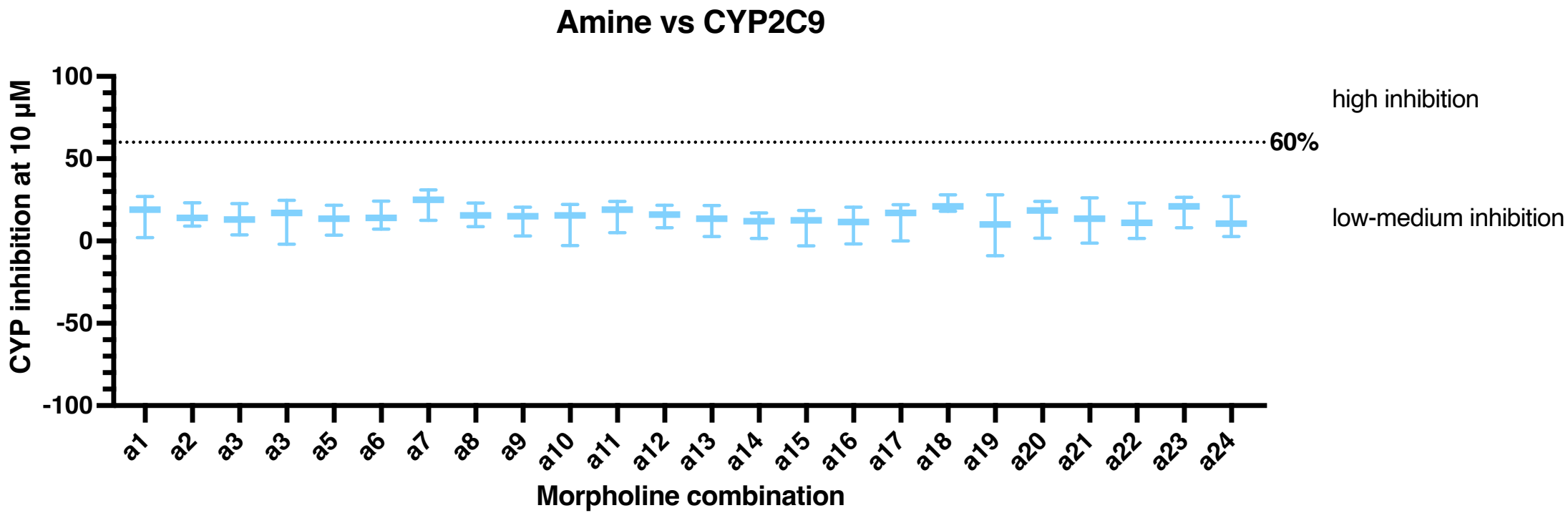

Figure S6: Dependence of HLM CYP2C9 inhibition on the Amine building blocks. Data points indicate the %inhibition of diastereomeric mixtures, grouped by Amine building blocks. **a)** *Inhibition dependence on the Amine with inhibition median per building block.* **b)** *Median inhibition per Amine with IQR.*

2.5 Additional relations between properties

a)

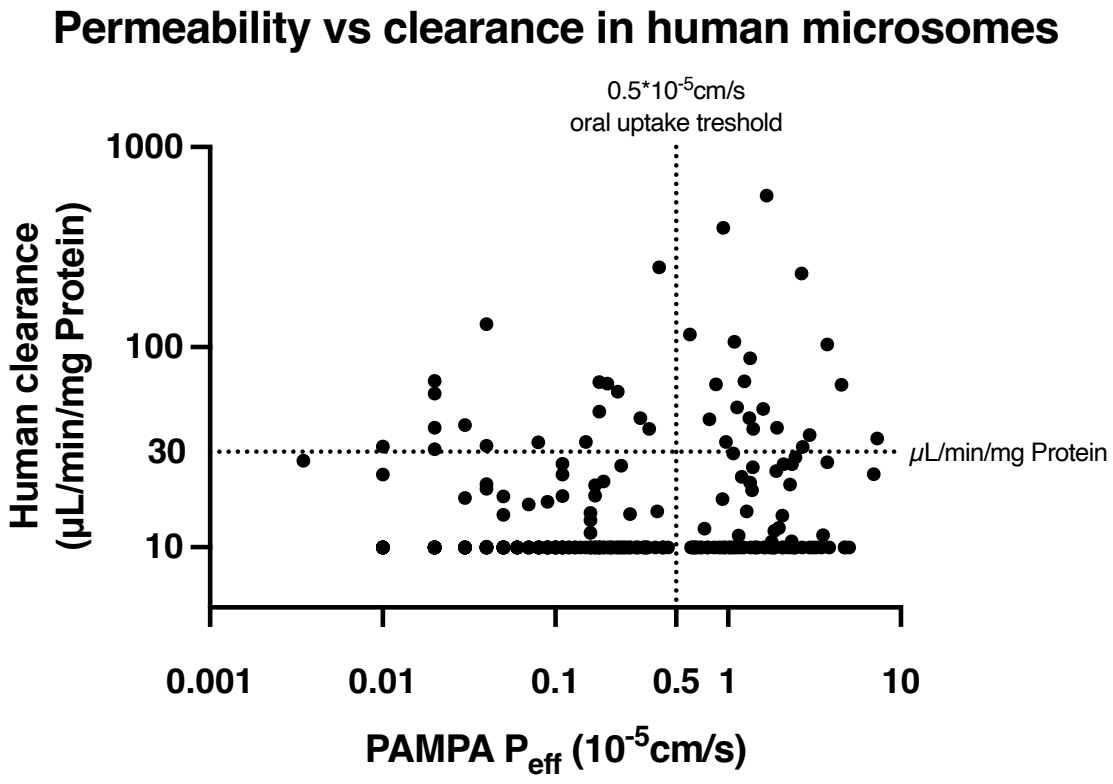

b)

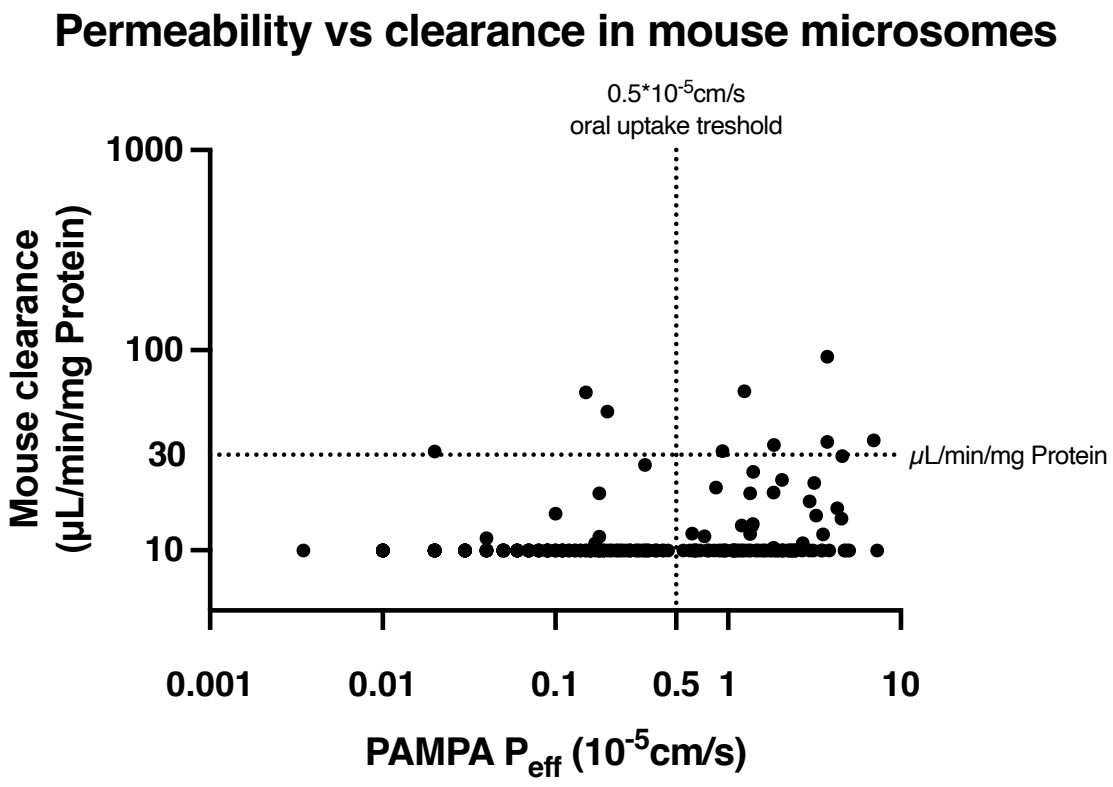

Figure S1: Datapoints represent diastereomeric mixtures. **a)** *Relation of human clearance to permeability.* **b)** *Relation of mouse clearance to permeability. 27 datapoints are omitted (P_{eff} = 0).*

a)

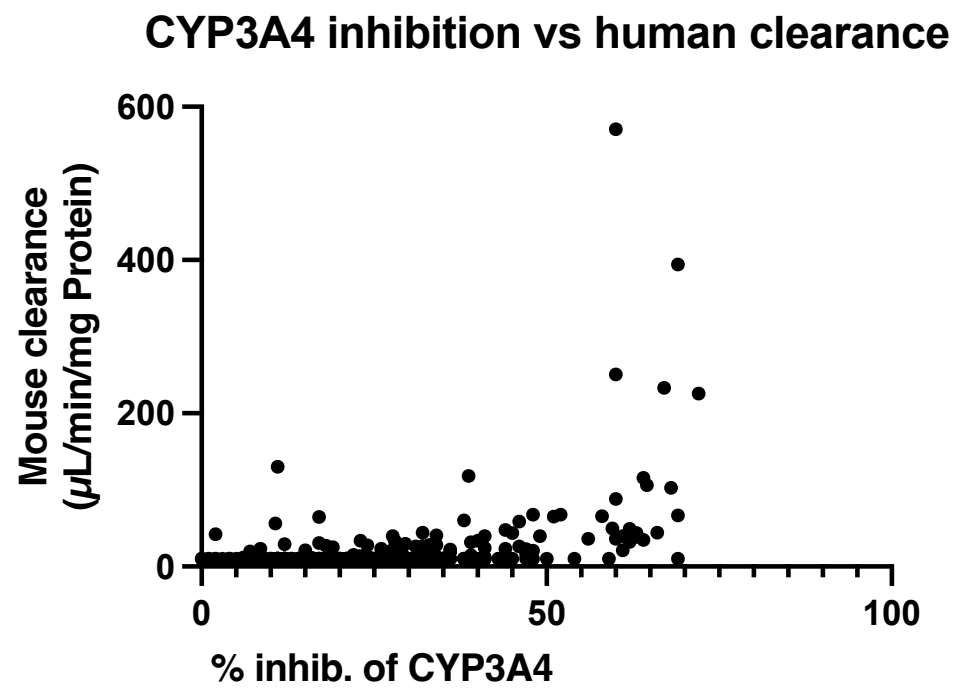

b)

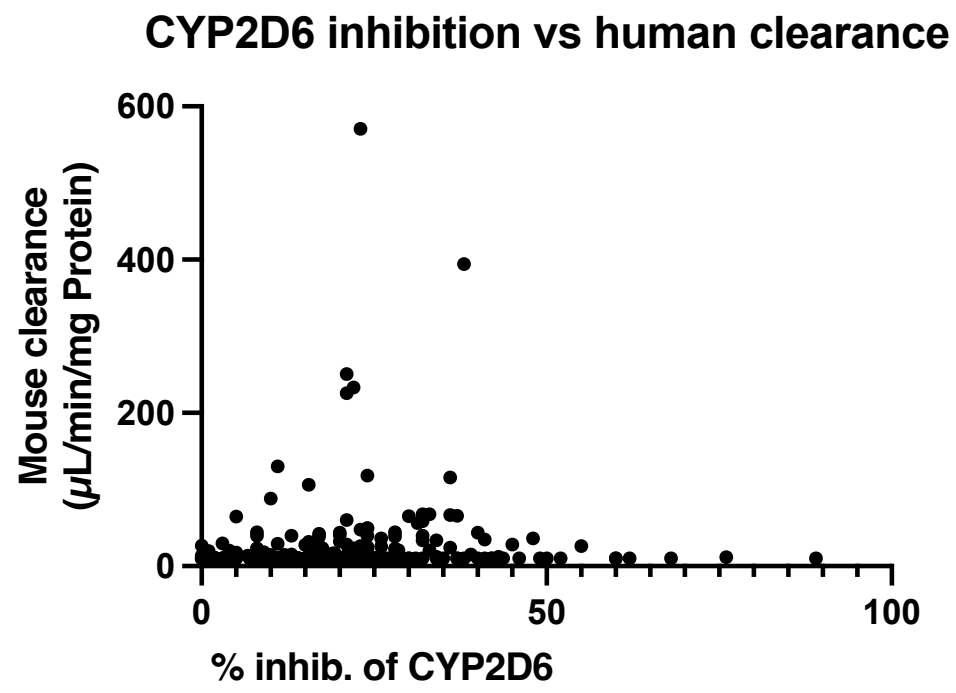

c)

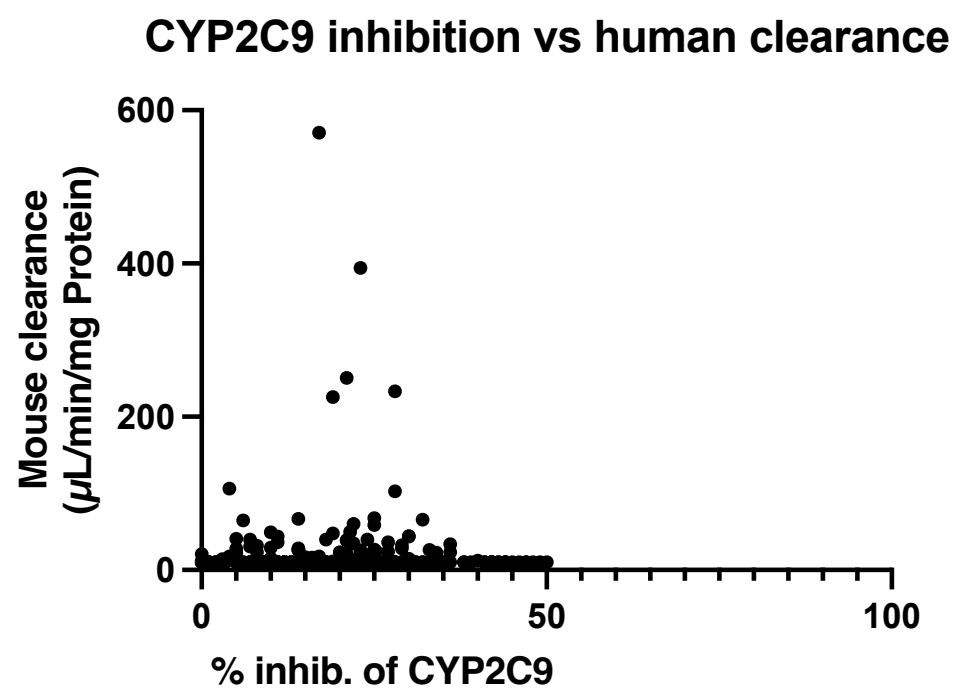

Figure S2: Datapoints represent diastereomeric mixtures. **a)** *Relation of human clearance to HLM CYP3A4 %inhibition.* **b)** *Relation of human clearance to HLM CYP2D6 %inhibition.* **c)** *Relation of human clearance to HLM CYP2C9 %inhibition. CYP inhibitions <0% are ommitted.*

2.5.1 Data distributions

2.5.1.1 Data availability

Parameter	Data availability (%)
Kinetic solubility	71
Lipophilicity (logD)	35
Permeability PAMPA Peff	66
Clearance in human microsomes	67
Clearance in mouse microsomes	61
HLM CYP3A4 inhibition	92
HLM CYP2D6 inhibition	90
HLM CYP2C9 inhibition	92

2.5.1.2 Data distribution histograms

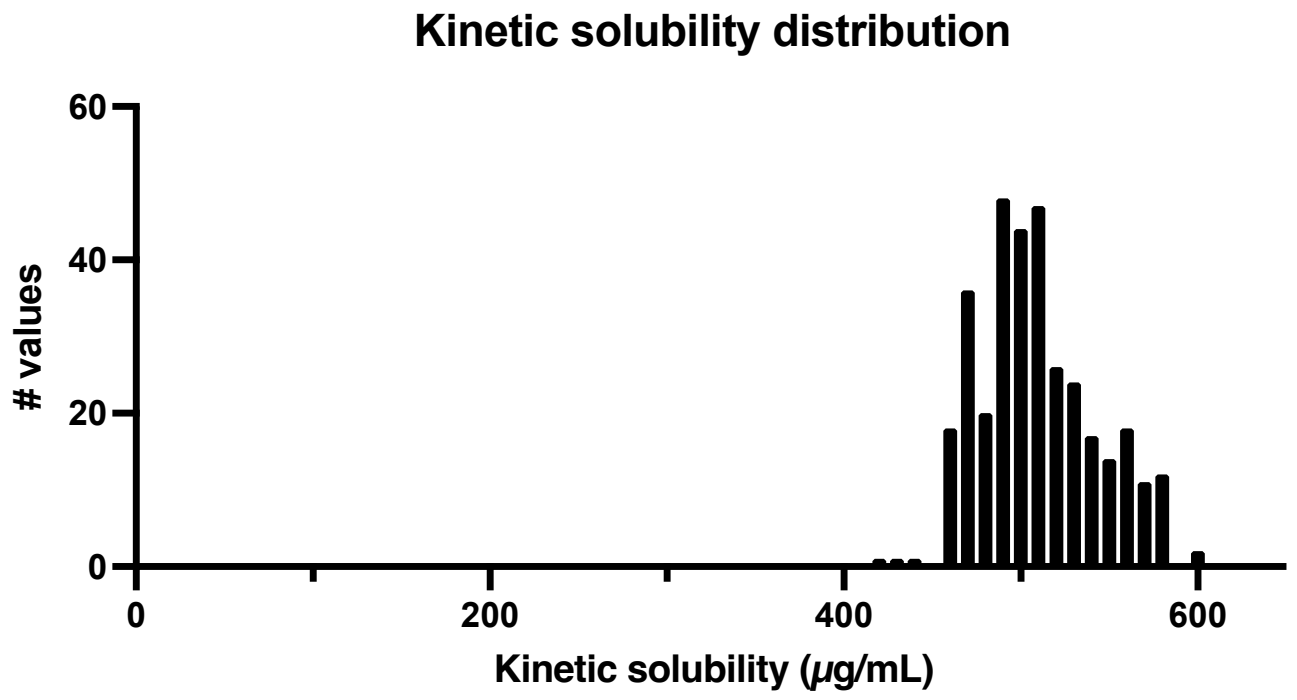

Figure S3: Frequency distribution of the library's aqueous kinetic solubility (pH 6.5).

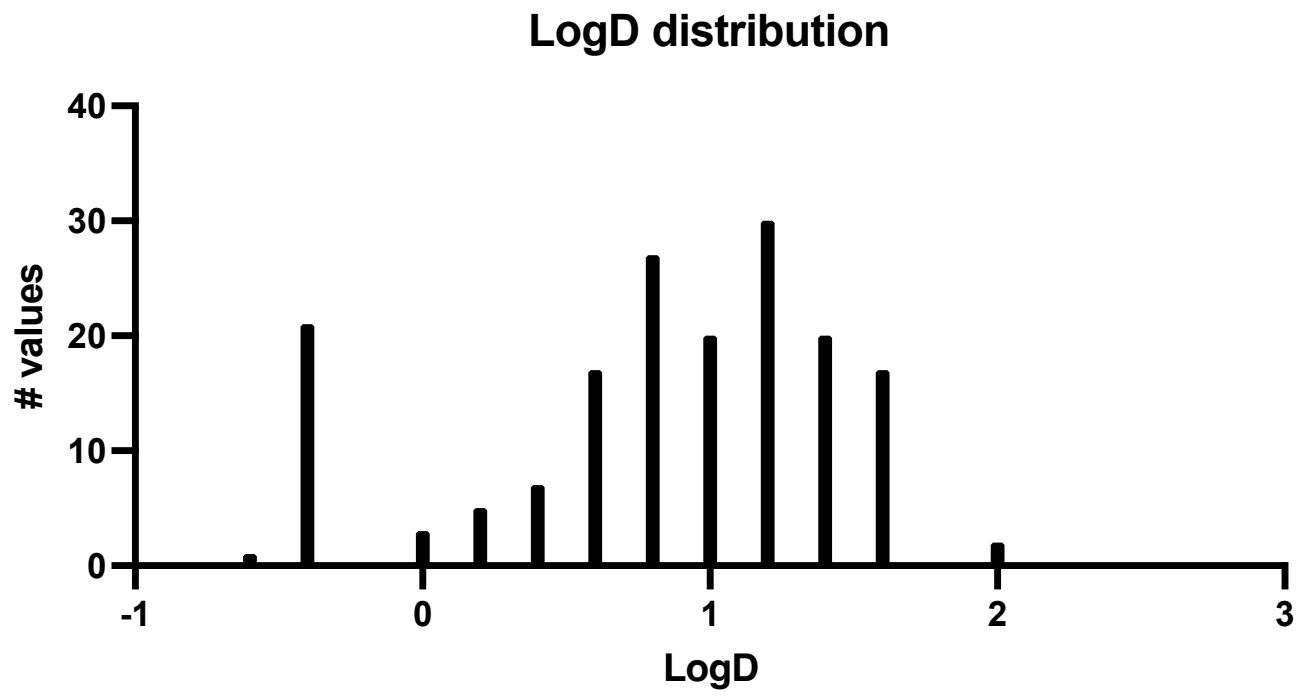

Figure S4: Frequency distribution of the library's lipophilicity (LogD).

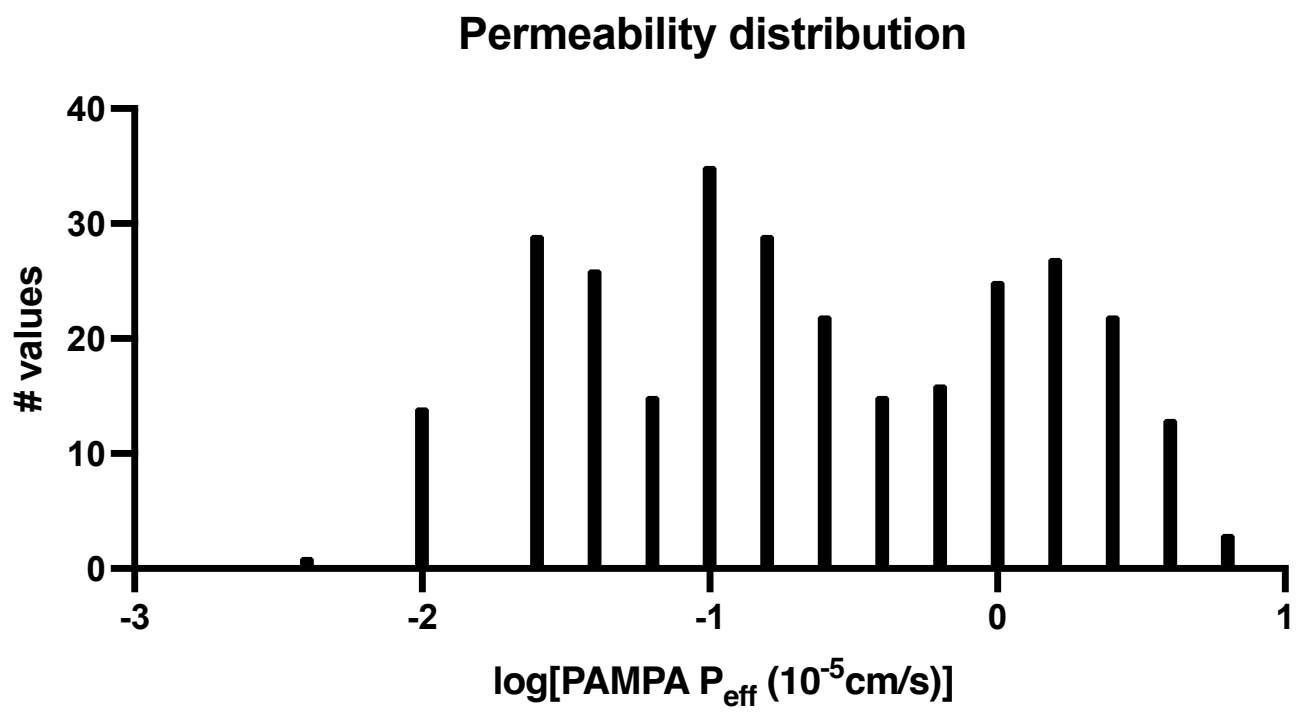

Figure S5: Frequency distribution of the library's permeabililty (PAMPA).

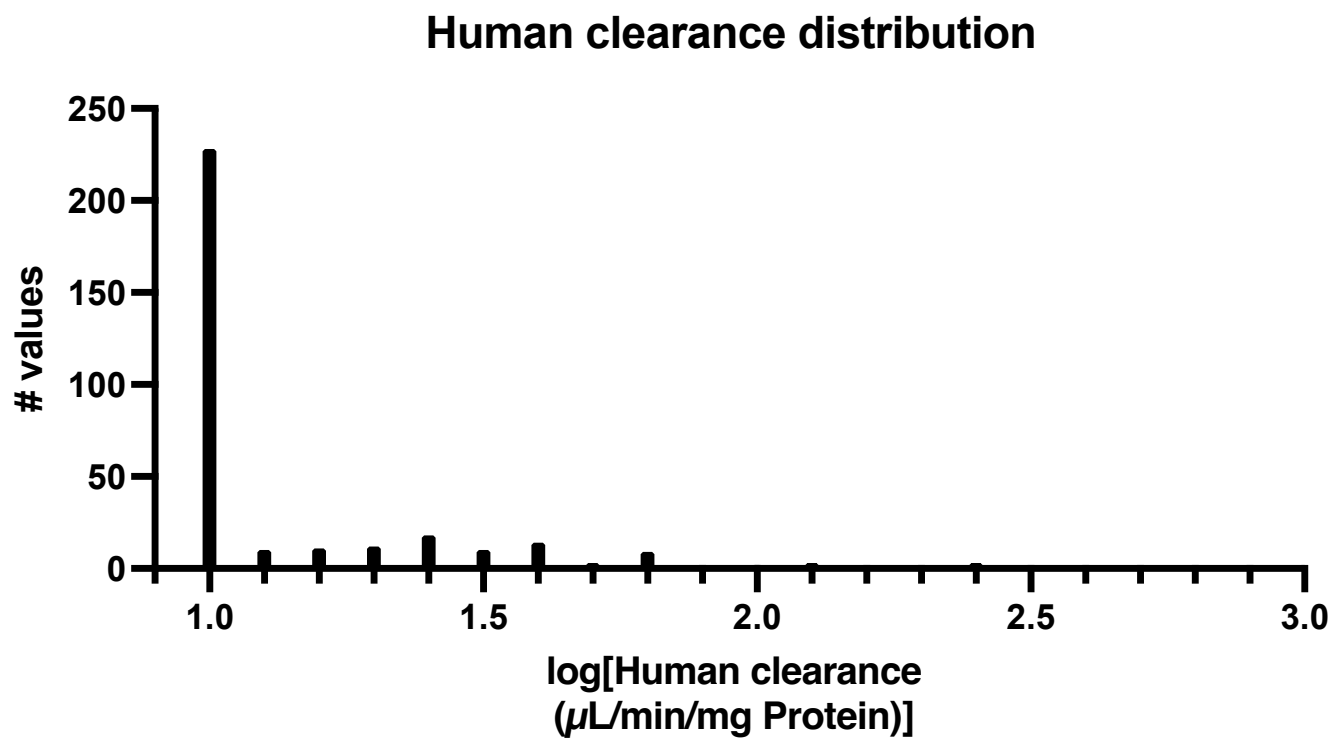

Figure S6: Frequency distribution of the library's clearance in human liver microsomes.

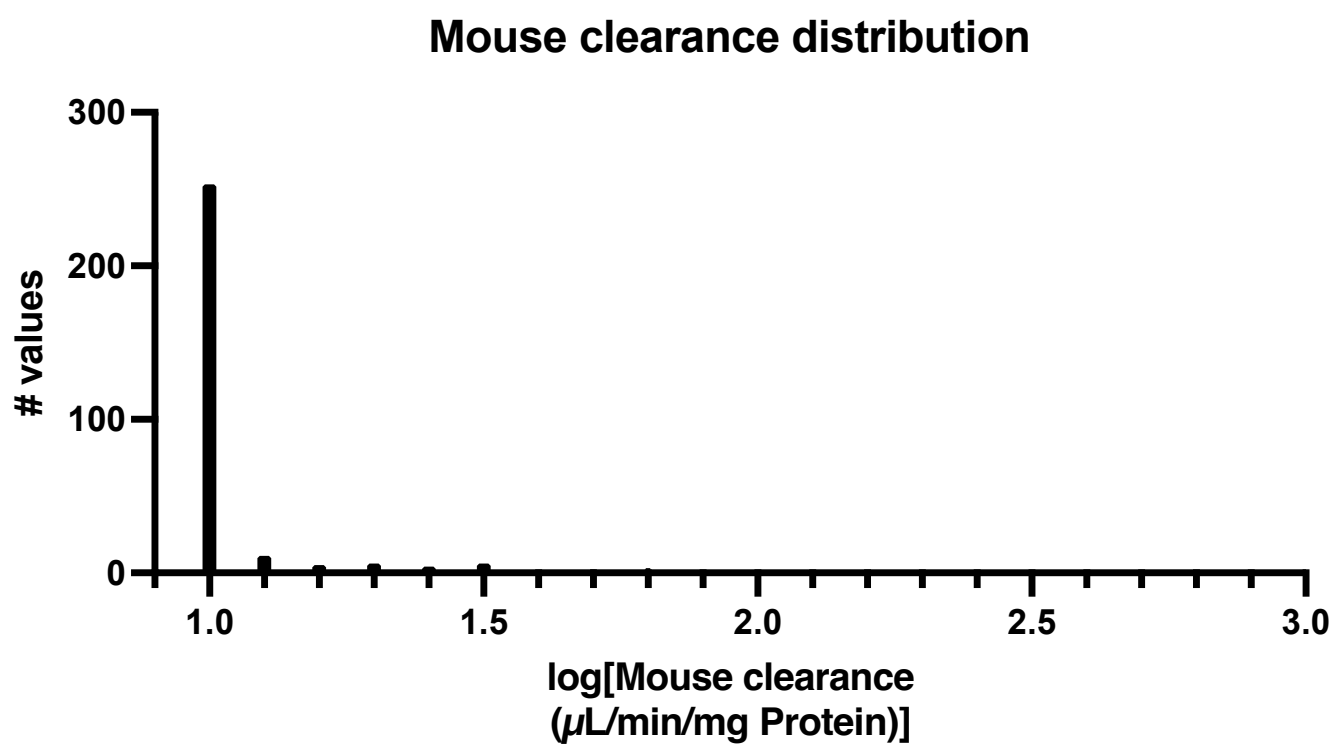

Figure S7: Frequency distribution of the library's clearance in mouse liver microsomes.

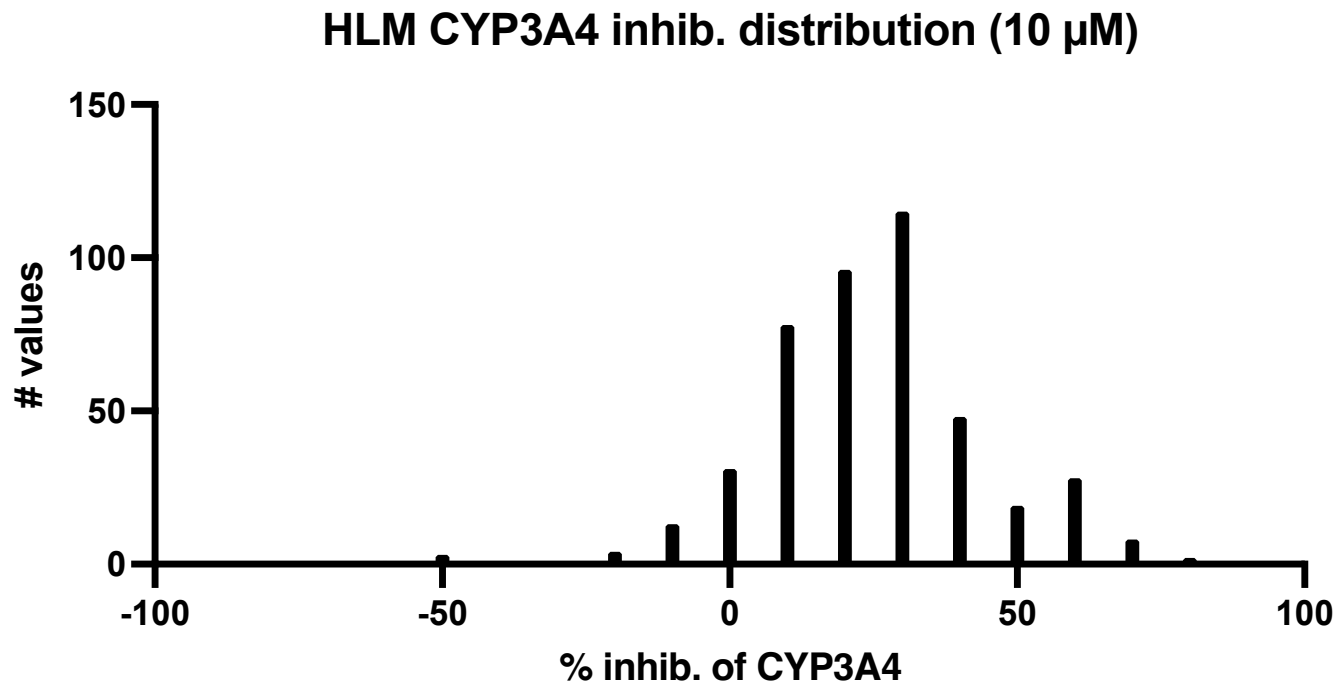

Figure S8: Frequency distribution of the library's % HLM CYP3A4 inhibition.

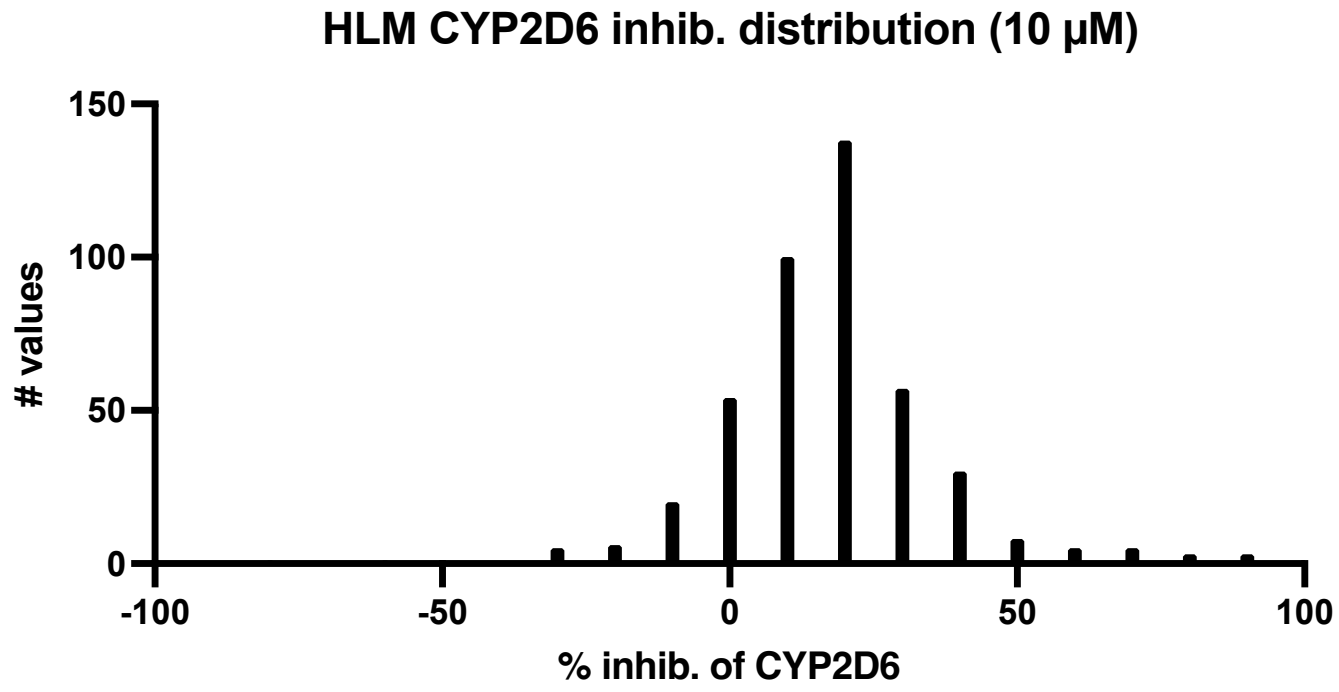

Figure S9: Frequency distribution of the library's % HLM CYP2D6 inhibition.

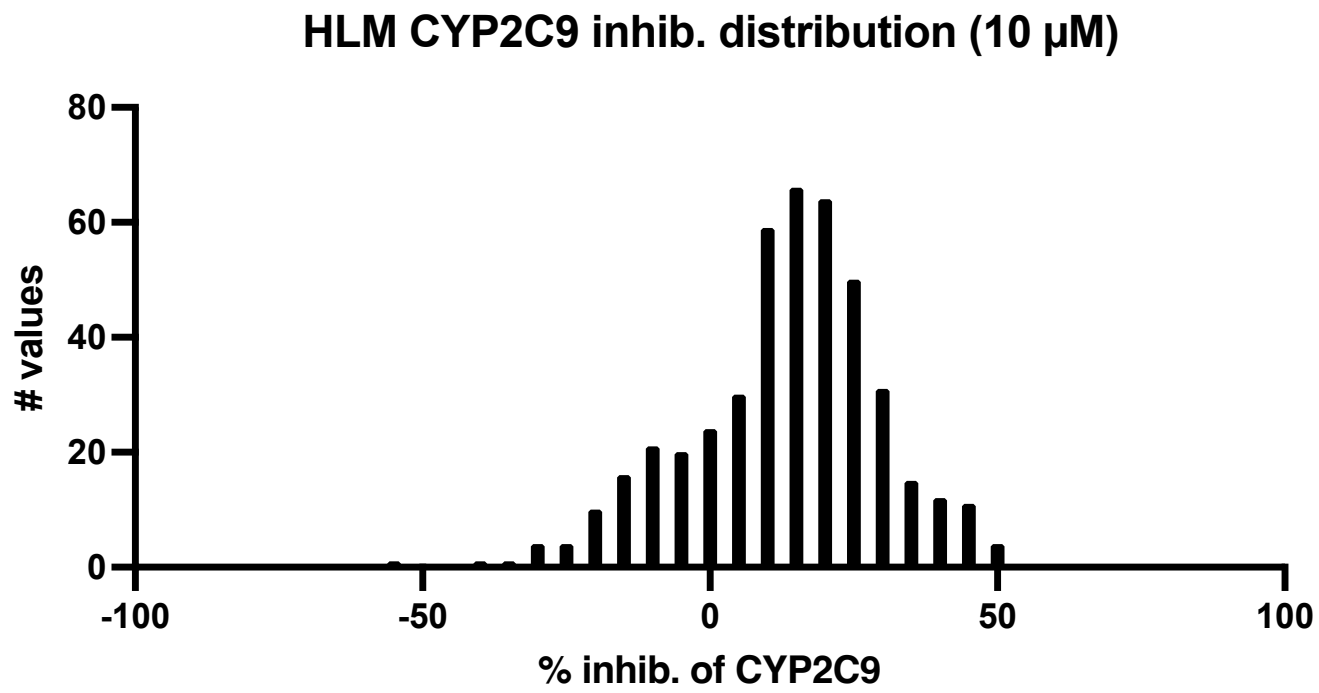

Figure S10: Frequency distribution of the library's % HLM CYP2C9 inhibition.

2.6 Linear model for permeability dependence on building blocks

a)

Prediction Expression

```
0.5088390866

+ Match( Morpholine 1 ) (
    0    ⇒ -0.416648119
    1    ⇒ 0.3639194846
    2    ⇒ -0.095968648
    3    ⇒ 0
    else ⇒ .
)

+ Match( Morpholine 2 ) (
    0    ⇒ -0.359792661
    1    ⇒ -0.054503829
    2    ⇒ 0.0223543788
    3    ⇒ -0.11490471
    4    ⇒ 0
    else ⇒ .
)

+ Match( Amine ) (
    0    ⇒ -0.618566332
    1    ⇒ -0.970071939
    2    ⇒ -1.943444156
    3    ⇒ -1.453634072
    4    ⇒ -0.648981966
    5    ⇒ -1.410850161
    6    ⇒ -1.623563413
    7    ⇒ -1.789200319
    8    ⇒ 0.1158226054
    9    ⇒ -1.470122579
    10   ⇒ -0.291208029
    11   ⇒ -1.848034923
    12   ⇒ -1.390383539
    13   ⇒ -1.524093823
    14   ⇒ -1.711766441
    15   ⇒ -0.091752316
    16   ⇒ -1.21035264
    17   ⇒ -0.198933284
    18   ⇒ -1.767960556
    19   ⇒ -0.534482379
    20   ⇒ -0.899714672
    21   ⇒ -1.767719568
    22   ⇒ -1.861307827
    23   ⇒ 0
    else ⇒ .
)

+ Match( Morpholine 2 ) (
    0    ⇒ Match( Morpholine 1 ) (
             0    ⇒ 0.3319891609
             1    ⇒ -0.144140068
             2    ⇒ 0.4538346042
             3    ⇒ 0
             else ⇒ .
         )
    1    ⇒ Match( Morpholine 1 ) (
             0    ⇒ 0.1909489639
             1    ⇒ -0.204122522
             2    ⇒ -0.023688516
             3    ⇒ 0
             else ⇒ .
         )
    2    ⇒ Match( Morpholine 1 ) (
             0    ⇒ 0
             1    ⇒ 0.0965311234
             2    ⇒ 0.2064905887
             3    ⇒ 0
             else ⇒ .
         )
    3    ⇒ Match( Morpholine 1 ) (
             0    ⇒ 0.8902874133
             1    ⇒ 0.1196249257
             2    ⇒ 0.2956738752
             3    ⇒ 0
             else ⇒ .
         )
    4    ⇒ Match( Morpholine 1 ) (
             0    ⇒ 0
             1    ⇒ 0
             2    ⇒ 0
             3    ⇒ 0
             else ⇒ .
         )
    else ⇒ .
)
```

b)

Effect Tests

Source	Nparm	DF	Wald ChiSquare	Prob > ChiSquare	
Amine	23	23	1646.3987	<.0001*	
Morpholine 1	3	3	40.55884	<.0001*	
Morpholine 2*Morpholine 1	12	11	30.389094	0.0014*	Levels removed: 1
Morpholine 2	4	4	17.618132	0.0015*	

c)

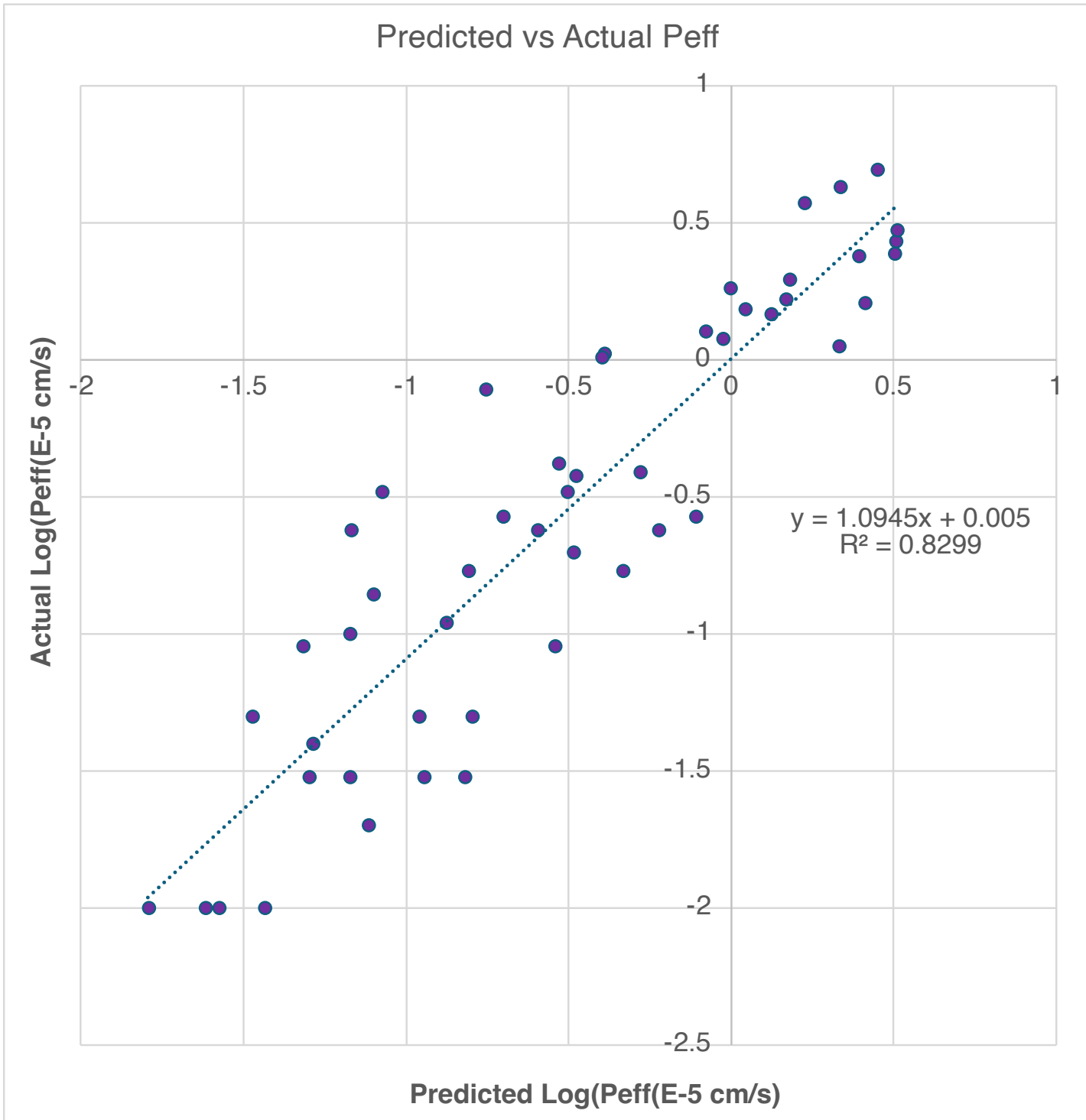

**R^2**	0.830
**RMSE**	0.332
**Spearman Rho**	0.917
**f-statistic**	6.12
**t-statistic**	2.47
**two-tailed p-value**	0.017
**n**	50

Figure S1: Generalized Regression Ridge model for PAMPA P_{eff} dependence on building blocks. **a)** *Prediction formula,* **b)** *Effect tests (no data for* ***mA****(0)–****mD****(2), therefore this level was removed),* **c)** *predicted vs actual PAMPA P_{eff} and linear fit on the test set and descriptors for the predicted vs actual corellation on the test set.*

2.7 ADME assay descriptions

The membrane permeability (PAMPA),[1,2] apical mouse P-gp efflux ratio,[3] lipophilicity (LogD, CAMDIS, EP2005102211A),[4] kinetic solubility (LYSA),[1] and microsomal stability (LM),[1] were measured according to reported procedures.

Cytochrome P450 inhibition assays at 10 µM compound concentration: The assay measures the formation of selective metabolites using LC-MS/MS analysis to determine values and percent inhibition. These methodologies assess both reversible and time-dependent inhibition (TDI) to characterize potential clinical drug-drug interaction (DDI) risks. HLM CYP3A4, CYP2D6, or CYP2C9 are incubated with NADPH and specific probe substrates: diclofenac, dextromethorphan, and midazolam respectively.

3 Cheminformatic workflows

3.1 VL enumeration and analysis

The library is enumerated from the 1 ketone, 5 iSnAP reagents and 24 amines which are imported in the workflow from SD-files. The assembly of building blocks is computed followed by the enumeration of all possible stereoisomers from undefined stereogenic elements. The VL was enumerated with the KNIME analytics platform using RDKit, Schrodinger and Marvin implementations.[5]

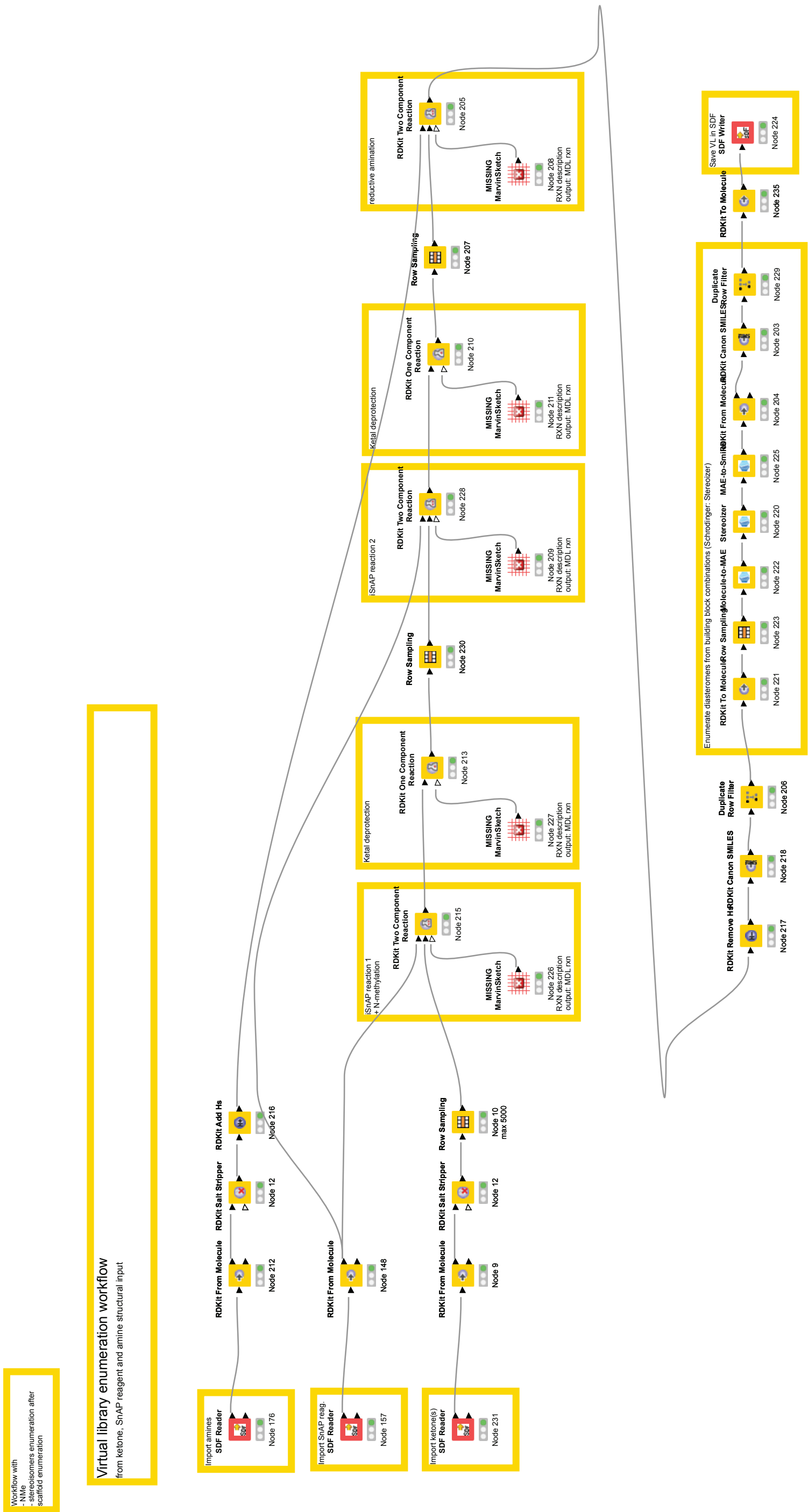

Scheme 3.1.1: Full VL enumeration KNIME workflow run on KNIME 4.7.1. RDKit, Schrodinger and Marvin nodes were used.

3.1.1 VL analysis

The Tanimoto similarities, SlogP, TPSA, HBD, HBA and number of rotatable bonds were calculated using the RDkit library in python or KNIME.[5]

3.1.2 3D shape analysis of pentacycle cores

Conformers were sampled using CREST-gfn2-hexane and subsequently filtered with CENSO using a scalable, computationally efficient DFT optimization (pbe-d4/def2-svp/cpcm cyclohexane), followed by single-point energy calculations (ωb97x-d4/def2-tzvpp/cpcm cyclohexane).[6,7] Computations were parallelized on the Euler cluster. The generated conformer ensembles were analyzed in the Jupyter notebook DataS6_RMSD_Rg_length.ipynb.

3.2 ¹³C NMR predictions for diastereomer assignment

3.2.1 Conformer sampling

A conformer ensemble was sampled with the Conformer-Rotamer Ensemble Sampling Tool (CREST, v2.12),[6] starting from each molecule with xyz coordinates (.xyz) and using the following command:

```
sbatch -n 128 --time=12:00:00 --wrap="crest structure.xyz --gbsa --
gfn2 -T 128 -nmr"
```

3.2.2 Conformer sorting, and ¹³C NMR calculations

The conformer ensemble was sorted by its energies with the Commandline Energetic SOrting (CENSO, v 1.2.0) tool.[7] The following CENSO configuration file ".censorc" was used together with the command:

```
sbatch -n 128 --time=24:00:00 --wrap="censo -inp crest_conformers.xyz
--maxthreads 128 --omp 1 >censo.out"
```

CENSO configuration file .censorc

```
$CENSO global configuration file: .censorc
$VERSION:1.2.0

ORCA: /cluster/software/commercial/orca/5.0.4/x86_64/bin/
ORCA version: 5.0.4
GFN-xTB: /cluster/home/elboschi/bin/xtb
CREST: /cluster/home/elboschi/bin/crest
mpshift: /path/including/binary/mpshift-binary
escf: /path/including/binary/escf-binary

#COSMO-RS
ctd          =          BP_TZVP_C30_1601.ctd          cdir          =
"/software/cluster/COSMOthermX16/COSMOtherm/CTDATA-FILES"   ldir   =
"/software/cluster/COSMOthermX16/COSMOtherm/CTDATA-FILES"
$ENDPROGRAMS

$CRE SORTING SETTINGS:
$GENERAL SETTINGS:
nconf: 200                      # ['all', 'number e.g. 10 up to all
conformers']
charge: 0                        # ['number e.g. 0']
unpaired: 0                      # ['number e.g. 0']
solvent: chcl3                 # ['gas', 'acetone', 'acetonitrile',
'aniline', 'benzaldehyde', 'benzene', 'ccl4', '...']
prog_rrho: xtb                   # ['xtb']
temperature: 298.15              # ['temperature in K e.g. 298.15']
```

```
trange: [273.15, 378.15, 5]       # ['temperature range [start, end,
step]']
multitemp: off                    # ['on', 'off']
evaluate_rrho: off                # ['on', 'off']
consider_sym: on                  # ['on', 'off']
bhess: off                        # ['on', 'off']
imagthr: automatic                 # ['automatic or e.g., -100    #
in cm-1']
sthr: automatic                   # ['automatic or e.g., 50     # in
cm-1']
scale: automatic                  # ['automatic or e.g., 1.0 ']
rmsdbias: off                     # ['on', 'off']
sm_rrho: alpb                     # ['alpb', 'gbsa']
progress: off                     # ['on', 'off']
check: on                         # ['on', 'off']
prog: orca                        # ['tm', 'orca']
func: b97-d3                       # ['b3-lyp', 'b3lyp', 'b3lyp-3c',
'b3lyp-d3', 'b3lyp-d3(0)', 'b3lyp-d4', 'b3lyp-nl', '...']
basis: def2-TZVP                 # ['automatic', 'def2-TZVP', 'def2-
mSVP', 'def2-mSVP', 'def2-mSVP', 'def2-mSVP', '...']
maxthreads: 128                    # ['number of threads e.g. 2']
omp: 1                              # ['number cores per thread e.g.
4']
balance: on                       # ['on', 'off']
cosmorsparam: automatic             # ['automatic', '12-fine', '12-
normal', '13-fine', '13-normal', '14-fine', '...']

$PART0 - CHEAP-PRESCREENING - SETTINGS:
part0: on                         # ['on', 'off']
func0: b97-d3                      # ['b3-lyp', 'b3lyp', 'b3lyp-3c',
'b3lyp-d3', 'b3lyp-d3(0)', 'b3lyp-d4', '...']
basis0: def2-SVP                # ['automatic', 'def2-SV(P)', 'def2-
TZVP', 'def2-mSVP', 'def2-mSVP', 'def2-mSVP', '...']
part0_gfnv: gfn2                  # ['gfn1', 'gfn2', 'gfnff']
part0_threshold: 4.0              # ['number e.g. 4.0']

$PART1 - PRESCREENING - SETTINGS:
# func and basis is set under GENERAL SETTINGS
part1: on                         # ['on', 'off']
smgsolv1: cpcm                  # ['alpb_gsolv', 'cosmo', 'cosmors',
'cosmors-fine', 'cpcm', 'dcosmors', '...']
part1_gfnv: gfn2                  # ['gfn1', 'gfn2', 'gfnff']
part1_threshold: 3.5              # ['number e.g. 5.0']

$PART2 - OPTIMIZATION - SETTINGS:
# func and basis is set under GENERAL SETTINGS
part2: on                         # ['on', 'off']
prog2opt: prog                 # ['tm', 'orca', 'prog', 'automatic']
```

```
part2_threshold: 2.5                # ['number e.g. 4.0']
sm2: cpcm                              # ['cosmo', 'cpcm', 'dcosmors',
'default', 'smd']
smgsolv2: cpcm                    # ['alpb_gsolv', 'cosmo', 'cosmors',
'cosmors-fine', 'cpcm', 'dcosmors', '...']
part2_gfnv: gfn2                    # ['gfn1', 'gfn2', 'gfnff']
ancopt: on                          # ['on']
hlow: 0.01                            # ['lowest force constant in ANC
generation, e.g. 0.01']
opt_spearman: on                    # ['on', 'off']
part2_P_threshold: 99                # ['Boltzmann sum threshold in %.
e.g. 95 (between 1 and 100)']
optlevel2: automatic                   # ['crude', 'sloppy', 'loose',
'lax', 'normal', 'tight', 'vtight', 'extreme', '...']
optcycles: 8                        # ['number e.g. 5 or 10']
spearmanthr: -4.0                      # ['value between -1 and 1, if
outside set automatically']
radsize: 10                         # ['number e.g. 8 or 10']
crestcheck: on                      # ['on', 'off']

$PART3 - REFINEMENT - SETTINGS:
part3: on                           # ['on', 'off']
prog3: prog                         # ['tm', 'orca', 'prog']
func3: b3lyp-d4                      # ['b3-lyp', 'b3lyp', 'b3lyp-3c',
'b3lyp-d3', 'b3lyp-d3(0)', 'b3lyp-d4', 'b3lyp-nl', '...']
basis3: def2-QZVPP                    # ['DZ', 'QZV', 'QZVP', 'QZVPP',
'SV(P)', 'SVP', 'TZVP', 'TZVPP', 'aug-cc-pV5Z', '...']
smgsolv3: cpcm                    # ['alpb_gsolv', 'cosmo', 'cosmors',
'cosmors-fine', 'cpcm', 'dcosmors', '...']
part3_gfnv: gfn2                    # ['gfn1', 'gfn2', 'gfnff']
part3_threshold: 99                  # ['Boltzmann sum threshold in %.
e.g. 95 (between 1 and 100)']

$NMR PROPERTY SETTINGS:
$PART4 SETTINGS:
part4: on                           # ['on', 'off']
couplings: off                      # ['on', 'off']
progJ: prog                         # ['tm', 'orca', 'prog']
funcJ: pbe0-d4                       # ['b3-lyp', 'b3lyp', 'b3lyp-3c',
'b3lyp-d3', 'b3lyp-d3(0)', 'b3lyp-d4', 'b3lyp-nl', '...']
basisJ: cc-pVTZ                       # ['DZ', 'QZV', 'QZVP', 'QZVPP',
'SV(P)', 'SVP', 'TZVP', 'TZVPP', 'aug-cc-pV5Z', '...']
sm4J: cpcm                             # ['cosmo', 'cpcm', 'dcosmors',
'smd']
shieldings: on                      # ['on', 'off']
progS: prog                         # ['tm', 'orca', 'prog']
funcS: pbe0-d4                       # ['b3-lyp', 'b3lyp', 'b3lyp-3c',
'b3lyp-d3', 'b3lyp-d3(0)', 'b3lyp-d4', 'b3lyp-nl', '...']
```

```
basisS: cc-pVTZ                    # ['DZ', 'QZV', 'QZVP', 'QZVPP',
'SV(P)', 'SVP', 'TZVP', 'TZVPP', 'aug-cc-pV5Z', '...']
sm4S: cpcm                          # ['cosmo', 'cpcm', 'dcosmors',
'smd']
reference_1H: TMS                 # ['TMS']
reference_13C: TMS                # ['TMS']
reference_19F: CFCl3              # ['CFCl3']
reference_29Si: TMS               # ['TMS']
reference_31P: TMP                # ['TMP', 'PH3']
1H_active: on                     # ['on', 'off']
13C_active: on                    # ['on', 'off']
19F_active: off                   # ['on', 'off']
29Si_active: off                  # ['on', 'off']
31P_active: off                   # ['on', 'off']
resonance_frequency: 600.0       # ['MHz number of your experimental
spectrometer setup']

$OPTICAL ROTATION PROPERTY SETTINGS:
$PART5 SETTINGS:
optical_rotation: off             # ['on', 'off']
funcOR: pbe                         # ['functional for opt_rot e.g.
pbe']
funcOR_SCF: r2scan-3c              # ['functional for SCF in opt_rot
e.g. r2scan-3c']
basisOR: def2-SVPD                   # ['basis set for opt_rot e.g.
def2-SVPD']
frequency_optical_rot: [589.0]    # ['list of freq in nm to evaluate
opt rot at e.g. [589, 700]']
$END CENSORC
```

Chemical shift calculation

Chemical shifts (δ) were calcualted from the computed isotropic shielding constants (σ) with the following formula:

$$\delta_{predicted} = \sigma_{ref} - \sigma_{predicted}$$

$$\delta_{predicted} = \sigma_{TMS} - \sigma_{predicted}, \sigma_{TMS} = 185 \text{ ppm}$$

4 LIBRARY SYNTHESIS

4.1 General remarks

Materials: All chemicals were commercially available, purchased from ABCR, Acros, Alfa Aesar, Apollo Scientific, Fluorochem, Merck, Sigma-Aldrich or TCI, and used as received. Solvents were used as received in ACS or HPLC grade.

Reaction monitoring by LC-MS

Liquid chromatography-mass spectrometry (LC-MS) measurements of small molecules were performed on the following instruments:

Bruker amaZon SL*:* LC: Agilent 1290 Infinity II system (Agilent Ltd., Germany); chromatography on Waters Acquity BEH C18 RP column (1.7 *μ*m particle size, 2.1 x 50 mm column dimension) at 25 °C, H_2O/CH_3CN + 0.1 v% formic acid with a gradient from 98/2 to 2/98 (v/v) over 7 min at 0.6 mL/min flow rate; UV/VIS chromatogram acquired on a diode array (DAD) LC-detector (190-940 nm, 2.0 nm resolution). MS: Bruker ESI-Qq-TOF system (compact, Bruker Daltonics, Germany); ion-trap mass analyzer, data collected between m/z = 50-1100, alternating between positive and negative mode. CsI solution injected after each run for mass shift calibration (positive and negative mode; quadratic). Trap calibrated regularly with Agilent Tunemix (PN G1969-85000). The data processing was done using the Compass DataAnalysis software (Bruker Daltonics, Germany). Agilent G6135C*:* LC: Agilent 1290 Infinity II system (Agilent Ltd., Germany); chromatography on Agilent C3 RP column (1.8 *μ*m particle size, 2.1 x 50 mm column dimension) at 25 °C, H_2O/CH_3CN + 0.1 v% formic acid with a gradient from 98/2 to 2/98 (v/v) over 7 min at 0.6 mL/min flow rate; UV/VIS chromatogram acquired on a diode array (DAD) LC-detector (190-450 nm, 2.0 nm resolution). MS: Agilent XT ESI-Q-system (Agilent, Germany); data collected between m/z = 100-1000 in positive and negative mode. The quadrupole is calibrated with Agilent Tunemix (PN G1969-85000). Agilent G6120C*:* LC: Agilent 1260 Infinity system (Agilent Ltd., Germany); chromatography on Waters BEH C18 RP column (1.8 *μ*m particle size, 2.1 x 50 mm column dimension) at 25 °C, H_2O/CH_3CN + 0.1 v% formic acid with a gradient from 98/2 to 2/98 (v/v) over 7 min at 0.6 mL/min flow rate; UV/VIS chromatogram acquired on a multiwavelength (MWD) detector (Standard wavelengths: 254 and 220 nm, 4.0 nm resolution). MS: Agilent 6120 ESI-Q-system (Agilent, Germany); data collected between m/z = 100-1000 in positive and negative mode. The quadrupol is calibrated with Agilent Tunemix (PN G1969-85000).

Purification

Extraction and washing steps were performed with technical grade solvents.

Flash column chromatography on silica was either performed manually on Sigma Aldrich SiO_2 Type F60 (high-purity grade, 60 Å pore size, 230-400 mesh particle size) using compressed air pressure or on Biotage Isolera™ using Büchi EcoFlex NP silica columns. The crude products were either loaded as solution in suitable solvents or by dry-loading onto silica and Celite.

HT reverse phase HPLC purification was performed on an Agilent 1290 Infinity II system with a Phenomenex Gemini C18 (100x30 mm) column and a 5–50 MeCN/H_2O(0.1% Et_3N) gradient over 11 min.

Characterization

NMR spectra were recorded on Bruker Avance 600 MHz, Bruker Avance 500 MHz, and Bruker Avance 400 MHz spectrometers. Chemical shifts are reported in parts per million (ppm) and peaks were referenced to residual protonated solvents. ^{19}F-NMR and ^{11}B-NMR spectra were referenced to an external standard of CF_3COOH and $BF_3 \cdot OEt_2$, respectively. NMR coupling constants (*J*) are reported in Hertz (Hz). All ^{13}C and ^{19}F spectra were measured with proton decoupling, unless otherwise specified. High-resolution mass spectra measurements were performed by the Molecular and Biomolecular Analysis Service (MoBiAS, ETH Zurich) on either a Bruker Daltonics maXis ESI-QTOF or on a Bruker solariX ESI/MALDI-FTICR mass spectrometer. LC-MS high resolution spectra were recorded with an Agilent LC-system consisting of Agilent 1290 high pressure gradient system, an Agilent 1290 Multisampler and an Agilent 6545 QTOF. The separation was achieved on a Zorbax Eclipse Plus C18 1,7 μm 2.1*50mm column at 55°C; A=0.02% formic acid in Water; B= acetonitrile with 0.01% formic acid at flow 0.8 mL/min. The gradient covered the following program: 0 min 5%B, 0.3 min 5%B, 4.5 min 99 %B 5 min 99%B. Ionization was performed in Agilents Multimode Source. The mass spectrometer was run in “2 GHz extended dynamic range” mode, resulting in a resolution of about 26 000 at m/z=922. Mass accuracy was ensured by internal drift correction.IR spectra were recorded on a JASCO FT-IR-4100 spectrometer and reported as wavenumbers in cm^{-1}. Only major peaks > 500 v/cm^{-1} are reported. Background measurements were performed prior to sample measurements. Optical rotations were measured on a Jasco P-2000, operating at the sodium D line with a 100 mm path length cell. Background measurements were performed prior to blank (used solvent) and sample measurements. Obtained values were averaged over 5 measurements.

4.2 Preparation of the copper catalyst $Cu(DMP)_2(OTf)_2$

1. Copper(II)-(2,6-dimethoxypyridine)$_2$-(trifluoromethanesulfonate)$_2$

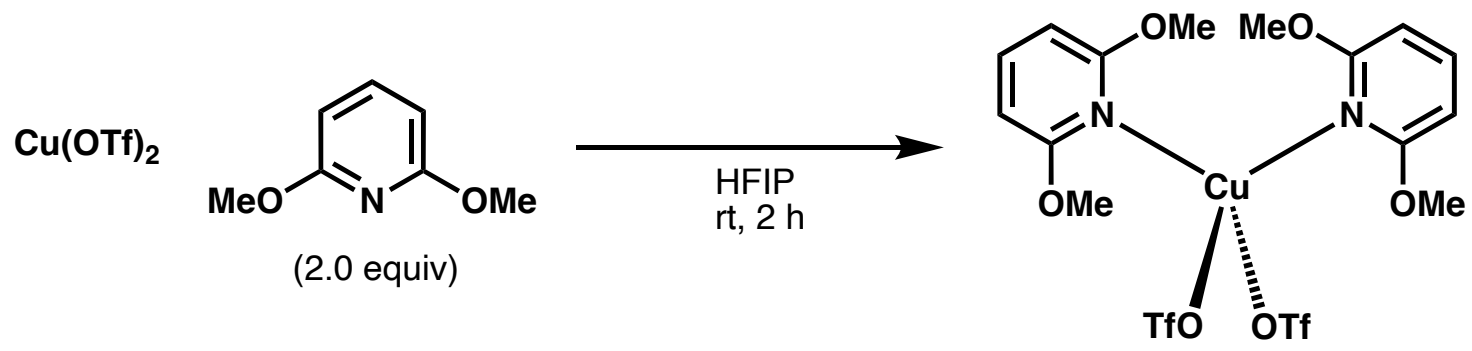

$Cu(OTf)_2$ (60 g, 0.17 mol, 1.0 equiv) was suspended in HFIP (1.0 L) and 2,6-dimethoxypyridine (44 mL, 0.33 mol, 2.0 equiv) was added. While the suspension was stirred at rt, the color changed from colorless, over light blue to green. The mixture was stirred at room temperature for 2 h, during which the color evolved from colorless to light blue and finally to green. The suspension was filtered to remove uncomplexed $Cu(OTf)_2$, and the filtrate was concentrated under reduced pressure. The residue was dried under high vacuum to remove residual unbound ligand, affording complex **1** as a highly hygroscopic turquoise solid (73 g, 0.11 mol, 69% yield) which was stored in a desiccator under N_2 atmosphere and used without further purification in subsequent steps.

4.3 SnAP reagents

The SnAP reagents used for the library synthesis illustrated below were synthesized according to literature procedures.[8]

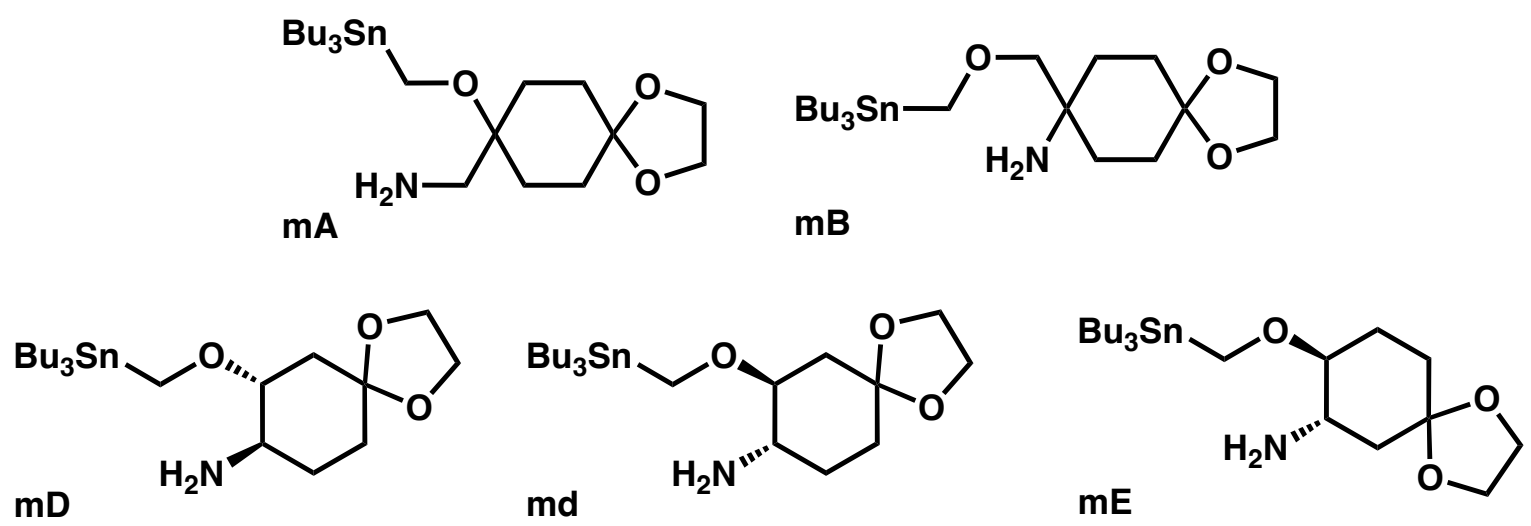

Figure 4.3.1: iSnAP reagents used in the library synthesis.

4.4 General procedures

GP1.1 SnAP protocol for mA, mD, md, mE

In an oven-dried round bottom flask under nitrogen atmosphere, the ketone (1.0 equiv), SnAP reagent (1.0 equiv), CH_2Cl_2 (0.5 M) and Ti(O*i*Pr)$_4$ (1.5 equiv) were added. After stirring the reaction overnight, $Cu(DMP)_2(OTf)_2$ (2.0 equiv) was added together with hexafluoroisopropanol (HFIP, freshly distilled over 4 Å molecular sieves, 0.05 M). After stirring the green solution at rt overnight, it was concentrated under reduced pressure and a minimal amount of CH_2Cl_2 and NH_3 (12% aq) were added while stirring until the color had fully changed from green to blue. The biphasic blue suspension was filtered through a celite plug, the phases of the filtrate were separated, and the aqueous phase was extracted 2 x with CH_2Cl_2, dried with Na_2SO_4 and concentrated *under reduced pressure*.

Purification by flash column chromatography, 0 – 5% MeOH/CH_2Cl_2 (tricycle) or 5 – 25% MeOH/CH_2Cl_2 (pentacycle) afforded the corresponding morpholines.

GP1.2 SnAP protocol for mB starting from a tricyclic ketone

In an oven-dried round bottom flask under nitrogen atmosphere, the ketone (1.0 equiv), SnAP reagent (1.0 equiv), and $Ti(O\textit{i}Pr)_4$ (3.0 equiv) were added. To solubilize the ketone, a minimal amount of CH_2Cl_2 was added and carefully removed under high vacuum. The yellow solution was stirred overnight at rt under high vacuum. The next morning, $Cu(DMP)_2(OTf)_2$ (2.0 equiv) in HFIP (freshly distilled over 4 Å molecular sieves, 0.05 M) were added. The green mixture was stirred at rt overnight. Work-up and purification were performed according to **GP1.1**.

GP2 N-methylation

To a solution of the morpholine derivative (1.0 equiv) in CH_2Cl_2 (0.1 M), formalin (37% aq, 1.2 equiv) and $NaBH(OAc)_3$ (1.5 equiv). The suspension was stirred for 1.5 h at rt, quenched with saturated aqueous $NaHCO_3$, and extracted with CH_2Cl_2. The crude product was used in the next step without further purification.

GP3 ketal deprotection

To a solution of N-methyl morpholine ketal (1.0 equiv) in THF (0.23 M) was added HCl (3.0 M aq, 60 equiv). The mixture was stirred at room temperature for 1–2 d. Reaction progress was monitored by LCMS and ^1H NMR spectroscopy. Upon completion, the mixture was basified with ice-cold NaOH (aq) and extracted 2 x with CH_2Cl_2. Purification by flash column chromatography, 0–5% $MeOH/CH_2Cl_2$ (tricycle) or 5 – 25% $MeOH/CH_2Cl_2$ (pentacycle) afforded the corresponding ketones.

4.5 Synthesis of tricycles

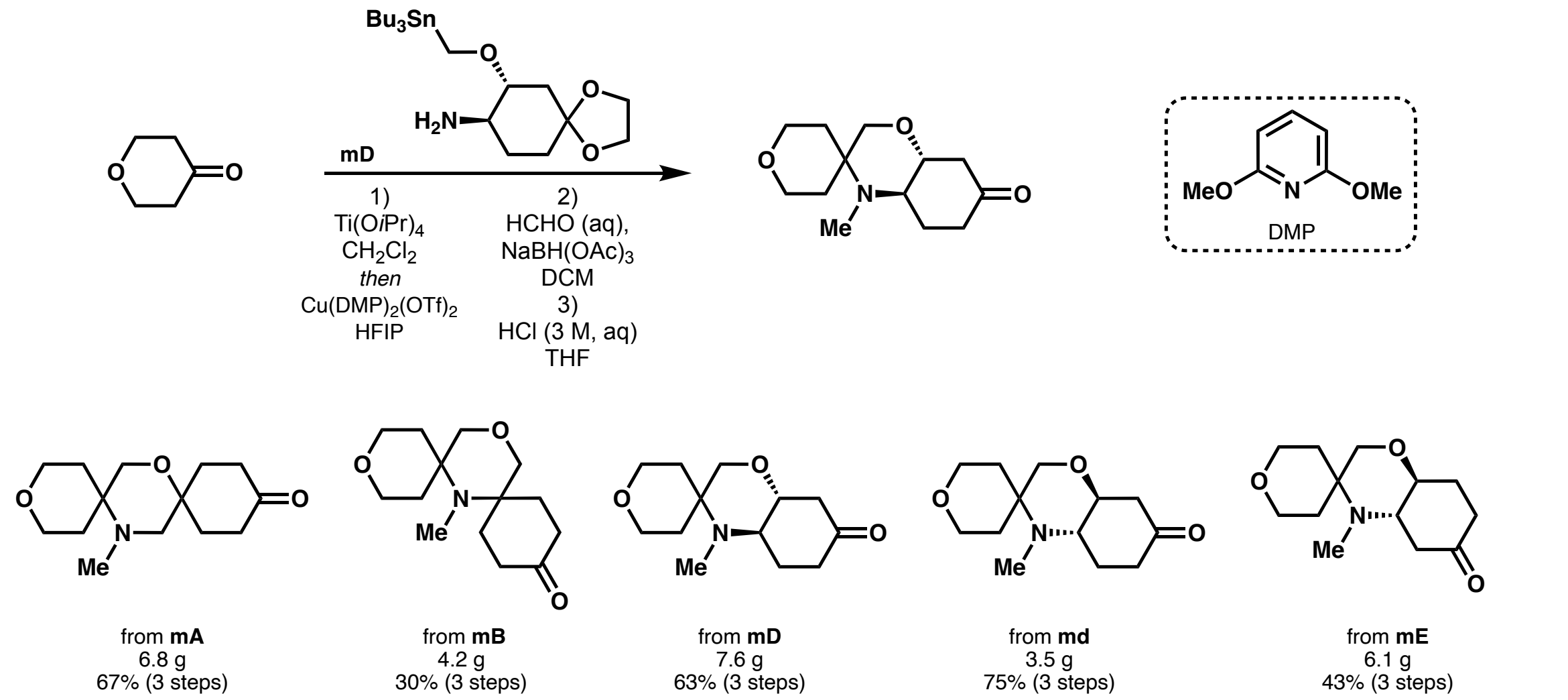

Scheme 4.5.1: Overview on the assembly of the first morpholines from tetrahydropyran-4-one and the SnAP reagents **mA**–**mE** *(Figure 4.3.1).*

2. 1,4,9,14-tetraoxa-17-azatrispiro[4.2.2.5^{11}.2^{8}.2^{5}]icosane

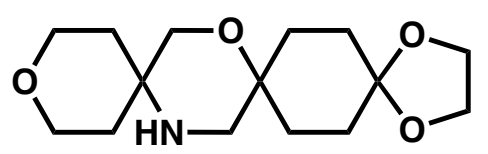

Tetrahydropyran-4-one (4.0 g, 40 mmol, 1.0 equiv) and SnAP reagent **mA** (20 g, 40 mmol, 1.0 equiv) were allowed to react according to **GP1.1** to afford the morpholine ketal **2** (7.7 g, 27 mmol, 68% yield) as a yellow solid.

^{1}H NMR (400 MHz, $CDCl_3$) δ 4.00 – 3.86 (m, 4H), 3.76 (ddd, *J* = 11.6, 7.9, 3.7 Hz, 2H), 3.59 (ddd, *J* = 11.7, 6.3, 4.0 Hz, 2H), 3.44 (s, 2H), 2.68 (d, *J* = 5.2 Hz, 2H), 2.05 – 1.94 (m, 2H), 1.81 (td, *J* = 12.8, 4.2 Hz, 2H), 1.71 – 1.40 (m, 8H), 1.21 – 1.11 (m, 1H).

^{13}C NMR (101 MHz, $CDCl_3$) δ 109.2, 69.7, 68.5, 64.5, 64.3, 63.7, 48.9, 47.7, 33.7, 30.0, 29.7.

HRMS (ESI): m/z, $[M+H]^+$ calc. for $C_{15}H_{26}NO_4$: 284.1856; found: 284.1850.

IR (ν/cm^{-1}, thin film) 2931, 2863, 1441, 1375, 1302, 1240, 1174, 1031, 925, 888, 854, 836, 784, 703, 668, 608, 578.

3. 17-methyl-1,4,9,14-tetraoxa-17-azatrispiro[4.2.2.5¹¹.2⁸.2⁵]icosane

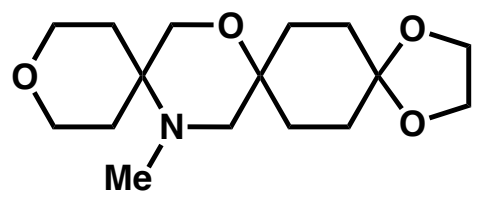

Morpholine ketal **2** (7.7 g, 27 mmol, 1.0 equiv) was allowed to react with formaldehyde (37% aq, 2.5 mL, 33 mmol, 1.2 equiv) and $NaBH(OAc)_3$ (6.8 g, 41 mmol, 1.5 equiv) according to **GP2** to afford the N-methylated morpholine **3** (7.9 g, 27 mmol, 98% yield) as a yellow solid.

^{1}H NMR (400 MHz, $CDCl_3$) δ 3.98 – 3.88 (m, 4H), 3.88 – 3.80 (m, 2H), 3.68 (s, 2H), 3.48 (td, *J* = 12.4, 2.3 Hz, 2H), 2.39 (s, 2H), 2.22 (s, 3H), 2.09 – 1.99 (m, 2H), 1.96 – 1.73 (m, 4H), 1.57 – 1.44 (m, 6H).

^{13}C NMR (101 MHz, $CDCl_3$) δ 109.2, 70.7, 64.8, 64.7, 64.4, 64.3, 58.3, 52.8, 37.0, 30.6, 30.1, 26.3.

HRMS (ESI): m/z, $[M+H]^+$ calc. for $C_{16}H_{28}NO_4$: 298.2013; found: 298.2015.

IR (ν/cm^{-1}, thin film) 2951, 2854, 2800, 1441, 1372, 1352, 1259, 1220, 1169, 1146, 1100, 1078, 1027, 943, 926, 909, 855, 831, 746, 677, 656, 610, 590, 517.

4. 7-methyl-3,15-dioxa-7-azadispiro[5.2.5⁹.2⁶]hexadecan-12-one

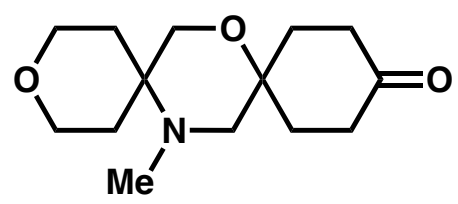

N-methyl morpholine ketal **3** (7.9 g, 27 mmol, 1.0 equiv) was allowed to react with HCl (1 M aq, 130 mL, 130 mmol, 4.8 equiv) according to **GP3** to afford the tricyclic morpholine ketone **4** (6.8 g, 27 mmol, 100% yield) as an off-white solid.

^{1}H NMR (400 MHz, $CDCl_3$) δ 3.94 – 3.86 (m, 2H), 3.76 (s, 2H), 3.51 (td, *J* = 12.3, 2.3 Hz, 2H), 2.64 – 2.52 (m, 2H), 2.46 (s, 2H), 2.43 – 2.35 (m, 2H), 2.26 (s, 3H), 2.25 – 2.22 (m, 1H), 2.22 – 2.18 (m, 1H), 2.00 – 1.89 (m, 2H), 1.62 (td, *J* = 13.6, 4.8 Hz, 2H), 1.56 – 1.50 (m, 2H).

^{13}C NMR (101 MHz, $CDCl_3$) δ 212.1, 70.4, 65.3, 64.7, 57.8, 52.8, 36.9, 36.4, 32.5, 26.3.

HRMS (ESI): m/z, $[M+H]^+$ calc. for $C_{14}H_{24}NO_3$: 254.1751; found: 254.1747.

IR (ν/cm^{-1}, thin film) 2955, 2859, 2802, 1711, 1457, 1411, 1354, 1315, 1222, 1184, 1170, 1142, 1121, 1101, 1076, 1028, 949, 882, 859.

5. 1,4,13,17-tetraoxa-9-azatrispiro[4.2.1.5^10.3^8.2^5]icosane

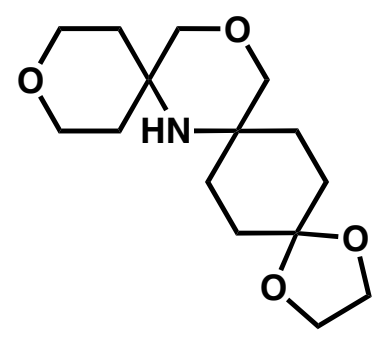

Tetrahydropyran-4-one (8.2 g, 82 mmol, 2.0 equiv) and SnAP reagent **mB** (20 g, 41 mmol, 1.0 equiv) were allowed to react at ambient pressure but otherwise following **GP1.2** to afford the morpholine ketal **5** (3.9 g, 14 mmol, 33% yield) as an off-white solid.

^{1}H NMR (400 MHz, $CDCl_3$) δ 3.98 – 3.88 (m, 4H), 3.82 (ddd, J = 11.2, 7.1, 3.8 Hz, 2H), 3.54 (ddd, J = 11.3, 7.2, 3.7 Hz, 2H), 3.47 (s, 2H), 3.42 (s, 2H), 1.90 – 1.77 (m, 2H), 1.70 – 1.49 (m, 10H), 0.87 (s, 1H).

^{13}C NMR (101 MHz, $CDCl_3$) δ 108.9, 77.1, 76.7, 64.4, 64.3, 64.0, 49.6, 48.0, 37.3, 34.2, 30.6.

HRMS (ESI): m/z, $[M+H]^+$ calc. for $C_{15}H_{26}NO_4$: 284.1856; found: 284.1856.

IR (ν/cm^{-1}, thin film) 2932, 2852, 1455, 1371, 1272, 1239, 1165, 1033, 1014, 946, 931, 916, 875, 845, 733, 661.

6. 9-methyl-1,4,13,17-tetraoxa-9-azatrispiro[4.2.1.5^10.3^8.2^5]icosane

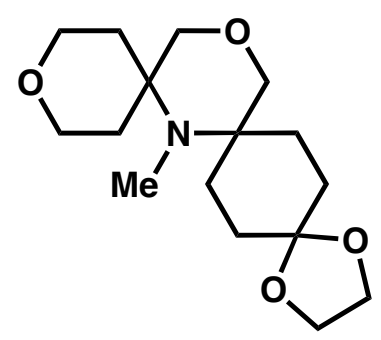

Morpholine ketal **5** (5.2 g, 18 mmol, 1.0 equiv) was allowed to react with formaldehyde (37% aq, 4.1 mL, 55 mmol, 3.0 equiv) and $NaBH(OAc)_3$ (12 g, 55 mmol, 3.0 equiv) according to **GP2**. The procedure was repeated with the crude product 2 x to reach full conversion of the starting material, affording the N-methylated morpholine **6** (5.4 g, 18 mmol, 100% yield) as an off-white solid.

^{1}H NMR (400 MHz, $CDCl_3$) δ 3.95 – 3.86 (m, 4H), 3.81 (ddt, J = 12.1, 5.8, 1.3 Hz, 2H), 3.72 (s, 2H), 3.66 (s, 2H), 3.41 (td, J = 12.4, 2.3 Hz, 2H), 2.31 (s, 3H), 2.14 – 1.96 (m, 4H), 1.66 – 1.52 (m, 4H), 1.52 – 1.44 (m, 2H), 1.38 (d, J = 14.9 Hz, 2H).

^{13}C NMR (101 MHz, $CDCl_3$) δ 108.1, 73.8, 73.6, 64.9, 64.4, 64.3, 54.9, 53.0, 31.6, 31.1, 28.0, 26.8.

HRMS (ESI): m/z, $[M+H]^+$ calc. for $C_{16}H_{28}NO_4$: 298.2013; found: 298.2014.

IR (ν/cm^{-1}, thin film) 2960, 2931, 2883, 2865, 2854, 1469, 1450, 1387, 1368, 1355, 1350, 1337, 1272, 1267, 1233, 1223, 1193, 1171, 1159, 1134, 1116, 1093, 1062, 1029, 1015, 1009, 997, 981, 956, 951, 940, 907, 896, 876, 849, 829, 804, 769, 729, 715, 690, 661, 648, 628, 620, 558, 521, 518.

7. 7-methyl-3,15-dioxa-7-azadispiro[5.1.5⁸.3⁶]hexadecan-11-one

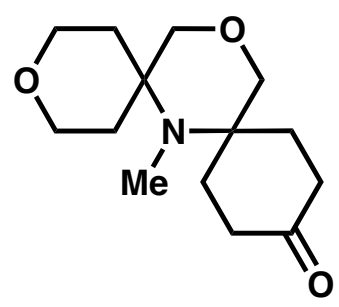

N-methyl morpholine ketal **6** (5.5 g, 18 mmol, 1.0 equiv) was allowed to react with HCl (3.0 M aq, 0.37 L, 1.1 mol, 60 equiv) according to **GP3** to afford the tricyclic morpholine ketone **7** (4.2 g, 17 mmol, 90% yield) as an off-white solid.

^{1}H NMR (400 MHz, $CDCl_3$) δ 3.84 (ddt, *J* = 12.2, 5.8, 1.4 Hz, 2H), 3.78 (s, 2H), 3.61 (s, 2H), 3.44 (td, *J* = 12.5, 2.3 Hz, 2H), 2.44 – 2.34 (m, 2H), 2.33 (s, 3H), 2.33 – 2.23 (m, 2H), 2.14 – 1.97 (m, 4H), 1.76 (dt, *J* = 13.7, 5.8 Hz, 2H), 1.42 – 1.33 (m, 2H).

^{13}C NMR (101 MHz, $CDCl_3$) δ 212.0, 74.3, 73.2, 64.7, 54.6, 52.9, 37.5, 30.8, 27.9, 27.3.

HRMS (ESI): m/z, $[M+H]^+$ calc. for $C_{14}H_{24}NO_3$: 254.1751; found: 254.1747.

IR (ν/cm^{-1}, thin film) 2958, 2851, 1714, 1457, 1238, 1194, 1182, 1165, 1149, 1123, 1103, 1055, 1012, 986, 967, 939, 855.

8. (4a'R,8a'R)-decahydro-2'H-dispiro[pyran-4,3'-benzo[b][1,4]oxazine-7',2''-[1,3]dioxolane]

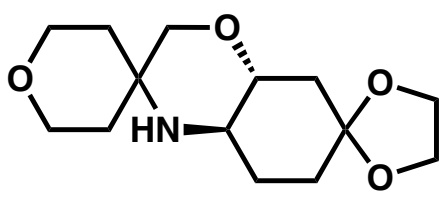

Tetrahydropyran-4-one (1.1 g, 11 mmol, 1.0 equiv) and SnAP reagent **mD** (5.3 g, 11 mmol, 1.0 equiv) were allowed to react according to **GP1.1** to afford the morpholine ketal **8** (2.2 g, 8.2 mmol, 72% yield) as a white solid.

^{1}H NMR (400 MHz, $CDCl_3$) δ 3.97 – 3.86 (m, 4H), 3.82 (d, *J* = 11.2 Hz, 1H), 3.73 (dtd, *J* = 11.7, 7.9, 3.7 Hz, 2H), 3.54 (dddd, *J* = 26.5, 11.4, 7.0, 3.8 Hz, 2H), 3.18 (d, *J* = 11.3 Hz, 1H), 3.05 (ddd, *J* = 12.1, 9.2, 4.3 Hz, 1H), 2.59 (ddd, *J* = 11.8, 9.2, 3.6 Hz, 1H), 1.97 – 1.83 (m, 2H), 1.78 (ddd, *J* = 13.7, 7.0, 3.7 Hz, 1H), 1.74 – 1.65 (m, 1H), 1.63 – 1.52 (m, 3H), 1.43 (dddd, *J* = 13.7, 7.6, 3.9, 1.0 Hz, 1H), 1.38 – 1.16 (m, 3H).

^{13}C NMR (101 MHz, $CDCl_3$) δ 109.1, 79.5, 75.3, 64.5, 64.4, 64.2, 63.2, 53.1, 49.2, 39.3, 36.4, 33.8, 32.2, 27.5.

HRMS (ESI): m/z, $[M+H]^+$ calc. for $C_{14}H_{24}NO_4$: 270.1700; found: 270.1702.

IR (ν/cm^{-1}, thin film) 2945, 2862, 1446, 1361, 1305, 1237, 1208, 1174, 1145, 1093, 1067, 1057, 1025, 947, 934, 922, 844, 806, 735, 686, 653, 613.

$[\alpha]_D^{23}$ –14.27 ° (c 0.09, CH_2Cl_2).

9. (4a'R,8a'R)-4'-methyldecahydro-2'H-dispiro[pyran-4,3'-benzo[b][1,4]oxazine-7',2''-[1,3]dioxolane]

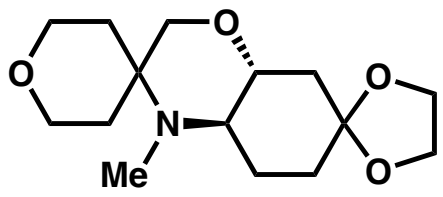

Morpholine ketal **8** (10 g, 37 mmol, 1.0 equiv) was allowed to react with formaldehyde (37% aq, 3.4 mL, 45 mmol, 1.2 equiv) and $NaBH(OAc)_3$ (12 g, 56 mmol, 1.5 equiv) according to **GP2** to afford the N-methylated morpholine **9** (10 g, 37 mmol, 99% yield) as an off-white solid.

^{1}H NMR (400 MHz, $CDCl_3$) δ 4.29 (d, *J* = 11.6 Hz, 1H), 4.00 – 3.86 (m, 5H), 3.81 (ddt, *J* = 11.8, 5.4, 1.6 Hz, 1H), 3.55 (td, *J* = 12.0, 2.7 Hz, 1H), 3.48 – 3.34 (m, 2H), 3.31 (dd, *J* = 11.6, 2.0 Hz, 1H), 2.34 (ddd, *J* = 11.4, 9.4, 3.7 Hz, 1H), 2.27 (s, 3H), 2.15 (ddd, *J* = 13.8, 12.4, 5.5 Hz, 1H), 2.02 – 1.88 (m, 3H), 1.83 – 1.70 (m, 2H), 1.64 (t, *J* = 12.2 Hz, 1H), 1.54 (td, *J* = 13.6, 4.3 Hz, 1H), 1.33 (dddd, *J* = 13.8, 12.9, 11.5, 3.8 Hz, 1H), 1.11 (dq, *J* = 13.9, 2.2 Hz, 1H).

^{13}C NMR (101 MHz, $CDCl_3$) δ 108.6, 77.9, 71.3, 64.9, 64.6, 64.5, 64.5, 60.6, 53.8, 39.9, 34.0, 32.5, 31.9, 25.5, 22.9.

HRMS (ESI): m/z, $[M+H]^+$ calc. for $C_{15}H_{26}NO_4$: 284.1856; found: 284.1851.

IR (ν/cm^{-1}, thin film) 2954, 2856, 2793, 1476, 1446, 1391, 1373, 1356, 1313, 1281, 1239, 1207, 1191, 1173, 1150, 1093, 1074, 1045, 1028, 1013, 1001, 979, 947, 931, 922, 853, 833, 806, 726, 687, 661, 621.

$[\alpha]_D^{24}$ –30.79 ° (c 0.1, CH_2Cl_2).

10. (4aR,8aR)-4-methyldecahydro-2H,7H-spiro[benzo[b][1,4]oxazine-3,4'-pyran]-7-one

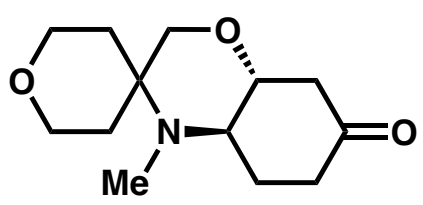

N-methyl morpholine ketal **9** (10 g, 36 mmol, 1.0 equiv) was allowed to react with HCl (3.0 M aq, 0.73 L, 2.2 mmol, 60 equiv) according to **GP3** to afford the tricyclic morpholine ketone **10** (7.6 g, 32 mmol, 88% yield) as an off-white solid.

^{1}H NMR (400 MHz, $CDCl_3$) δ 4.33 (d, *J* = 11.7 Hz, 1H), 3.94 (ddt, *J* = 12.0, 5.5, 1.6 Hz, 1H), 3.83 (ddt, *J* = 11.9, 5.5, 1.6 Hz, 1H), 3.55 (td, *J* = 12.1, 2.7 Hz, 1H), 3.48 – 3.35 (m, 2H), 3.31 (dd, *J* = 11.7, 2.1 Hz, 1H), 2.75 – 2.60 (m, 2H), 2.51 – 2.36 (m, 2H), 2.36 – 2.12 (m, 6H), 1.96 (dq, *J* = 13.3, 2.3 Hz, 1H), 1.81 (dddd, *J* = 13.2, 12.2, 5.5, 2.1 Hz, 1H), 1.37 (tdd, *J* = 13.4, 11.3, 4.6 Hz, 1H), 1.11 (dq, *J* = 13.8, 2.2 Hz, 1H).

^{13}C NMR (101 MHz, $CDCl_3$) δ 207.5, 78.1, 71.4, 64.8, 64.5, 60.0, 53.8, 46.2, 39.6, 32.3, 32.1, 25.8, 22.8.

HRMS (ESI): m/z, $[M+H]^+$ calc. for $C_{13}H_{22}NO_3$: 240.1594; found: 240.1596.

IR (ν/cm^{-1}, thin film) 2961, 2927, 2854, 2797, 1717, 1477, 1453, 1424, 1355, 1298, 1242, 1223, 1207, 1187, 1172, 1153, 1114, 1105, 1074, 1024, 998, 955, 850, 828, 625.

$[\alpha]_D^{25}$ –29.99 ° (c 0.09, CH_2Cl_2).

11. (4a'S,8a'S)-decahydro-2'H-dispiro[pyran-4,3'-benzo[b][1,4]oxazine-7',2''-[1,3]dioxolane]

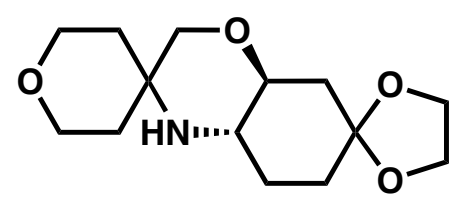

Tetrahydropyran-4-one (2.0 g, 20 mmol, 1.0 equiv) and SnAP reagent **md** (9.5 g, 20 mmol, 1.0 equiv) were allowed to react according to **GP1.1** to afford the morpholine ketal **11** (4.3 g, 16 mmol, 80% yield) as an off-white solid.

^{1}H NMR (400 MHz, $CDCl_3$) δ 4.00 – 3.82 (m, 5H), 3.76 (dtd, *J* = 11.6, 7.7, 3.7 Hz, 2H), 3.57 (dddd, *J* = 26.5, 11.4, 7.0, 3.7 Hz, 2H), 3.21 (d, *J* = 11.3 Hz, 1H), 3.08 (ddd, *J* = 11.8, 9.3, 4.3 Hz, 1H), 2.63 (ddd, *J* = 11.4, 9.2, 3.8 Hz, 1H), 2.01 – 1.86 (m, 2H), 1.81 (ddd, *J* = 13.7, 6.9, 3.6 Hz, 1H), 1.77 – 1.68 (m, 1H), 1.66 – 1.53 (m, 3H), 1.47 (dddd, *J* = 13.6, 7.6, 3.9, 1.0 Hz, 1H), 1.41 – 1.26 (m, 2H), 1.04 (s, 1H).

^{13}C NMR (101 MHz, $CDCl_3$) δ 109.2, 79.6, 75.4, 64.5, 64.5, 64.3, 63.3, 53.2, 49.2, 39.4, 36.5, 33.9, 32.3, 27.7.

HRMS (ESI): m/z, $[M+H]^+$ calc. for $C_{14}H_{24}NO_4$: 270.1700; found: 270.1697.

IR (ν/cm^{-1}, thin film) 2944, 2863, 1442, 1361, 1304, 1237, 1145, 1095, 1067, 1057, 1025, 1003, 947, 934, 922, 843, 735.

$[\alpha]_D^{25}$ +26.43 ° (c 0.06, CH_2Cl_2).

12. (4a'S,8a'S)-4'-methyldecahydro-2'H-dispiro[pyran-4,3'-benzo[b][1,4]oxazine-7',2''-[1,3]dioxolane]

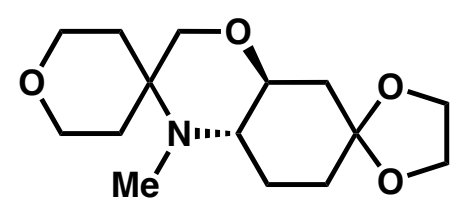

Morpholine ketal **11** (4.3 g, 16 mmol, 1.0 equiv) was allowed to react with formaldehyde (37% aq, 1.4 mL, 19 mmol, 1.2 equiv) and $NaBH(OAc)_3$ (5.1 g, 24 mmol, 1.5 equiv) according to **GP2**. The procedure was repeated with the crude product 1x to reach full conversion of the starting material, affording the N-methylated morpholine **12** (4.5 g, 16 mmol, 100% yield) as an off-white solid.

^{1}H NMR (400 MHz, $CDCl_3$) δ 4.28 (d, *J* = 11.6 Hz, 1H), 3.99 – 3.85 (m, 5H), 3.80 (ddt, *J* = 12.8, 5.4, 1.6 Hz, 1H), 3.54 (td, *J* = 12.1, 2.7 Hz, 1H), 3.47 – 3.33 (m, 2H), 3.30 (dd, *J* = 11.6, 2.0 Hz, 1H), 2.34 (ddd, *J* = 11.4, 9.4, 3.7 Hz, 1H), 2.26 (s, 3H), 2.14 (ddd, *J* = 13.9, 12.4, 5.5 Hz, 1H), 2.00 – 1.87 (m, 3H), 1.82 – 1.69 (m, 2H), 1.63 (t, *J* = 12.2 Hz, 1H), 1.53 (td, *J* = 13.6, 4.3 Hz, 1H), 1.32 (dddd, *J* = 13.8, 12.9, 11.5, 3.8 Hz, 1H), 1.10 (dq, *J* = 13.8, 2.2 Hz, 1H).

^{13}C NMR (101 MHz, $CDCl_3$) δ 108.5, 77.7, 71.2, 64.8, 64.4, 64.4, 64.4, 60.4, 53.6, 39.8, 33.8, 32.4, 31.7, 25.3, 22.8.

HRMS (ESI): m/z, $[M+H]^+$ calc. for $C_{15}H_{26}NO_4$: 284.1856; found: 284.1851.

IR (ν/cm^{-1}, thin film) 2956, 2929, 2857, 2795, 1476, 1446, 1373, 1357, 1313, 1239, 1208, 1191, 1173, 1151, 1104, 1094, 1075, 1046, 1028, 1013, 1001, 980, 947, 932, 922, 833, 661.

$[\alpha]_D^{25}$ +31.25 ° (c 0.08, CH_2Cl_2).

13. (4aS,8aS)-4-methyldecahydro-2H,7H-spiro[benzo[b][1,4]oxazine-3,4'-pyran]-7-one

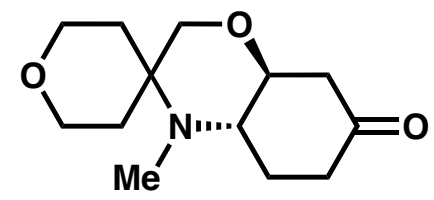

N-methyl morpholine ketal **12** (4.5 g, 16 mmol, 1.0 equiv) was allowed to react with HCl (3.0 M aq, 0.32 L, 0.95 mol, 60 equiv) according to **GP3** to afford the tricyclic morpholine ketone **13** (3.5 g, 15 mmol, 94% yield) as an off-white solid.

^{1}H NMR (400 MHz, $CDCl_3$) δ 4.34 (d, *J* = 11.7 Hz, 1H), 3.95 (ddd, *J* = 12.2, 5.4, 1.6 Hz, 1H), 3.84 (ddd, *J* = 12.0, 5.5, 1.6 Hz, 1H), 3.56 (td, *J* = 12.0, 2.7 Hz, 1H), 3.49 – 3.36 (m, 2H), 3.32 (dt, *J* = 11.6, 1.6 Hz, 1H), 2.76 – 2.61 (m, 2H), 2.52 – 2.36 (m, 2H), 2.36 – 2.12 (m, 6H), 1.97 (dd, *J* = 13.3, 2.5 Hz, 1H), 1.88 – 1.75 (m, 1H), 1.47 – 1.30 (m, 1H), 1.12 (dt, *J* = 13.8, 1.8 Hz, 1H).

^{13}C NMR (101 MHz, $CDCl_3$) δ 207.5, 78.1, 71.4, 64.9, 64.5, 60.0, 53.8, 46.2, 39.7, 32.3, 32.1, 25.8, 22.8.

HRMS (ESI): m/z, $[M+CH_3OH+H]^+$ calc. for $C_{14}H_{26}NO_4$: 272.1856; found: 272.1853.

IR (ν/cm^{-1}, thin film) 2965, 2959, 2947, 2862, 2805, 1712, 1456, 1362, 1310, 1304, 1295, 1223, 1208, 1188, 1175, 1151, 1110, 1101, 1076, 1038, 1028, 1017, 998, 971, 956, 852, 830, 728, 703, 626, 532.

$[\alpha]_D^{25}$ +38.87 ° (c 0.06, CH_2Cl_2).

14. (4a'S,8a'S)-octahydro-2'H,4'H,5'H-dispiro[pyran-4,3'-benzo[b][1,4]oxazine-6',2''-[1,3]dioxolane]

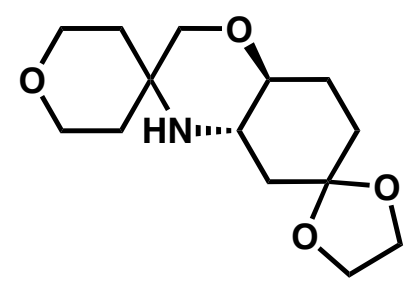

Tetrahydropyran-4-one (6.0 g, 60 mmol, 1.0 equiv) and SnAP reagent **mE** (29 g, 60 mmol, 1.0 equiv) were allowed to react according to **GP1.1** to afford the morpholine ketal **14** (7.1 g, 26 mmol, 44% yield) as a yellow oil.

^{1}H NMR (400 MHz, $CDCl_3$) δ 4.01 – 3.84 (m, 5H), 3.75 (dddd, *J* = 11.5, 9.6, 7.4, 3.8 Hz, 2H), 3.57 (dddd, *J* = 23.1, 11.5, 7.0, 3.8 Hz, 2H), 3.23 (d, *J* = 11.3 Hz, 1H), 2.89 (dtd, *J* = 18.4, 8.6, 3.9 Hz, 2H), 2.00 – 1.69 (m, 5H), 1.60 (ddd, *J* = 8.9, 7.0, 2.9 Hz, 2H), 1.52 – 1.40 (m, 2H), 1.37 – 1.26 (m, 1H), 1.04 (s, 1H).

^{13}C NMR (101 MHz, $CDCl_3$) δ 108.6, 81.7, 75.6, 64.6, 64.5, 64.3, 63.3, 50.9, 49.1, 40.2, 36.4, 33.4, 32.3, 27.2.

HRMS (ESI): m/z, $[M+H]^+$ calc. for $C_{14}H_{24}NO_4$: 270.1700; found: 270.1686.

IR (ν/cm^{-1}, thin film) 2946, 2868, 1446, 1363, 1285, 1245, 1209, 1173, 1150, 1106, 1082, 1023, 947, 928, 845.

$[\alpha]_D^{23}$ –4.03 ° (c 0.11, CH_2Cl_2).

15. (4a'S,8a'S)-4'-methyloctahydro-2'H,4'H,5'H-dispiro[pyran-4,3'-benzo[b][1,4]oxazine-6',2''-[1,3]dioxolane]

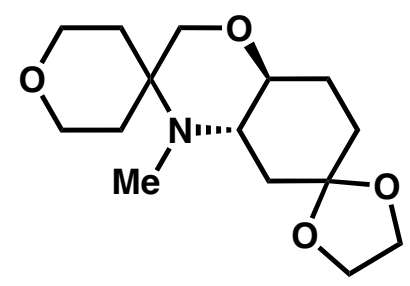

Morpholine ketal **14** (7.0 g, 26 mmol, 1.0 equiv) was allowed to react with formaldehyde (37% aq, 2.4 mL, 31 mmol, 1.2 equiv) and $NaBH(OAc)_3$ (8.3 g, 39 mmol, 1.5 equiv) according to **GP2**. The procedure was repeated with the crude product 1x to reach full conversion of the starting material, affording the N-methylated morpholine **15** (7.2 g, 25 mmol, 98% yield) as an off-white solid.

^{1}H NMR (400 MHz, $CDCl_3$) δ 4.30 (d, J = 11.6 Hz, 1H), 4.02 – 3.86 (m, 5H), 3.81 (ddt, J = 11.7, 5.2, 1.7 Hz, 1H), 3.54 (td, J = 12.1, 2.7 Hz, 1H), 3.43 (td, J = 12.1, 2.4 Hz, 1H), 3.30 (dd, J = 11.6, 2.0 Hz, 1H), 3.20 (ddd, J = 10.8, 9.3, 4.1 Hz, 1H), 2.62 (ddd, J = 12.2, 9.4, 3.9 Hz, 1H), 2.22 (s, 3H), 2.14 (ddd, J = 13.8, 12.4, 5.5 Hz, 1H), 2.01 (ddd, J = 12.8, 3.9, 2.9 Hz, 1H), 1.93 (dq, J = 13.3, 2.3 Hz, 1H), 1.86 – 1.71 (m, 3H), 1.70 – 1.52 (m, 2H), 1.42 (t, J = 12.5 Hz, 1H), 1.10 (dq, J = 13.8, 2.2 Hz, 1H).

^{13}C NMR (101 MHz, $CDCl_3$) δ 108.9, 79.8, 71.3, 64.9, 64.6, 64.5, 64.5, 57.8, 53.6, 38.2, 32.8, 32.5, 31.5, 27.7, 23.0.

HRMS (ESI): m/z, $[M+H]^+$ calc. for $C_{15}H_{26}NO_4$: 284.1856; found: 284.1850.

IR (ν/cm^{-1}, thin film) 2952, 2852, 1475, 1446, 1373, 1354, 1294, 1250, 1208, 1173, 1145, 1104, 1046, 1027, 1016, 1001, 958, 948, 927, 838, 801, 689, 625

$[\alpha]_D^{23}$ +70 ° (c 0.07, CH_2Cl_2).

16. (4aS,8aS)-4-methyloctahydro-2H,4H-spiro[benzo[b][1,4]oxazine-3,4'-pyran]-6(5H)-one

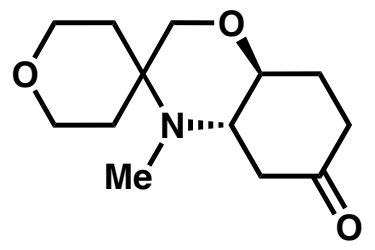

N-methyl morpholine ketal **15** (7.2 g, 25 mmol, 1.0 equiv) was allowed to react with HCl (3.0 M aq, 0.51 L, 1.5 mol, 60 equiv) according to **GP3** to afford the tricyclic morpholine ketone **16** (6.1 g, 25 mmol, 100% yield) as an off-white solid.

^{1}H NMR (400 MHz, $CDCl_3$) δ 4.37 (d, J = 11.6 Hz, 1H), 3.91 (ddt, J = 11.9, 5.5, 1.5 Hz, 1H), 3.82 (ddt, J = 11.9, 5.6, 1.6 Hz, 1H), 3.60 – 3.48 (m, 2H), 3.43 (td, J = 12.2, 2.4 Hz, 1H), 3.34 (dd, J = 11.7, 2.1 Hz, 1H), 2.74 – 2.59 (m, 2H), 2.47 – 2.32 (m, 2H), 2.28 – 2.06 (m, 6H), 1.91 – 1.81 (m, 1H), 1.80 – 1.63 (m, 2H), 1.09 (dd, J = 13.8, 2.2 Hz, 1H).

^{13}C NMR (101 MHz, $CDCl_3$) δ 208.1, 78.8, 71.5, 64.8, 64.4, 59.0, 53.5, 44.4, 38.9, 32.3, 31.5, 28.3, 22.5.

HRMS (ESI): m/z, $[M+H]^+$ calc. for $C_{13}H_{22}NO_3$: 240.1594; found: 240.1592.

IR (ν/cm^{-1}, thin film) 2957, 2926, 2851, 2797, 1712, 1459, 1428, 1355, 1315, 1290, 1256, 1241, 1199, 1172, 1124, 1104, 1071, 1052, 1023, 1009, 1000, 956, 903, 853, 841, 631.

$[\alpha]_D^{23}$ +23.95 ° (c 0.06, CH_2Cl_2).

4.6 Synthesis of pentacycles

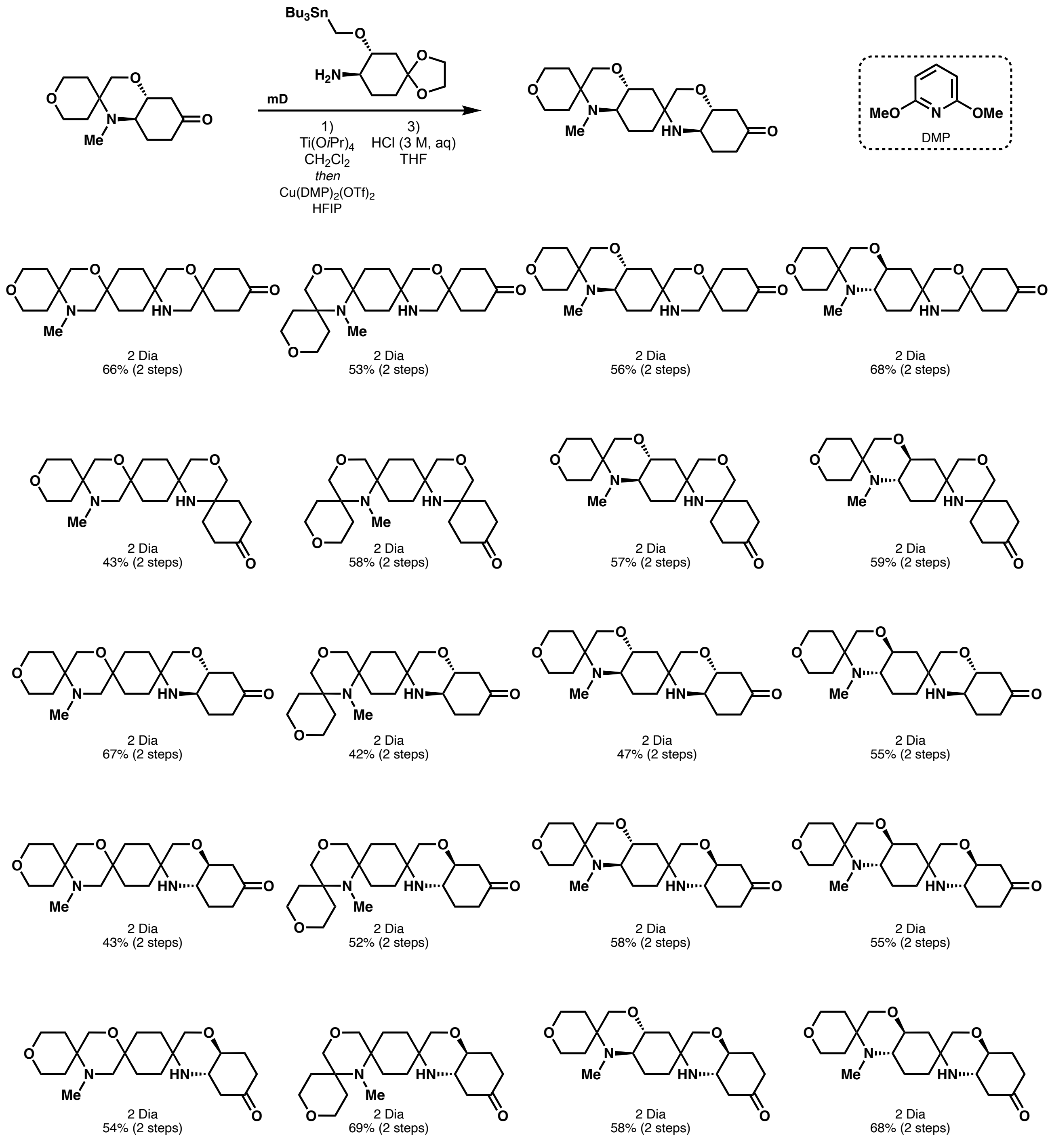

Scheme 4.6.1: Overview on the assembly of the second morpholines from morpholine containing tricycles and the SnAP reagents **mA**–**mE** *(Figure 4.3.1).*

17. 23-methyl-1,4,9,15,20-pentaoxa-23,27-diazapentaspiro-[4.2.2.2.2.5^{17}.2^{14}.2^{11}.2^{8}.2^{5}]triacontane

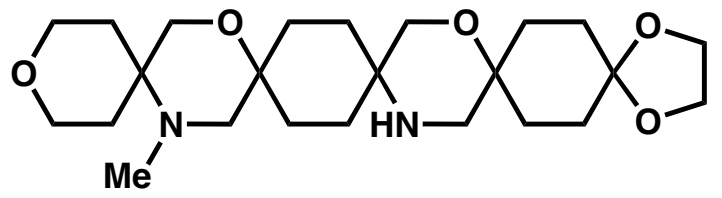

N-methyl morpholine ketone **4** (1.3 g, 5.0 mmol, 1.0 equiv) and SnAP reagent **mA** (2.5 g, 5.0 mmol, 1.0 equiv) were allowed to react according to **GP1.1** to afford the morpholine ketal **17** as two separated diastereomers with 1.4:1 d.r., both isolated as white solids.

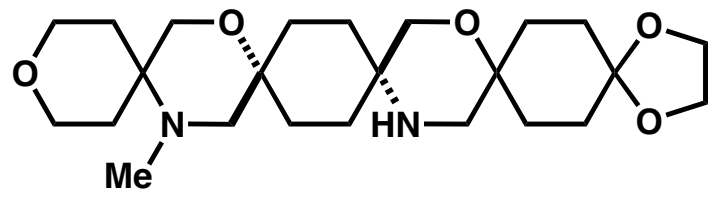

(11s,14s)-23-methyl-1,4,9,15,20-pentaoxa-23,27-diazapentaspiro-[4.2.2.2.2.5^{17}.2^{14}.2^{11}.2^{8}.2^{5}]-triacontane **17a** (0.87 g, 2.0 mmol, 40% yield, unpolar, major diastereomer, white solid)

The relative configuration was elucidated by comparison of experimental to predicted ^{13}C NMR chemical shifts and confirmed by X-Ray crystallography (*vide infra*).

^{1}H NMR (400 MHz, $CDCl_3$) δ 3.98 – 3.89 (m, 4H), 3.86 (dd, *J* = 11.7, 5.4 Hz, 2H), 3.68 (s, 2H), 3.49 (dd, *J* = 12.2, 2.2 Hz, 2H), 3.45 (s, 2H), 2.70 (s, 2H), 2.39 (s, 2H), 2.23 (s, 3H), 2.01 (d, *J* = 13.0 Hz, 2H), 1.96 – 1.73 (m, 6H), 1.64 – 1.31 (m, 13H).

^{13}C NMR (126 MHz, $CDCl_3$) δ 109.3, 71.7, 70.0, 67.0, 64.7, 64.7, 64.5, 64.3, 57.8, 52.8, 49.9, 49.2, 37.0, 30.1, 29.8, 29.2, 28.9, 26.3

HRMS (ESI): m/z, $[M+H]^+$ calc. for $C_{24}H_{41}N_2O_5$: 437.3010; found: 437.3003.

IR (ν/cm^{-1}, thin film) 3251, 2934, 2863, 2795, 1440, 1371, 1292, 1221, 1195, 1176, 1099, 1080, 1066, 1054, 1029, 975, 937, 917, 872, 856, 846, 831, 798, 763, 734, 667, 618, 598.

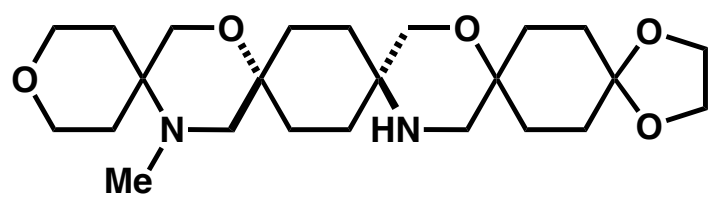

(11r,14r)-23-methyl-1,4,9,15,20-pentaoxa-23,27-diazapentaspiro-[4.2.2.2.2.5^{17}.2^{14}.2^{11}.2^{8}.2^{5}]-triacontane **17b** (0.63 g, 1.4 mmol, 29% yield, polar, minor diastereomer, white solid)

The relative configuration was elucidated by comparison of experimental to predicted ^{13}C NMR chemical shifts and confirmed by X-Ray crystallography (*vide infra*).

^{1}H NMR (400 MHz, $CDCl_3$) δ 3.99 – 3.89 (m, 4H), 3.85 (dd, *J* = 11.9, 5.2 Hz, 2H), 3.67 (s, 2H), 3.48 (td, *J* = 12.3, 2.2 Hz, 2H), 3.33 (s, 2H), 2.62 (s, 2H), 2.40 (s, 2H), 2.22 (s, 3H), 1.97 (d, *J* = 13.3 Hz, 2H), 1.95 – 1.85 (m, 2H), 1.85 – 1.74 (m, 4H), 1.58 – 1.38 (m, 12H), 1.34 (d, *J* = 5.4 Hz, 1H).

^{13}C NMR (126 MHz, $CDCl_3$) δ 109.1, 71.6, 70.1, 69.3, 64.6, 64.5, 64.3, 64.1, 58.6, 52.7, 49.3, 49.1, 36.8, 29.9, 29.5, 27.7, 27.4, 26.1.

HRMS (ESI): m/z, $[M+H]^+$ calc. for $C_{24}H_{41}N_2O_5$: 437.3010; found: 437.3004.

IR (ν/cm^{-1}, thin film) 3321, 2941, 2856, 1436, 1374, 1302, 1223, 1169, 1103, 1091, 1059, 1030, 973, 930, 873, 856, 831, 781, 663, 604.

18a. *(9s,12s)-7-methyl-3,21,25-trioxa-7,13-diazatetraspiro[5.2.2.2.5^{15}.2^{12}.2^{9}.2^{6}]-hexacosan-18-one*

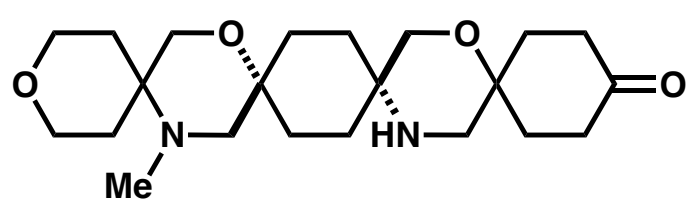

Pentacyclic ketals **17a** (0.87 g, 2.0 mmol, 1.0 equiv) was allowed to react separately with HCl (1.0 M aq, 10 mL, 10 mmol, 5.0 equiv) according to **GP3** to afford the pentacyclic morpholine ketone **18a** (0.76 g, 1.9 mmol, 97% yield) as a white solid.

The relative configuration was elucidated in the previous step.

^{1}H NMR (400 MHz, $CDCl_3$) δ 3.86 (dd, *J* = 12.0, 5.3 Hz, 2H), 3.68 (s, 2H), 3.53 – 3.41 (m, 4H), 2.75 (s, 2H), 2.56 (td, *J* = 14.3, 6.1 Hz, 2H), 2.40 (s, 2H), 2.39 – 2.29 (m, 2H), 2.26 – 2.17 (m, 5H), 1.95 – 1.85 (m, 4H), 1.67 – 1.35 (m, 10H), 1.20 (s, 1H).

^{13}C NMR (101 MHz, $CDCl_3$) δ 212.1, 71.6, 69.7 (2C), 67.4, 64.7, 57.8, 52.8, 49.9, 48.8, 37.0, 36.4, 31.9, 29.2, 28.8, 26.2.

HRMS (ESI): m/z, $[M+CH_3OH+H]^+$ calc. for $C_{23}H_{41}N_2O_5$: 425.3010; found: 425.3002.

IR (ν/cm^{-1}, thin film) 3324, 2925, 2859, 2805, 2241, 1712, 1441, 1354, 1292, 1237, 1223, 1200, 1179, 1159, 1128, 1117, 1100, 1069, 1025, 992, 976, 909, 867, 853, 841, 829, 726, 708, 646, 617, 597, 504.

18b ***(9r,12r)-7-methyl-3,21,25-trioxa-7,13-diazatetraspiro[5.2.2.2.5^{15}.2^{12}.2^{9}.2^{6}]-hexacosan-18-one***

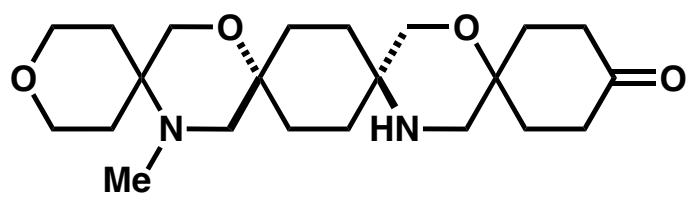

Pentacyclic ketals **17b** (0.63 g, 1.4 mmol, 1.0 equiv) was allowed to react with HCl (1.0 M aq, 7.2 mL, 7.2 mmol, 5.0 equiv) according to **GP3** to afford the pentacyclic morpholine ketone **18b** (0.54 g, 1.4 mmol, 95% yield) as a white solid.

The relative configuration was elucidated in the previous step.

^{1}H NMR (400 MHz, $CDCl_3$) δ 3.86 (dd, *J* = 11.9, 5.3 Hz, 2H), 3.68 (s, 2H), 3.48 (td, *J* = 12.3, 2.2 Hz, 2H), 3.40 (s, 2H), 2.68 (s, 2H), 2.58 (td, *J* = 14.1, 5.9 Hz, 2H), 2.41 (s, 2H), 2.35 – 2.27 (m, 2H), 2.26 – 2.17 (m, 5H), 1.97 – 1.80 (m, 4H), 1.64 – 1.45 (m, 10H), 1.15 (s, 1H).

^{13}C NMR (101 MHz, $CDCl_3$) δ 212.0, 71.7, 70.7, 69.1, 64.7 (2 C), 58.8, 52.8, 49.5, 48.8, 37.0, 36.4, 31.8, 27.8, 27.4, 26.3.

HRMS (ESI): m/z, $[M+H]^+$ calc. for $C_{22}H_{37}N_2O_4$: 393.2748; found: 393.2743.

IR (ν/cm^{-1}, thin film) 3319, 2943, 2854, 2241, 1710, 1456, 1222, 1179, 1103, 1078, 1056, 1026, 917, 851, 729.

19. 22-methyl-1,4,14,19,27-pentaoxa-9,22-diazapentaspiro[4.2.1.2.2.5^{16}.2^{13}.2^{10}.3^{8}.2^{5}]-triacontane

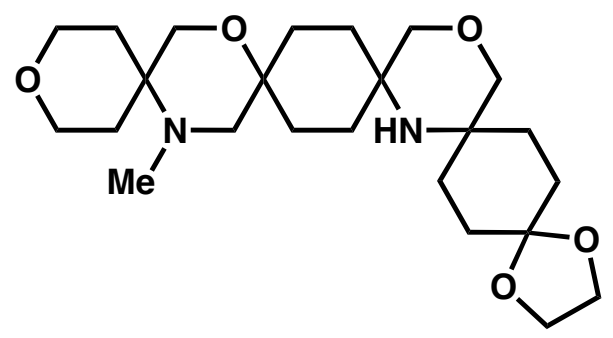

N-methyl morpholine ketone **4** (1.5 g, 6.0 mmol, 1.0 equiv) and SnAP reagent **mB** (2.9 g, 6.0 mmol, 1.0 equiv) were allowed to react according to **GP1.2** to afford the morpholine ketal **19** as a mixture of 2 diastereomers (1.2 g, 2.8 mmol, 47% yield, 1.5:1 d.r. by ^{1}H NMR [CH_2 at 2.42 and 2.36 ppm]) as white solid. Assignment to diastereomers could not be established from the mixture.

^{1}H NMR (400 MHz, $CDCl_3$) δ 3.91 (tt, *J* = 5.2, 2.5 Hz, 10.2H), 3.85 (dd, *J* = 11.8, 5.2 Hz, 5.3H), 3.67 (d, *J* = 5.1 Hz, 4.8H), 3.53 – 3.43 (m, 8.3H), 3.43 – 3.33 (m, 6.5H), 2.42 (s, 2.2H), 2.36 (s, 3.2H), 2.23 (d, *J* = 9.3 Hz, 7.5H), 1.86 (dddt, *J* = 27.3, 23.4, 17.9, 9.0 Hz, 16.3H), 1.59 (dddd, *J* = 44.4, 23.8, 16.3, 10.4 Hz, 32.2H), 1.30 (ddd, *J* = 14.9, 9.7, 5.5 Hz, 2.9H). Peaks reported as mixture of diastereomers (overlapped: total integral).

^{13}C NMR (101 MHz, $CDCl_3$) δ 109.0, 108.9, 78.6, 77.4, 77.0, 75.2, 71.7, 71.1, 64.7, 64.6, 64.3, 64.3, 64.3, 58.4, 58.1, 52.9, 50.5, 49.8, 49.7, 49.2, 37.0, 36.9, 34.3, 34.0, 32.1, 31.5, 30.6, 29.5, 28.4, 26.2. Peaks reported as mixture of diastereomers.

HRMS (ESI): m/z, $[M+H]^+$ calc. for $C_{24}H_{41}N_2O_5$: 437.3010; found: 437.2999.

20. 20-methyl-12,17,25-trioxa-7,20-diazatetraspiro[5.1.2.2.5^14^.2^11^.2^8^.3^6^]hexacosan-3-one

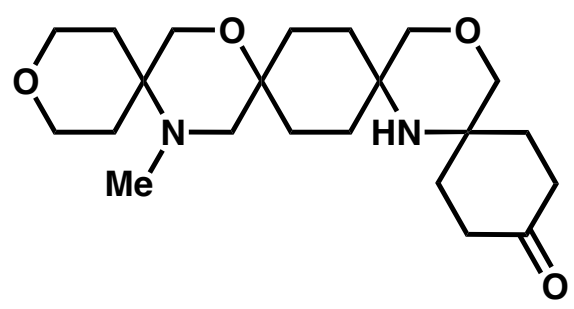

Pentacyclic ketal (mixture of 2 diastereomers) **19** (1.2 g, 2.8 mmol, 1.0 equiv) was allowed to react with HCl (3.0 M aq, 55 mL, 0.17 mol, 60 equiv) according to **GP3** to afford the pentacyclic morpholine ketone **20** (1.0 g, 2.5 mmol, 91% yield, 1.6:1 d.r. by ^{1}H NMR [CH_2 at 2.42 and 2.36 ppm]) as white solid. Assignment to diastereomers could not be established from the mixture.

^{1}H NMR (400 MHz, $CDCl_3$) δ 3.89 – 3.80 (m, 5.3H), 3.67 (d, *J* = 6.6 Hz, 4.7H), 3.51 (s, 3.2H), 3.48 (dd, *J* = 12.2, 2.1 Hz, 5.5H), 3.41 (d, *J* = 1.4 Hz, 6.7H), 2.73 – 2.58 (m, 5.4H), 2.42 (s, 2.0H), 2.36 (s, 3.3H), 2.29 – 2.16 (m, 13.3H), 2.03 – 1.83 (m, 14.0H), 1.78 (d, *J* = 5.1 Hz, 2.2H), 1.74 – 1.53 (m, 16.1H), 1.52 – 1.42 (m, 6.9H), 1.28 (ddd, *J* = 14.1, 11.7, 4.3 Hz, 3.2H), 0.78 (s, 2.0H). Peaks reported as mixture of diastereomers (overlapped: total integral).

^{13}C NMR (101 MHz, $CDCl_3$) δ 211.8, 211.7, 78.7, 77.4, 77.3, 75.3, 71.6, 71.0, 64.7, 64.7, 58.3, 52.8, 50.3, 49.8, 49.3, 49.0, 37.0, 37.0, 36.8, 36.8, 36.0, 35.7, 31.6, 31.4, 29.4, 28.6, 26.2. Peaks reported as mixture of diastereomers.

HRMS (ESI): m/z, $[M+H]^+$ calc. for $C_{22}H_{37}N_2O_4$: 393.2748; found: 393.2739.

21. (4a'''R,8a'''R)-4'-methyldecahydro-2'''H-tetraspiro[pyran-4,5'-morpholine-2',1''-cyclohexane-4'',3'''-benzo[b][1,4]oxazine-7''',2''''-[1,3]dioxolane]

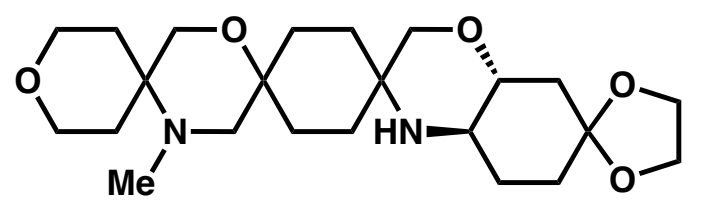

N-methyl morpholine ketone **4** (1.3 g, 5.0 mmol, 1.0 equiv) and SnAP reagent **mD** (2.4 g, 5.0 mmol, 1.0 equiv) were allowed to react according to **GP1.1** to afford the morpholine ketal **21** as a mixture of 2 diastereomers (1.5 g,3.5 mmol, 71% yield, 1.6:1 d.r. by ^{1}H NMR [CH at 2.68 and 2.54 ppm]) as white solid. Assignment to diastereomers could not be established from the mixture.

^{1}H NMR (400 MHz, $CDCl_3$) δ 3.98 – 3.80 (m, 17.0H), 3.73 – 3.61 (m, 6.7H), 3.47 (tdd, *J* = 12.3, 6.1, 2.4 Hz, 5.2H), 3.23 – 3.14 (m, 2.5H), 3.05 (dddd, *J* = 23.6, 11.9, 9.2, 4.3 Hz, 2.6H), 2.68 (ddd, *J* = 11.3, 9.2, 3.8 Hz, 1.0H), 2.54 (ddd, *J* = 11.7, 9.2, 3.6 Hz, 1.6H), 2.47 – 2.32 (m, 5.2H), 2.22 (s, 7.4H), 2.01 – 1.69 (m, 18.4H), 1.64 – 1.19 (m, 30.5H), 1.17 – 1.05 (m, 2.1H). Peaks reported as mixture of diastereomers (overlapped: total integral).

^{13}C NMR (101 MHz, $CDCl_3$) δ 109.3, 109.2, 79.7, 79.5, 73.8, 71.8, 71.5, 64.7, 64.7, 64.7, 64.6, 64.5, 64.5, 58.4, 57.9, 53.5, 53.4, 52.8, 52.8, 51.4, 51.0, 39.5, 39.4, 37.0, 37.0, 34.0, 33.8, 32.1, 31.1, 29.5, 29.0, 28.5, 27.8, 27.7, 27.6, 26.7, 26.6, 26.3, 26.2, 25.9, 25.4. Peaks reported as mixture of diastereomers.

HRMS (ESI): m/z, $[M+H]^+$ calc. for $C_{23}H_{39}N_2O_5$: 423.2853; found: 423.2848.

22. (4aR,8aR)-4''-methyldecahydro-2H,7H-trispiro[benzo[b][1,4]oxazine-3,1'-cyclohexane-4',2''-morpholine-5'',4'''-pyran]-7-one

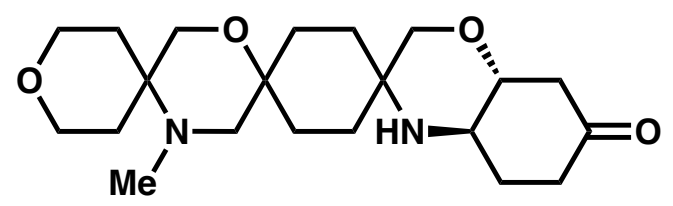

Pentacyclic ketal (mixture of 2 diastereomers) **21** (1.1 g, 2.6 mmol, 1.0 equiv) was allowed to react with HCl (sat., 13 mL, 0.16 mol, 60 equiv) according to **GP3** to afford the pentacyclic morpholine ketone **22** (0.94 g, 2.5 mmol, 95% yield, 1.7:1 d.r. by ^{1}H NMR [CH at 2.94 and 2.68 ppm]) as white solid. Assignment to diastereomers could not be established from the mixture.

^{1}H NMR (400 MHz, $CDCl_3$) δ 3.98 – 3.82 (m, 7.1H), 3.76 – 3.63 (m, 7.0H), 3.49 (tdd, *J* = 12.2, 5.8, 2.3 Hz, 5.4H), 3.28 – 3.20 (m, 2.6H), 3.11 (dddd, *J* = 21.4, 14.0, 8.9, 4.8 Hz, 3.6H), 2.94 (ddd, *J* = 12.8, 9.3, 3.7 Hz, 1.5H), 2.68 (ddt, *J* = 14.4, 4.9, 1.9 Hz, 2.6H), 2.51 – 2.31 (m, 13.5H), 2.25 (d, *J* = 2.4 Hz, 8.2H), 2.13 – 1.78 (m, 16.9H), 1.70 – 1.23 (m, 24.2H), 1.11 (d, *J* = 11.6 Hz, 1.6H). Peaks reported as mixture of diastereomers (overlapped: total integral). Calibrated integral with overlapping CH_3 signal at 2.25 ppm.

^{13}C NMR (101 MHz, $CDCl_3$) δ 207.6, 207.6, 79.4, 79.1, 77.0, 73.2, 71.4, 71.3, 64.6, 64.5, 64.5, 64.4, 64.3, 58.4, 57.7, 52.7, 52.7, 51.3, 50.8, 46.1, 45.9, 39.8, 39.6, 36.8, 31.9, 30.6, 29.4, 28.8, 28.3, 27.8, 27.7, 27.3, 26.6, 26.4, 26.1, 25.8, 25.1. Peaks reported as mixture of diastereomers.

HRMS (ESI): m/z, $[M+H]^+$ calc. for $C_{21}H_{35}N_2O_4$: 379.2591; found: 379.2588.

23. (4a'''S,8a'''S)-4'-methyldecahydro-2'''H-tetraspiro[pyran-4,5'-morpholine-2',1''-cyclohexane-4'',3'''-benzo[b][1,4]oxazine-7''',2''''-[1,3]dioxolane]

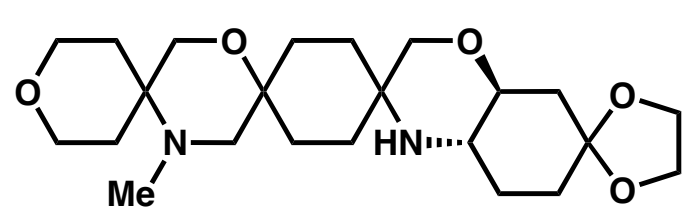

N-methyl morpholine ketone **4** (1.1 g, 4.2 mmol, 1.0 equiv) and SnAP reagent **md** (2.0 g, 4.2 mmol, 1.0 equiv) were allowed to react according to **GP1.1** to afford the morpholine ketal **23** as a mixture of 2 diastereomers (0.92 g, 2.2 mmol, 52% yield, 1.5:1 d.r. by ^{1}H NMR [CH at 2.71 and 2.58 ppm]) as white solid. Assignment to diastereomers could not be established from the mixture.

^{1}H NMR (400 MHz, $CDCl_3$) δ 3.98 – 3.81 (m, 17.0H), 3.72 – 3.61 (m, 6.4H), 3.47 (tdd, *J* = 12.1, 5.3, 2.2 Hz, 5.0H), 3.29 – 3.19 (m, 2.4H), 3.17 – 3.04 (m, 2.4H), 2.71 (ddd, *J* = 11.5, 9.2, 3.6 Hz, 1.0H), 2.58 (ddd, *J* = 12.5, 9.4, 3.6 Hz, 1.6H), 2.50 – 2.34 (m, 5.3H), 2.22 (d, *J* = 1.9 Hz, 7.3H), 2.02 – 1.84 (m, 12.1H), 1.83 – 1.22 (m, 36.0H), 1.21 – 1.14 (m, 1.9H). Peaks reported as mixture of diastereomers (overlapped: total integral).

^{13}C NMR (101 MHz, $CDCl_3$) δ 109.1, 109.1, 79.3, 79.2, 73.4, 71.7, 71.4, 64.7, 64.7, 64.6, 64.6, 64.5, 64.5, 58.2, 58.0, 53.6, 53.5, 52.9, 52.8, 51.9, 39.5, 39.5, 37.0, 37.0, 34.0, 33.8, 31.8, 30.7, 29.5, 28.9, 28.4, 27.5, 27.5, 27.3, 26.5, 26.5, 26.3, 26.2, 26.0, 25.3. Peaks reported as mixture of diastereomers.

HRMS (ESI): m/z, $[M+H]^+$ calc. for $C_{23}H_{39}N_2O_5$: 423.2853; found: 423.2848.

24. (4aS,8aS)-4''-methyldecahydro-2H,7H-trispiro[benzo[b][1,4]oxazine-3,1'-cyclohexane-4',2''-morpholine-5'',4'''-pyran]-7-one

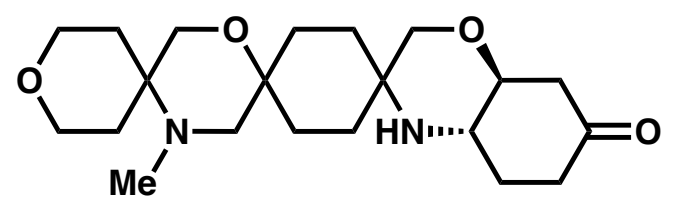

Pentacyclic ketal (mixture of 2 diastereomers) **23** (0.87 g, 2.0 mmol, 1.0 equiv) was allowed to react with HCl (3.0 M, 39 mL, 0.12 mol, 60 equiv) according to **GP3** to afford the pentacyclic morpholine ketone **24** (0.62 g, 1.6 mmol, 82% yield, 1.7:1 d.r. by ^{1}H NMR [CH at 2.92 and 2.65 ppm]) as white solid. Assignment to diastereomers could not be established from the mixture.

^{1}H NMR (400 MHz, $CDCl_3$) δ 3.95 – 3.80 (m, 6.9H), 3.74 – 3.61 (m, 7.3H), 3.47 (tdd, *J* = 12.2, 5.9, 2.4 Hz, 5.6H), 3.26 – 3.18 (m, 2.7H), 3.16 – 3.02 (m, 3.7H), 2.92 (ddd, *J* = 11.5, 9.2, 3.7 Hz, 1.6H), 2.65 (ddt, *J* = 14.3, 4.9, 1.9 Hz, 2.7H), 2.49 – 2.28 (m, 14.3H), 2.23 (d, *J* = 2.1 Hz, 8.1H), 2.11 – 1.77 (m, 17.3H), 1.69 – 1.06 (m, 26.7H). Peaks reported as mixture of diastereomers (overlapped: total integral). Calibrated integral with overlapping CH_3 signal at 2.23 ppm.

^{13}C NMR (101 MHz, $CDCl_3$) δ 207.8, 207.7, 79.5, 79.3, 77.1, 73.3, 71.6, 71.4, 64.7, 64.7, 64.6, 58.5, 57.9, 52.9, 52.8, 51.4, 51.0, 46.2, 46.1, 40.0, 39.8, 37.0, 32.0, 30.7, 29.6, 29.0, 28.4, 27.9, 27.8, 27.5, 26.8, 26.5, 26.3, 26.2, 25.9, 25.2. Peaks reported as mixture of diastereomers.

HRMS (ESI): m/z, $[M+H]^+$ calc. for $C_{21}H_{35}N_2O_4$: 379.2591; found: 379.2593.

25. (4a'''S,8a'''S)-4'-methyloctahydro-2'''H,4'''H,5'''H-tetraspiro[pyran-4,5'-morpholine-2',1''-cyclohexane-4'',3'''-benzo[b][1,4]oxazine-6''',2''''-[1,3]dioxolane]

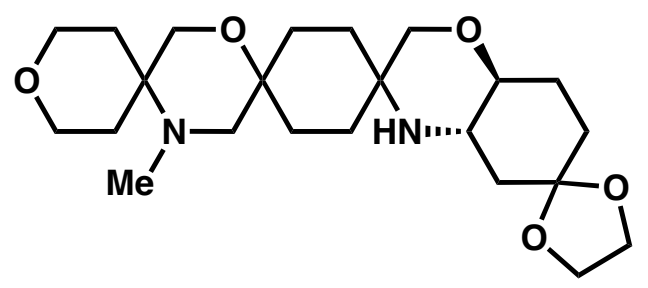

N-methyl morpholine ketone **4** (1.3 g, 5.0 mmol, 1.0 equiv) and SnAP reagent **mE** (2.4 g, 5.0 mmol, 1.0 equiv) were allowed to react according to **GP1.1** to afford the morpholine ketal **25** as a mixture of 2 diastereomers (1.3 g, 3.0 mmol, 59% yield, 1.5:1 d.r. by ^{1}H NMR [CH_3 at 2.22 and 2.21 ppm, partial overlap]) as white solid. Assignment to diastereomers could not be established from the mixture.

^{1}H NMR (400 MHz, $CDCl_3$) δ 3.99 – 3.79 (m, 16.2H), 3.75 – 3.57 (m, 6.2H), 3.54 – 3.40 (m, 4.8H), 3.22 (d, *J* = 11.3 Hz, 2.1H), 3.01 – 2.73 (m, 4.3H), 2.46 – 2.36 (m, 4.6H), 2.22 (s, 3.0H), 2.21 (s, 3.7H), 2.01 – 1.01 (m, 48.0H). Peaks reported as mixture of diastereomers (overlapped: total integral).

^{13}C NMR (101 MHz, $CDCl_3$) δ 108.5, 81.8, 81.3, 74.0, 71.7, 71.5, 64.7, 64.7, 64.6, 64.6, 64.6, 64.6, 64.5, 64.5, 58.3, 57.9, 52.8, 51.2, 40.1, 37.0, 37.0, 33.4, 33.4, 32.0, 30.9, 29.3, 29.2, 28.7, 27.3, 27.2, 26.9, 26.6, 26.5, 26.0, 25.6, 25.3. Peaks reported as mixture of diastereomers.

HRMS (ESI): m/z, $[M+H]^+$ calc. for $C_{23}H_{39}N_2O_5$: 423.2853; found: 423.2844.

26. (4aS,8aS)-4''-methyloctahydro-2H,4H-trispiro[benzo[b][1,4]oxazine-3,1'-cyclohexane-4',2''-morpholine-5'',4'''-pyran]-6(5H)-one

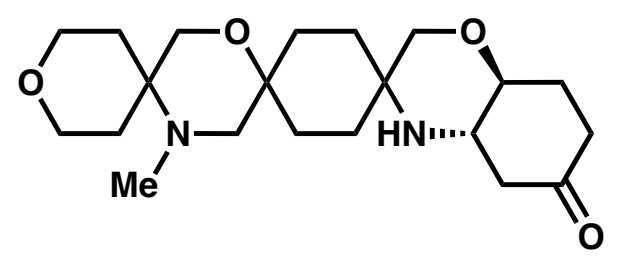

Pentacyclic ketal (mixture of 2 diastereomers) **25** (1.2 g, 2.8 mmol, 1.0 equiv) was allowed to react with HCl (3.0 M, 57 mL, 0.17 mol, 60 equiv) according to **GP3** to afford the pentacyclic morpholine ketone **26** (0.98 g, 2.6 mmol, 92% yield, 1.5:1 d.r. by ^{1}H NMR [CH at 3.04 and 2.88 ppm]) as white solid. Assignment to diastereomers could not be established from the mixture.

^{1}H NMR (400 MHz, $CDCl_3$) δ 3.92 (d, *J* = 11.2 Hz, 1.1H), 3.85 (dt, *J* = 10.5, 4.5 Hz, 4.7H), 3.73 – 3.60 (m, 5.9H), 3.47 (tdd, *J* = 12.3, 5.4, 2.4 Hz, 4.7H), 3.38 – 3.21 (m, 4.6H), 3.04 (ddd, *J* = 12.2, 9.1, 5.3 Hz, 1.0H), 2.88 (ddd, *J* = 13.4, 9.2, 4.5 Hz, 1.4H), 2.49 – 2.29 (m, 12.1H), 2.29 – 2.17 (m, 8.9H), 2.10 (m, 2.6H), 2.00 (dd, *J* = 13.0, 4.7 Hz, 1.2H), 1.97 – 1.04 (m, 32.9H). Peaks reported as mixture of diastereomers (overlapped: total integral).

^{13}C NMR (101 MHz, $CDCl_3$) δ 207.8, 80.2, 79.8, 77.5, 73.6, 71.5, 71.4, 64.7, 64.6, 58.5, 57.9, 52.9, 52.5, 52.5, 51.2, 50.8, 46.1, 46.1, 39.3, 39.3, 37.0, 36.9, 31.9, 30.6, 29.5, 29.0, 28.3, 28.0, 28.0, 27.4, 26.7, 26.6, 26.3, 26.1, 25.9, 25.1. Peaks reported as mixture of diastereomers.

HRMS (ESI): m/z, $[M+H]^+$ calc. for $C_{21}H_{35}N_2O_4$: 379.2591; found: 379.2588.

27. 15-methyl-1,4,9,19,23-pentaoxa-15,27-diazapentaspiro[4.2.2.2.1.5^{16}.3^{14}.2^{11}.2^{8}.2^{5}]triacontane

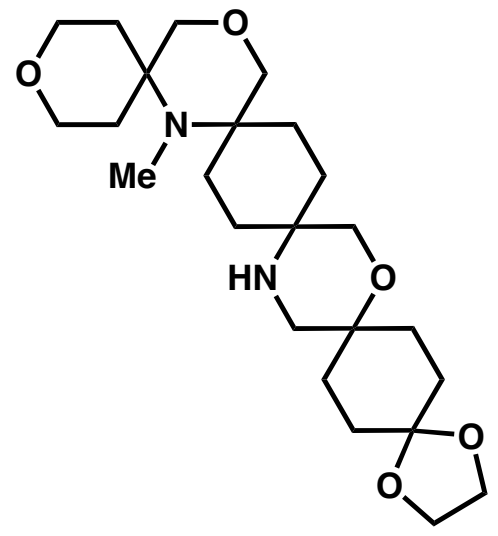

N-methyl morpholine ketone **7** (1.1 g, 4.5 mmol, 1.0 equiv) and SnAP reagent **mA** (2.2 g, 4.5 mmol, 1.0 equiv) were allowed to react according to **GP1.1** to afford the morpholine ketal **27** as a mixture of 2 diastereomers (1.3 g, 3.0 mmol, 65% yield, 1.4:1 d.r. by ^{1}H NMR [CH_2 at 2.68 and 2.61 ppm]) as off-white solid. Assignment to diastereomers could not be established from the mixture.

^{1}H NMR (400 MHz, $CDCl_3$) δ 3.98 – 3.86 (m, 12.5H), 3.85 – 3.77 (m, 4.8H), 3.72 (d, *J* = 2.8 Hz, 4.8H), 3.65 (s, 2.5H), 3.60 (s, 2.1H), 3.54 (s, 3.3H), 3.41 (tt, *J* = 12.4, 2.3 Hz, 5.0H), 3.23 (s, 2.0H), 2.68 (s, 2.8H), 2.61 (s, 2.0H), 2.34 (s, 2.8H), 2.28 (s, 4.1H), 2.15 – 1.07 (m, 61.7H). Peaks reported as mixture of diastereomers (overlapped: total integral).

^{13}C NMR (101 MHz, $CDCl_3$) δ 109.3, 109.1, 74.1, 74.0, 73.5, 70.8, 69.6, 69.5, 65.0, 64.9, 64.9, 64.5, 64.5, 64.3, 55.7, 53.1, 49.6, 49.2, 49.0, 48.5, 31.2, 31.2, 30.4, 30.0, 30.0, 29.7, 28.9, 27.1, 26.8, 26.4, 25.0, 24.9. Peaks reported as mixture of diastereomers.

HRMS (ESI): m/z, $[M+H]^+$ calc. for $C_{24}H_{41}N_2O_5$: 437.3010; found: 437.3004.

28. 7-methyl-3,20,25-trioxa-7,12-diazatetraspiro[5.1.2.2.5^14^.2^11^.2^8^.3^6^]hexacosan-17-one

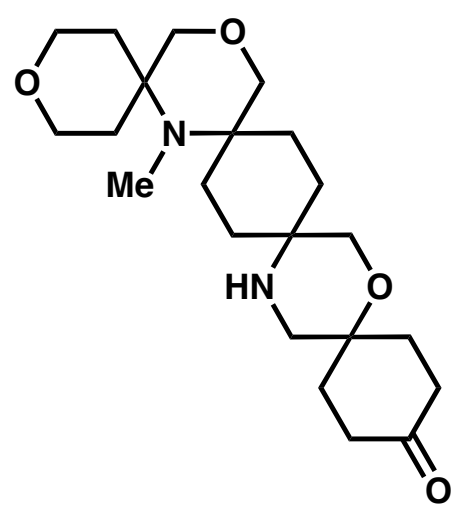

Pentacyclic ketal (mixture of 2 diastereomers) **27** (1.2 g, 2.8 mmol, 1.0 equiv) was allowed to react with HCl (3.0 M, 55 mL, 0.16 mol, 60 equiv) according to **GP3** to afford the pentacyclic morpholine ketone **28** (0.89 g, 2.3 mmol, 82% yield, 1.4:1 d.r. by ^{1}H NMR [CH at 2.74 and 2.67ppm]) as white solid. Assignment to diastereomers could not be established from the mixture.

^{1}H NMR (400 MHz, $CDCl_3$) δ 3.87 – 3.77 (m, 4.8H), 3.73 (d, *J* = 3.5 Hz, 4.6H), 3.65 (s, 2.7H), 3.61 (d, *J* = 3.9 Hz, 4.5H), 3.42 (tt, *J* = 12.4, 2.1 Hz, 4.9H), 3.29 (s, 1.9H), 2.74 (s, 2.7H), 2.67 (s, 2.0H), 2.55 (ddt, *J* = 17.3, 9.4, 3.1 Hz, 5.2H), 2.39 – 2.15 (m, 17.4H), 2.08 (tdd, *J* = 13.6, 8.9, 5.7 Hz, 5.4H), 1.99 – 1.87 (m, 4.8H), 1.78 (td, *J* = 14.1, 4.5 Hz, 5.3H), 1.62 – 1.10 (m, 23.1H). Peaks reported as mixture of diastereomers (overlapped: total integral).

^{13}C NMR (101 MHz, $CDCl_3$) δ 211.9, 211.7, 74.0, 73.5, 71.2, 69.4, 69.2, 65.6, 64.9, 64.9, 55.7, 53.1, 49.5, 48.9, 48.6, 48.5, 36.4, 36.3, 31.7, 31.2, 30.5, 28.7, 27.1, 26.8, 26.5, 24.8. Peaks reported as mixture of diastereomers.

HRMS (ESI): m/z, $[M+H]^+$ calc. for $C_{22}H_{37}N_2O_4$: 393.2748; found: 393.2741.

29. 14-methyl-1,4,18,22,27-pentaoxa-9,14-diazapentaspiro[4.2.1.2.1.5^15^.3^13^.2^10^.3^8^.2^5^]triacontane

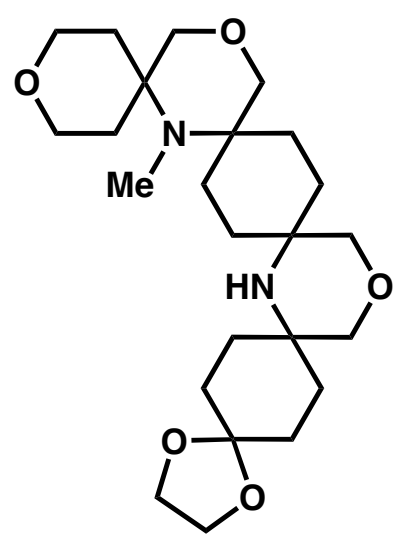

N-methyl morpholine ketone **7** (1.3 g, 5.0 mmol, 1.0 equiv) and SnAP reagent **mB** (2.5 g, 5.0 mmol, 1.0 equiv) were allowed to react according to **GP1.2** to afford the morpholine ketal **29** as a mixture of 2 diastereomers (1.4 g, 3.2 mmol, 66% yield, 1.5:1 d.r. by ^{1}H NMR [CH_2 at 3.51 and 3.24 ppm]) as off-white solid. Assignment to diastereomers could not be established from the mixture.

^{1}H NMR (400 MHz, $CDCl_3$) δ 3.98 – 3.89 (m, 12.1H), 3.72 (d, *J* = 4.5 Hz, 5.4H), 3.64 (s, 3.1H), 3.58 (s, 2.1H), 3.51 (s, 3.1H), 3.47 – 3.35 (m, 11.4H), 3.24 (s, 2.0H), 2.38 (s, 2.9H), 2.27 (s, 4.6H), 2.19 – 2.00 (m, 7.9H), 1.94 – 1.23 (m, 48.0H), 1.17 (td, *J* = 13.9, 4.0 Hz, 1.8H). Peaks reported as mixture of diastereomers (overlapped: total integral).

^{13}C NMR (101 MHz, $CDCl_3$) δ 108.9, 79.4, 77.2, 77.0, 74.0, 74.0, 73.9, 73.5, 64.9, 64.4, 64.4, 64.3, 55.2, 55.2, 55.2, 55.1, 53.2, 53.1, 53.1, 53.1, 49.8, 49.6, 49.1, 48.7, 34.2, 34.0, 33.5,

33.0, 31.2, 30.8, 30.5, 27.3, 26.8, 26.7, 25.3, 25.3, 25.3, 25.2. Peaks reported as mixture of diastereomers.

HRMS (ESI): m/z, $[M+H]^+$ calc. for $C_{24}H_{41}N_2O_5$: 437.3010; found: 437.3016.

30. 7-methyl-3,20,25-trioxa-7,12-diazatetraspiro[5.1.2.1.5^{13}.3^{11}.2^{8}.3^{6}]hexacosan-16-one

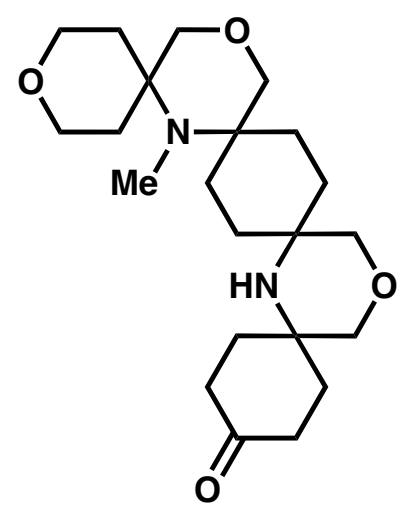

Pentacyclic ketal (mixture of 2 diastereomers) **29** (1.4 g, 3.2 mmol, 1.0 equiv) was allowed to react with HCl (3.0 M, 66 mL, 0.20 mol, 60 equiv) according to **GP3** to afford the pentacyclic morpholine ketone **30** (1.1 g, 2.8 mmol, 88% yield, 1.7:1 d.r. by ^{1}H NMR [CH_2 at 3.65 and 3.30 ppm]) as white solid. Assignment to diastereomers could not be established from the mixture.

^{1}H NMR (400 MHz, $CDCl_3$) δ 3.86 – 3.78 (m, 5.6H), 3.72 (s, 5.1H), 3.65 (s, 3.4H), 3.57 (d, *J* = 3.1 Hz, 5.1H), 3.46 – 3.36 (m, 11.1H), 3.30 (s, 2.0H), 2.74 – 2.57 (m, 5.7H), 2.33 (s, 3.0H), 2.28 (s, 5.6H), 2.26 (s, 19.6H), 1.84 – 1.59 (m, 16.1H), 1.46 – 1.17 (m, 18.8H). Peaks reported as mixture of diastereomers (overlapped: total integral).

^{13}C NMR (101 MHz, $CDCl_3$) δ 211.72, 211.64, 79.46, 74.23, 74.09, 73.90, 73.51, 64.88, 55.11, 53.11, 49.77, 49.36, 49.02, 48.77, 36.89, 36.73, 36.68, 35.80, 35.69, 34.36, 32.99, 32.90, 31.17, 27.08, 26.70, 26.56, 25.40. Peaks reported as mixture of diastereomers.

HRMS (ESI): m/z, $[M+H]^+$ calc. for $C_{22}H_{37}N_2O_4$: 393.2748; found: 393.2751.

31. (4a'''R,8a'''R)-4'-methyldecahydro-2'''H-tetraspiro[pyran-4,3'-morpholine-5',1''-cyclohexane-4'',3'''-benzo[b][1,4]oxazine-7''',2''''-[1,3]dioxolane]

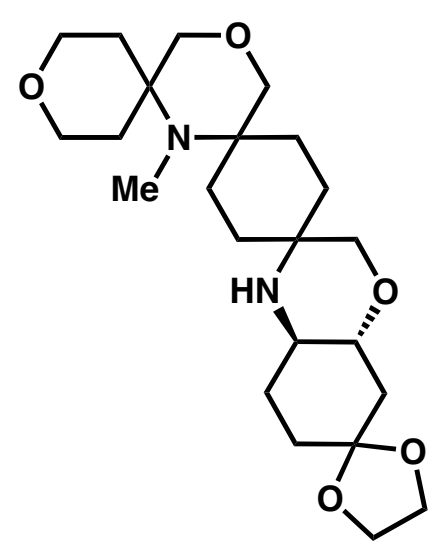

N-methyl morpholine ketone **7** (1.1 g, 4.5 mmol, 1.0 equiv) and SnAP reagent **mD** (2.1 g, 4.5 mmol, 1.0 equiv) were allowed to react according to **GP1.1** to afford the morpholine ketal **31** as a mixture of 2 diastereomers (1.2 g, 2.9 mmol, 65% yield, 1.5:1 d.r. by ^{1}H NMR [CH at 2.66 and 2.49 ppm]) as off-white solid. Assignment to diastereomers could not be established from the mixture.

^{1}H NMR (400 MHz, $CDCl_3$) δ 4.12 (d, *J* = 11.4 Hz, 0.8H), 3.92 (tt, *J* = 5.3, 2.9 Hz, 10.3H), 3.84 – 3.53 (m, 11.3H), 3.49 – 3.35 (m, 5.0H), 3.20 (d, *J* = 11.1 Hz, 1.2H), 3.15 – 2.96 (m, 2.6H), 2.72 – 2.61 (m, 1.0H), 2.56 – 2.45 (m, 1.5H), 2.41 (dt, *J* = 14.1, 3.3 Hz, 0.9H), 2.35 (s,

3.3H), 2.31 – 2.16 (m, 3.8H), 2.16 – 0.96 (m, 42.2H). Peaks reported as mixture of diastereomers (overlapped: total integral).

13C NMR (101 MHz, CDCl3) δ 109.3, 109.1, 79.7, 79.6, 78.2, 74.1, 74.0, 73.5, 73.5, 72.3, 64.9, 64.9, 64.6, 64.5, 64.5, 64.5, 55.7, 53.7, 53.1, 51.1, 50.0, 39.5, 34.1, 33.9, 33.9, 32.0, 31.3, 31.2, 31.1, 31.0, 28.3, 27.8, 27.5, 27.1, 26.8, 26.7, 26.2, 25.2, 24.4. Peaks reported as mixture of diastereomers.

HRMS (ESI): m/z, $[M+H]^+$ calc. for $C_{23}H_{39}N_2O_5$: 423.2853; found: 423.2847.

32. (4aR,8aR)-4''-methyldecahydro-2H,7H-trispiro[benzo[b][1,4]oxazine-3,1'-cyclohexane-4',3''-morpholine-5'',4'''-pyran]-7-one

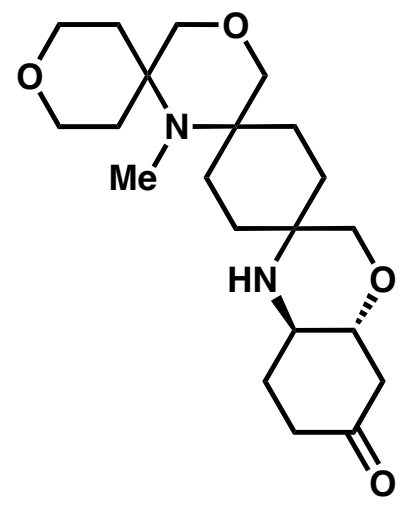

Pentacyclic ketal (mixture of 2 diastereomers) **31** (1.2 g, 2.8 mmol, 1.0 equiv) was allowed to react with HCl (3.0 M, 55 mL, 0.16 mol, 60 equiv) according to **GP3** to afford the pentacyclic morpholine ketone **32** (0.67 g, 1.8 mmol, 65% yield, 1.5:1 d.r. by ^{1}H NMR [CH at 2.87 and 4.13 ppm]) as off-white solid. Assignment to diastereomers could not be established from the mixture.

^{1}H NMR (400 MHz, $CDCl_3$) δ 4.13 (d, J = 11.4 Hz, 1.0H), 3.86 – 3.51 (m, 14.7H), 3.50 – 3.35 (m, 6.3H), 3.22 (d, J = 11.2 Hz, 1.7H), 3.16 – 2.98 (m, 4.2H), 2.87 (ddd, J = 11.5, 9.2, 3.7 Hz, 1.5H), 2.71 – 2.59 (m, 2.5H), 2.56 – 2.24 (m, 17.1H), 2.16 – 1.78 (m, 11.8H), 1.76 – 1.63 (m, 1.5H), 1.55 – 1.07 (m, 21.8H). Peaks reported as mixture of diastereomers (overlapped: total integral).

^{13}C NMR (101 MHz, $CDCl_3$) δ 207.7, 207.5, 79.5, 79.4, 77.8, 74.0, 73.9, 73.5, 72.0, 64.9, 64.9, 64.9, 55.7, 55.6, 53.0, 52.5, 50.9, 49.9, 46.2, 46.1, 40.0, 39.9, 34.0, 31.8, 31.3, 31.2, 31.2, 31.1, 28.6, 27.9, 27.8, 27.1, 26.8, 26.7, 26.6, 26.4, 25.2, 24.3. Peaks reported as mixture of diastereomers.

HRMS (ESI): m/z, $[M+H]^+$ calc. for $C_{21}H_{35}N_2O_4$: 379.2591; found: 379.2583.

33. (4a'''S,8a'''S)-4'-methyldecahydro-2'''H-tetraspiro[pyran-4,3'-morpholine-5',1''-cyclohexane-4'',3'''-benzo[b][1,4]oxazine-7''',2''''-[1,3]dioxolane]

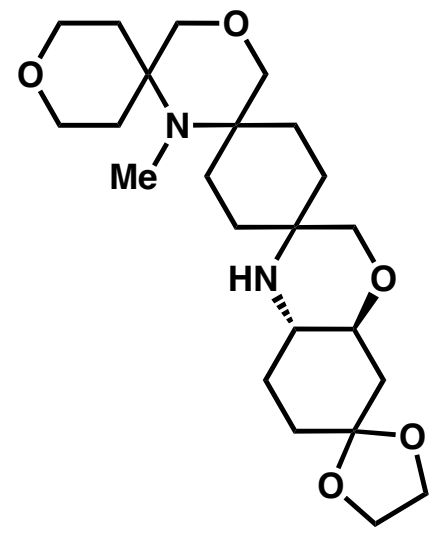

N-methyl morpholine ketone **7** (1.3 g, 5.0 mmol, 1.0 equiv) and SnAP reagent **md** (2.4 g, 5.0 mmol, 1.0 equiv) were allowed to react according to **GP1.1** to afford the morpholine ketal **33** as a mixture of 2 diastereomers (1.5 g, 3.5 mmol, 69% yield, 1.5:1 d.r. by ^{1}H NMR [CH at 2.67 and 2.49 ppm]) as off-white solid. Assignment to diastereomers could not be established from the mixture.

^{1}H NMR (400 MHz, $CDCl_3$) δ 4.12 (d, *J* = 11.4 Hz, 0.9H), 4.00 – 3.85 (m, 10.7H), 3.86 – 3.76 (m, 4.9H), 3.76 – 3.64 (m, 5.5H), 3.60 (s, 2.5H), 3.47 – 3.36 (m, 5.5H), 3.20 (d, *J* = 11.1 Hz, 1.3H), 3.15 – 2.97 (m, 2.8H), 2.66 (ddd, *J* = 12.8, 9.2, 3.6 Hz, 1.0H), 2.49 (ddd, *J* = 13.1, 9.2, 3.6 Hz, 1.5H), 2.45 – 2.37 (m, 1.0H), 2.35 (s, 3.6H), 2.27 (s, 3.9H), 2.18 – 1.04 (m, 44.6H). Peaks reported as mixture of diastereomers (overlapped: total integral).

^{13}C NMR (101 MHz, $CDCl_3$) δ 109.3, 109.1, 79.7, 79.6, 78.2, 74.1, 74.0, 73.5, 73.5, 72.3, 64.9, 64.9, 64.6, 64.5, 64.5, 64.5, 55.7, 53.7, 53.1, 51.2, 51.1, 50.0, 39.5, 34.1, 33.9, 33.8, 32.0, 31.3, 31.2, 31.1, 31.0, 28.2, 27.8, 27.5, 27.5, 27.1, 26.8, 26.7, 26.2, 25.2, 24.4. Peaks reported as mixture of diastereomers.

HRMS (ESI): m/z, $[M+H]^+$ calc. for $C_{23}H_{39}N_2O_5$: 423.2853; found: 423.2842.

34. (4aS,8aS)-4''-methyldecahydro-2H,7H-trispiro[benzo[b][1,4]oxazine-3,1'-cyclohexane-4',3''-morpholine-5'',4'''-pyran]-7-one

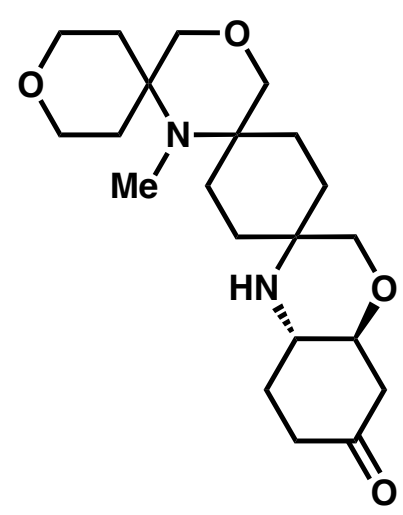

Pentacyclic ketal (mixture of 2 diastereomers) **33** (1.5 g, 3.5 mmol, 1.0 equiv) was allowed to react with HCl (3.0 M, 69 mL, 0.21 mol, 60 equiv) according to **GP3** to afford the pentacyclic morpholine ketone **34** (0.98 g, 2.6 mmol, 75% yield, 1.4:1 d.r. by ^{1}H NMR [CH at 2.88 and 4.13 ppm]) as off-white solid. Assignment to diastereomers could not be established from the mixture.

^{1}H NMR (400 MHz, $CDCl_3$) δ 4.13 (d, *J* = 11.4 Hz, 1.0H), 3.86 – 3.57 (m, 17.6H), 3.49 – 3.37 (m, 7H), 3.22 (d, *J* = 11.2 Hz, 2H), 3.16 – 3.00 (m, 4H), 2.88 (ddd, *J* = 11.5, 9.2, 3.7 Hz, 1H), 2.72 – 2.60 (m, 3H), 2.54 – 2.25 (m, 19H), 2.16 – 1.67 (m, 14H), 1.59 – 1.07 (m, 29H). Peaks reported as mixture of diastereomers (overlapped: total integral).

^{13}C NMR (101 MHz, $CDCl_3$) δ 207.8, 207.6, 79.5, 79.4, 77.8, 74.0, 73.9, 73.5, 73.5, 72.0, 64.9, 64.9, 64.9, 55.8, 55.6, 53.1, 52.5, 50.9, 49.9, 46.2, 46.1, 40.0, 39.9, 34.0, 31.8, 31.3, 31.2, 31.0, 28.6, 27.9, 27.8, 27.1, 26.8, 26.7, 26.6, 26.4, 25.2, 24.3. Peaks reported as mixture of diastereomers.

HRMS (ESI): m/z, $[M+H]^+$ calc. for $C_{21}H_{35}N_2O_4$: 379.2591; found: 379.2591.

35. (4a'''S,8a'''S)-4'-methyloctahydro-2'''H,4'''H,5'''H-tetraspiro[pyran-4,3'-morpholine-5',1''-cyclohexane-4'',3'''-benzo[b][1,4]oxazine-6''',2''''-[1,3]dioxolane]

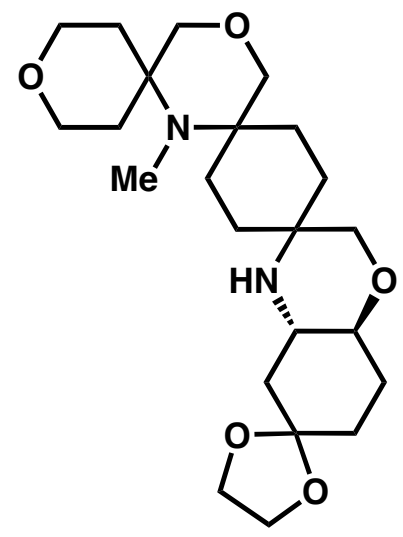

N-methyl morpholine ketone **7** (1.3 g, 5.0 mmol, 1.0 equiv) and SnAP reagent **mE** (2.4 g, 5.0 mmol, 1.0 equiv) were allowed to react according to **GP1.1** to afford the morpholine ketal **35** as a mixture of 2 diastereomers (1.6 g, 3.8 mmol, 76% yield, 1.2:1 d.r. by ^{1}H NMR [CH at 4.14 and 2.74 ppm]) as off-white solid. Assignment to diastereomers could not be established from the mixture.

^{1}H NMR (400 MHz, $CDCl_3$) δ 4.14 (d, *J* = 11.4 Hz, 1.0H), 4.03 – 3.89 (m, 9.7H), 3.86 – 3.77 (m, 4.6H), 3.77 – 3.57 (m, 8.9H), 3.48 – 3.36 (m, 5.5H), 3.22 (d, *J* = 11.1 Hz, 1.2H), 3.14 (d, *J* = 11.4 Hz, 0.9H), 2.99 – 2.80 (m, 3.2H), 2.74 (ddd, *J* = 12.6, 9.2, 3.8 Hz, 1.2H), 2.44 (d, *J* = 13.6 Hz, 1.0H), 2.33 (s, 3.4H), 2.27 (s, 4.5H), 2.13 – 1.05 (m, 43.5H). Peaks reported as mixture of diastereomers (overlapped: total integral).

^{13}C NMR (101 MHz, $CDCl_3$) δ 108.7, 108.5, 81.8, 81.6, 78.5, 74.1, 74.0, 73.5, 72.5, 64.9, 64.9, 64.9, 64.9, 64.7, 64.6, 64.5, 64.5, 55.9, 55.6, 53.2, 53.1, 51.3, 50.9, 49.8, 40.5, 40.0, 33.9, 33.5, 33.3, 32.0, 31.3, 31.1, 31.1, 28.3, 27.2, 27.2, 27.1, 26.8, 26.7, 26.7, 26.2, 25.3, 24.4. Peaks reported as mixture of diastereomers.

HRMS (ESI): m/z, $[M+H]^+$ calc. for $C_{23}H_{39}N_2O_5$: 423.2853; found: 423.2849.

36. (4aS,8aS)-4''-methyloctahydro-2H,4H-trispiro[benzo[b][1,4]oxazine-3,1'-cyclohexane-4',3''-morpholine-5'',4'''-pyran]-6(5H)-one

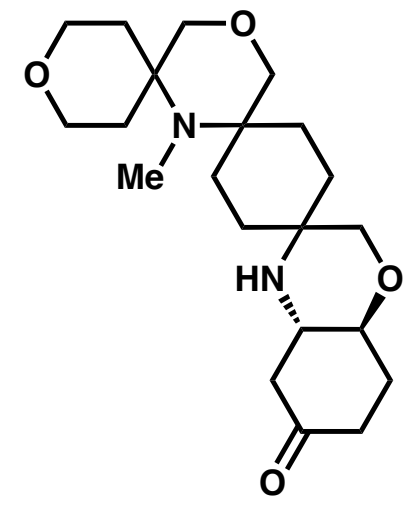

Pentacyclic ketal (mixture of 2 diastereomers) **35** (1.5 g, 3.5 mmol, 1.0 equiv) was allowed to react with HCl (3.0 M, 69 mL, 0.21 mol, 60 equiv) according to **GP3** to afford the pentacyclic morpholine ketone **36** (1.2 g, 3.2 mmol, 91% yield, 1.2:1 d.r. by ^{1}H NMR [CH at 2.83 and 3.01 ppm]) as white solid. Assignment to diastereomers could not be established from the mixture.

^{1}H NMR (400 MHz, $CDCl_3$) δ 4.16 (d, *J* = 11.4 Hz, 1.0H), 3.85 – 3.77 (m, 5.1H), 3.77 – 3.55 (m, 8.9H), 3.49 (d, *J* = 11.1 Hz, 1.4H), 3.46 – 3.37 (m, 4.6H), 3.35 – 3.18 (m, 4.5H), 3.01 (ddd, *J* = 12.8, 9.2, 4.7 Hz, 1.0H), 2.83 (ddd, *J* = 13.3, 9.2, 4.4 Hz, 1.2H), 2.51 – 2.15 (m, 18.9H), 2.16 – 2.01 (m, 6.8H), 1.96 (td, *J* = 13.6, 5.1 Hz, 1.3H), 1.85 (tt, *J* = 14.1, 4.1 Hz, 2.5H), 1.78 – 1.61 (m, 4.1H), 1.61 – 1.03 (m, 21H). Peaks reported as mixture of diastereomers (overlapped: total integral).

^{13}C NMR (101 MHz, $CDCl_3$) δ 207.8, 207.6, 80.2, 80.1, 78.2, 74.0, 73.9, 73.5, 72.3, 64.9, 64.9, 55.6, 53.1, 52.7, 52.4, 50.7, 49.8, 46.2, 46.1, 39.3, 39.3, 33.9, 31.8, 31.4, 31.2, 31.1, 31.0, 28.6, 28.1, 28.0, 27.0, 26.9, 26.7, 26.5, 26.3, 25.2, 24.2. Peaks reported as mixture of diastereomers.

HRMS (ESI): m/z, $[M+H]^+$ calc. for $C_{21}H_{35}N_2O_4$: 379.2591; found: 379.2587.

37. (4a'R,8a'R)-4'-methyldecahydro-2'H-tetraspiro[pyran-4,3'-benzo[b][1,4]oxazine-7',5''-morpholine-2'',1'''-cyclohexane-4''',2''''-[1,3]dioxolane]

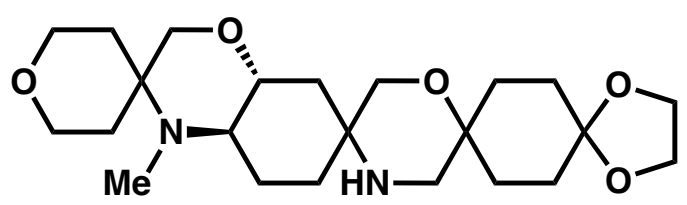

N-methyl morpholine ketone **10** (1.2 g, 5.0 mmol, 1.0 equiv) and SnAP reagent **mA** (2.4 g, 5.0 mmol, 1.0 equiv) were allowed to react according to **GP1.1** to afford the morpholine ketal **37** as a mixture of 2 diastereomers (1.5 g, 3.6 mmol, 72% yield, 1.5:1 d.r. by ^{1}H NMR [CH_2 at 2.72 and 2.64 ppm, partial overlap]) as off-white solid. Assignment to diastereomers could not be established from the mixture.

^{1}H NMR (400 MHz, $CDCl_3$) δ 4.35 – 4.25 (m, 1.2H), 3.99 – 3.87 (m, 6.5H), 3.86 – 3.77 (m, 1.3H), 3.60 – 3.36 (m, 4.6H), 3.36 – 3.25 (m, 2.2H), 3.20 (td, *J* = 10.9, 3.8 Hz, 0.8H), 2.77 – 2.68 (m, 1.5H), 2.68 – 2.58 (m, 1.0H), 2.38 – 2.22 (m, 5.7H), 2.22 – 1.03 (m, 25.7H). Peaks reported as mixture of diastereomers (overlapped: total integral).

^{13}C NMR (101 MHz, $CDCl_3$) δ 109.2, 109.2, 76.4, 71.6, 70.6, 69.9, 69.8, 66.1, 64.9, 64.5, 64.5, 64.5, 64.3, 64.3, 61.9, 61.8, 54.0, 51.4, 51.2, 49.4, 48.9, 38.7, 36.9, 33.1, 32.5, 32.4, 31.8,

31.6, 31.0, 30.0, 30.0, 29.9, 29.6, 25.2, 23.8, 22.8, 22.6. Peaks reported as mixture of diastereomers.

HRMS (ESI): m/z, $[M+H]^+$ calc. for $C_{23}H_{39}N_2O_5$: 423.2853; found: 423.2849.

38. (4a''R,8a''R)-4''-methyldecahydro-2''H-trispiro[cyclohexane-1,2'-morpholine-5',7''-benzo[b][1,4]oxazine-3'',4'''-pyran]-4-one

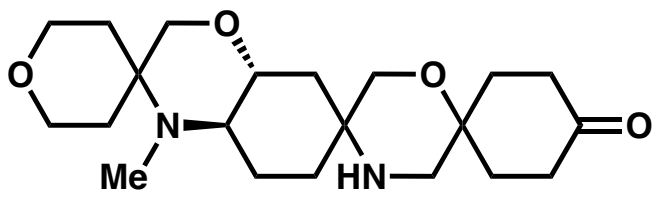

Pentacyclic ketal (mixture of 2 diastereomers) **37** (1.7 g, 3.7 mmol, 1.0 equiv) was allowed to react with HCl (3.0 M, 74 mL, 0.22 mol, 60 equiv) according to **GP3** to afford the pentacyclic morpholine ketone **38** (1.1 g, 2.9 mmol, 78% yield, 1.7 d.r. by ^{1}H NMR [CH_2 at 2.74 and 2.67 ppm, partial overlap]) as white solid. Assignment to diastereomers could not be established from the mixture.

^{1}H NMR (400 MHz, $CDCl_3$) δ 4.29 (dd, *J* = 11.6, 6.2 Hz, 2.0H), 3.89 (dd, *J* = 12.0, 5.4 Hz, 2.1H), 3.82 – 3.74 (m, 2.1H), 3.61 – 3.23 (m, 11.4H), 3.19 (ddd, *J* = 12.7, 9.2, 4.0 Hz, 1.2H), 2.80 – 2.61 (m, 4.2H), 2.60 – 2.46 (m, 4.2H), 2.39 – 2.03 (m, 21.6H), 1.96 – 1.79 (m, 5.2H), 1.78 – 1.67 (m, 2.2H), 1.62 – 1.46 (m, 4.5H), 1.43 – 1.03 (m, 9.9H). Peaks reported as mixture of diastereomers (overlapped: total integral).

^{13}C NMR (101 MHz, $CDCl_3$) δ 211.8, 211.7, 77.2, 76.4, 71.5, 71.5, 70.9, 69.5, 69.4, 66.6, 64.8, 64.4, 64.4, 61.8, 61.7, 51.2, 51.1, 49.0, 48.4, 38.7, 36.6, 36.3, 36.3, 36.3, 33.2, 32.5, 32.3, 32.0, 31.7, 31.6, 31.6, 31.5, 30.9, 25.1, 23.7, 22.7, 22.5. Peaks reported as mixture of diastereomers.

HRMS (ESI): m/z, $[M+H]^+$ calc. for $C_{21}H_{35}N_2O_4$: 379.2591; found: 379.2585.

39. (4a'R,8a'R)-4'-methyldecahydro-2'H-tetraspiro[pyran-4,3'-benzo[b][1,4]oxazine-7',3''-morpholine-5'',1'''-cyclohexane-4''',2''''-[1,3]dioxolane]

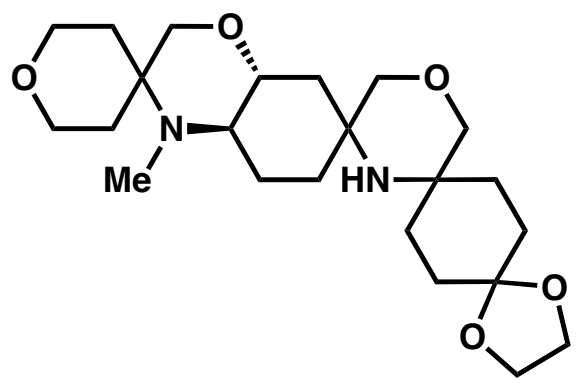

N-methyl morpholine ketone **10** (1.4 g, 6.0 mmol, 1.0 equiv) and SnAP reagent **mB** (2.9 g, 6.0 mmol, 1.0 equiv) were allowed to react according to **GP1.2** to afford the morpholine ketal **39** as a mixture of 2 diastereomers (1.7 g, 4.0 mmol, 66% yield, 5:1 d.r. by ^{13}C NMR [C(O)O at 108.9 and 109.0 ppm, to be interpreted with caution as relaxation rate might differ]) as off-white solid. Assignment to diastereomers could not be established from the mixture.

^{1}H NMR (400 MHz, $CDCl_3$) δ 4.32 (dd, *J* = 11.6, 3.7 Hz, 1.0H), 3.99 – 3.86 (m, 5.4H), 3.81 (dd, *J* = 11.9, 5.3 Hz, 1.0H), 3.18 (d, *J* = 10.0 Hz, 7.8H), 2.36 – 2.11 (m, 5.7H), 2.01 – 1.45 (m, 13.3H), 1.41 – 0.80 (m, 5.4H). Peaks reported as mixture of diastereomers (overlapped: total integral). Calibrated integral with CH at 4.32 ppm.

^{13}C NMR (101 MHz, $CDCl_3$) δ 109.0, 108.9, 79.1, 77.0, 74.9, 71.6, 64.9, 64.5, 64.5, 64.4, 64.4, 64.3, 64.3, 62.1, 61.3, 53.9, 51.6, 51.2, 49.7, 49.2, 41.7, 41.2, 36.0, 35.2, 34.4, 34.2, 34.1, 33.9, 32.6, 32.4, 31.7, 31.6, 30.5, 30.5, 30.3, 25.4, 24.3, 22.5. Peaks reported as mixture of diastereomers.

HRMS (ESI): m/z, $[M+H]^+$ calc. for $C_{23}H_{39}N_2O_5$: 423.2853; found: 423.2847.

40. (4a''R,8a''R)-4''-methyldecahydro-2''H-trispiro[cyclohexane-1,3'-morpholine-5',7''-benzo[b][1,4]oxazine-3'',4'''-pyran]-4-one

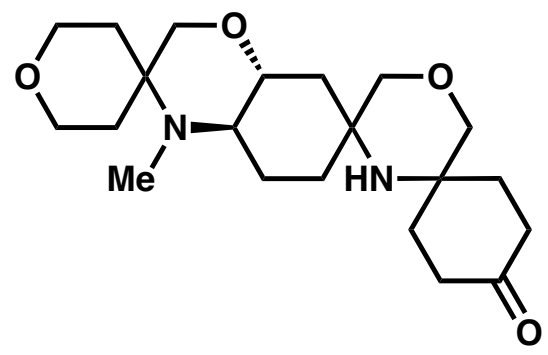

Pentacyclic ketal (mixture of 2 diastereomers) **39** (1.7 g, 3.9 mmol, 1.0 equiv) was allowed to react with HCl (3.0 M, 78 mL, 0.23 mol, 60 equiv) according to **GP3** to afford the pentacyclic morpholine ketone **40** (1.3 g, 3.4 mmol, 86% yield, 5.7:1 d.r. by ^{13}C NMR [C=O at 211.5 and 111.3 ppm, to be interpreted with caution as relaxation rate might differ]) as white solid. Assignment to diastereomers could not be established from the mixture.

^{1}H NMR (400 MHz, $CDCl_3$) δ 4.34 – 4.26 (m, 1.0H), 3.89 (dd, *J* = 12.1, 5.3 Hz, 1.1H), 3.78 (dd, *J* = 11.9, 5.3 Hz, 1.1H), 3.69 – 3.10 (m, 8.4H), 2.70 – 2.54 (m, 2.1H), 2.38 – 2.07 (m, 8.2H), 2.04 – 1.79 (m, 5.3H), 1.79 – 1.52 (m, 3.2H), 1.48 – 0.99 (m, 4.0H), 0.85 (d, *J* = 24.2 Hz, 0.7H). Peaks reported as mixture of diastereomers (overlapped: total integral).

^{13}C NMR (101 MHz, $CDCl_3$) δ 211.5, 211.3, 79.2, 77.2, 75.1, 71.6, 64.8, 64.4, 64.4, 62.0, 61.2, 53.9, 51.4, 51.2, 49.4, 49.0, 41.0, 40.9, 36.8, 36.7, 36.7, 36.6, 35.9, 35.8, 35.6, 35.5, 35.3, 35.0, 32.3, 31.6, 31.6, 25.3, 24.5, 22.4. Peaks reported as mixture of diastereomers.

HRMS (ESI): m/z, $[M+H]^+$ calc. for $C_{21}H_{35}N_2O_4$: 379.2591; found: 379.2582.

41. (4a'R,4a''R,8a'R,8a''R)-4'-methylhexadecahydro-2'H,2''H-trispiro[pyran-4,3'-benzo[b][1,4]oxazine-7',3''-benzo[b][1,4]oxazine-7'',2'''-[1,3]dioxolane]

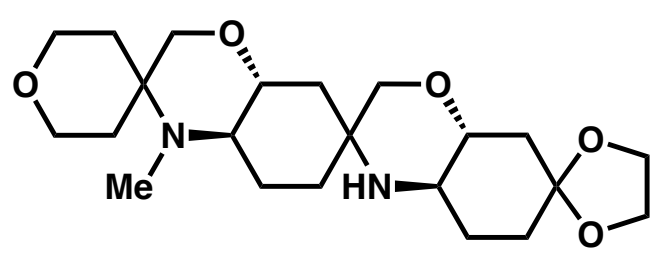

N-methyl morpholine ketone **7** (1.2 g, 5.0 mmol, 1.0 equiv) and SnAP reagent **mD** (2.4 g, 5.0 mmol, 1.0 equiv) were allowed to react according to **GP1.1** to afford the morpholine ketal **41** as a mixture of 2 diastereomers (1.3 g, 3.2 mmol, 62% yield, 2.4:1 d.r. by ^{1}H NMR [2xCH at 2.47 and CH at 2.80 ppm]) as off-white solid. Assignment to diastereomers could not be established from the mixture.

^{1}H NMR (400 MHz, $CDCl_3$) δ 4.36 – 4.26 (m, 3.5H), 4.03 – 3.84 (m, 19.7H), 3.84 – 3.76 (m, 3.7H), 3.60 – 3.20 (m, 19.3H), 3.20 – 3.00 (m, 4.6H), 2.80 (ddd, *J* = 12.4, 4.2, 2.5 Hz, 1.0H), 2.70 (ddd, *J* = 11.5, 9.3, 3.7 Hz, 1.1H), 2.53 – 2.40 (m, 4.7H), 2.37 – 2.22 (m, 14.0H), 2.15 (dddd, *J* = 14.3, 12.5, 5.6, 1.8 Hz, 4.1H), 2.00 – 1.79 (m, 11.1H), 1.79 – 0.93 (m, 45.2H). Peaks reported as mixture of diastereomers (overlapped: total integral).

^{13}C NMR (101 MHz, $CDCl_3$) δ 109.2, 109.1, 79.7, 79.5, 77.9, 77.8, 76.1, 73.6, 71.6, 71.5, 64.9, 64.8, 64.5, 64.5, 64.5, 64.5, 64.4, 61.9, 61.7, 54.0, 53.2, 53.2, 52.8, 52.6, 39.9, 39.4, 39.4, 36.8, 36.0, 33.9, 33.9, 33.8, 32.4, 32.3, 31.7, 31.7, 31.6, 29.0, 27.7, 27.6, 24.9, 23.9, 22.6, 22.5. Peaks reported as mixture of diastereomers.

HRMS (ESI): m/z, $[M+H]^+$ calc. for $C_{22}H_{37}N_2O_5$: 409.2697; found: 409.2697.

42. (4aR,4a'R,8aR,8a'R)-4'-methylhexadecahydro-2H,2'H,7H-dispiro[benzo[b][1,4]oxazine-3,7'-benzo[b][1,4]oxazine-3',4''-pyran]-7-one

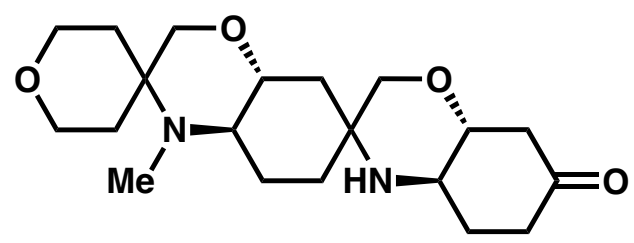

Pentacyclic ketal (mixture of 2 diastereomers) **41** (1.2 g, 2.8 mmol, 1.0 equiv) was allowed to react with HCl (3.0 M, 56 mL, 0.17 mol, 60 equiv) according to **GP3** to afford the pentacyclic morpholine ketone **42** (0.78 g, 2.1 mmol, 76% yield, 2.2:1 d.r. by ^{1}H NMR [overlapping CH of both diastereomers at 3.80 and separated CH at 3.19 ppm]) as off-white solid. Assignment to diastereomers could not be established from the mixture.

^{1}H NMR (400 MHz, $CDCl_3$) δ 4.37 – 4.28 (m, 3.1H), 4.00 (d, J = 11.5 Hz, 1.0H), 3.96 – 3.86 (m, 3.3H), 3.85 – 3.75 (m, 3.2H), 3.60 – 3.36 (m, 11.0H), 3.35 – 3.23 (m, 6.3H), 3.19 (dd, J = 11.6, 2.0 Hz, 1.0H), 3.16 – 3.03 (m, 4.3H), 2.88 (ddd, J = 11.4, 9.1, 3.7 Hz, 3.1H), 2.72 – 2.59 (m, 3.4H), 2.54 – 2.24 (m, 24.9H), 2.21 – 2.10 (m, 3.3H), 1.97 – 1.68 (m, 13.2H), 1.63 – 1.48 (m, 3.5H), 1.48 – 1.04 (m, 19.2H). Peaks reported as mixture of diastereomers (overlapped: total integral).

^{13}C NMR (101 MHz, $CDCl_3$) δ 207.6, 207.4, 79.5, 79.3, 77.8, 76.1, 73.2, 71.7, 71.6, 64.9, 64.8, 64.5, 64.4, 61.8, 61.7, 54.1, 53.9, 53.3, 52.7, 52.6, 46.1, 46.0, 39.9, 39.7, 39.7, 37.0, 36.0, 32.5, 32.4, 31.7, 31.7, 28.8, 27.8, 27.7, 24.9, 23.9, 22.6, 22.5. Peaks reported as mixture of diastereomers.

HRMS (ESI): m/z, $[M+H]^+$ calc. for $C_{20}H_{33}N_2O_4$: 365.2435; found: 365.2432.

43. (4a'R,4a''S,8a'R,8a''S)-4'-methylhexadecahydro-2'H,2''H-trispiro[pyran-4,3'-benzo[b][1,4]oxazine-7',3''-benzo[b][1,4]oxazine-7'',2'''-[1,3]dioxolane]

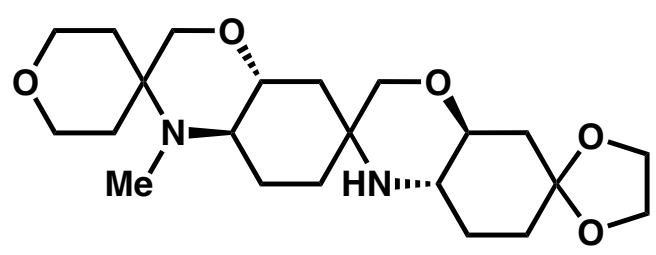

N-methyl morpholine ketone **10** (1.2 g, 5.0 mmol, 1.0 equiv) and SnAP reagent **md** (2.4 g, 5.0 mmol, 1.0 equiv) were allowed to react according to **GP1.1** to afford the morpholine ketal **43** as a mixture of 2 diastereomers (1.5 g, 3.7 mmol, 72% yield, 1:1 d.r. by ^{13}C NMR [C(O)O at 109.1 and 109.0 ppm, to be interpreted with caution as relaxation rate might differ]) as off-white solid. Assignment to diastereomers could not be established from the mixture.

^{1}H NMR (400 MHz, $CDCl_3$) δ 4.29 (dd, J = 13.2, 11.6 Hz, 1.0H), 4.01 – 3.83 (m, 5.9H), 3.82 – 3.74 (m, 1.0H), 3.58 – 3.47 (m, 1.1H), 3.47 – 3.18 (m, 4.2H), 3.16 – 2.96 (m, 1.5H), 2.72 – 2.53 (m, 2.0H), 2.36 – 2.21 (m, 4.1H), 2.14 (dddd, J = 13.8, 12.6, 5.6, 2.9 Hz, 1.2H),

1.99 – 1.78 (m, 3.0H), 1.78 – 1.00 (m, 13H). Peaks reported as mixture of diastereomers (overlapped: total integral). Calibrated integral with CH at 4.29 ppm.

^{13}C NMR (101 MHz, $CDCl_3$) δ 109.2, 109.0, 79.5, 79.4, 77.9, 77.0, 76.6, 72.8, 71.5, 71.4, 64.9, 64.8, 64.5, 64.5, 64.5, 64.4, 64.4, 61.7, 61.7, 54.0, 53.9, 53.7, 53.1, 52.8, 52.5, 42.0, 39.4, 39.3, 34.7, 34.0, 33.8, 33.8, 32.4, 32.3, 31.7, 31.6, 31.0, 27.6, 27.5, 25.5, 23.5, 22.5, 22.4. Peaks reported as mixture of diastereomers.

HRMS (ESI): m/z, $[M+H]^+$ calc. for $C_{22}H_{37}N_2O_5$: 409.2697; found: 409.2695.

44. (4aS,4a'R,8aS,8a'R)-4'-methylhexadecahydro-2H,2'H,7H-dispiro[benzo[b][1,4]oxazine-3,7'-benzo[b][1,4]oxazine-3',4''-pyran]-7-one

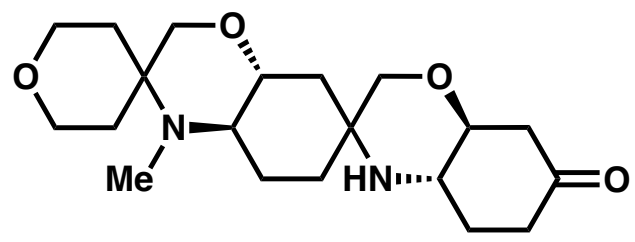

Pentacyclic ketal (mixture of 2 diastereomers) **43** (1.5 g, 3.6 mmol, 1.0 equiv) was allowed to react with HCl (3.0 M, 71 mL, 0.22 mol, 60 equiv) according to **GP3** to afford the pentacyclic morpholine ketone **44** (1.1 g, 2.9 mmol, 81% yield, 1:1 d.r. by ^{1}H NMR [CH at 3.81 and 3.91 ppm]) as off-white solid. Assignment to diastereomers could not be established from the mixture.

^{1}H NMR (400 MHz, $CDCl_3$) δ 4.39 – 4.26 (m, 2.0H), 3.99 (d, *J* = 11.4 Hz, 1.0H), 3.95 – 3.88 (m, 2.1H), 3.86 – 3.76 (m, 2.0H), 3.59 – 3.37 (m, 6.2H), 3.34 (dd, *J* = 11.6, 2.0 Hz, 1.0H), 3.30 – 3.23 (m, 3.0H), 3.19 – 2.98 (m, 5.1H), 2.73 – 2.60 (m, 4.0H), 2.48 – 2.24 (m, 14.3H), 2.16 (ddt, *J* = 13.8, 12.4, 6.1 Hz, 1.9H), 2.04 – 1.65 (m, 9.1H), 1.50 – 1.03 (m, 12.6H). Peaks reported as mixture of diastereomers (overlapped: total integral).

^{13}C NMR (101 MHz, $CDCl_3$) δ 207.7, 207.4, 79.4, 79.2, 77.5, 77.0, 76.6, 72.4, 71.6, 71.5, 64.9, 64.5, 61.7, 61.7, 54.1, 53.9, 53.1, 52.8, 52.6, 52.5, 46.1, 46.0, 42.0, 39.9, 39.8, 34.6, 33.8, 32.5, 32.4, 31.8, 31.6, 31.3, 27.8, 27.7, 25.6, 23.5, 22.5, 22.5. Peaks reported as mixture of diastereomers.

HRMS (ESI): m/z, $[M+H]^+$ calc. for $C_{20}H_{33}N_2O_4$: 365.2435; found: 365.2430.

45. (4a'R,4a''S,8a'R,8a''S)-4'-methyltetradecahydro-2'H,2''H,4''H,5''H-trispiro[pyran-4,3'-benzo[b][1,4]oxazine-7',3''-benzo[b][1,4]oxazine-6'',2'''-[1,3]dioxolane]

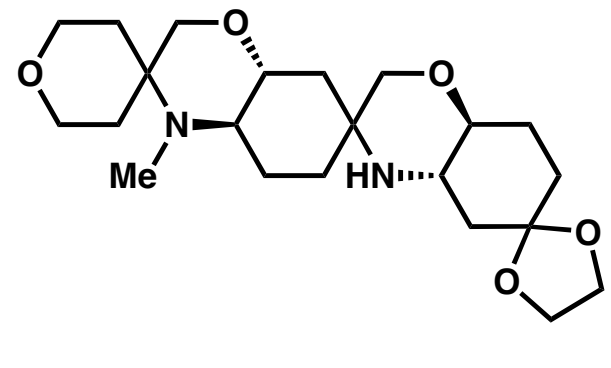

N-methyl morpholine ketone **10** (1.2 g, 5.0 mmol, 1.0 equiv) and SnAP reagent **mE** (2.4 g, 5.0 mmol, 1.0 equiv) were allowed to react according to **GP1.1** to afford the morpholine ketal **45** as a mixture of 2 diastereomers (1.5 g, 3.8 mmol, 75% yield, 1.9:1 d.r. by ^{1}H NMR [2xCH at 4.31 and CH_2 at 3.12 ppm]) as off-white solid. Assignment to diastereomers could not be established from the mixture.

^{1}H NMR (400 MHz, $CDCl_3$) δ 4.36 – 4.26 (m, 2.9H), 4.03 – 3.85 (m, 17.3H), 3.85 – 3.75 (m, 3.0H), 3.60 – 3.20 (m, 14.0H), 3.12 (ddd, *J* = 13.0, 9.4, 4.1 Hz, 2.0H), 3.00 – 2.83 (m, 5.7H), 2.68 – 2.57 (m, 2.9H), 2.37 – 2.21 (m, 11.8H), 2.15 (ddd, *J* = 13.8, 12.5, 5.6 Hz, 3.2H), 2.04 – 1.50 (m, 27.5H), 1.49 – 0.91 (m, 19.1H). Peaks reported as mixture of diastereomers (overlapped: total integral).

^{13}C NMR (101 MHz, $CDCl_3$) δ 108.6, 108.5, 81.8, 81.4, 78.3, 73.0, 71.5, 64.9, 64.9, 64.6, 64.6, 64.5, 64.5, 61.7, 53.9, 52.7, 52.3, 51.4, 50.9, 42.0, 40.2, 40.1, 34.7, 34.1, 33.4, 33.3, 32.5, 32.4, 31.8, 31.6, 31.1, 27.2, 27.1, 25.5, 23.5, 22.6, 22.4. Peaks reported as mixture of diastereomers.

HRMS (ESI): m/z, $[M+H]^+$ calc. for $C_{22}H_{37}N_2O_5$: 409.2697; found: 409.2695.

46. (4aS,4a'R,8aS,8a'R)-4'-methyltetradecahydro-2H,2'H,4H-dispiro[benzo[b][1,4]oxazine-3,7'-benzo[b][1,4]oxazine-3',4''-pyran]-6(5H)-one

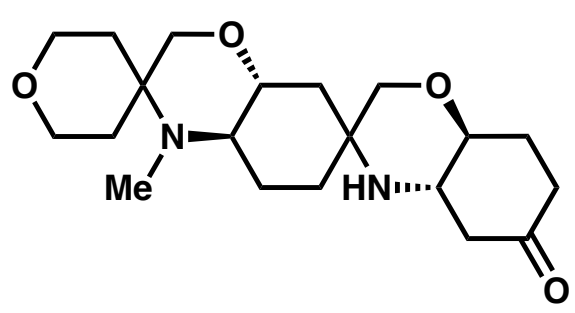

Pentacyclic ketal (mixture of 2 diastereomers) **45** (1.4 g, 3.4 mmol, 1.0 equiv) was allowed to react with HCl (3.0 M, 67 mL, 0.20 mol, 60 equiv) according to **GP3** to afford the pentacyclic morpholine ketone **46** (0.96 g, 2.6 mmol, 78% yield, 2.6:1 d.r. by ^{1}H NMR [2xCH at 4.31 and CH at 4.01 ppm]) as off-white solid. Assignment to diastereomers could not be established from the mixture.

^{1}H NMR (400 MHz, $CDCl_3$) δ 4.36 – 4.27 (m, 3.6H), 4.01 (d, *J* = 11.3 Hz, 2.6H), 3.91 (dd, *J* = 12.0, 5.4 Hz, 3.9H), 3.80 (ddd, *J* = 11.8, 5.3, 1.5 Hz, 3.6H), 3.60 – 3.21 (m, 20.7H), 3.14 (ddd, *J* = 12.9, 9.5, 4.0 Hz, 2.5H), 3.09 – 2.93 (m, 3.6H), 2.66 – 2.51 (m, 3.8H), 2.49 – 2.04 (m, 36.6H), 1.98 (dq, *J* = 13.4, 3.7 Hz, 2.6H), 1.93 – 1.82 (m, 4.8H), 1.80 – 1.62 (m, 9.8H), 1.44 – 0.96 (m, 18.9H). Peaks reported as mixture of diastereomers (overlapped: total integral).

^{13}C NMR (101 MHz, $CDCl_3$) δ 207.6, 207.6, 80.1, 79.8, 78.0, 76.6, 72.8, 71.6, 71.5, 64.9, 64.6, 64.5, 64.5, 61.7, 61.6, 53.9, 52.8, 52.6, 52.3, 52.2, 46.0, 45.9, 42.0, 39.3, 34.5, 33.8, 32.4, 32.4, 31.8, 31.6, 31.2, 28.0, 27.9, 25.6, 23.5, 22.5, 22.4. Peaks reported as mixture of diastereomers. Peaks reported as mixture of diastereomers.

HRMS (ESI): m/z, $[M+H]^+$ calc. for $C_{20}H_{33}N_2O_4$: 365.2435; found: 365.2430.

47. (4a'S,8a'S)-4'-methyldecahydro-2'H-tetraspiro[pyran-4,3'-benzo[b][1,4]oxazine-7',5''-morpholine-2'',1'''-cyclohexane-4''',2''''-[1,3]dioxolane]

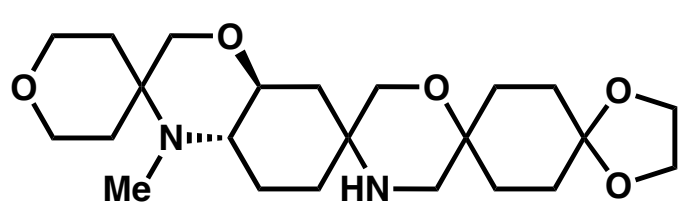

N-methyl morpholine ketone **13** (1.2 g, 5.0 mmol, 1.0 equiv) and SnAP reagent **mA** (2.5 g, 5.0 mmol, 1.0 equiv) were allowed to react according to **GP1.1** to afford the morpholine ketal **47** as a mixture of 2 diastereomers (1.6 g, 3.8 mmol, 76% yield, 1.6:1 d.r. by ^{1}H NMR [CH_2 at

2.68 and 2.61 ppm, partial overlap]) as off-white solid. Assignment to diastereomers could not be established from the mixture.

^{1}H NMR (400 MHz, $CDCl_3$) δ 4.32 – 4.23 (m, 2.6H), 3.96 – 3.84 (m, 13.6H), 3.83 – 3.74 (m, 2.6H), 3.58 – 3.21 (m, 14.3H), 3.17 (ddd, *J* = 12.1, 9.3, 4.1 Hz, 1.5H), 2.74 – 2.54 (m, 5.2H), 2.35 – 2.18 (m, 12.2H), 2.18 – 1.66 (m, 25.7H), 1.61 – 1.27 (m, 13.7H), 1.24 – 1.01 (m, 10.2H).Peaks reported as mixture of diastereomers (overlapped: total integral). Calibrated integral with overlapping CH_2 at 2.74 – 2.54 ppm.

^{13}C NMR (101 MHz, $CDCl_3$) δ 109.2, 109.1, 76.4, 71.5, 71.5, 70.5, 69.8, 69.7, 66.1, 64.9, 64.5, 64.4, 64.4, 64.3, 64.2, 64.2, 61.8, 61.7, 53.8, 51.2, 51.1, 49.4, 48.8, 38.7, 36.9, 33.1, 32.5, 32.4, 31.7, 31.6, 30.9, 30.0, 30.0, 29.9, 29.8, 29.5, 29.5, 25.1, 23.8, 22.7, 22.5. Peaks reported as mixture of diastereomers. Peaks reported as mixture of diastereomers.

HRMS (ESI): m/z, $[M+H]^+$ calc. for $C_{23}H_{39}N_2O_5$: 423.2853; found: 423.2844.

48. (4a"S,8a"S)-4"-methyldecahydro-2"H-trispiro[cyclohexane-1,2'-morpholine-5',7"-benzo[b][1,4]oxazine-3",4'''-pyran]-4-one

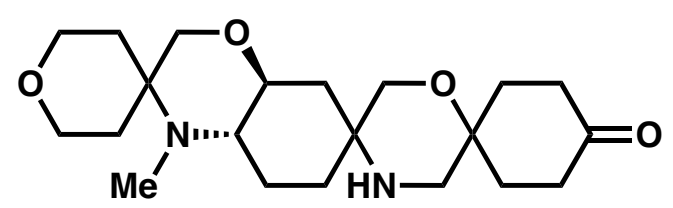

Pentacyclic ketal (mixture of 2 diastereomers) **47** (1.7 g, 3.7 mmol, 1.0 equiv) was allowed to react with HCl (3.0 M, 74 mL, 0.22 mol, 60 equiv) according to **GP3** to afford the pentacyclic morpholine ketone **48** (1.3 g, 3.3 mmol, 90% yield, 1.4:1 d.r. by ^{1}H NMR [CH_2 at 2.76 and 2.69 ppm]) as white solid. Assignment to diastereomers could not be established from the mixture.

^{1}H NMR (400 MHz, $CDCl_3$) δ 4.35 – 4.28 (m, 2.3H), 3.91 (ddd, *J* = 12.1, 5.4, 1.5 Hz, 2.4H), 3.84 – 3.76 (m, 2.4H), 3.63 – 3.34 (m, 10.4H), 3.30 (td, *J* = 11.8, 2.3 Hz, 3.0H), 3.21 (td, *J* = 11.3, 4.1 Hz, 1.4H), 2.81 – 2.63 (m, 4.8H), 2.62 – 2.48 (m, 4.7H), 2.40 – 2.05 (m, 25.2H), 1.99 – 1.81 (m, 5.8H), 1.80 – 1.69 (m, 2.5H), 1.63 – 1.49 (m, 5.2H), 1.43 – 1.31 (m, 1.9H), 1.25 – 1.04 (m, 8.6H). Peaks reported as mixture of diastereomers (overlapped: total integral). Calibrated integral with overlapping CH_2 at 2.74 – 2.54 ppm.

^{13}C NMR (101 MHz, $CDCl_3$) δ 211.9, 211.8, 77.2, 76.4, 71.6, 71.5, 71.0, 69.6, 69.5, 66.6, 64.9, 64.5, 64.5, 61.8, 61.8, 54.0, 51.3, 51.1, 49.1, 48.4, 38.7, 36.6, 36.4, 36.4, 36.3, 33.2, 32.5, 32.4, 32.1, 31.8, 31.7, 31.6, 31.5, 30.9, 25.1, 23.8, 22.7, 22.5. Peaks reported as mixture of diastereomers.

HRMS (ESI): m/z, $[M+H]^+$ calc. for $C_{21}H_{35}N_2O_4$: 379.2591; found: 379.2589.

49. (4a'S,8a'S)-4'-methyldecahydro-2'H-tetraspiro[pyran-4,3'-benzo[b][1,4]oxazine-7',3''-morpholine-5'',1'''-cyclohexane-4''',2''''-[1,3]dioxolane]

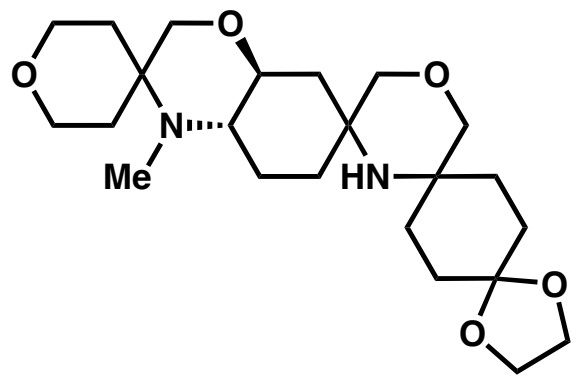

N-methyl morpholine ketone **13** (1.2 g, 5.0 mmol, 1.0 equiv) and SnAP reagent **mB** (2.5 g, 5.0 mmol, 1.0 equiv) were allowed to react according to **GP1.2** to afford the morpholine ketal **49** as a mixture of 2 diastereomers (1.6 g, 3.8 mmol, 75% yield, 2.3:1 d.r. by ^{1}H NMR [CH at 3.13 and 3.61 ppm, partial overlap]) as off-white solid. Assignment to diastereomers could not be established from the mixture.

^{1}H NMR (400 MHz, $CDCl_3$) δ 4.33 – 4.26 (m, 3.2H), 3.94 – 3.82 (m, 17.8H), 3.78 (dd, J = 11.9, 5.4 Hz, 3.4H), 3.65 – 3.56 (m, 1.0H), 3.56 – 3.48 (m, 5.8H), 3.47 – 3.19 (m, 17.7H), 3.13 (ddd, J = 13.0, 9.6, 4.1 Hz, 2.3H), 2.32 – 2.19 (m, 13.1H), 2.19 – 2.08 (m, 5.8H), 1.98 – 1.42 (m, 43.9H), 1.38 – 0.98 (m, 14.1H), 0.85 (s, 2.4H). Peaks reported as mixture of diastereomers (overlapped: total integral)

^{13}C NMR (101 MHz, $CDCl_3$) δ 108.9, 108.8, 79.1, 77.4, 77.0, 76.8, 74.9, 71.6, 71.5, 64.9, 64.9, 64.5, 64.5, 64.3, 64.3, 64.3, 64.3, 64.2, 62.1, 61.2, 53.9, 53.8, 51.6, 51.2, 49.6, 49.1, 41.6, 41.1, 36.0, 35.2, 34.3, 34.1, 34.1, 33.8, 32.4, 32.3, 31.6, 31.5, 30.5, 30.5, 25.4, 24.2, 22.5, 22.4. Peaks reported as mixture of diastereomers.

HRMS (ESI): m/z, $[M+H]^+$ calc. for $C_{23}H_{39}N_2O_5$: 423.2853; found: 423.2846.

50. (4a''S,8a''S)-4''-methyldecahydro-2''H-trispiro[cyclohexane-1,3'-morpholine-5',7''-benzo[b][1,4]oxazine-3'',4'''-pyran]-4-one

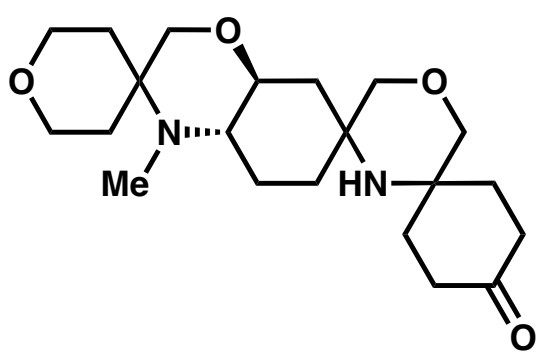

Pentacyclic ketal (mixture of 2 diastereomers) **49** (1.5 g, 3.6 mmol, 1.0 equiv) was allowed to react with HCl (3.0 M, 73 mL, 0.22 mol, 60 equiv) according to **GP3** to afford the pentacyclic morpholine ketone **50** (1.1 g, 2.8 mmol, 78% yield, 1.4:1 d.r. determined in previous step) as an off-white solid. Assignment to diastereomers could not be established from the mixture.

^{1}H NMR (400 MHz, $CDCl_3$) δ 4.36 – 4.28 (m, 1.0H), 3.91 (ddd, J = 12.2, 5.4, 2.8 Hz, 1.1H), 3.80 (dd, J = 11.9, 5.4 Hz, 1.1H), 3.64 – 3.35 (m, 6.4H), 3.33 – 3.11 (m, 1.9H), 2.70 – 2.55 (m, 2.1H), 2.36 – 2.10 (m, 8.3H), 2.05 – 1.84 (m, 5.3H), 1.80 – 1.61 (m, 3.0H), 1.49 – 1.02 (m, 3.7H), 0.85 (s, 0.35H). Peaks reported as mixture of diastereomers (overlapped: total integral). Calibrated integral with overlapping CH signal at 4.36 – 4.28 ppm.

^{13}C NMR (101 MHz, $CDCl_3$) δ 211.5, 211.3, 79.2, 77.2, 75.2, 71.6, 64.9, 64.5, 64.5, 62.1, 61.2, 53.9, 51.5, 51.2, 49.4, 49.1, 41.0, 40.9, 36.8, 36.8, 36.7, 36.7, 35.9, 35.8, 35.7, 35.5, 35.3, 35.0, 32.4, 31.7, 31.6, 25.3, 24.6, 22.4. Peaks reported as mixture of diastereomers.

HRMS (ESI): m/z, $[M+H]^+$ calc. for $C_{21}H_{35}N_2O_4$: 379.2591; found: 379.2587.

51. (4a'S,4a''R,8a'S,8a''R)-4'-methylhexadecahydro-2'H,2''H-trispiro[pyran-4,3'-benzo[b][1,4]oxazine-7',3''-benzo[b][1,4]oxazine-7'',2'''-[1,3]dioxolane]

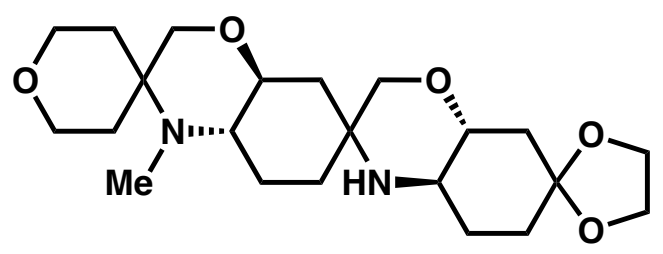

N-methyl morpholine ketone **13** (1.2 g, 5.0 mmol, 1.0 equiv) and SnAP reagent **mD** (2.4 g, 5.0 mmol, 1.0 equiv) were allowed to react according to **GP1.1** to afford the morpholine ketal **51** as a mixture of 2 diastereomers (1.5 g, 3.6 mmol, 72% yield, , 1:1 d.r. by ^{13}C NMR [C(O)O at 109.2 and 109.1 ppm, to be interpreted with caution as relaxation rate might differ]) as off-white solid. Assignment to diastereomers could not be established from the mixture.

^{1}H NMR (400 MHz, $CDCl_3$) δ 4.29 (dd, *J* = 12.9, 11.6 Hz, 2.0H), 3.98 – 3.83 (m, 11.5H), 3.82 – 3.74 (m, 1.9H), 3.52 (tdd, *J* = 12.1, 10.6, 2.7 Hz, 2.2H), 3.46 – 3.34 (m, 3.8H), 3.30 (dd, *J* = 11.6, 2.0 Hz, 1.0H), 3.26 – 3.16 (m, 2.9H), 3.16 – 2.96 (m, 2.9H), 2.71 – 2.53 (m, 4.0H), 2.23 (s, 7.8H), 2.13 (dddd, *J* = 13.7, 12.5, 5.6, 2.7 Hz, 2.1H), 1.99 – 1.77 (m, 6.1H), 1.77 – 1.47 (m, 11.8H), 1.47 – 0.99 (m, 13.6H). Peaks reported as mixture of diastereomers (overlapped: total integral)

^{13}C NMR (101 MHz, $CDCl_3$) δ 109.2, 109.1, 79.5, 79.5, 77.9, 77.0, 76.6, 72.9, 71.5, 71.4, 64.9, 64.8, 64.5, 64.5, 64.4, 64.4, 61.7, 61.7, 54.0, 53.9, 53.7, 53.1, 52.7, 52.5, 42.0, 39.4, 39.3, 34.7, 34.0, 33.8, 33.8, 32.4, 32.3, 31.7, 31.6, 31.0, 27.6, 27.5, 25.5, 23.5, 22.5, 22.4. Peaks reported as mixture of diastereomers.

HRMS (ESI): m/z, $[M+H]^+$ calc. for $C_{22}H_{37}N_2O_5$: 409.2697; found: 409.2690.

52. (4aR,4a'S,8aR,8a'S)-4'-methylhexadecahydro-2H,2'H,7H-dispiro[benzo[b][1,4]oxazine-3,7'-benzo[b][1,4]oxazine-3',4''-pyran]-7-one

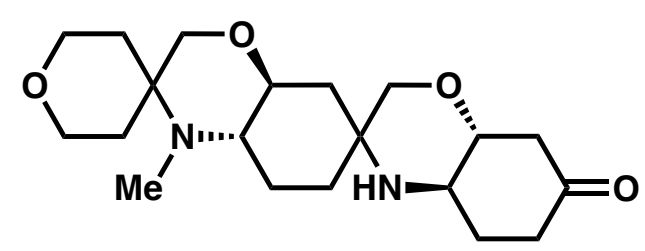

Pentacyclic ketal (mixture of 2 diastereomers) **51** (1.4 g, 3.4 mmol, 1.0 equiv) was allowed to react with HCl (3.0 M, 69 mL, 0.21 mol, 60 equiv) according to **GP3** to afford the pentacyclic morpholine ketone **52** (0.95 g, 2.6 mmol, 76% yield, 1.6:1 d.r. by ^{1}H NMR [CH at 4.30 and 3.35 ppm, partial overlap]) as an off-white solid. Assignment to diastereomers could not be established from the mixture.

^{1}H NMR (400 MHz, $CDCl_3$) δ 4.35 (d, *J* = 11.6 Hz, 1.0H), 4.30 (d, *J* = 11.7 Hz, 1.6H), 3.99 (d, *J* = 11.4 Hz, 1.7H), 3.96 – 3.86 (m, 2.8H), 3.86 – 3.75 (m, 2.7H), 3.61 – 3.21 (m, 12.9H), 3.19 – 2.97 (m, 7.0H), 2.74 – 2.60 (m, 5.2H), 2.49 – 2.23 (m, 18.6H), 2.16 (ddt, *J* = 13.8, 12.4, 6.2 Hz, 3.1H), 2.07 – 1.95 (m, 1.9H), 1.95 – 1.65 (m, 10.9H), 1.48 – 1.03 (m, 17.2H). Peaks reported as mixture of diastereomers (overlapped: total integral)

^{13}C NMR (101 MHz, $CDCl_3$) δ 207.7, 207.4, 79.4, 79.2, 77.5, 77.0, 76.6, 72.4, 71.6, 71.5, 64.9, 64.5, 61.7, 61.7, 53.9, 53.1, 52.8, 52.6, 52.5, 46.1, 46.0, 42.0, 39.9, 39.8, 34.6, 33.8, 32.5, 32.4, 31.8, 31.6, 31.3, 27.8, 27.7, 25.6, 23.5, 22.5, 22.4. Peaks reported as mixture of diastereomers.

HRMS (ESI): m/z, $[M+H]^+$ calc. for $C_{20}H_{33}N_2O_4$: 365.2435; found: 365.2430.

53. (4a'S,4a''S,8a'S,8a''S)-4'-methylhexadecahydro-2'H,2''H-trispiro[pyran-4,3'-benzo[b][1,4]oxazine-7',3''-benzo[b][1,4]oxazine-7'',2'''-[1,3]dioxolane]

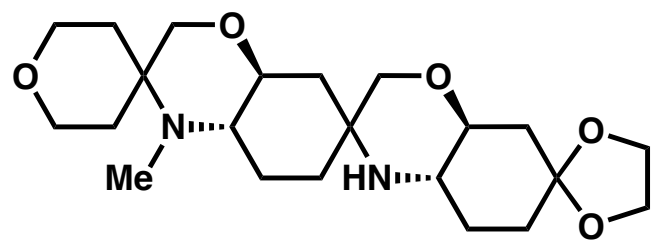

N-methyl morpholine ketone **13** (1.2 g, 5.0 mmol, 1.0 equiv) and SnAP reagent **md** (2.4 g, 5.0 mmol, 1.0 equiv) were allowed to react according to **GP1.1** to afford the morpholine ketal **53** as a mixture of 2 diastereomers (1.5 g, 3.6 mmol, 72% yield, 2.1:1 d.r. by ^{1}H NMR [CH at 4.32 ppm, overlapped and CH 2.81 ppm]) as off-white solid. Assignment to diastereomers could not be established from the mixture.

^{1}H NMR (400 MHz, $CDCl_3$) δ 4.37 – 4.28 (m, 3.1H), 4.00 (d, *J* = 11.5 Hz, 1.4H), 3.97 – 3.86 (m, 17.8H), 3.81 (dd, *J* = 11.9, 5.3 Hz, 3.5H), 3.63 – 3.23 (m, 18.0H), 3.18 (dd, *J* = 11.5, 1.9 Hz, 1.1H), 3.14 – 3.02 (m, 3.3H), 2.81 (ddd, *J* = 12.4, 2.9, 2.7 Hz, 1.0H), 2.71 (ddd, *J* = 11.9, 9.2, 3.7 Hz, 1.1H), 2.55 – 2.40 (m, 4.4H), 2.40 – 2.22 (m, 12.9H), 2.22 – 2.11 (m, 3.9H), 2.01 – 1.45 (m, 36.4H), 1.44 – 1.21 (m, 10.8H), 1.20 – 1.02 (m, 8.9H). Peaks reported as mixture of diastereomers (overlapped: total integral)

^{13}C NMR (101 MHz, $CDCl_3$) δ 109.2, 109.1, 79.7, 79.5, 77.9, 77.7, 77.4, 76.1, 73.6, 71.6, 71.5, 64.9, 64.8, 64.6, 64.6, 64.5, 64.5, 64.5, 62.0, 61.7, 54.3, 54.0, 53.2, 52.8, 52.7, 40.0, 39.5, 39.4, 36.8, 35.9, 34.0, 33.9, 32.4, 32.3, 31.7, 31.7, 29.0, 27.6, 24.9, 23.8, 22.7, 22.5. Peaks reported as mixture of diastereomers.

HRMS (ESI): m/z, $[M+H]^+$ calc. for $C_{22}H_{37}N_2O_5$: 409.2697; found: 409.2691.

54. (4aS,4a'S,8aS,8a'S)-4'-methylhexadecahydro-2H,2'H,7H-dispiro[benzo[b][1,4]oxazine-3,7'-benzo[b][1,4]oxazine-3',4''-pyran]-7-one

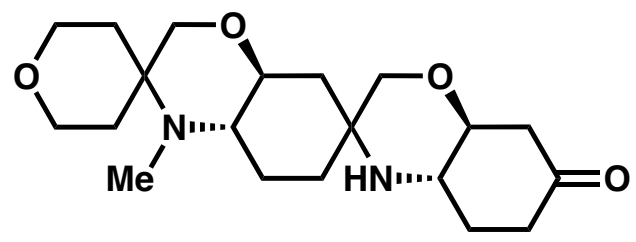

Pentacyclic ketal (mixture of 2 diastereomers) **53** (1.4 g, 3.4 mmol, 1.0 equiv) was allowed to react with HCl (3.0 M, 69 mL, 0.21 mol, 60 equiv) according to **GP3** to afford the pentacyclic morpholine ketone **54** (0.94 g, 2.6 mmol, 76% yield, 1.9:1 d.r. by ^{1}H NMR [CH at 4.33, overlapped and 3.20 ppm]) as an off-white solid. Assignment to diastereomers could not be established from the mixture.

^{1}H NMR (400 MHz, $CDCl_3$) δ 4.39 – 4.29 (m, 2.9H), 4.05 – 3.98 (m, 1.0H), 3.98 – 3.86 (m, 3.2H), 3.86 – 3.76 (m, 3.0H), 3.61 – 3.37 (m, 10.1H), 3.37 – 3.23 (m, 5.8H), 3.20 (dd, *J* = 11.6, 1.9 Hz, 1.0H), 3.17 – 3.03 (m, 3.9H), 2.89 (ddd, *J* = 11.5, 9.1, 3.7 Hz, 2.9H), 2.72 – 2.60 (m, 3.1H), 2.55 – 2.27 (m, 19.4H), 2.25 (s, 2.8H), 2.23 – 2.11 (m, 3.2H), 1.98 – 1.86 (m, 5.8H), 1.86 – 1.68 (m, 6.0H), 1.64 – 1.21 (m, 13.7H), 1.21 – 1.07 (m, 6.8H). Peaks reported as mixture of diastereomers (overlapped: total integral)

^{13}C NMR (101 MHz, $CDCl_3$) δ 207.6, 207.5, 79.5, 79.3, 77.8, 77.4, 76.1, 73.2, 71.7, 71.6, 64.9, 64.8, 64.5, 64.4, 61.8, 61.7, 54.1, 53.9, 53.3, 52.7, 52.6, 46.1, 46.0, 39.9, 39.7, 39.7, 37.1, 36.0, 32.5, 32.4, 31.7, 31.7, 28.8, 27.8, 27.7, 24.9, 23.9, 22.6, 22.5. Peaks reported as mixture of diastereomers.

HRMS (ESI): m/z, $[M+H]^+$ calc. for $C_{20}H_{33}N_2O_4$: 365.2435; found: 365.2439.

55. (4a'S,4a''S,8a'S,8a''S)-4'-methyltetradecahydro-2'H,2''H,4''H,5''H-trispiro[pyran-4,3'-benzo[b][1,4]oxazine-7',3''-benzo[b][1,4]oxazine-6'',2'''-[1,3]dioxolane]

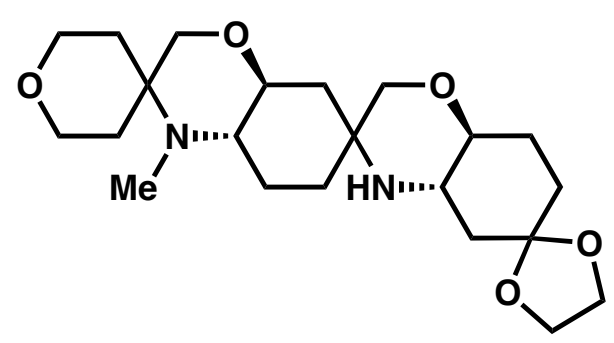

N-methyl morpholine ketone **13** (1.2 g, 5.0 mmol, 1.0 equiv) and SnAP reagent **mE** (2.4 g, 5.0 mmol, 1.0 equiv) were allowed to react according to **GP1.1** to afford the morpholine ketal **55** as a mixture of 2 diastereomers (1.4 g, 3.3 mmol, 66% yield, 1.2:1 d.r. by ^{1}H NMR [CH at 4.01 ppm, partially overlapped and 2.74 ppm]) as off-white solid. Assignment to diastereomers could not be established from the mixture.

^{1}H NMR (400 MHz, $CDCl_3$) δ 4.36 – 4.27 (m, 2.1H), 4.01 (d, *J* = 11.5 Hz, 1.0H), 3.92 (m, 10.5H), 3.85 – 3.75 (m, 2.0H), 3.61 – 3.15 (m, 11.3H), 3.05 – 2.79 (m, 3.6H), 2.74 (ddd, *J* = 12.1, 9.3, 4.0 Hz, 1.2H), 2.47 (dq, *J* = 14.4, 3.3 Hz, 1.3H), 2.38 – 2.22 (m, 8.1H), 2.15 (ddd, *J* = 14.1, 12.4, 5.5 Hz, 2.2H), 1.95 – 1.41 (m, 20.1H), 1.41 – 1.21 (m, 5.2H), 1.20 – 1.01 (m, 5.8H). Peaks reported as mixture of diastereomers (overlapped: total integral)

^{13}C NMR (101 MHz, $CDCl_3$) δ 108.6, 108.4, 81.7, 81.6, 78.1, 77.8, 76.2, 73.8, 71.6, 64.9, 64.9, 64.6, 64.5, 64.5, 64.5, 64.5, 64.5, 61.8, 61.7, 53.9, 52.5, 51.5, 50.9, 40.1, 39.9, 37.0, 36.0, 33.4, 33.4, 32.5, 32.4, 31.8, 31.6, 28.9, 27.2, 27.2, 24.9, 24.0, 22.6, 22.4. Peaks reported as mixture of diastereomers.

HRMS (ESI): m/z, $[M+H]^+$ calc. for $C_{22}H_{37}N_2O_5$: 409.2697; found: 409.2693.

56. (4aS,4a'S,8aS,8a'S)-4'-methyltetradecahydro-2H,2'H,4H-dispiro[benzo[b][1,4]oxazine-3,7'-benzo[b][1,4]oxazine-3',4''-pyran]-6(5H)-one

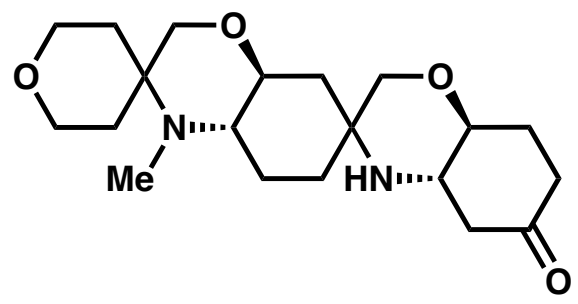

Pentacyclic ketal (mixture of 2 diastereomers) **55** (1.2 g, 2.9 mmol, 1.0 equiv) was allowed to react with HCl (3.0 M, 59 mL, 0.18 mol, 60 equiv) according to **GP3** to afford the pentacyclic morpholine ketone **56** (1.1 g, 2.9 mmol, 99% yield, 1.6:1 d.r. by ^{1}H NMR [CH at 4.32, overlapped and 4.04 ppm]) as an off-white solid. Assignment to diastereomers could not be established from the mixture.

^{1}H NMR (400 MHz, $CDCl_3$) δ 4.38 – 4.25 (m, 2.6H), 4.04 (d, *J* = 11.4 Hz, 1.0H), 3.91 (ddd, *J* = 11.7, 4.5, 2.2 Hz, 2.7H), 3.85 – 3.76 (m, 2.6H), 3.60 – 3.22 (m, 17.0H), 3.04 (ddd, *J* = 12.6, 9.2, 4.9 Hz, 0.9H), 2.90 – 2.75 (m, 2.5H), 2.50 – 2.03 (m, 27.5H), 1.97 – 1.81 (m, 5.2H), 1.80 – 1.47 (m, 8.2H), 1.38 – 1.19 (m, 5.9H), 1.17 – 1.03 (m, 6.7H).

^{13}C NMR (101 MHz, $CDCl_3$) δ 207.55, 207.43, 80.09, 79.87, 77.79, 77.61, 76.00, 73.46, 71.55, 64.76, 64.73, 64.38, 64.33, 61.63, 61.55, 53.86, 52.75, 52.35, 52.28, 52.19, 45.97, 45.84, 39.59, 39.16, 39.14, 36.89, 35.90, 32.37, 32.27, 31.63, 31.53, 28.66, 27.85, 27.80, 24.83, 23.72, 22.46, 22.35.

HRMS (ESI): m/z, $[M+H]^+$ calc. for $C_{20}H_{33}N_2O_4$: 365.2435; found: 365.2426.

4.7 Automated reductive amination and purification

GP4: Reductive amination

The reaction was launched, run and worked-up on an Opentrons OT-2 liquid handler using a 1'000 µL and 300 µL pipette and a heater-shaker module. Heat-sealing was performed with an Eppendorf HeatSealer S200 at 165 °C, 1.5 s. The reaction was run in Axygen® 96-well Clear Round Bottom 2 mL Polypropylene Deep Well Plates P-DW-20-C-S. Nitrogen blowdown was performed with EquaVAP® evaporators (24 or 96-well).

Silica supported cyanoborohydride (0.13 g, 0.95 – 1.1 mmol/g, 0.12 – 0.15 mmol, 5 – 6 equiv) was transferred to each well with a calibrated spoon. The ketone solution (0.078 M in 2:1 MeOH / HFIP, 0.33 mL, 0.025 mmol, 1.0 equiv) and amine solution (0.083 M in MeOH, 0.30 mL, 0.025 mmol, 1.0 equiv) were added. The well plate was heat-sealed and shaken at 900 rpm overnight at rt. The silica supported reagent was removed by sedimentation for 2 min, collecting the solution and extracting the silica 2 x with MeOH (0.30 mL). The collected extracts were evaporated by N_2 blowdown. The crude mixtures were filtered with a syringe filter (0.22 µM pore size) before HPLC injection.

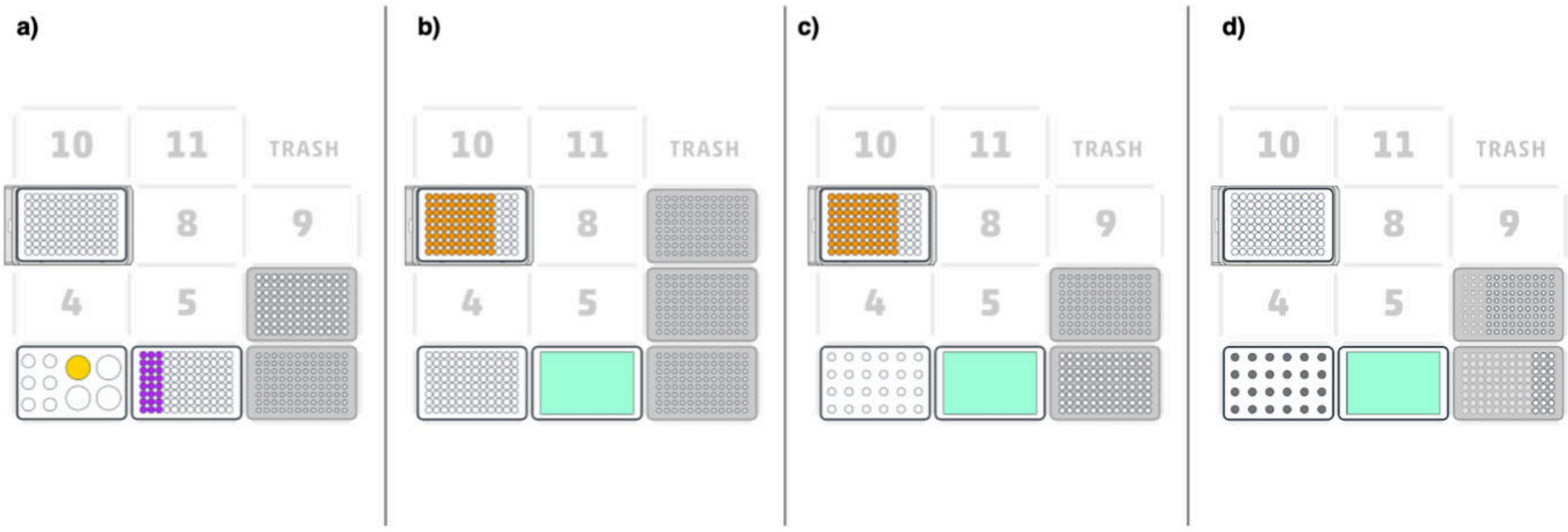

Figure 4.7.1: **a)** *Reaction launch (starting deck, ketone solution yellow, amine solutions violet); ketone is combined with all amines separately 2 x each.* **b)** *Silica removal (starting deck, reaction suspensions orange, methanol turquoise), transfer from reaction plate to evaporation plate and silica extractions.* **c)** *Dissolving of crude mixtures from evaporation plate and combining triplicates into collection vial each on 24 vial rack (starting deck, evaporated reaction extracts orange, methanol turquoise).* **d)** *Ending deck with crude mixtures in vials (crude mixtures grey, methanol turquoise).*

4.7.1 Separated diastereomers

57. (57a&b) 9s,12s)-7-methyl-N-(pyridin-4-ylmethyl)-3,21,25-trioxa-7,13-diazatetraspiro[5.2.2.2.5^{15}.2^{12}.2^{9}.2^{6}]-hexacosan-18-amine

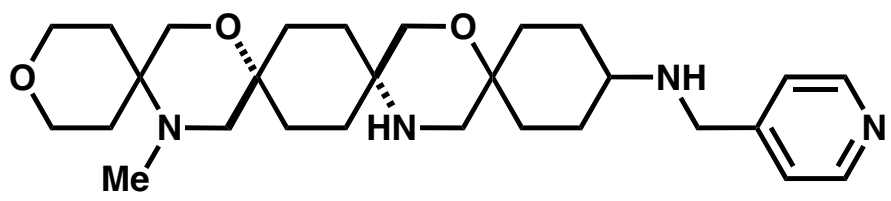

The 9*s*,12*s* pentacyclic ketone **17a** (20 mg, 0.050 mmol, 1.0 equiv) was combined with pyridin-4-ylmethanamine (5.4 mg, 0.050 mmol, 1.0 equiv). MeOH (1.0 mL), HFIP (0.25 mL) and silica supported cyanoborohydride (1.0 mmol/g, 0.26 g, 0.25 mmol, 5.0 equiv). The suspension was stirred overnight at rt. Subsequently, the suspension was filtered through a plug of celite through a fritted syringe and syringe filter (0.22 µm). The crude solution was concentrated *under reduced pressure* and purified with diastereomer separation by SFC (Daicel CHIRALPAK® IK, 20 x 250 mm, 55% MeOH + 0.2% Et_2NH).

The relative configuration was elucidated by comparison of experimental to predicted ^{13}C NMR chemical shifts and confirmed by X-Ray crystallography (*vide infra*).

57a (9S,12s,15s,18S)-7-methyl-N-(pyridin-4-ylmethyl)-3,21,25-trioxa-7,13-diazatetraspiro-[5.2.2.2.515.212.29.26]hexacosan-18-amine (6.0, 12 µmol, 24% yield)

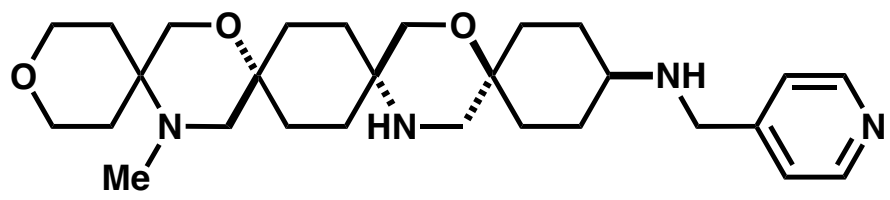

^{1}H NMR (600 MHz, $CDCl_3$) δ 8.54 – 8.50 (m, 2H), 7.28 – 7.24 (m, 2H), 3.89 – 3.82 (m, 4H), 3.68 (s, 2H), 3.48 (td, *J* = 12.3, 2.2 Hz, 2H), 3.44 (s, 2H), 2.65 (s, 2H), 2.47 (tt, *J* = 10.9, 3.9 Hz, 1H), 2.39 (s, 2H), 2.23 (s, 3H), 2.10 (d, *J* = 13.6 Hz, 2H), 1.94 – 1.85 (m, 4H), 1.76 – 1.68 (m, 2H), 1.67 – 1.51 (m, 5H), 1.48 (d, *J* = 13.4 Hz, 2H), 1.46 – 1.21 (m, 5H), 1.11 (td, *J* = 13.6, 3.7 Hz, 2H).

^{13}C NMR (151 MHz, $CDCl_3$) δ 150.2, 149.9, 123.0, 71.6, 70.2, 66.5, 64.7, 64.7, 57.8, 56.4, 52.8, 50.0, 49.9, 49.9, 37.0, 30.9, 29.2, 28.8, 28.1, 26.3.

HRMS (ESI): m/z, $[M+H]^+$ calc. for $C_{28}H_{45}N_4O_3$: 485.3486; found: 485.3493.

IR (ν/cm^{-1}, thin film) 2923, 2849, 2794, 1599, 1441, 1409, 1369, 1358, 1261, 1240, 1220, 1179, 1166, 1154, 1128, 1117, 1105, 1074, 1055, 1028, 1003, 992, 975, 943, 934, 916, 875, 855, 846, 833, 819, 805, 791, 760, 731, 701, 662, 632, 614, 596.

57b (9S,12s,15r,18R)-7-methyl-N-(pyridin-4-ylmethyl)-3,21,25-trioxa-7,13-diazatetraspiro-[5.2.2.2.515.212.29.26]hexacosan-18-amine (3.0, 6.0 µmol, 12% yield)

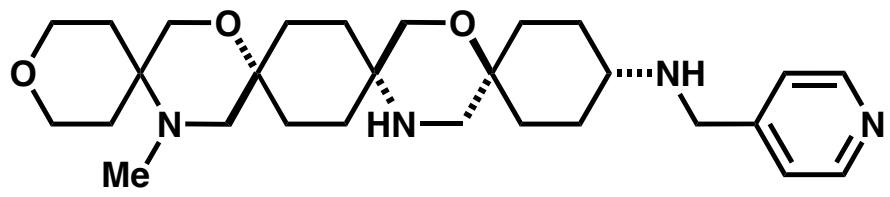

^{1}H NMR (600 MHz, $CDCl_3$) δ 8.55 – 8.51 (m, 2H), 7.26 – 7.24 (m, 2H), 3.87 (dd, J = 11.9, 5.3 Hz, 2H), 3.80 (s, 2H), 3.69 (s, 2H), 2.81 (s, 2H), 2.65 (tt, J = 8.1, 3.7 Hz, 1H), 2.40 (s, 2H), 2.24 (s, 3H), 1.95 – 1.81 (m, 8H), 1.63 – 1.45 (m, 10H), 1.43 – 1.35 (m, 4H), 1.30 – 1.21 (m, 4H).

^{13}C NMR (151 MHz, $CDCl_3$) δ 150.1, 150.0, 123.1, 71.7, 71.6, 67.2, 64.7, 64.7, 57.8, 55.0, 52.8, 50.4, 49.9, 47.0, 37.0, 30.3, 29.2, 28.9, 28.5, 26.3.

HRMS (ESI): m/z, $[M+H]^+$ calc. for $C_{28}H_{45}N_4O_3$: 485.3486; found: 485.3491.

IR (ν/cm^{-1}, thin film) 2925, 2854, 2800, 1731, 1671, 1598, 1456, 1413, 1356, 1303, 1260, 1221, 1082, 1025, 855, 800, 507.

57 (57c&d) (9r,12r)-7-methyl-N-(pyridin-4-ylmethyl)-3,21,25-trioxa-7,13-diazatetraspiro[5.2.2.2.5^{15}.2^{12}.2^{9}.2^{6}]-hexacosan-18-amine

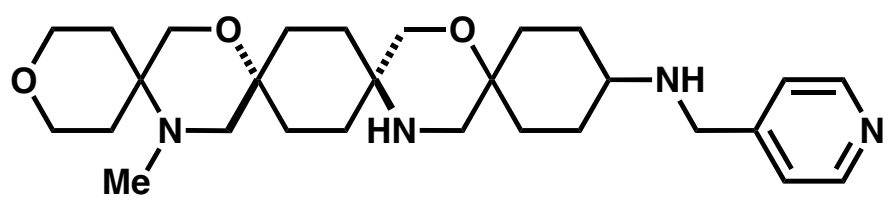

The 9*r*,12*r* pentacyclic ketone **17b** (79 mg, 0.20 mmol, 1.0 equiv) was combined with pyridin-4-ylmethanamine (22 mg, 0.20 mmol, 1.0 equiv). MeOH (1.3 mL), HFIP (0.33 mL) and silica supported cyanoborohydride (1.1 mmol/g, 0.92 g, 1.0 mmol, 5.0 equiv). The suspension was stirred overnight at rt. Subsequently, the suspension was filtered through a plug of celite packed in a fritted syringe equipped with a syringe filter (0.22 µm). The crude solution was concentrated *under reduced pressure* and purified with diastereomer separation by SFC (Daicel CHIRALCEL® OD-H, 20 x 250 mm, 25% MeOH + 0.2% Et_2NH).

The relative configuration was elucidated by comparison of experimental to predicted ^{13}C NMR chemical shifts and confirmed by X-Ray crystallography (*vide infra*).

57c (9R,12r,15s,18S)-7-methyl-N-(pyridin-4-ylmethyl)-3,21,25-trioxa-7,13-diazatetraspiro-[5.2.2.2.5^{15}.2^{12}.2^{9}.2^{6}]hexacosan-18-amine (45 mg, 92 µmol, 46% yield)

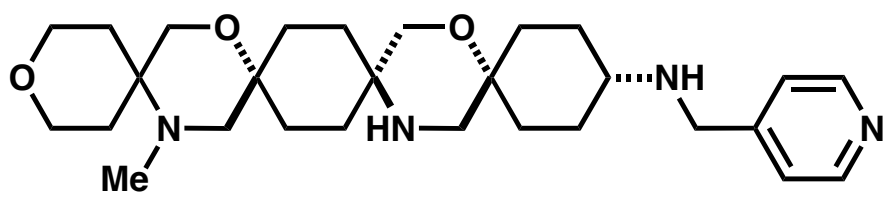

^{1}H NMR (600 MHz, $CDCl_3$) δ 8.51 (d, *J* = 5.0 Hz, 2H), 7.26 (d, *J* = 6.0 Hz, 2H), 3.88 – 3.82 (m, 4H), 3.67 (s, 2H), 3.48 (td, *J* = 12.2, 2.2 Hz, 2H), 3.33 (s, 2H), 2.58 (s, 2H), 2.45 (ddt, *J* = 10.8, 7.6, 3.8 Hz, 1H), 2.40 (s, 2H), 2.22 (s, 3H), 2.05 (d, *J* = 13.6 Hz, 2H), 1.90 (td, *J* = 13.0, 5.4 Hz, 3H), 1.81 (d, *J* = 10.6 Hz, 3H), 1.72 (dd, *J* = 13.0, 3.8 Hz, 2H), 1.57 – 1.46 (m, 8H), 1.42 (dddd, *J* = 13.1, 12.9, 3.3 Hz, 2H), 1.08 (td, *J* = 13.6, 3.7 Hz, 2H).

^{13}C NMR (151 MHz, $CDCl_3$) δ 150.2, 149.9, 123.1, 71.7, 69.7, 69.6, 64.7, 64.7, 58.6, 56.3, 52.9, 49.9, 49.8, 49.8, 37.0, 30.7, 28.0, 27.9, 27.5, 26.3.

HRMS (ESI): m/z, $[M+H]^+$ calc. for $C_{28}H_{45}N_4O_3$: 485.3486; found: 485.3485.

IR (ν/cm^{-1}, thin film) 2927, 2854, 2803, 1601, 1456, 1441, 1414, 1367, 1353, 1303, 1223, 1178, 1142, 1117, 1103, 1077, 1025, 1014, 999, 977, 935, 924, 880, 843, 832, 799, 764, 731, 700, 668, 658, 639, 626, 617, 600, 590, 581, 577, 559, 526, 518, 513.

57d (9R,12r,15r,18R)-7-methyl-N-(pyridin-4-ylmethyl)-3,21,25-trioxa-7,13-diazatetraspiro[5.2.2.2.5^{15}.2^{12}.2^{9}.2^{6}]hexacosan-18-amine (20 mg, 41 µmol, 20% yield)

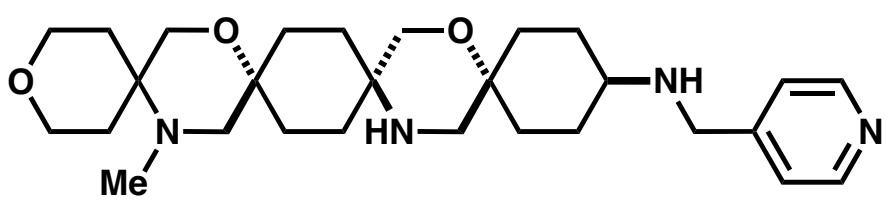

^{1}H NMR (600 MHz, $CDCl_3$) δ 8.52 (d, *J* = 5.1 Hz, 2H), 7.25 (d, *J* = 5.0 Hz, 1H), 3.86 (dd, *J* = 11.9, 5.2 Hz, 2H), 3.79 (s, 2H), 3.67 (s, 2H), 3.48 (td, *J* = 12.3, 2.2 Hz, 2H), 3.39 (s, 2H), 2.75 (s, 2H), 2.71 – 2.63 (m, 1H), 2.42 (s, 2H), 2.22 (s, 3H), 1.96 – 1.78 (m, 9H), 1.64 – 1.43 (m, 12H), 1.31 – 1.22 (m, 2H).

^{13}C NMR (151 MHz, $CDCl_3$) δ 150.0, 149.9, 123.1, 71.7, 70.9, 70.2, 64.7, 64.7, 58.6, 54.8, 52.8, 50.4, 49.8, 47.0, 37.0, 29.9, 28.2, 27.9, 27.4, 26.3.

HRMS (ESI): m/z, $[M+H]^+$ calc. for $C_{28}H_{45}N_4O_3$: 485.3486; found: 485.3484.

IR (ν/cm^{-1}, thin film) 2932, 2856, 2802, 1601, 1455, 1414, 1367, 1353, 1303, 1265, 1223, 1178, 1155, 1117, 1103, 1079, 1025, 1014, 994, 975, 949, 935, 920, 878, 845, 831, 804, 776, 701, 677, 667, 649, 639, 614, 596, 570, 557, 546, 540, 532, 523, 518, 513, 509, 504.

58. **(58a–c)** ***(4aR,8aR)-N-((5-fluoro-1H-indol-2-yl)methyl)-4''-methyldecahydro-2H,4H-trispiro-[benzo[b][1,4]oxazine-3,1'-cyclohexane-4',2''-morpholine-5'',4'''-pyran]-7-amine***

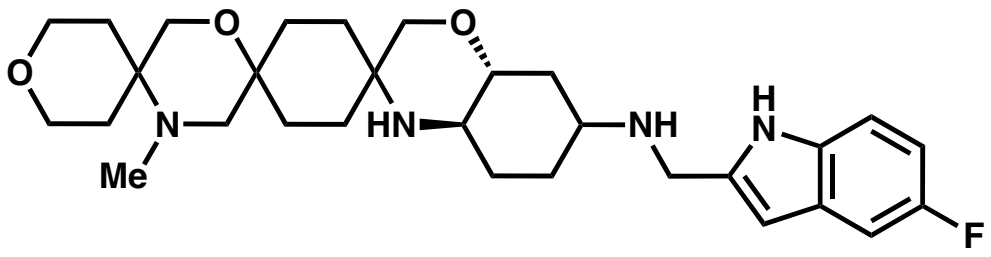

The pentacyclic ketone **22** (0.19 g, 0.50 mmol, 1.0 equiv) was combined with (5-fluoro-1*H*-indol-2-yl)methanamine (82 mg, 0.50 mmol, 1.0 equiv). MeOH (3.3 mL), HFIP (0.83 mL) and silica supported cyanoborohydride (1.1 mmol/g, 2.3 g, 1.1 mmol, 5.0 equiv). The suspension was stirred overnight at rt. Subsequently, the suspension was filtered through a plug of celite packed in a fritted syringe equipped with a syringe filter (0.22 µm). The crude solution was concentrated *under reduced pressure* and purified with diastereomer separation by SFC (Daicel CHIRALPAK® IA, 20 x 250 mm, 36% MeOH + 0.2% Et_2NH) delivering the 4 separated diastereomers.

The relative configuration was elucidated by comparison of experimental to predicted ^{13}C NMR chemical shifts and confirmed for the 3*r*,4a*R*,4'*R*,7*R*,8a*R* diastereomer (**58c**) by X-Ray crystallography (*vide infra*).

58a (3s,4aR,4'S,7R,8aR)-N-((5-fluoro-1H-indol-2-yl)methyl)-4''-methyldecahydro-2H,4H-trispiro-[benzo[b][1,4]oxazine-3,1'-cyclohexane-4',2''-morpholine-5'',4'''-pyran]-7-amine (14 mg, 27 µmol, 5.4% yield)

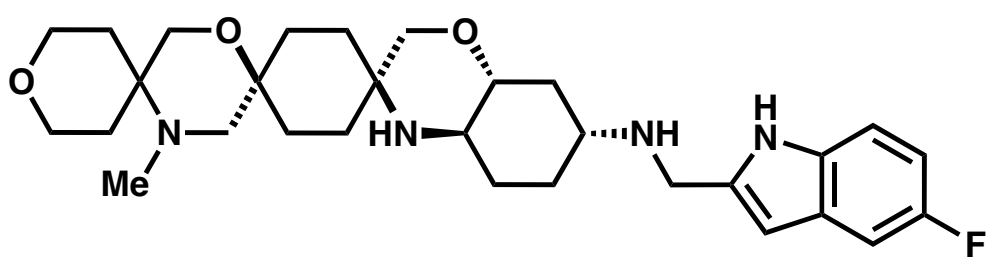

^1H NMR (600 MHz, $CDCl_3$) δ 8.64 (s, 1H), 7.22 (ddt, *J* = 8.7, 4.4, 0.7 Hz, 1H), 7.17 (dd, *J* = 9.6, 2.5 Hz, 1H), 6.87 (ddd, *J* = 9.4, 8.8, 2.5 Hz, 1H), 6.28 (dd, *J* = 2.0, 1.0 Hz, 1H), 3.99 (s, 2H), 3.90 – 3.84 (m, 3H), 3.68 (s, 2H), 3.49 (tdd, *J* = 12.2, 4.6, 2.3 Hz, 2H), 3.21 (dd, *J* = 11.2, 1.2 Hz, 1H), 2.90 (ddd, *J* = 11.5, 9.0, 3.9 Hz, 1H), 2.73 – 2.62 (m, 2H), 2.43 – 2.36 (m, 2H), 2.24 (s, 3H), 2.18 (dtd, *J* = 11.7, 3.8, 2.1 Hz, 1H), 2.03 – 1.85 (m, 6H), 1.61 (td, *J* = 12.8, 3.5 Hz, 2H), 1.53 – 1.44 (m, 4H), 1.44 – 1.14 (m, 7H).

^{13}C NMR (151 MHz, $CDCl_3$) δ 158.0 (d, *J* = 233.7 Hz), 139.7, 132.5, 129.0 (d, *J* = 10.2 Hz), 111.3 (d, *J* = 9.7 Hz), 109.8 (d, *J* = 26.2 Hz), 105.1 (d, *J* = 23.5 Hz), 100.1 (d, *J* = 4.5 Hz), 80.2, 73.8, 71.5, 64.8, 64.7, 64.7, 64.7, 57.9, 55.3, 54.0, 52.8, 51.6, 44.3, 37.5, 37.0, 32.2, 31.8, 29.5, 29.4, 29.1, 26.8, 26.3.

^{19}F NMR (565 MHz, $CDCl_3$) δ -125.11 (td, *J* = 9.5, 4.4 Hz, 1F).

HRMS (ESI): m/z, $[M+H]^+$ calc. for $C_{30}H_{44}FN_4O_3$: 527.3392; found: 527.3392.

IR (ν/cm^{-1}, thin film) 3277, 2926, 2854, 2804, 1627, 1586, 1486, 1452, 1352, 1304, 1265, 1222, 1169, 1101, 1092, 1068, 1025, 1014, 952, 854, 832, 787, 764, 733, 701, 600.

$[\alpha]_D^{26}$ –88.52 ° (c 0.05, CH_2Cl_2).

58b (3s,4aR,4'S,7S,8aR)-N-((5-fluoro-1H-indol-2-yl)methyl)-4''-methyldecahydro-2H,4H-trispiro-[benzo[b][1,4]oxazine-3,1'-cyclohexane-4',2''-morpholine-5'',4'''-pyran]-7-amine (32 mg, 61 µmol, 12% yield)

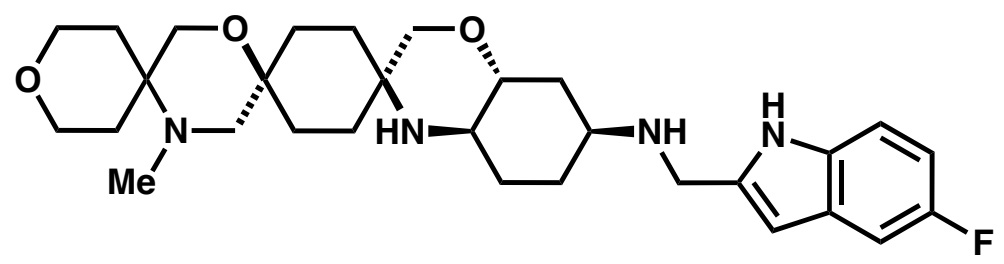

^{1}H NMR (600 MHz, $CDCl_3$) δ 8.52 (s, 1H), 7.25 – 7.22 (m, 1H), 7.17 (dd, *J* = 9.7, 2.5 Hz, 1H), 6.88 (td, *J* = 9.1, 2.6 Hz, 1H), 6.27 (s, 1H), 4.00 – 3.84 (m, 4H), 3.76 – 3.63 (m, 3H), 3.53 – 3.45 (m, 2H), 3.34 – 3.30 (m, 1H), 3.13 (t, *J* = 3.0 Hz, 1H), 2.60 (td, *J* = 9.8, 4.2 Hz, 1H), 2.48 – 2.40 (m, 2H), 2.24 (s, 3H), 2.01 – 1.75 (m, 7H), 1.67 (td, *J* = 12.9, 3.8 Hz, 2H), 1.58 – 1.42 (m, 11H), 1.17 – 1.11 (m, 1H).

^{13}C NMR (151 MHz, $CDCl_3$) δ 158.0 (d, *J* = 233.6 Hz), 140.1, 132.4 (d, *J* = 3.7 Hz), 129.0 (d, *J* = 10.2 Hz), 111.3 (d, *J* = 9.8 Hz), 109.7 (d, *J* = 26.2 Hz), 105.0 (d, *J* = 23.5 Hz), 99.9 (d, *J* = 4.4 Hz), 78.2, 77.5, 71.7, 64.8, 64.8, 64.7, 58.4, 54.7, 53.2, 52.9, 51.3, 44.9, 37.0 (d, *J* = 1.6 Hz), 35.1, 31.1, 29.0, 28.7, 27.6, 26.7, 26.6, 25.8, 25.4.

^{19}F NMR (565 MHz, $CDCl_3$) δ -125.14 (td, *J* = 9.5, 4.4 Hz, 1F).

HRMS (ESI): m/z, $[M+H]^+$ calc. for $C_{30}H_{44}FN_4O_3$: 527.3392; found: 527.3393.

IR (ν/cm^{-1}, thin film) 3287, 2944, 2857, 1628, 1582, 1486, 1453, 1349, 1307, 1223, 1169, 1117, 1093, 1025, 954, 854, 784, 765, 757, 732, 504.

$[\alpha]_D^{25}$ –99.32 ° (c 0.05, CH_2Cl_2).

58c (3r,4aR,4'R,7R,8aR)-N-((5-fluoro-1H-indol-2-yl)methyl)-4''-methyldecahydro-2H,4H-trispiro-[benzo[b][1,4]oxazine-3,1'-cyclohexane-4',2''-morpholine-5'',4'''-pyran]-7-amine (46 mg, 87 µmol, 17% yield)

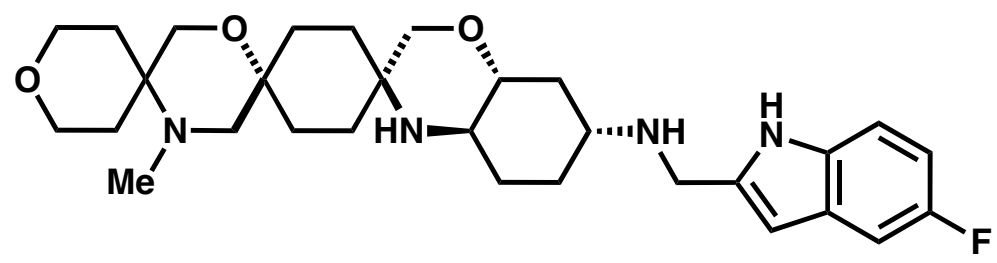

^{1}H NMR (600 MHz, $CDCl_3$) δ 8.73 (s, 1H), 7.19 (dd, *J* = 8.8, 4.4 Hz, 1H), 7.16 (dd, *J* = 9.6, 2.5 Hz, 1H), 6.85 (ddd, *J* = 9.3, 8.7, 2.5 Hz, 1H), 6.26 (dd, *J* = 2.1, 1.0 Hz, 1H), 3.95 (t, *J* = 1.2 Hz, 2H), 3.86 (dt, *J* = 12.7, 6.4 Hz, 2H), 3.73 – 3.62 (m, 3H), 3.48 (tdd, *J* = 12.6, 10.6, 2.3 Hz, 2H), 3.21 (d, *J* = 11.3 Hz, 1H), 2.83 (ddd, *J* = 11.4, 9.1, 3.9 Hz, 1H), 2.63 (tt, *J* = 11.2, 3.9 Hz, 1H), 2.53 (ddd, *J* = 10.8, 9.1, 3.8 Hz, 1H), 2.46 – 2.37 (m, 2H), 2.22 (s, 3H), 2.20 – 2.13 (m, 1H), 1.99 – 1.86 (m, 4H), 1.80 (tff, *J* = 15.3 Hz, 2H), 1.68 – 1.59 (m, 2H), 1.57 – 1.30 (m, 7H), 1.27 – 1.06 (m, 4H).

^{13}C NMR (151 MHz, $CDCl_3$) δ 157.9 (d, *J* = 233.6 Hz), 139.9, 132.4, 128.9 (d, *J* = 10.2 Hz), 111.3 (d, *J* = 9.7 Hz), 109.6 (d, *J* = 26.2 Hz), 105.0 (d, *J* = 23.4 Hz), 100.0 (d, *J* = 4.4 Hz), 80.5, 77.3, 71.7, 64.7, 64.7, 64.6, 58.4, 55.2, 54.0, 52.8, 51.1, 44.4, 37.7, 37.0, 32.1, 31.0, 29.4, 28.6, 27.5, 26.6, 25.8, 25.3.

^{19}F NMR (565 MHz, $CDCl_3$) δ -125.10 (td, *J* = 9.5, 4.4 Hz, 1F).

HRMS (ESI): m/z, $[M+H]^+$ calc. for $C_{30}H_{44}FN_4O_3$: 527.3392; found: 527.3392.

IR (ν/cm^{-1}, thin film) 3284, 2927, 2854, 1671, 1628, 1586, 1488, 1453, 1352, 1304, 1262, 1222, 1171, 1117, 1092, 1067, 1025, 1014, 853, 797, 763, 736, 599.

$[\alpha]_D^{26}$ –66.80 ° (c 0.05, CH_2Cl_2).

58d (3r,4aR,4'R,7S,8aR)-N-((5-fluoro-1H-indol-2-yl)methyl)-4''-methyldecahydro-2H,4H-trispiro-[benzo[b][1,4]oxazine-3,1'-cyclohexane-4',2''-morpholine-5'',4'''-pyran]-7-amine (15 mg, 28 µmol, 5.7% yield)

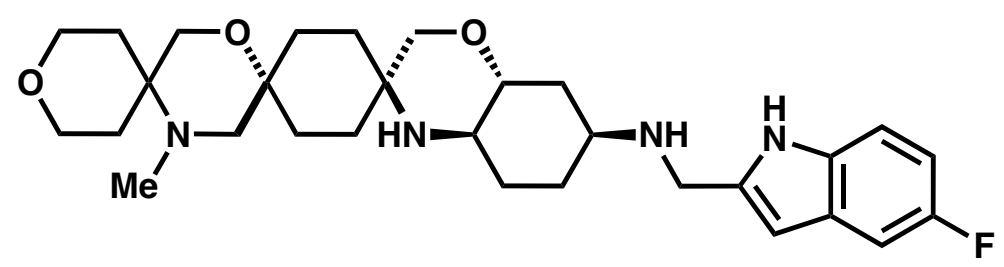

^{1}H NMR (600 MHz, $CDCl_3$) δ 8.56 (s, 1H), 7.26 – 7.23 (m, 1H), 7.19 – 7.14 (m, 1H), 6.87 (ddd, *J* = 9.4, 8.8, 2.5 Hz, 1H), 6.29 – 6.24 (m, 1H), 4.00 – 3.84 (m, 5H), 3.69 (s, 2H), 3.49 (tt, *J* = 12.2, 2.6 Hz, 2H), 3.38 (q, *J* = 7.3 Hz, 1H), 3.33 – 3.28 (m, 1H), 3.13 (t, *J* = 3.1 Hz, 1H), 2.76 (ddd, *J* = 11.2, 9.1, 4.0 Hz, 1H), 2.45 – 2.37 (m, 2H), 2.24 (s, 3H), 2.10 – 2.01 (m, 1H), 2.01 – 1.84 (m, 5H), 1.77 (dt, *J* = 13.7, 2.7 Hz, 1H), 1.65 – 1.24 (m, 13H).

^{13}C NMR (151 MHz, $CDCl_3$) δ 158.0 (d, *J* = 233.6 Hz), 140.1, 132.4, 129.0 (d, *J* = 10.2 Hz), 111.3 (d, *J* = 9.7 Hz), 109.7 (d, *J* = 26.2 Hz), 105.0 (d, *J* = 23.5 Hz), 99.8 (d, *J* = 4.5 Hz), 77.8, 73.9, 71.5, 64.7, 64.7, 64.7, 58.0, 54.8, 53.2, 52.8, 51.8, 44.9, 37.0, 34.9, 32.2, 29.6, 29.1, 28.7, 26.9, 26.5, 26.3. 1 C overlapping.

^{19}F NMR (565 MHz, $CDCl_3$) δ -125.20 (td, *J* = 9.5, 4.4 Hz, 1F).

HRMS (ESI): m/z, $[M+H]^+$ calc. for $C_{30}H_{44}FN_4O_3$: 527.3392; found: 527.3392.

IR (ν/cm^{-1}, thin film) 3277, 2927, 2855, 2806, 1628, 1586, 1486, 1453, 1357, 1303, 1223, 1169, 1102, 1072, 1025, 854, 787, 761, 735.

$[\alpha]_D^{25}$ –18.20 ° (c 0.05, CH_2Cl_2).

59.* (59a–c) *(4aS,4a'S,8aS,8a'S)-N-(2-(1H-indol-3-yl)ethyl)-4'-methylhexadecahydro-2H,2'H,4H-dispiro-[benzo[b][1,4]oxazine-3,7'-benzo[b][1,4]oxazine-3',4''-pyran]-6-amine

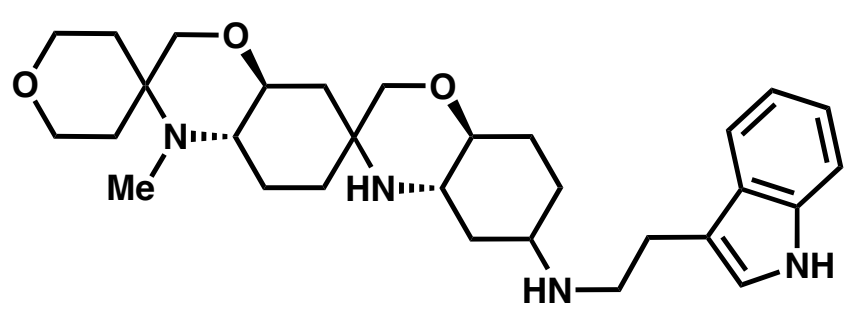

The pentacyclic ketone **56** (73 mg, 0.20 mmol, 1.0 equiv) was combined with (2-(1*H*-indol-3-yl)ethan-1-amine (32 mg, 0.20 mmol, 1.0 equiv). MeOH (1.3 mL), HFIP (0.34 mL) and silica supported cyanoborohydride (1.1 mmol/g, 0.90 g, 1.1 mmol, 5.0 equiv). The suspension was stirred overnight at rt. Subsequently, the suspension was filtered through a plug of celite packed in a fritted syringe equipped with a syringe filter (0.22 µm). The crude solution was concentrated *under reduced pressure* and purified with diastereomer separation by SFC (Daicel CHIRALPAK® IB, 20 x 250 mm, 33% MeOH + 0.2% Et_2NH) delivering the 4 separated diastereomers.

The relative configuration was elucidated by comparison of experimental to predicted ^{13}C NMR chemical shifts.

59a (3S,4aS,4a'S,6R,8aS,8a'S)-N-(2-(1H-indol-3-yl)ethyl)-4'-methylhexadecahydro-2H,2'H,4H-dispiro[benzo[b][1,4]oxazine-3,7'-benzo[b][1,4]oxazine-3',4''-pyran]-6-amine (13 mg, 26 µmol, 13% yield)

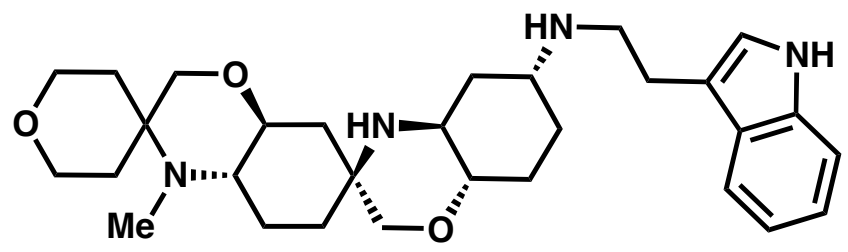

¹H NMR (500 MHz, $CDCl_3$) δ 8.18 (s, 1H), 7.62 (d, *J* = 7.9 Hz, 1H), 7.34 (d, *J* = 8.0 Hz, 1H), 7.18 (t, *J* = 7.5 Hz, 1H), 7.11 (t, *J* = 7.5 Hz, 1H), 7.06 (s, 1H), 4.40 (d, *J* = 11.6 Hz, 1H), 3.97 (d, *J* = 11.5 Hz, 2H), 3.84 (dd, *J* = 12.3, 5.3 Hz, 1H), 3.61 (td, *J* = 12.4, 2.6 Hz, 1H), 3.53 – 3.40 (m, 1H), 3.36 – 3.25 (m, 2H), 3.17 (d, *J* = 11.4 Hz, 1H), 3.07 – 2.78 (m, 8H), 2.38 – 2.27 (m, 1H), 2.26 (s, 2H), 2.19 (td, *J* = 13.1, 5.5 Hz, 1H), 1.95 (d, *J* = 13.6 Hz, 1H), 1.92 – 1.86 (m, 1H), 1.85 – 1.71 (m, 2H), 1.67 – 1.42 (m, 6H), 1.29 – 1.01 (m, 7H).

¹³C NMR (126 MHz, $CDCl_3$) δ 136.5, 127.6, 122.1, 122.1, 119.4, 119.0, 114.4, 111.4, 82.9, 78.0, 73.7, 71.7, 65.0, 64.6, 61.8, 54.0, 53.0, 52.0, 49.0, 47.5, 37.0, 36.0, 35.5, 32.4, 31.8, 29.3, 25.9, 25.3, 25.0, 22.6.

HRMS (ESI): m/z, $[M+H]^+$ calc. for $C_{30}H_{45}N_4O_3$: 509.3486; found: 509.3488.

IR (ν/cm^{-1}, thin film) 3274, 2925, 2854, 1667, 1618, 1453, 1355, 1262, 1171, 1119, 1099, 1064, 1029, 1016, 836, 804, 739, 668.

$[\alpha]_D^{26}$ –58.12 ° (c 0.05, CH_2Cl_2).

59b (3S,4aS,4a'S,6S,8aS,8a'S)-N-(2-(1H-indol-3-yl)ethyl)-4'-methylhexadecahydro-2H,2'H,4H-dispiro[benzo[b][1,4]oxazine-3,7'-benzo[b][1,4]oxazine-3',4''-pyran]-6-amine (10 mg, 20 µmol, 10% yield)

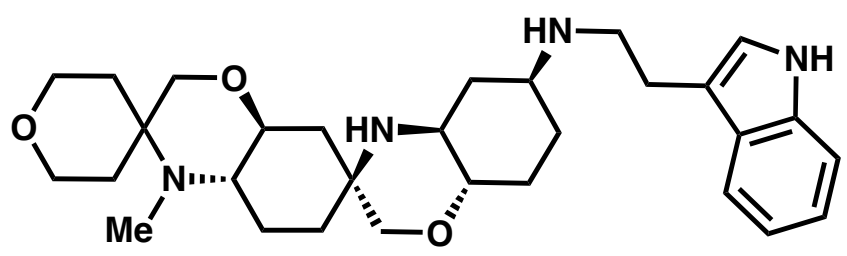

¹H NMR (500 MHz, $CDCl_3$) δ 8.07 (s, 1H), 7.60 (d, *J* = 8.0 Hz, 1H), 7.36 (d, *J* = 8.2 Hz, 1H), 7.19 (ddd, *J* = 8.2, 7.0, 1.2 Hz, 1H), 7.11 (ddd, *J* = 7.9, 7.0, 1.0 Hz, 1H), 7.02 (d, *J* = 2.0 Hz, 1H), 4.34 (d, *J* = 11.7 Hz, 1H), 4.01 (d, *J* = 11.5 Hz, 1H), 3.93 (ddt, *J* = 11.8, 5.6, 1.4 Hz, 1H), 3.82 (ddt, *J* = 12.8, 5.3, 1.3 Hz, 1H), 3.56 (td, *J* = 12.2, 2.6 Hz, 1H), 3.49 – 3.38 (m, 1H), 3.35 – 3.23 (m, 2H), 3.17 (dd, *J* = 11.4, 1.9 Hz, 1H), 2.98 – 2.94 (m, 3H), 2.88 (ddd, *J* = 11.2, 9.1, 4.1 Hz, 1H), 2.80 (ddd, *J* = 12.4, 4.3, 2.4 Hz, 1H), 2.72 (ddd, *J* = 11.6, 9.1, 3.6 Hz, 1H), 2.61 (tt, *J* = 11.2, 3.8 Hz, 1H), 2.34 (ddd, *J* = 11.4, 9.4, 3.7 Hz, 1H), 2.25 (s, 3H), 2.17 (ddd, *J* = 13.7, 12.5, 5.5 Hz, 1H), 1.98 – 1.70 (m, 6H), 1.55 (dq, *J* = 14.2, 3.6 Hz, 1H), 1.45 – 0.94 (m, 10H).

¹³C NMR (126 MHz, $CDCl_3$) δ 136.5, 127.6, 122.2, 122.0, 119.5, 119.0, 114.1, 111.3, 82.4, 77.9, 73.8, 71.7, 65.0, 64.6, 61.8, 55.5, 53.9, 52.8, 52.6, 47.4, 38.5, 37.0, 36.1, 32.4, 31.8, 31.7, 28.7, 26.1, 25.0, 22.5.

HRMS (ESI): m/z, $[M+H]^+$ calc. for $C_{30}H_{45}N_4O_3$: 509.3486; found: 509.3485.

IR (ν/cm^{-1}, thin film) 3268, 2925, 2855, 1622, 1452, 1355, 1265, 1191, 1171, 1117, 1092, 1065, 1029, 1015, 939, 835, 735, 700, 667.

[α]$_D^{26}$ –73.80 ° (c 0.05, CH_2Cl_2).

59c (3R,4aS,4a'S,6R,8aS,8a'S)-N-(2-(1H-indol-3-yl)ethyl)-4'-methylhexadecahydro-2H,2'H,4H-dispiro[benzo[b][1,4]oxazine-3,7'-benzo[b][1,4]oxazine-3',4''-pyran]-6-amine (11 mg, 22 μmol, 11% yield)

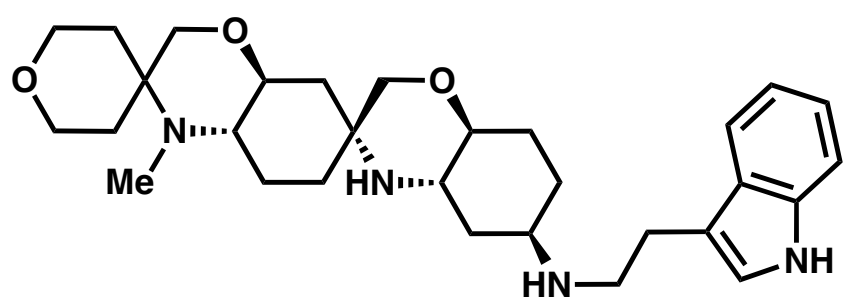

^{1}H NMR (500 MHz, $CDCl_3$) δ 8.06 (s, 1H), 7.62 (dt, *J* = 7.9, 1.0 Hz, 1H), 7.36 (dt, *J* = 8.1, 0.9 Hz, 1H), 7.19 (ddd, *J* = 8.2, 7.0, 1.2 Hz, 1H), 7.11 (ddd, *J* = 8.0, 7.0, 1.0 Hz, 1H), 7.04 (s, 1H), 4.32 (d, *J* = 11.6 Hz, 1H), 3.93 (dd, *J* = 12.0, 5.3 Hz, 1H), 3.82 (dd, *J* = 11.9, 5.3 Hz, 1H), 3.55 (td, *J* = 12.1, 2.6 Hz, 1H), 3.49 – 3.39 (m, 3H), 3.35 – 3.27 (m, 2H), 2.98 – 2.93 (m, 3H), 2.90 – 2.74 (m, 4H), 2.40 (dd, *J* = 14.2, 3.3 Hz, 1H), 2.29 (s, 4H), 2.23 – 2.13 (m, 1H), 1.90 (dd, *J* = 13.2, 2.3 Hz, 1H), 1.78 (dddd, *J* = 26.5, 14.0, 6.8, 3.7 Hz, 3H), 1.61 (ddt, *J* = 17.8, 7.6, 3.1 Hz, 3H), 1.49 (ddd, *J* = 12.4, 4.4, 3.1 Hz, 2H), 1.31 – 1.16 (m, 4H), 1.12 (dd, *J* = 13.8, 2.1 Hz, 1H), 1.04 (td, *J* = 13.9, 3.6 Hz, 1H), 1.00 – 0.86 (m, 1H).

^{13}C NMR (126 MHz, $CDCl_3$) δ 136.5, 127.7, 122.2, 121.9, 119.4, 119.0, 114.4, 111.3, 83.3, 78.1, 76.4, 71.7, 64.9, 64.5, 61.8, 53.9, 52.9, 52.1, 48.4, 47.6, 40.1, 36.0, 32.6, 31.7, 29.2, 28.9, 25.9, 25.4, 24.1, 22.6.

HRMS (ESI): m/z, $[M+H]^+$ calc. for $C_{30}H_{45}N_4O_3$: 509.3486; found: 509.3486.

IR (ν/cm^{-1}, thin film) 3287, 2927, 2854, 1615, 1447, 1356, 1265, 1192, 1173, 1121, 1102, 1073, 1064, 1024, 834, 766, 739, 701, 661.

[α]$_D^{26}$ –50.00 ° (c 0.05, CH_2Cl_2).

59d (3R,4aS,4a'S,6S,8aS,8a'S)-N-(2-(1H-indol-3-yl)ethyl)-4'-methylhexadecahydro-2H,2'H,4H-dispiro[benzo[b][1,4]oxazine-3,7'-benzo[b][1,4]oxazine-3',4''-pyran]-6-amine (32 mg, 63 μmol, 31% yield)

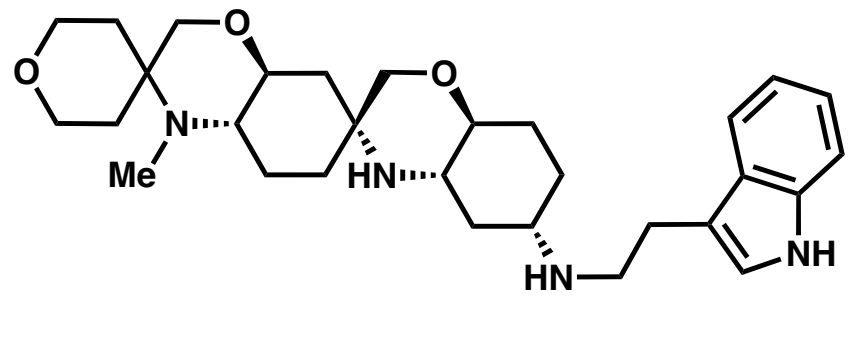

^{1}H NMR (500 MHz, $CDCl_3$) δ 8.27 (s, 1H), 7.61 (d, *J* = 7.9 Hz, 1H), 7.38 – 7.32 (m, 1H), 7.19 (ddd, *J* = 8.2, 7.0, 1.2 Hz, 1H), 7.11 (ddd, *J* = 7.9, 7.0, 0.9 Hz, 1H), 7.03 (d, *J* = 2.1 Hz, 1H), 4.32 (d, *J* = 11.6 Hz, 1H), 3.92 (dd, *J* = 11.8, 5.0 Hz, 1H), 3.82 (dd, *J* = 12.0, 5.0 Hz, 1H), 3.59 – 3.38 (m, 4H), 3.35 – 3.27 (m, 2H), 2.96 (s, 4H), 2.85 (ddd, *J* = 11.2, 9.1, 4.2 Hz, 1H), 2.60 (tt, *J* = 11.1, 3.9 Hz, 1H), 2.55 – 2.47 (m, 1H), 2.42 (dq, *J* = 14.2, 3.2 Hz, 1H), 2.28 (s, 4H), 2.17 (ddd, *J* = 13.8, 12.4, 5.5 Hz, 1H), 1.96 – 1.70 (m, 6H), 1.50 (ddd, *J* = 12.6, 4.2, 2.8 Hz, 1H), 1.44 – 1.02 (m, 8H), 0.98 (q, *J* = 11.6 Hz, 1H).

^{13}C NMR (126 MHz, $CDCl_3$) δ 136.2, 127.2, 121.8, 121.7, 119.1, 118.7, 113.6, 111.0, 82.2, 77.8, 75.9, 71.3, 64.6, 64.1, 61.5, 55.2, 53.6, 52.2, 51.8, 47.0, 39.6, 38.1, 32.2, 31.3, 31.3, 28.6, 28.4, 25.8, 23.6, 22.3.

HRMS (ESI): m/z, $[M+H]^+$ calc. for $C_{30}H_{45}N_4O_3$: 509.3486; found: 509.3485.

IR (ν/cm^{-1}, thin film) 3284, 2928, 2856, 1670, 1618, 1454, 1352, 1261, 1120, 1101, 1064, 1016, 833, 800, 741.

$[\alpha]_D^{26}$ –80.36 ° (c 0.05, CH_2Cl_2).

5 X-RAY CRYSTALLOGRAPHY DATA

Compound 17a (deposition number 2557952)

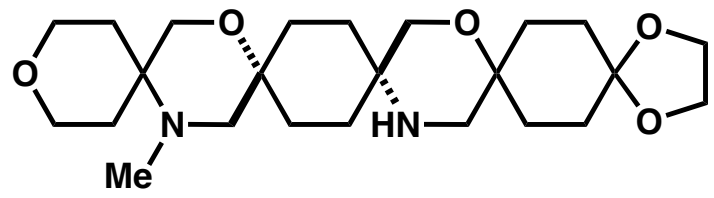

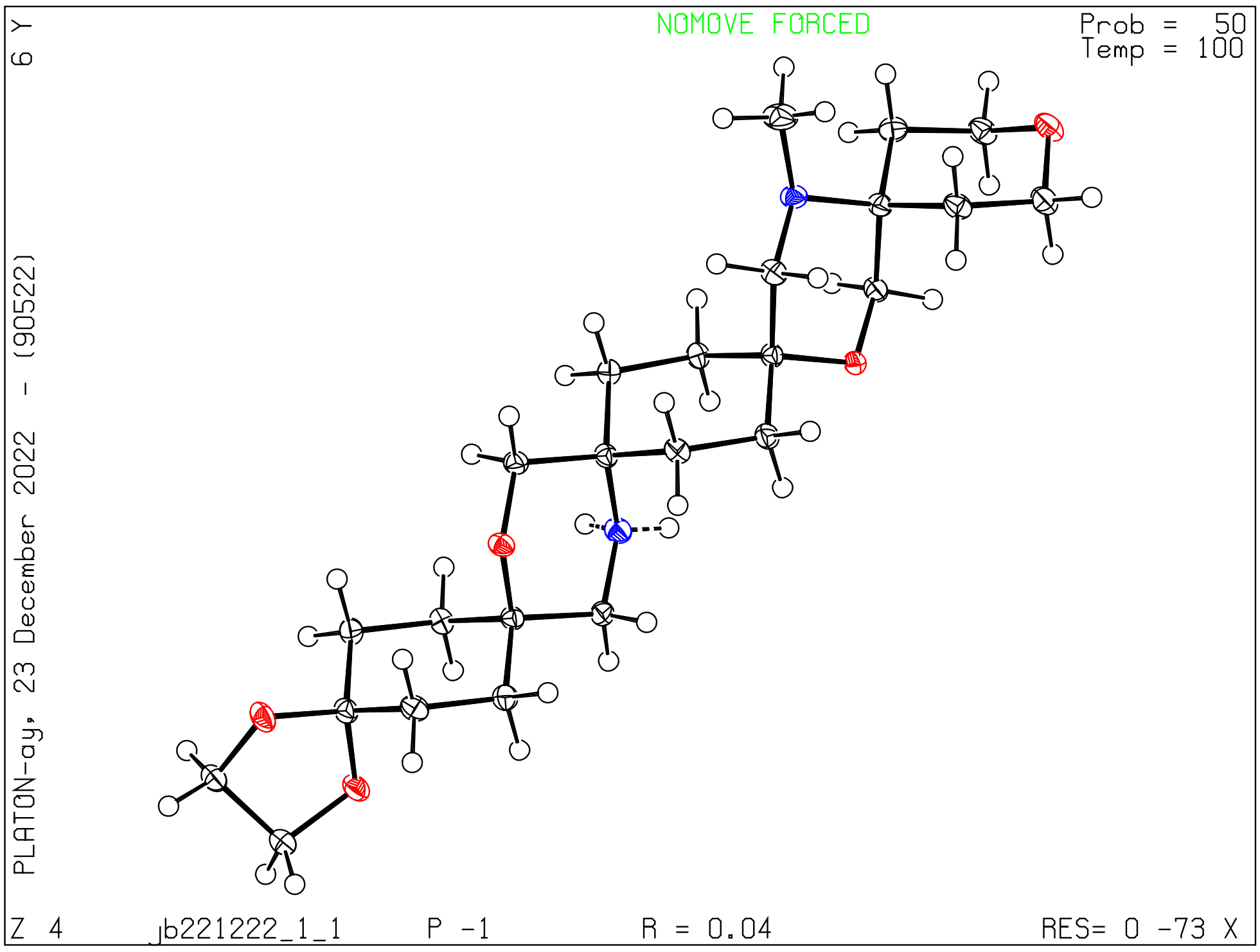

Single crystals of $C_{24}H_{40}N_2O_5$ **[jb221222_1_1]** were **grown by slow evaporation at rt from EtOAc**. A suitable crystal was selected and **measured** on a **XtaLAB Synergy, Dualflex, Pilatus 200K** diffractometer. The crystal was kept at 100.0(1) K during data collection.

Crystal Data for $C_{24}H_{40}N_2O_5$ (M =436.58 g/mol): triclinic, space group P-1 (no. 2), a = 7.7891(2) Å, b = 9.8085(3) Å, c = 14.6727(3) Å, α = 85.710(2)°, β = 80.397(2)°, γ = 85.235(2)°, V = 1099.34(5) Å^3, Z = 2, T = 100.0(1) K, μ(Cu Kα) = 0.737 mm^{-1}, Dcalc = 1.319 g/cm^3, 33317 reflections measured (9.064° ≤ 2Θ ≤ 159.268°), 4702 unique (R_{int} = 0.0296, R_{sigma} = 0.0167) which were used in all calculations. The final R_1 was 0.0353 (I > 2σ(I)) and wR_2 was 0.1002 (all data).

Comment: The NH protons are disordered about the −1 special position. Therefore, their occupancies must be constrained to 50% per orientation. There are no H-acceptors in proximity, rendering this distribution reasonable.

Compound 17b (deposition number 2557953)

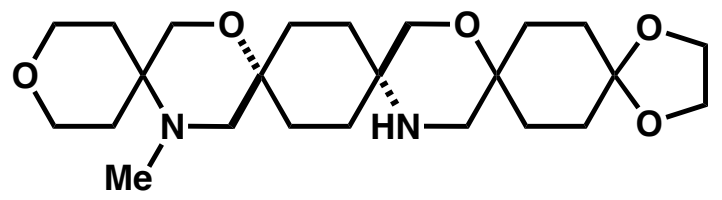

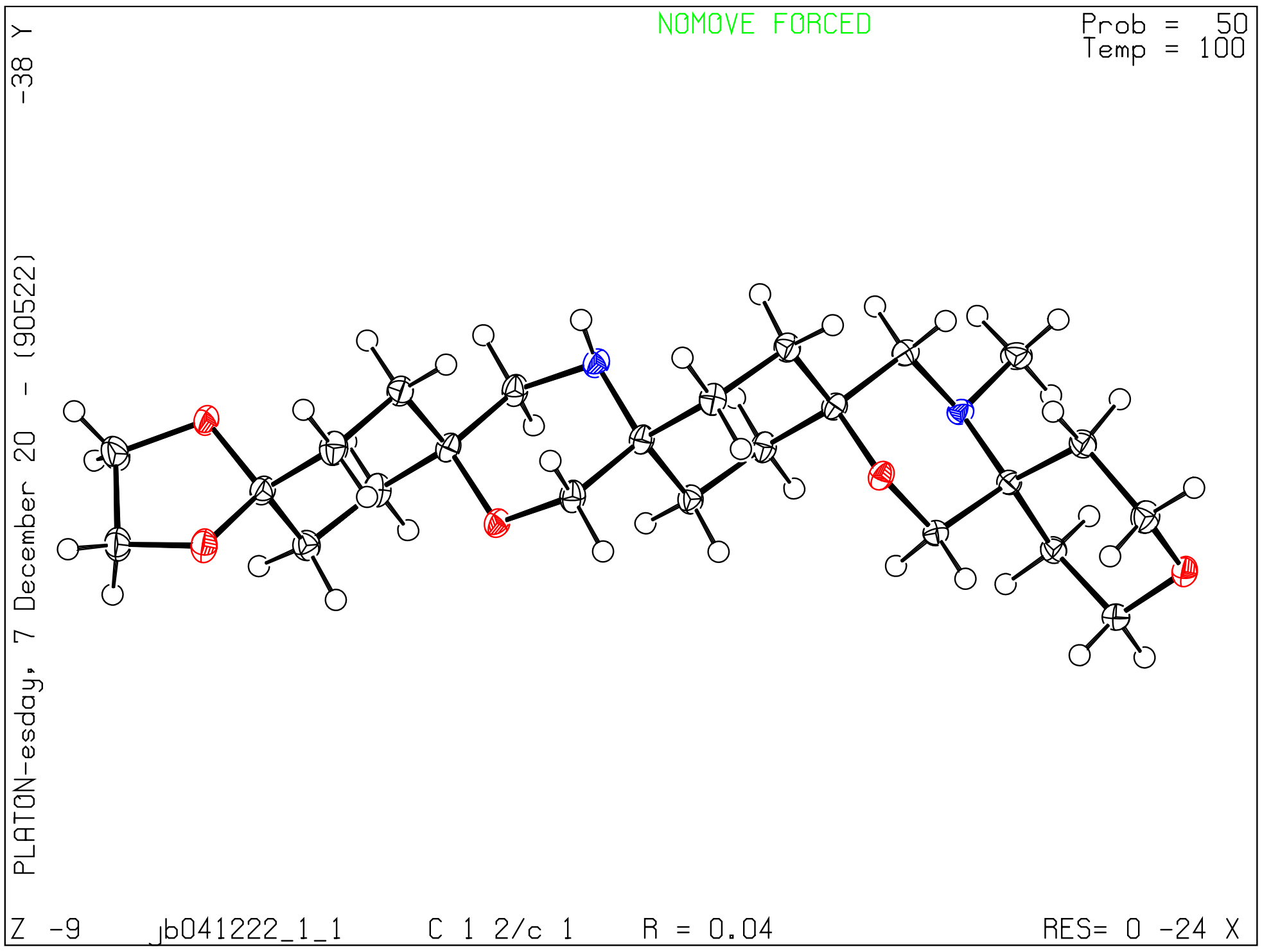

Single crystals of $C_{24}H_{40}N_2O_5$ **[jb041222_1_1]** were **grown by slow evaporation at rt from EtOAc**. A suitable crystal was selected and **measured** on a **XtaLAB Synergy, Dualflex, Pilatus 200K** diffractometer. The crystal was kept at 100.0(1) K during data collection.

Crystal Data for $C_{24}H_{40}N_2O_5$ (M =436.58 g/mol): monoclinic, space group C2/c (no. 15), a = 31.3299(3) Å, b = 6.24090(10) Å, c = 22.9427(2) Å, β = 93.4350(10)°, V = 4477.85(9) Å^3, Z = 8, T = 100.0(1) K, μ(Cu Kα) = 0.724 mm^{-1}, Dcalc = 1.295 g/cm^3, 45907 reflections measured (7.72° ≤ 2Θ ≤ 159.696°), 4791 unique (R_{int} = 0.0376, R_{sigma} = 0.0184) which were used in all calculations. The final R_1 was 0.0364 (I > 2σ(I)) and wR_2 was 0.0988 (all data).

Compound 57a (deposition number 2557955)

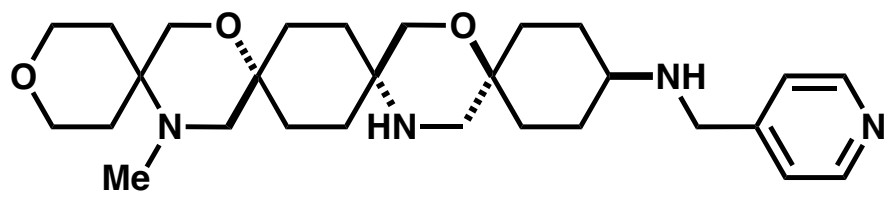

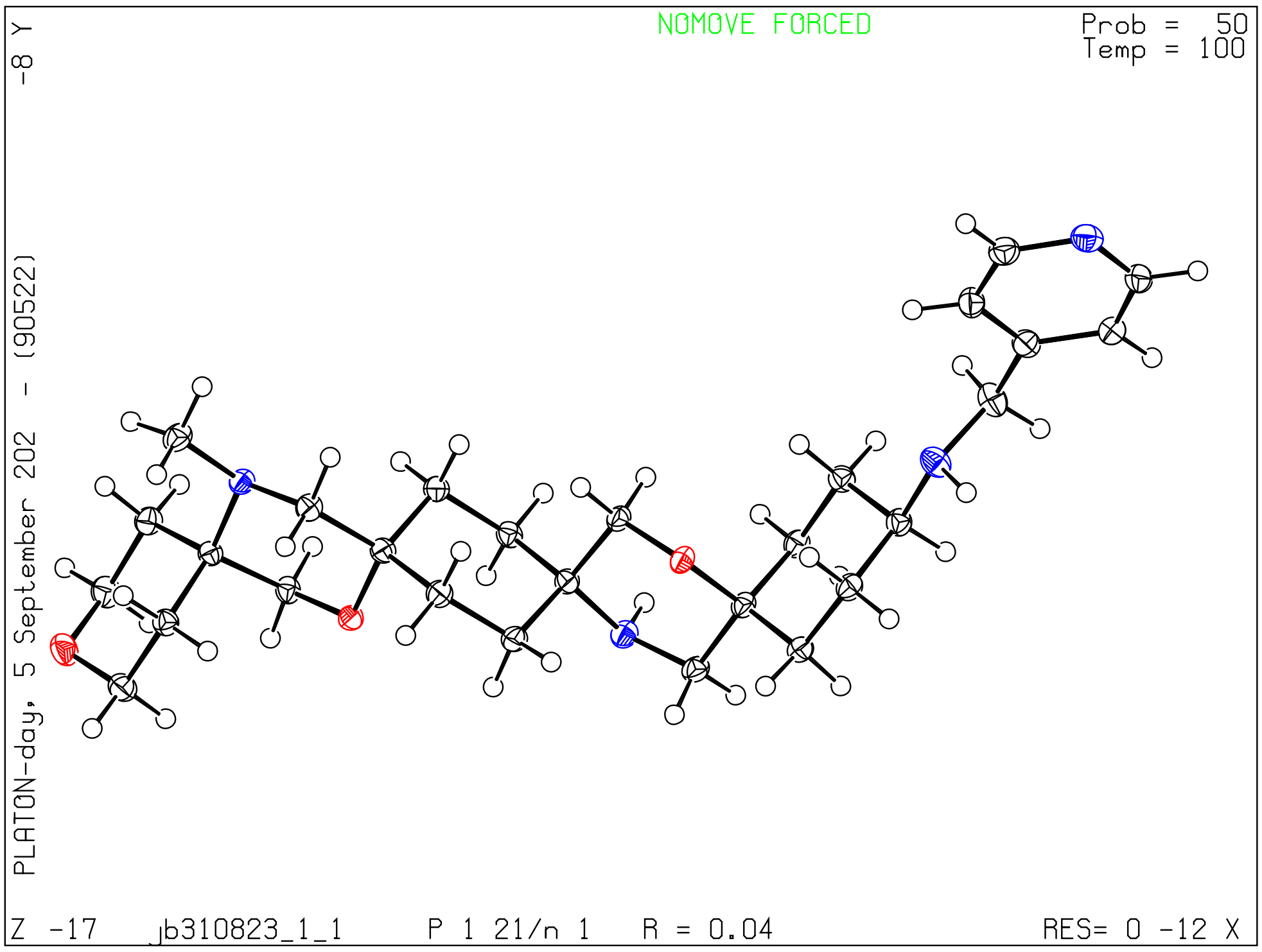

Single crystals of $C_{28}H_{44}N_4O_3$ **[jb310823_1_1]** were **grown by slow evaporation at rt from EtOAc**. A suitable crystal was selected and **measured** on a **XtaLAB Synergy, Dualflex, Pilatus 300K** diffractometer. The crystal was kept at 100.0(1) K during data collection.

Crystal Data for $C_{28}H_{44}N_4O_3$ (M =484.67 g/mol): monoclinic, space group P2$_1$/n (no. 14), a = 14.19890(10) Å, b = 10.76000(10) Å, c = 17.1200(2) Å, β = 104.5580(10)°, V = 2531.62(4) Å^3, Z = 4, T = 100.0(1) K, μ(Cu Kα) = 0.656 mm^{-1}, Dcalc = 1.272 g/cm^3, 77798 reflections measured (7.252° ≤ 2Θ ≤ 159.852°), 5333 unique (R_{int} = 0.0389, R_{sigma} = 0.0160) which were used in all calculations. The final R_1 was 0.0366 (I > 2σ(I)) and wR_2 was 0.0958 (all data).

Compound 57b (deposition number 2557956)

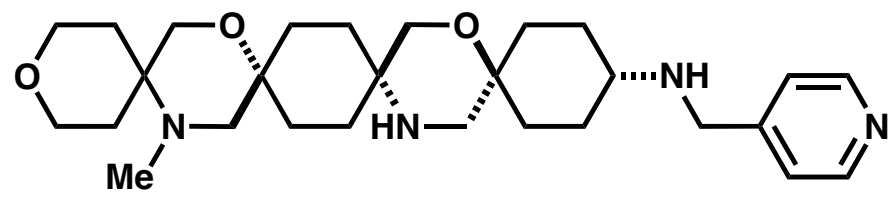

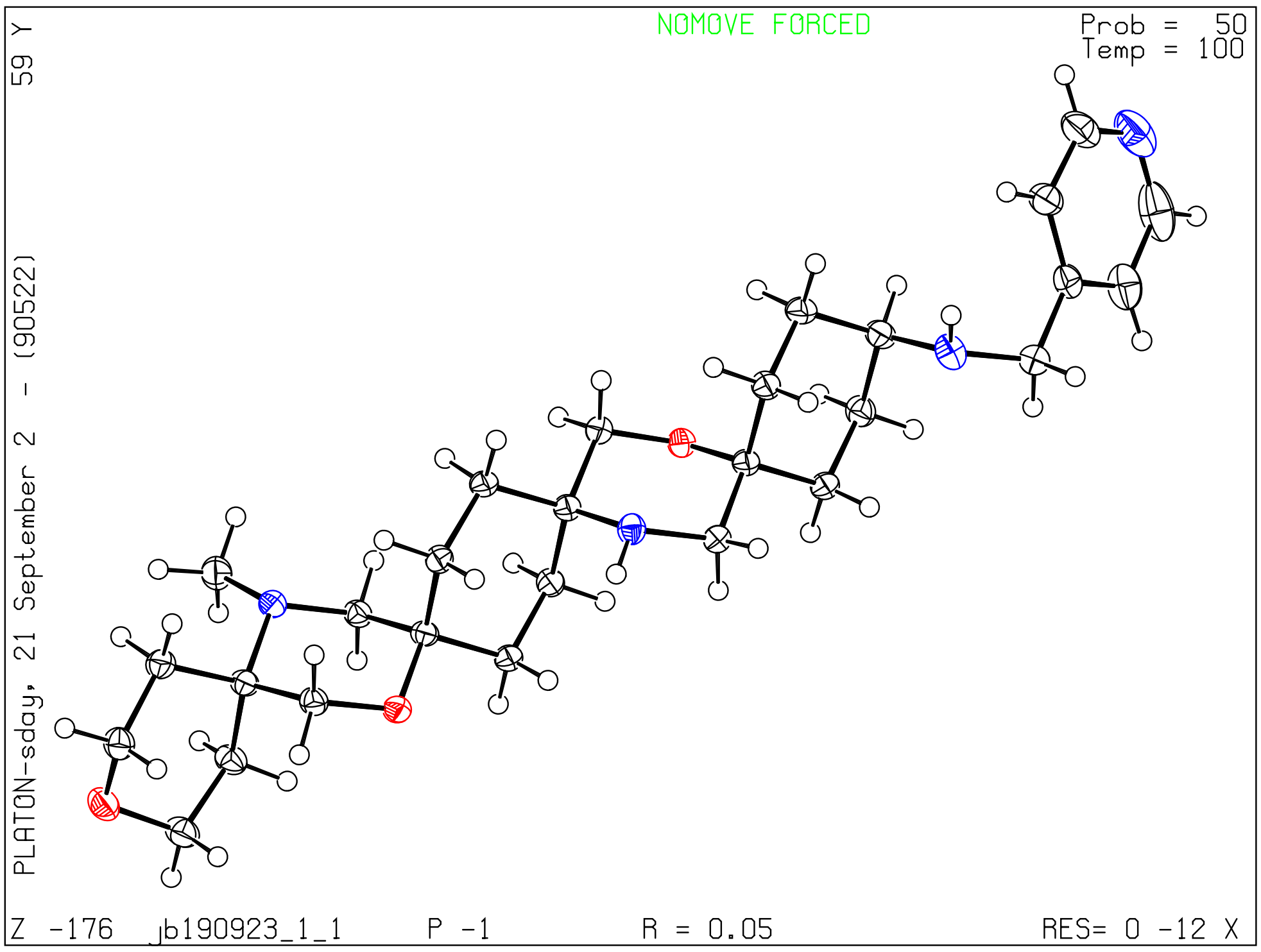

Single crystals of $C_{28}H_{44}N_4O_3$ **[jb190923_1_1]** were **grown by slow evaporation at rt from EtOAc**. A suitable crystal was selected and **measured** on a **XtaLAB Synergy, Dualflex, Pilatus 300K** diffractometer. The crystal was kept at 100.0(1) K during data collection.

Crystal Data for $C_{28}H_{44}N_4O_3$ (M =484.67 g/mol): triclinic, space group P-1 (no. 2), a = 6.85240(10) Å, b = 11.7365(2) Å, c = 16.8337(4) Å, α = 70.617(2)°, β = 84.671(2)°, γ = 77.168(2)°, V = 1244.92(4) Å^3, Z = 2, T = 100.0(1) K, μ(Cu Kα) = 0.667 mm^{-1}, Dcalc = 1.293 g/cm^3, 37046 reflections measured (8.156° ≤ 2Θ ≤ 159.666°), 4986 unique (R_{int} = 0.0588, R_{sigma} = 0.0264) which were used in all calculations. The final R_1 was 0.0463 (I > 2σ(I)) and wR_2 was 0.1388 (all data).

Comments: The proton on N2 is disordered over two positions with a ratio of 50:50 and the proton on N3 is disordered over two positions 65:35. There are no H-acceptors in proximity, rendering these distributions reasonable. The pyridyl group rotates a tiny bit.

Compound 57c (deposition number 2557957)

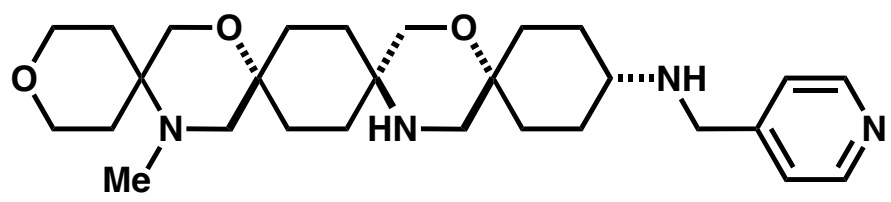

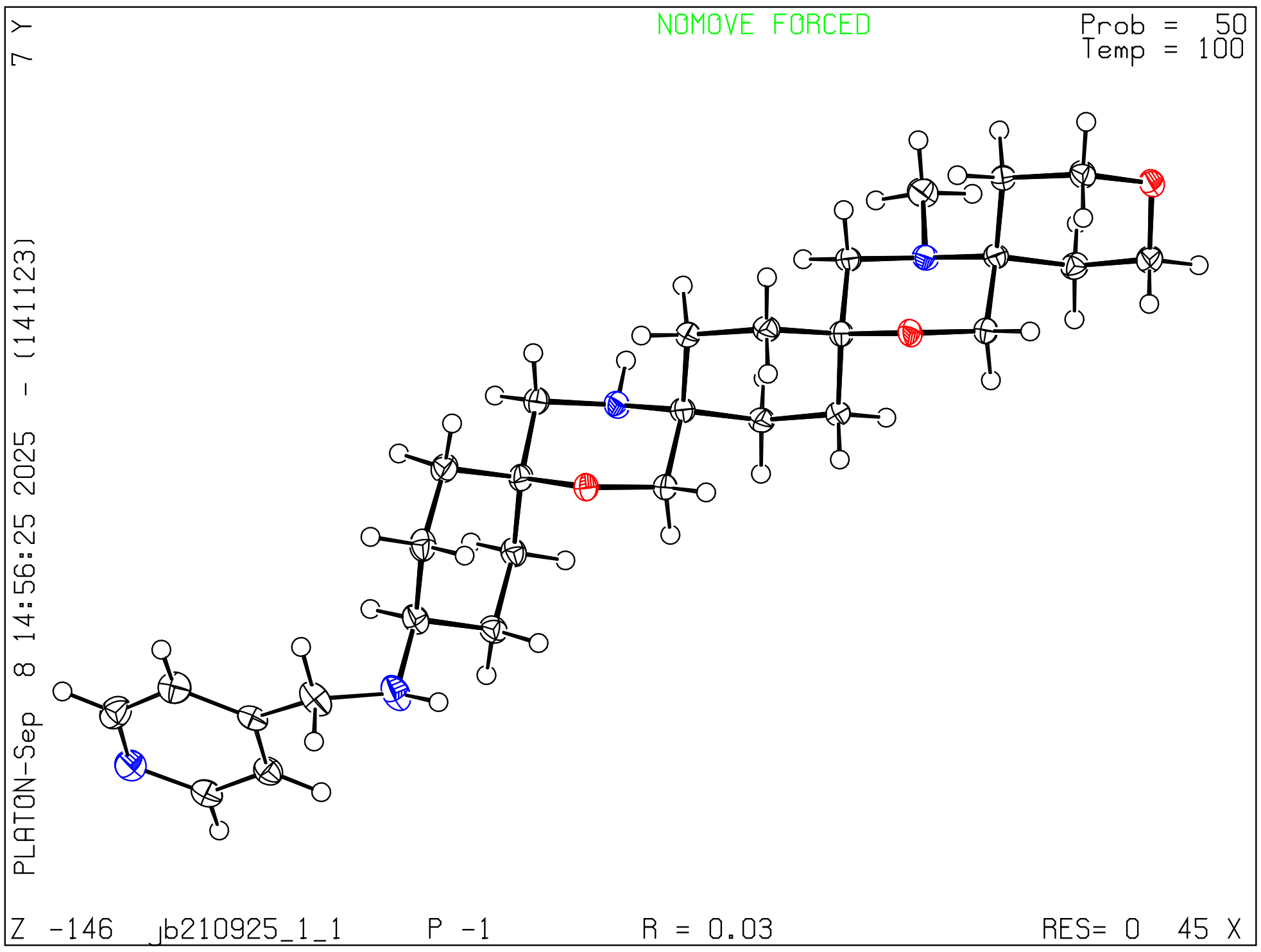

Single crystals of $C_{28}H_{44}N_4O_3$ **[jb210925_1_1]** were **grown by slow evaporation at rt from EtOAc**. A suitable crystal was selected and **measured** on a **XtaLAB Synergy R, HyPix-Arc 150** diffractometer. The crystal was kept at 100.0(1) K during data collection.

Crystal Data for $C_{28}H_{44}N_4O_3$ (M =484.67 g/mol): triclinic, space group P-1 (no. 2), a = 6.68330(10) Å, b = 14.0468(2) Å, c = 14.6785(2) Å, α = 68.9090(10)°, β = 87.8700(10)°, γ = 78.6150(10)°, V = 1259.53(3) Å^3, Z = 2, T = 100.0(1) K, μ(Cu Kα) = 0.659 mm^{-1}, Dcalc = 1.278 g/cm^3, 49786 reflections measured (6.458° ≤ 2Θ ≤ 148.624°), 4892 unique (R_{int} = 0.0245, R_{sigma} = 0.0128) which were used in all calculations. The final R_1 was 0.0339 (I > 2σ(I)) and wR_2 was 0.0915 (all data).

Comment: The aminomethylpyridine is disordered with a 70:30 occupancy.

Compound 57d (deposition number 2557958)

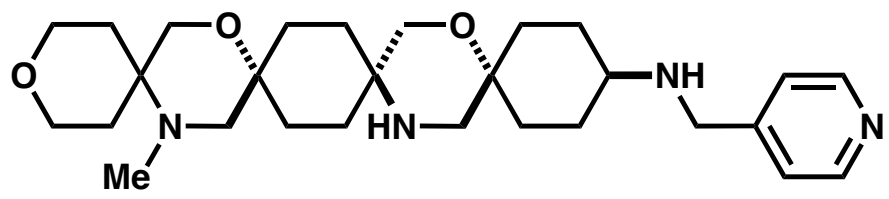

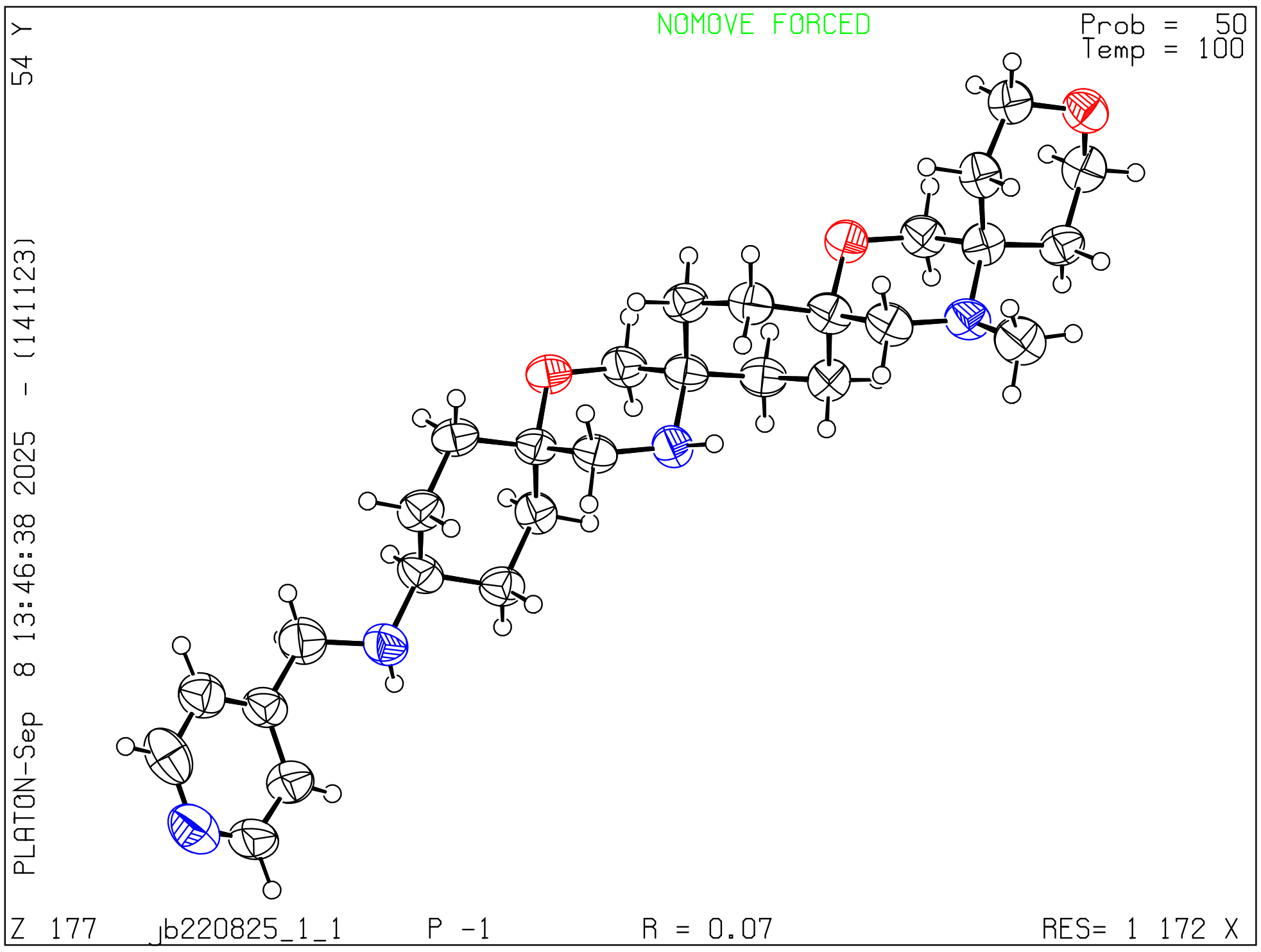

Single crystals of $C_{28}H_{52}N_4O_7$ **[jb220825_1_1]** were **grown by slow evaporation at rt from EtOAc**. A suitable crystal was selected and **measured** on a **XtaLAB Synergy R, HyPix-Arc 150** diffractometer. The crystal was kept at 100.0(1) K during data collection.

Crystal Data for $C_{28}H_{52}N_4O_7$ (M =556.73 g/mol): triclinic, space group P-1 (no. 2), a = 6.7167(5) Å, b = 13.3369(6) Å, c = 16.9845(8) Å, α = 70.988(4)°, β = 87.368(5)°, γ = 86.763(5)°, V = 1435.57(15) Å^3, Z = 2, T = 100.0(1) K, μ(Cu Kα) = 0.748 mm^{-1}, Dcalc = 1.288 g/cm^3, 12857 reflections measured (5.506° ≤ 2Θ ≤ 102.766°), 2886 unique (R_{int} = 0.0537, R_{sigma} = 0.0488) which were used in all calculations. The final R_1 was 0.0734 (I > 2σ(I)) and wR_2 was 0.2395 (all data).

Comment: Extremely thin and weakly diffracting sample (ellipsoids indicate reduced occupancy of water molecules and disorder). This was the best resolution that could be achieved before radiation damage sets in.

Compound 58c (deposition number 2557959)

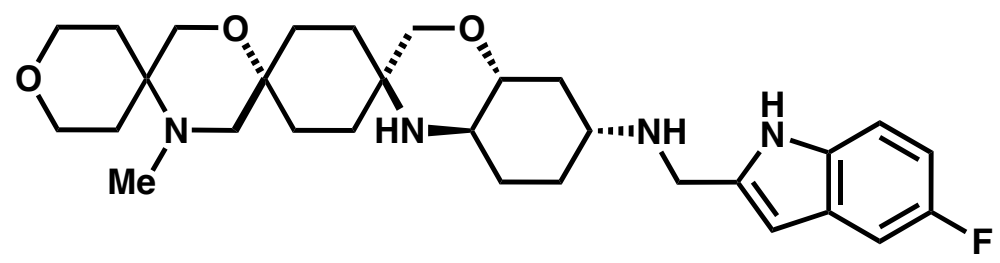

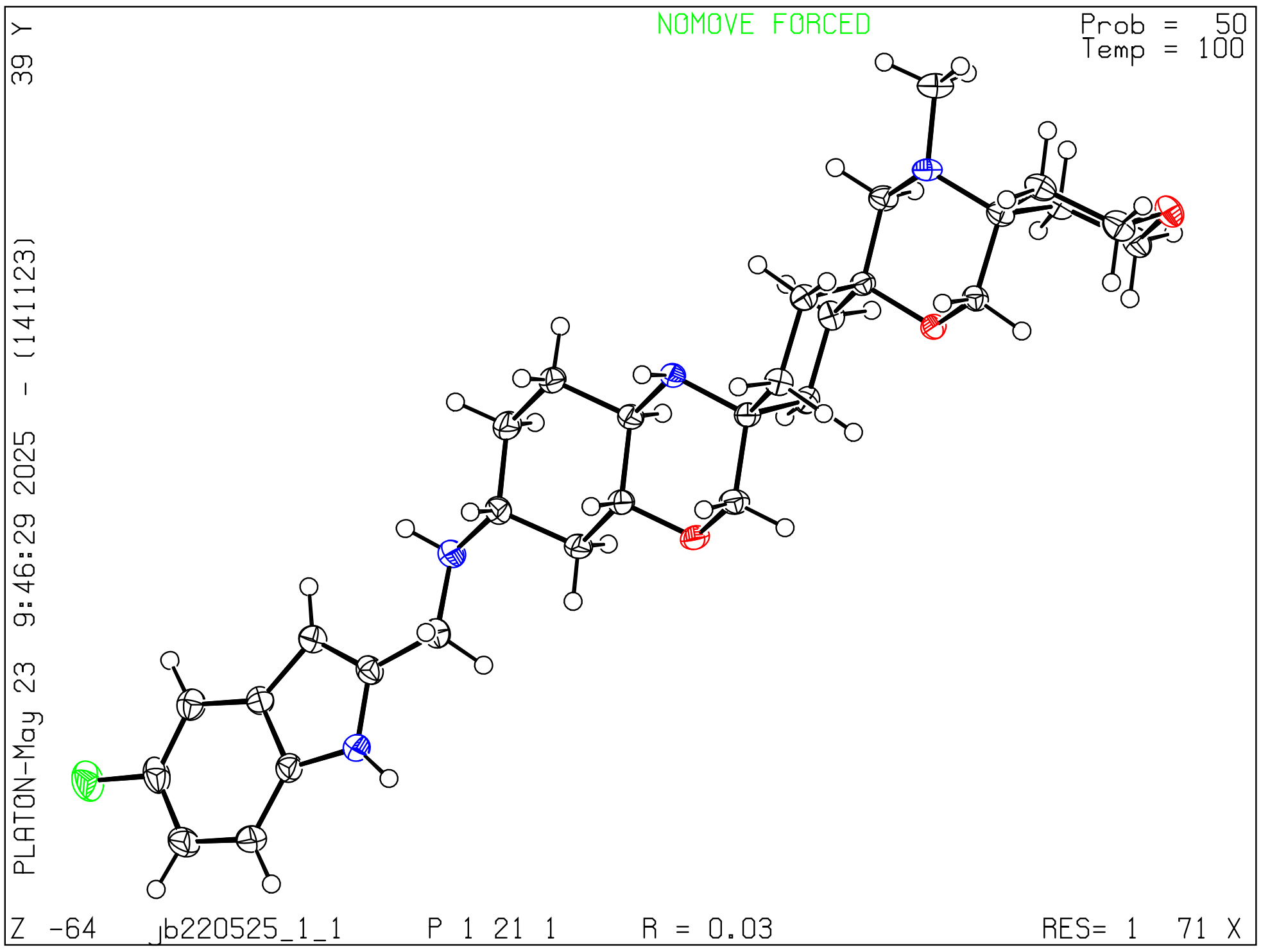

Single crystals of $C_{30}H_{45}FN_4O_4$ **[jb220525_1_1]** were **grown by layer diffusion with the solvent *i*PrOH and antisolvent cyclohexane**. A suitable crystal was selected and **measured** on a **XtaLAB Synergy R, HyPix-Arc 150** diffractometer.

Crystal Data for $C_{30}H_{45}FN_4O_4$ (M =544.70 g/mol): monoclinic, space group P2$_1$ (no. 4), a = 9.56700(10) Å, b = 6.13380(10) Å, c = 23.5917(3) Å, β = 98.1600(10)°, V = 1370.39(3) Å^3, Z = 2, T = 100.0(1) K, μ(Cu Kα) = 0.749 mm^{-1}, Dcalc = 1.320 g/cm^3, 39477 reflections measured (7.572° ≤ 2Θ ≤ 148.604°), 5337 unique (R_{int} = 0.0330, R_{sigma} = 0.0224) which were used in all calculations. The final R_1 was 0.0327 (I > 2σ(I)) and wR_2 was 0.0811 (all data).

6 References

1 Y. Liu, J. Wu, M. Zhou, W. Chen, D. Li, Z. Wang, B. Hornsperger, J. D. Aebi, H.-P. Märki, B. Kuhn, L. Wang, A. Kuglstatter, J. Benz, S. Müller, R. Hochstrasser, G. Ottaviani, J. Xin, S. Kirchner, S. Mohr, P. Verry, W. Riboulet, H. C. Shen, A. V Mayweg, K. Amrein and X. Tan, *J. Med. Chem.*, 2020, **63**, 6876–6897.

2 X. Chen, A. Murawski, K. Patel, C. L. Crespi and P. V Balimane, *Pharm. Res.*, 2008, **25**, 1511–1520.

3 H. Fischer, M. Ullah, C. C. de la Cruz, T. Hunsaker, C. Senn, T. Wirz, B. Wagner, D. Draganov, F. Vazvaei, M. Donzelli, A. Paehler, M. Merchant and L. Yu, *Neuro. Oncol.*, 2020, **22**, 819–829.

4 B. Wagner, H. Fischer, M. Kansy, A. Seelig and F. Assmus, *Eur. J. Pharm. Sci.*, 2015, **68**, 68–77.

5 M. R. Berthold, N. Cebron, F. Dill, T. R. Gabriel, T. Kötter, T. Meinl, P. Ohl, C. Sieb, K. Thiel and B. Wiswedel, in *Studies in Classification, Data Analysis, and Knowledge Organization (GfKL 2007)*, Springer, 2007.

6 P. Pracht, F. Bohle and S. Grimme, *Phys. Chem. Chem. Phys.*, 2020, **22**, 7169–7192.

7 S. Grimme, F. Bohle, A. Hansen, P. Pracht, S. Spicher and M. Stahn, *J. Phys. Chem. A*, 2021, **125**, 4039–4054.

8 F. Saito, N. Trapp and J. W. Bode, *J. Am. Chem. Soc.*, 2019, **141**, 5544–5554.

6 NMR SPECTRA OF INTERMEDIATES

Compound 2: ^{1}H NMR ($CDCl_3$) top, ^{13}C NMR ($CDCl_3$) bottom

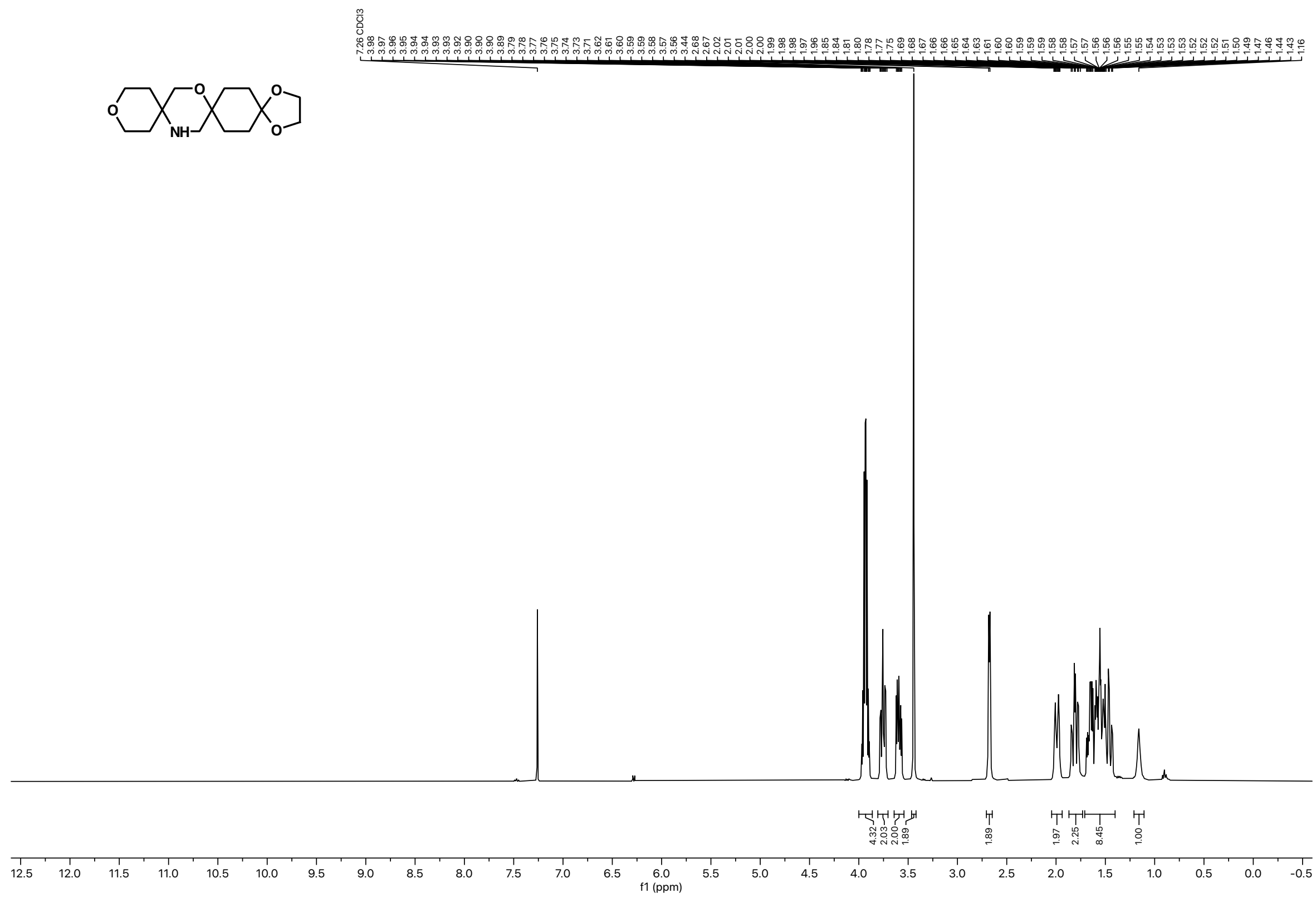

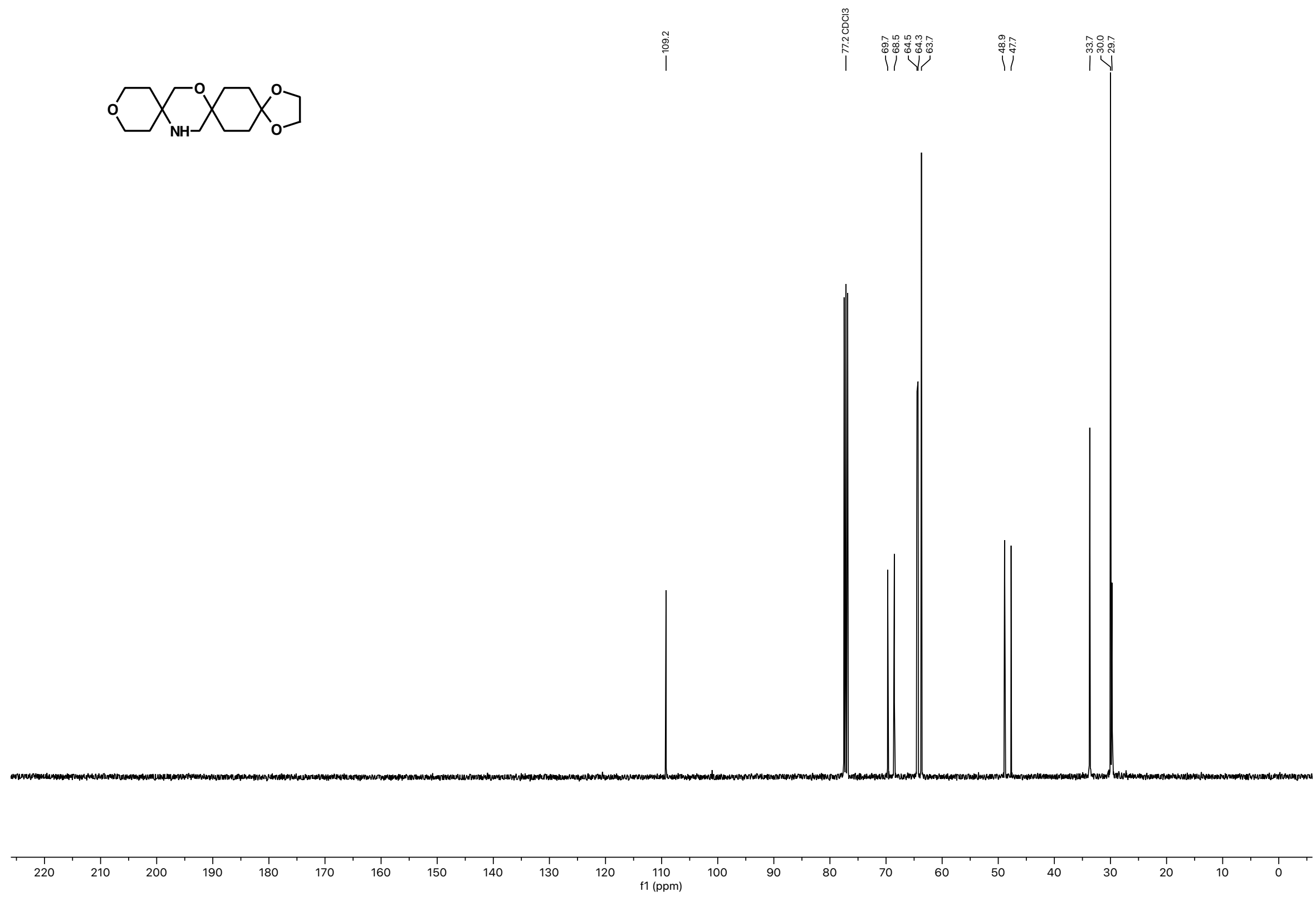

Compound 3: ¹H NMR ($CDCl_3$) top, ¹³C NMR ($CDCl_3$) bottom

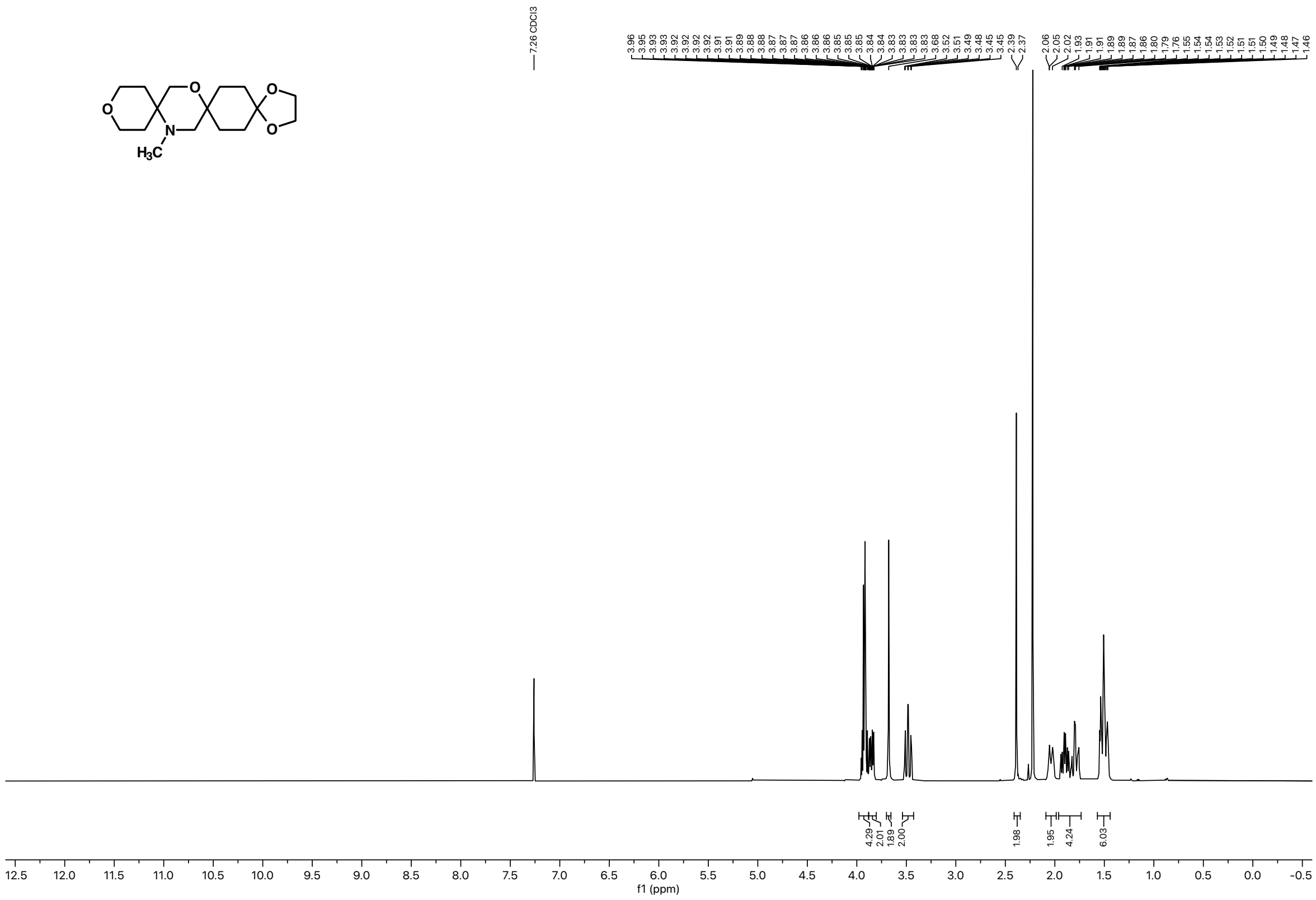

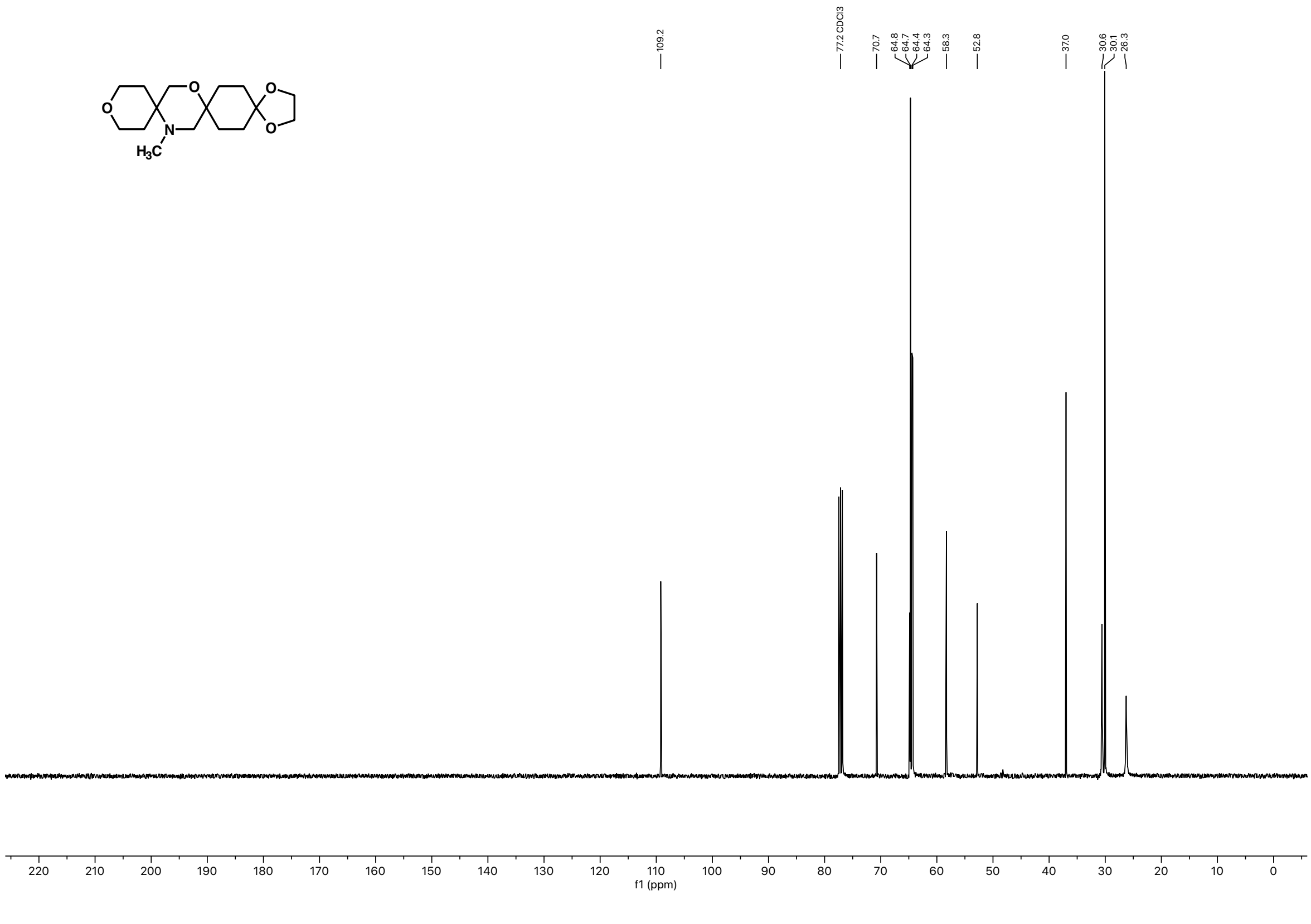

Compound 4: ^{1}H NMR ($CDCl_3$) top, ^{13}C NMR ($CDCl_3$) bottom

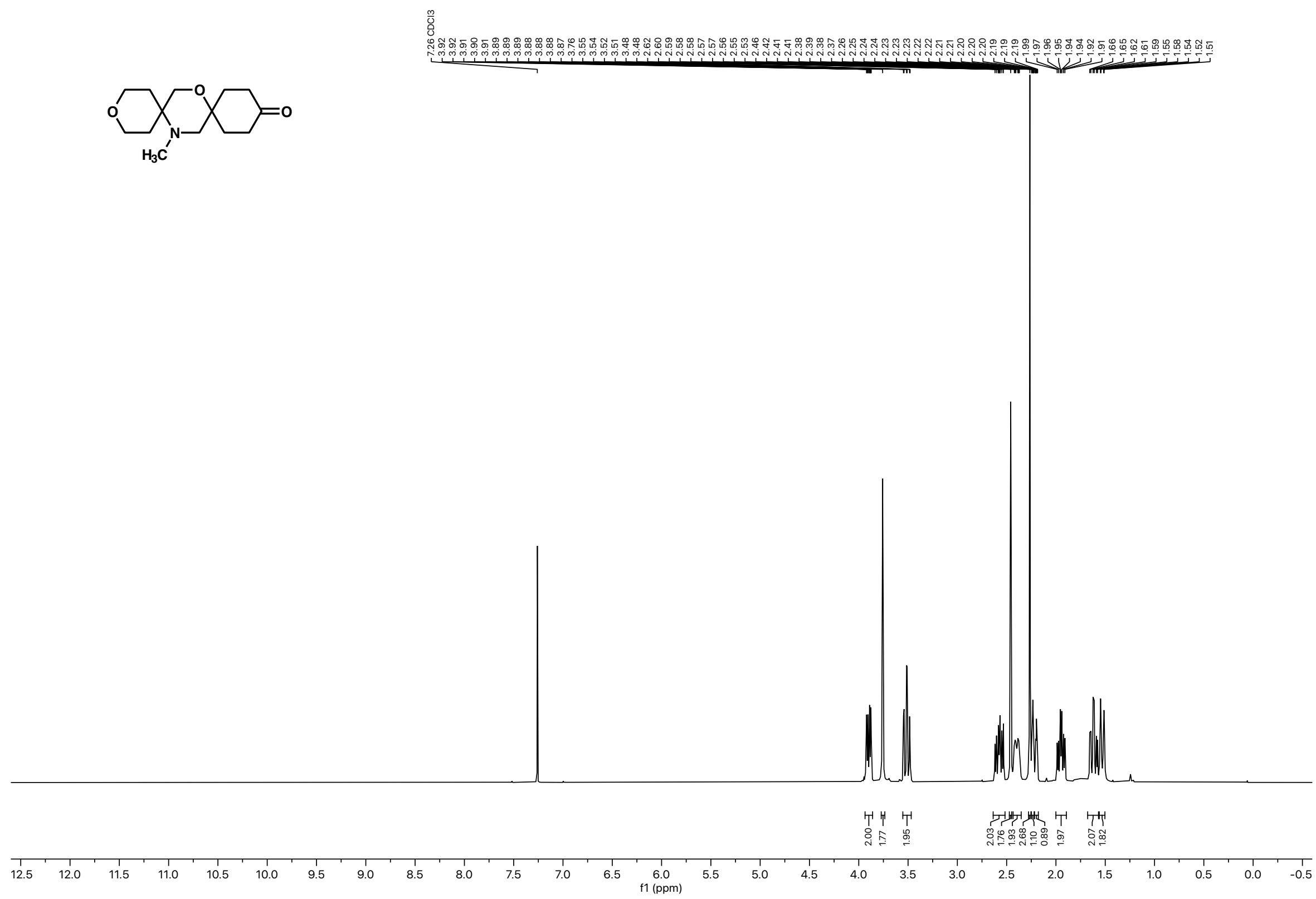

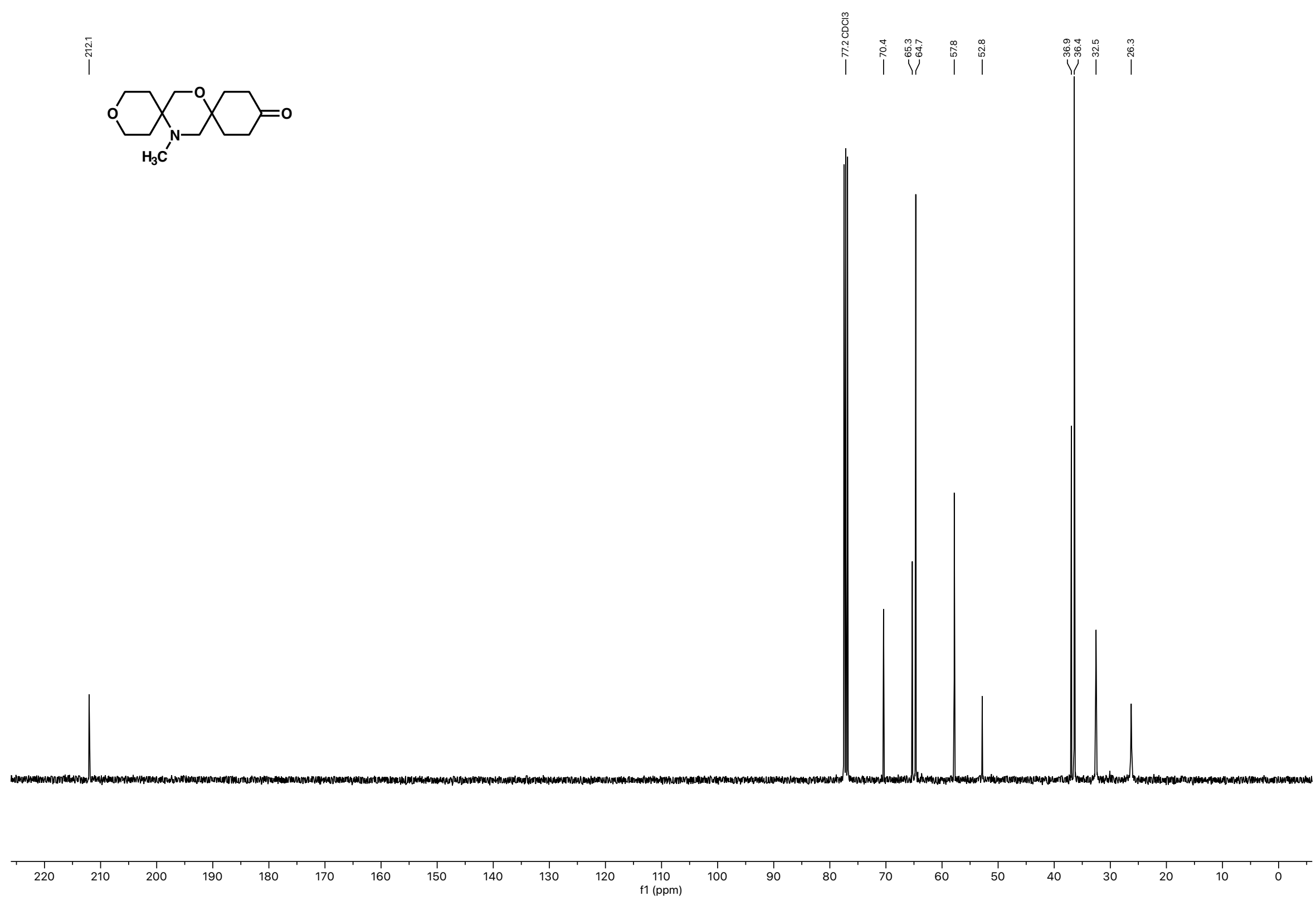

Compound 5: ^{1}H NMR (CDCl$_3$) top, ^{13}C NMR (CDCl$_3$) bottom

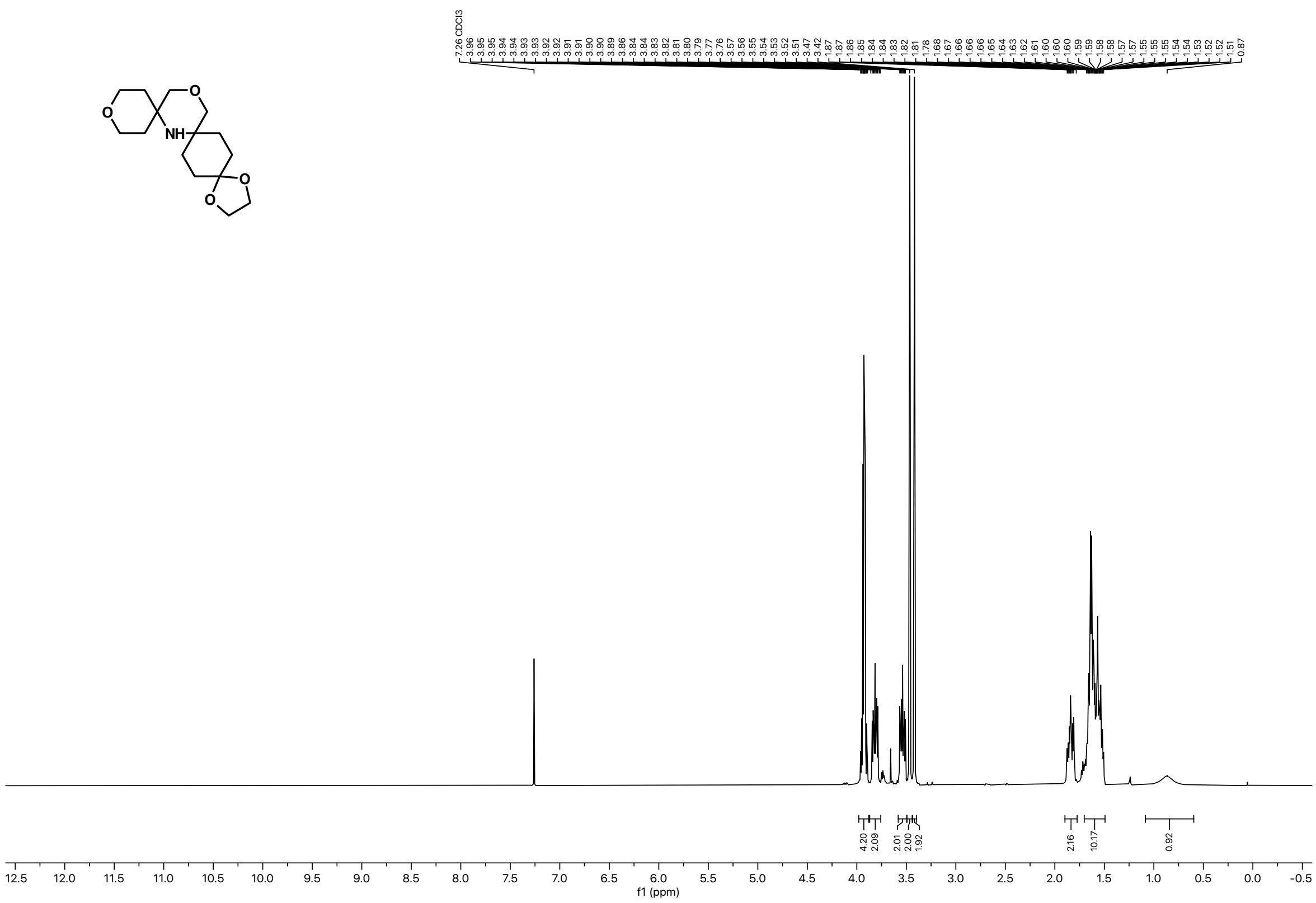

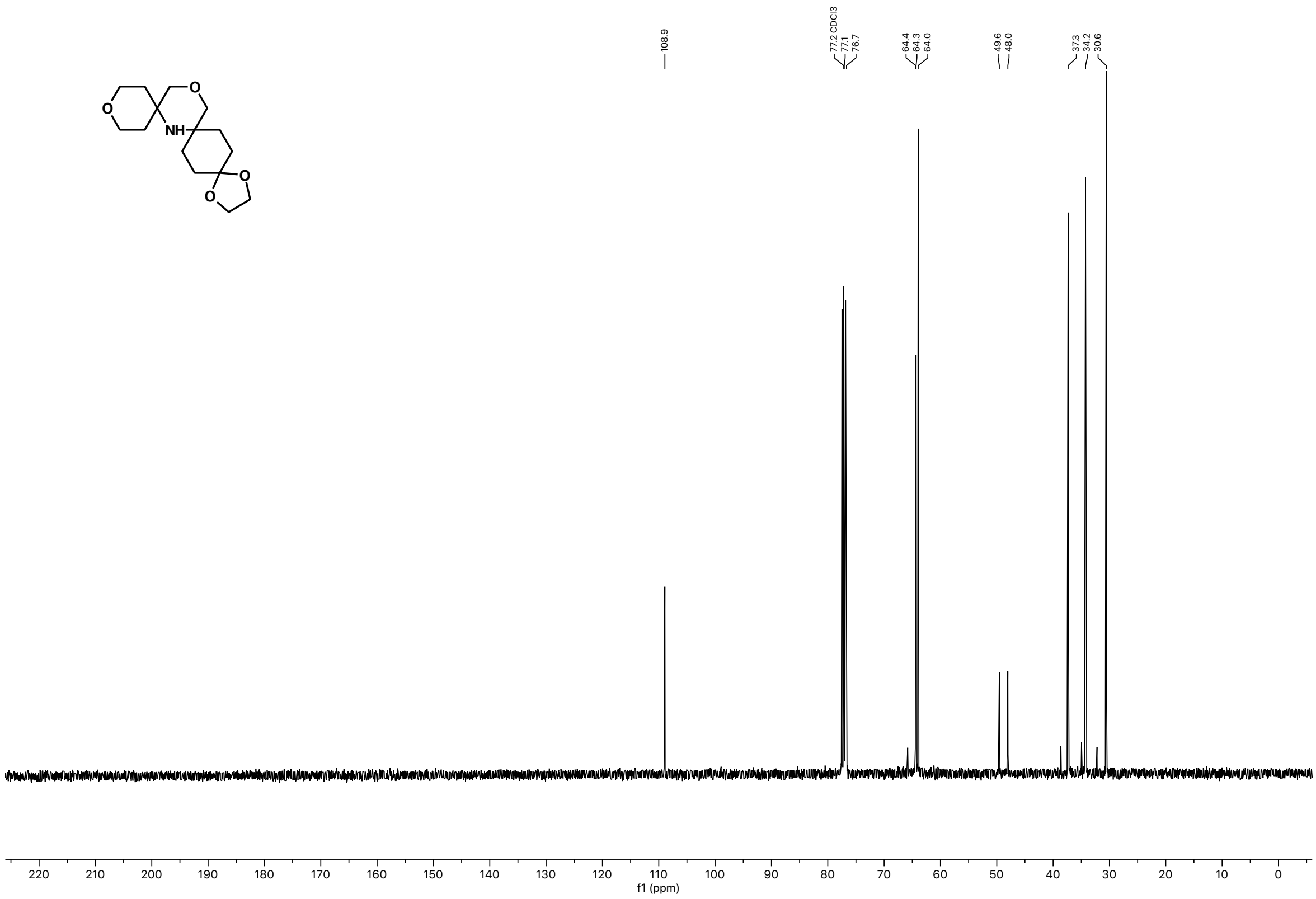

Compound 6: ^{1}H NMR ($CDCl_3$) top, ^{13}C NMR ($CDCl_3$) bottom

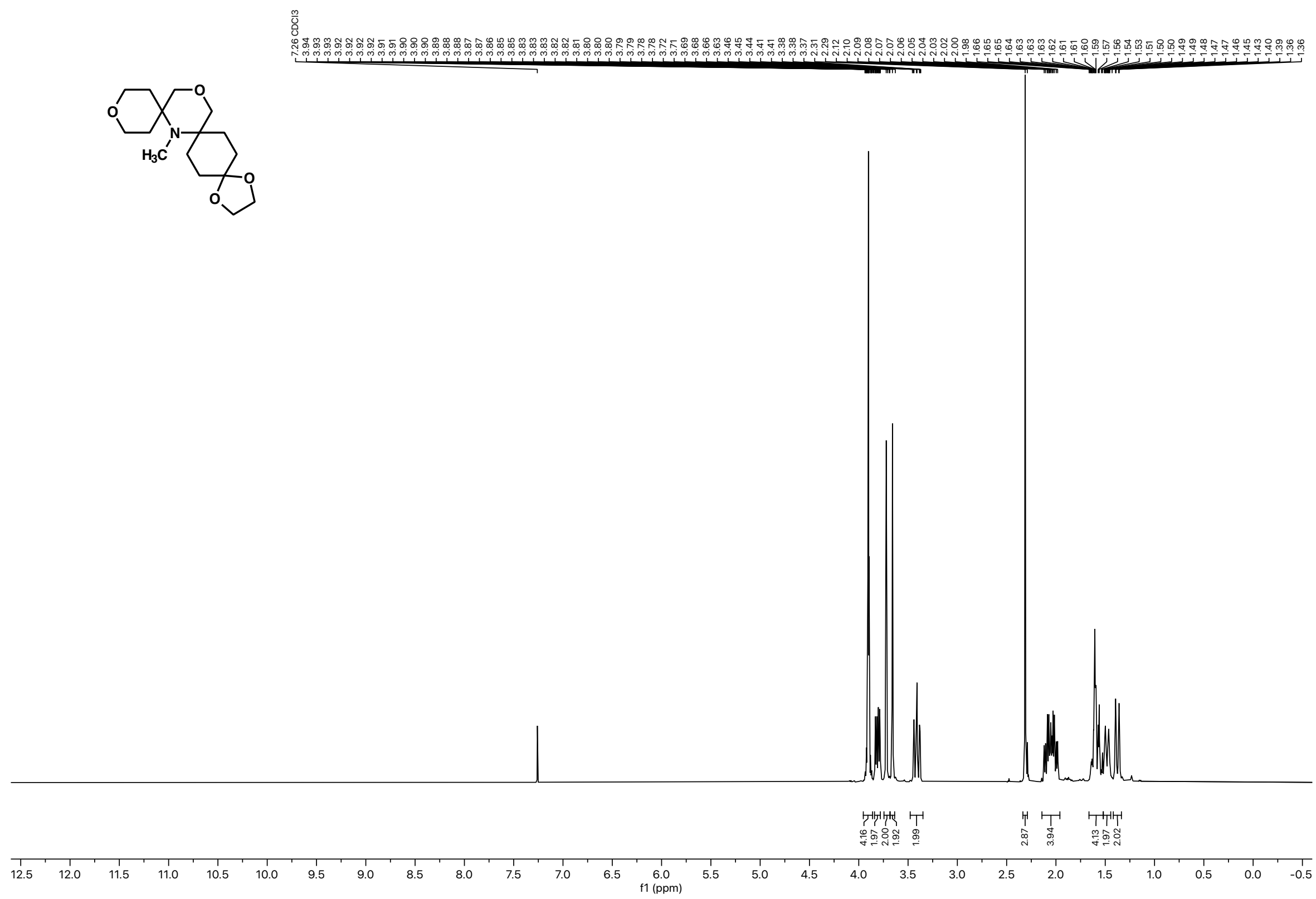

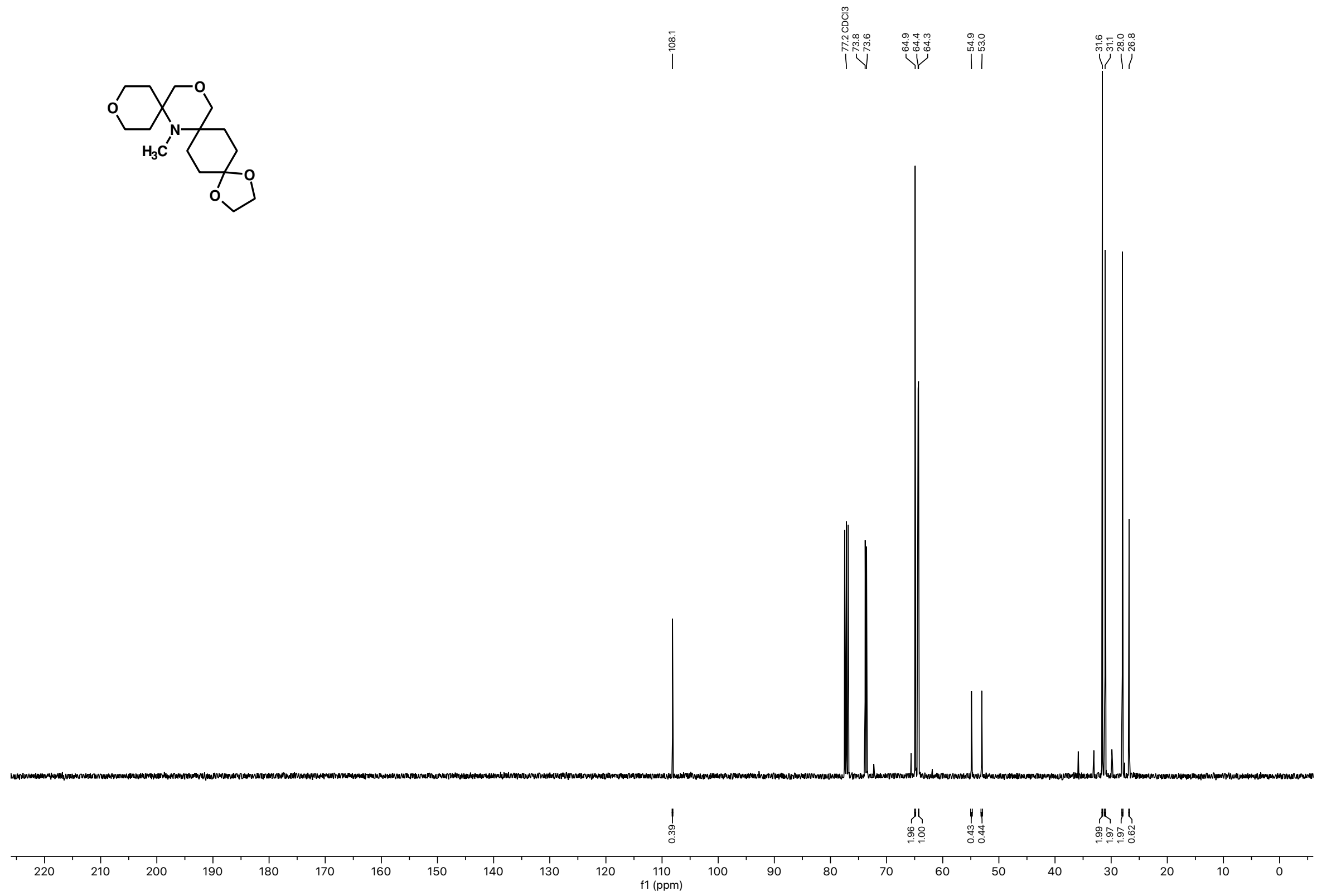

Compound 7: ¹H NMR ($CDCl_3$) top, ¹³C NMR ($CDCl_3$) bottom

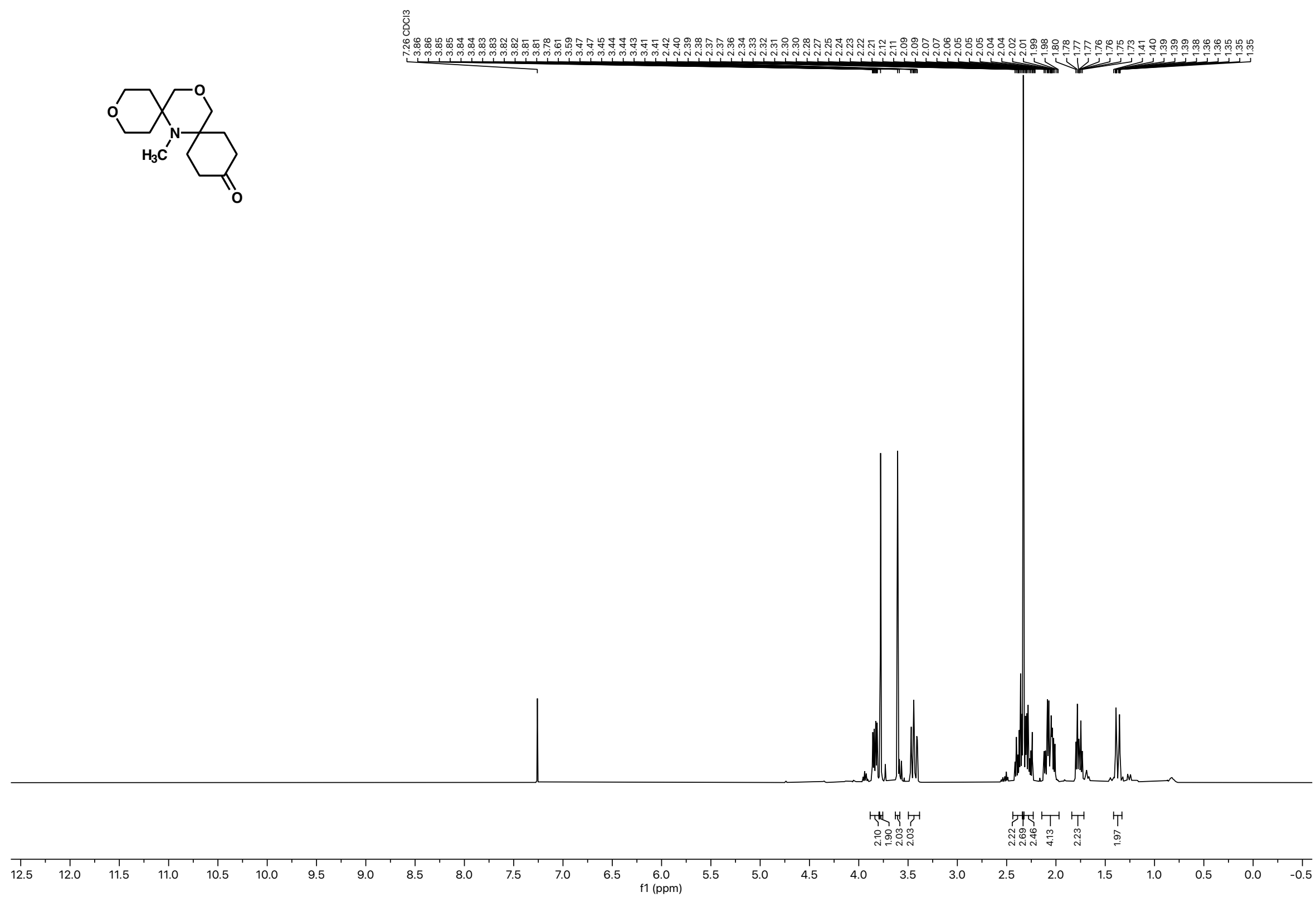

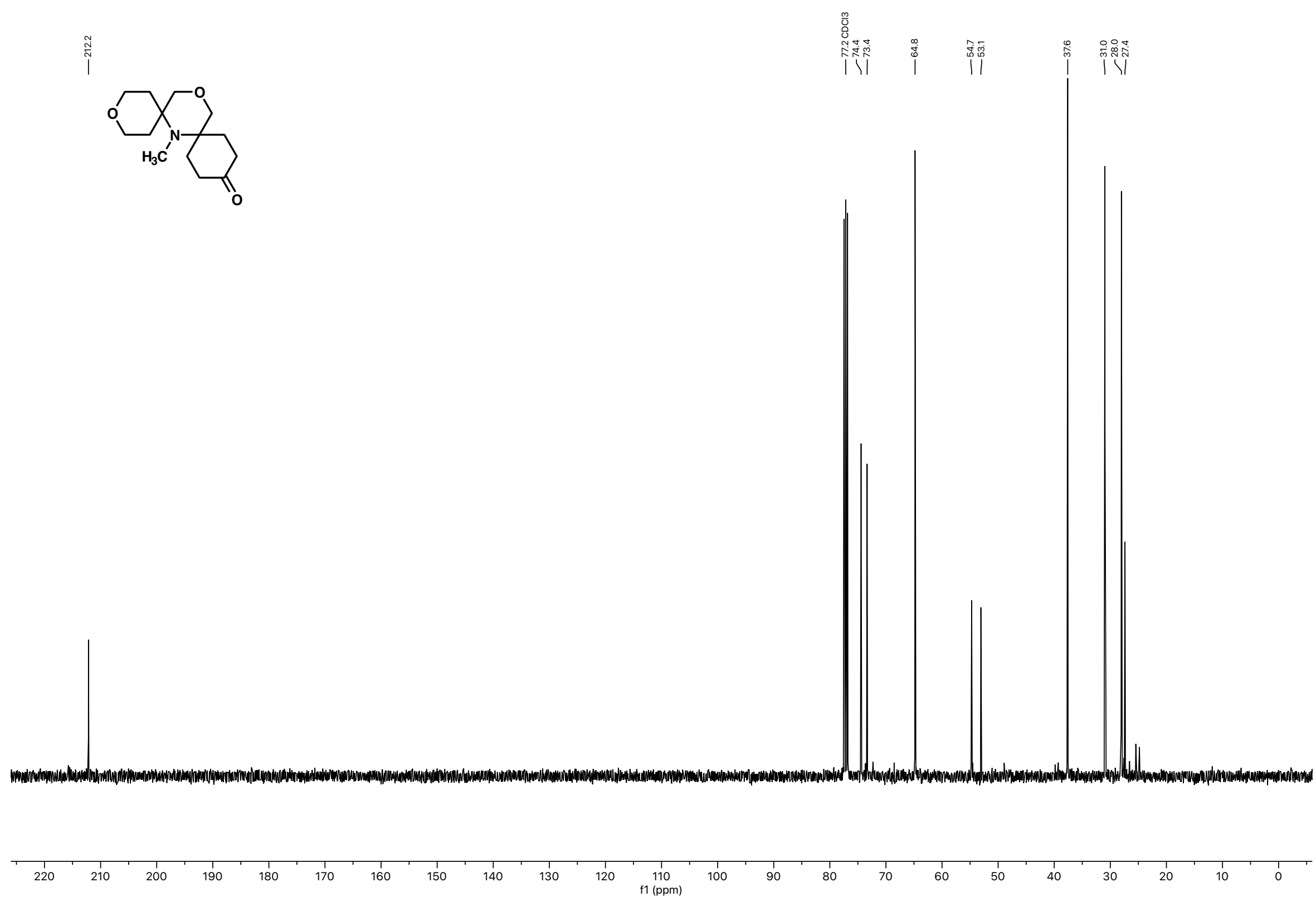

Compound 8: ^{1}H NMR ($CDCl_3$) top, ^{13}C NMR ($CDCl_3$) bottom

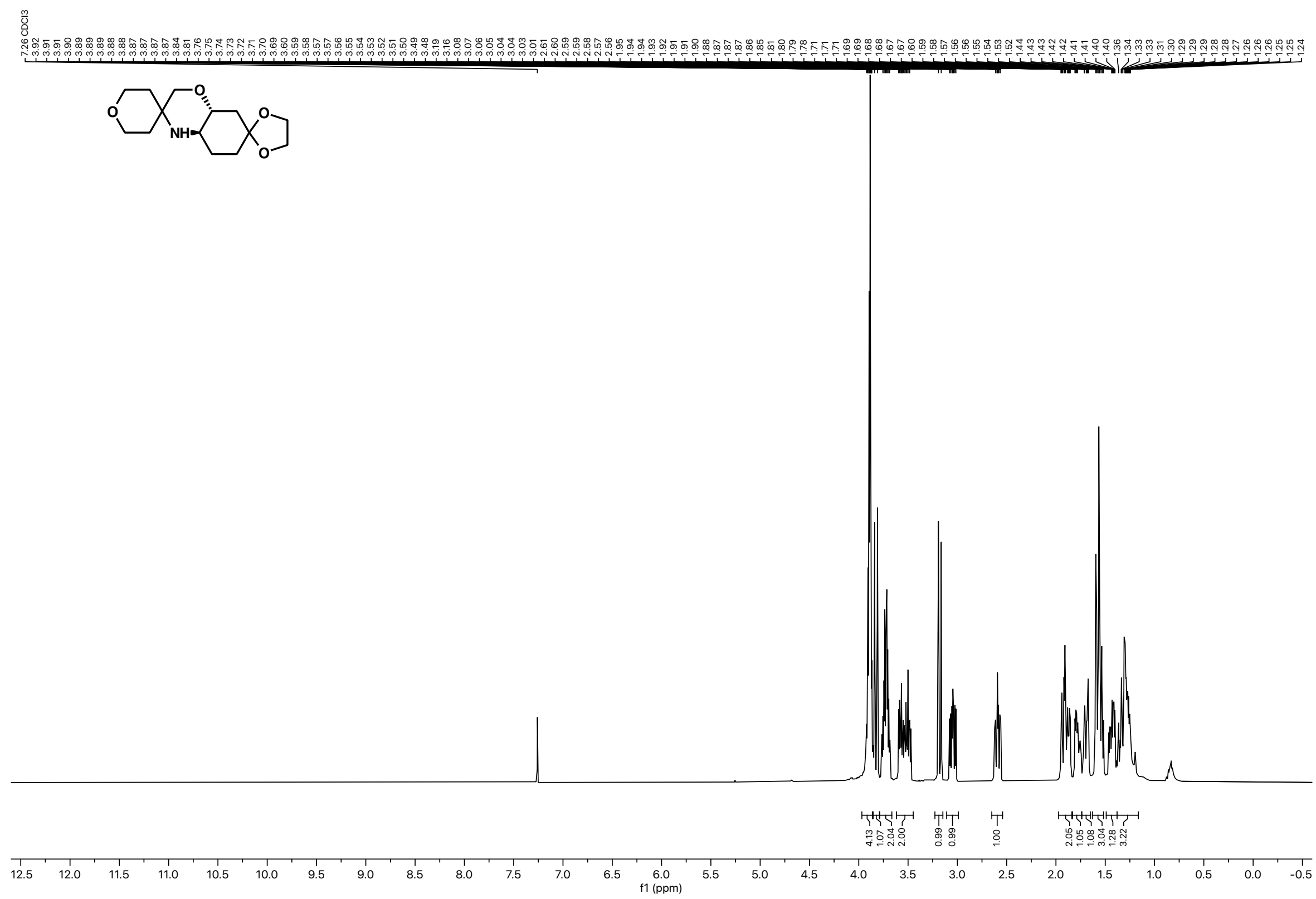

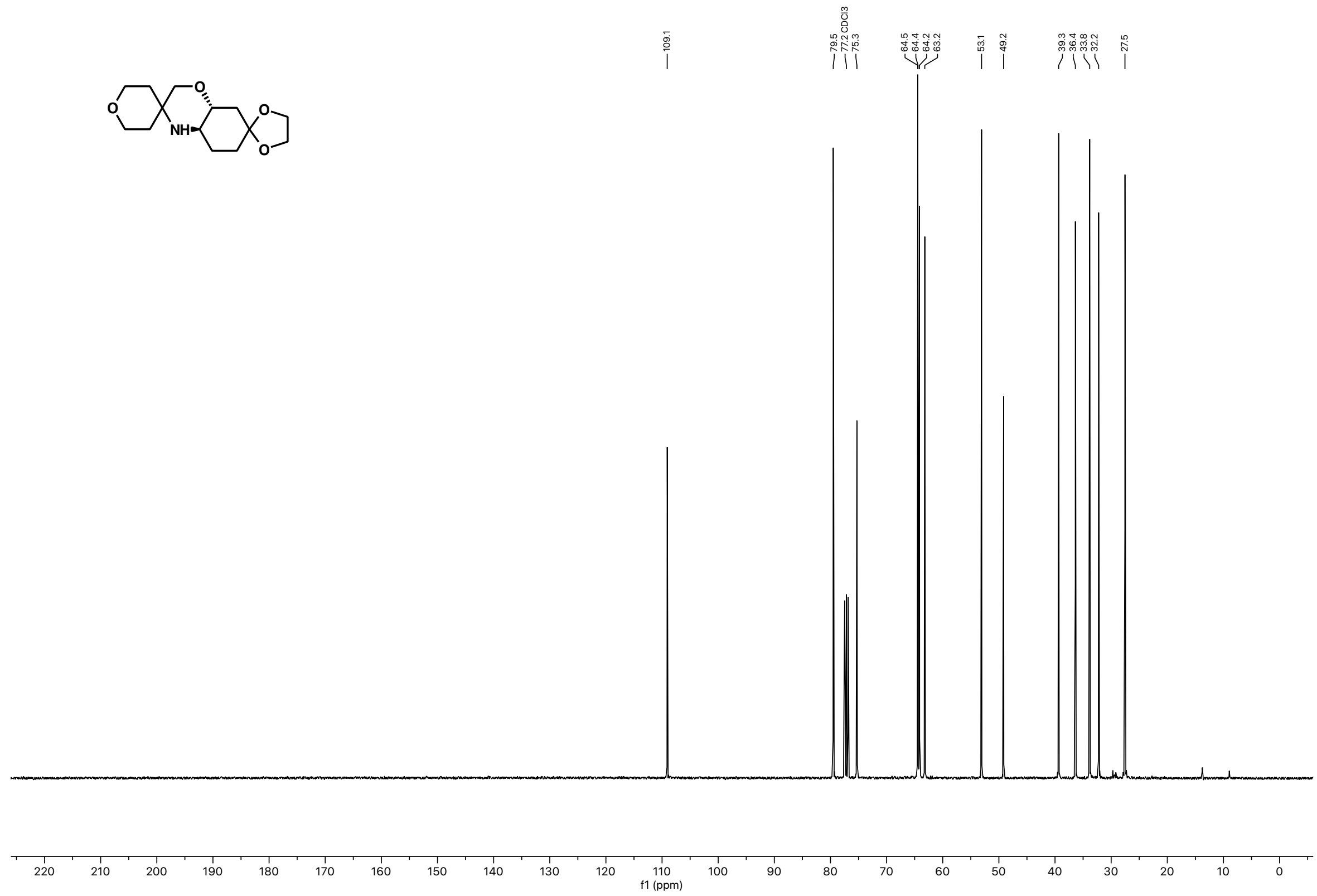

Compound 9: ^{1}H NMR ($CDCl_3$) top, ^{13}C NMR ($CDCl_3$) bottom

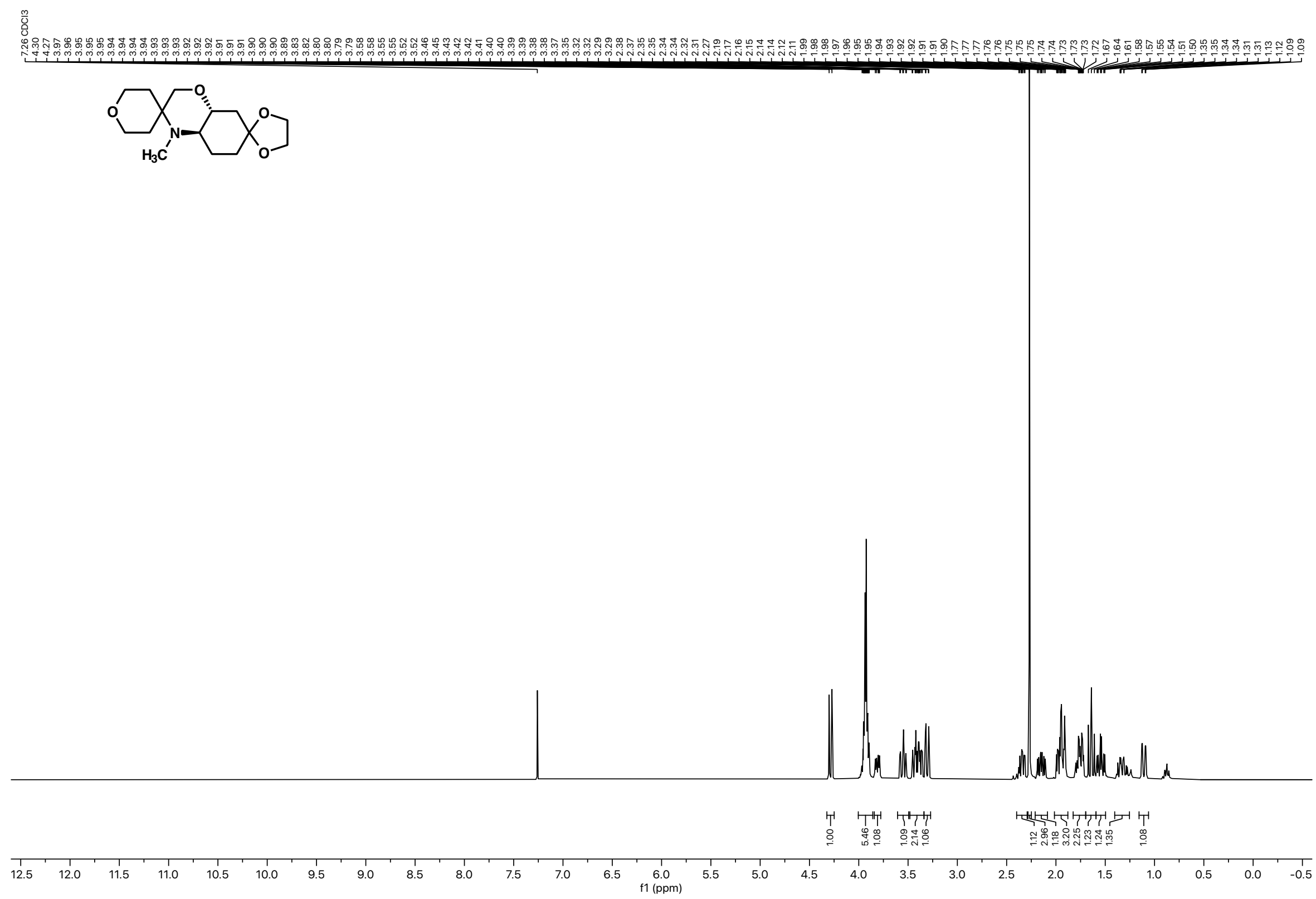

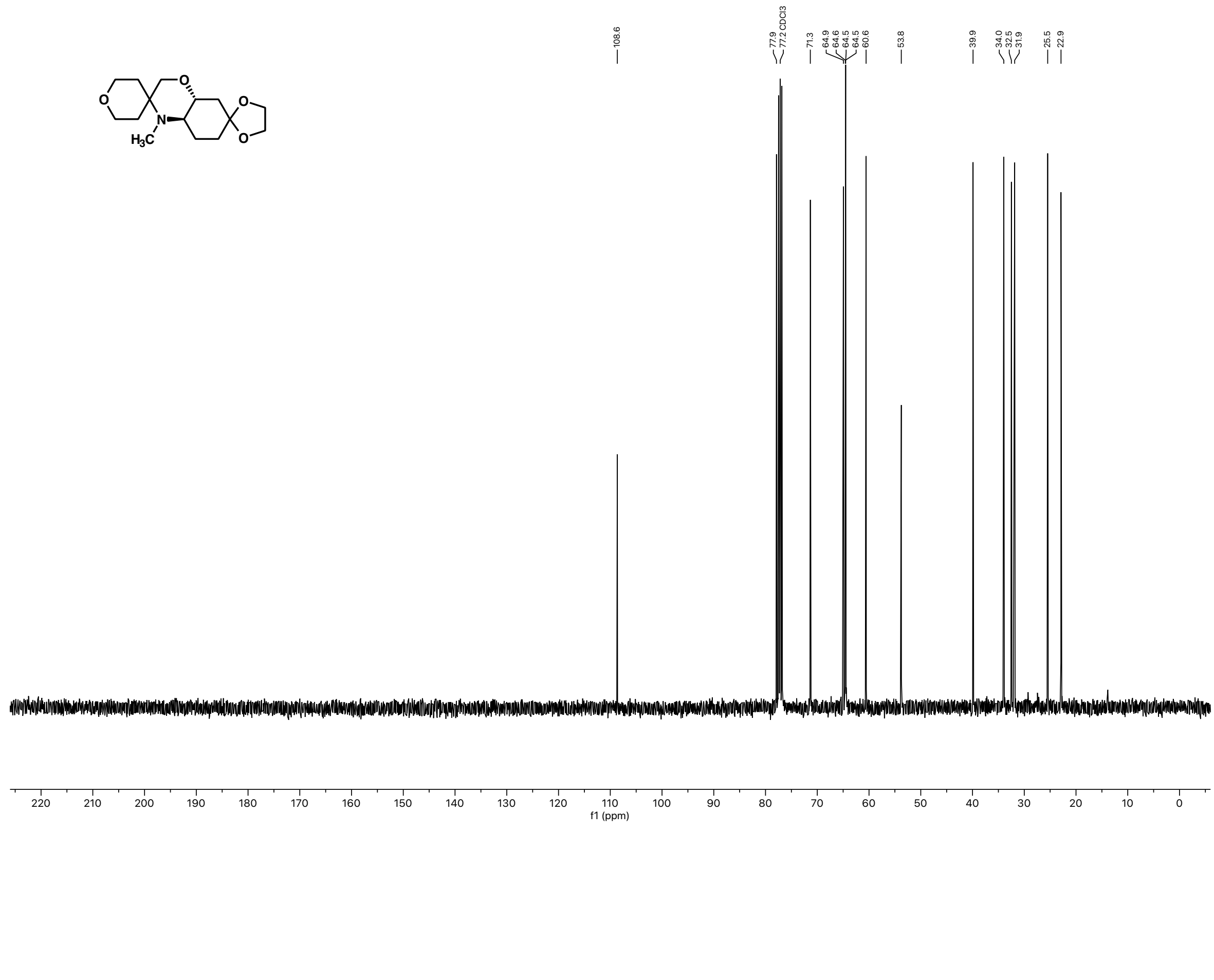

Compound 10: ^{1}H NMR ($CDCl_3$) top, ^{13}C NMR ($CDCl_3$) bottom

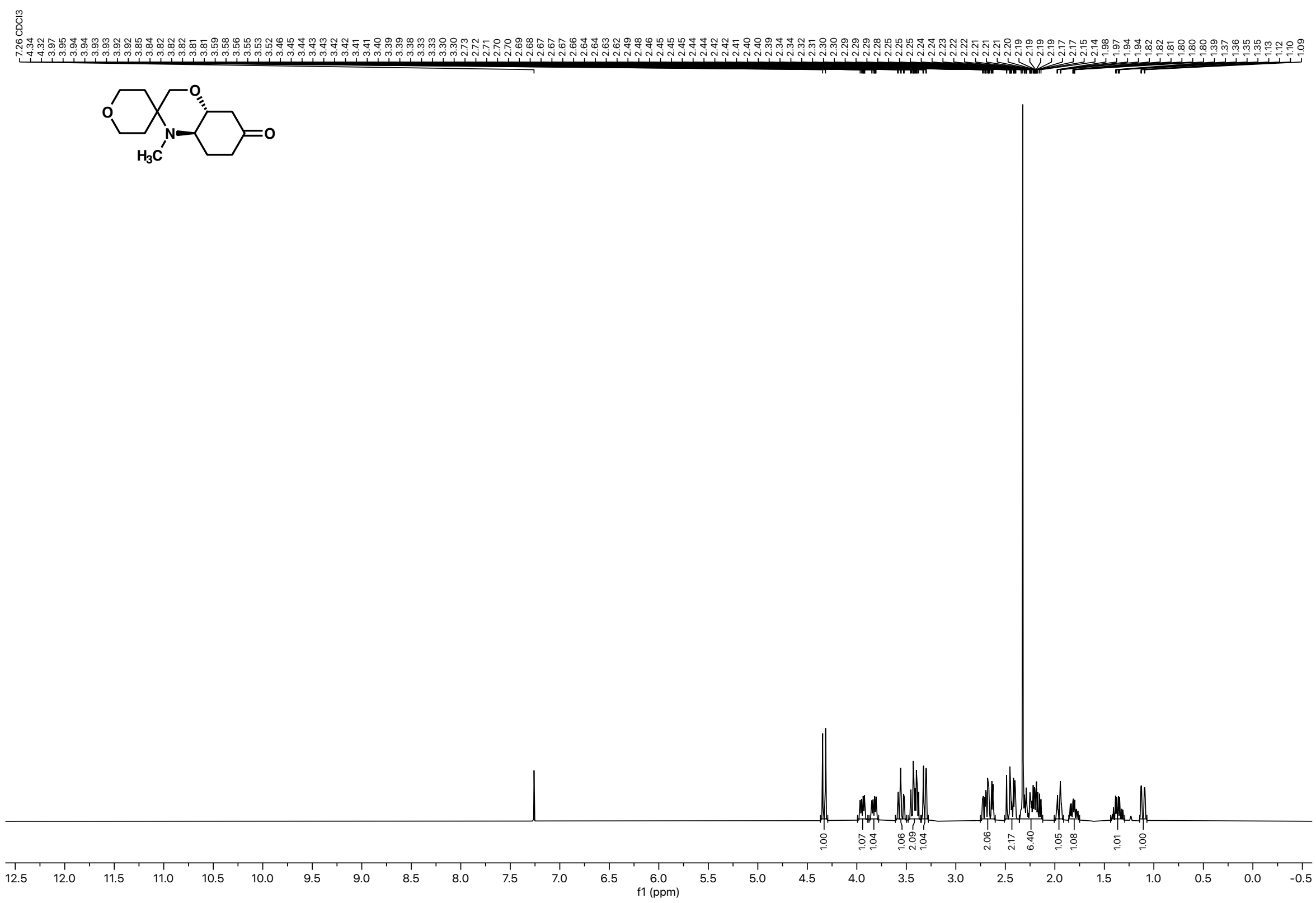

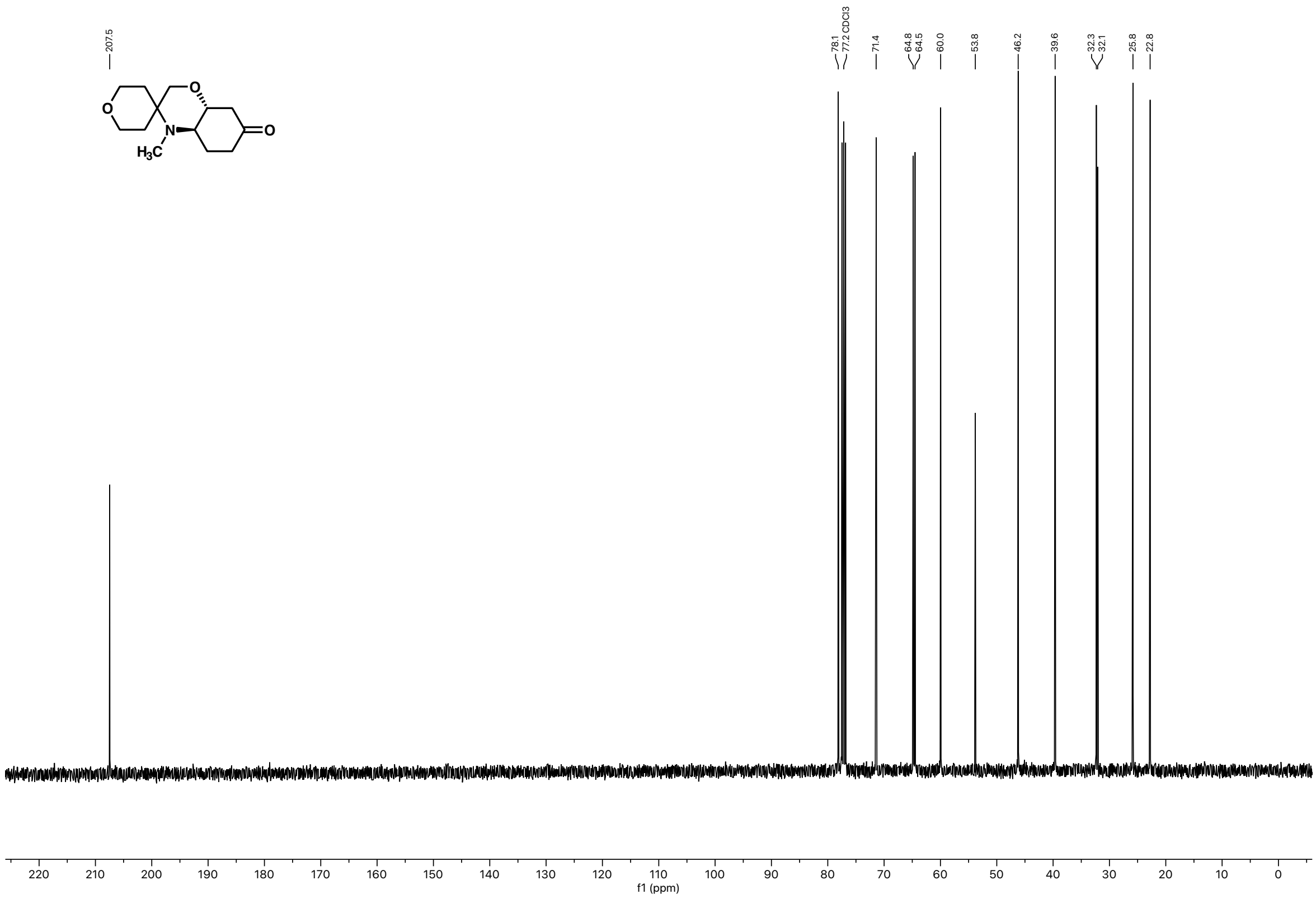

Compound 11: ^{1}H NMR ($CDCl_3$) top, ^{13}C NMR ($CDCl_3$) bottom

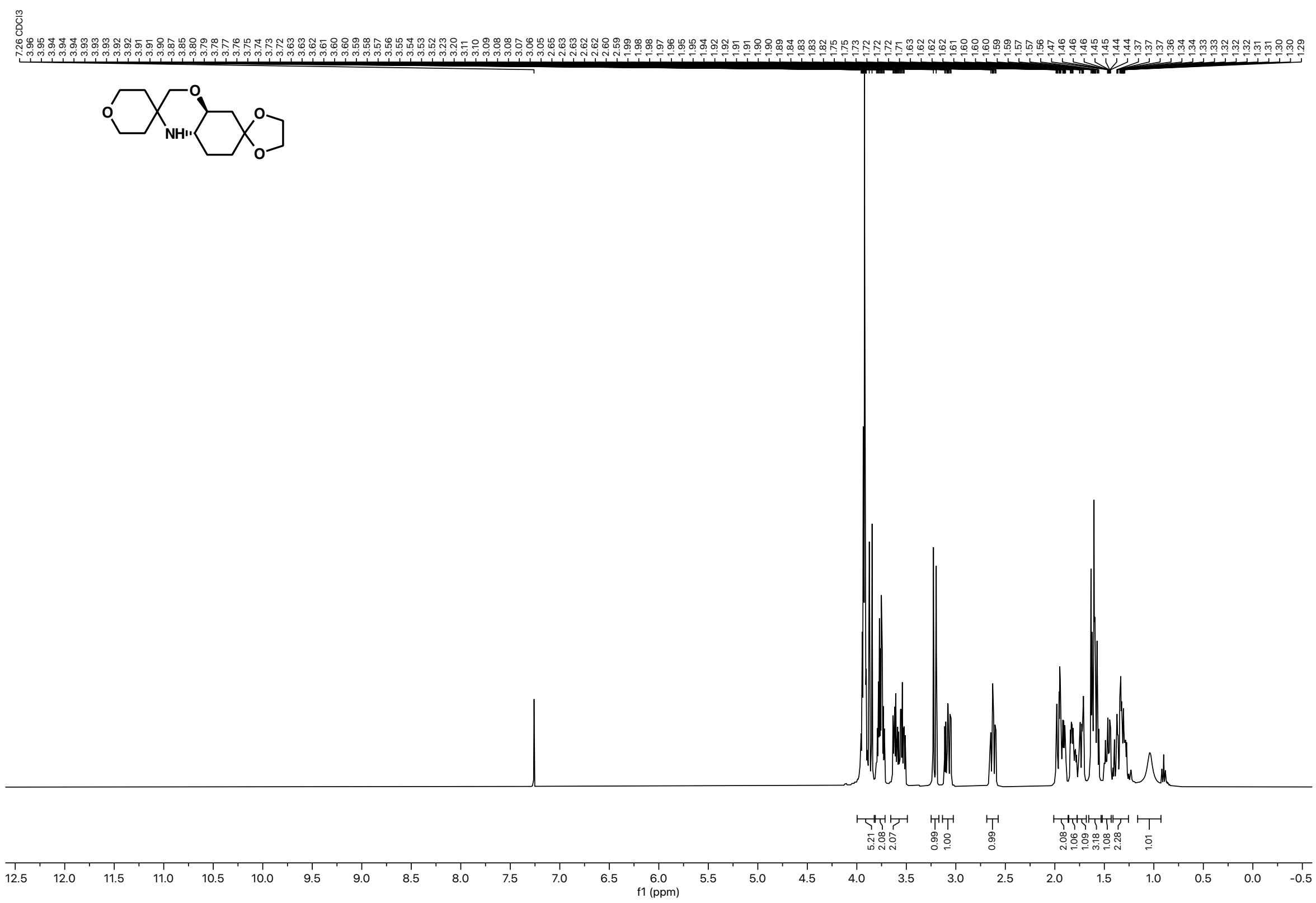

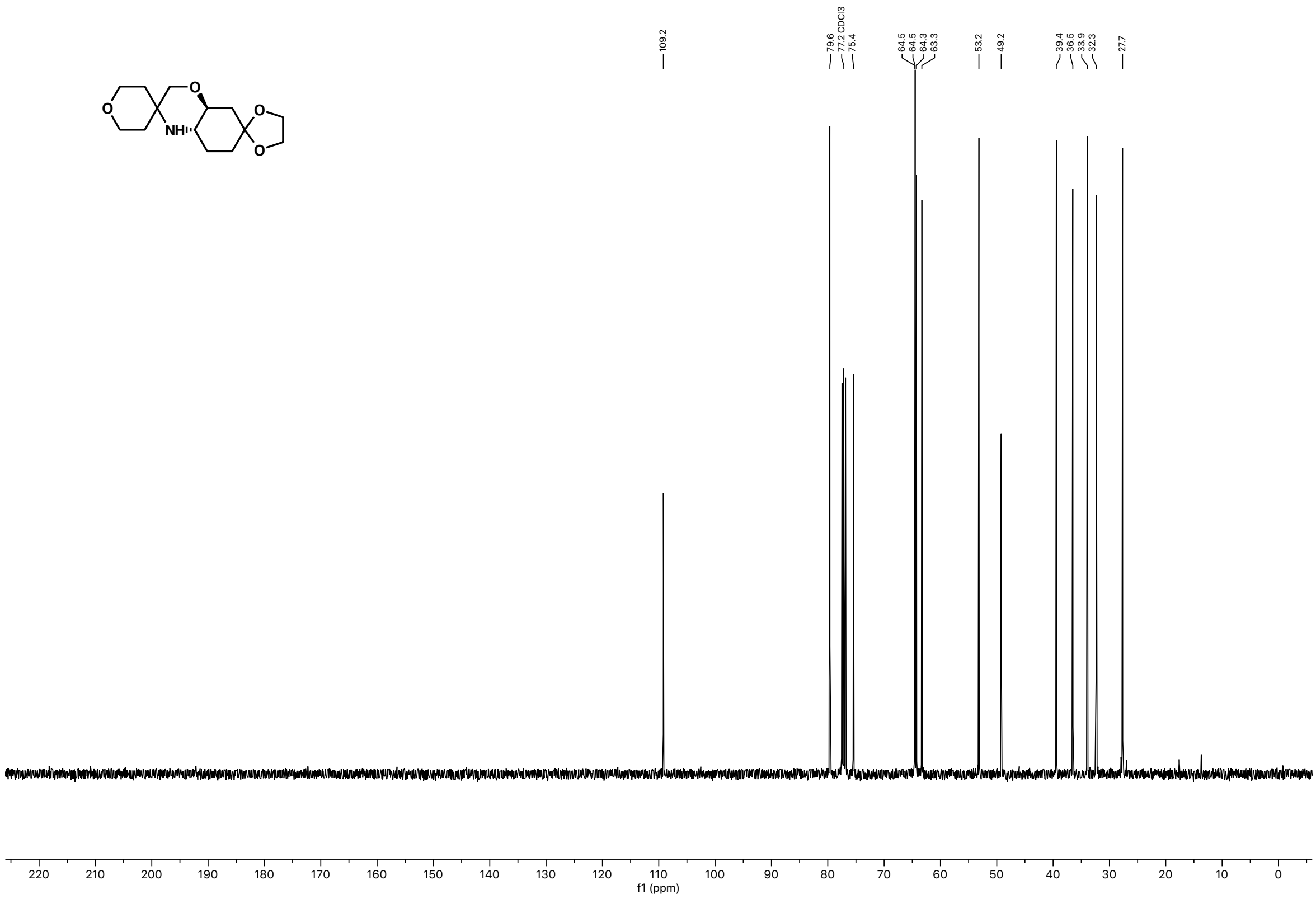

Compound 12: ^{1}H NMR ($CDCl_3$) top, ^{13}C NMR ($CDCl_3$) bottom

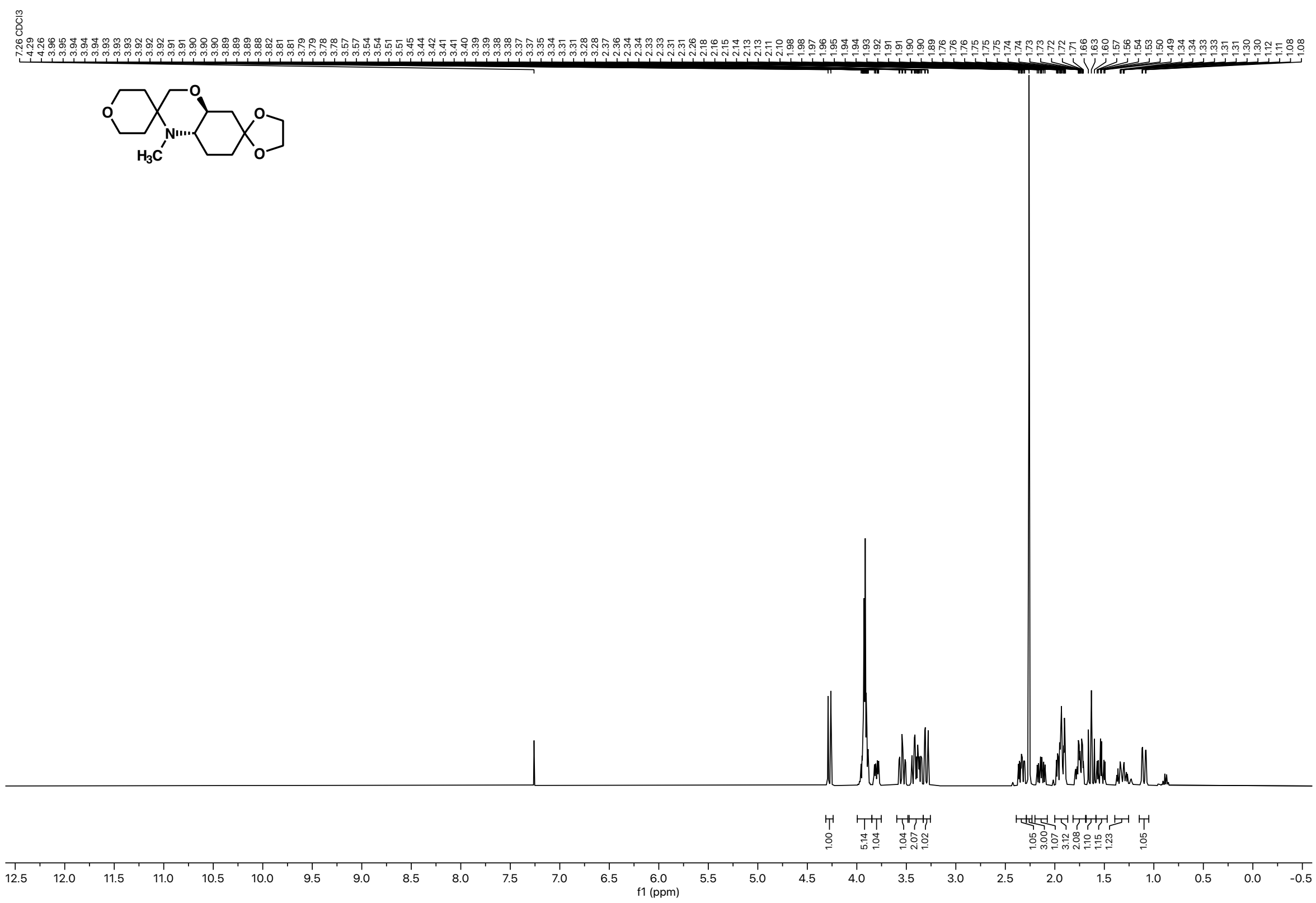

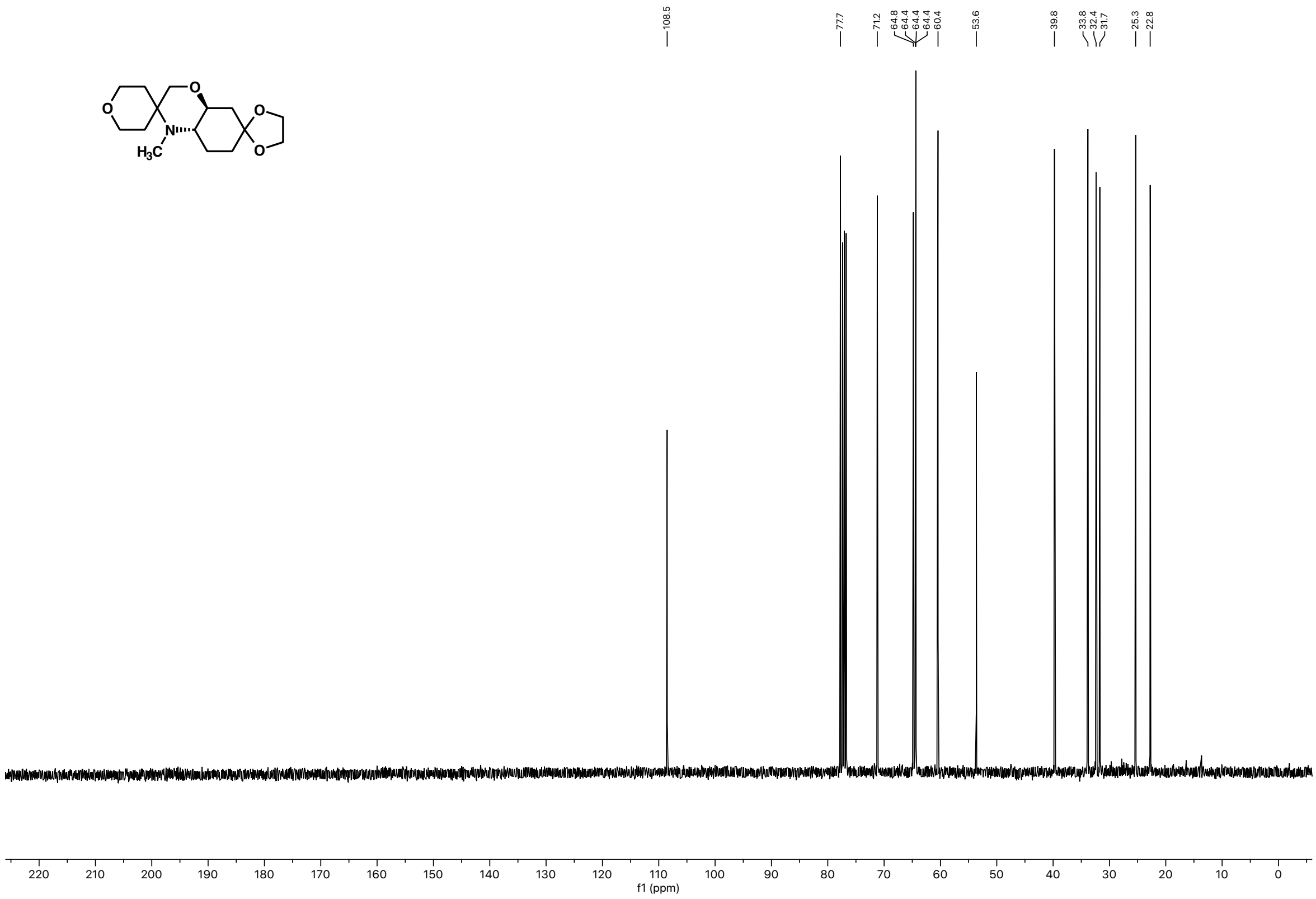

Compound 13: ^{1}H NMR ($CDCl_3$) top, ^{13}C NMR ($CDCl_3$) bottom

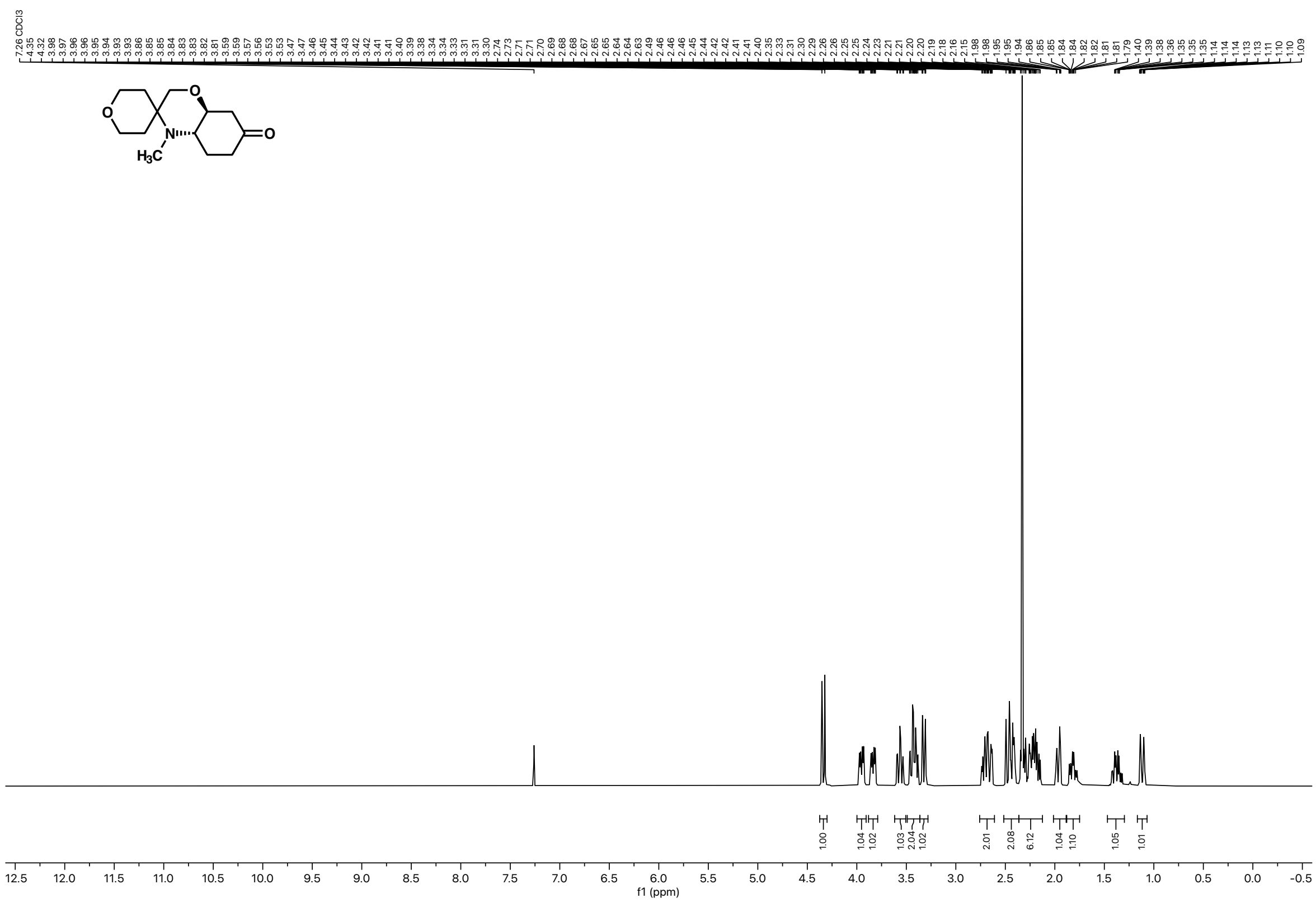

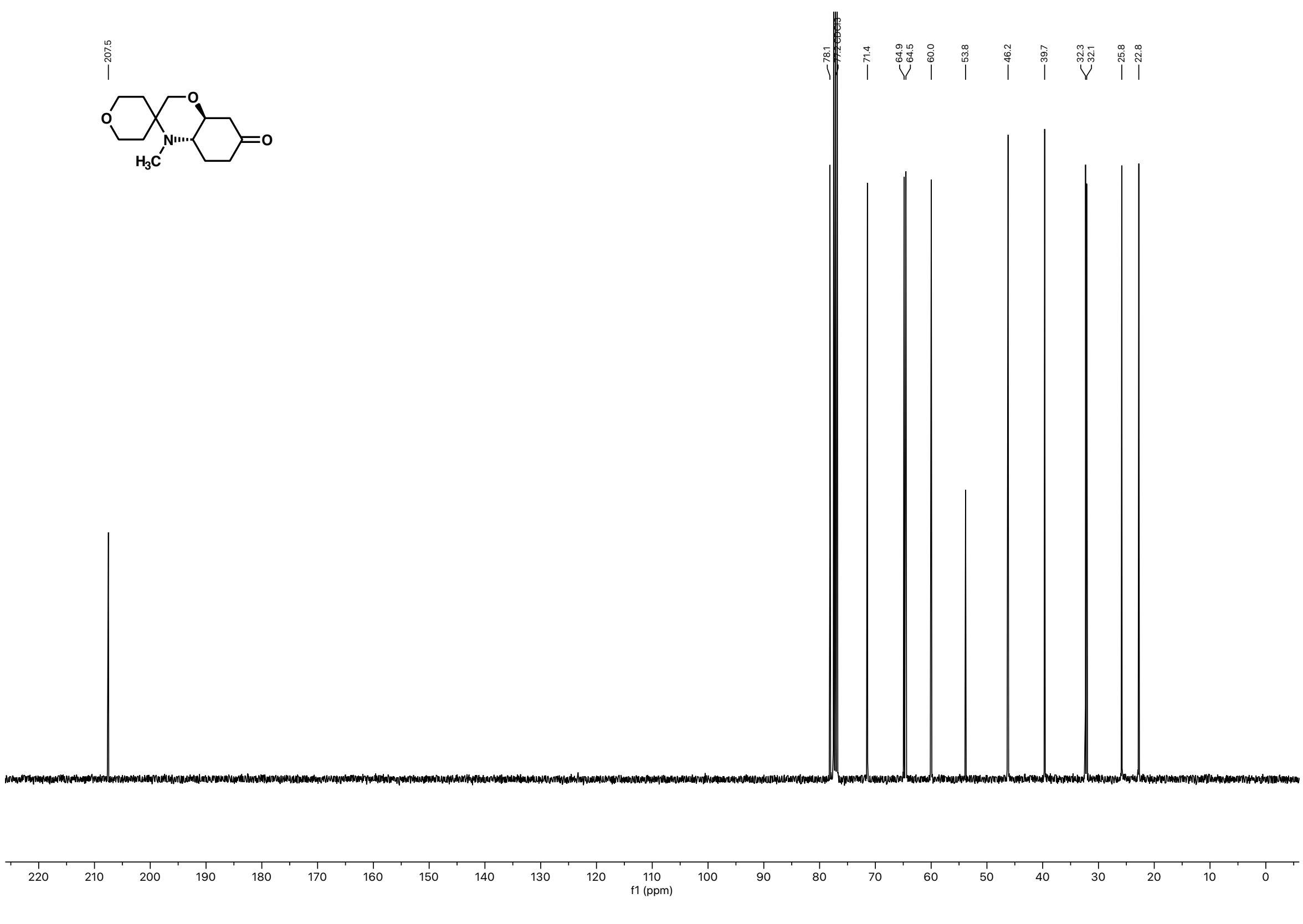

Compound 14: ^{1}H NMR ($CDCl_3$) top, ^{13}C NMR ($CDCl_3$) bottom

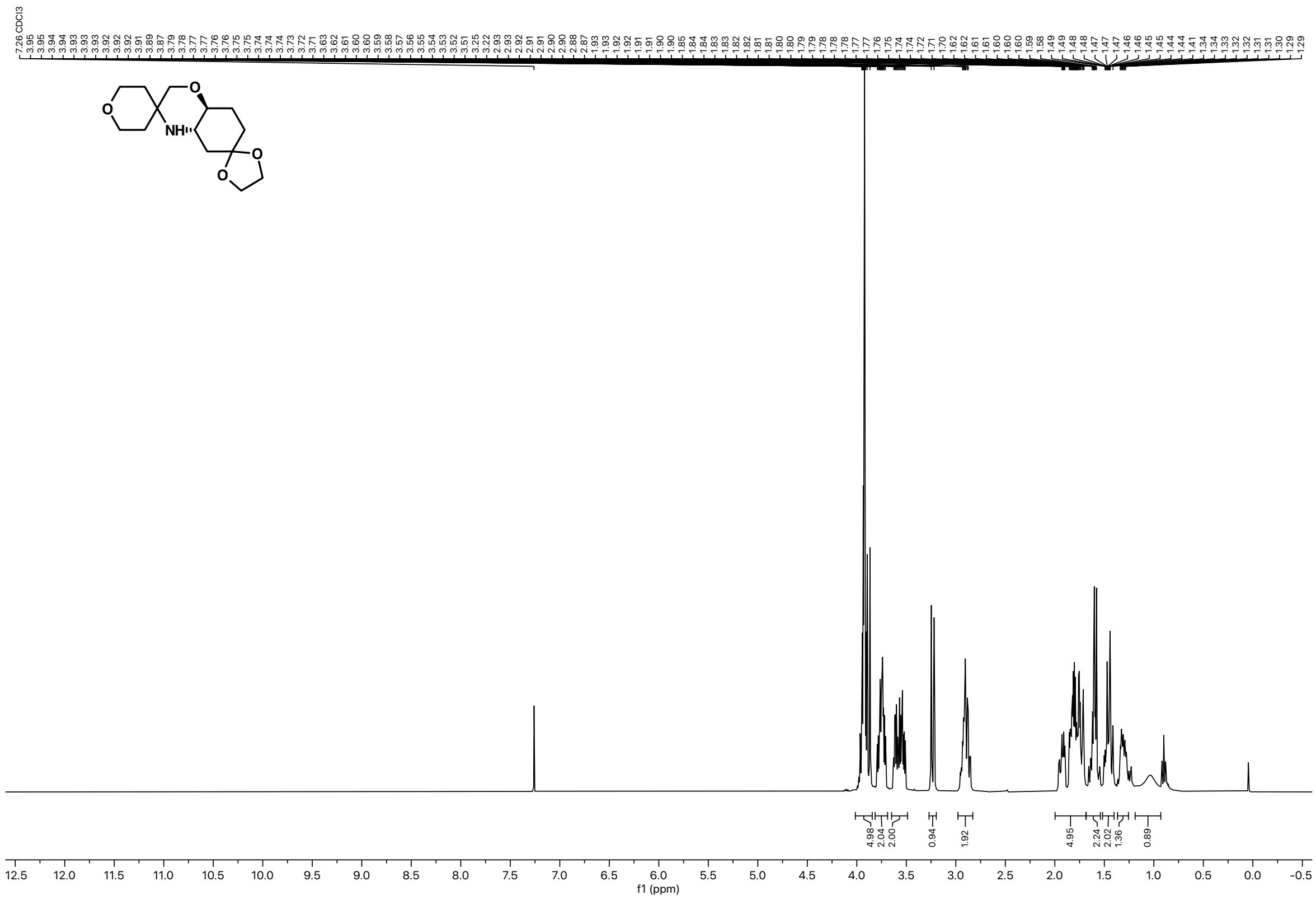

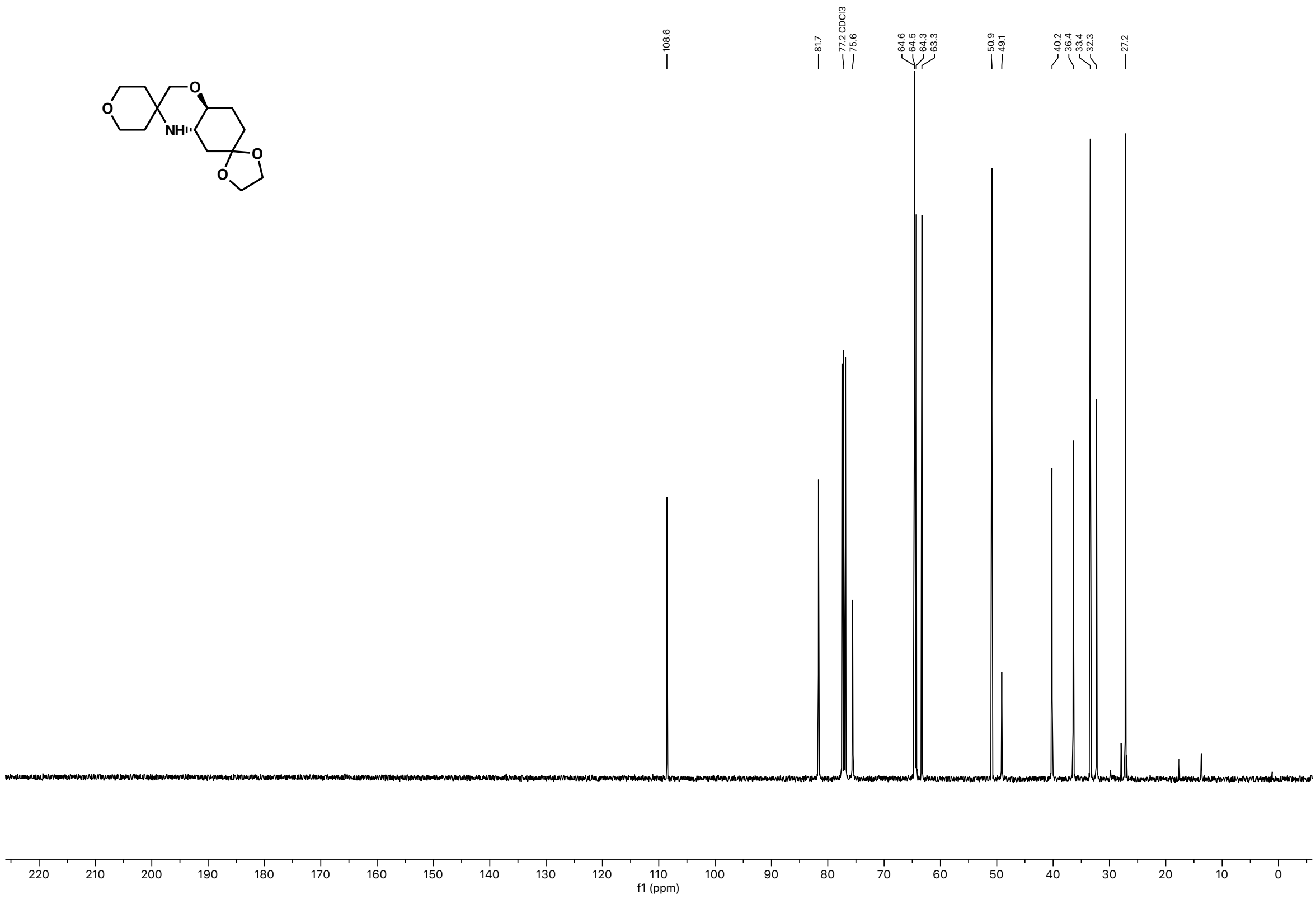

Compound 15: ^{1}H NMR ($CDCl_3$) top, ^{13}C NMR ($CDCl_3$) bottom

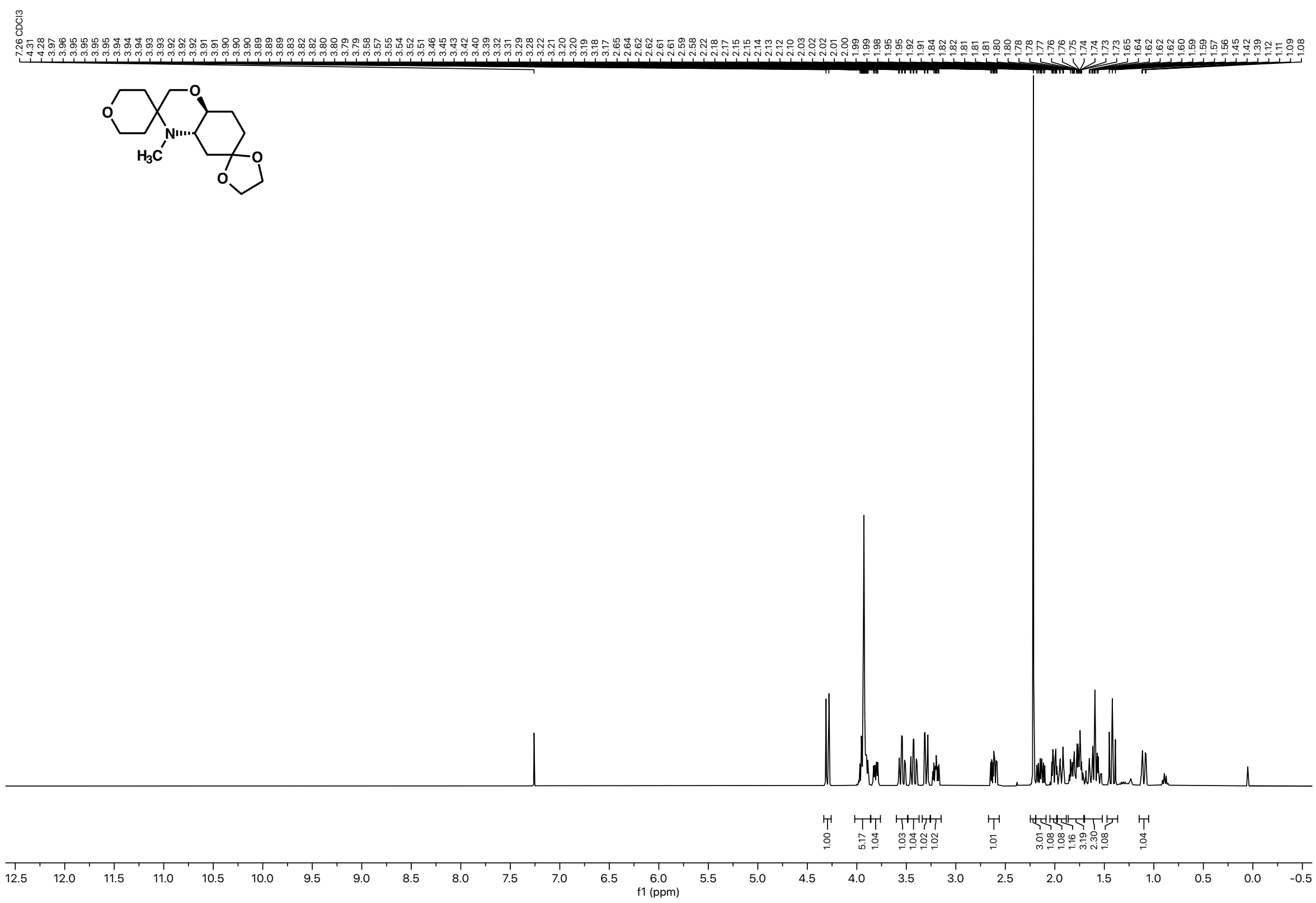

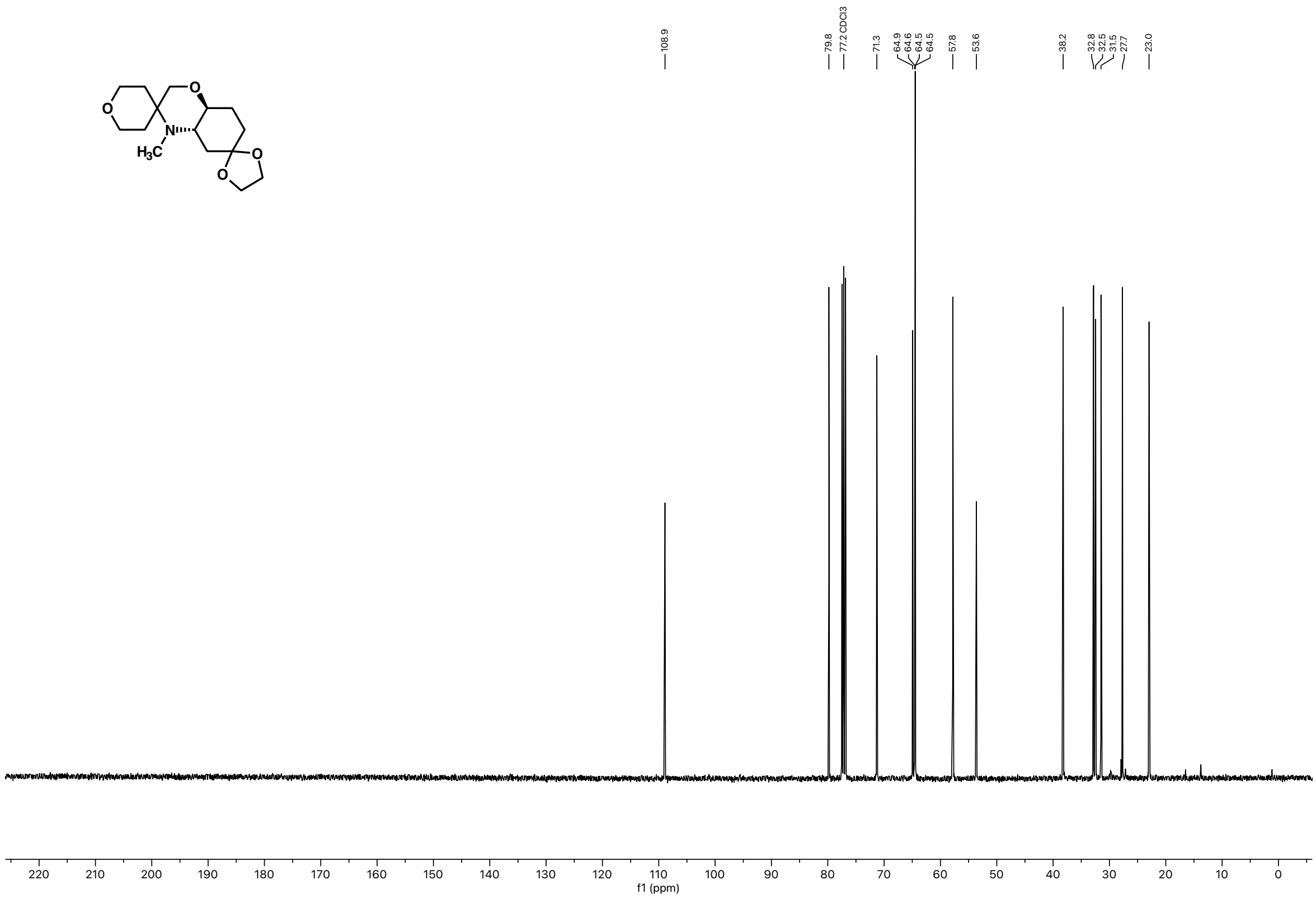

Compound 16: ^{1}H NMR ($CDCl_3$) top, ^{13}C NMR ($CDCl_3$) bottom

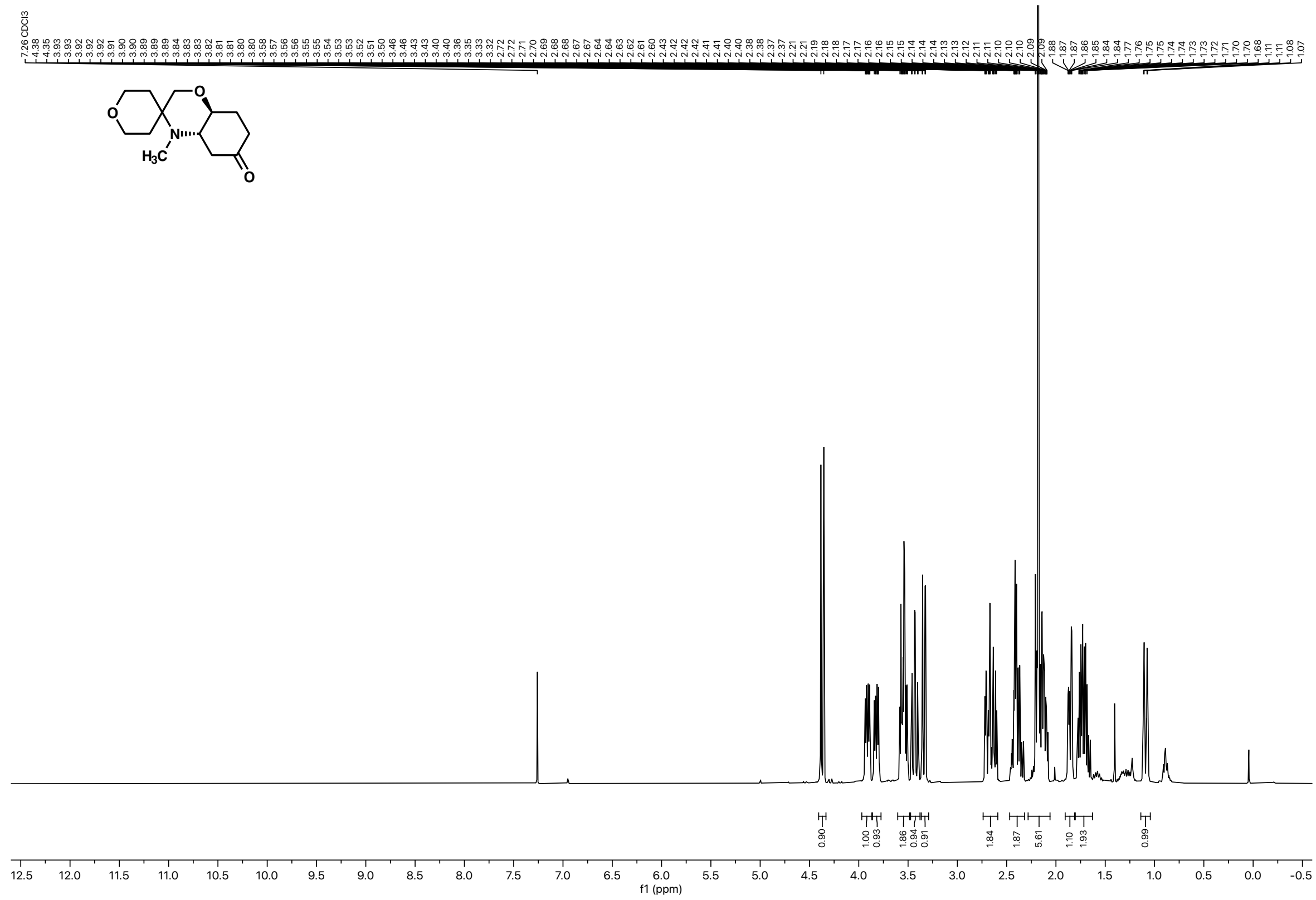

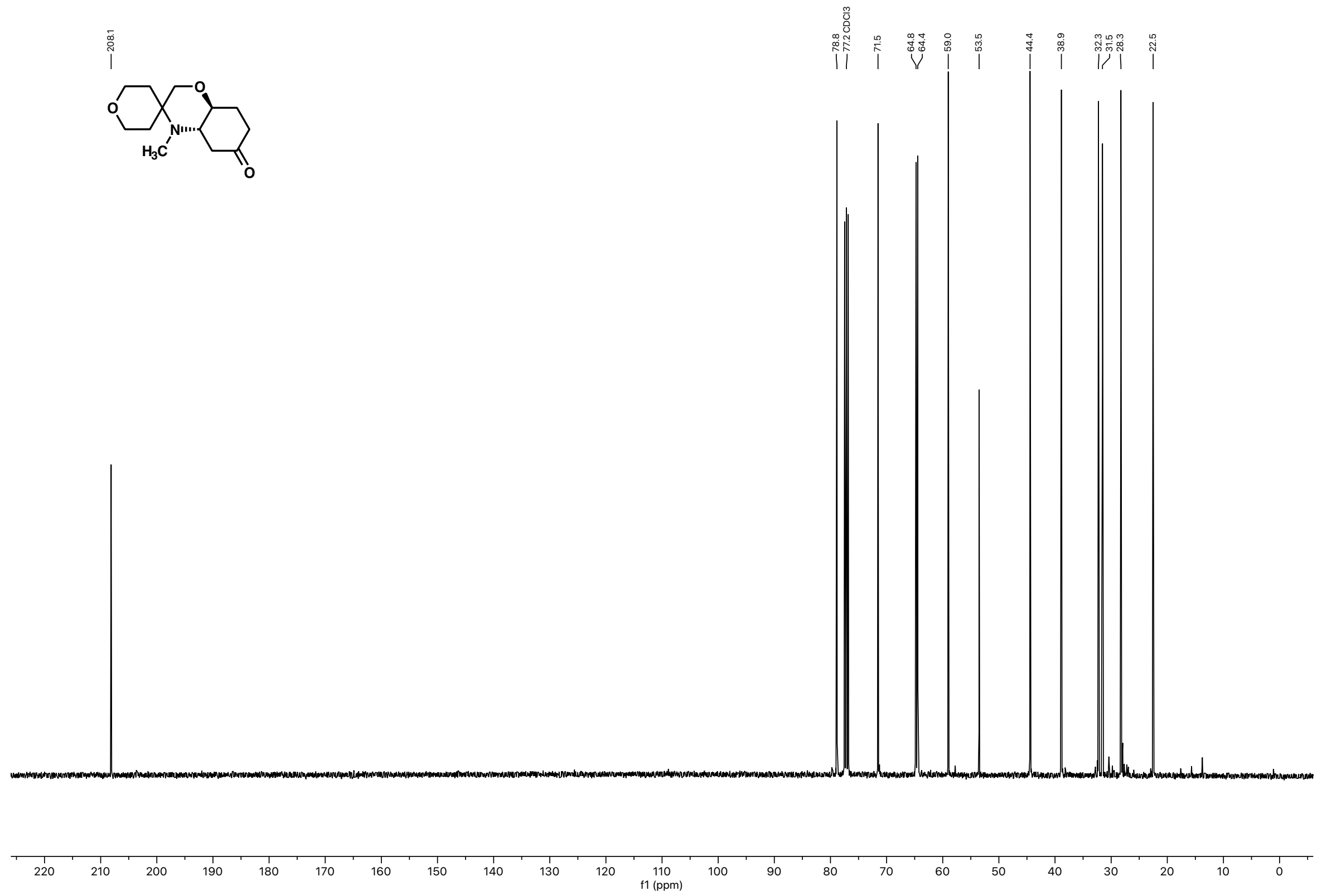

Compound 17a: ^{1}H NMR ($CDCl_3$) top, ^{13}C NMR ($CDCl_3$) bottom

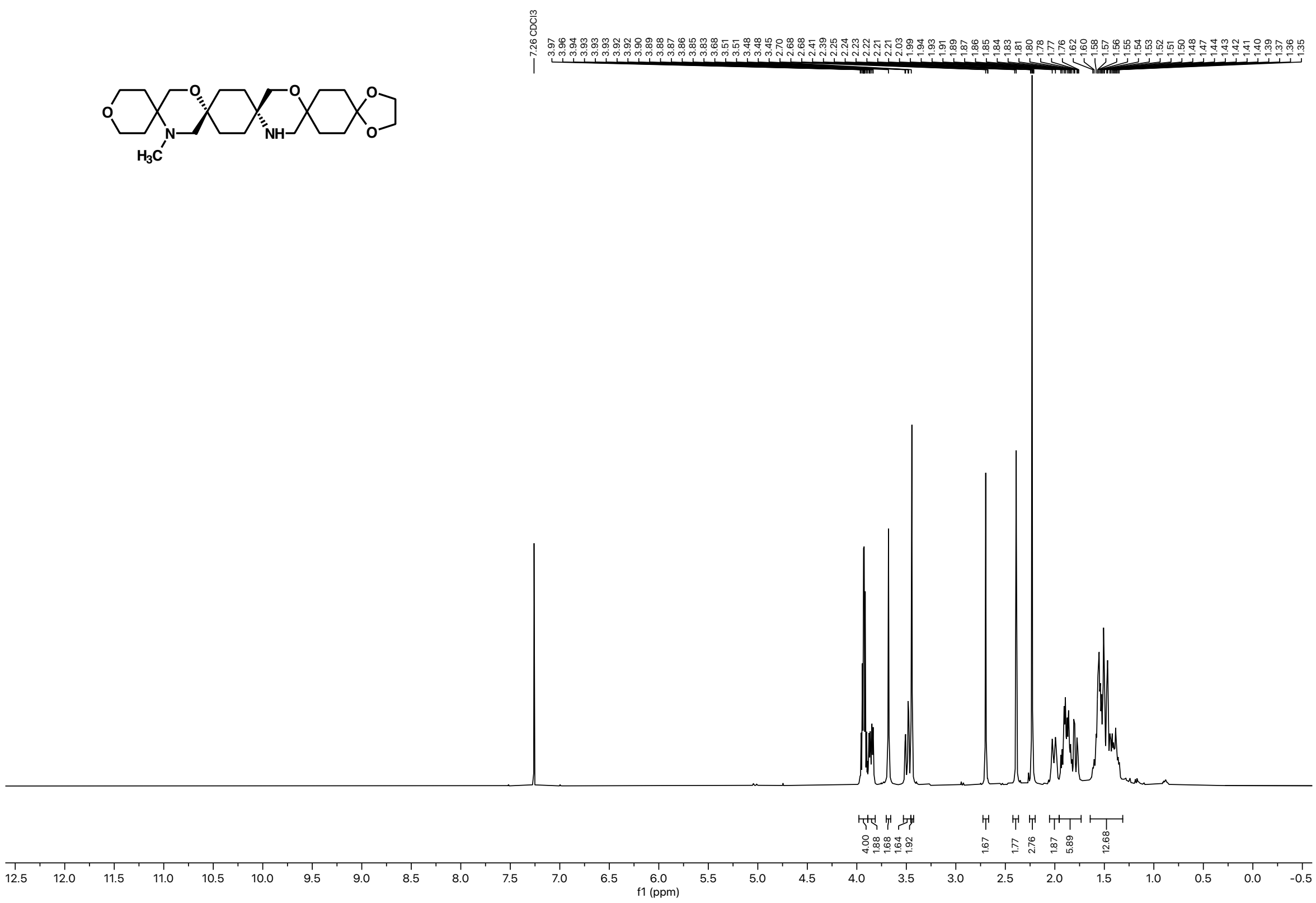

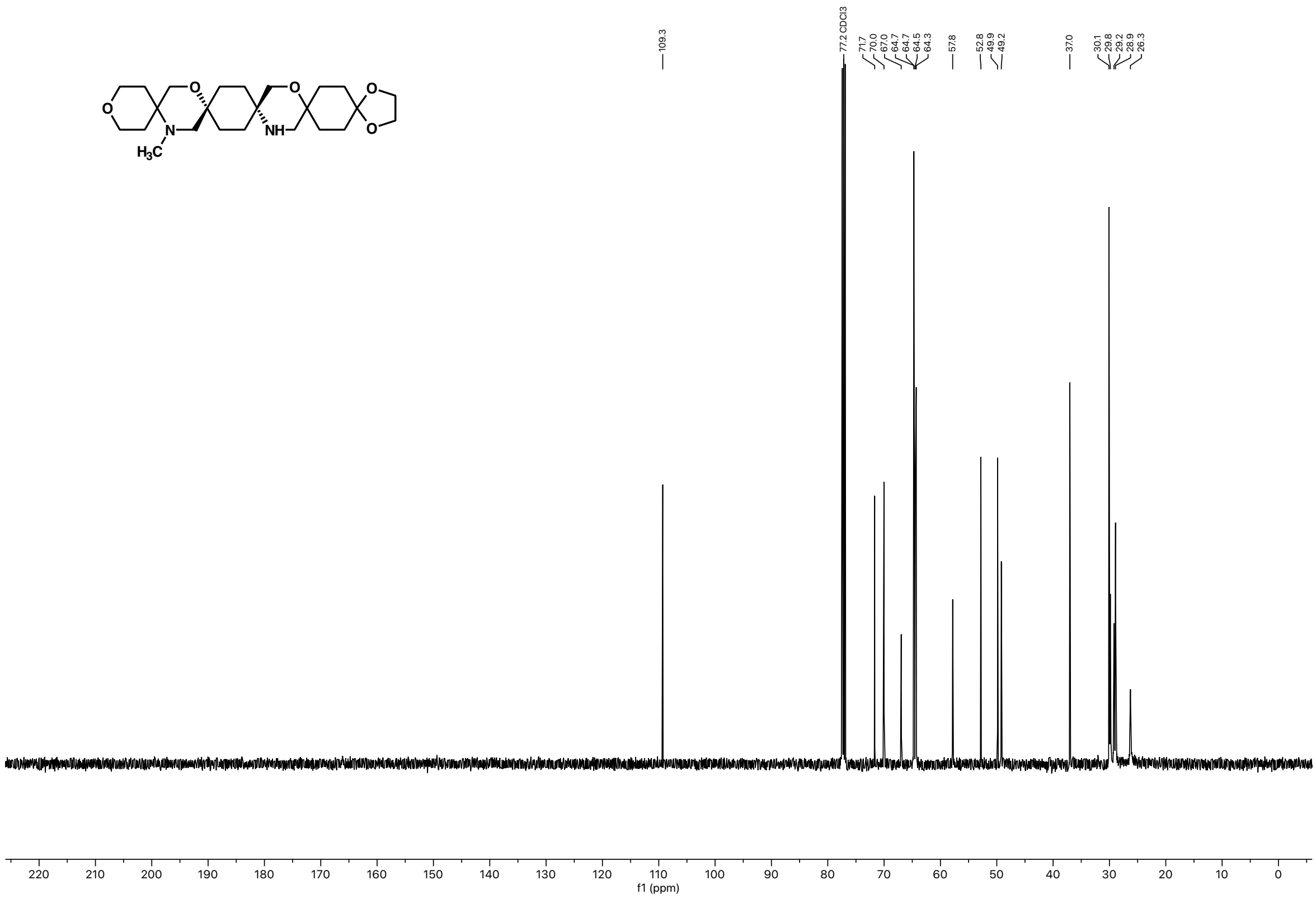

Compound 17b: ^{1}H NMR ($CDCl_3$) top, ^{13}C NMR ($CDCl_3$) bottom

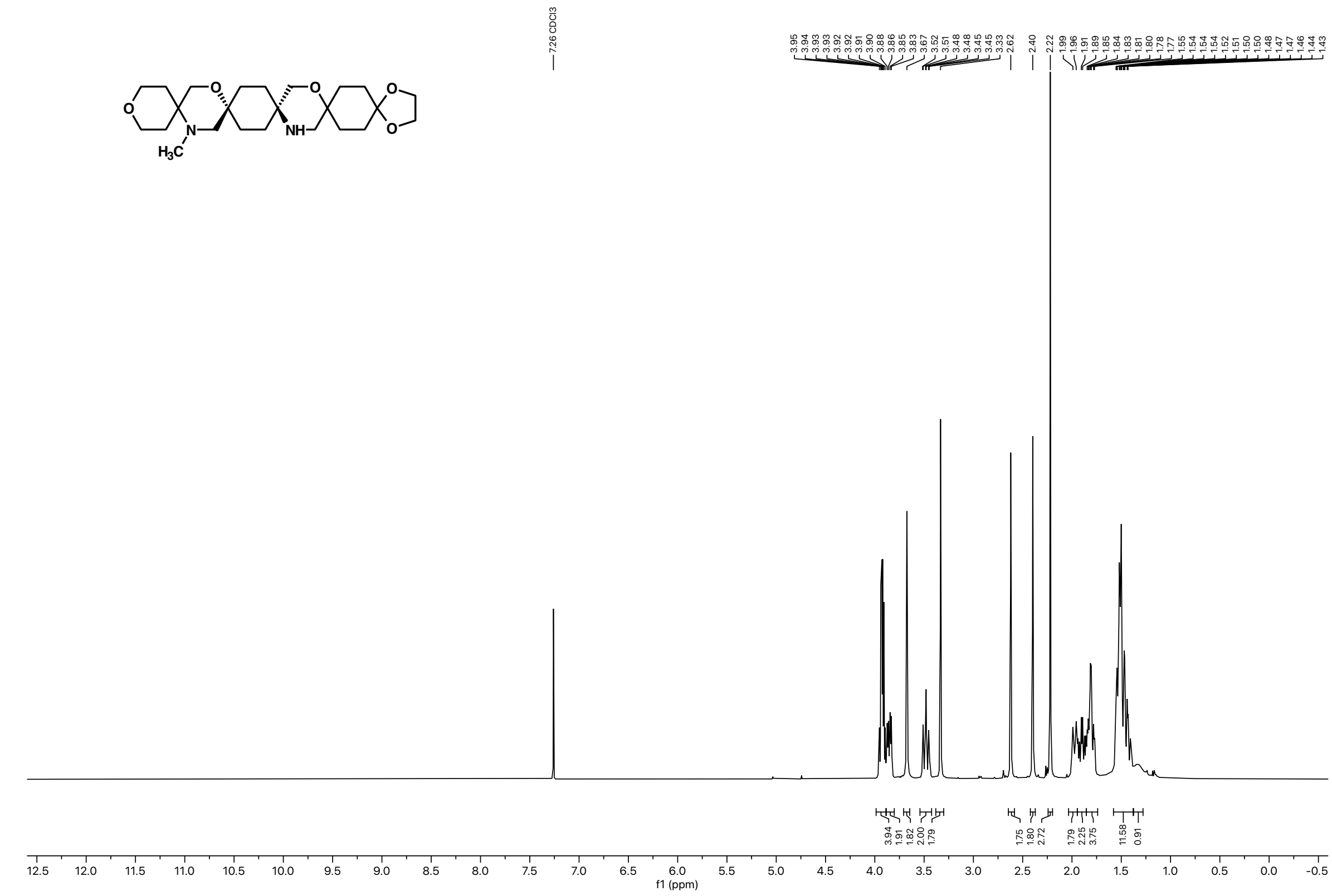

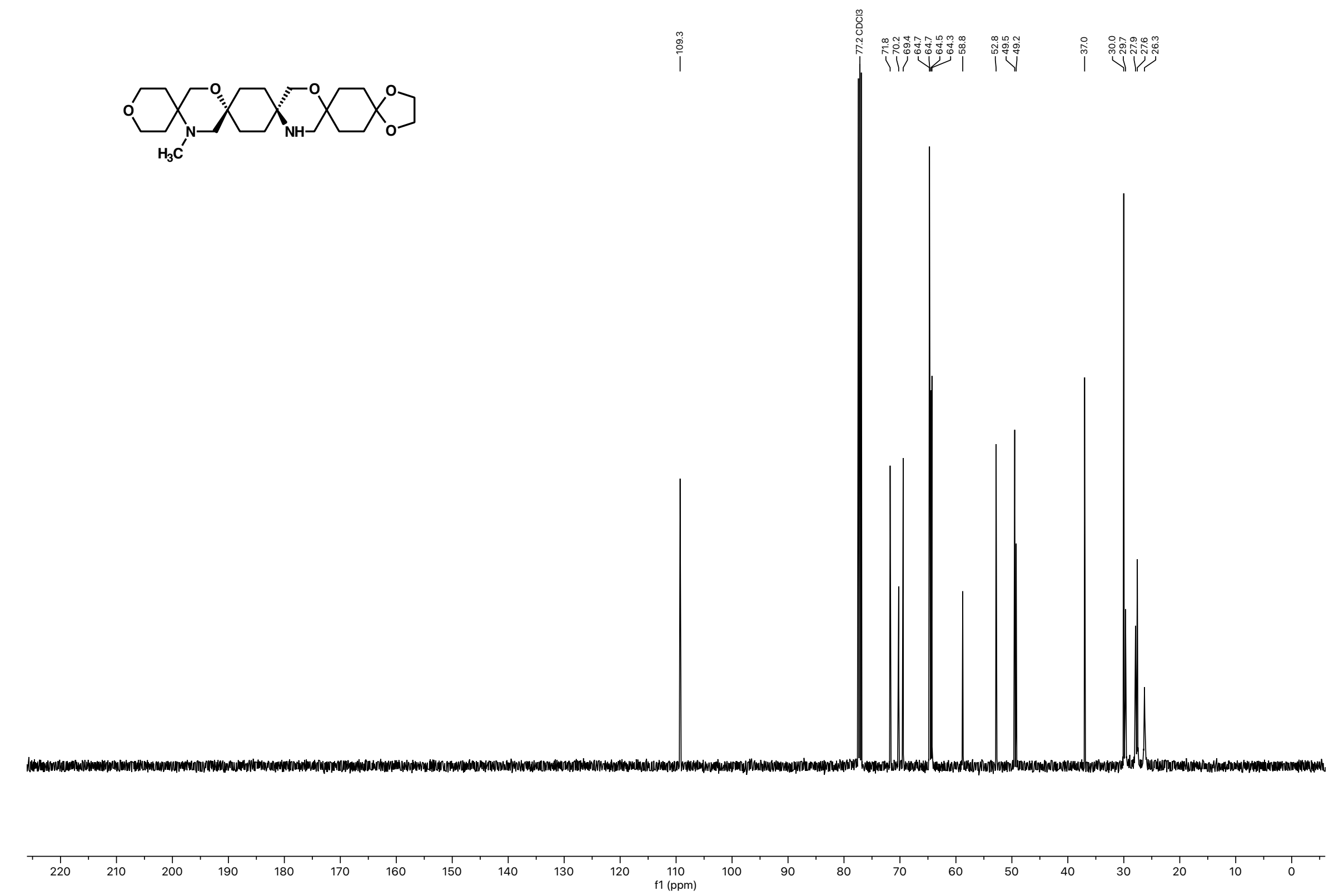

Compound 18a: ^{1}H NMR ($CDCl_3$) top, ^{13}C NMR ($CDCl_3$) bottom

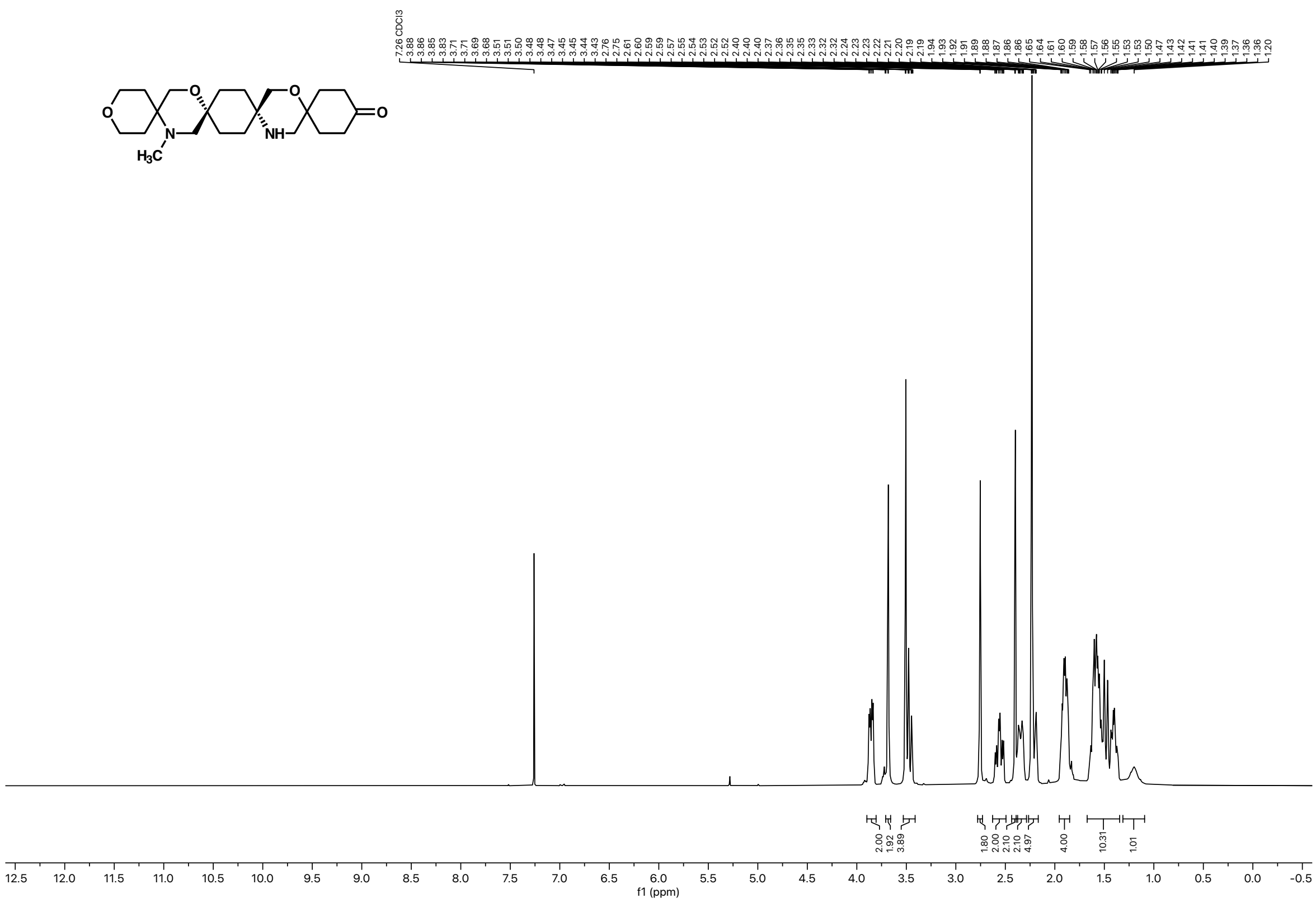

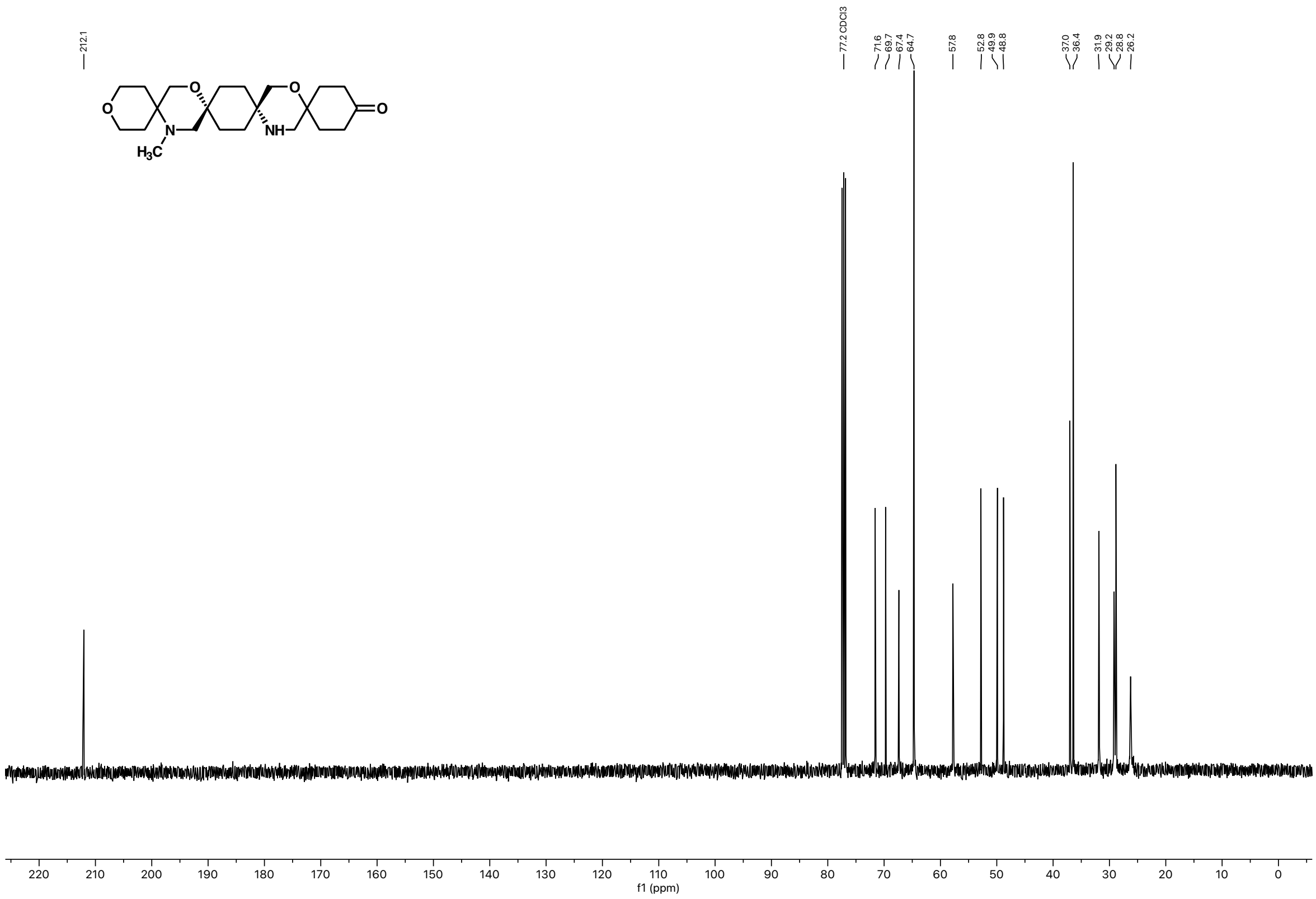

Compound 18b: ^{1}H NMR ($CDCl_3$) top, ^{13}C NMR ($CDCl_3$) bottom

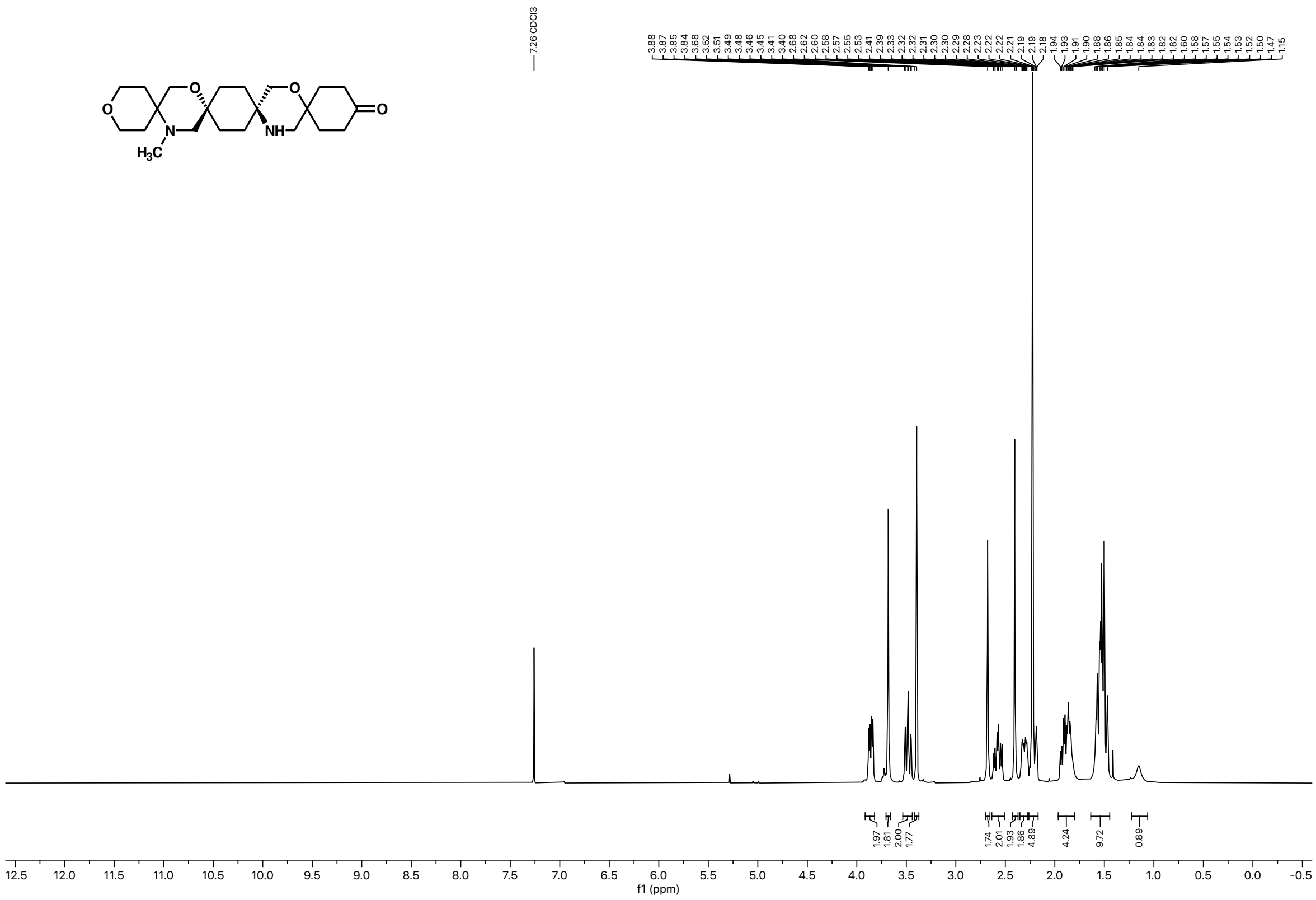

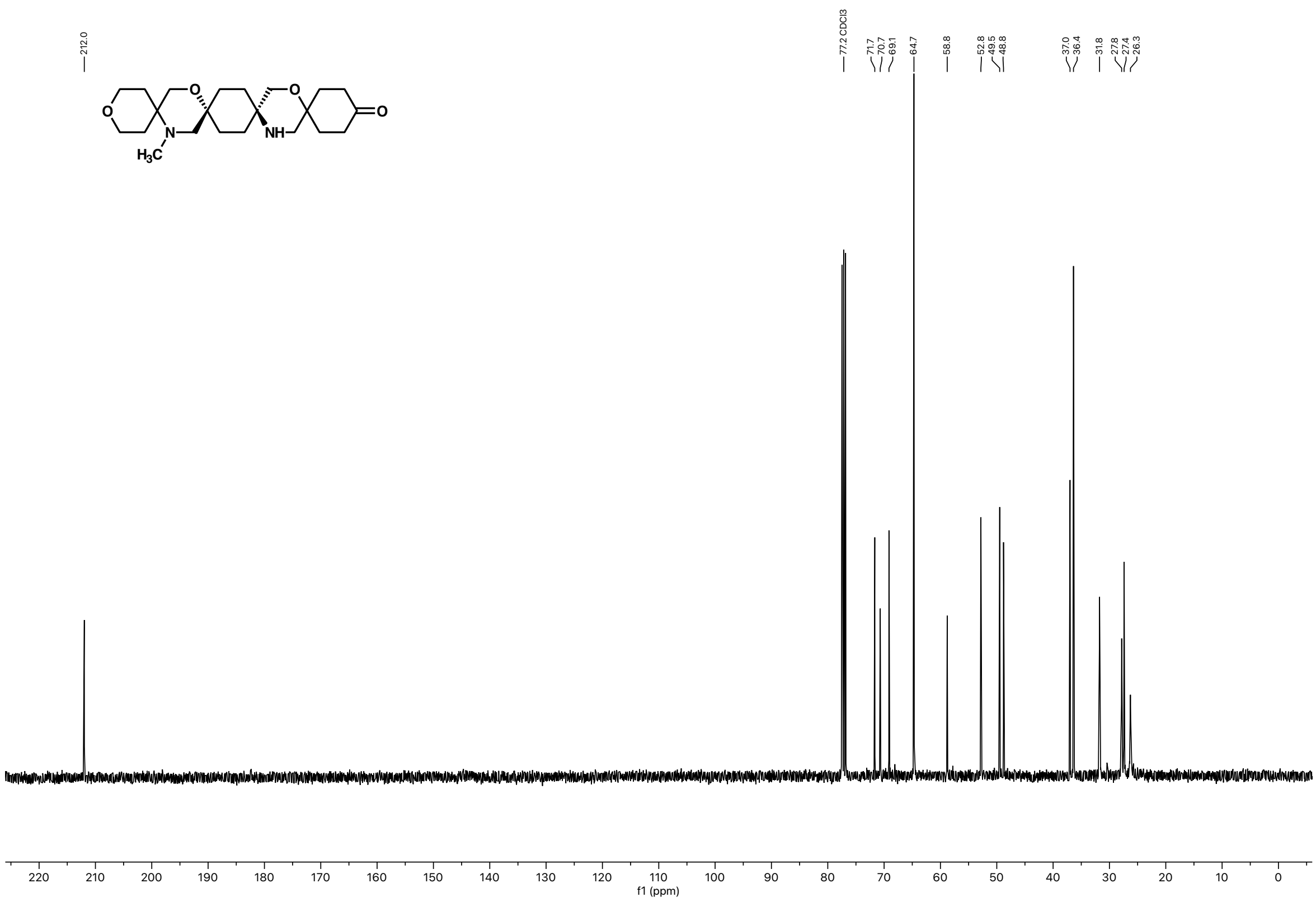

Compound 19: ^{1}H NMR ($CDCl_3$) top, ^{13}C NMR ($CDCl_3$) bottom

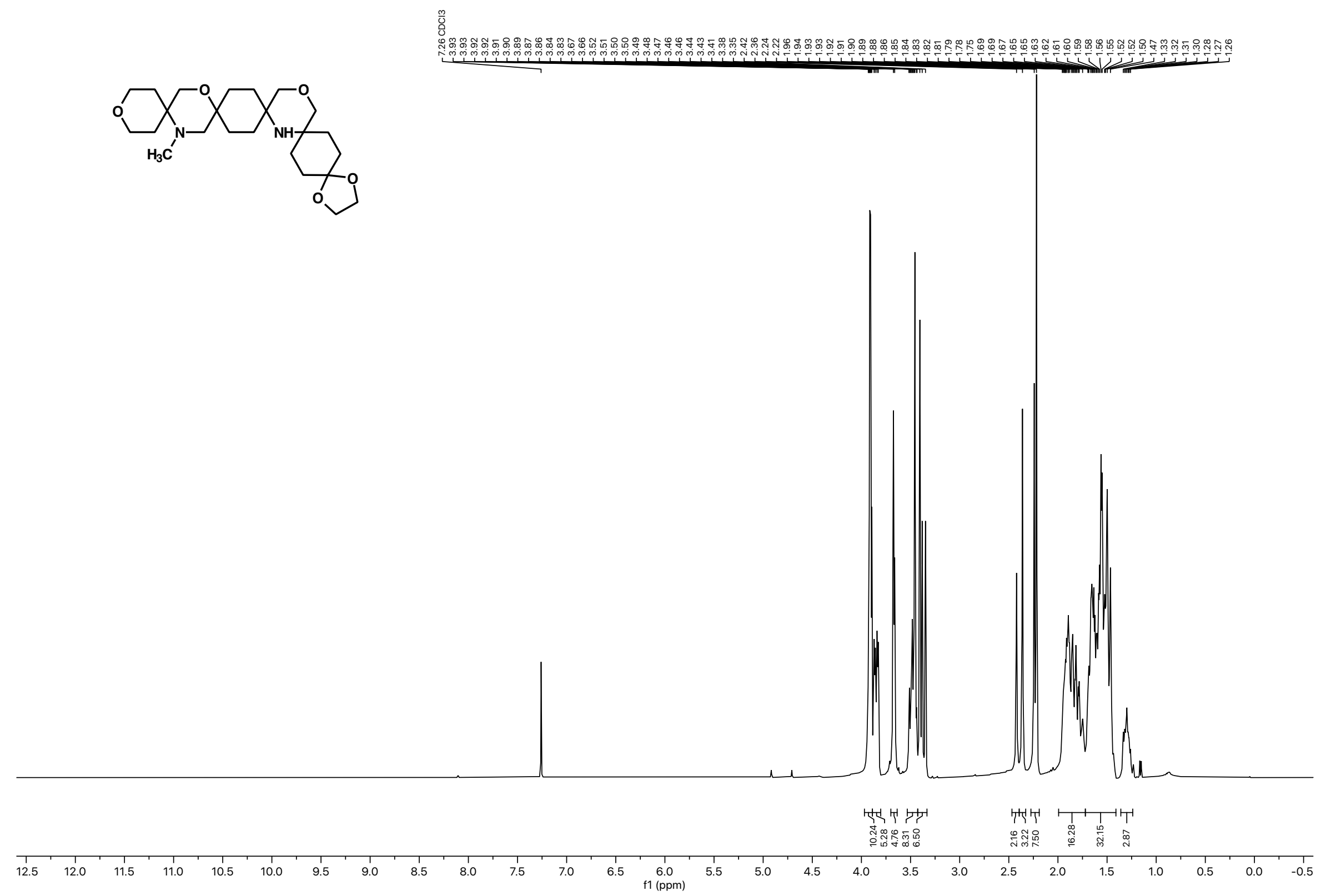

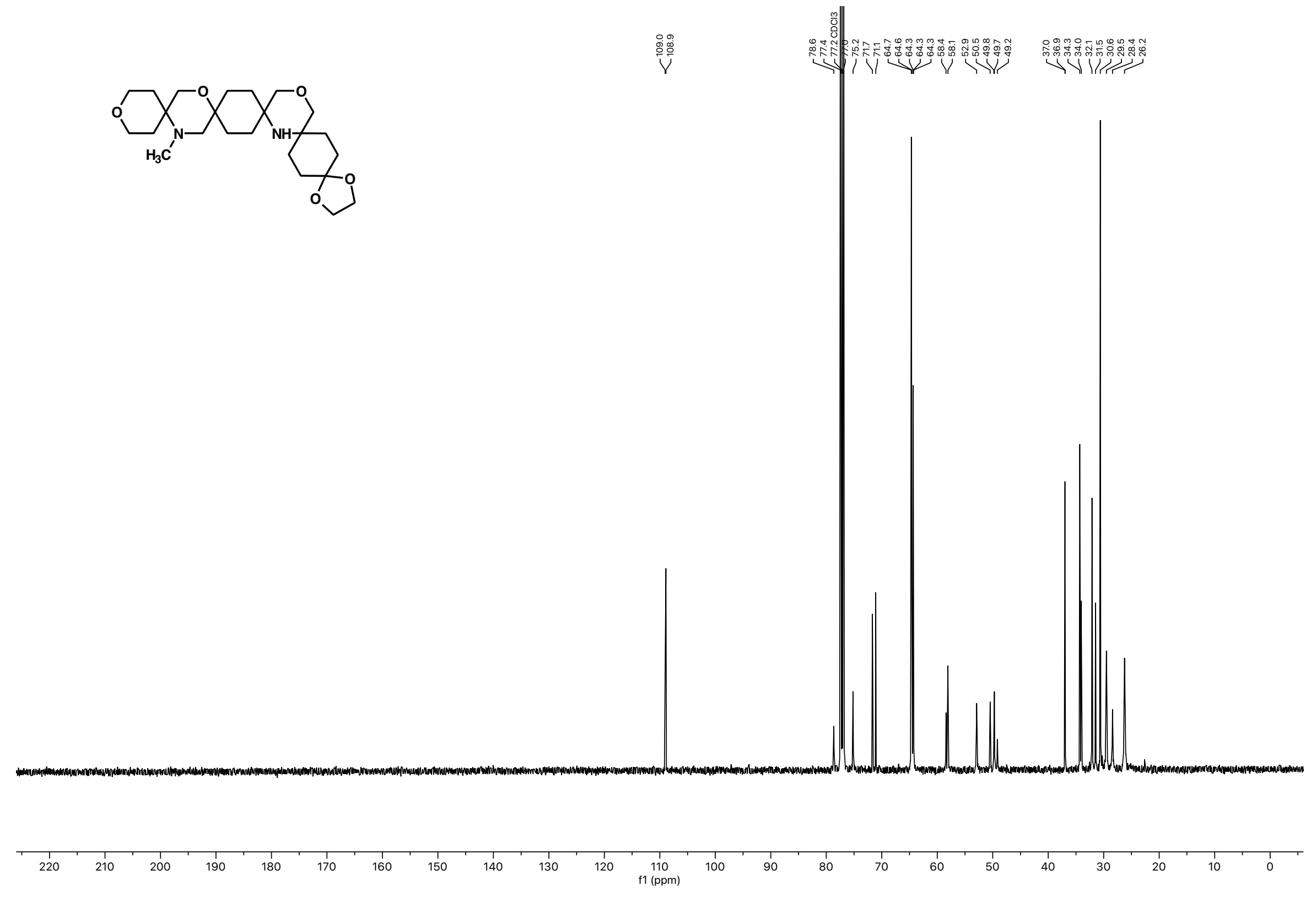

Compound 20: ^{1}H NMR ($CDCl_3$) top, ^{13}C NMR ($CDCl_3$) bottom

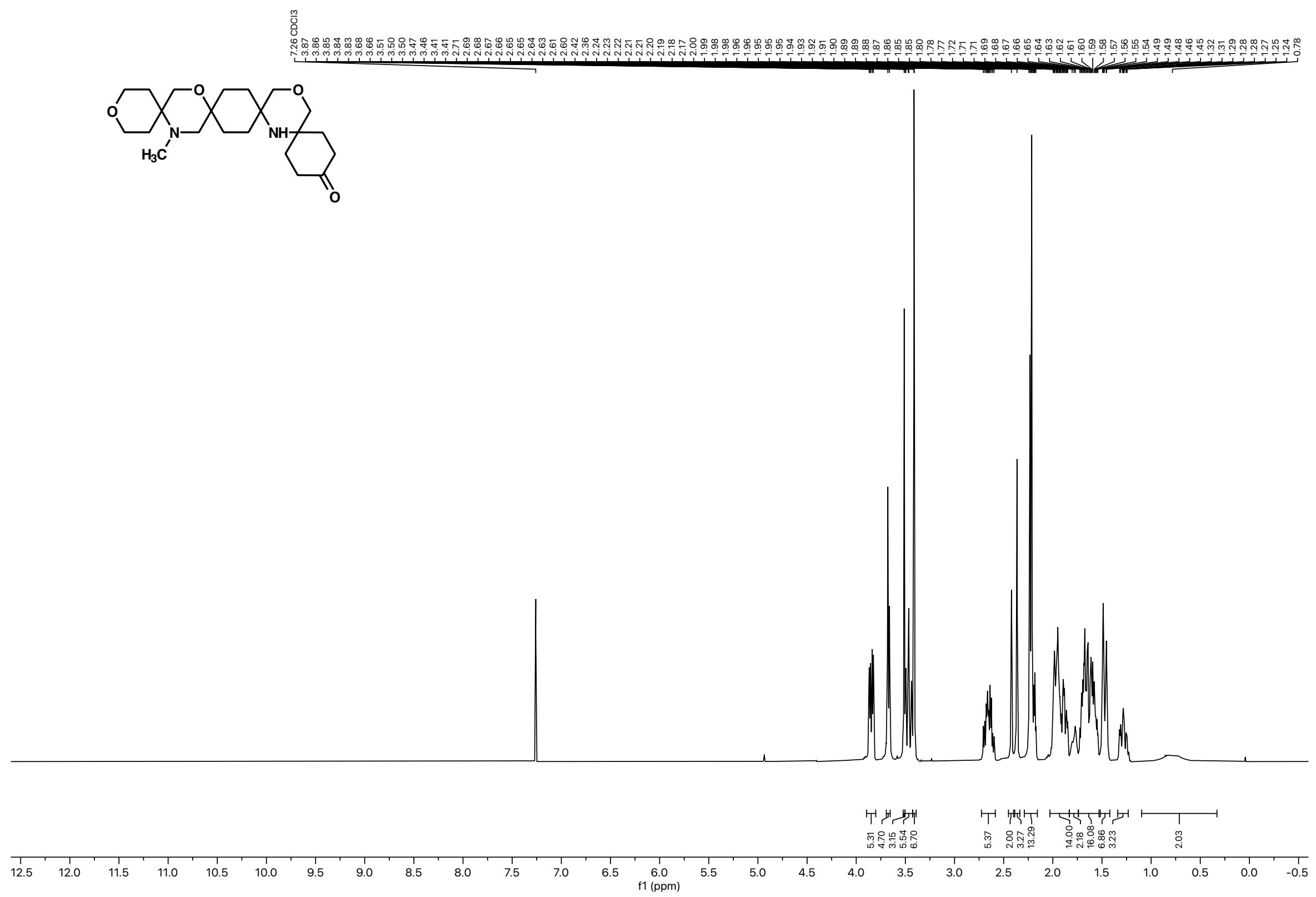

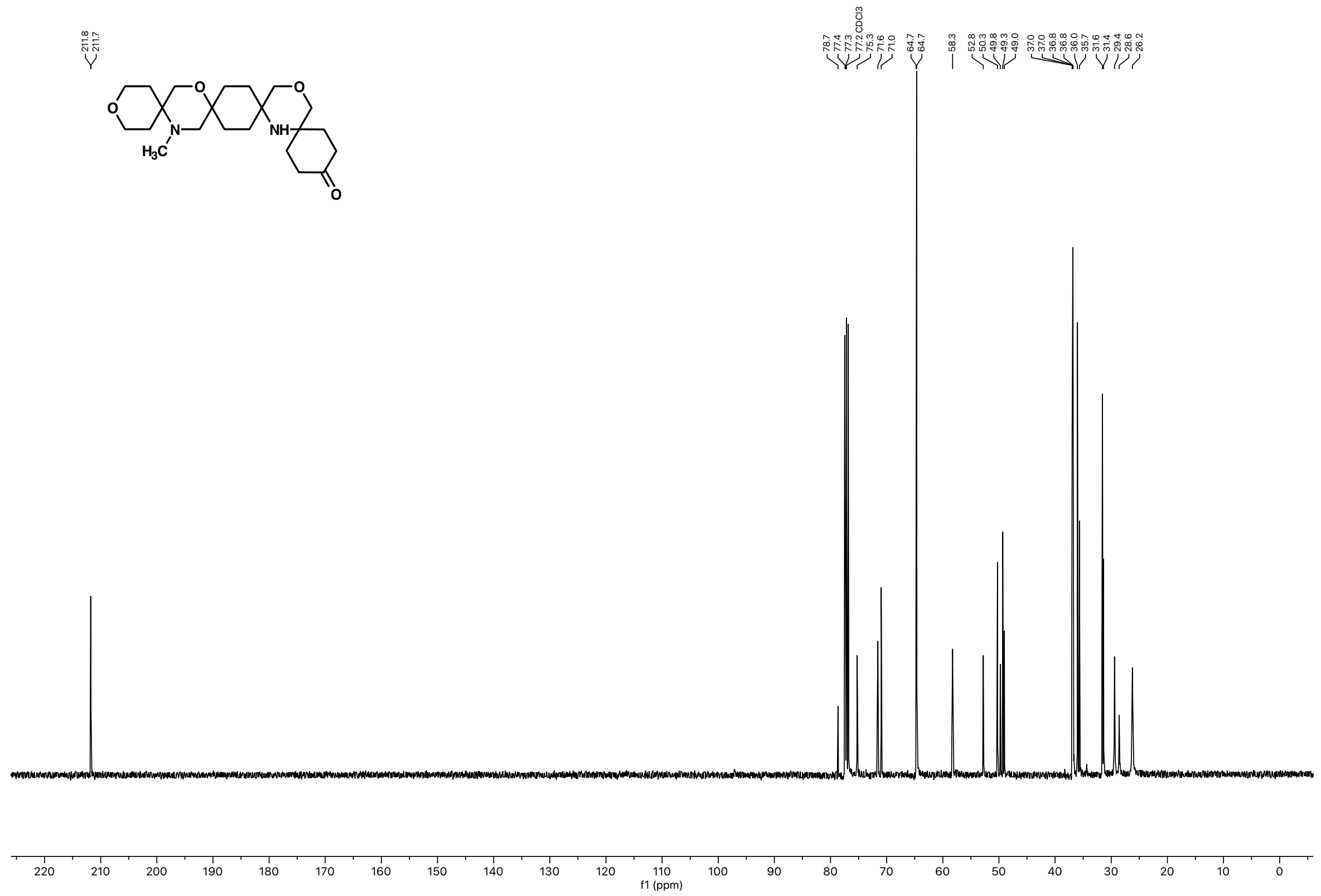

Compound 21: ¹H NMR ($CDCl_3$) top, ¹³C NMR ($CDCl_3$) bottom

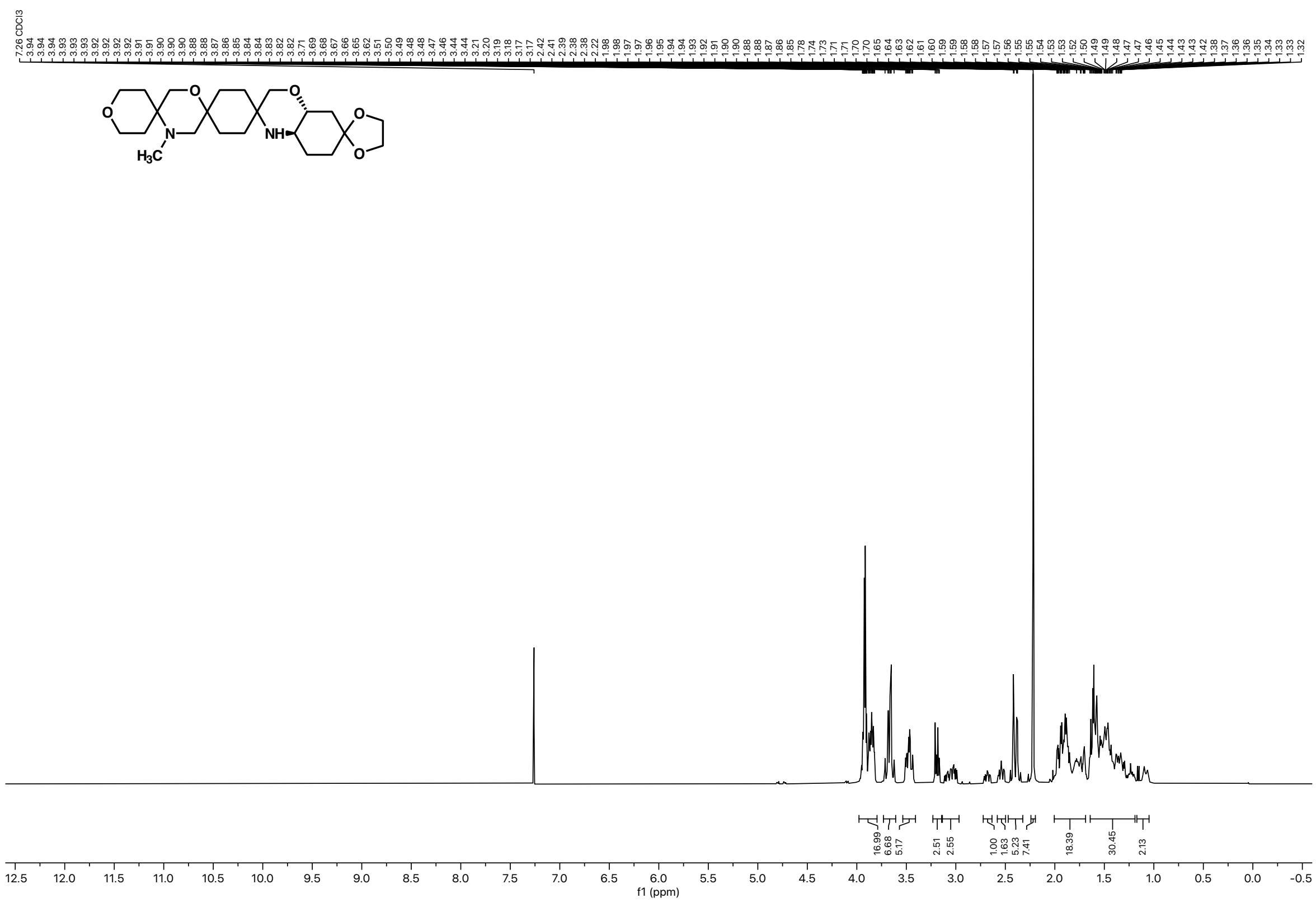

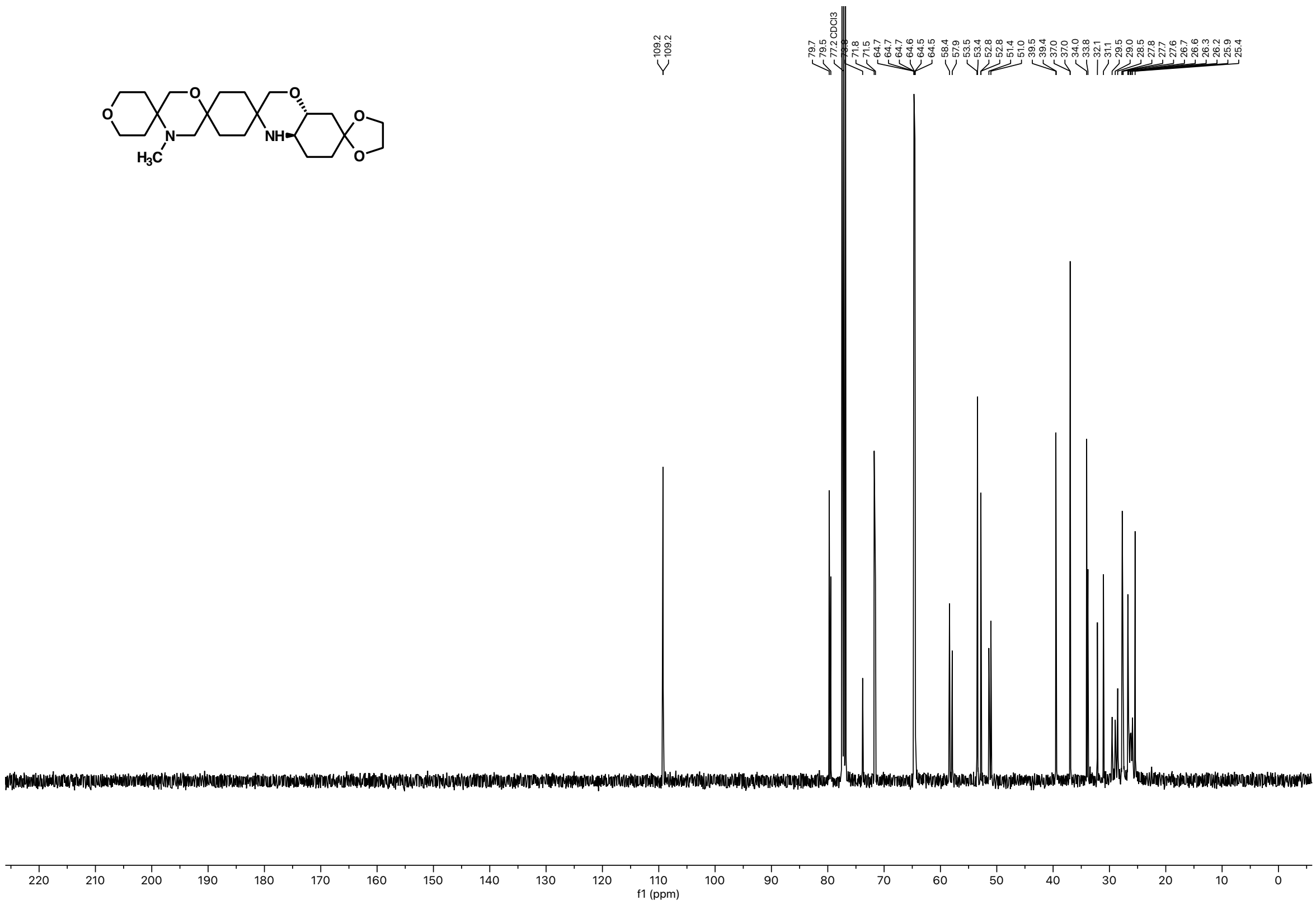

Compound 22: ^{1}H NMR ($CDCl_3$) top, ^{13}C NMR ($CDCl_3$) bottom

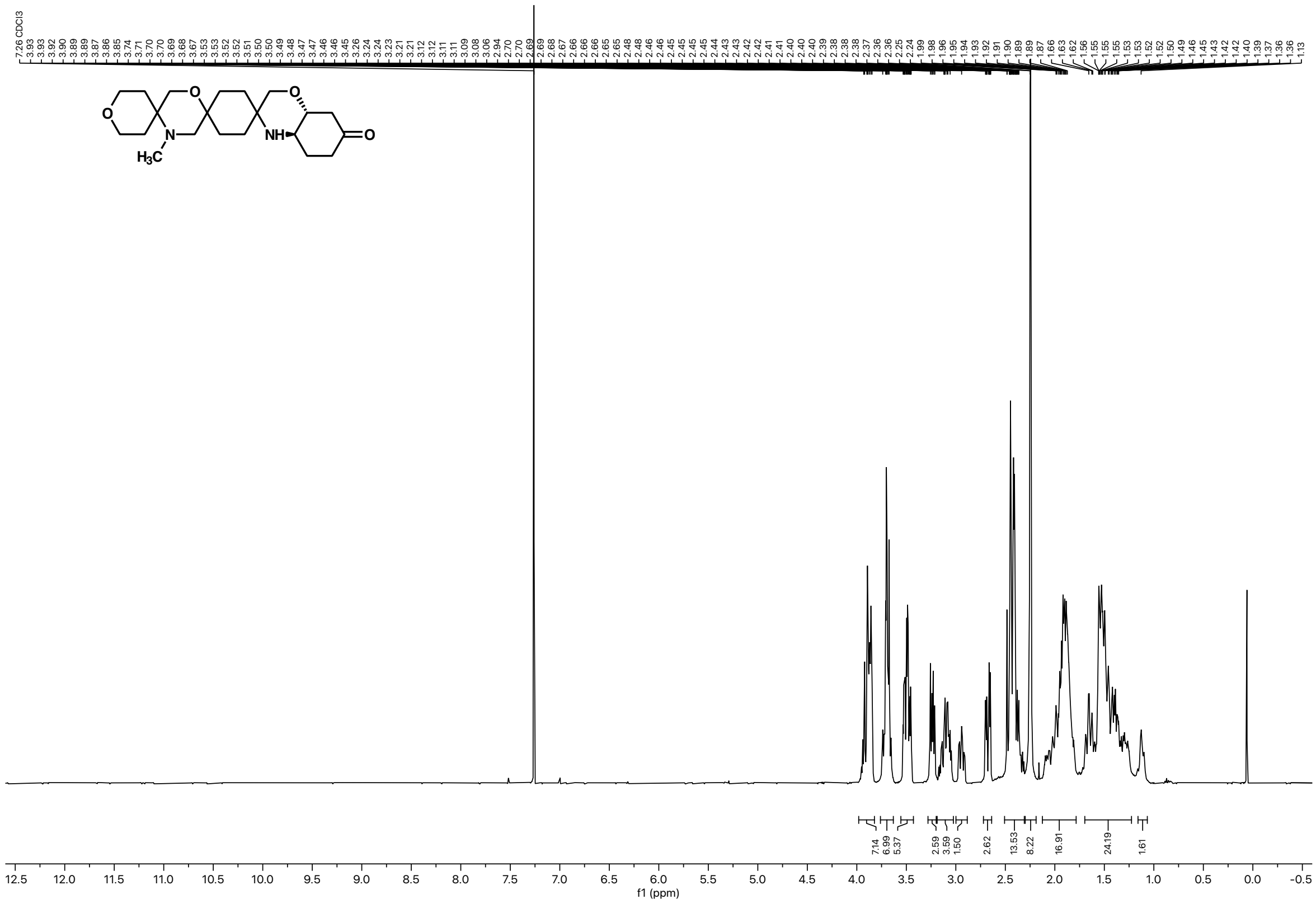

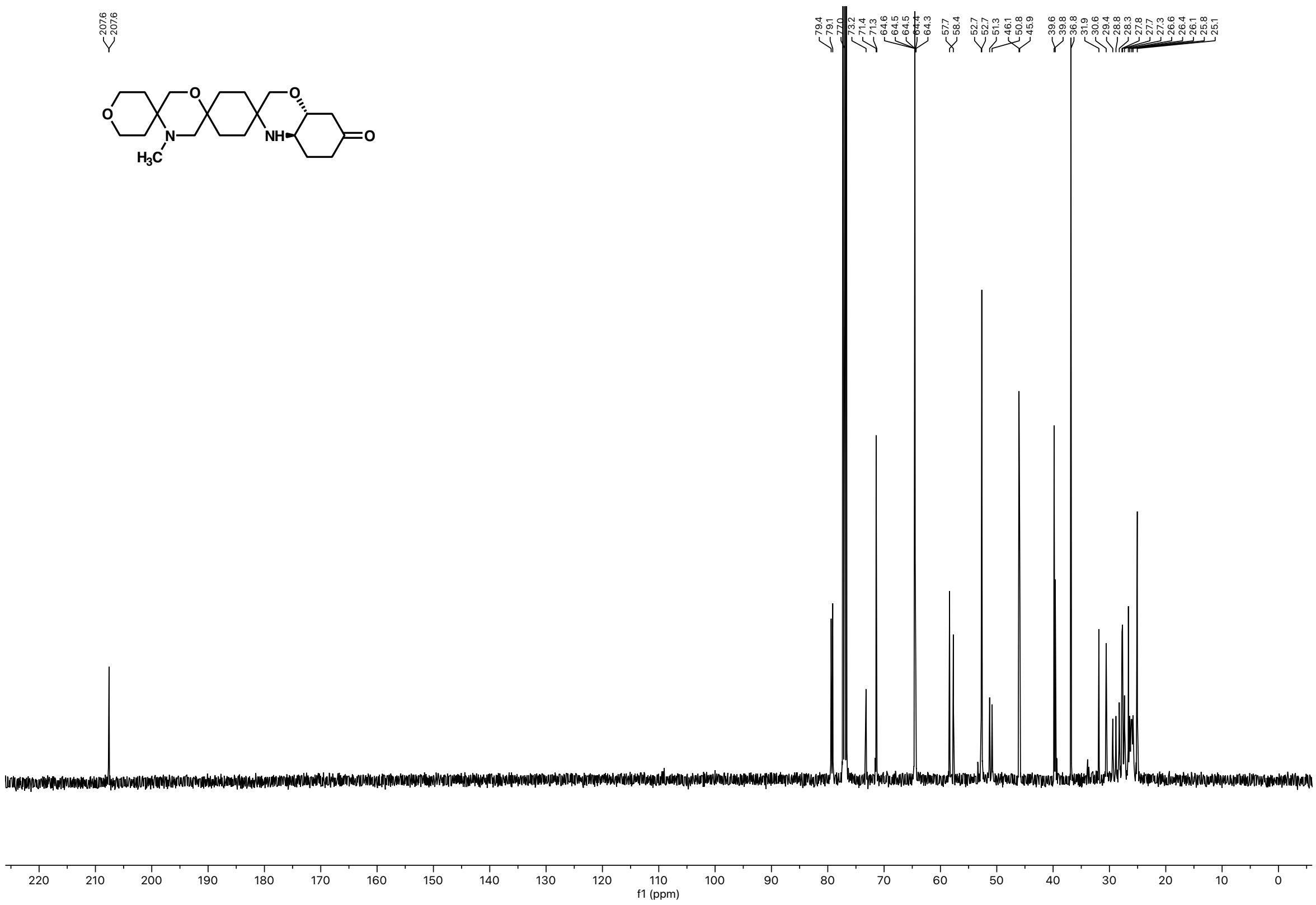

Compound 23: ¹H NMR ($CDCl_3$) top, ¹³C NMR ($CDCl_3$) bottom

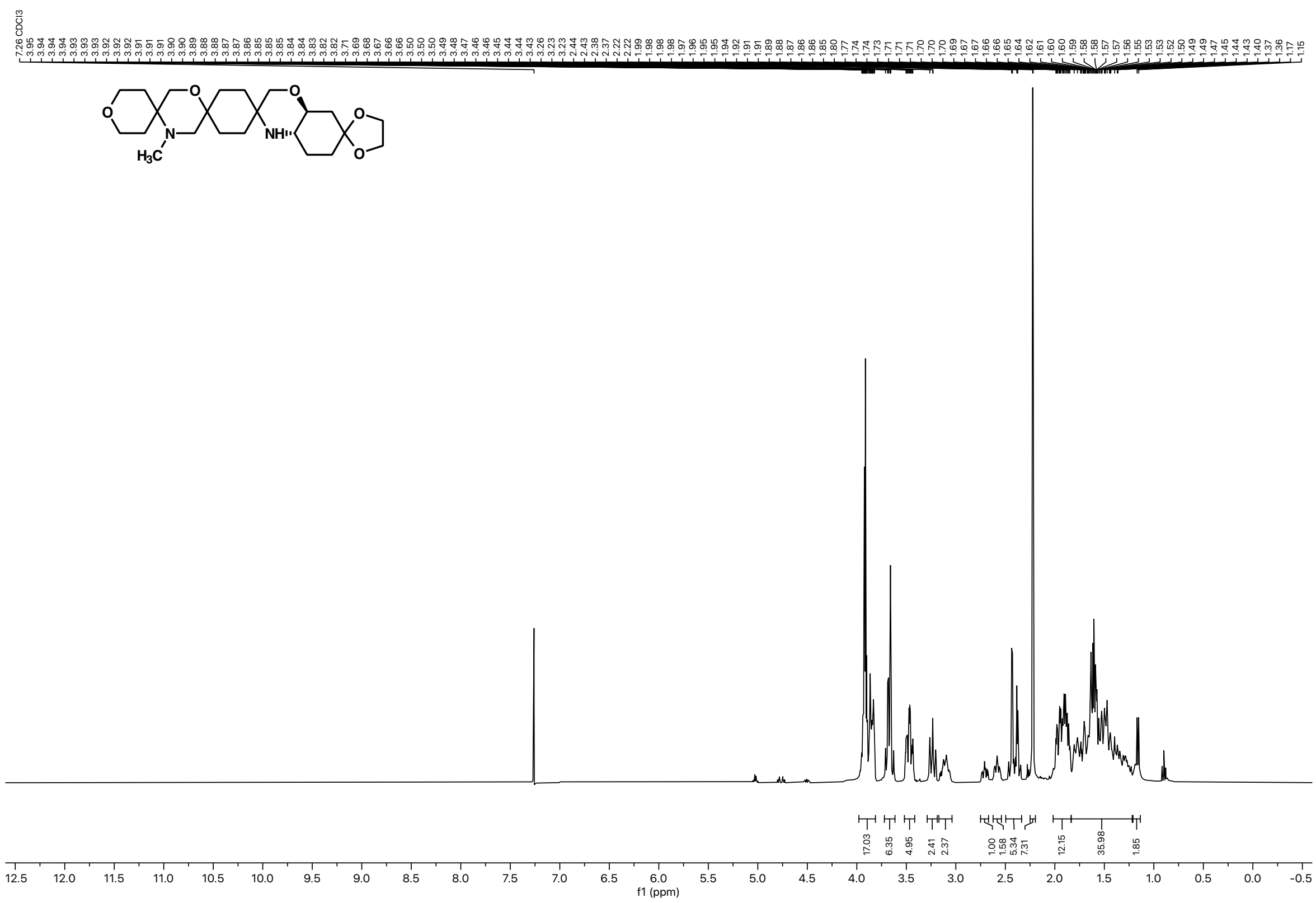

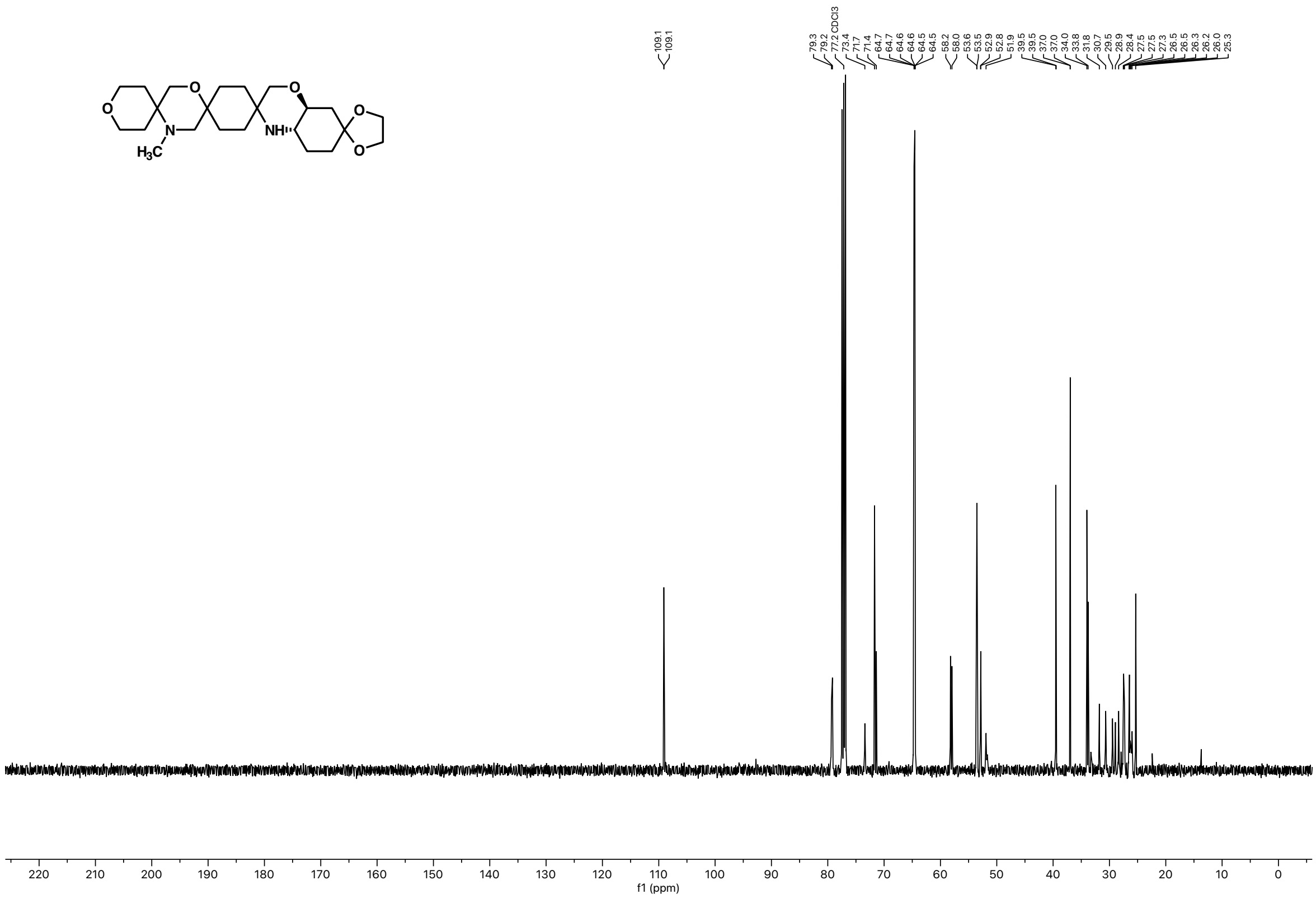

Compound 24: ^{1}H NMR ($CDCl_3$) top, ^{13}C NMR ($CDCl_3$) bottom

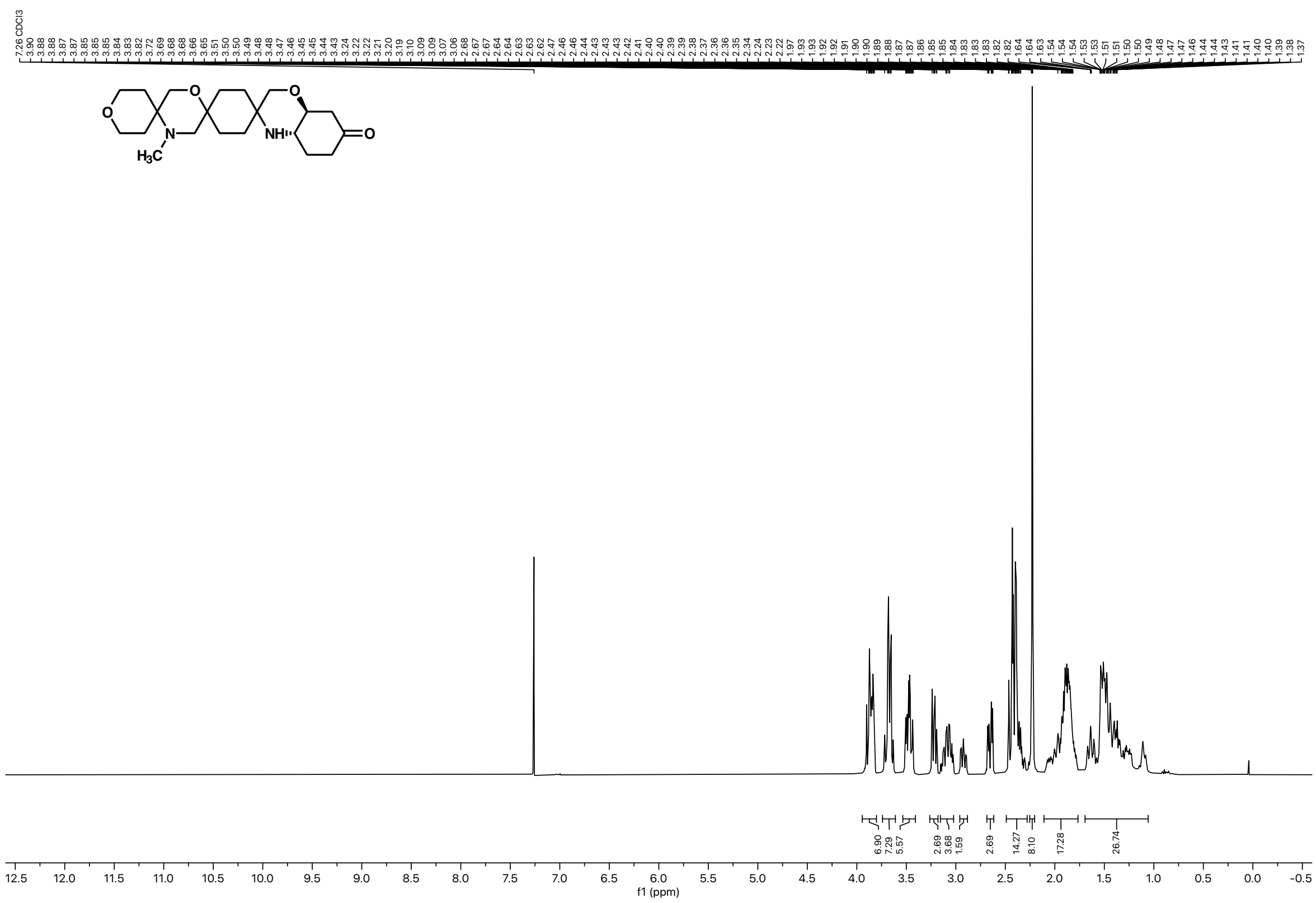

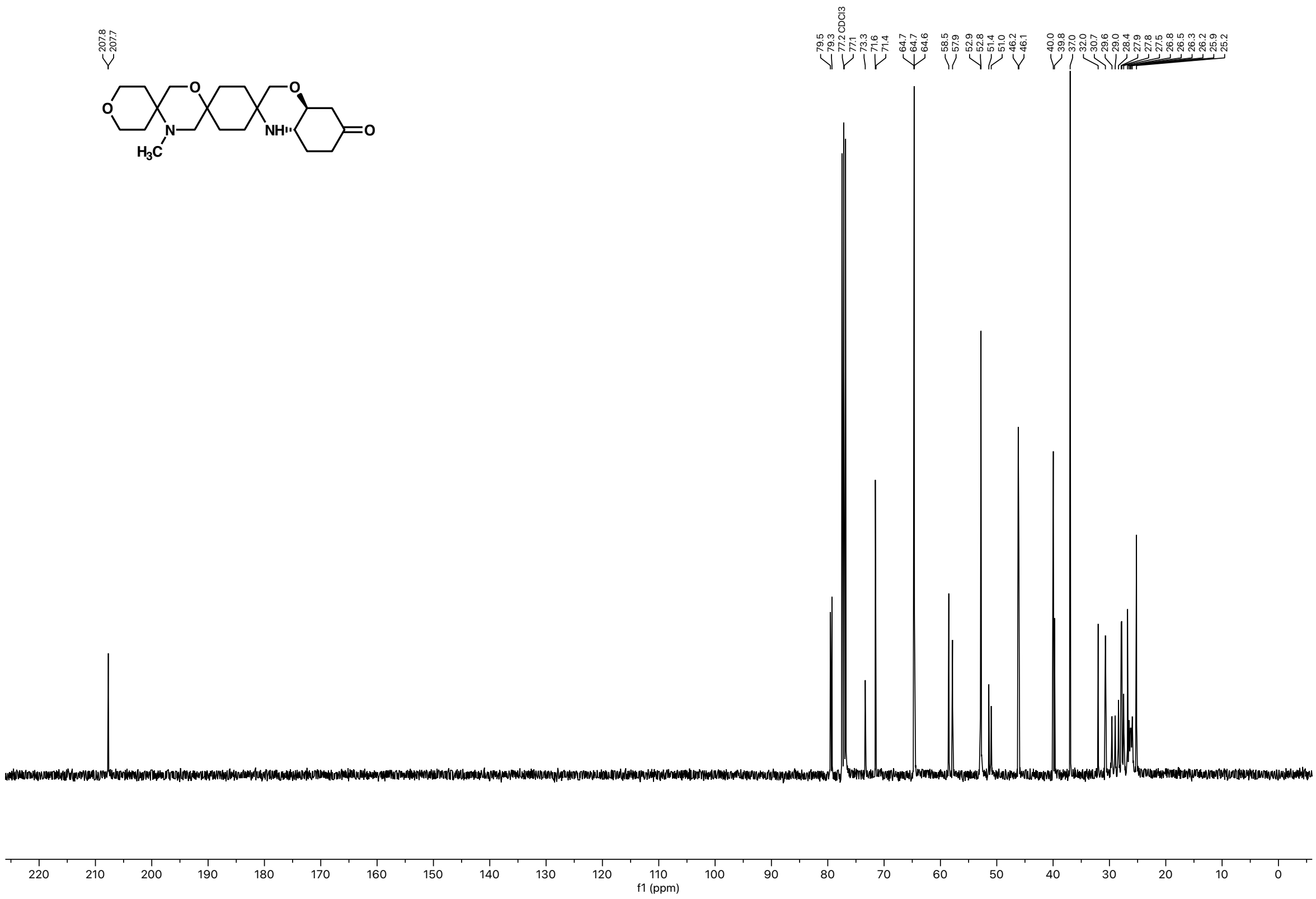

Compound 25: ^{1}H NMR ($CDCl_3$) top, ^{13}C NMR ($CDCl_3$) bottom

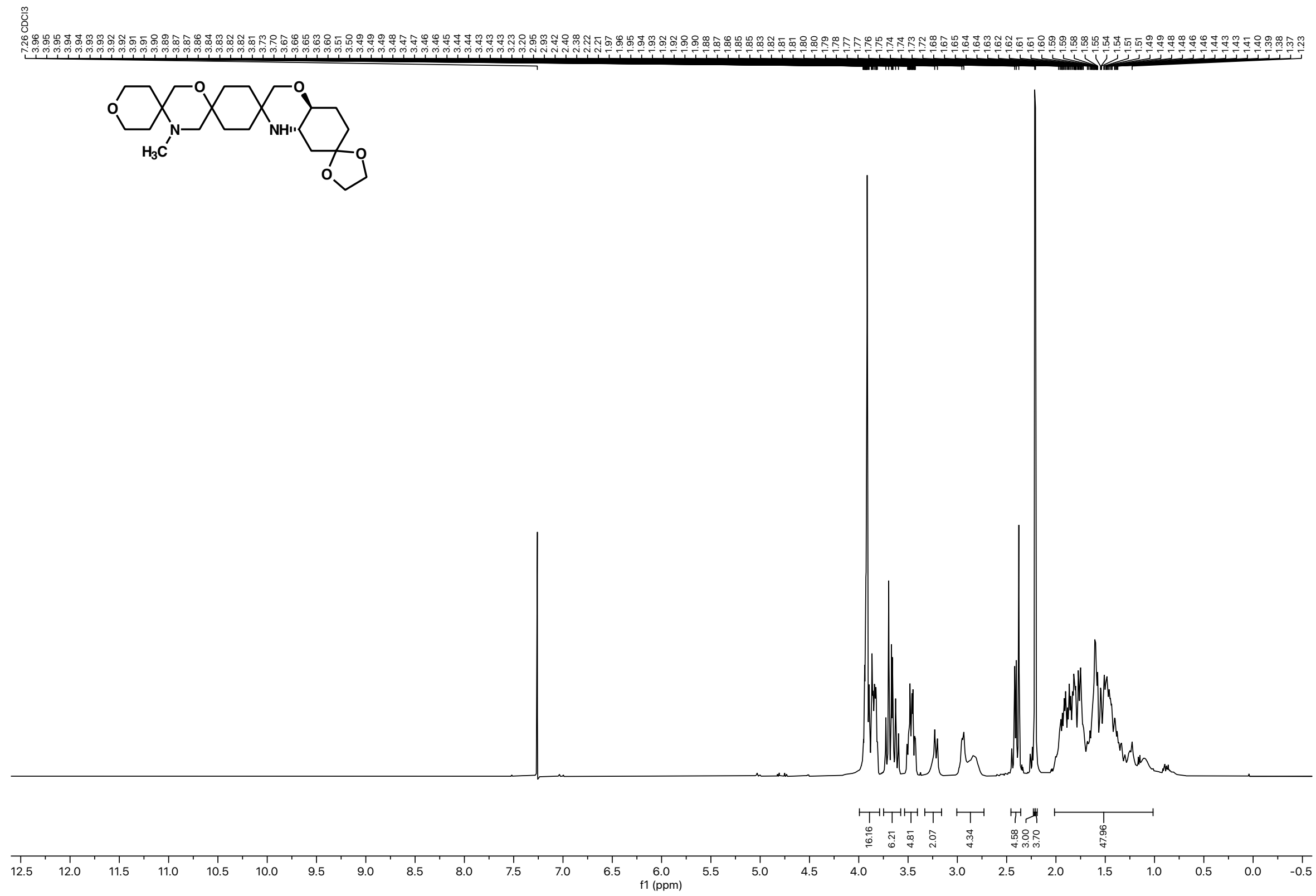

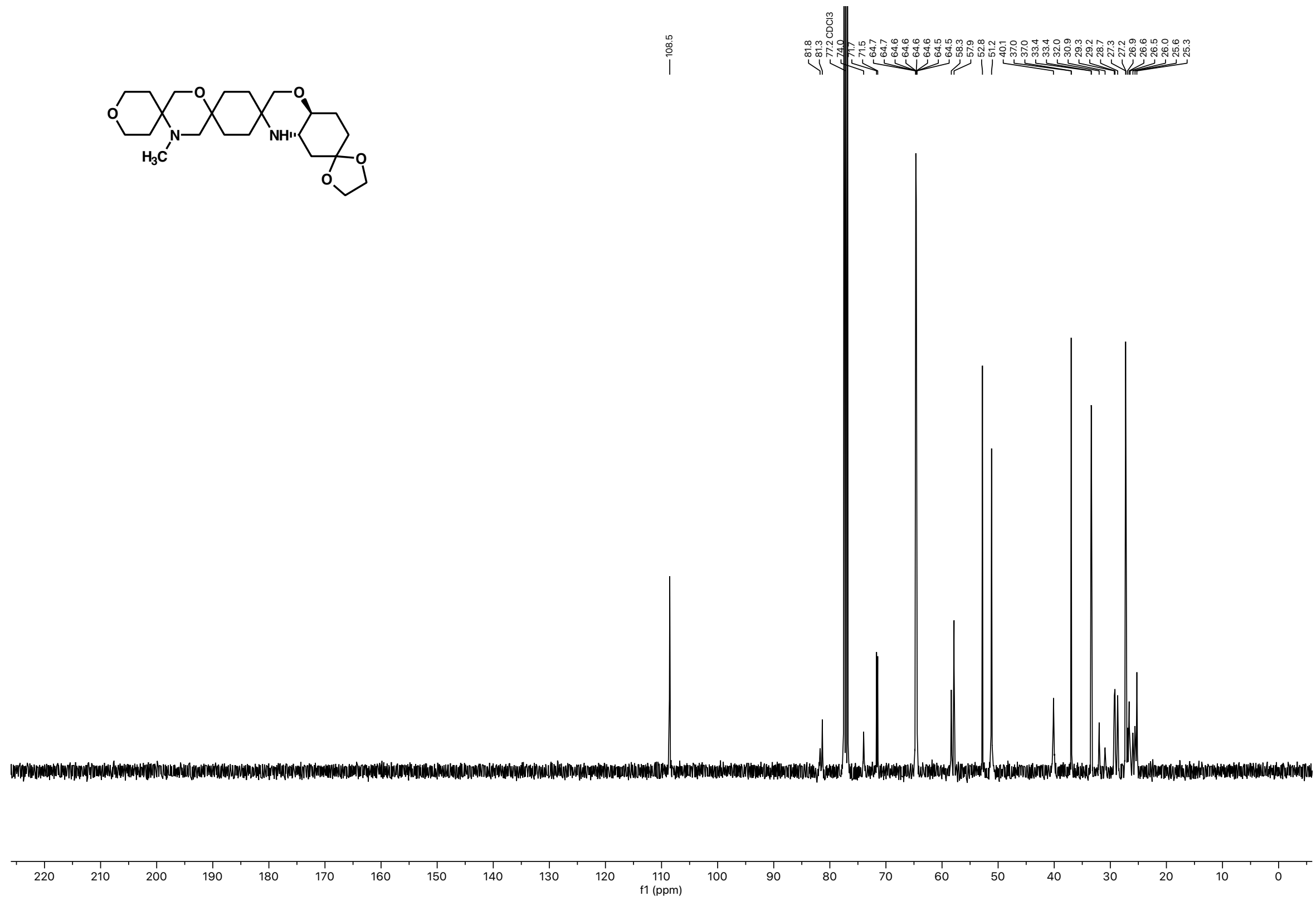

Compound 26: ^{1}H NMR ($CDCl_3$) top, ^{13}C NMR ($CDCl_3$) bottom

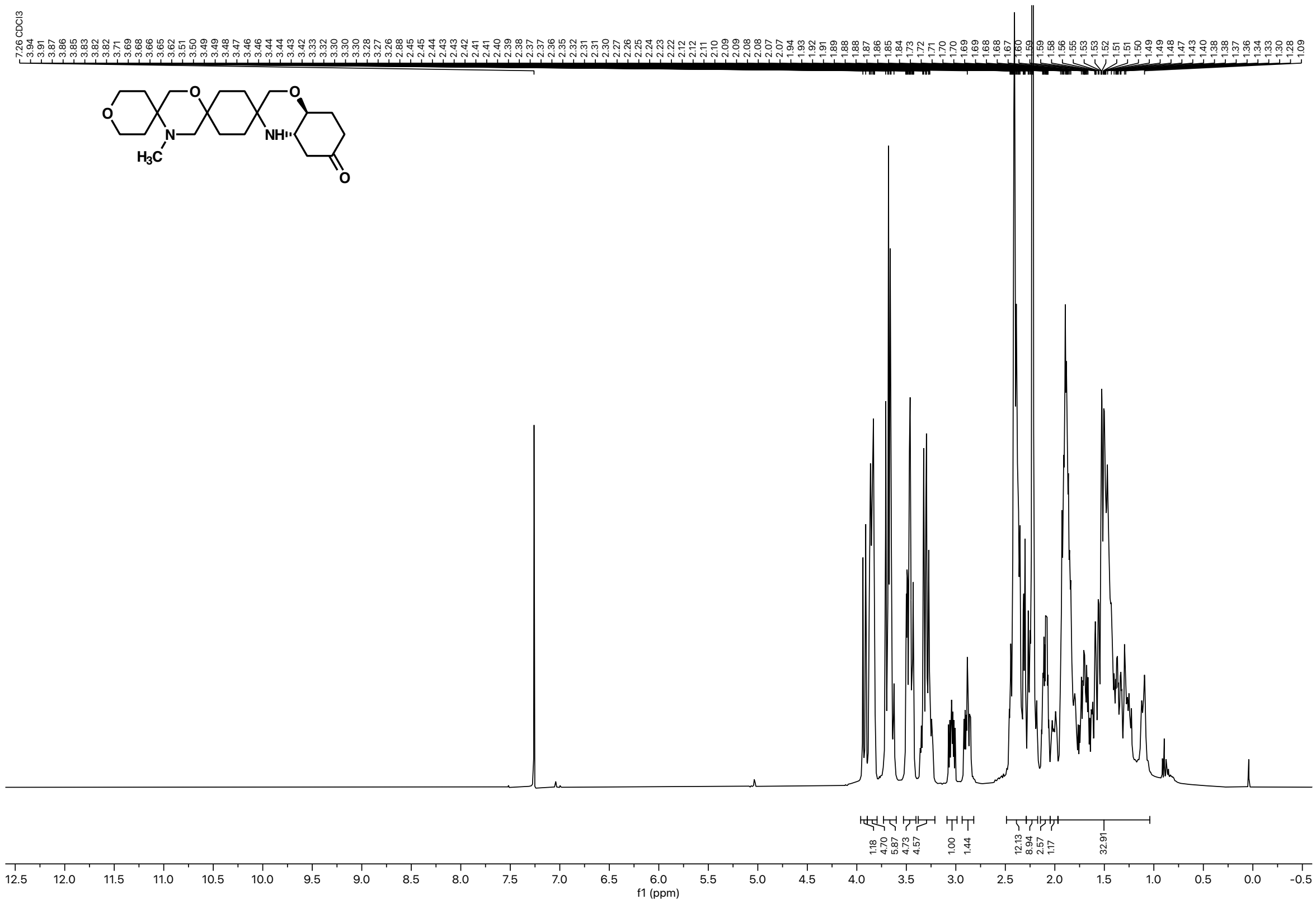

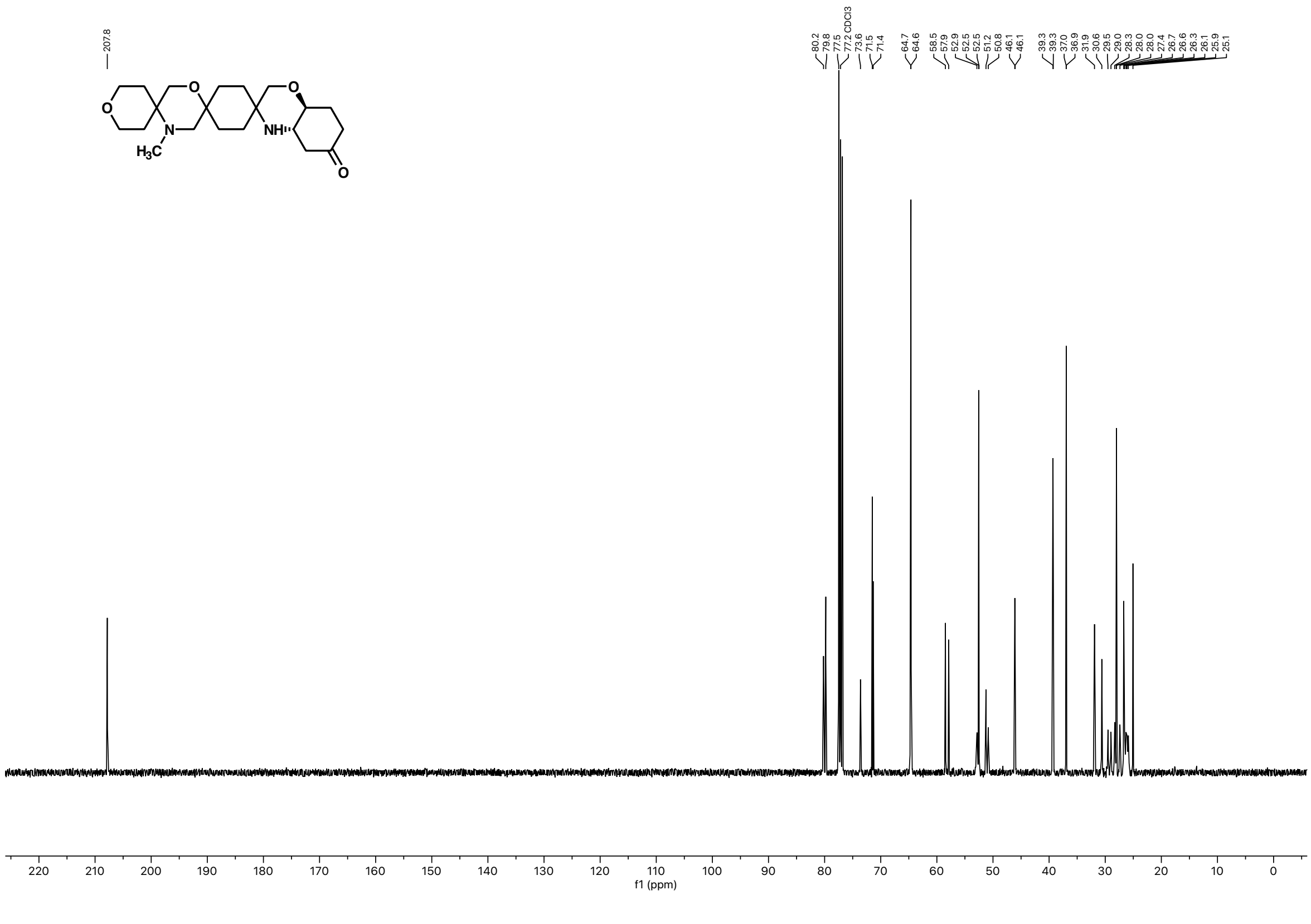

Compound 27: ^{1}H NMR ($CDCl_3$) top, ^{13}C NMR ($CDCl_3$) bottom

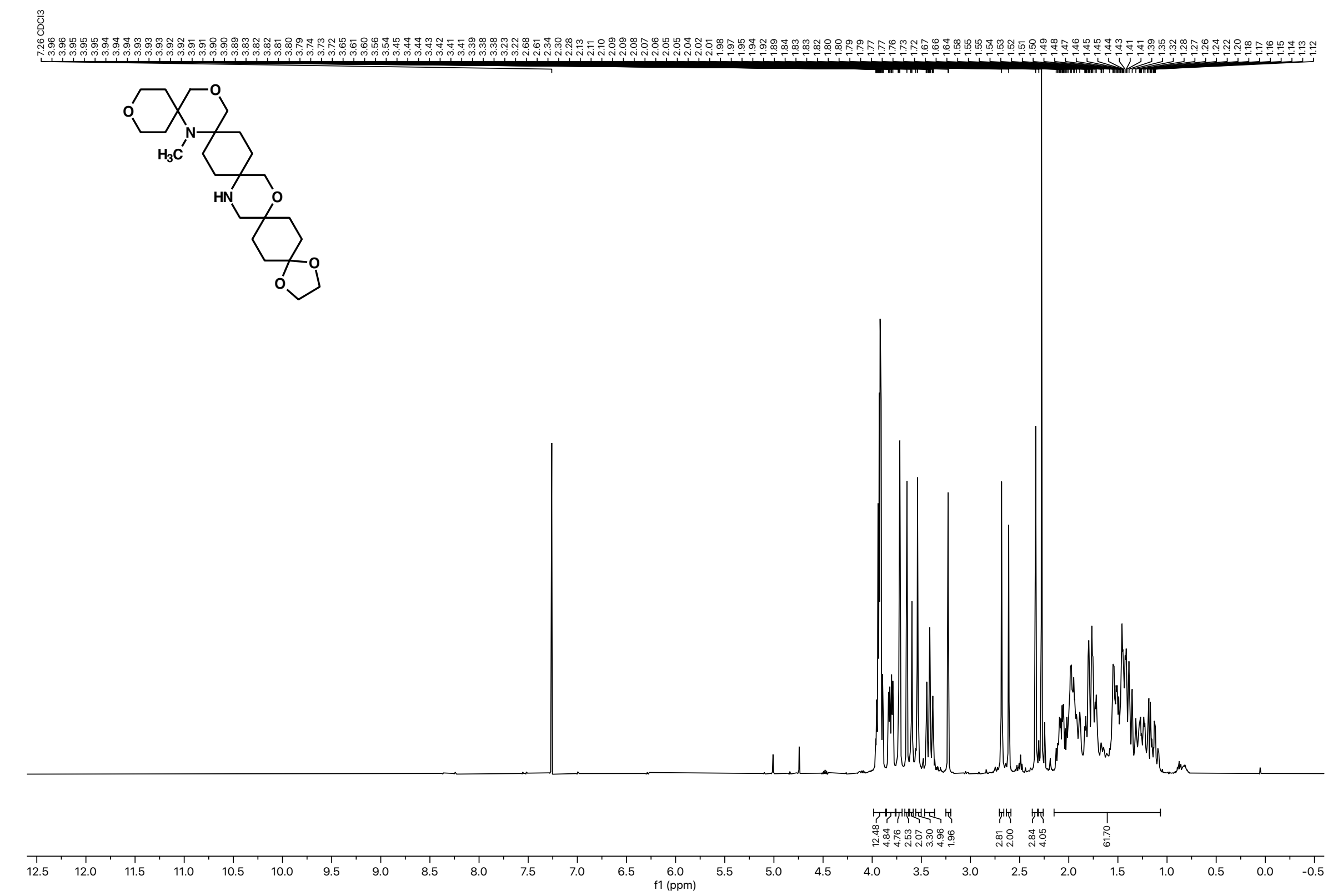

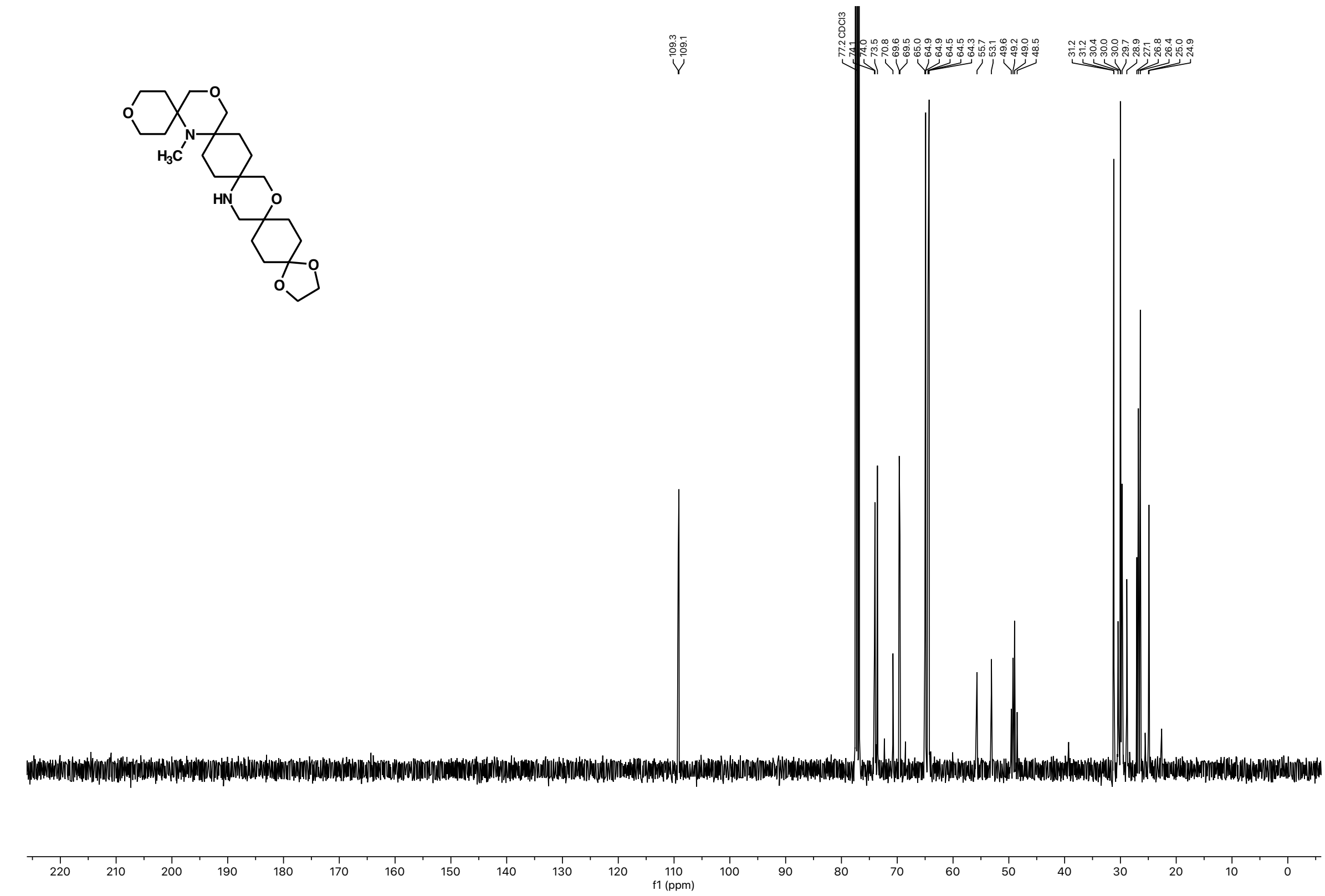

Compound 28: ^{1}H NMR ($CDCl_3$) top, ^{13}C NMR ($CDCl_3$) bottom

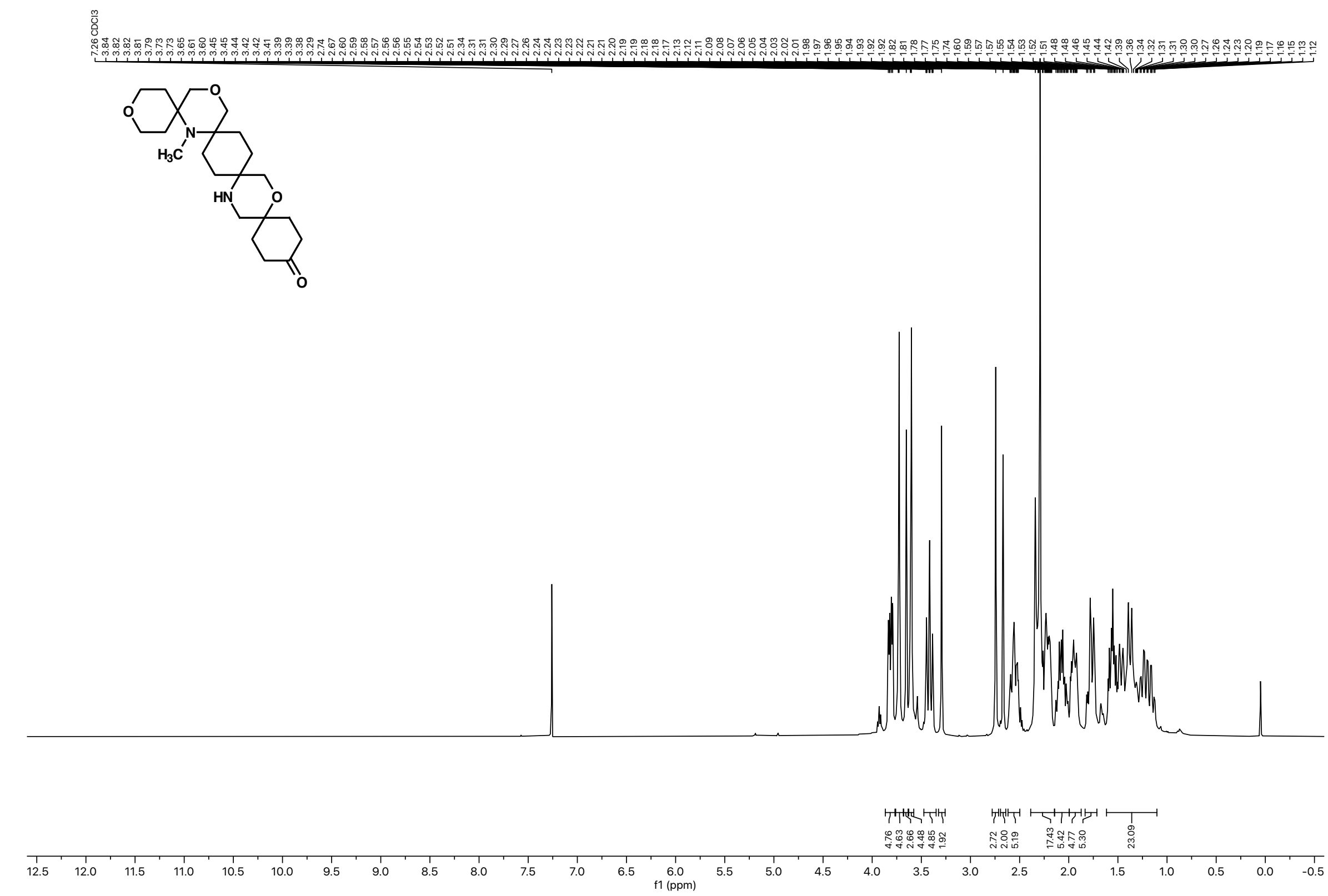

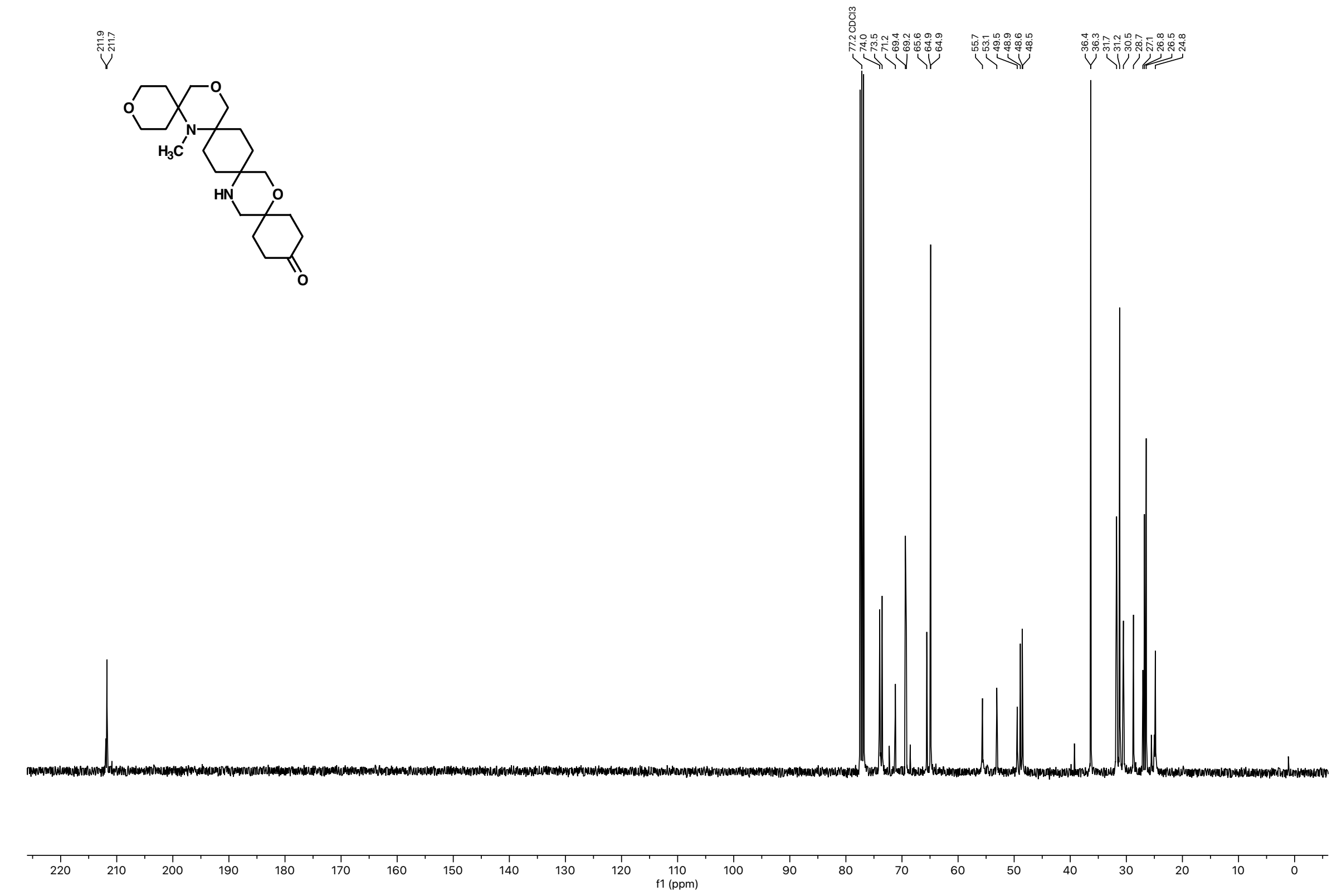

Compound 29: ^{1}H NMR ($CDCl_3$) top, ^{13}C NMR ($CDCl_3$) bottom

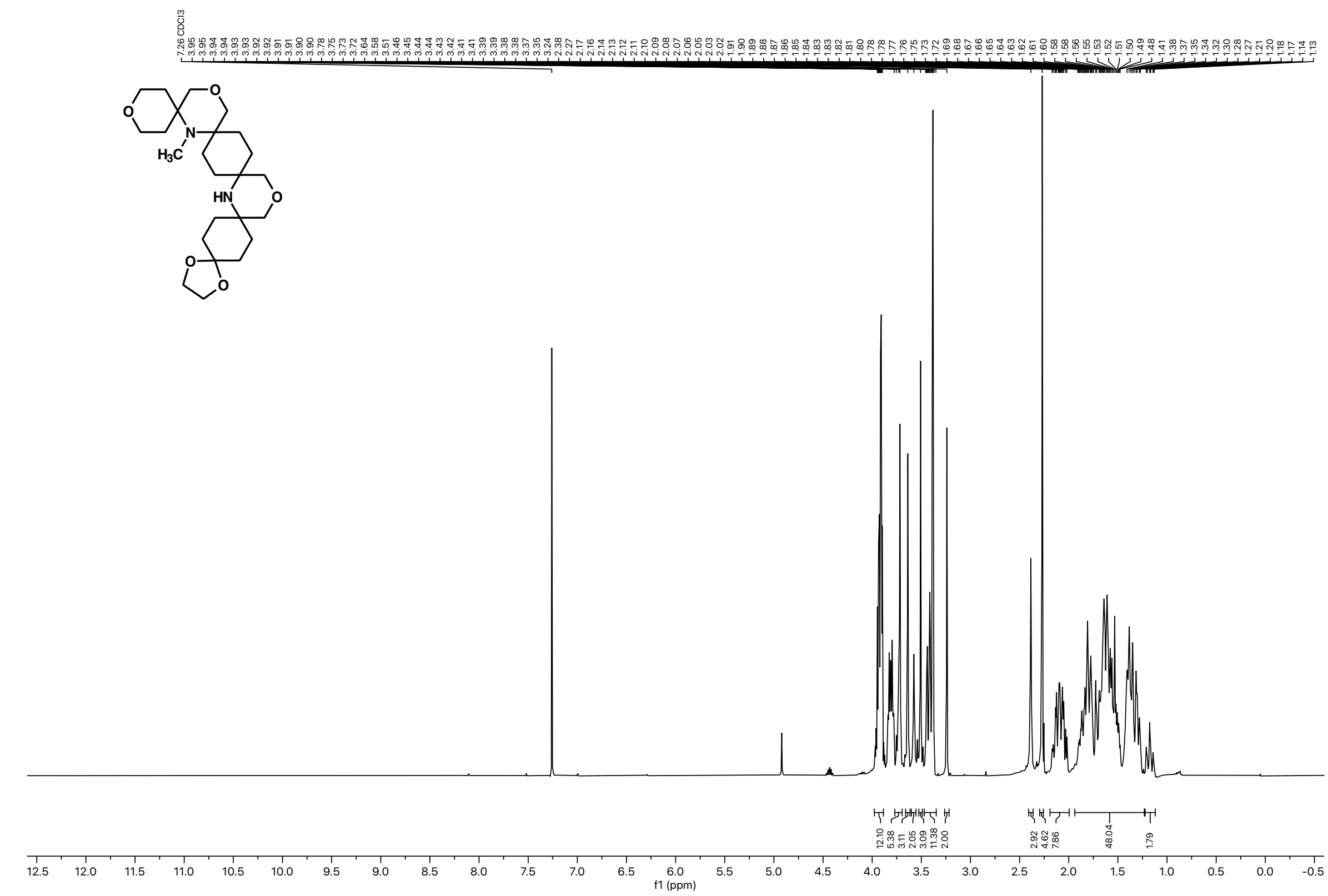

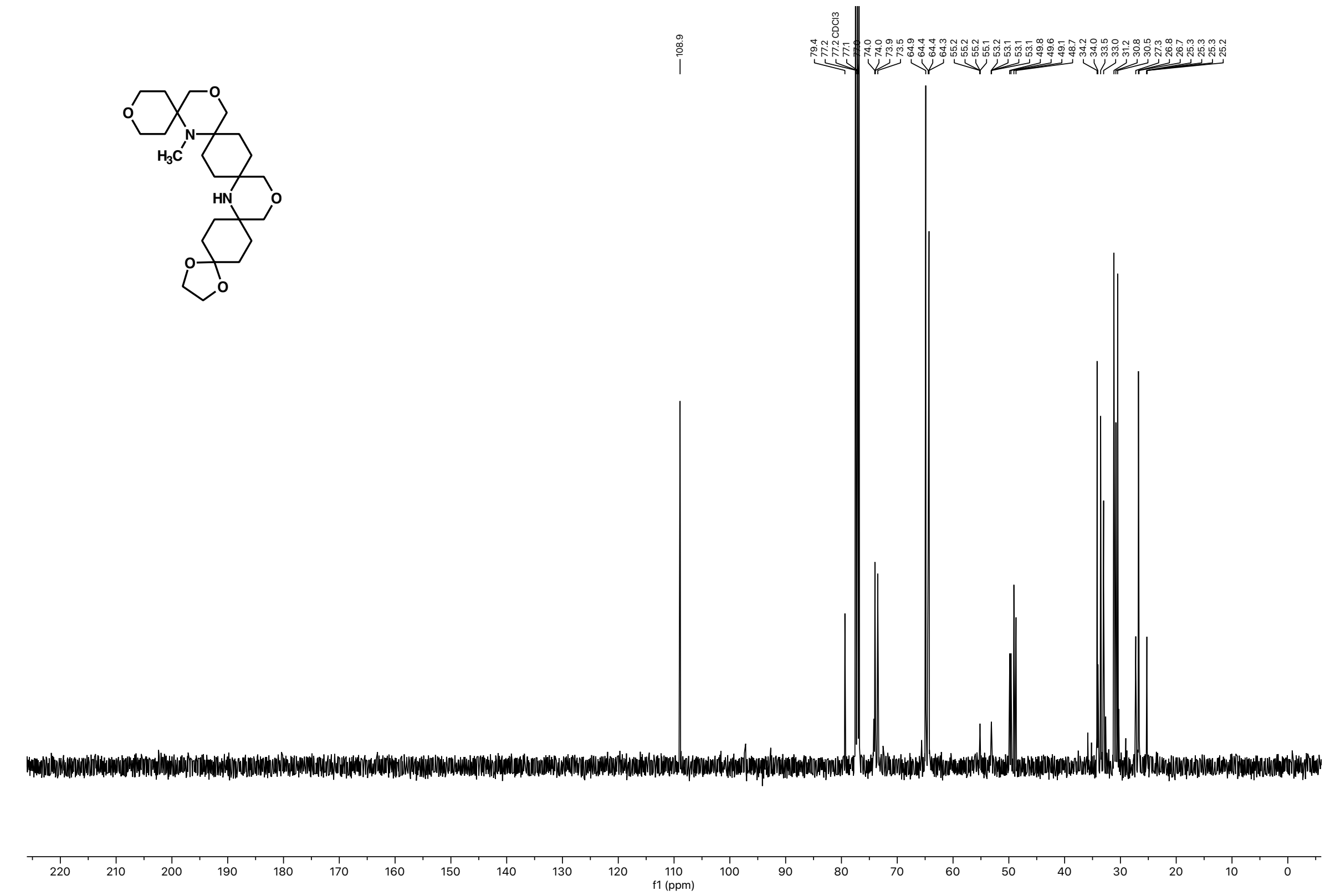

Compound 30: ^{1}H NMR ($CDCl_3$) top, ^{13}C NMR ($CDCl_3$) bottom

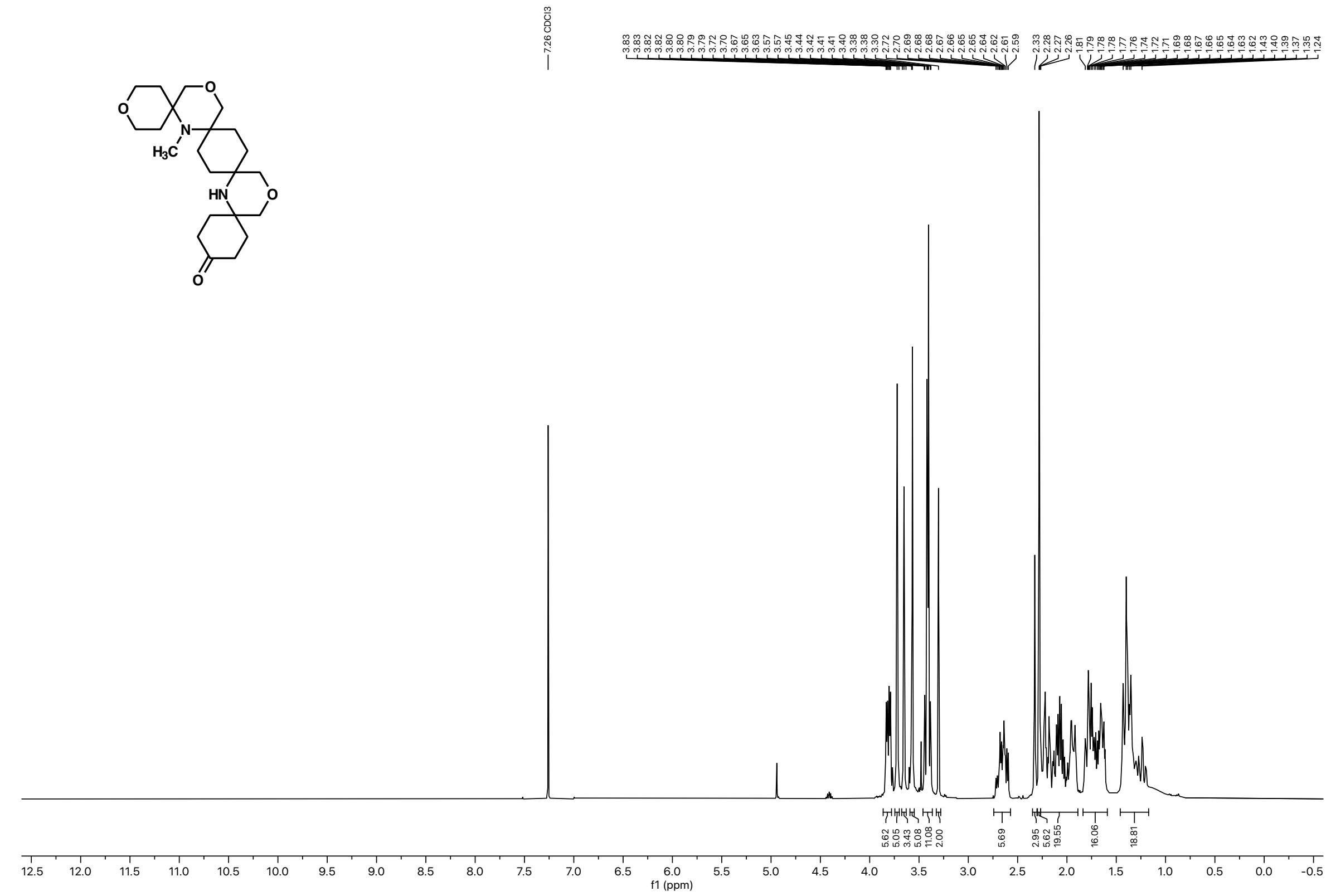

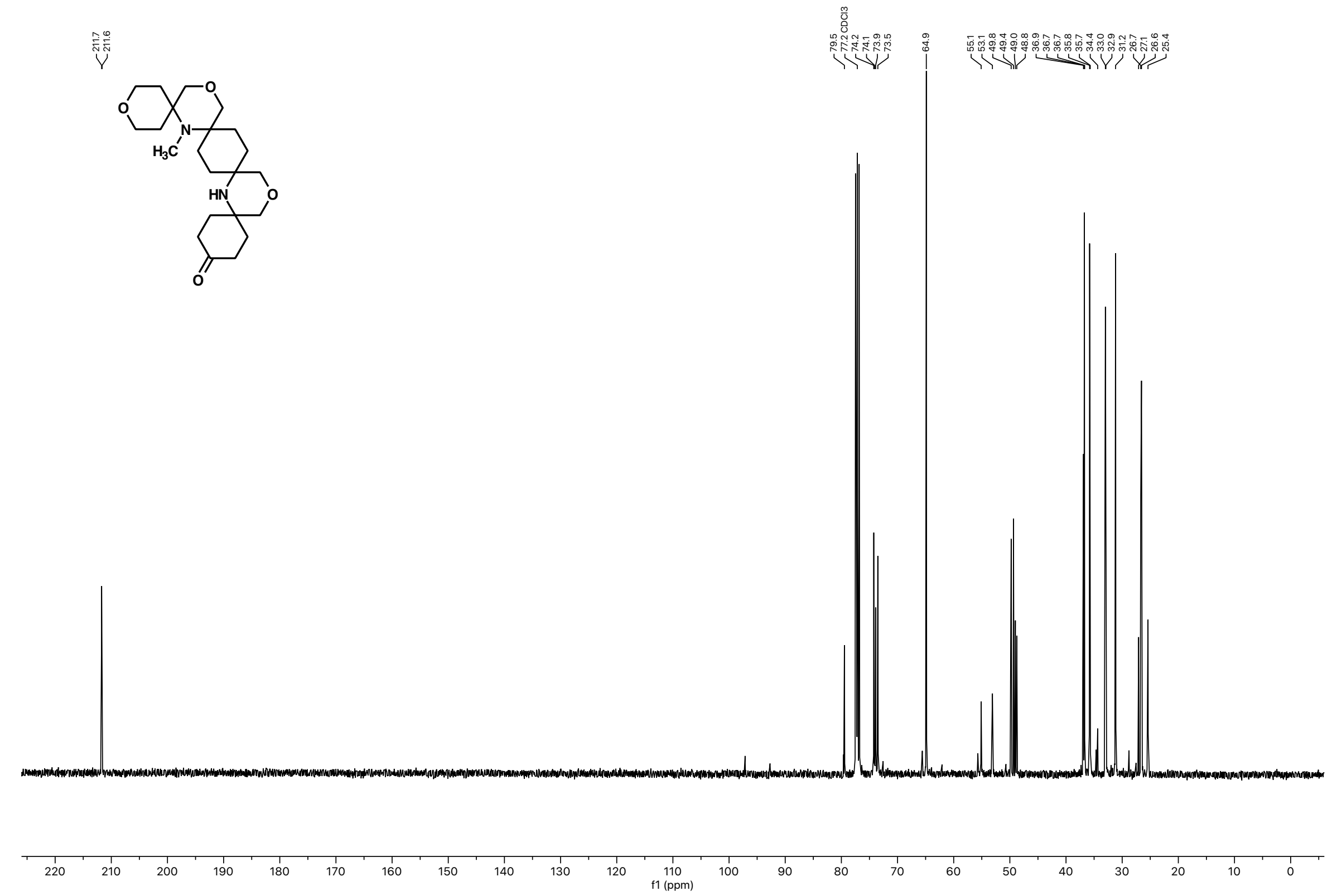

Compound 31: ^{1}H NMR ($CDCl_3$) top, ^{13}C NMR ($CDCl_3$) bottom

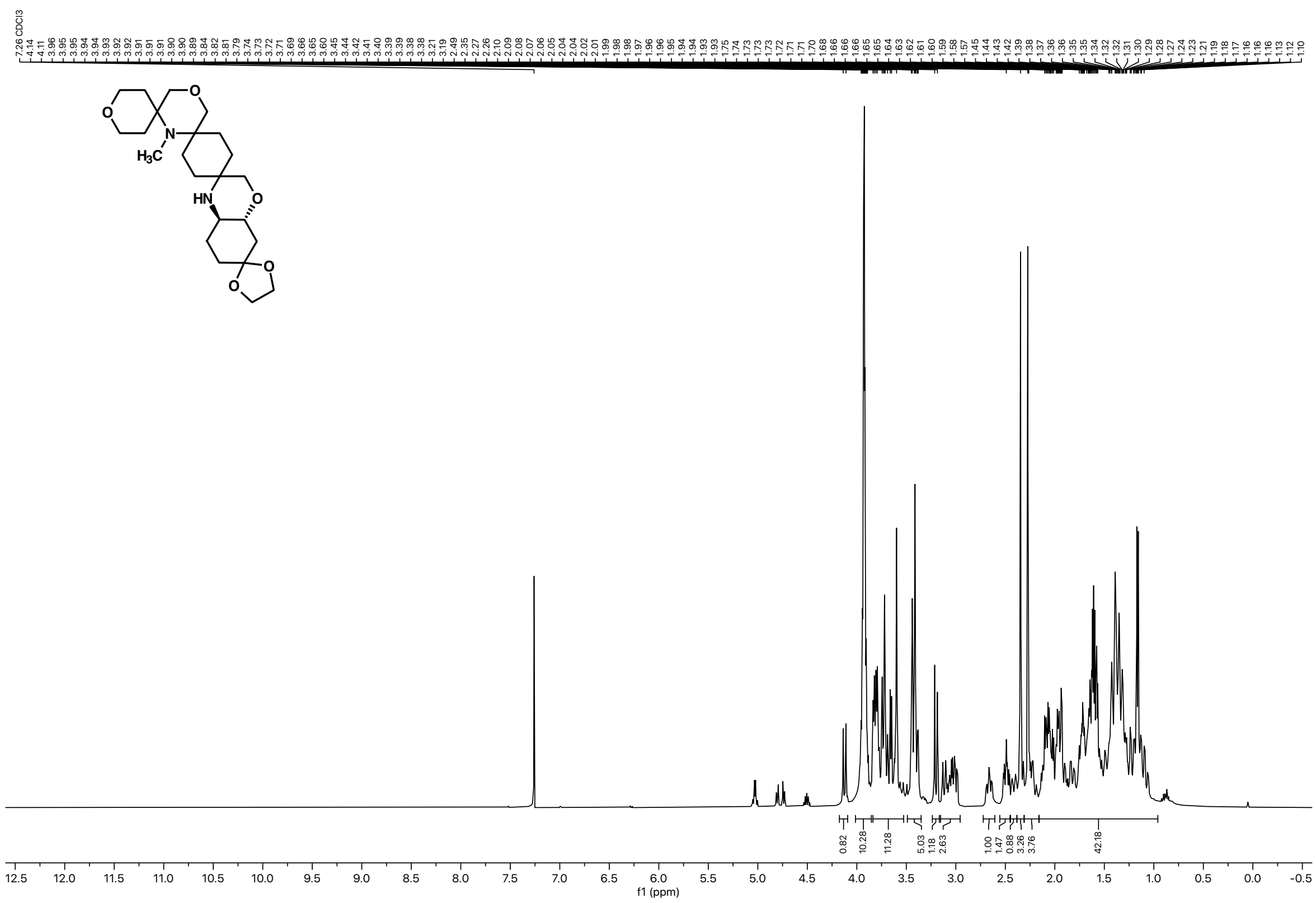

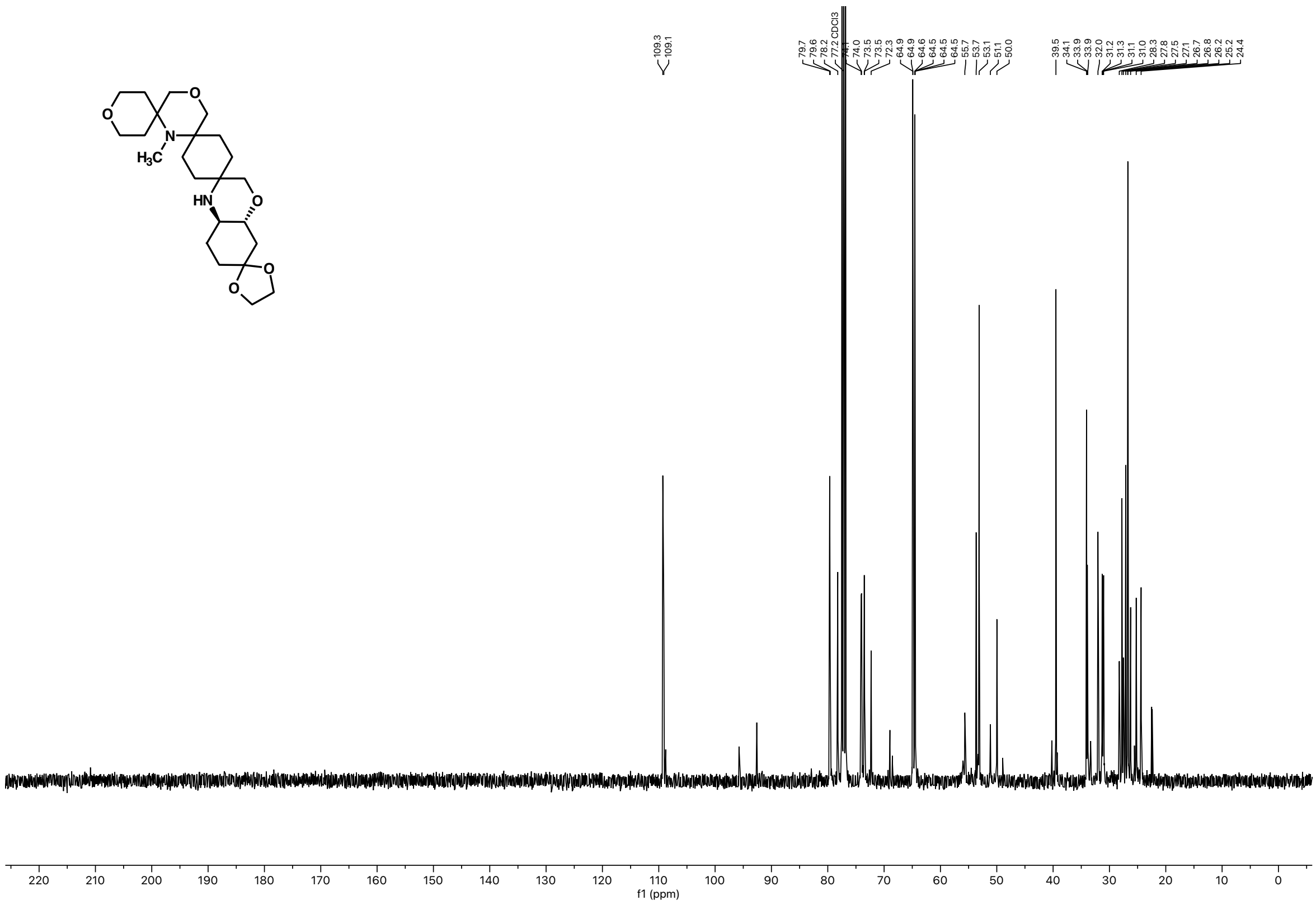

Compound 32: ¹H NMR ($CDCl_3$) top, ¹³C NMR ($CDCl_3$) bottom

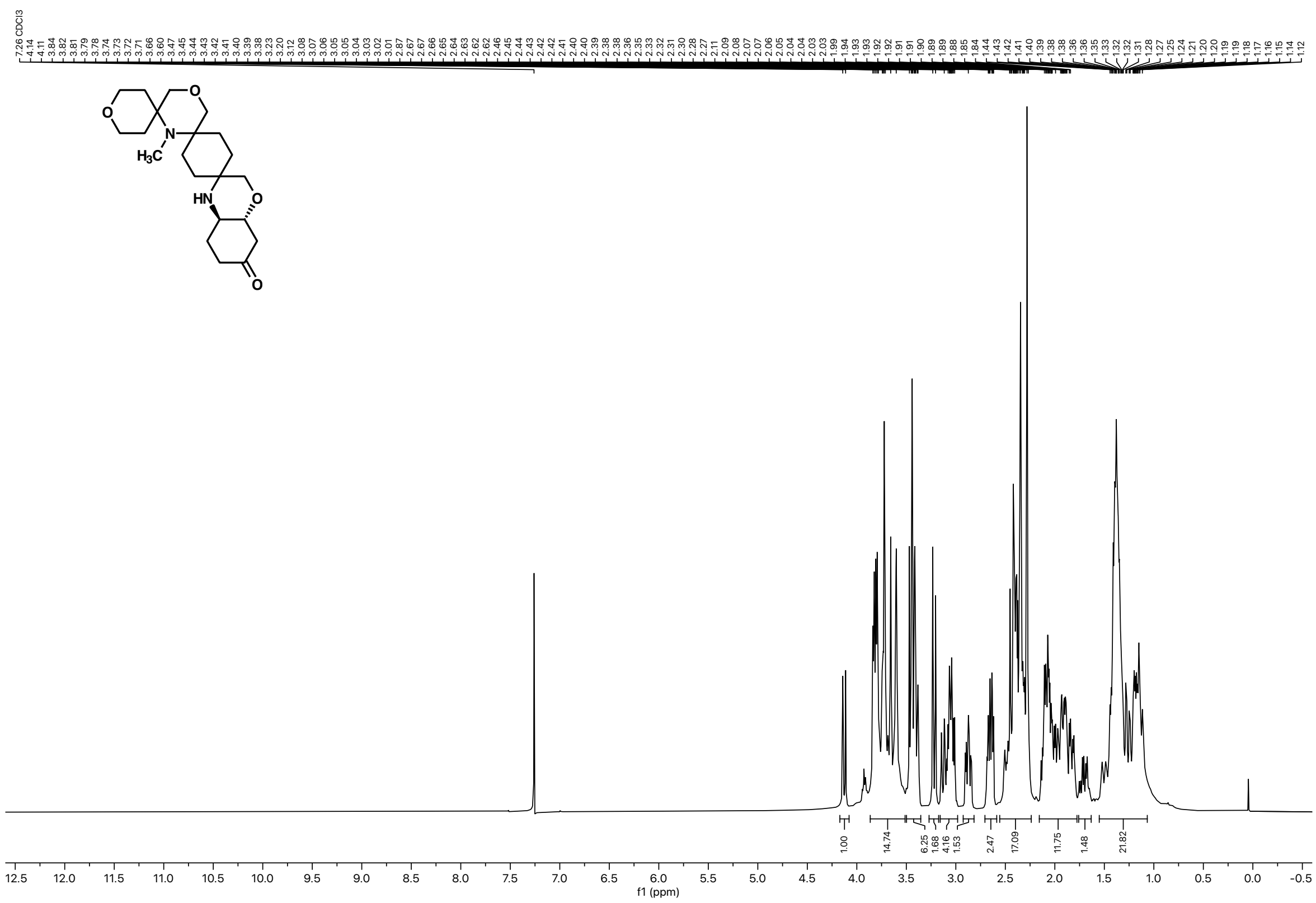

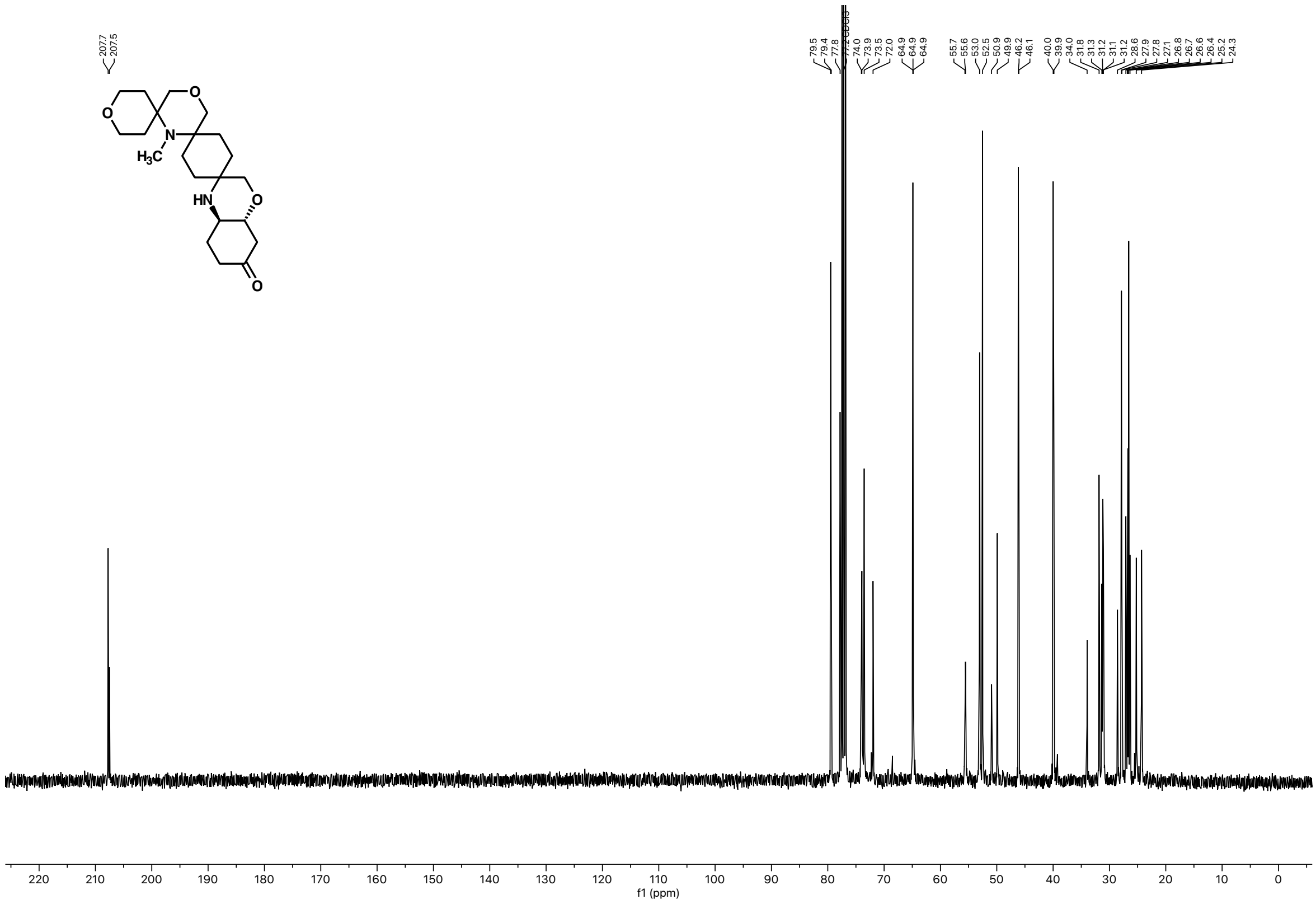

Compound 33: ^{1}H NMR ($CDCl_3$) top, ^{13}C NMR ($CDCl_3$) bottom

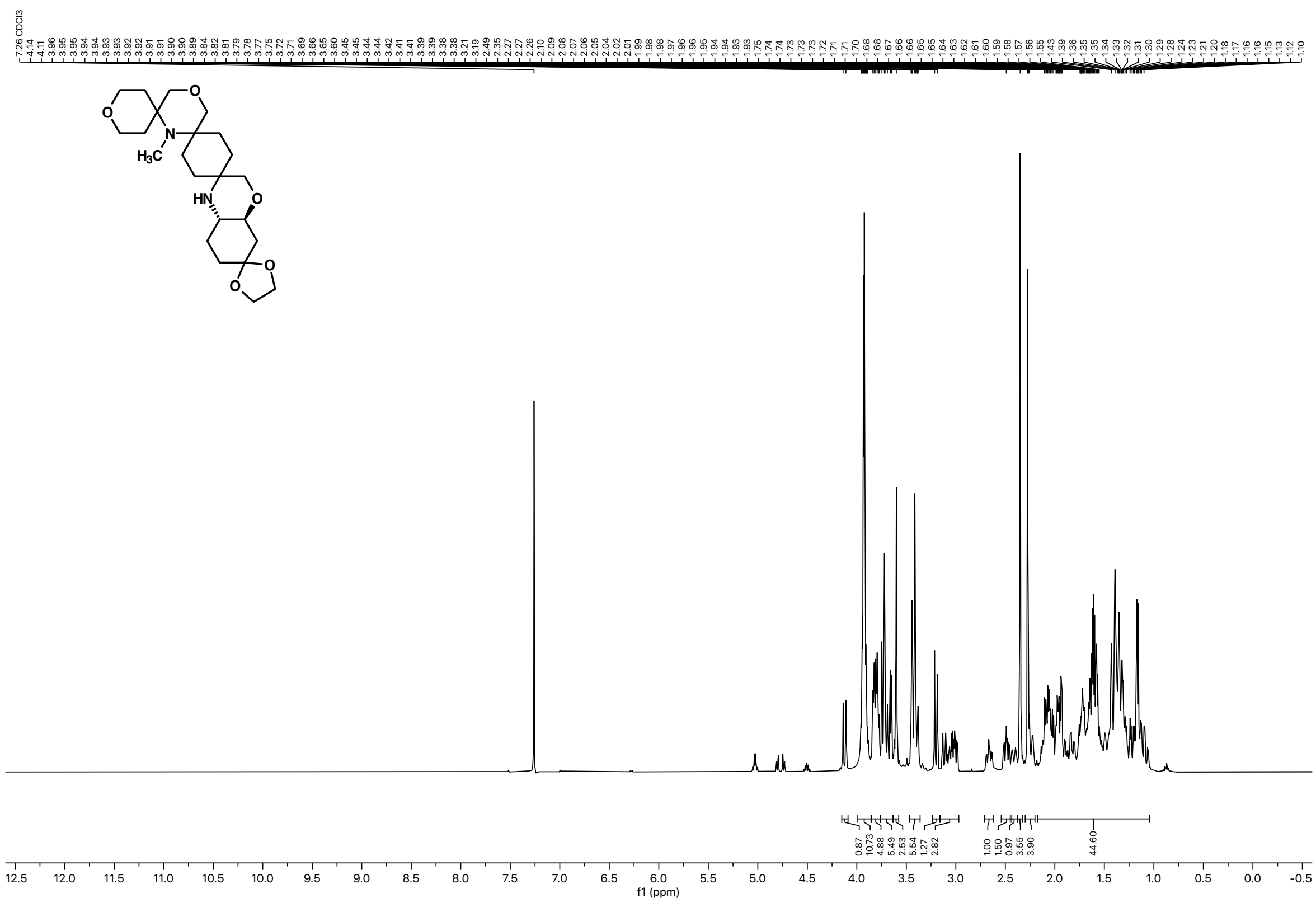

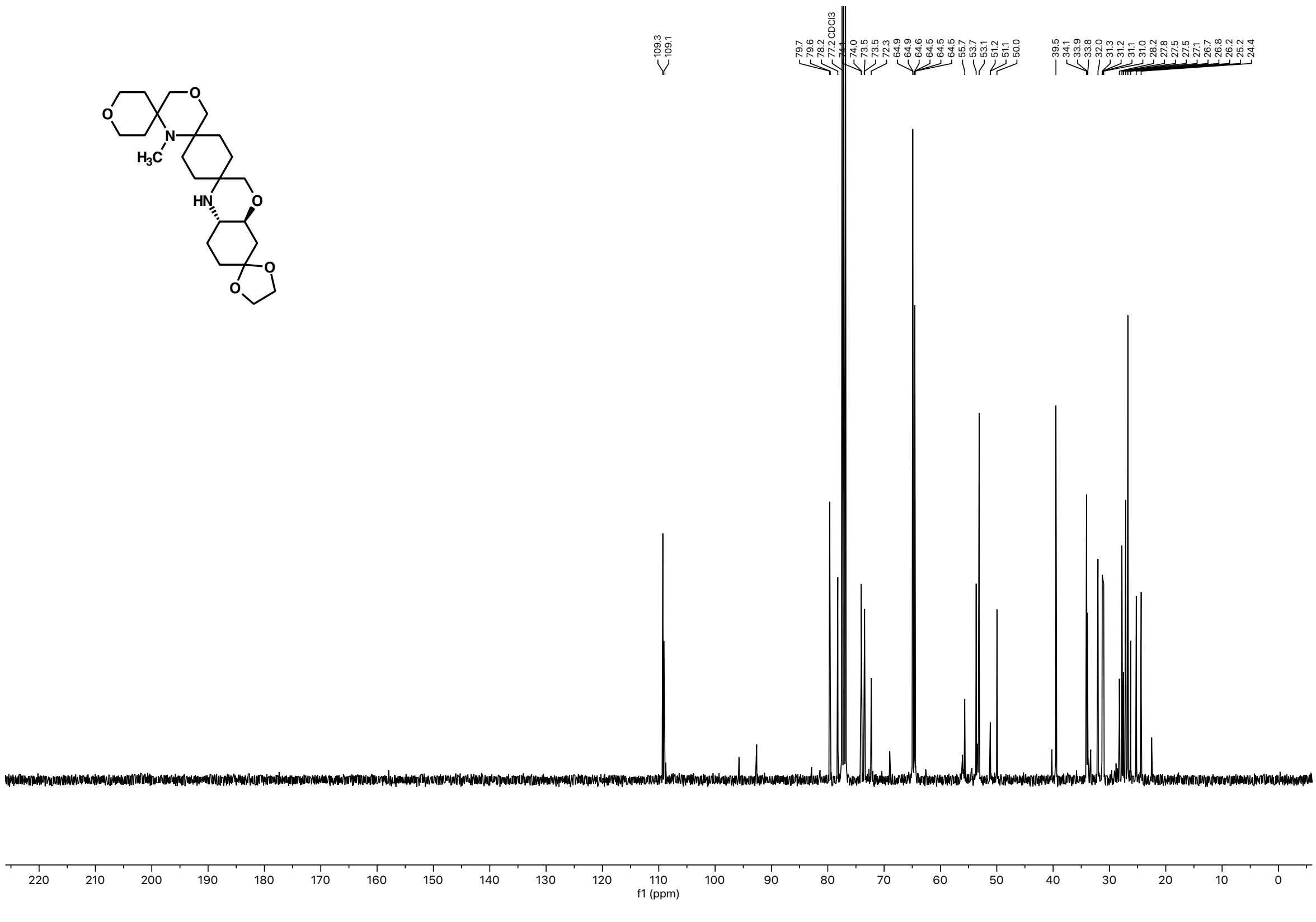

Compound 34: ^{1}H NMR ($CDCl_3$) top, ^{13}C NMR ($CDCl_3$) bottom

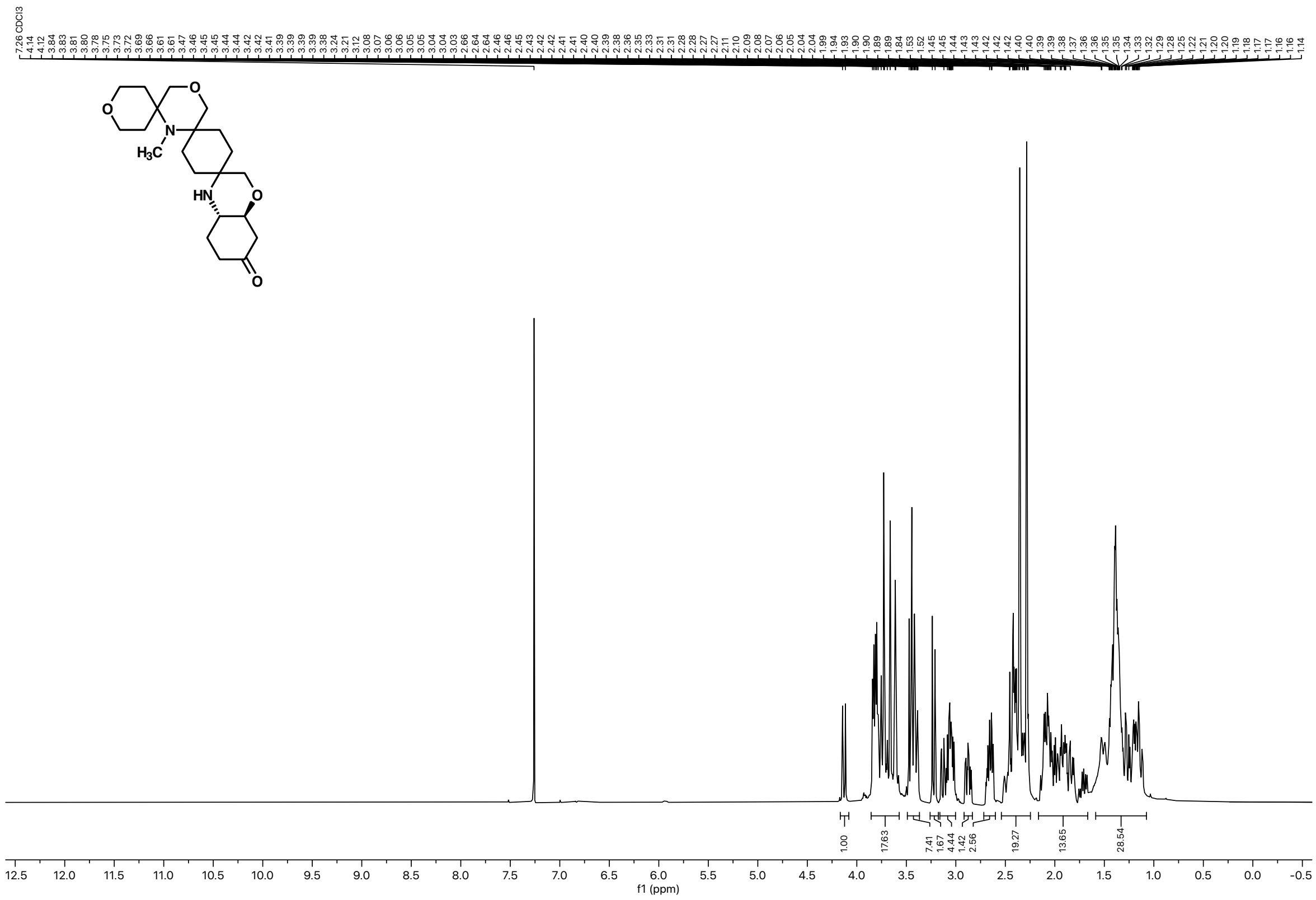

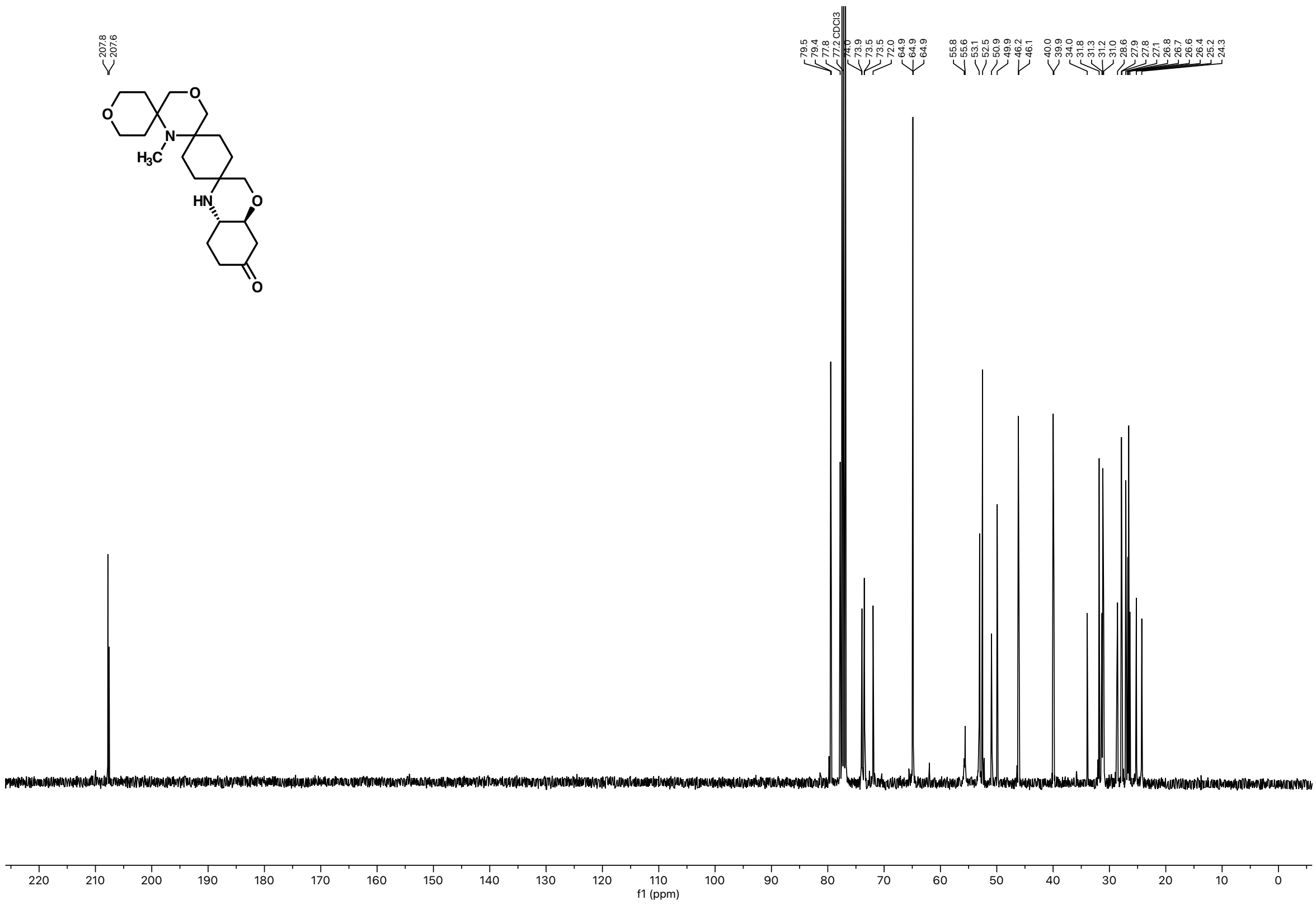

Compound 35: ^{1}H NMR ($CDCl_3$) top, ^{13}C NMR ($CDCl_3$) bottom

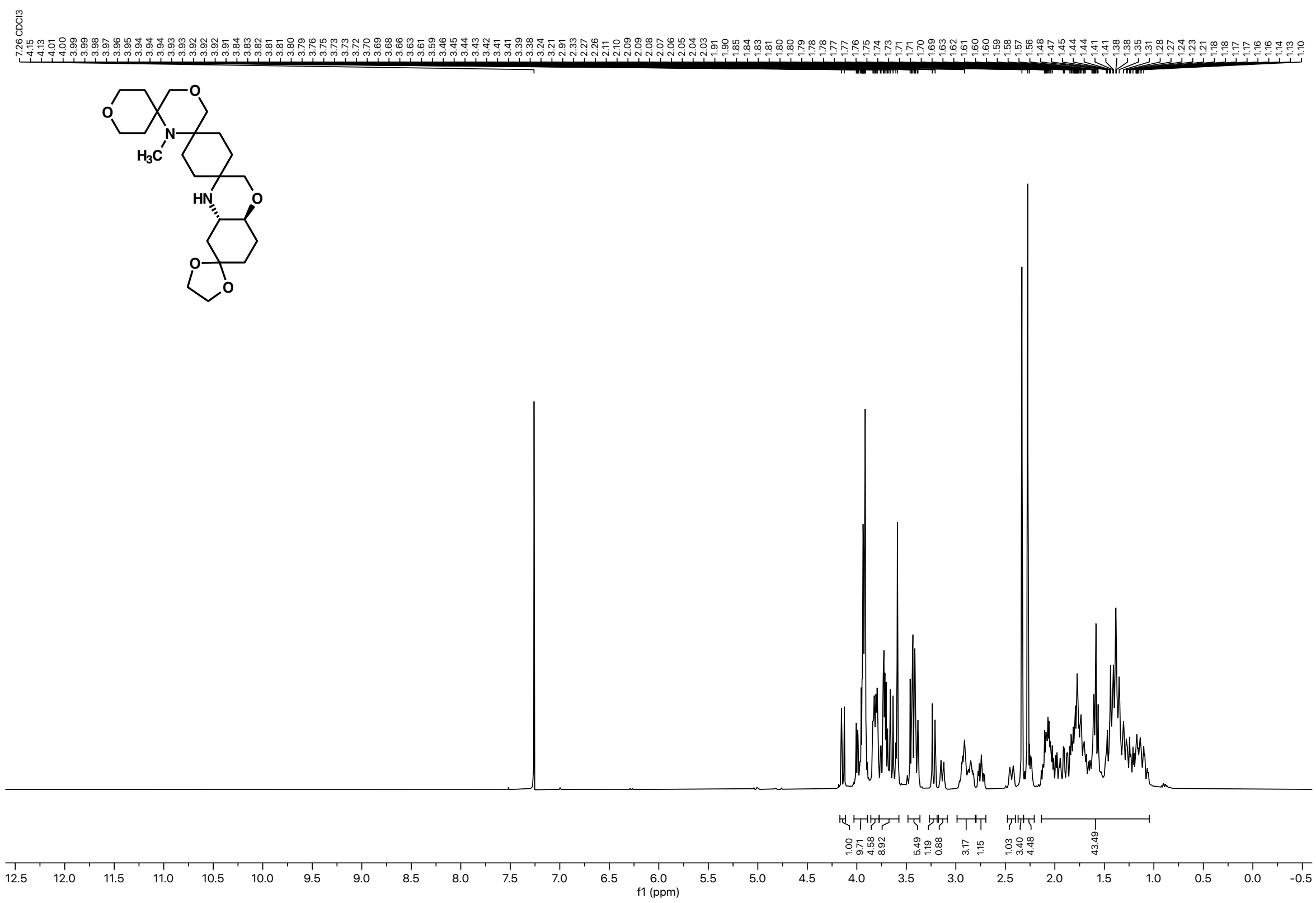

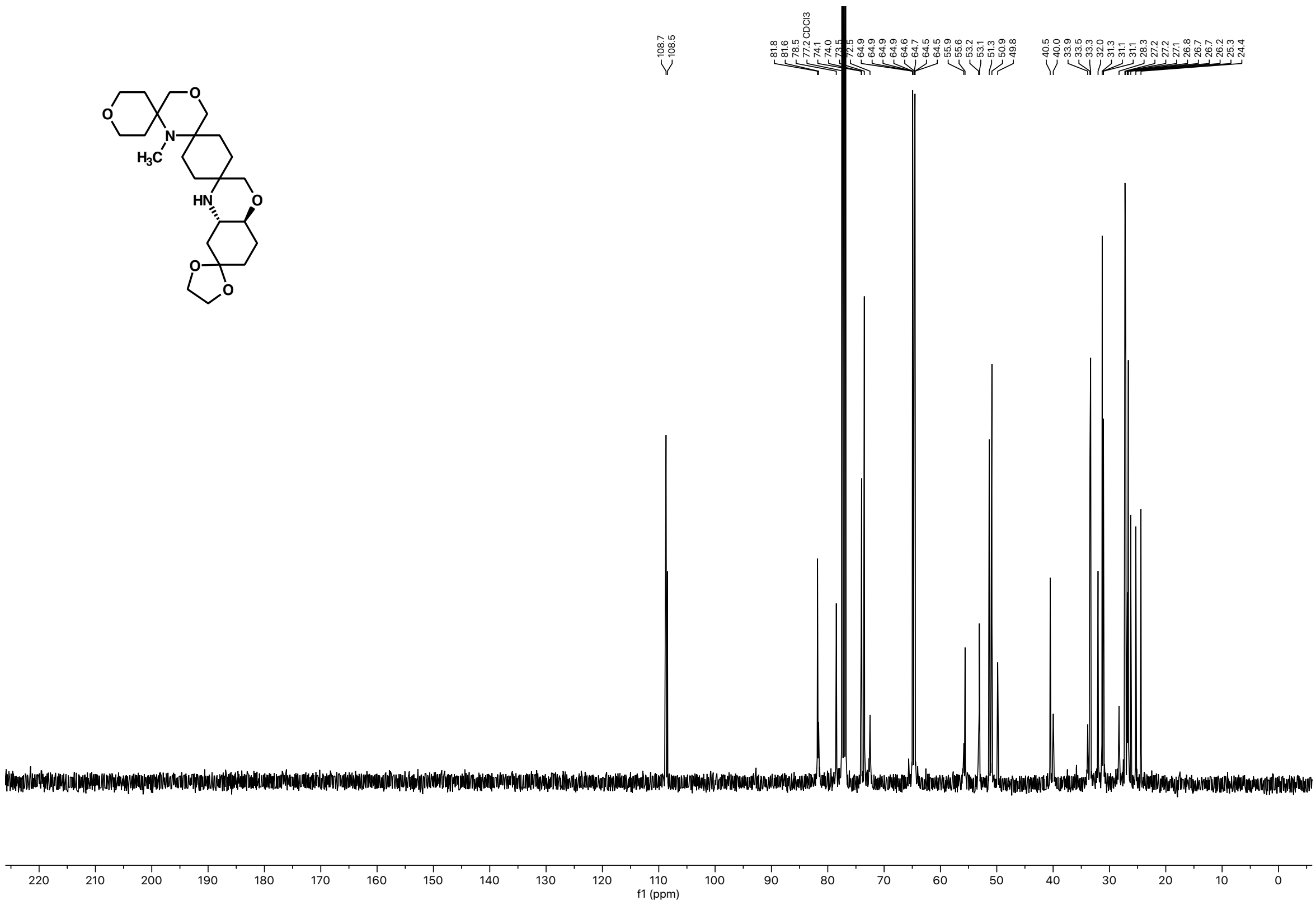

Compound 36: ^{1}H NMR ($CDCl_3$) top, ^{13}C NMR ($CDCl_3$) bottom

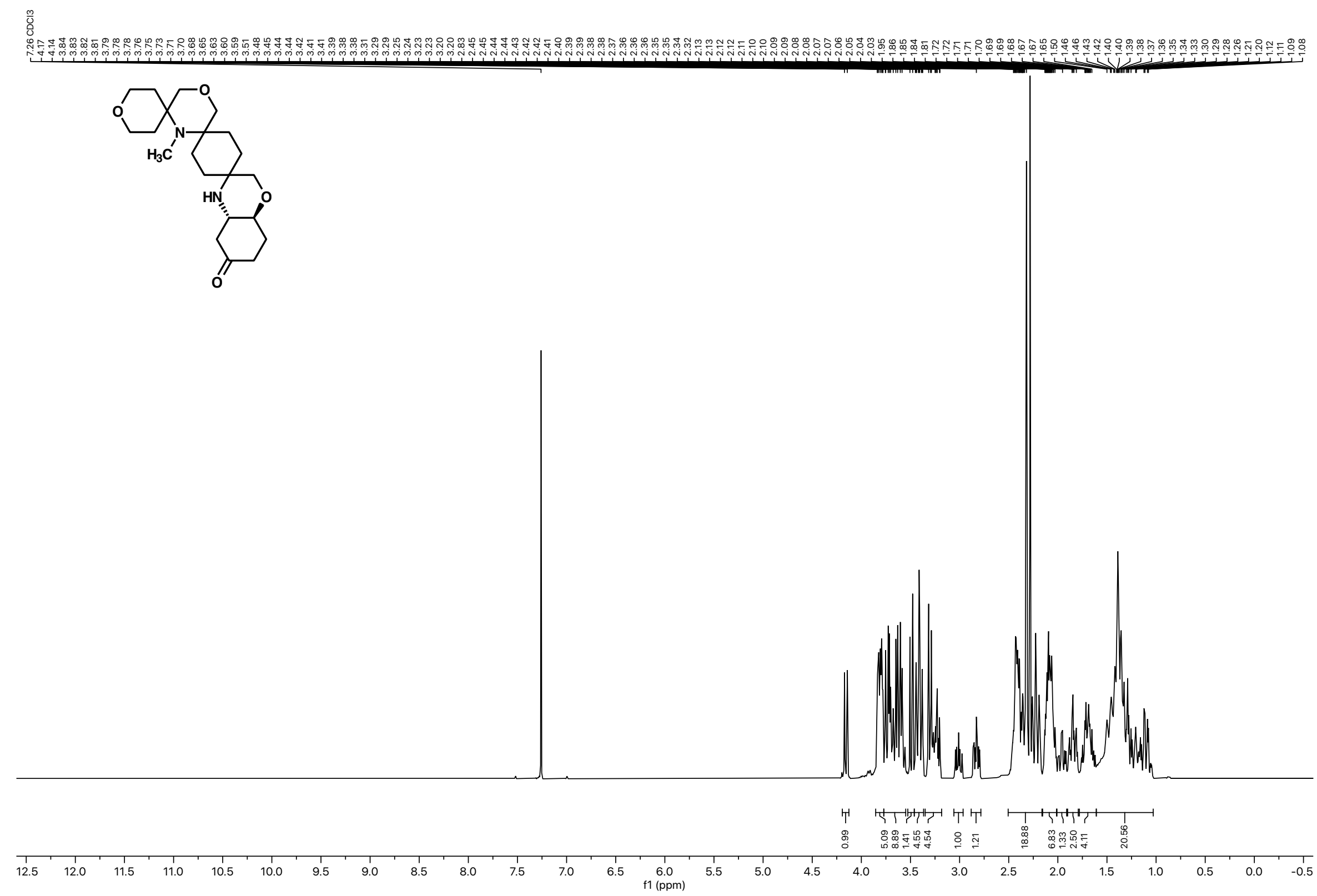

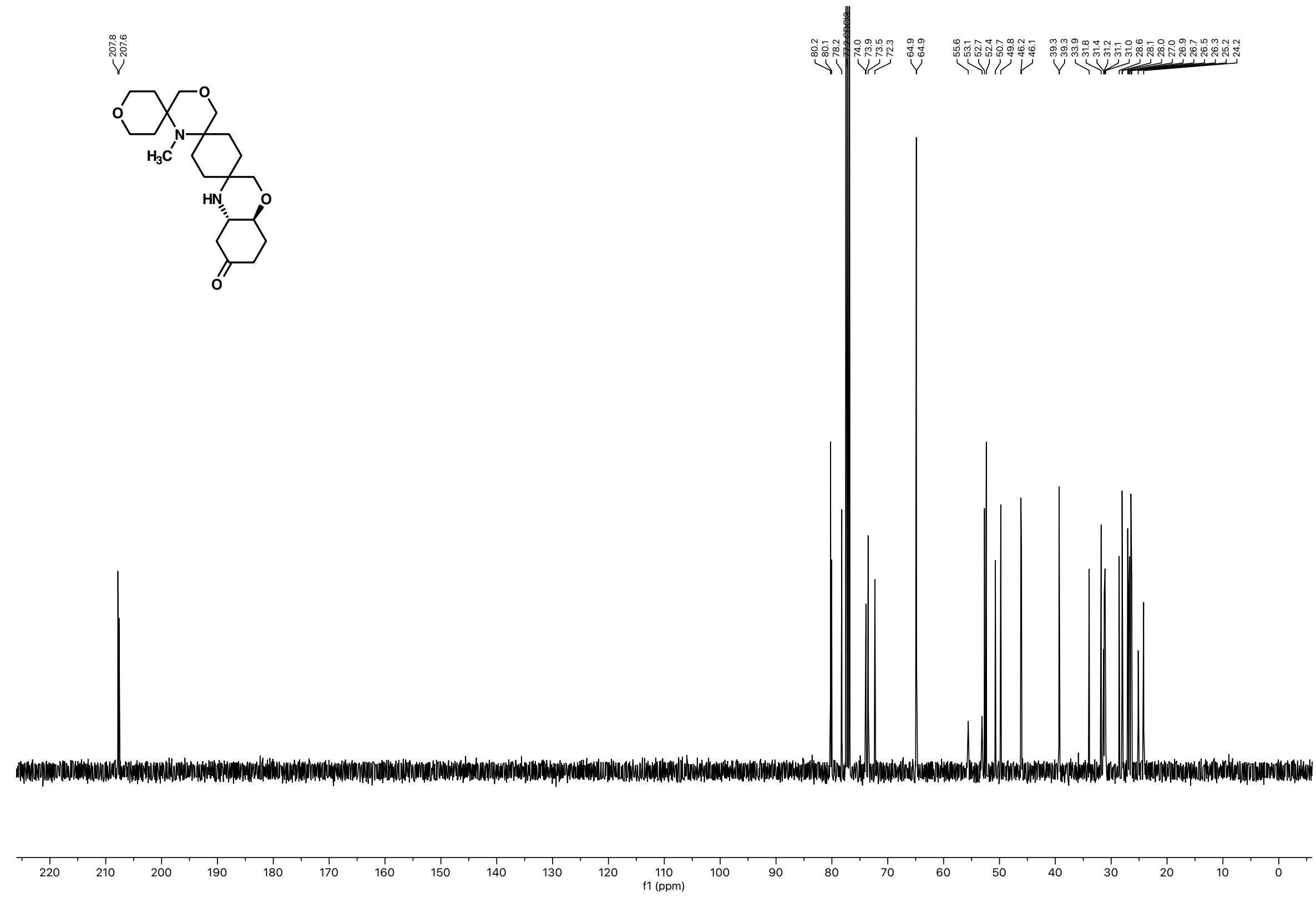

Compound 37: ^{1}H NMR ($CDCl_3$) top, ^{13}C NMR ($CDCl_3$) bottom

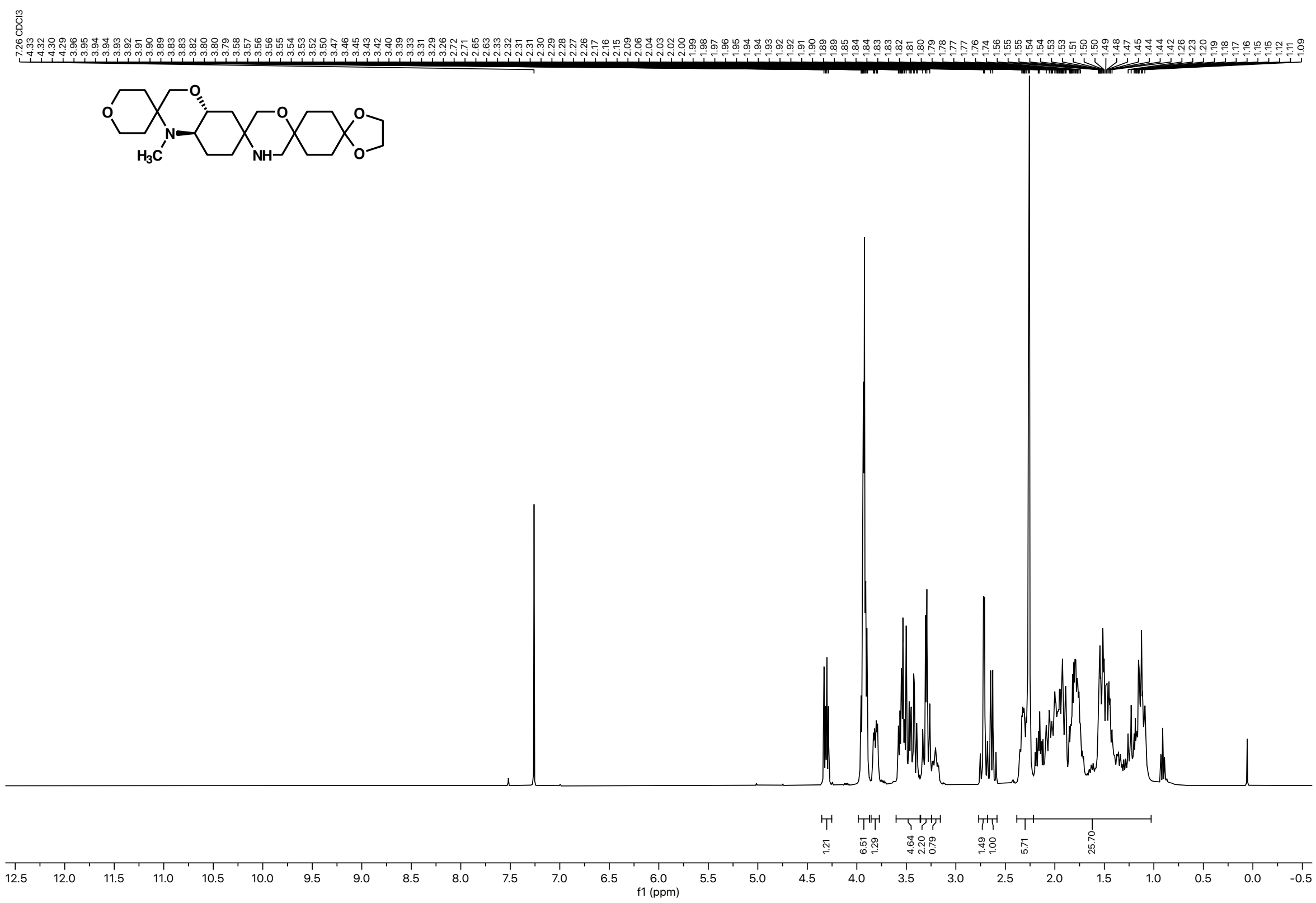

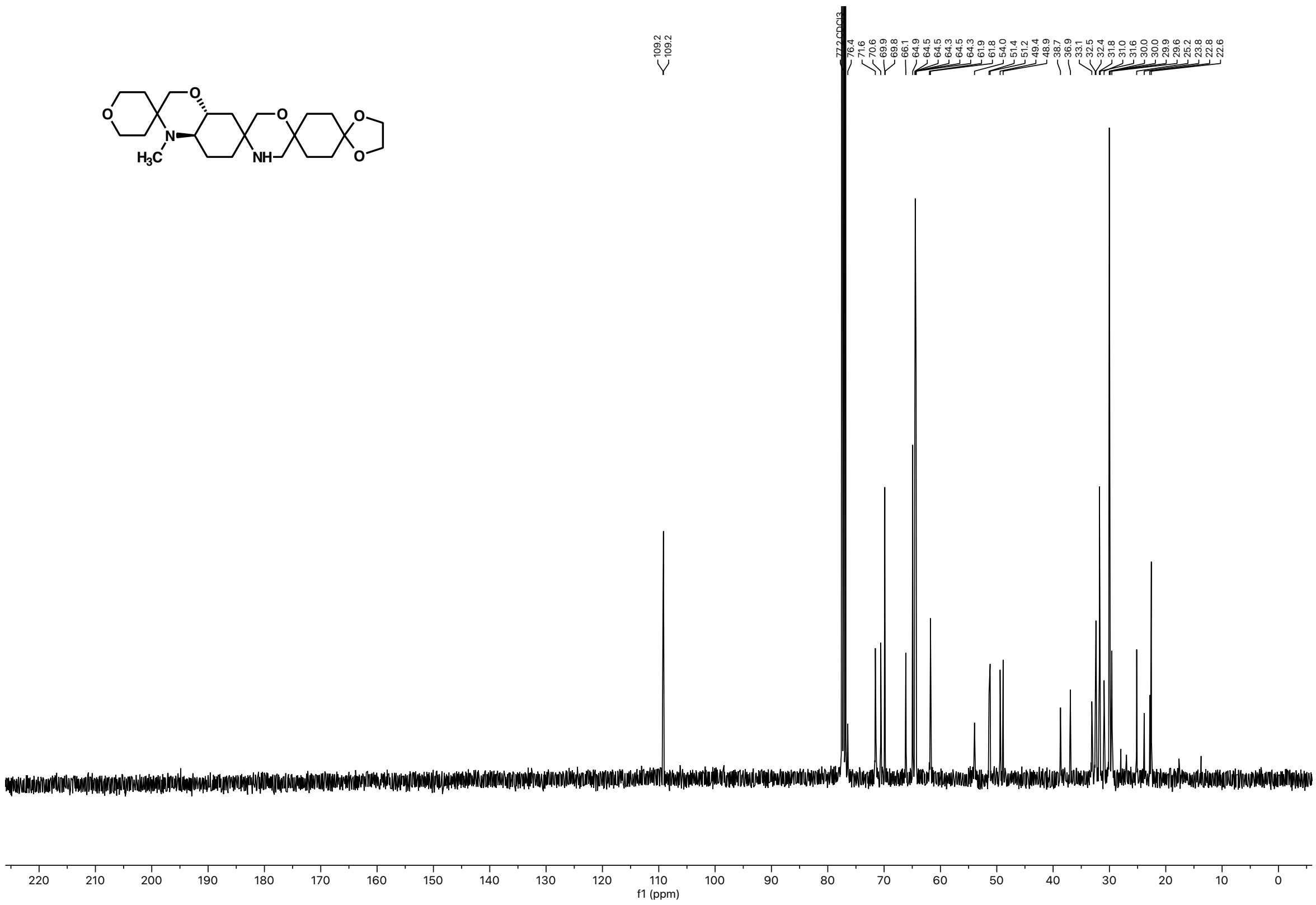

Compound 38: ^{1}H NMR ($CDCl_3$) top, ^{13}C NMR ($CDCl_3$) bottom

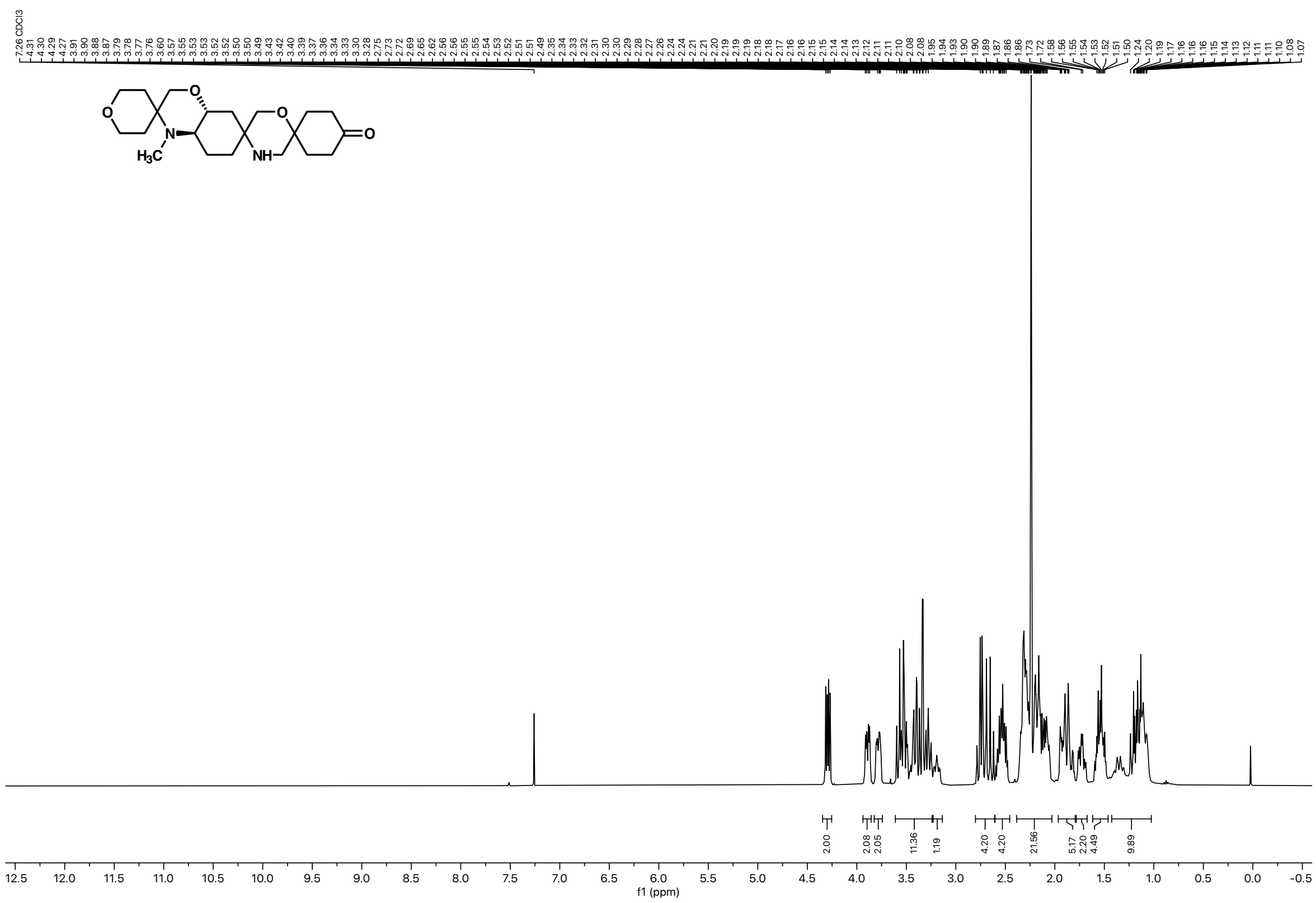

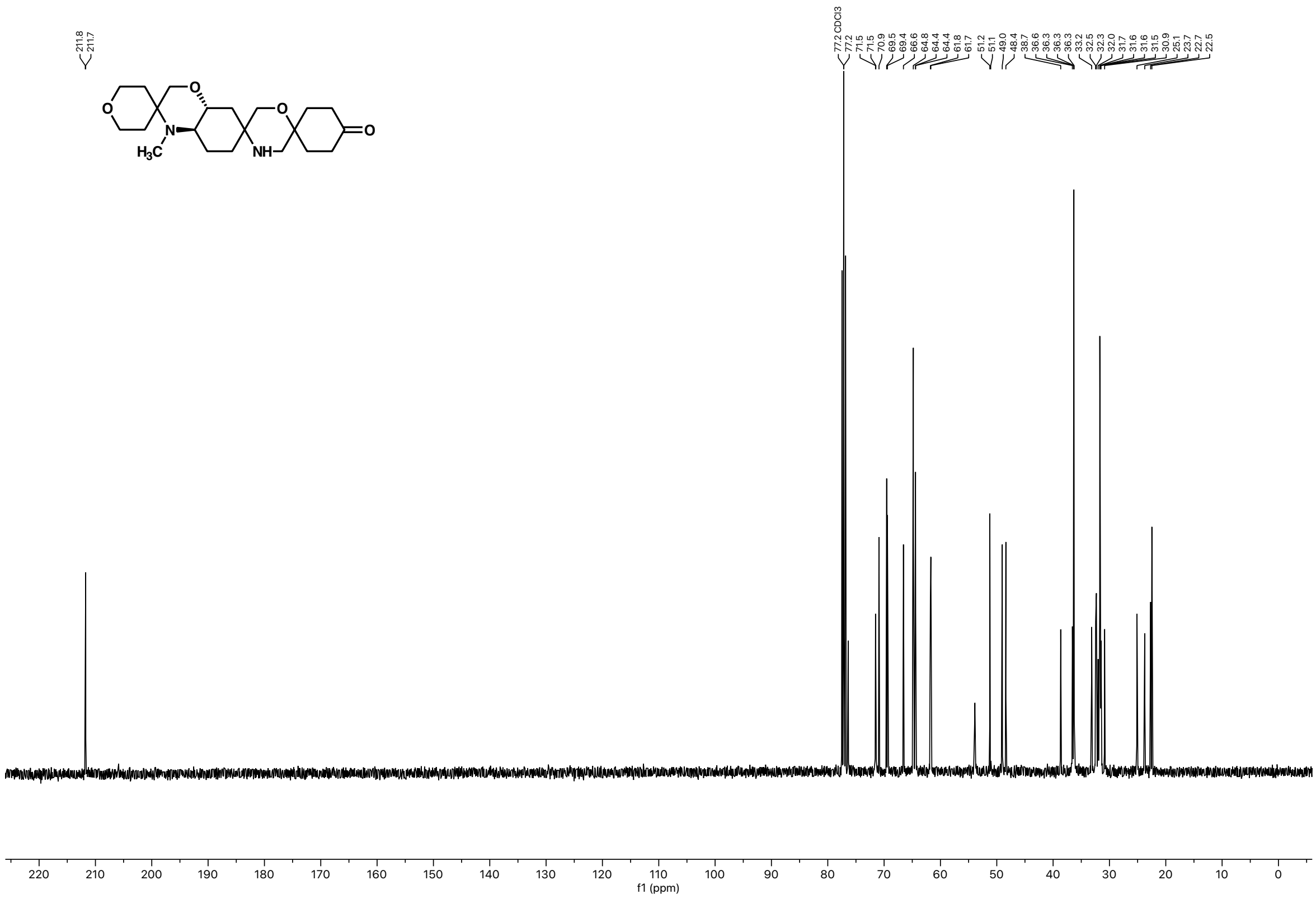

Compound 39: ^{1}H NMR ($CDCl_3$) top, ^{13}C NMR ($CDCl_3$) bottom

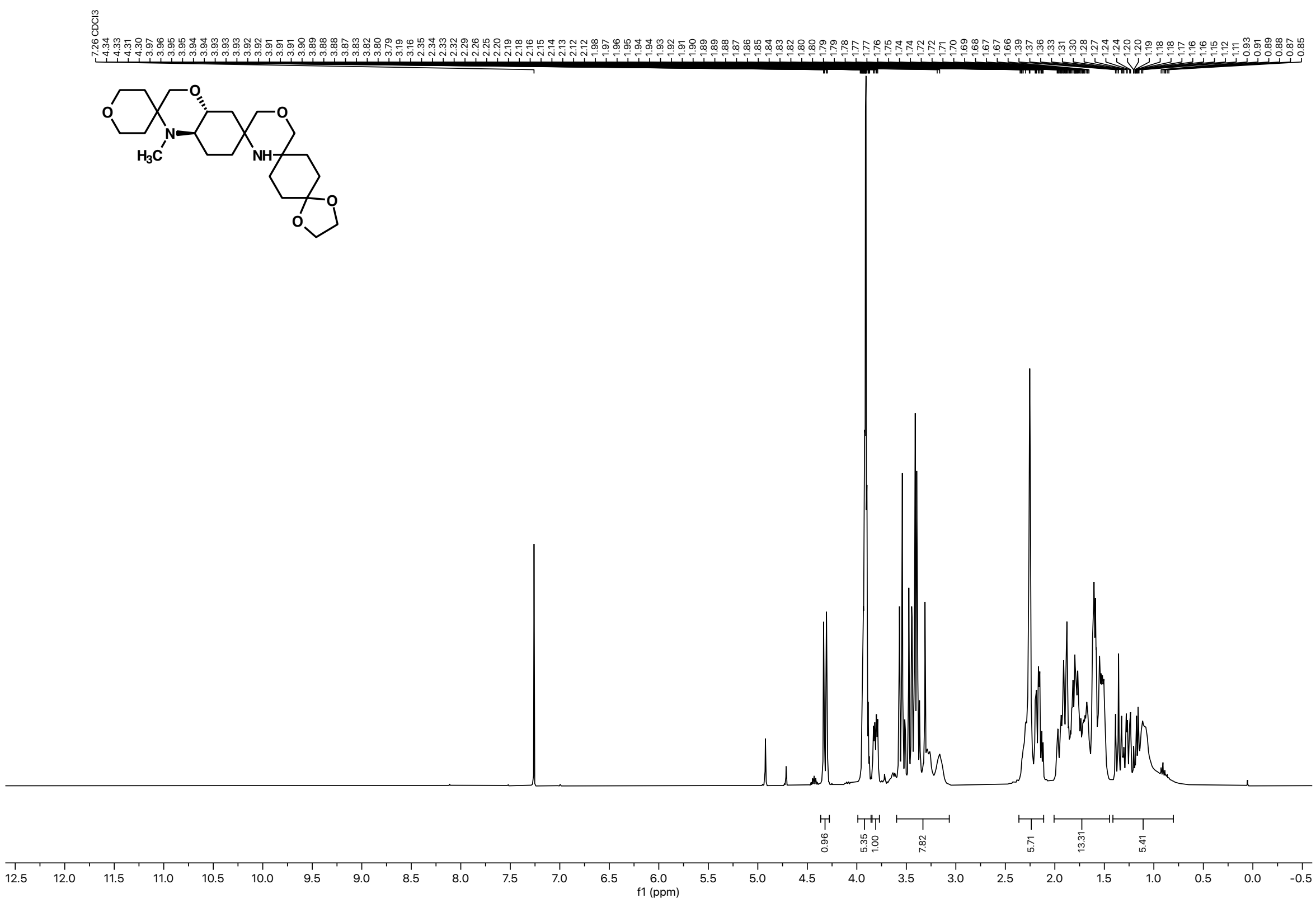

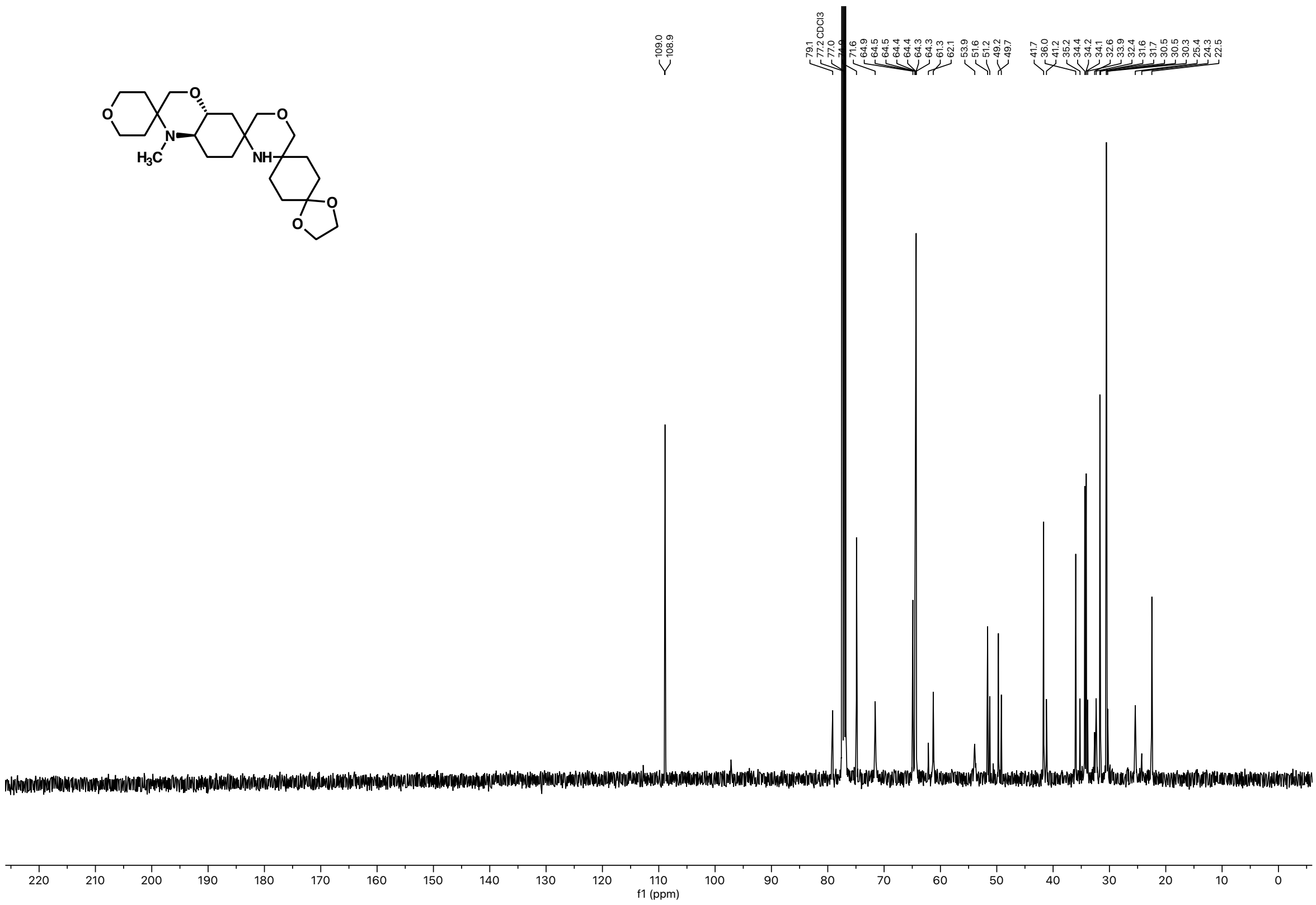

Compound 40: ^{1}H NMR ($CDCl_3$) top, ^{13}C NMR ($CDCl_3$) bottom

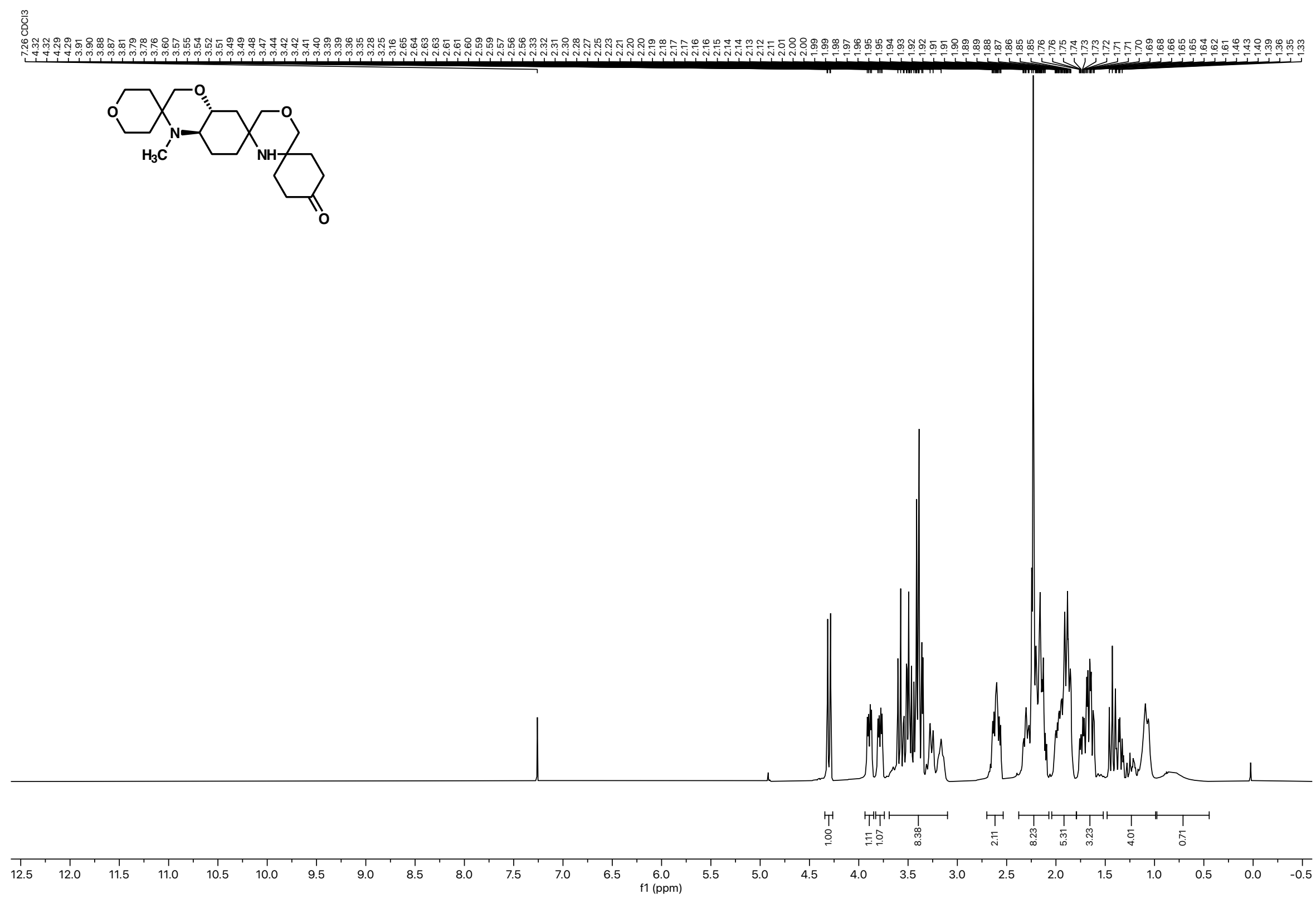

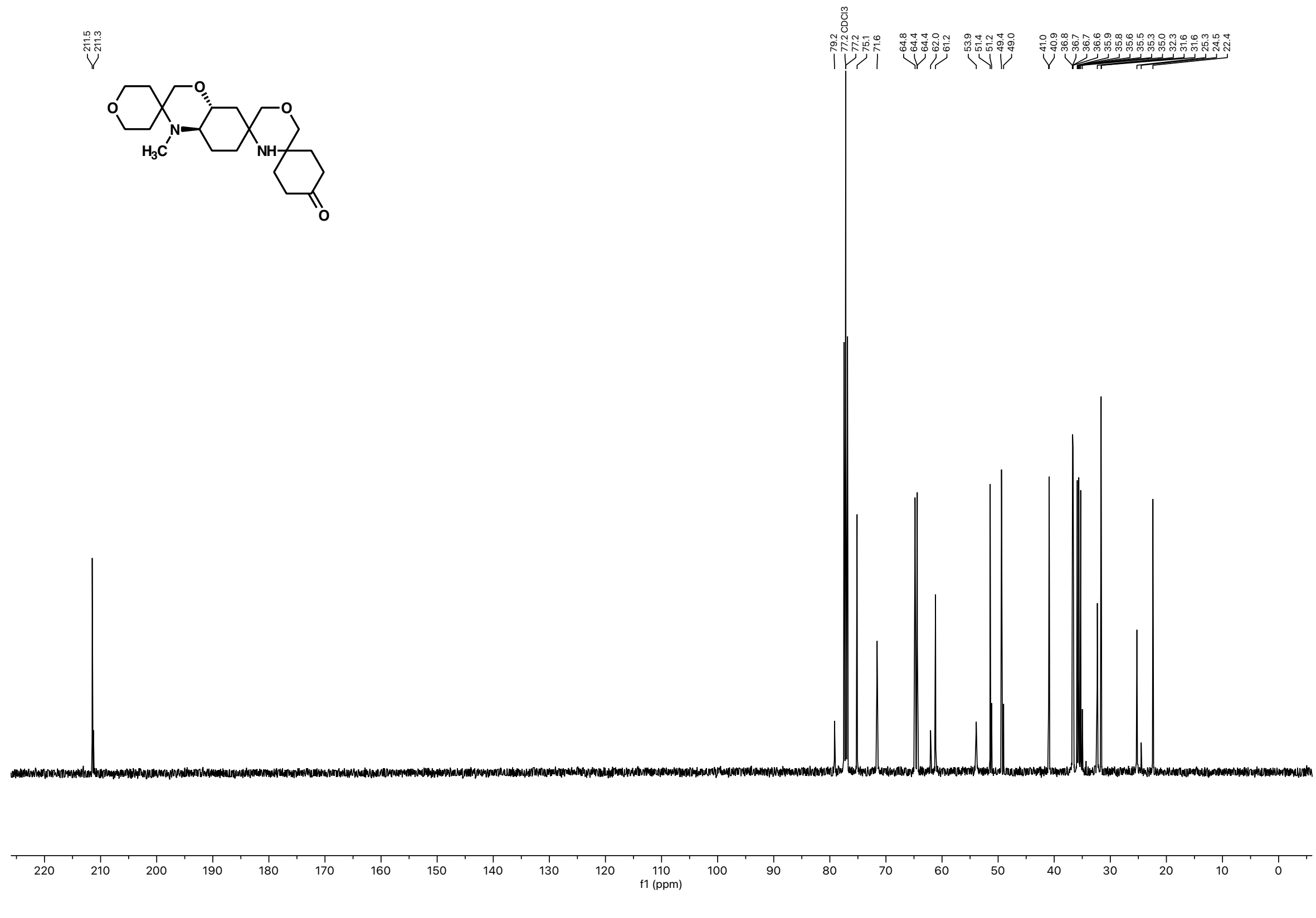

Compound 41: ^{1}H NMR ($CDCl_3$) top, ^{13}C NMR ($CDCl_3$) bottom

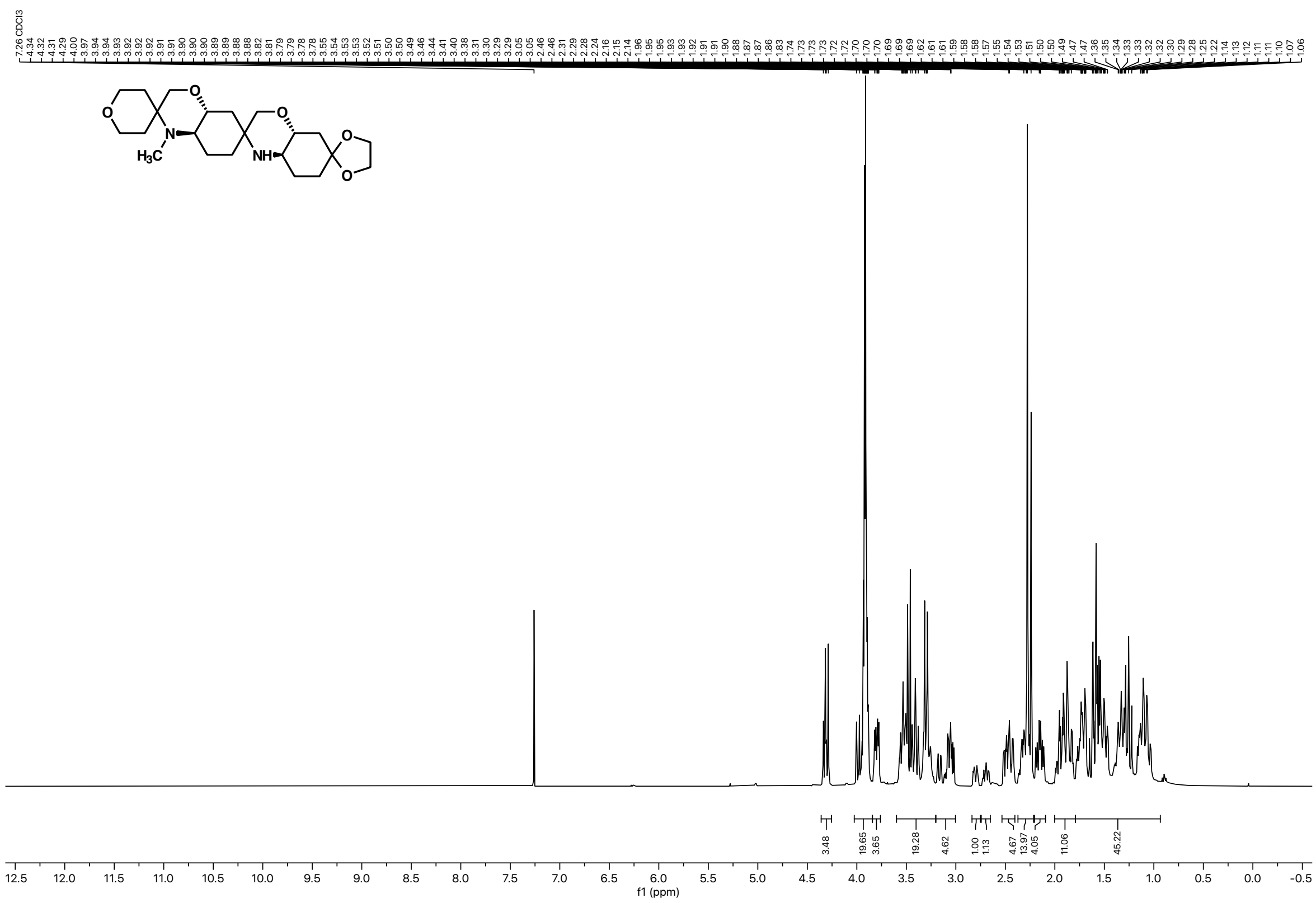

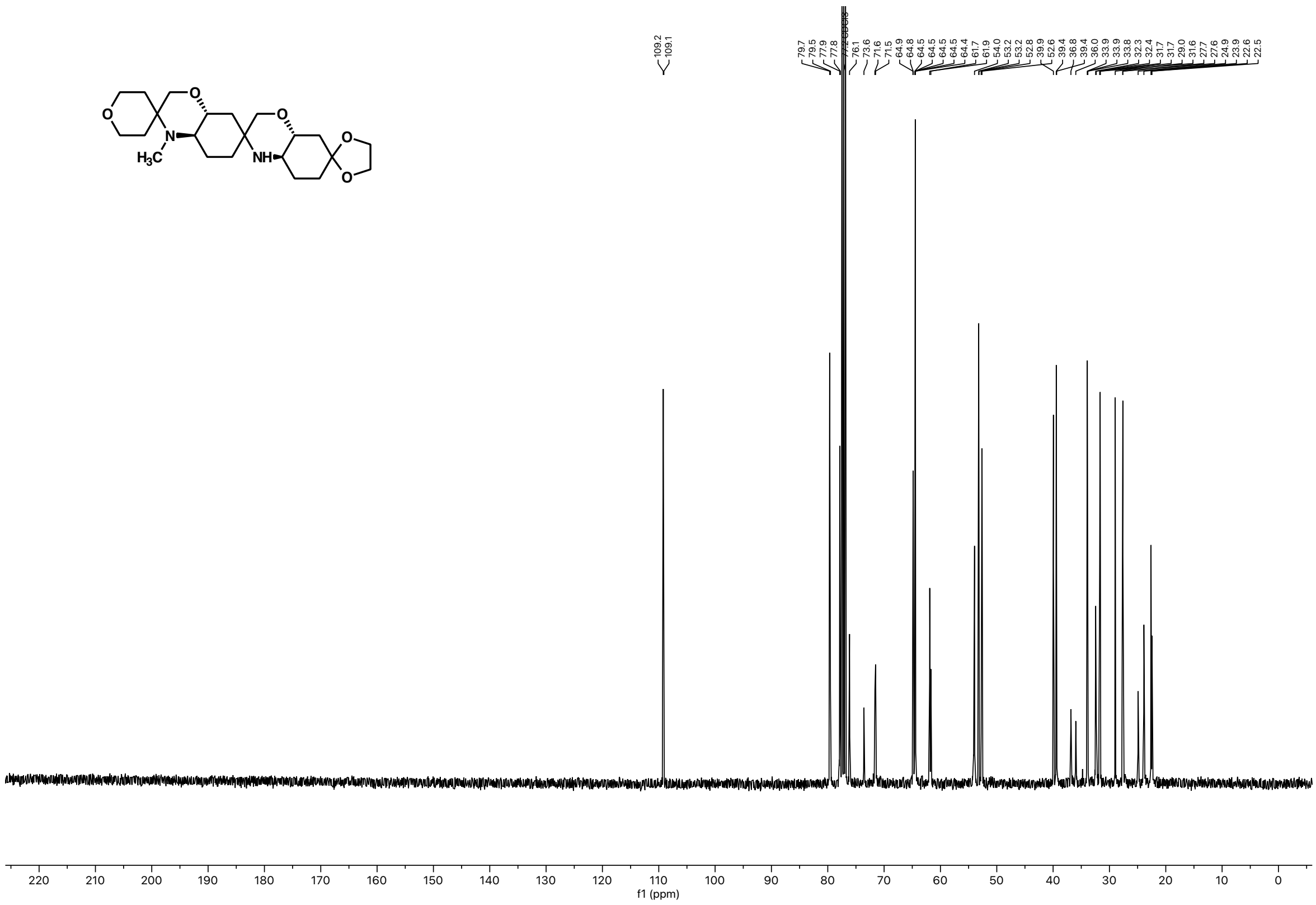

Compound 42: ^{1}H NMR ($CDCl_3$) top, ^{13}C NMR ($CDCl_3$) bottom

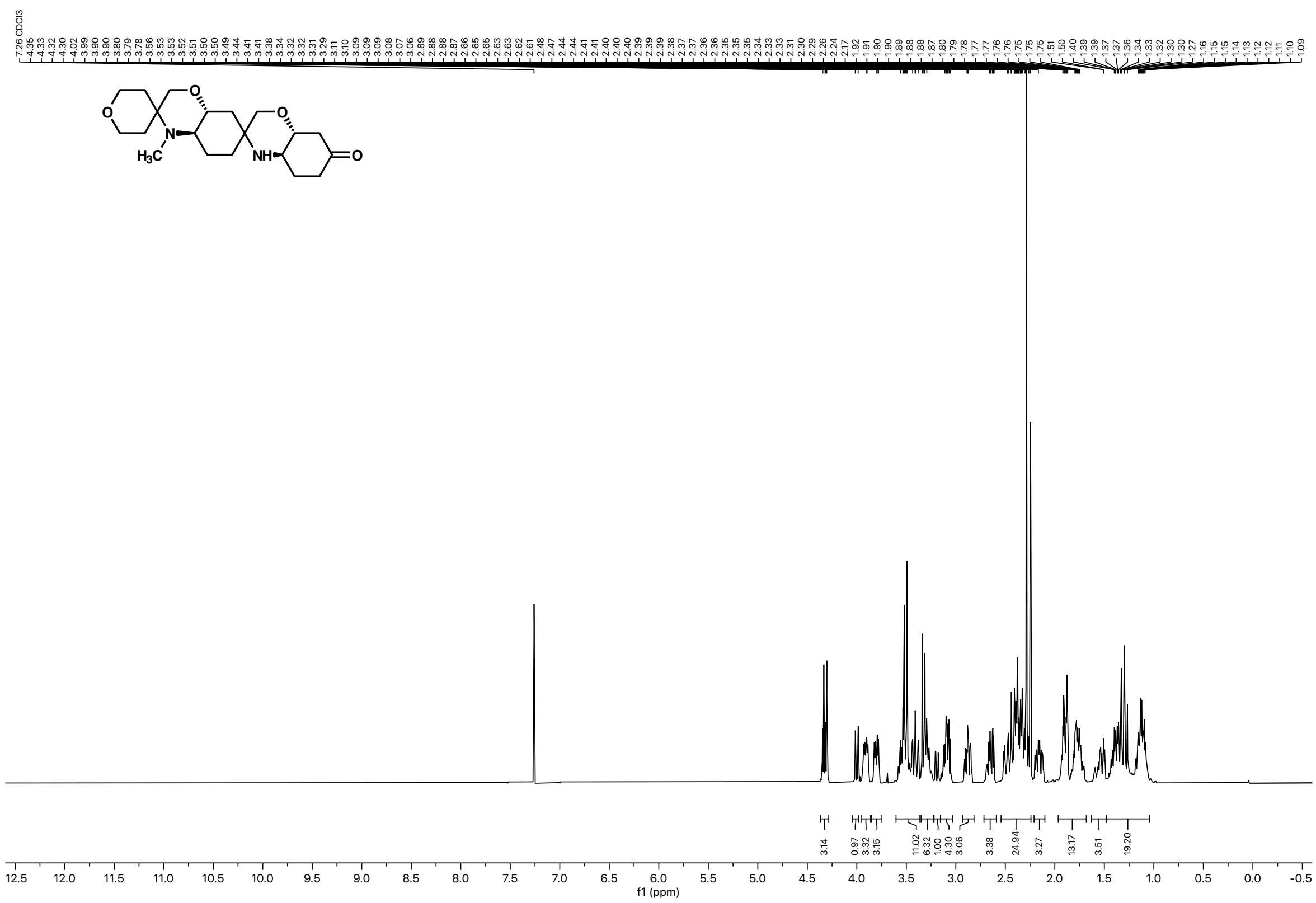

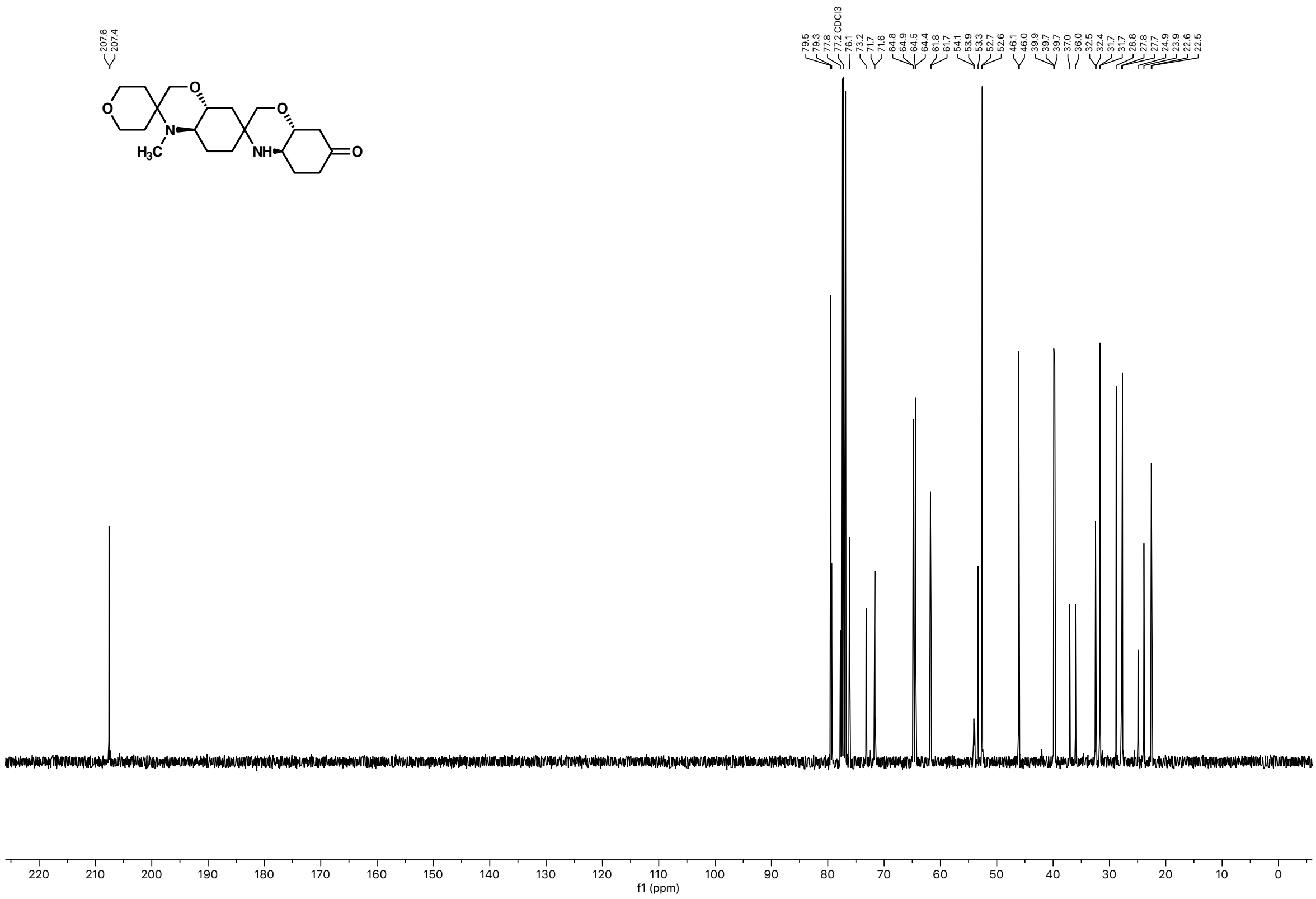

Compound 43: ^{1}H NMR ($CDCl_3$) top, ^{13}C NMR ($CDCl_3$) bottom

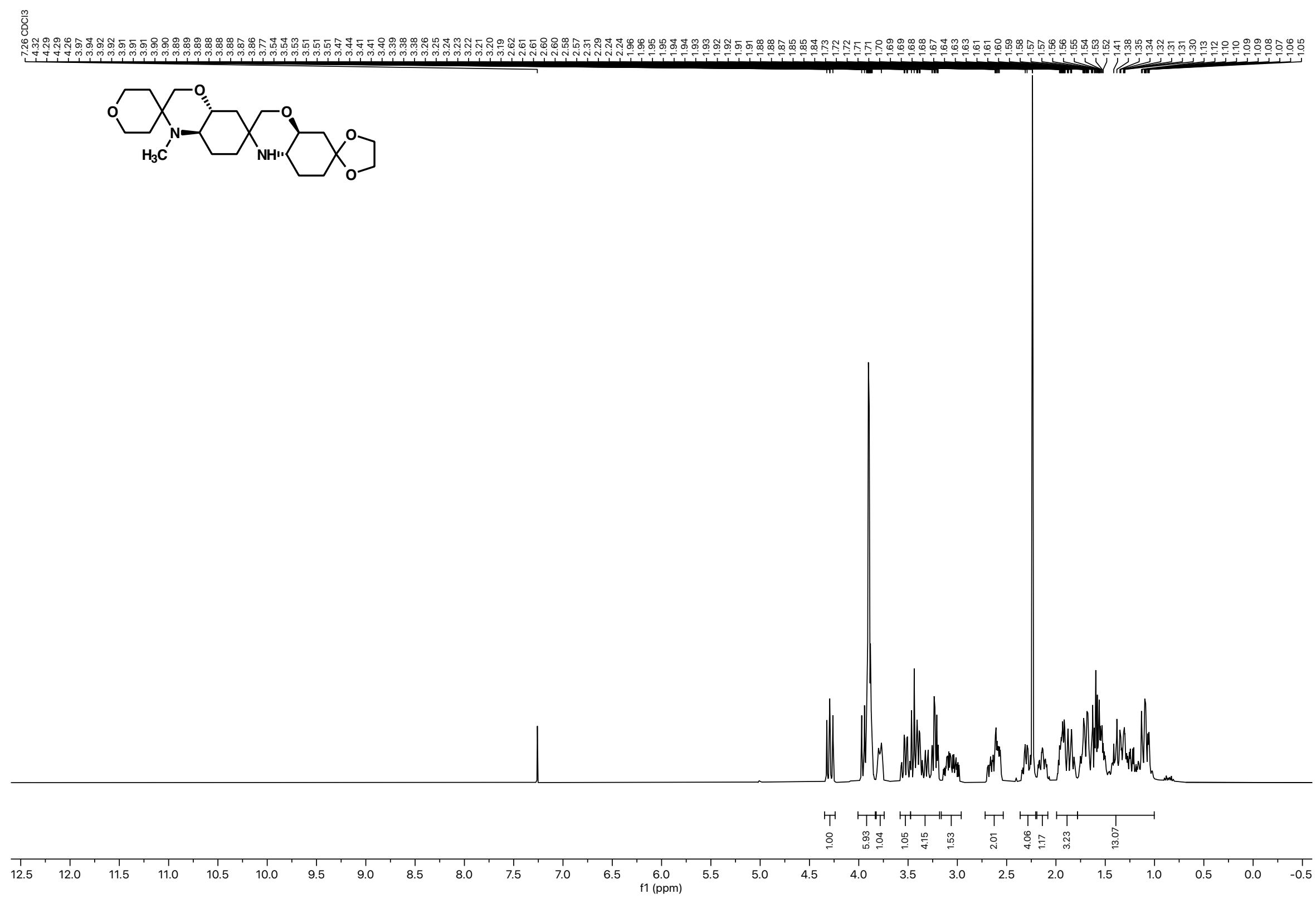

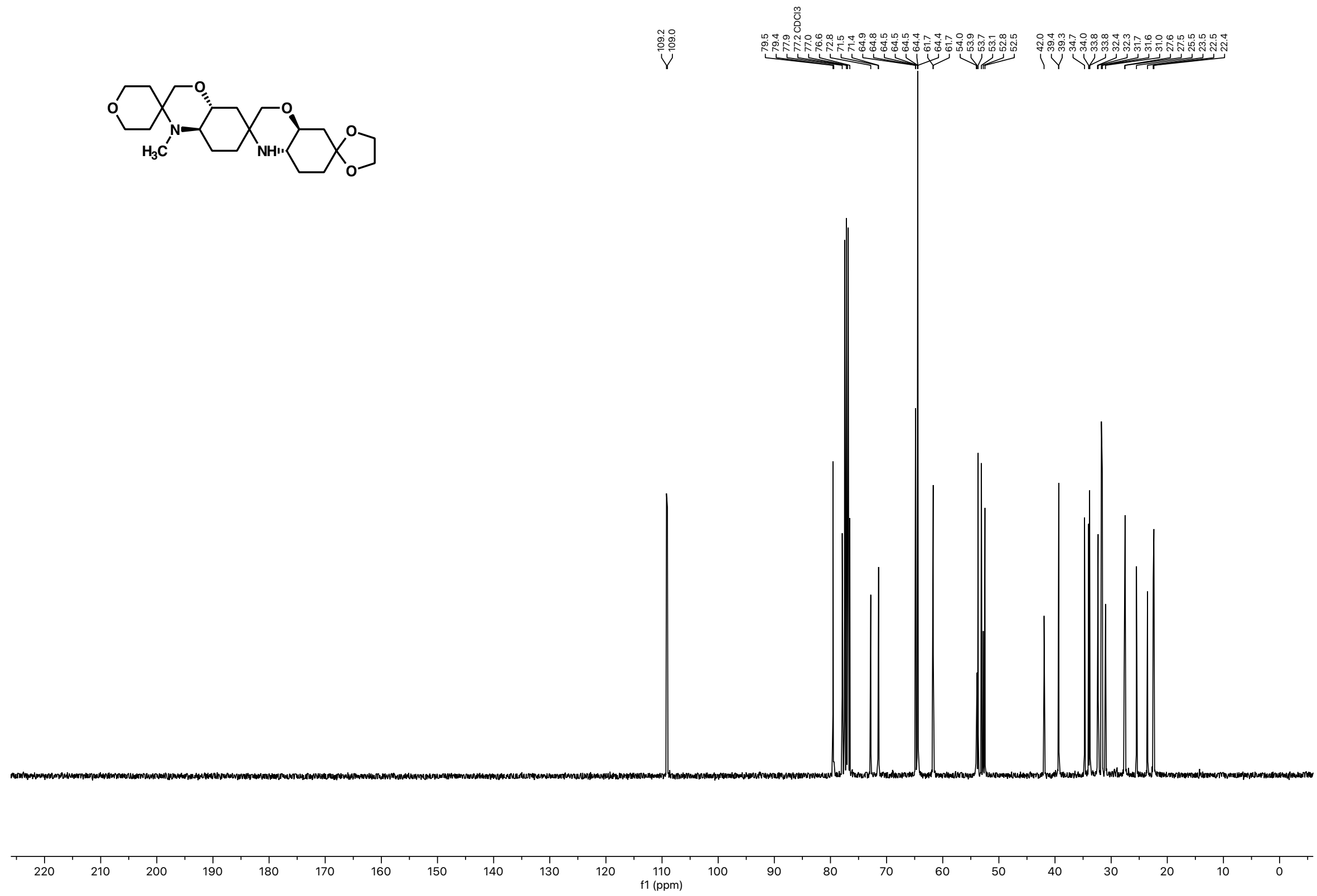

Compound 44: ^{1}H NMR ($CDCl_3$) top, ^{13}C NMR ($CDCl_3$) bottom

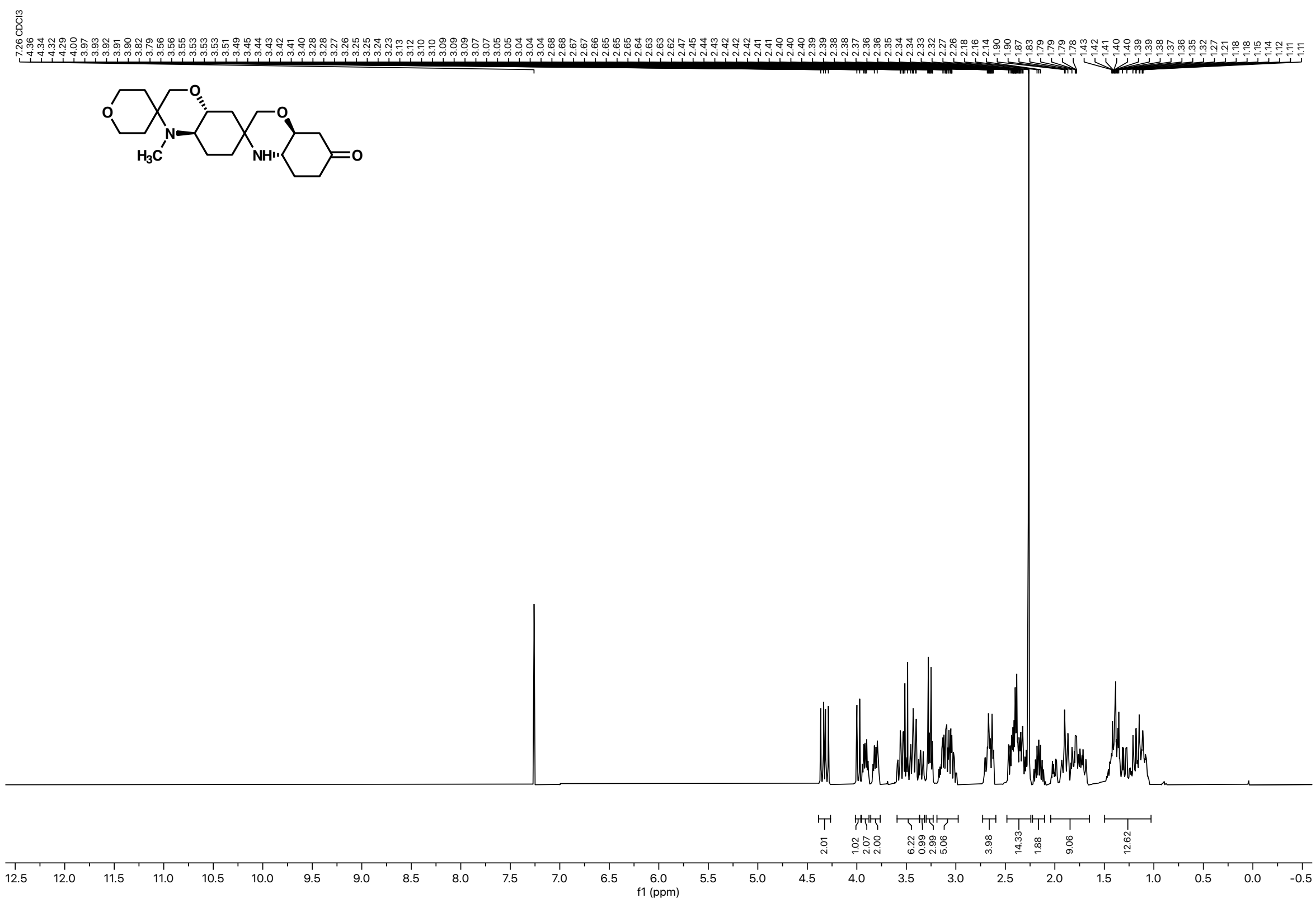

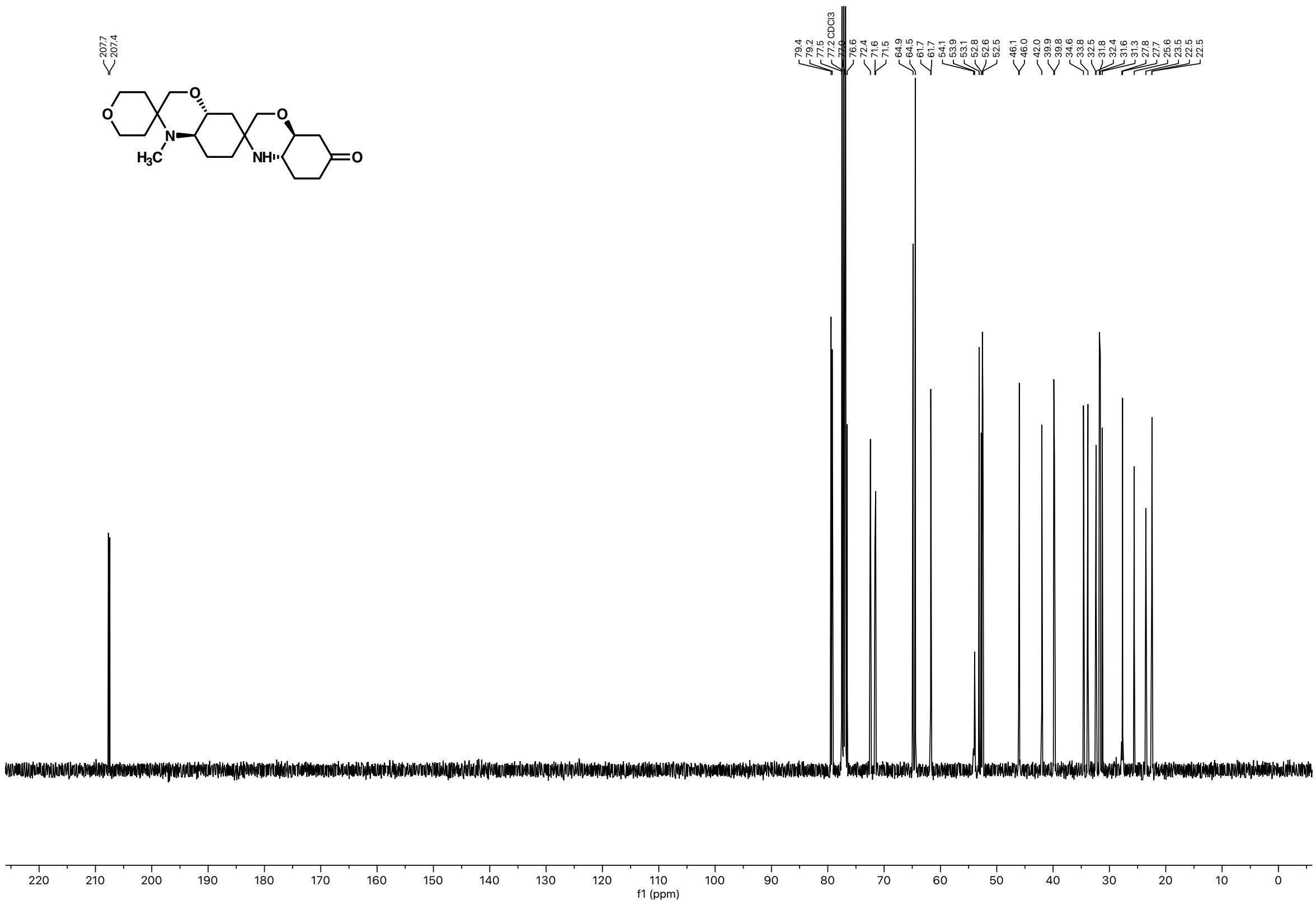

Compound 45: ^{1}H NMR ($CDCl_3$) top, ^{13}C NMR ($CDCl_3$) bottom

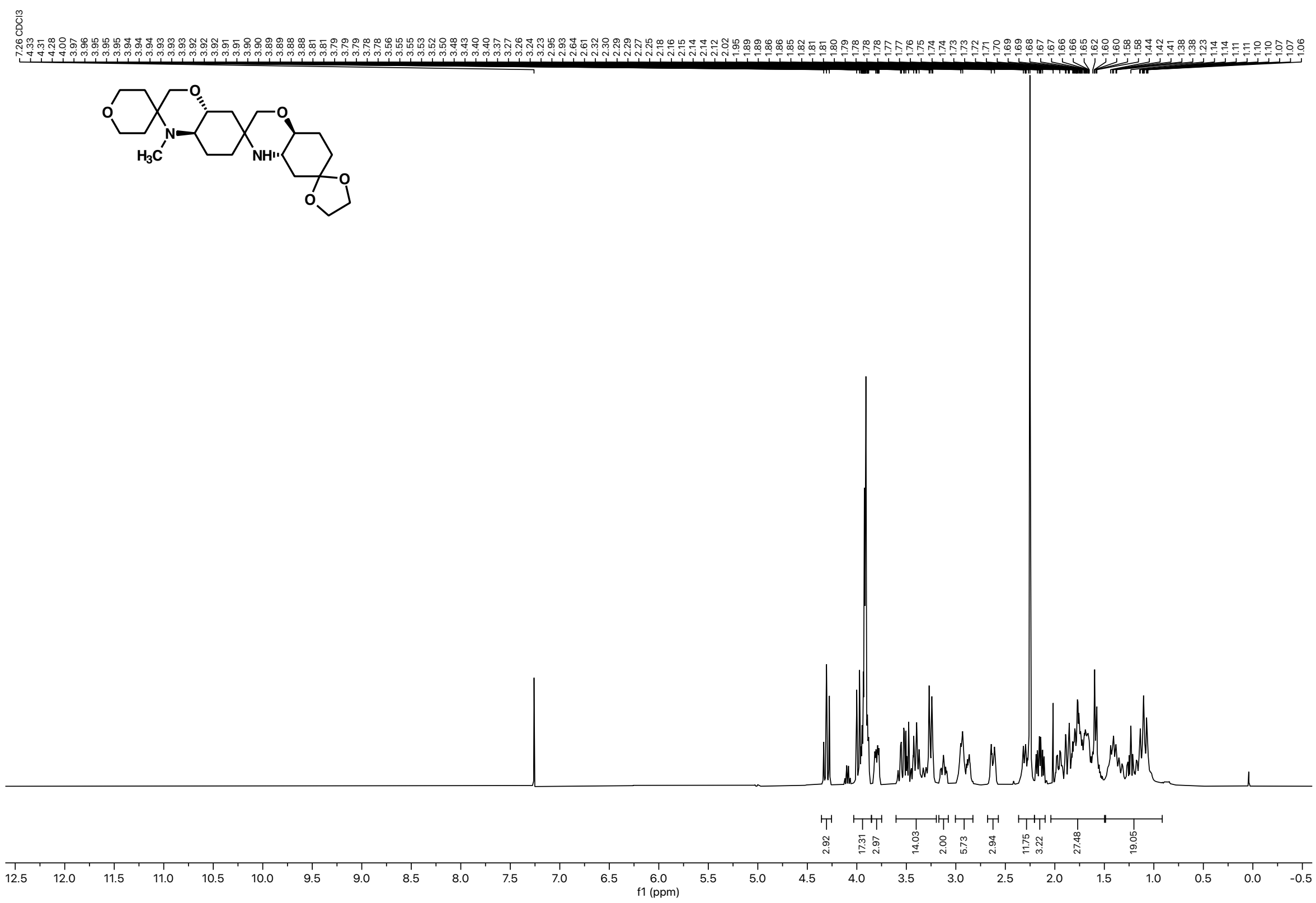

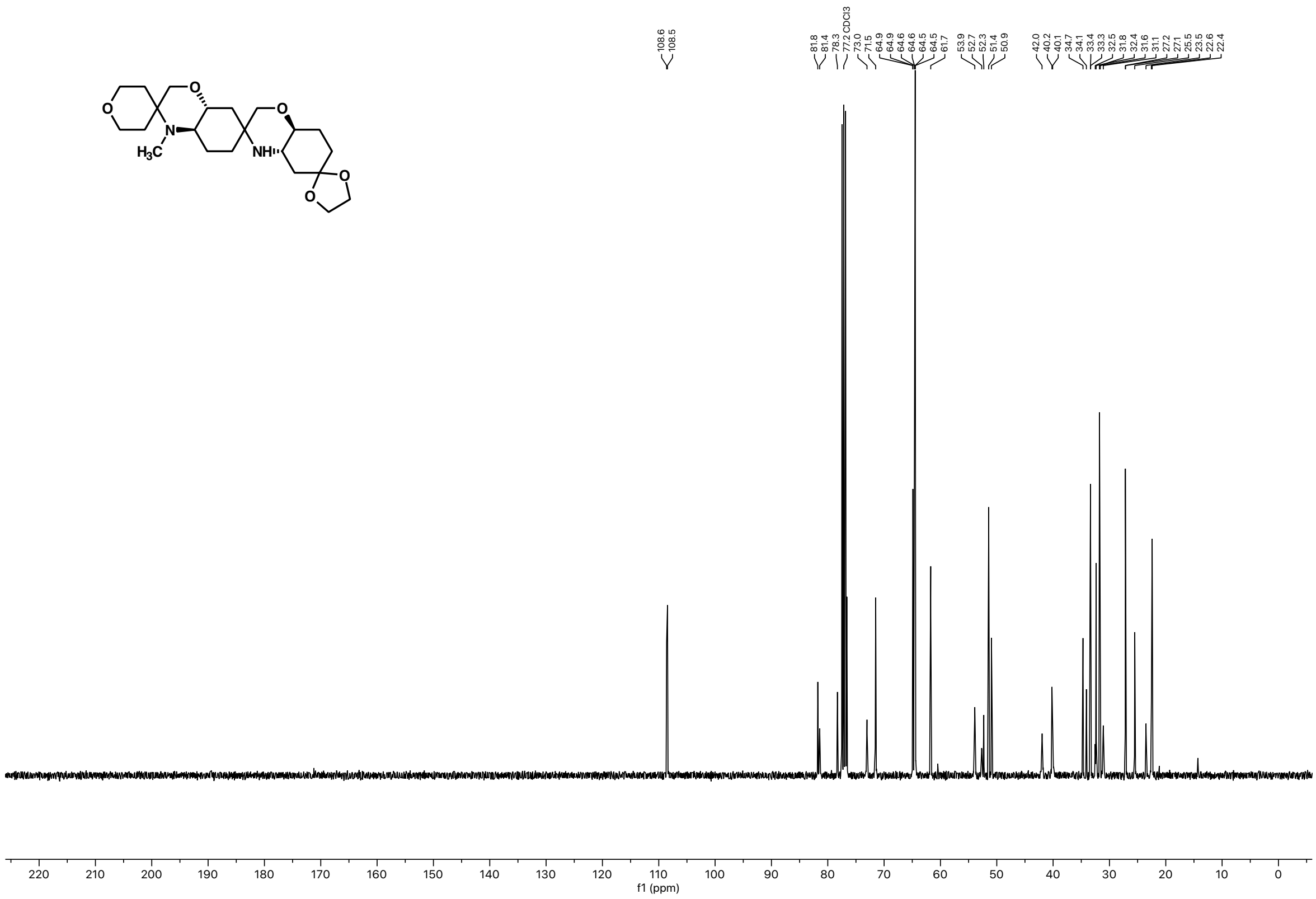

Compound 46: ^{1}H NMR ($CDCl_3$) top, ^{13}C NMR ($CDCl_3$) bottom

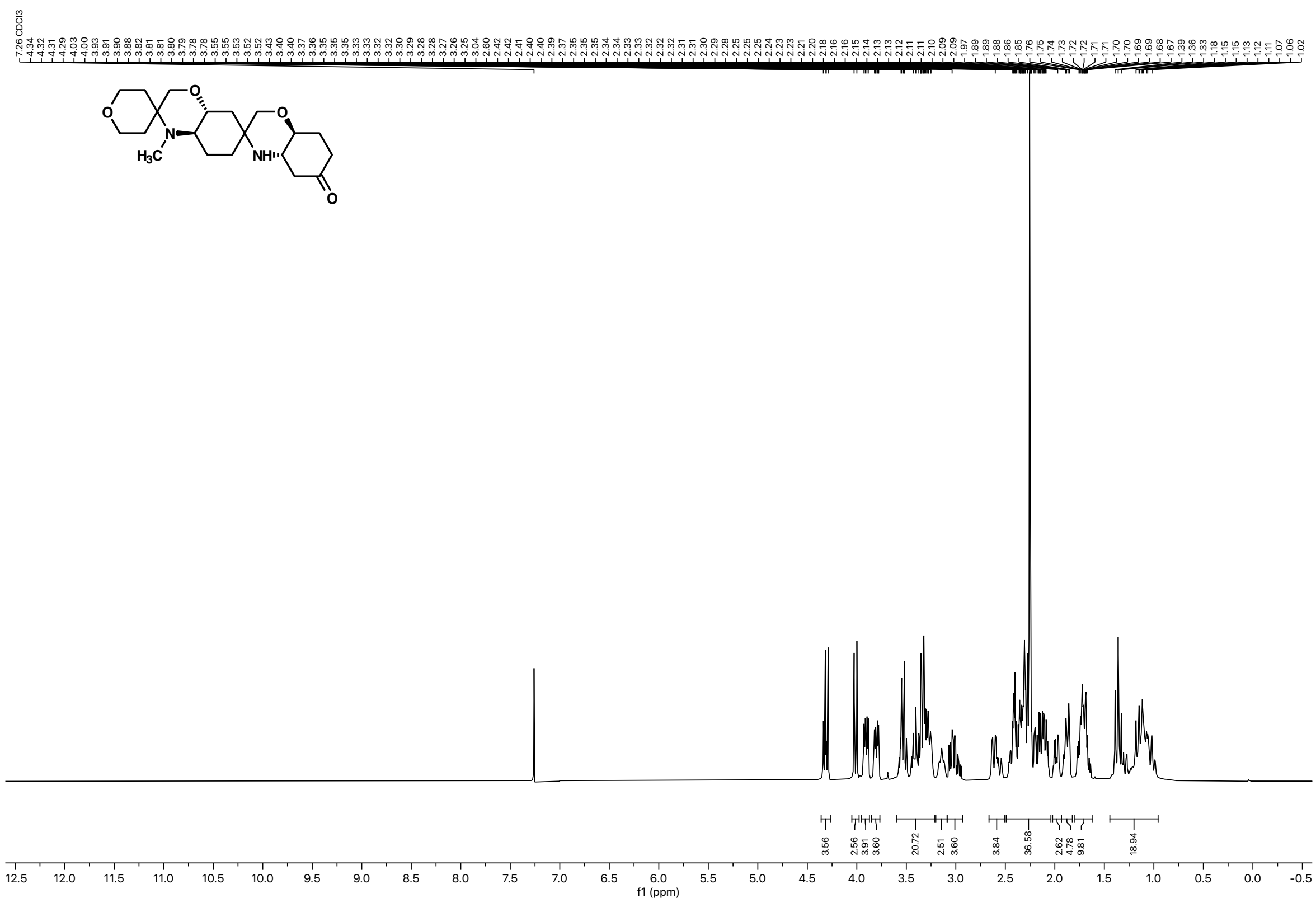

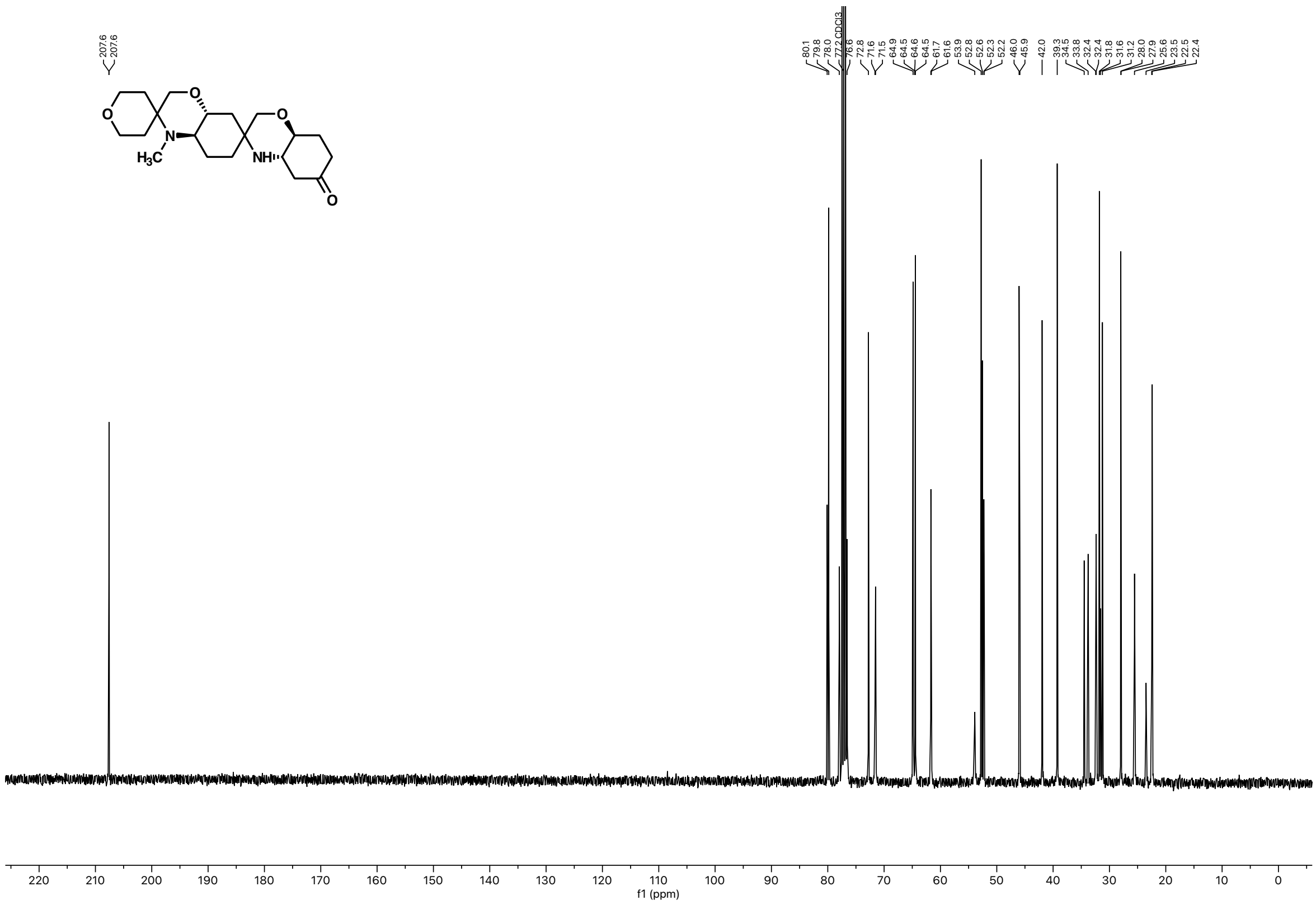

Compound 47: ^{1}H NMR ($CDCl_3$) top, ^{13}C NMR ($CDCl_3$) bottom

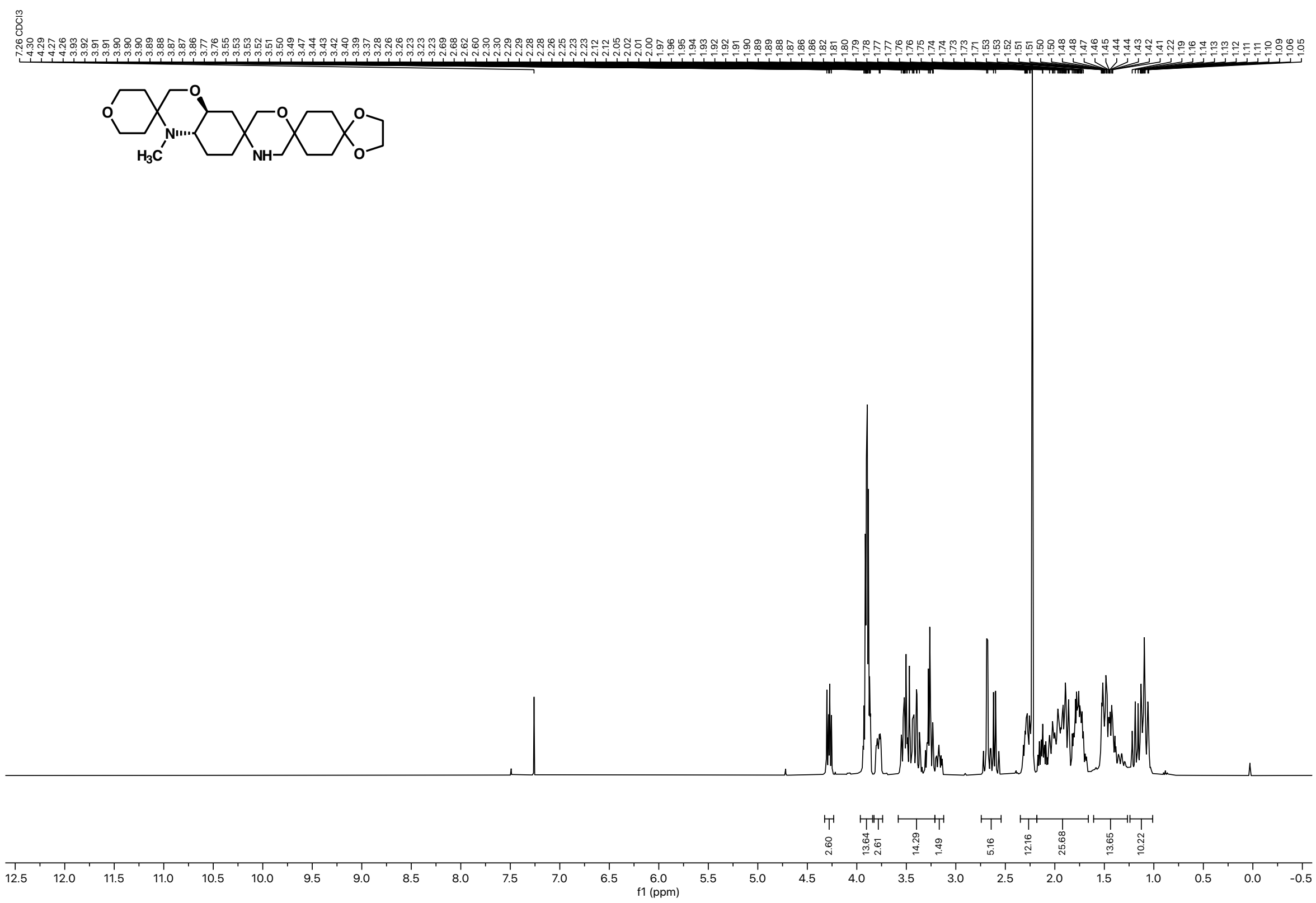

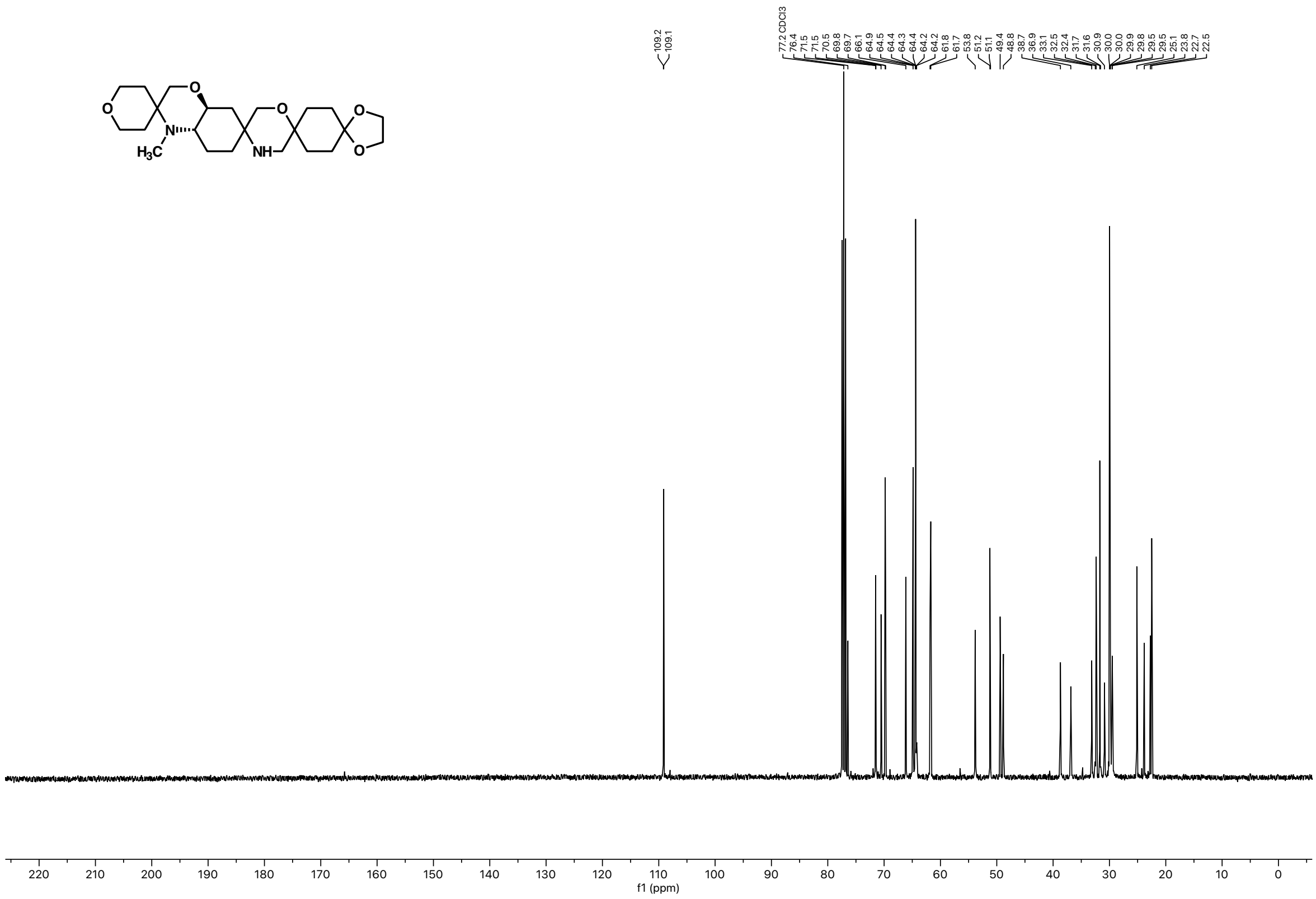

Compound 48: ^{1}H NMR ($CDCl_3$) top, ^{13}C NMR ($CDCl_3$) bottom

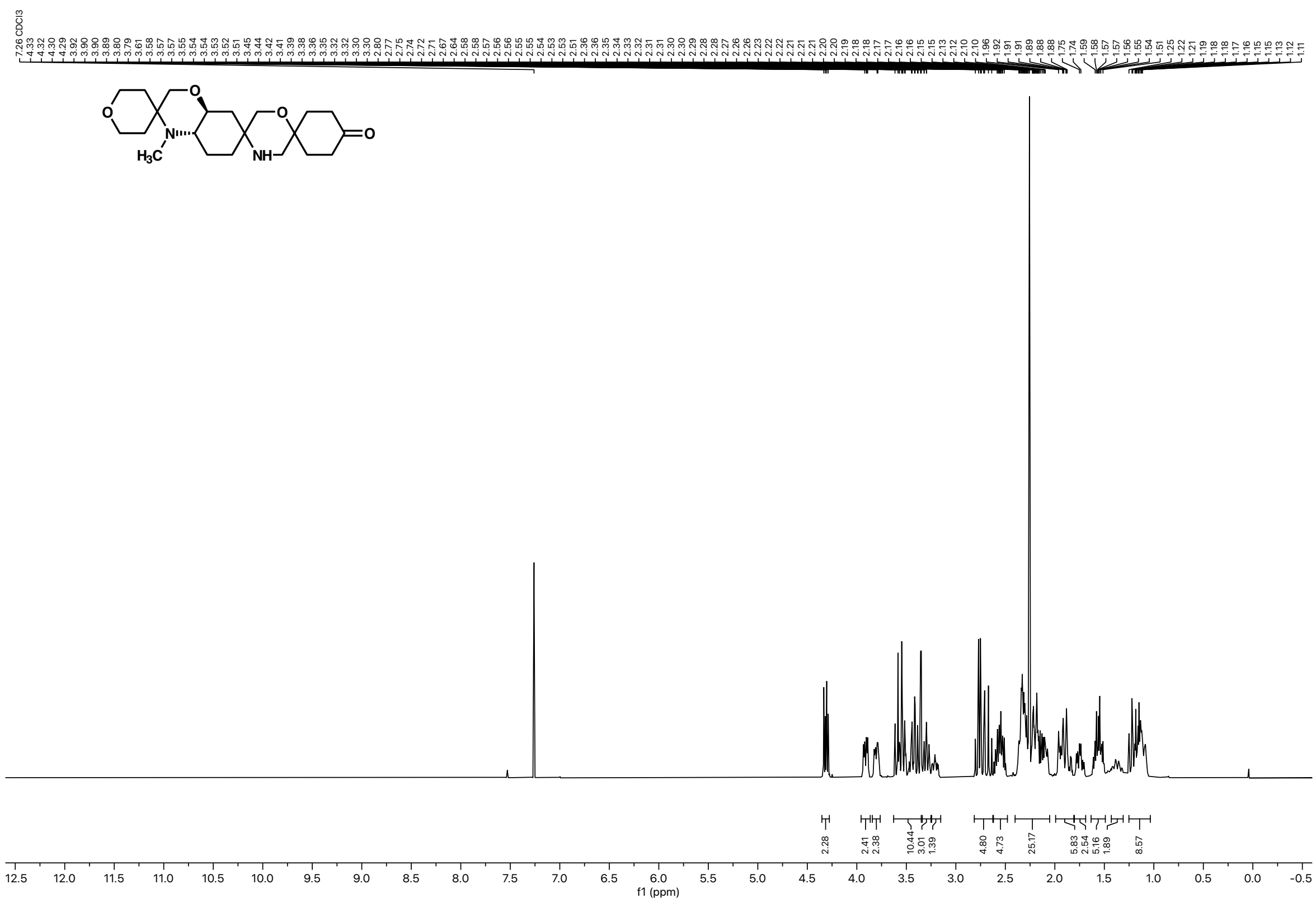

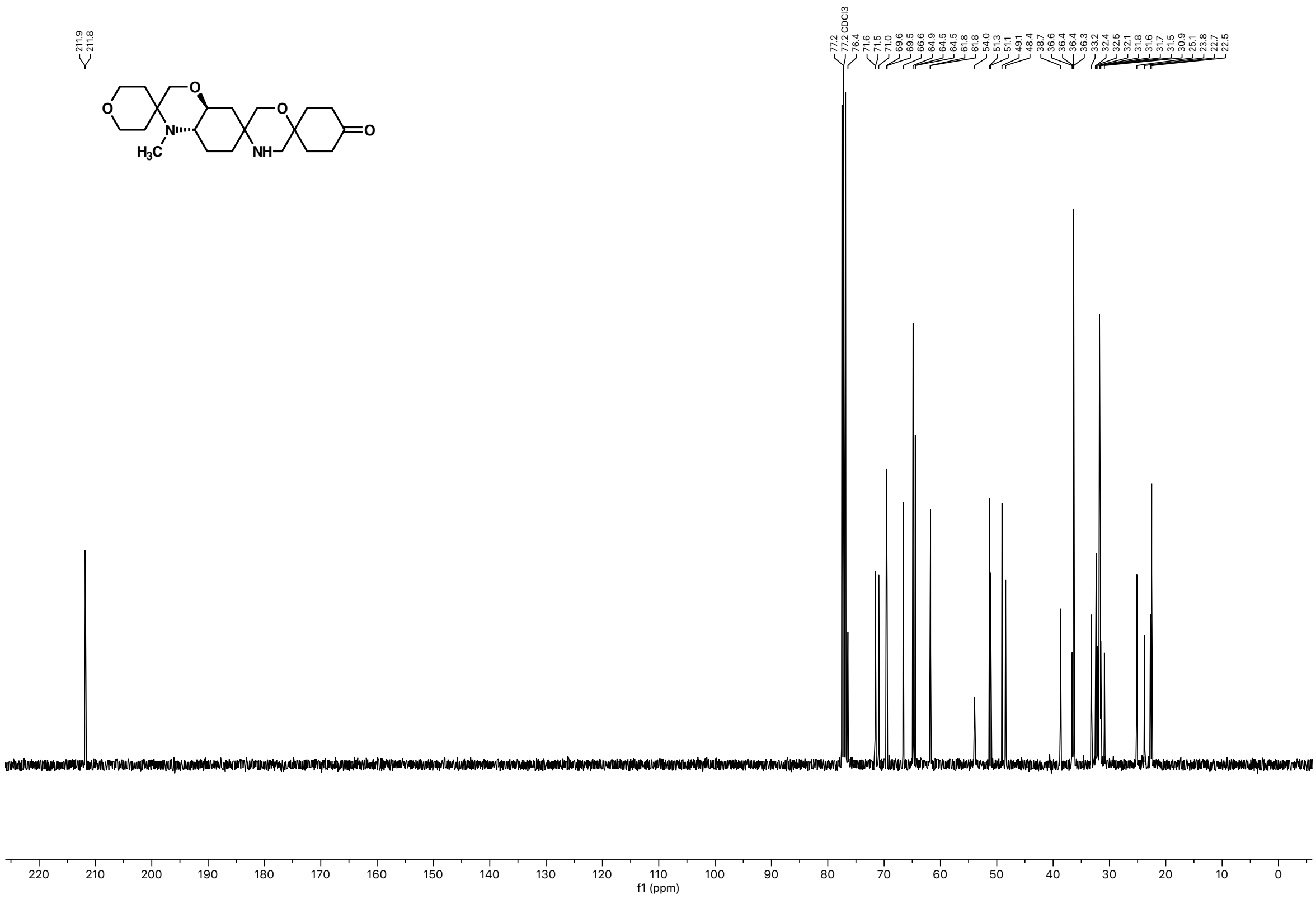

Compound 49: ^{1}H NMR ($CDCl_3$) top, ^{13}C NMR ($CDCl_3$) bottom

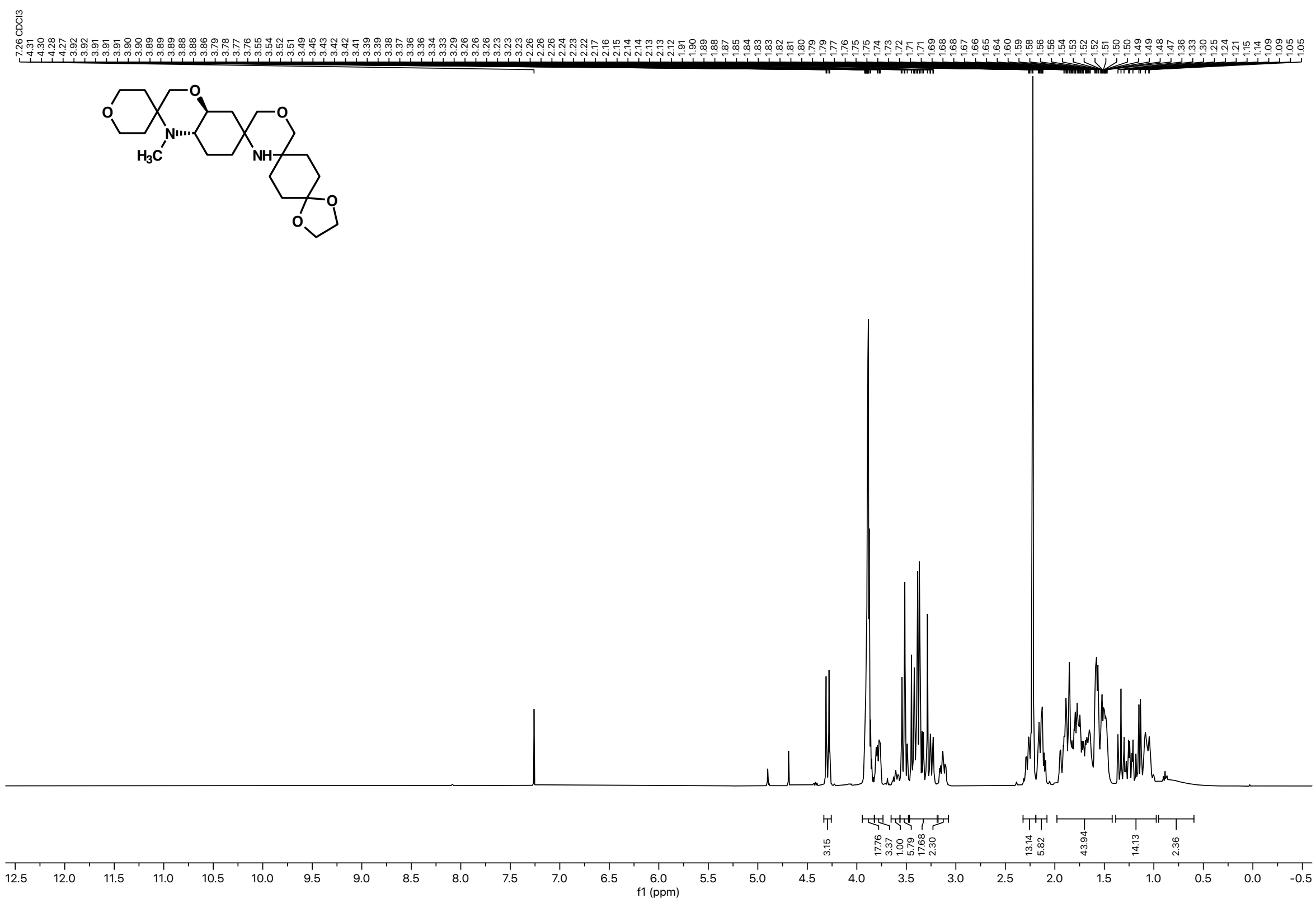

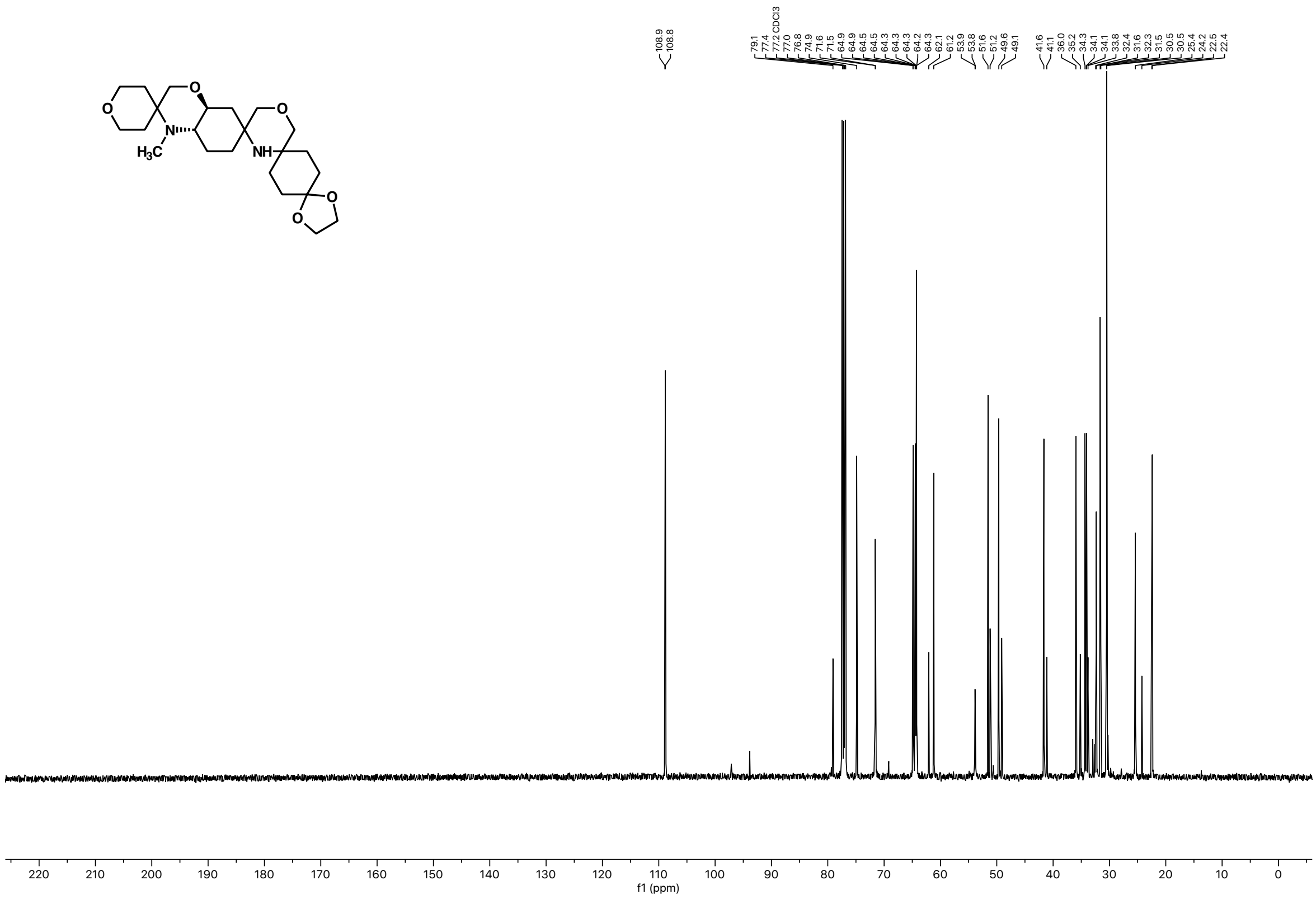

Compound 50: ^{1}H NMR ($CDCl_3$) top, ^{13}C NMR ($CDCl_3$) bottom

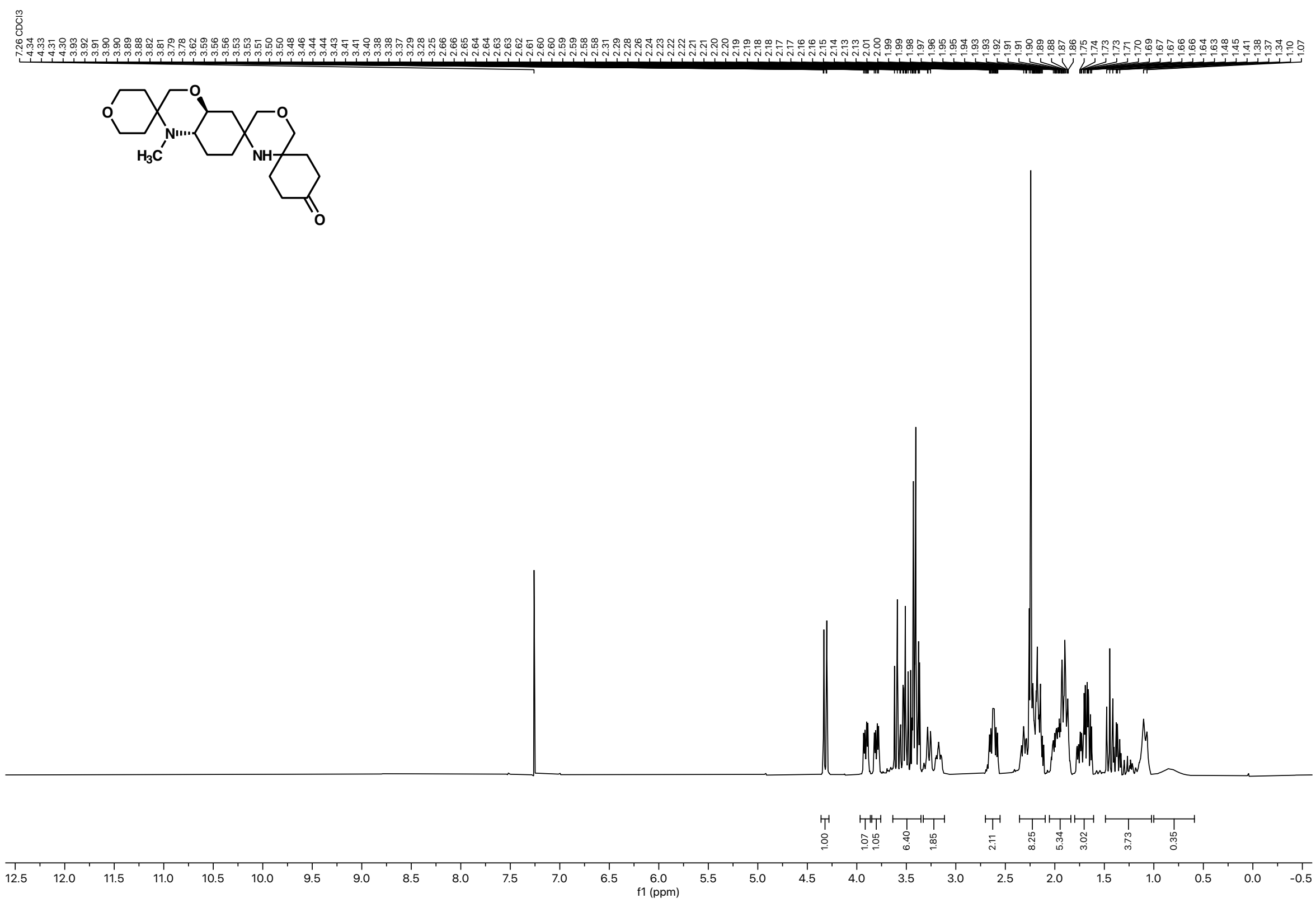

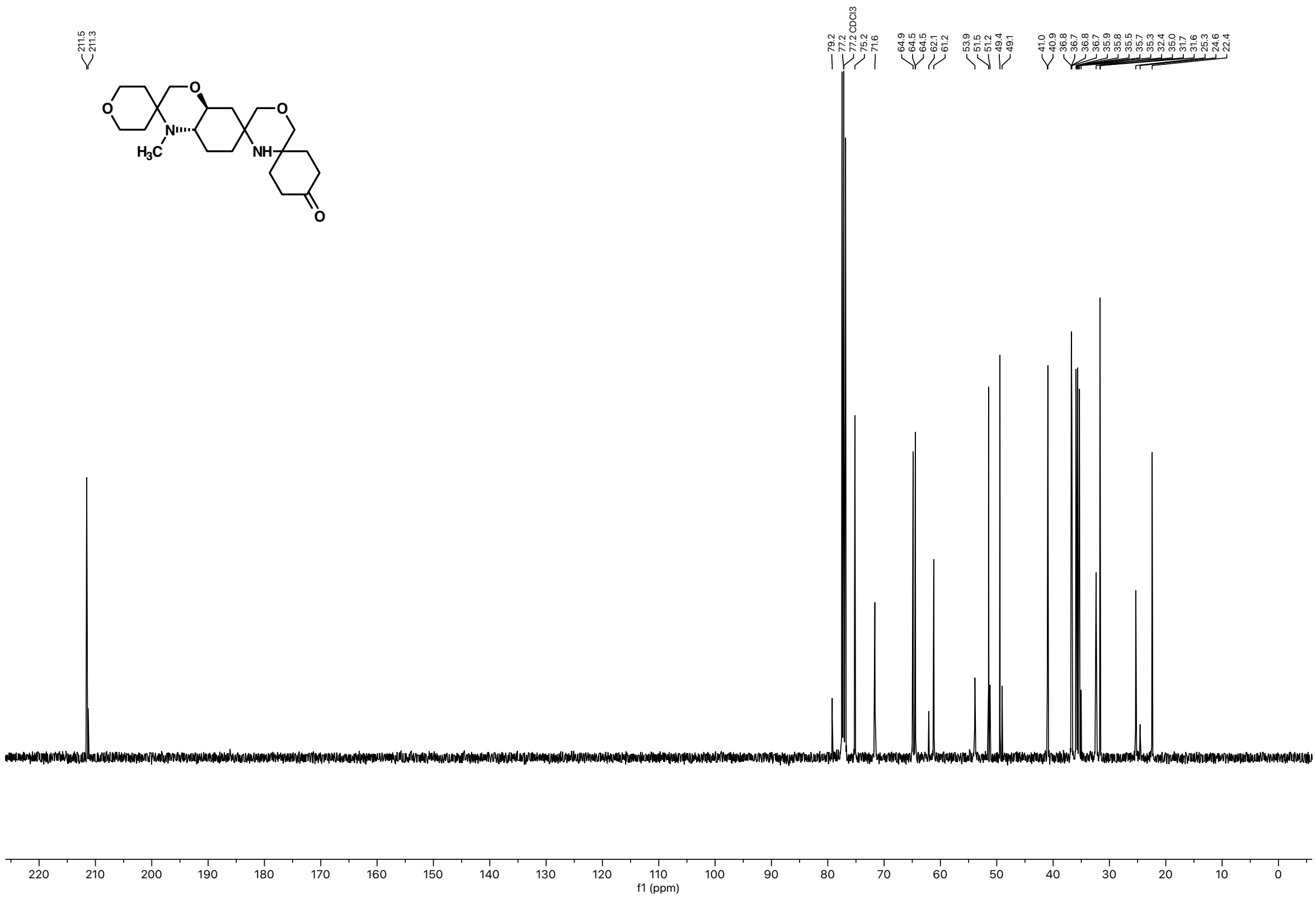

Compound 51: ^{1}H NMR ($CDCl_3$) top, ^{13}C NMR ($CDCl_3$) bottom

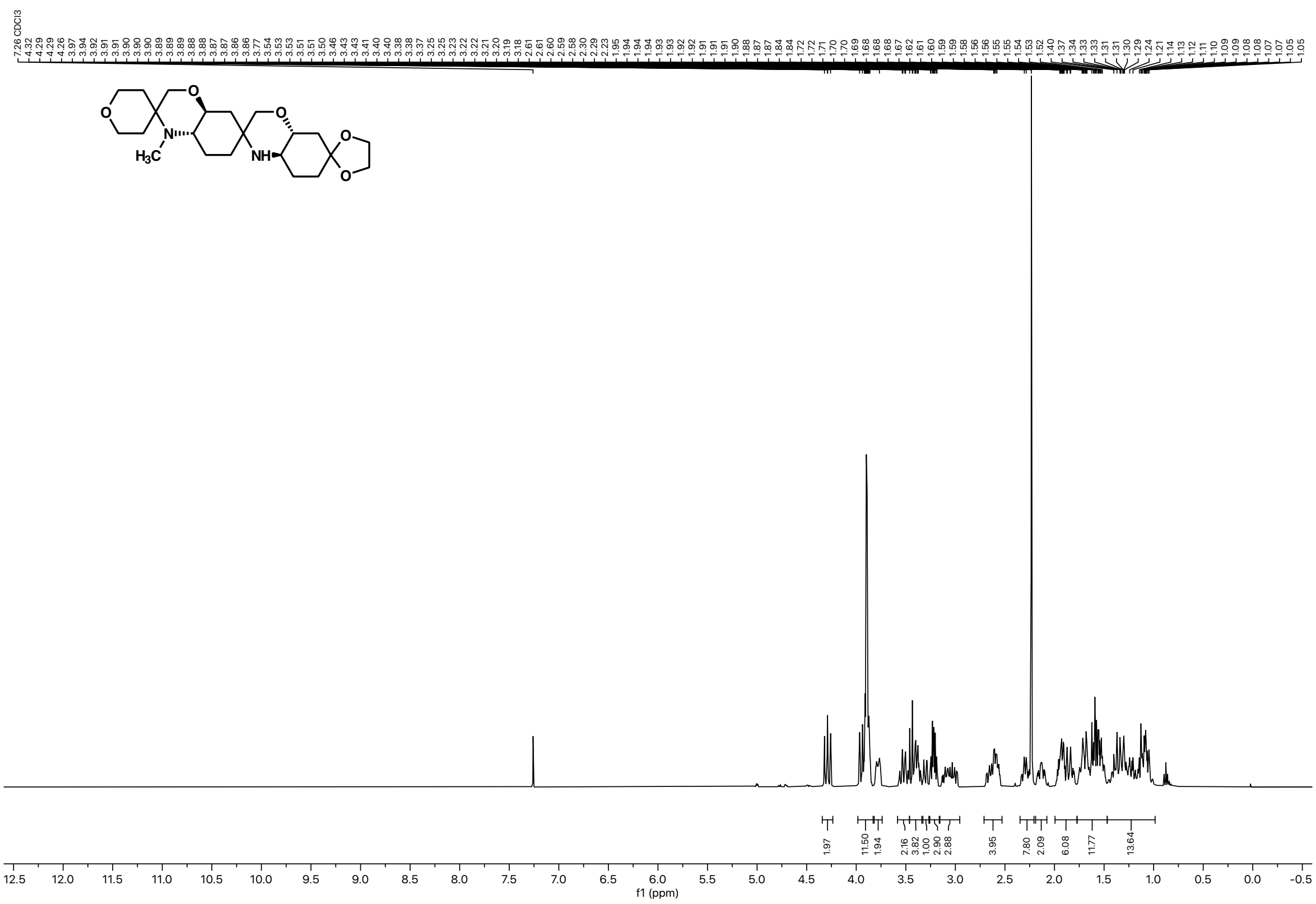

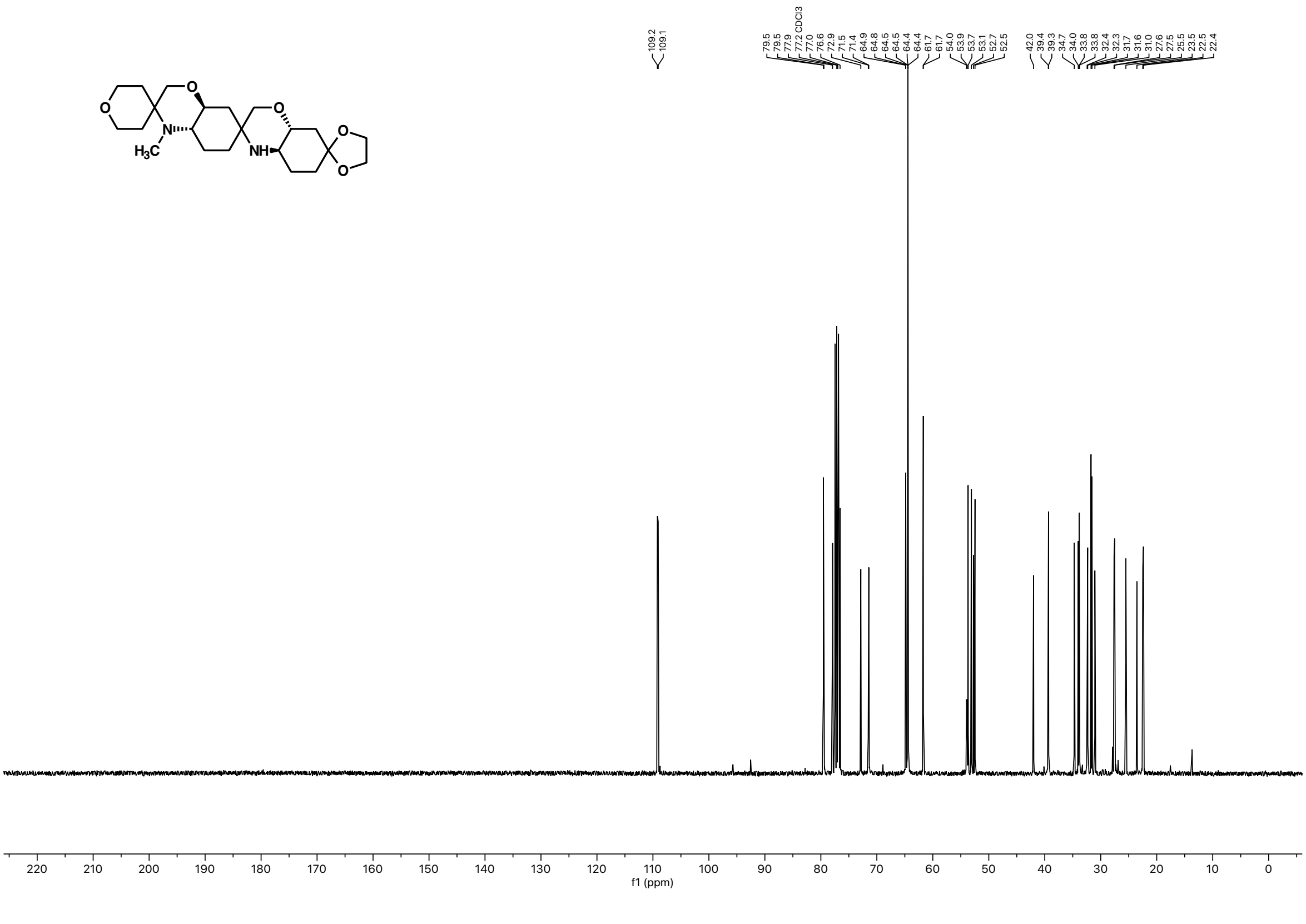

Compound 52: ¹H NMR ($CDCl_3$) top, ¹³C NMR ($CDCl_3$) bottom

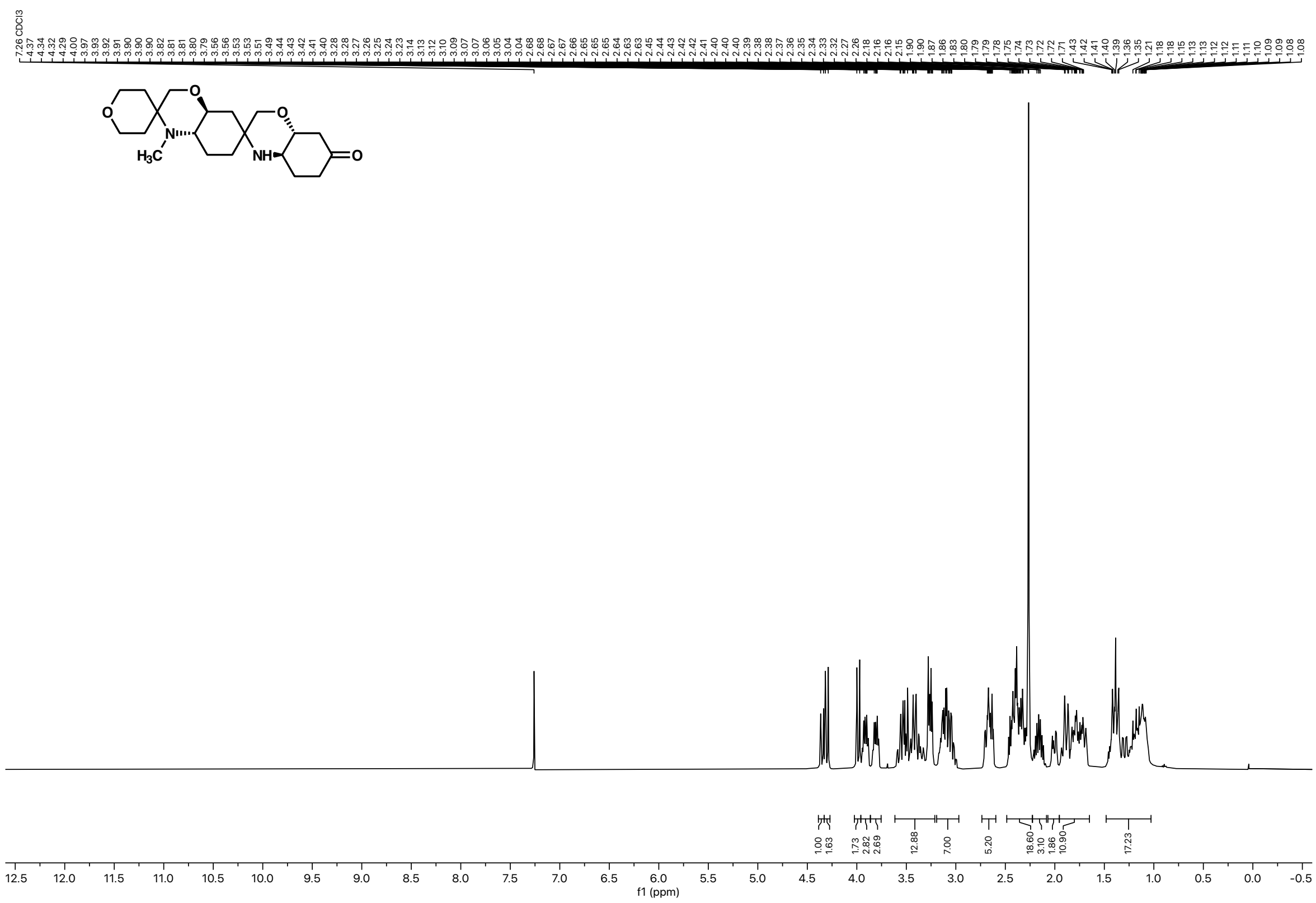

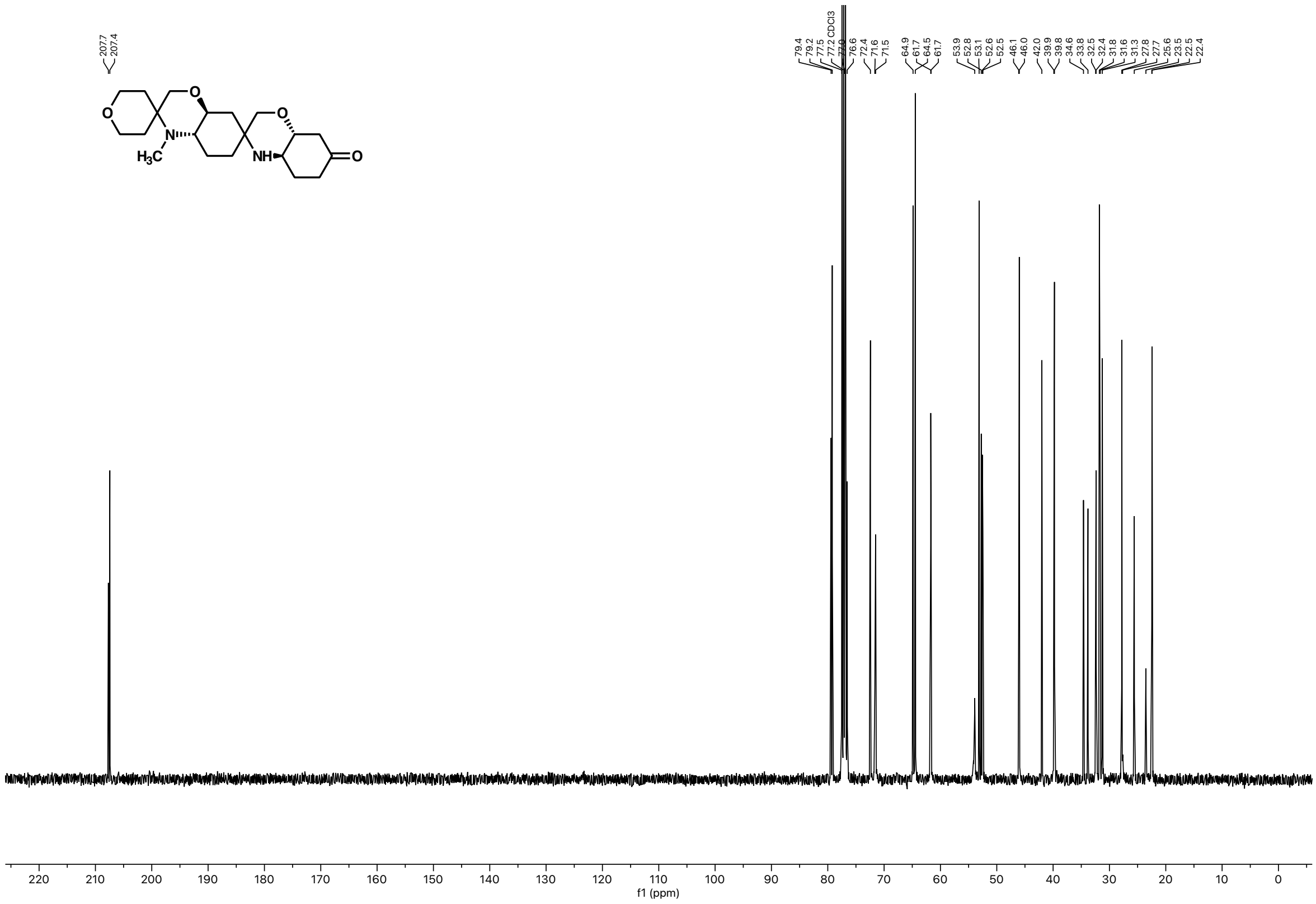

Compound 53: ^{1}H NMR ($CDCl_3$) top, ^{13}C NMR ($CDCl_3$) bottom

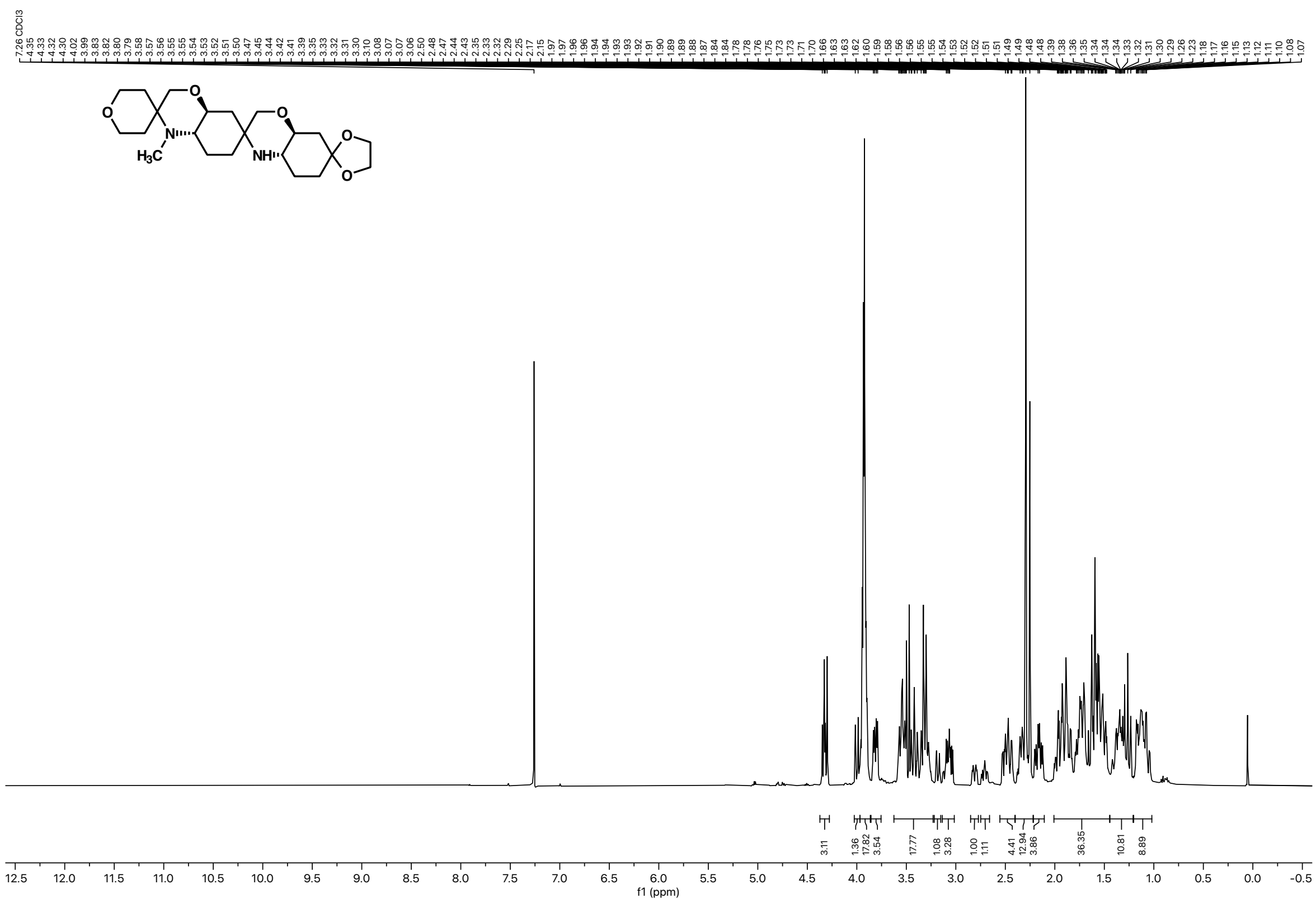

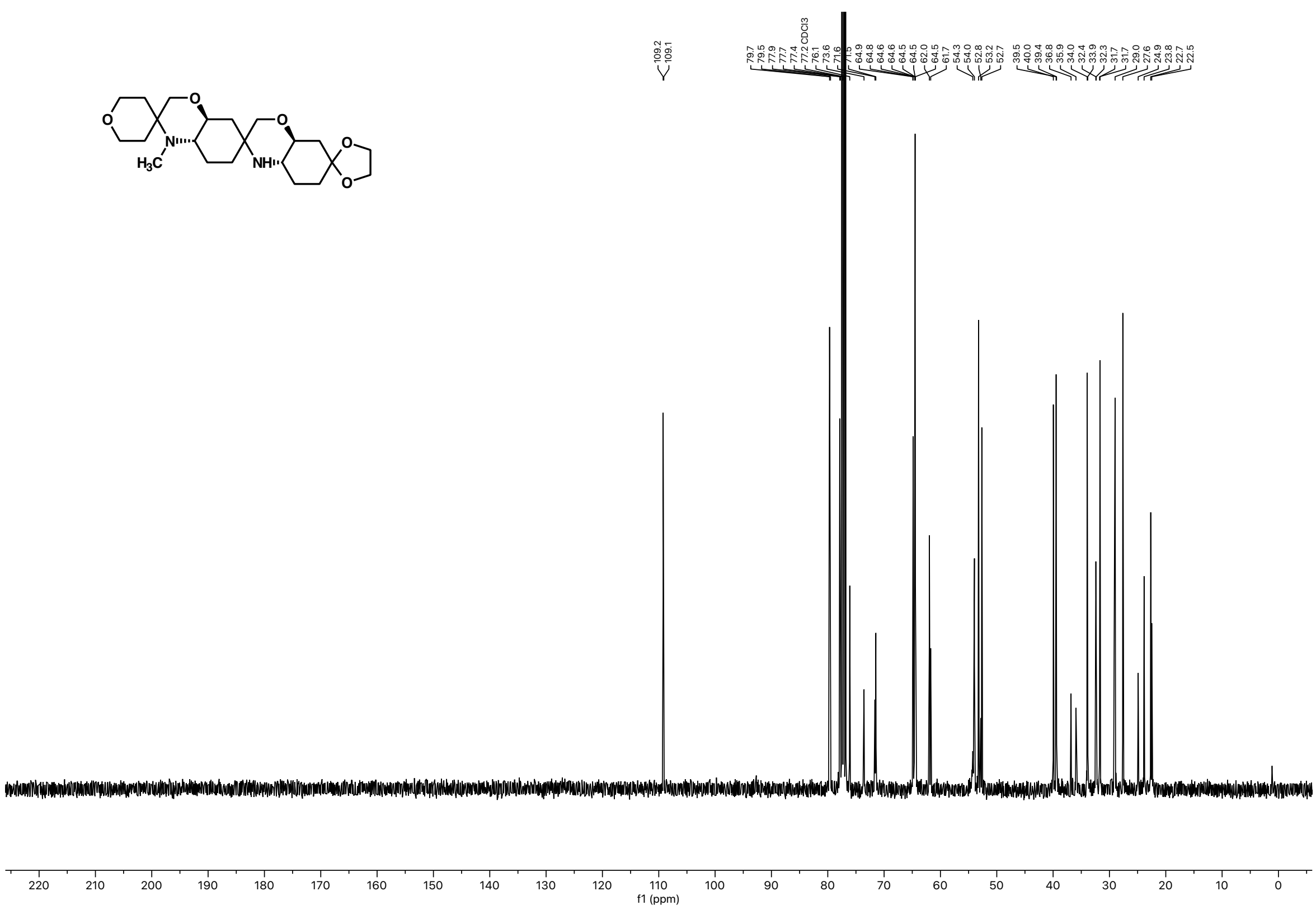

Compound 54: ^{1}H NMR ($CDCl_3$) top, ^{13}C NMR ($CDCl_3$) bottom

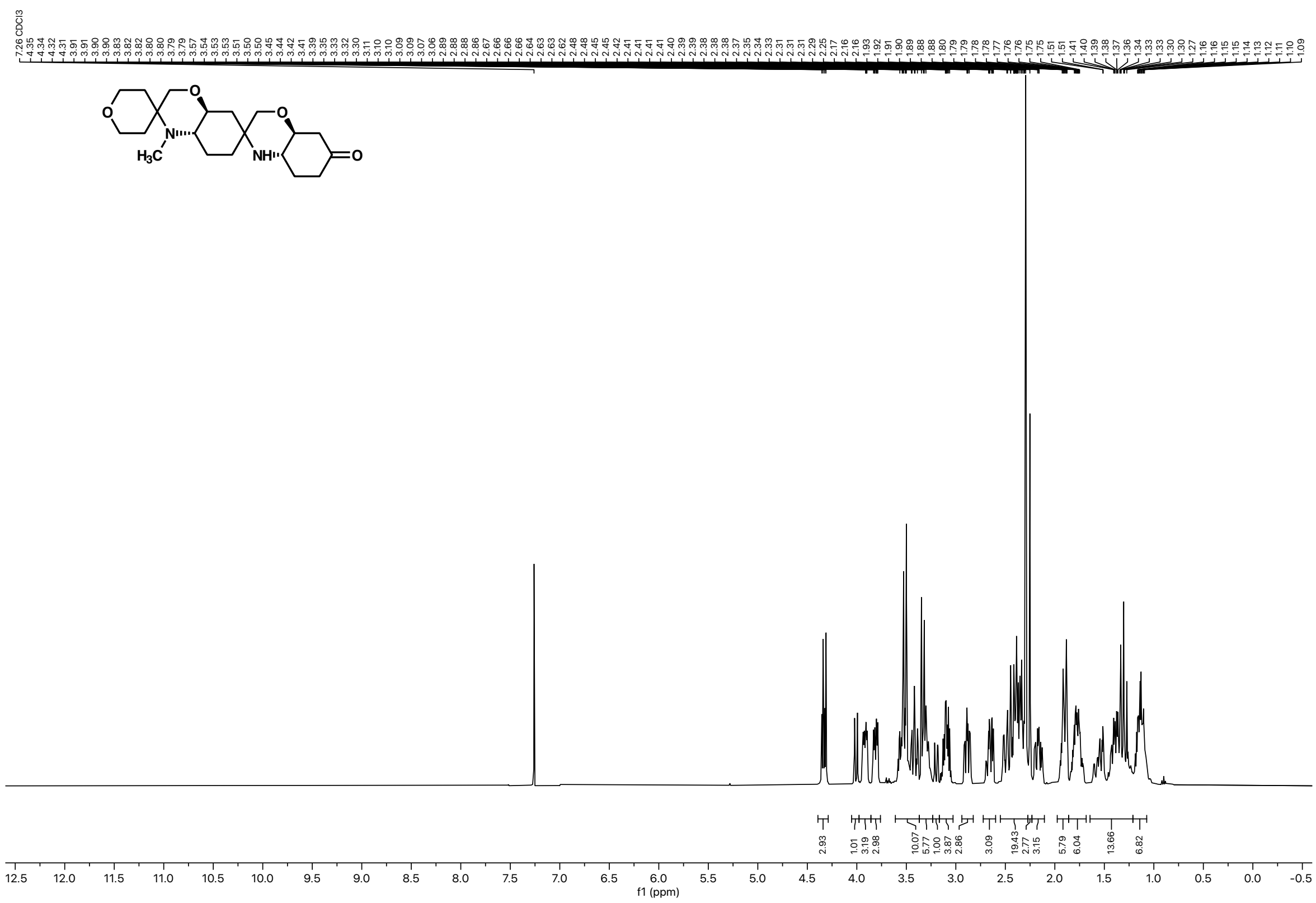

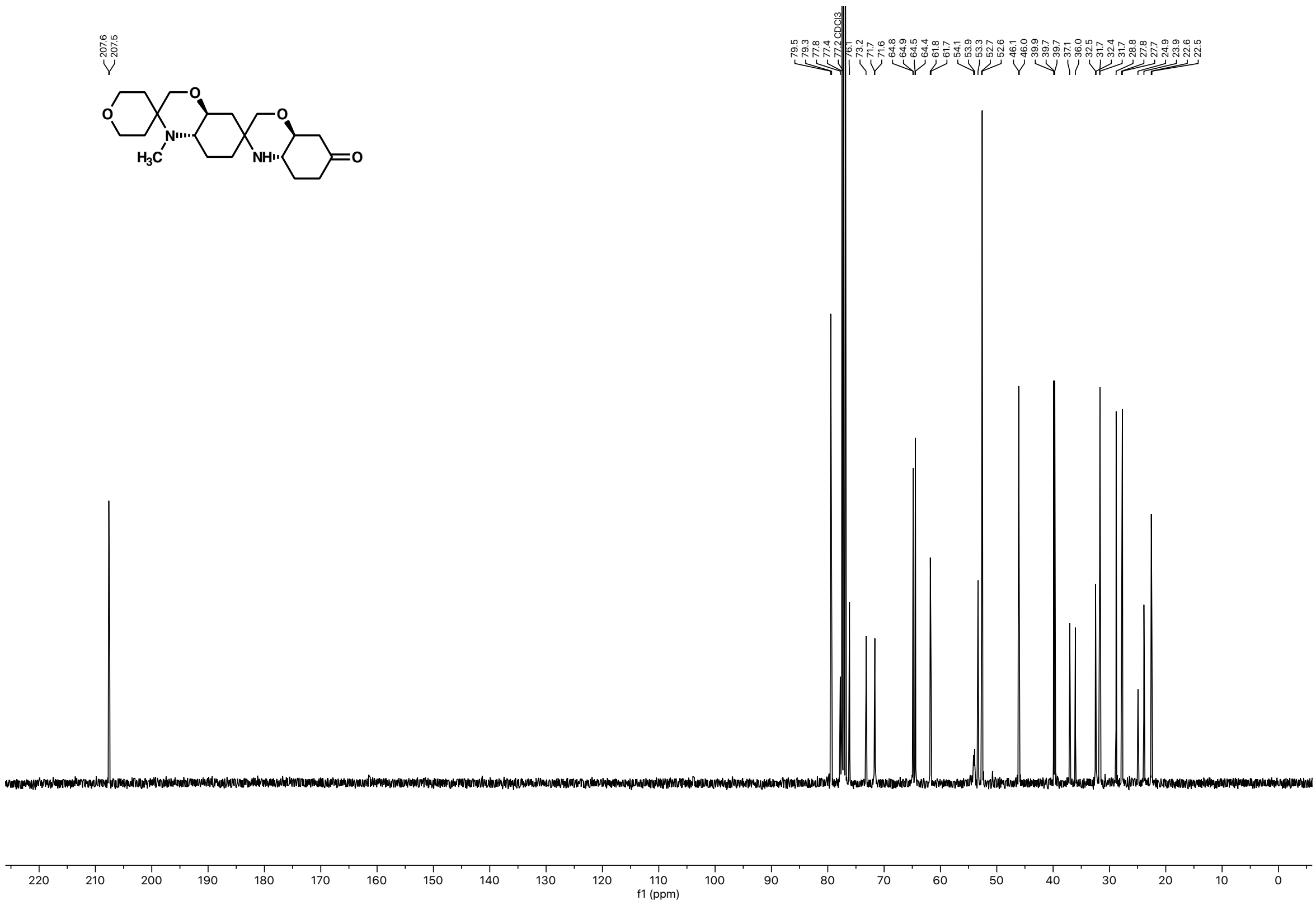

Compound 55: ^{1}H NMR ($CDCl_3$) top, ^{13}C NMR ($CDCl_3$) bottom

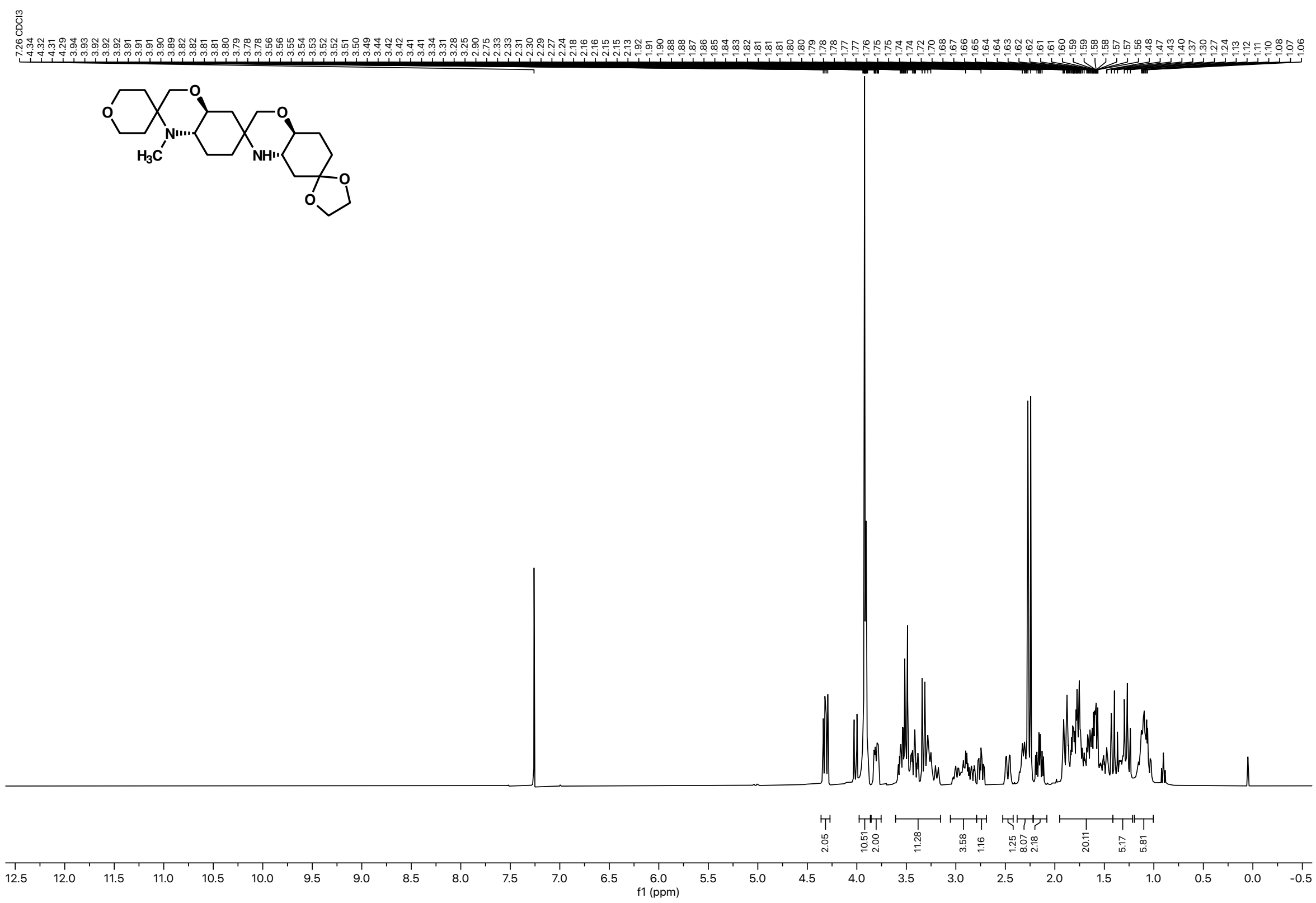

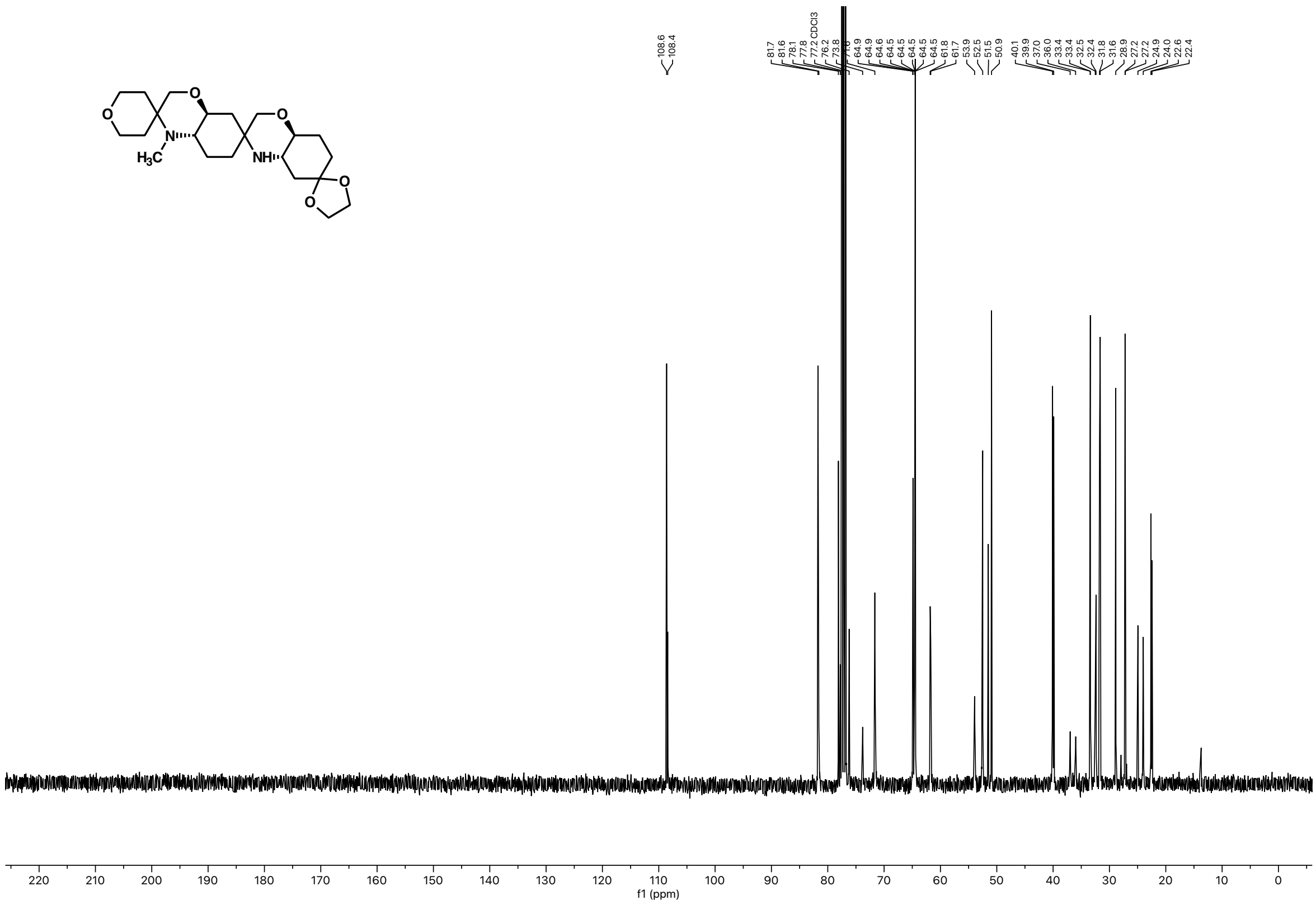

Compound 56: ^{1}H NMR ($CDCl_3$) top, ^{13}C NMR ($CDCl_3$) bottom

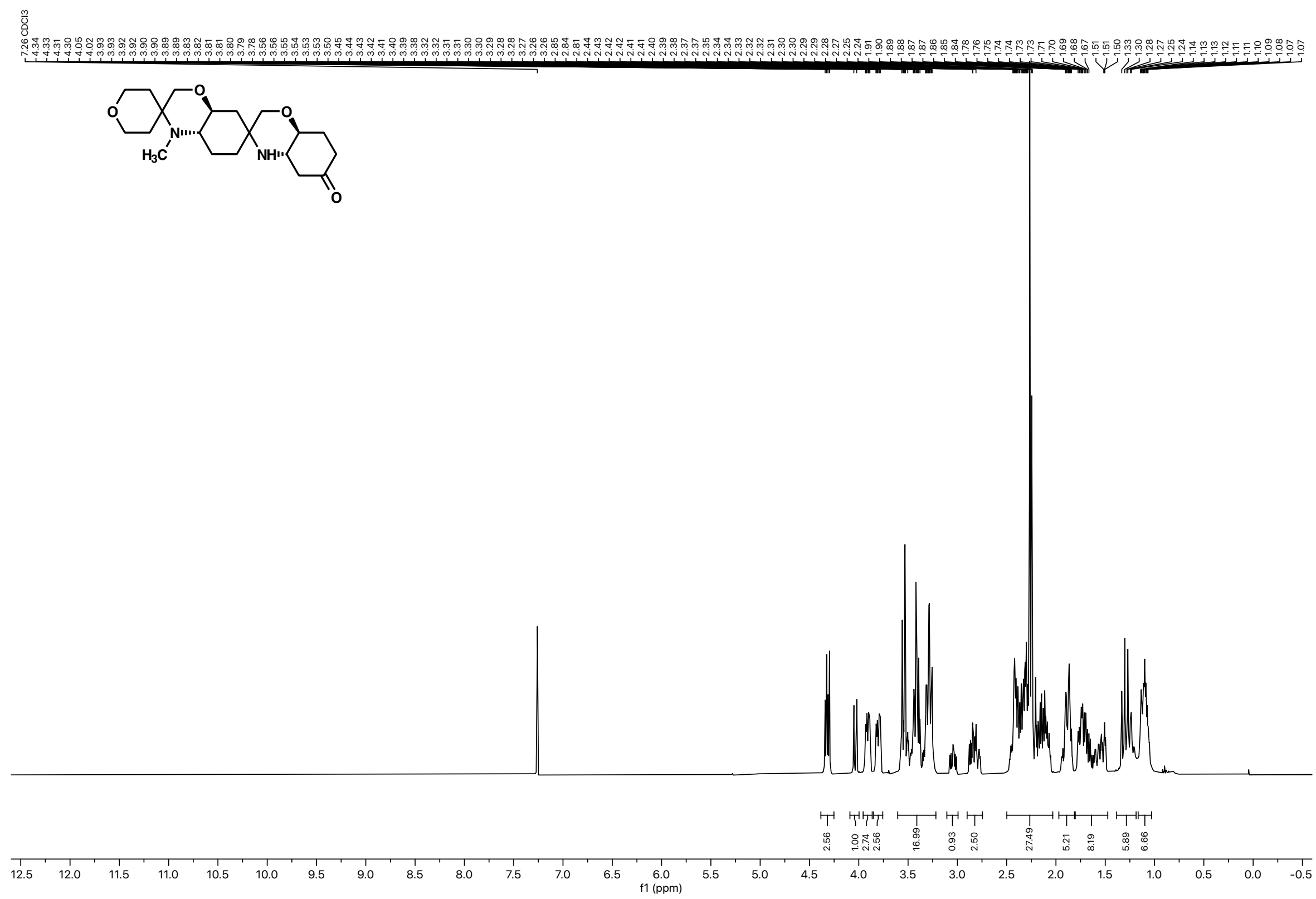

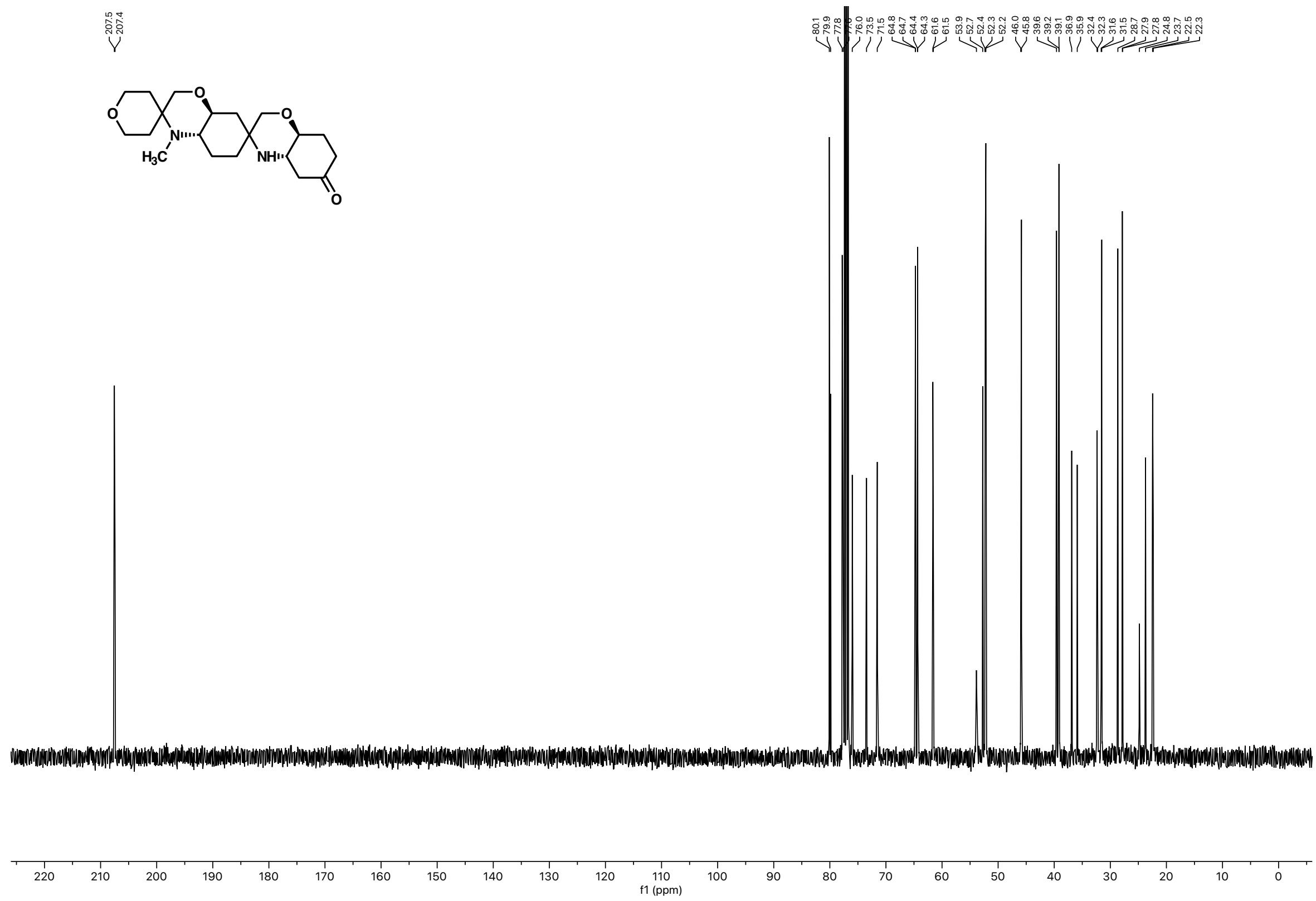

6.1.1.1 NMR of separated diastereomers

Compound 57a: ^{1}H NMR ($CDCl_3$) top, ^{13}C NMR ($CDCl_3$) bottom

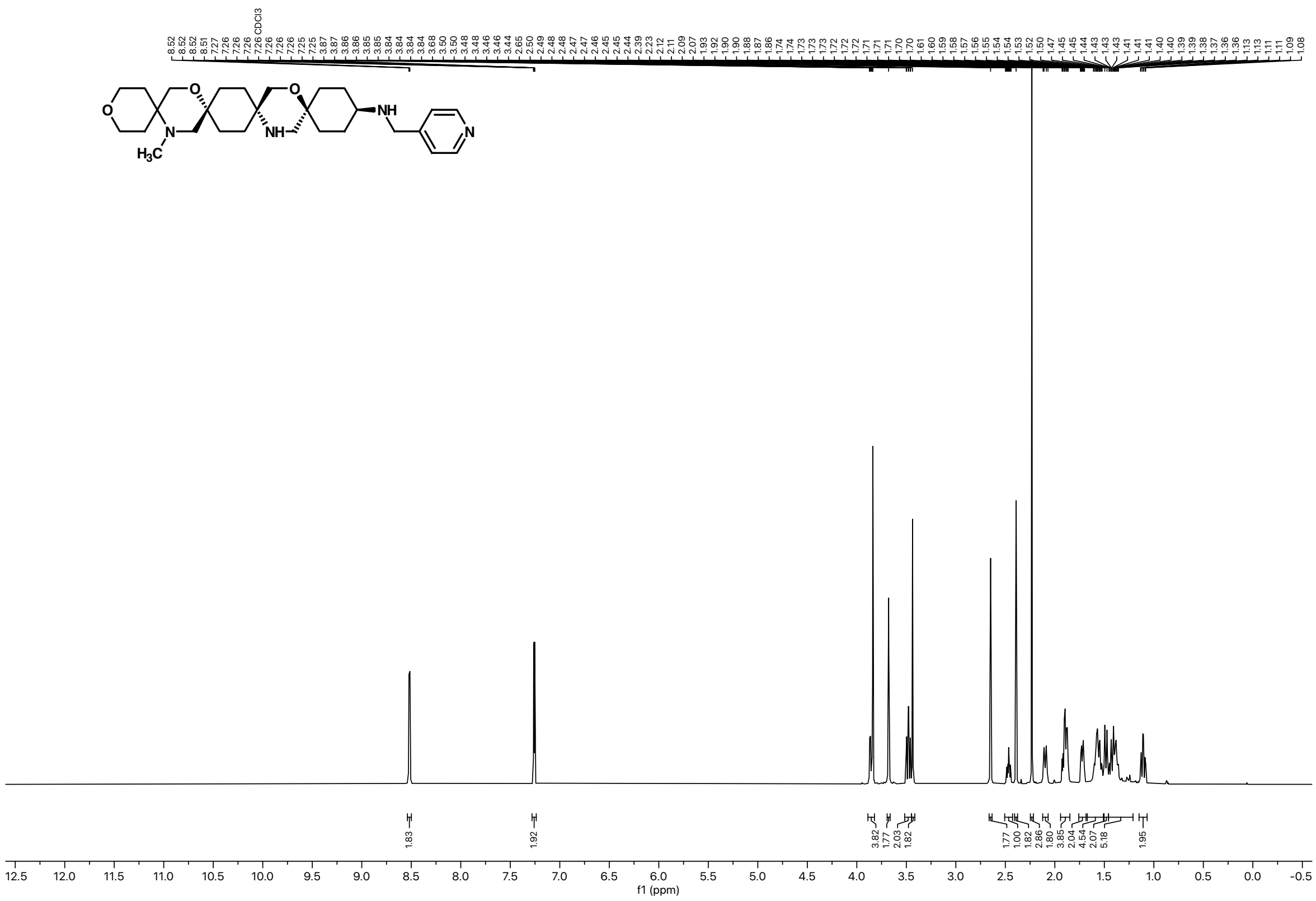

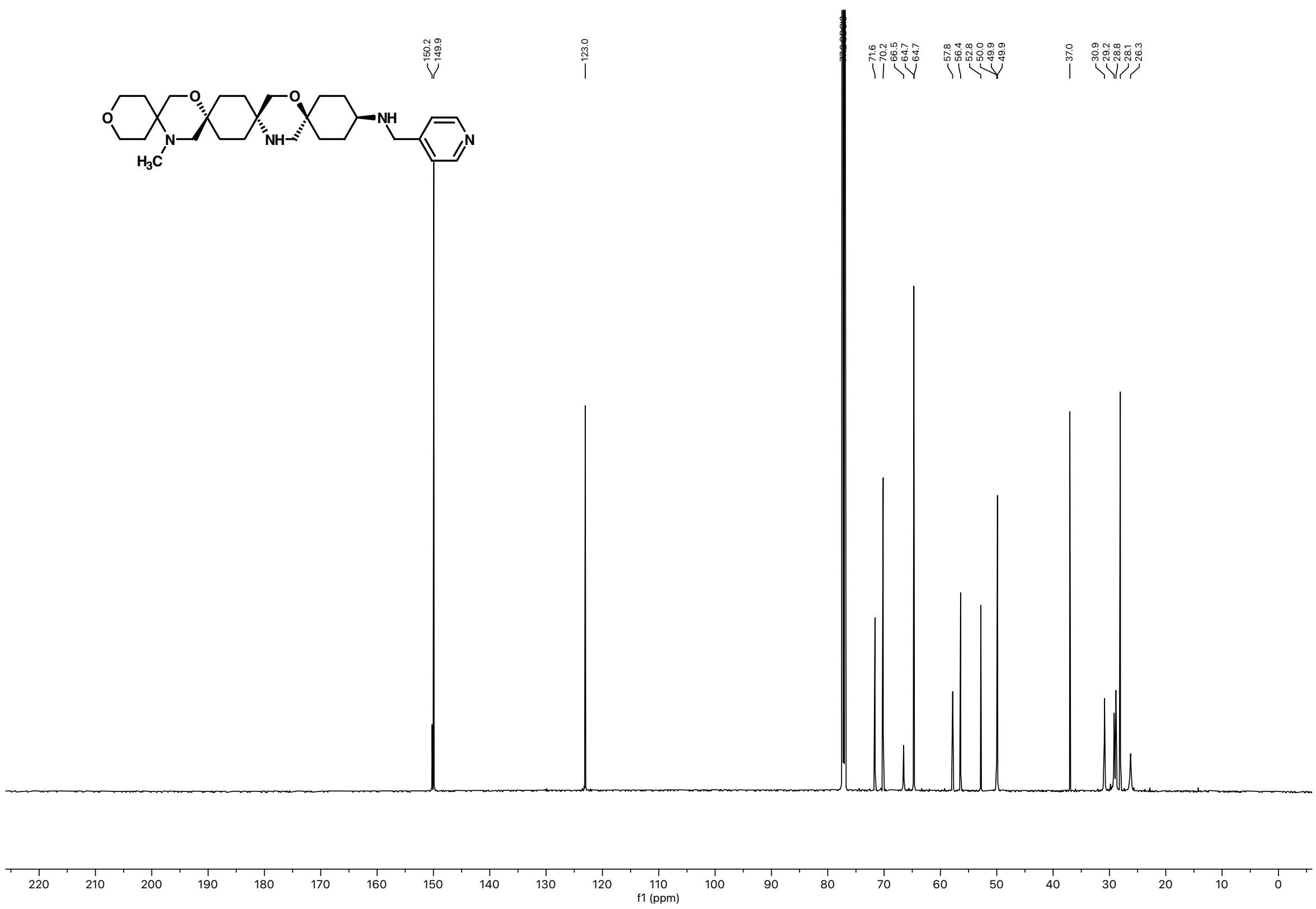

Compound 57b: ^{1}H NMR ($CDCl_3$) top, ^{13}C NMR ($CDCl_3$) bottom

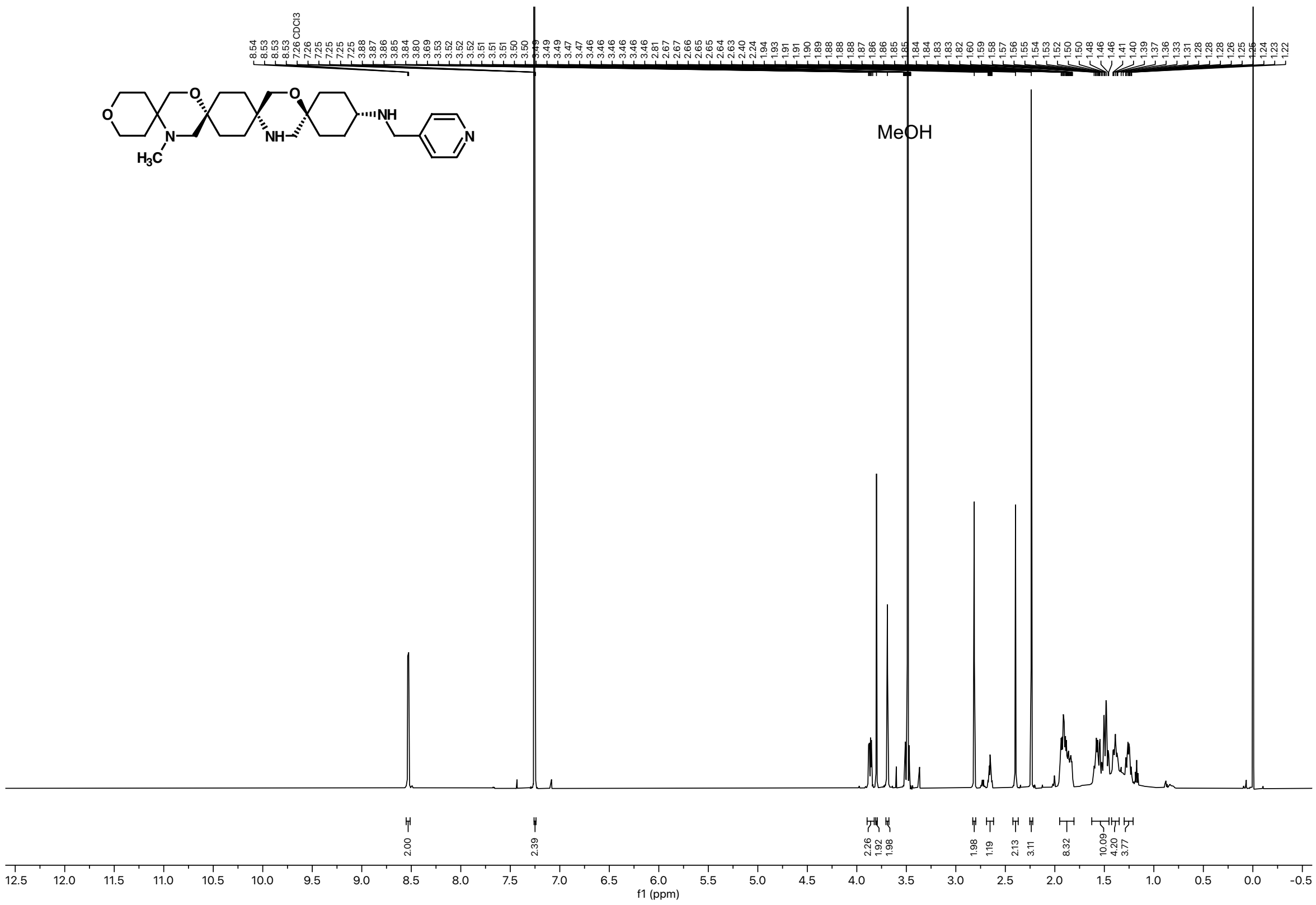

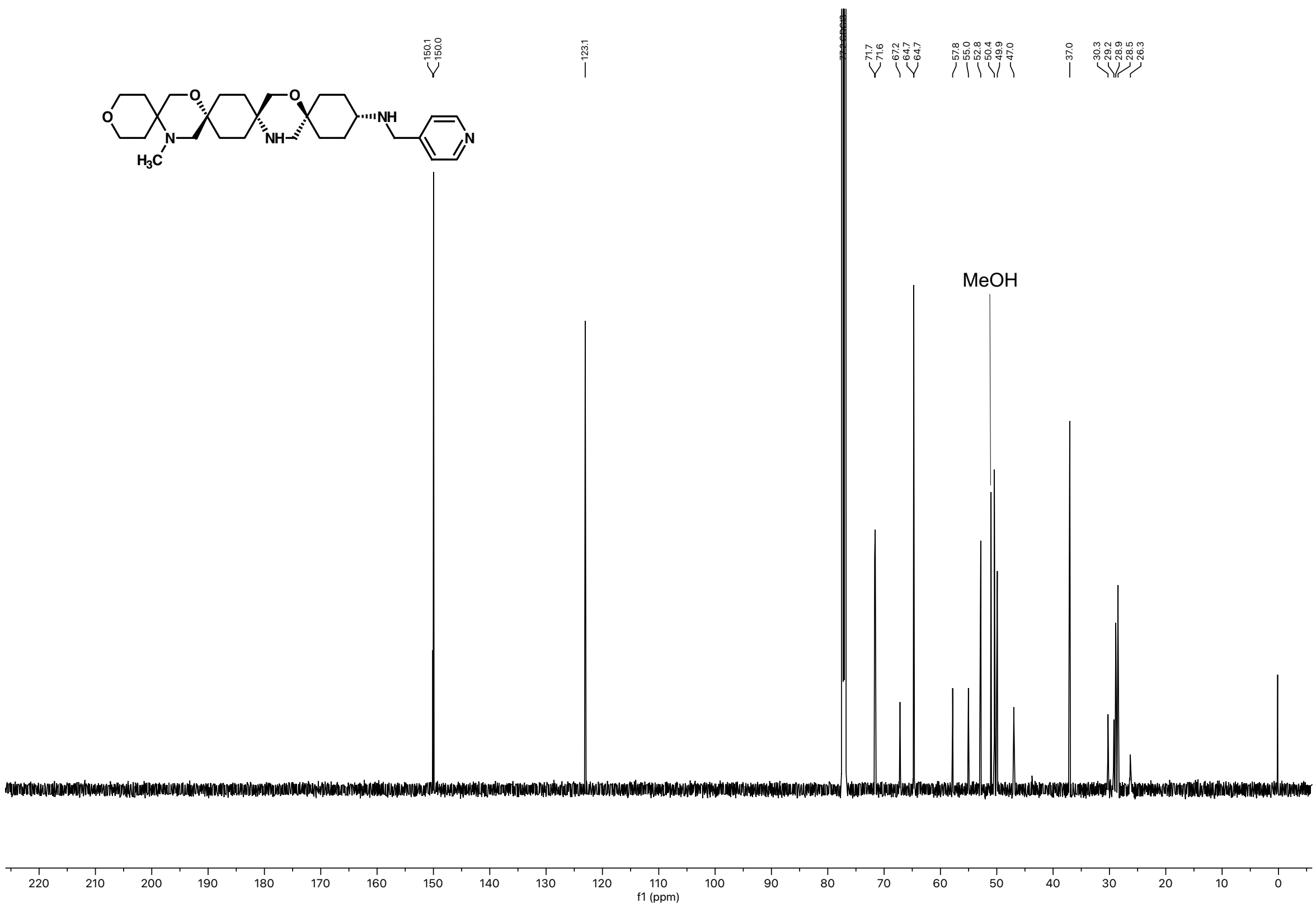

Compound 57c: ^{1}H NMR ($CDCl_3$) top, ^{13}C NMR ($CDCl_3$) bottom

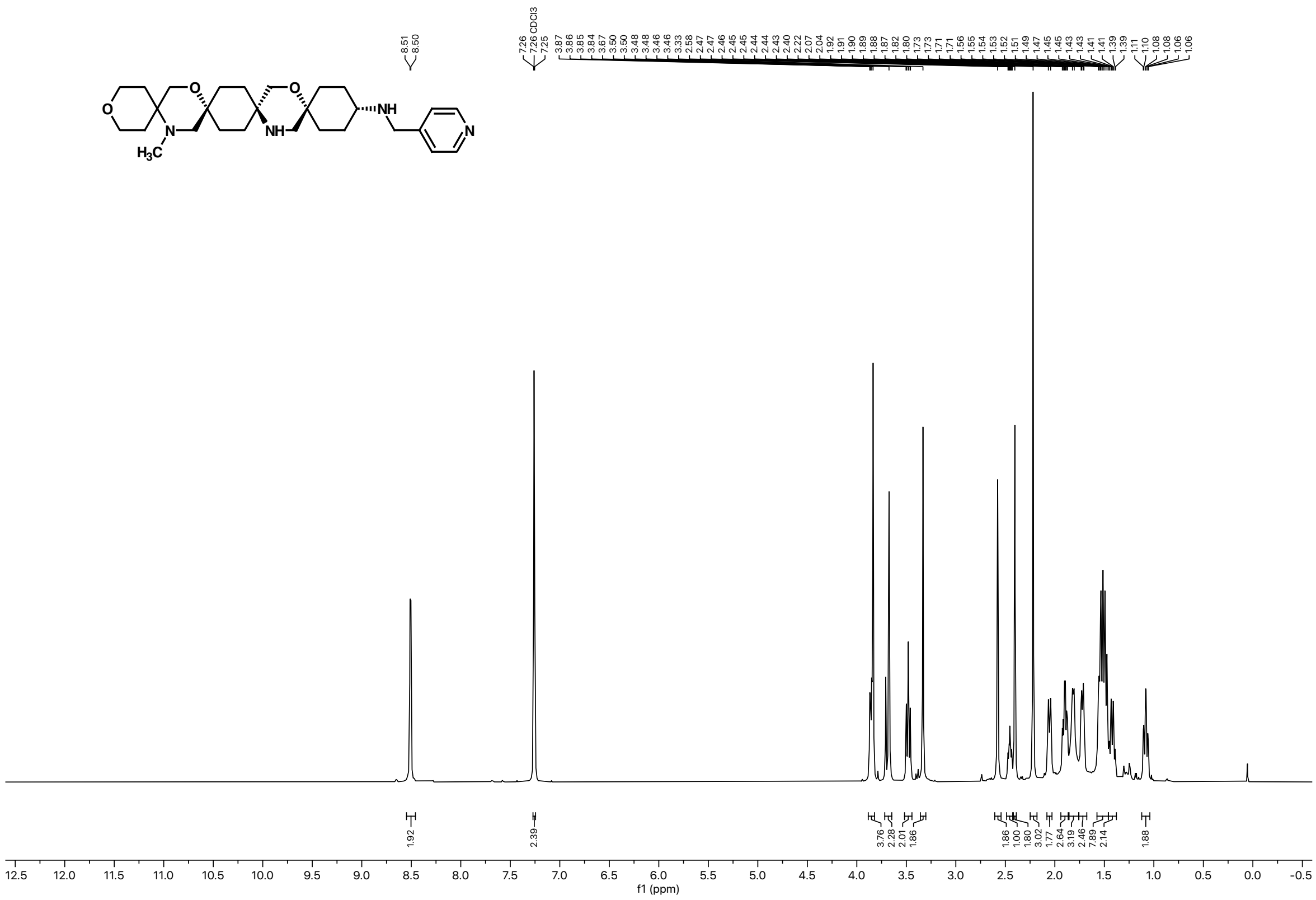

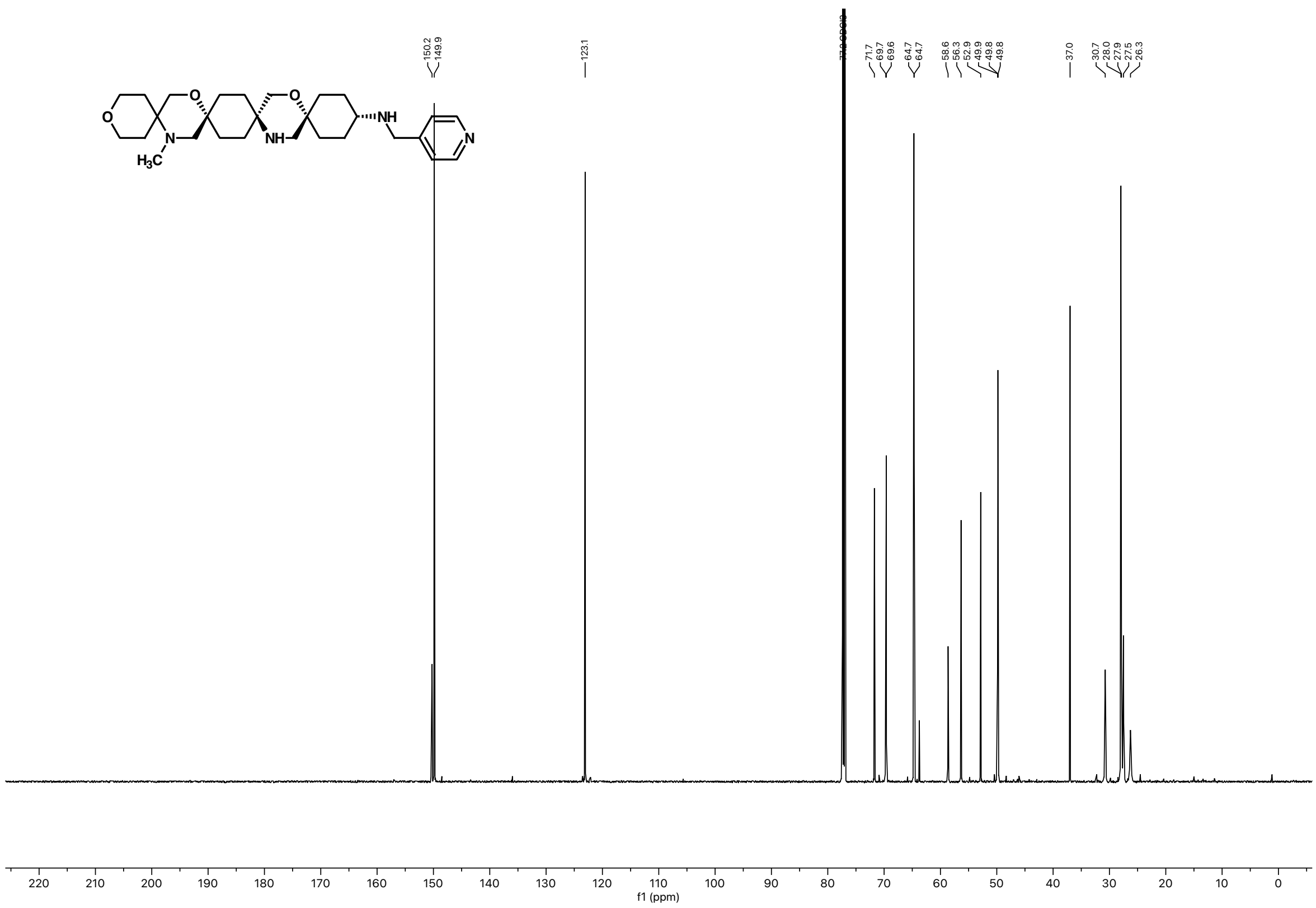

Compound 57d: ^{1}H NMR ($CDCl_3$) top, ^{13}C NMR ($CDCl_3$) bottom

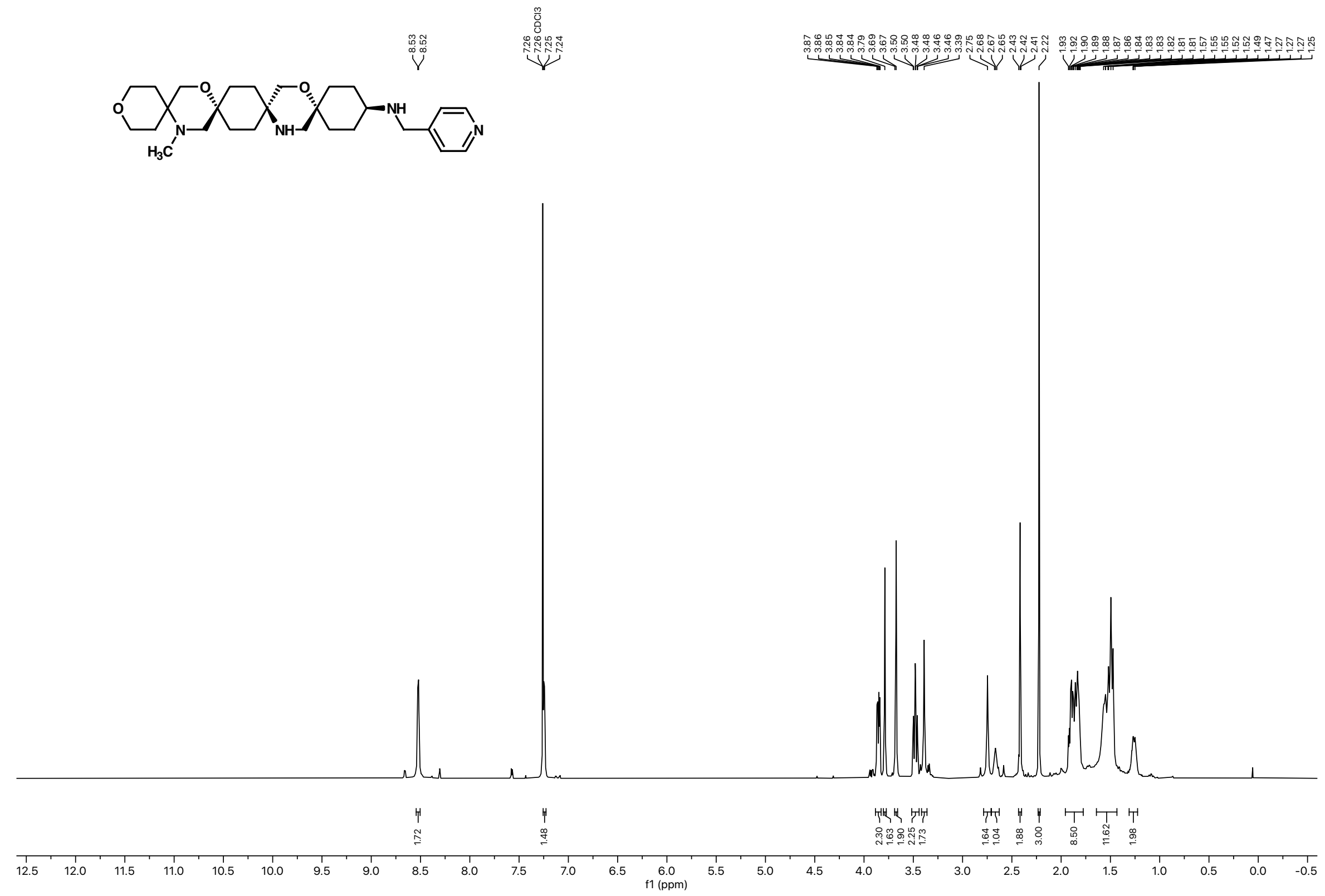

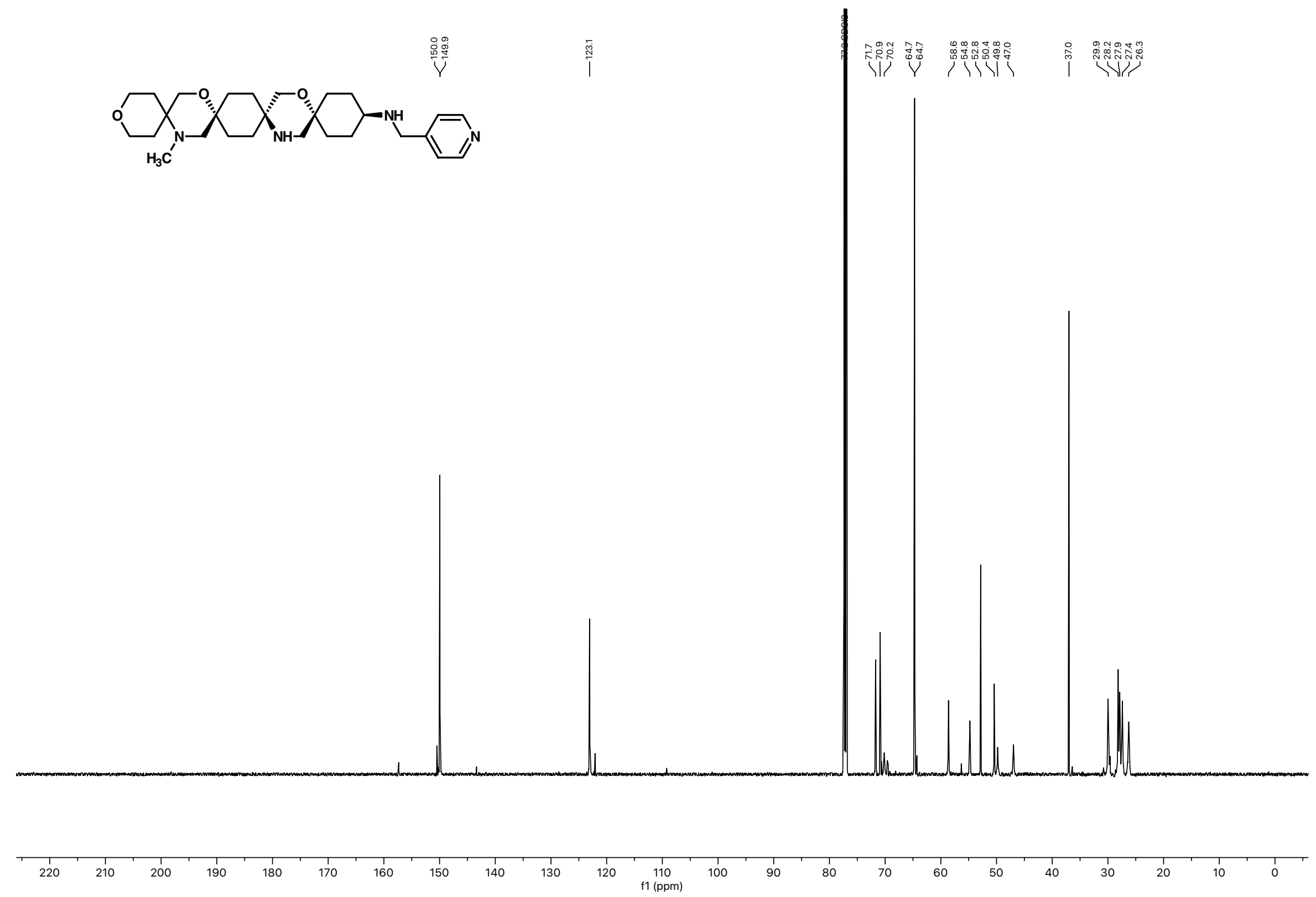

Compound 58a: ^{1}H NMR ($CDCl_3$) top, ^{13}C NMR ($CDCl_3$) bottom

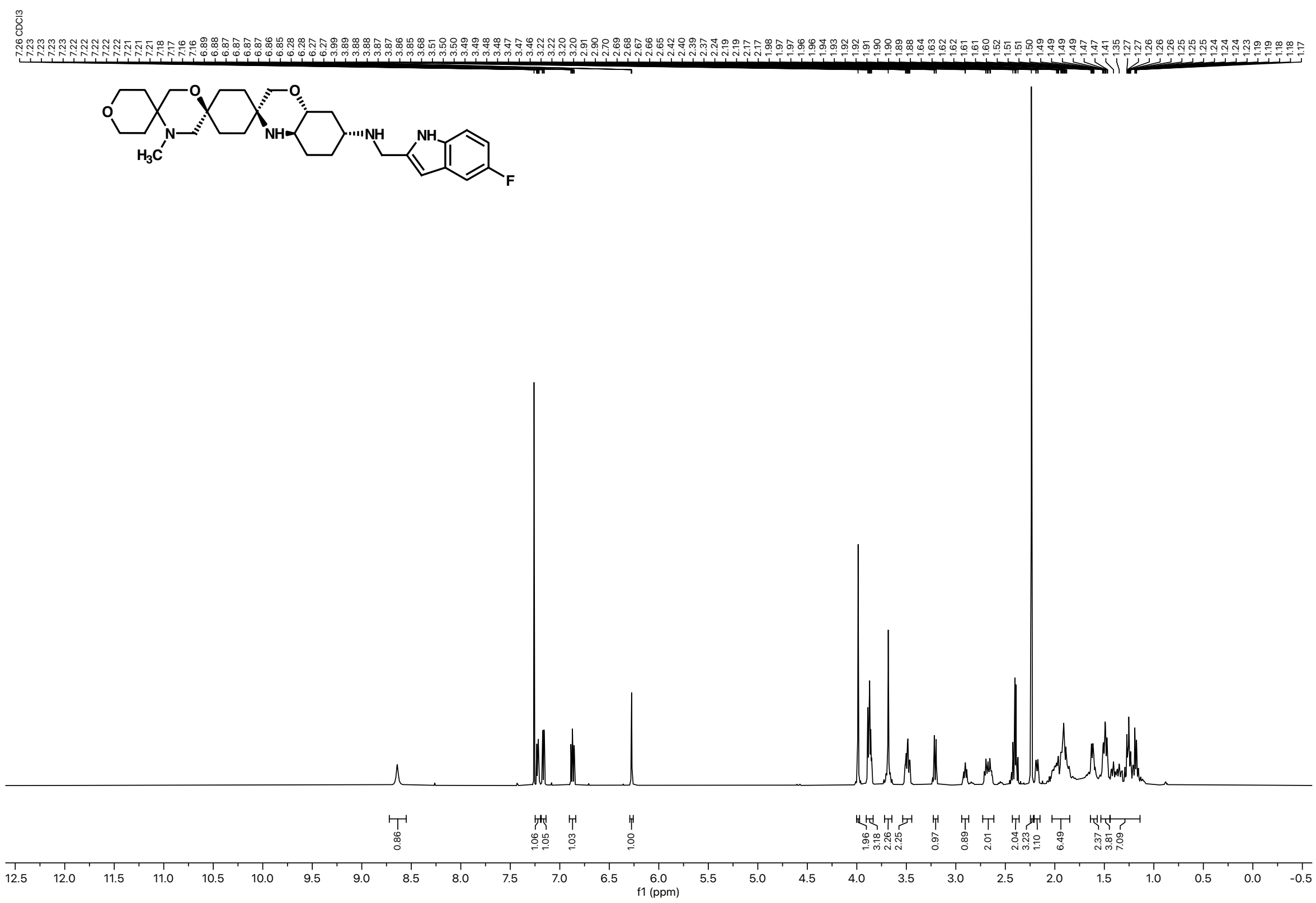

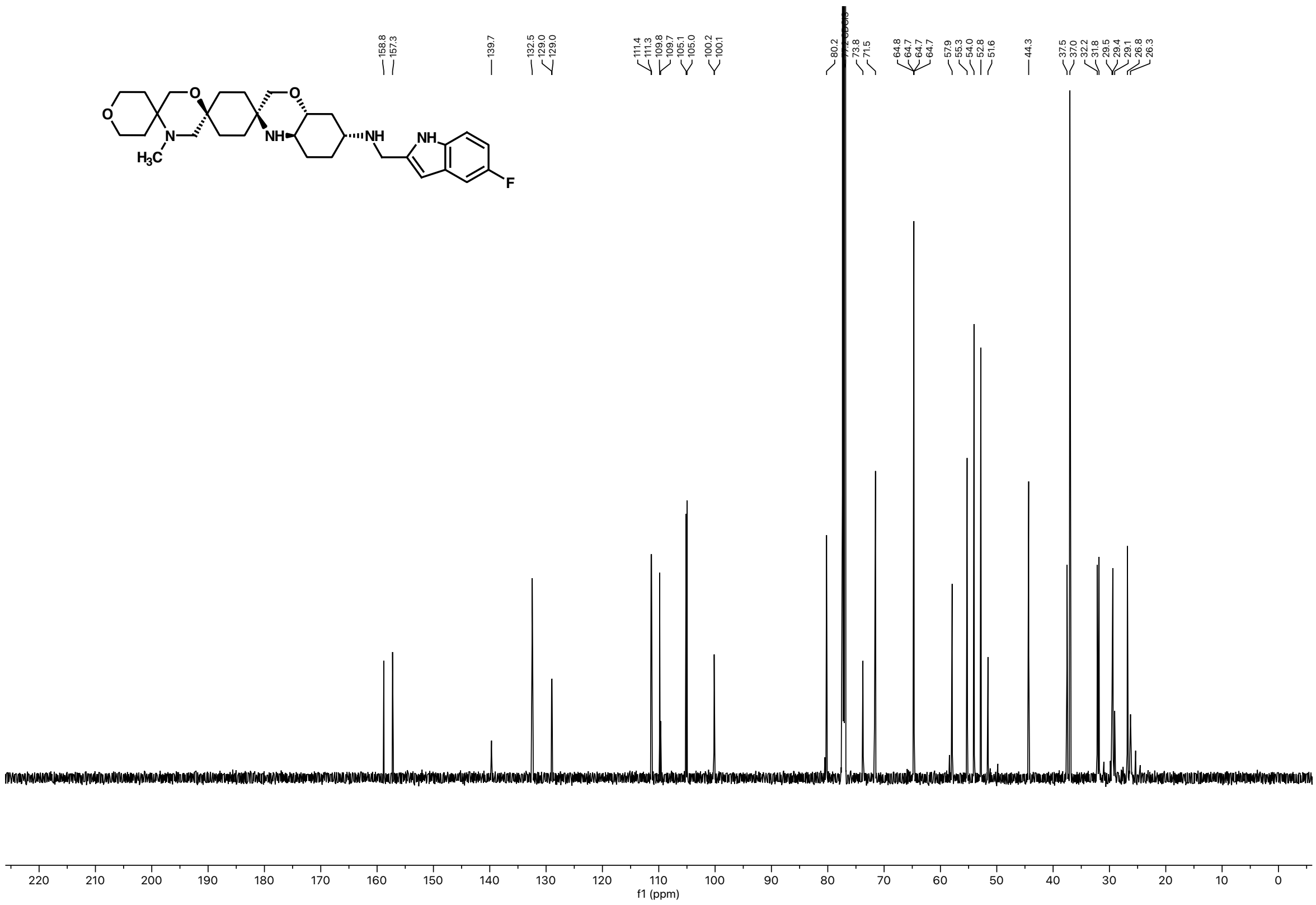

Compound 58a: ^{19}F NMR ($CDCl_3$)

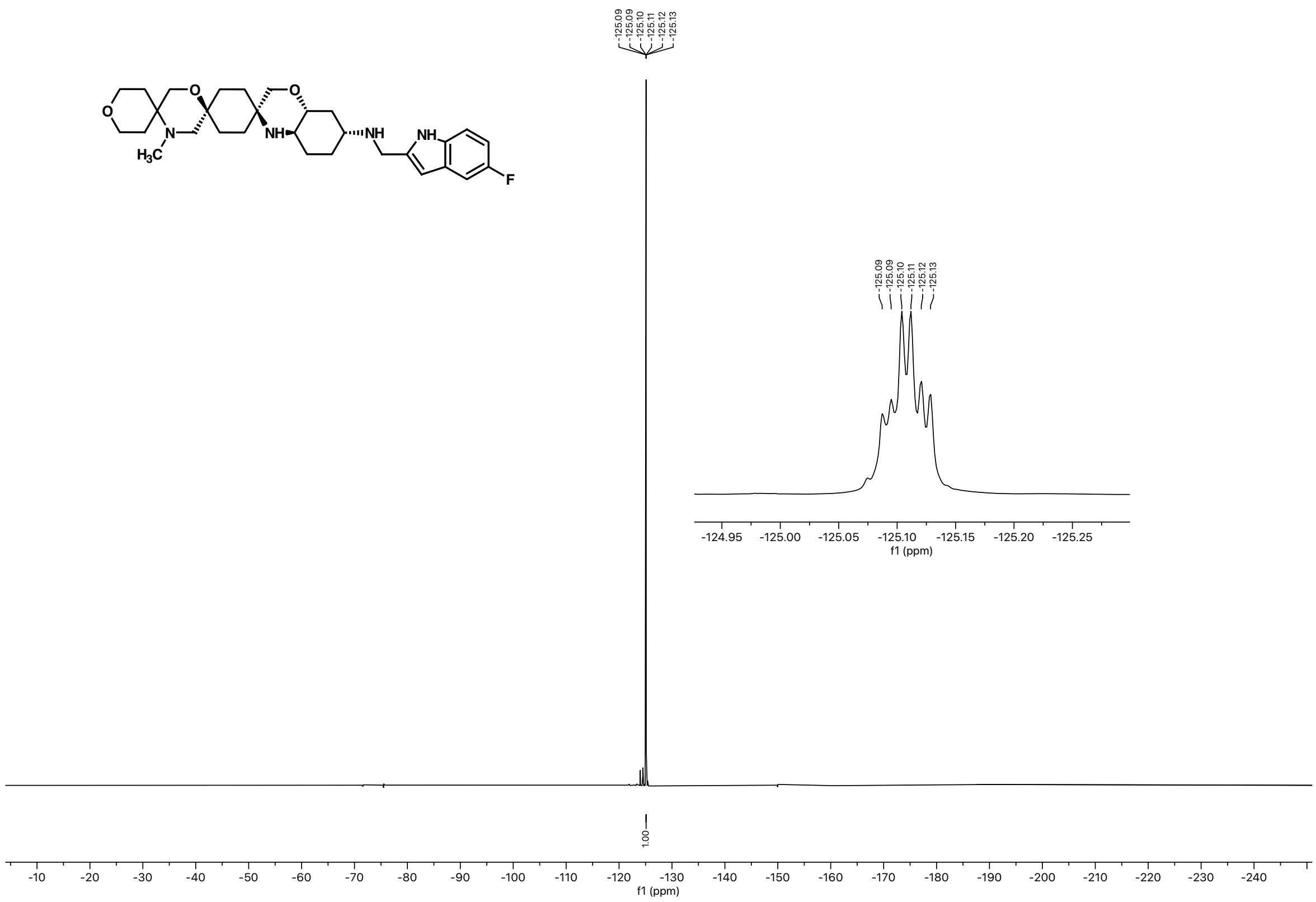

Compound 58b: ^{1}H NMR ($CDCl_3$) top, ^{13}C NMR ($CDCl_3$) bottom

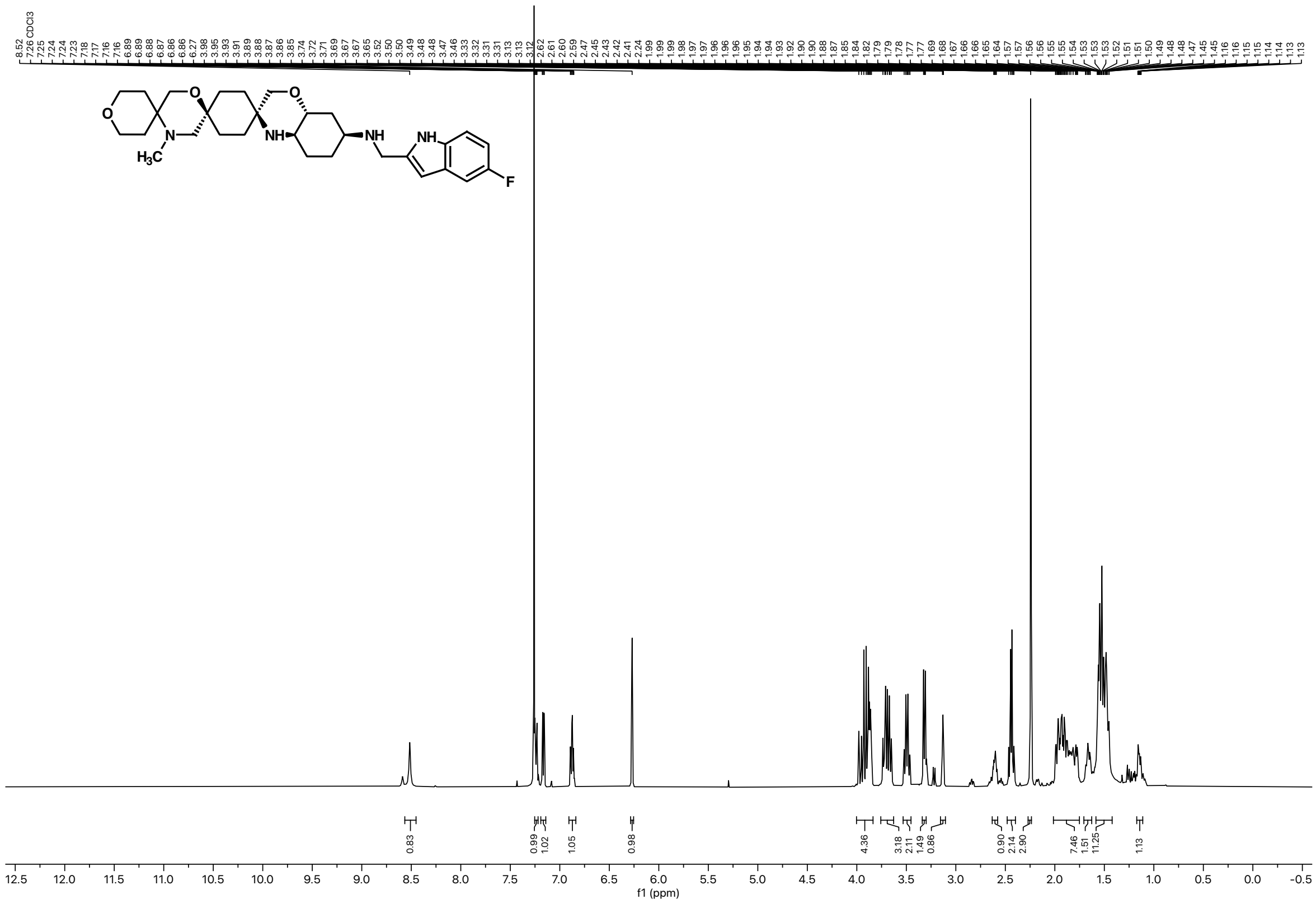

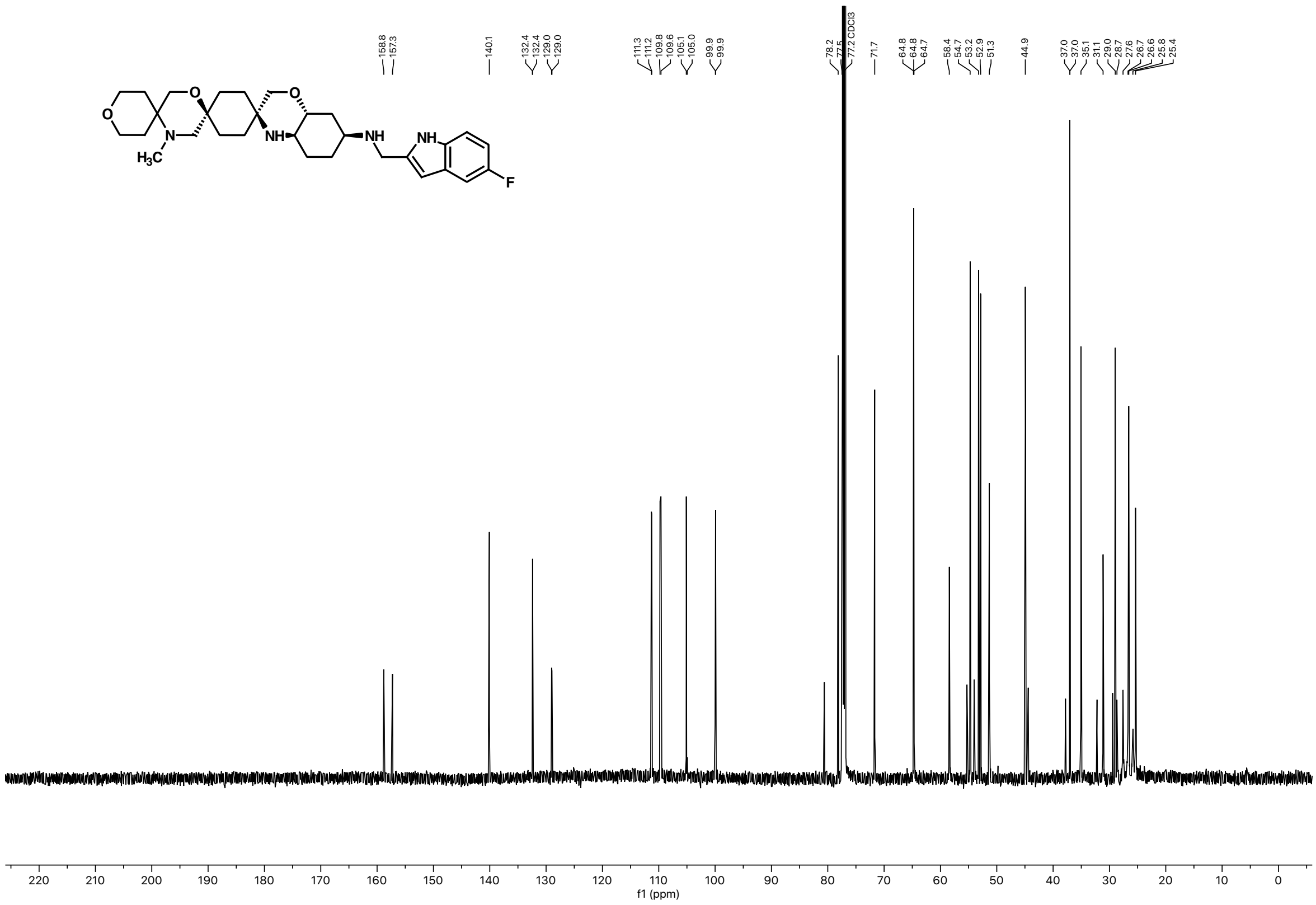

Compound 58b: ^{19}F NMR ($CDCl_3$)

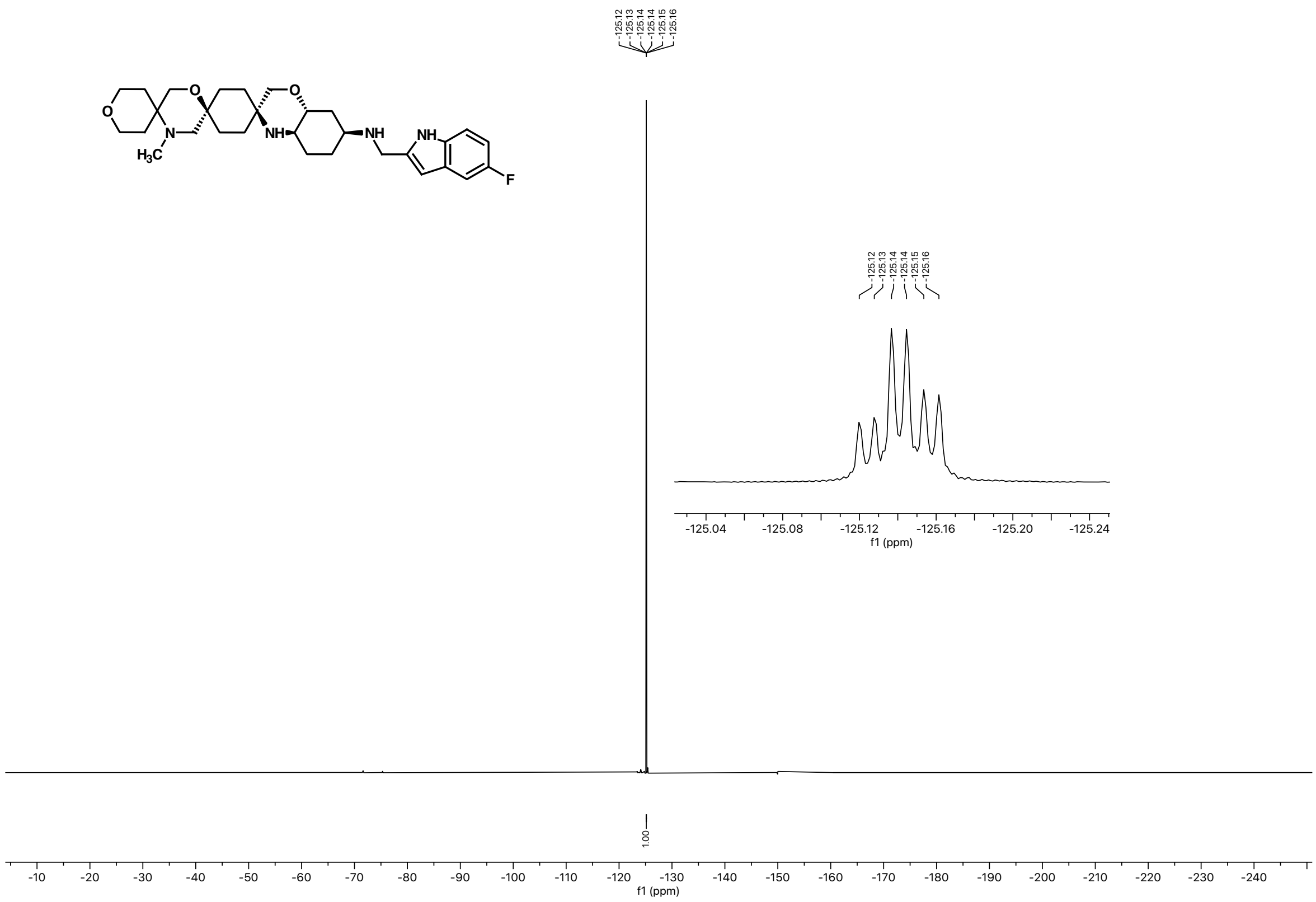

Compound 58c: ^{1}H NMR ($CDCl_3$) top, ^{13}C NMR ($CDCl_3$) bottom

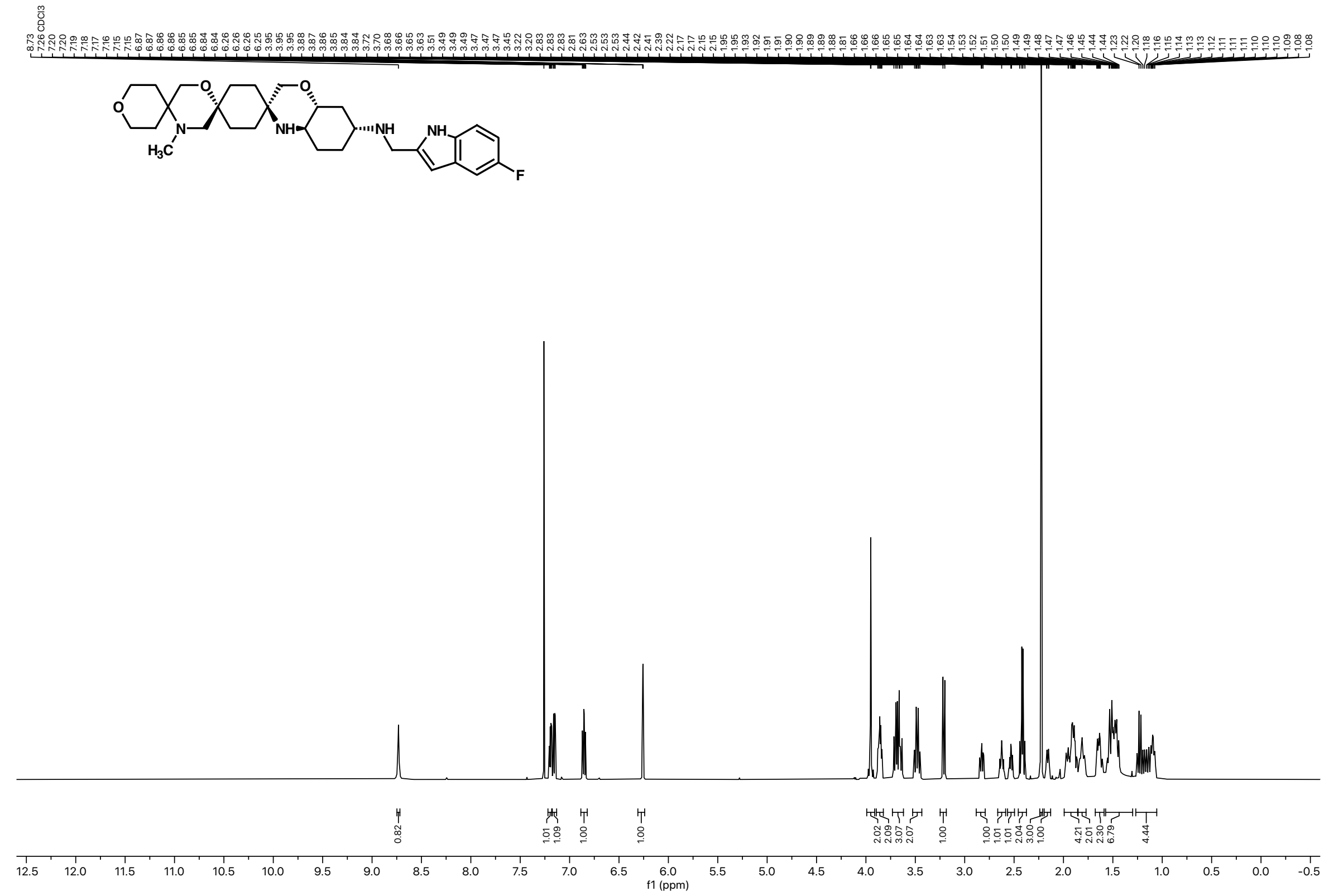

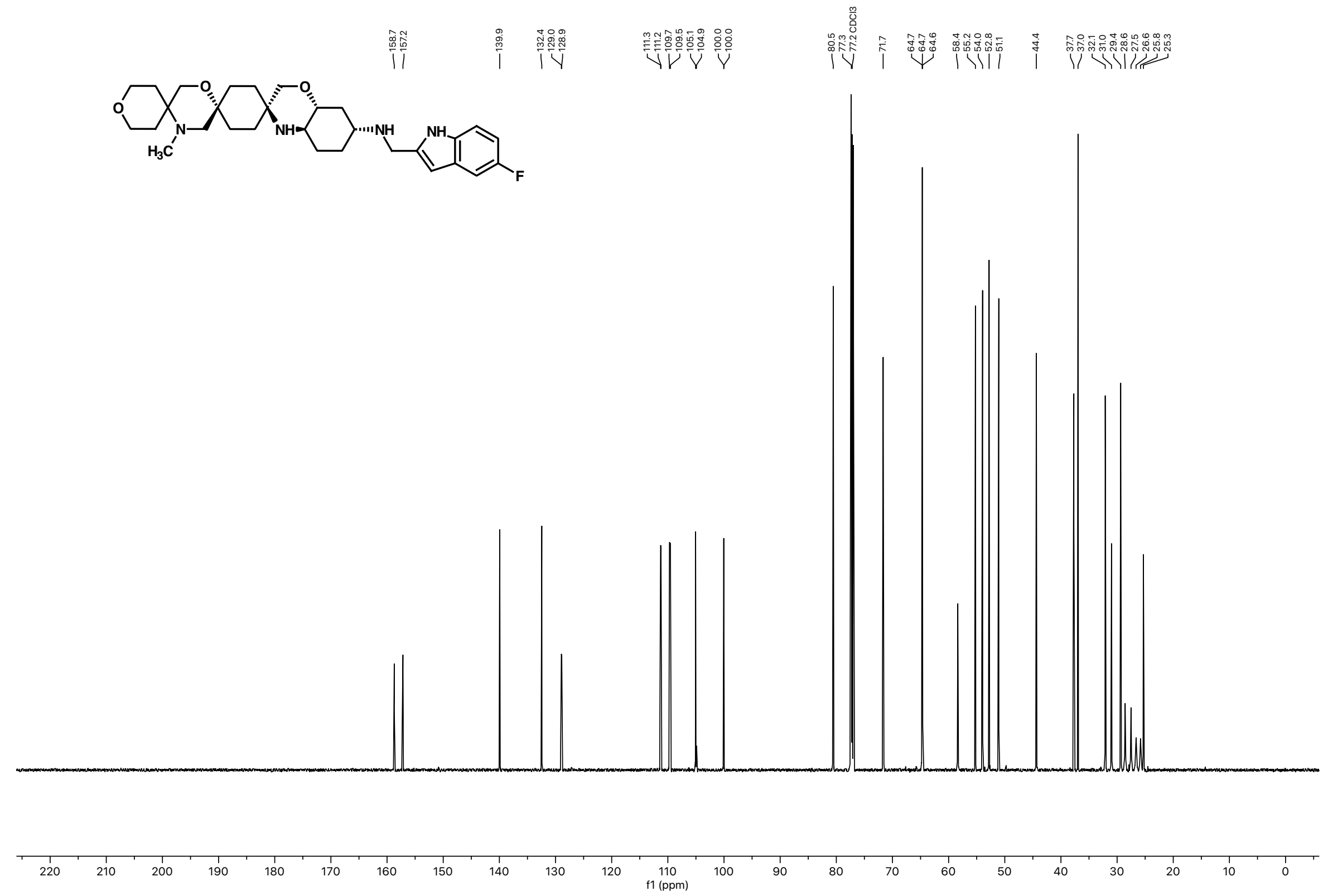

Compound 58c: ^{19}F NMR ($CDCl_3$)

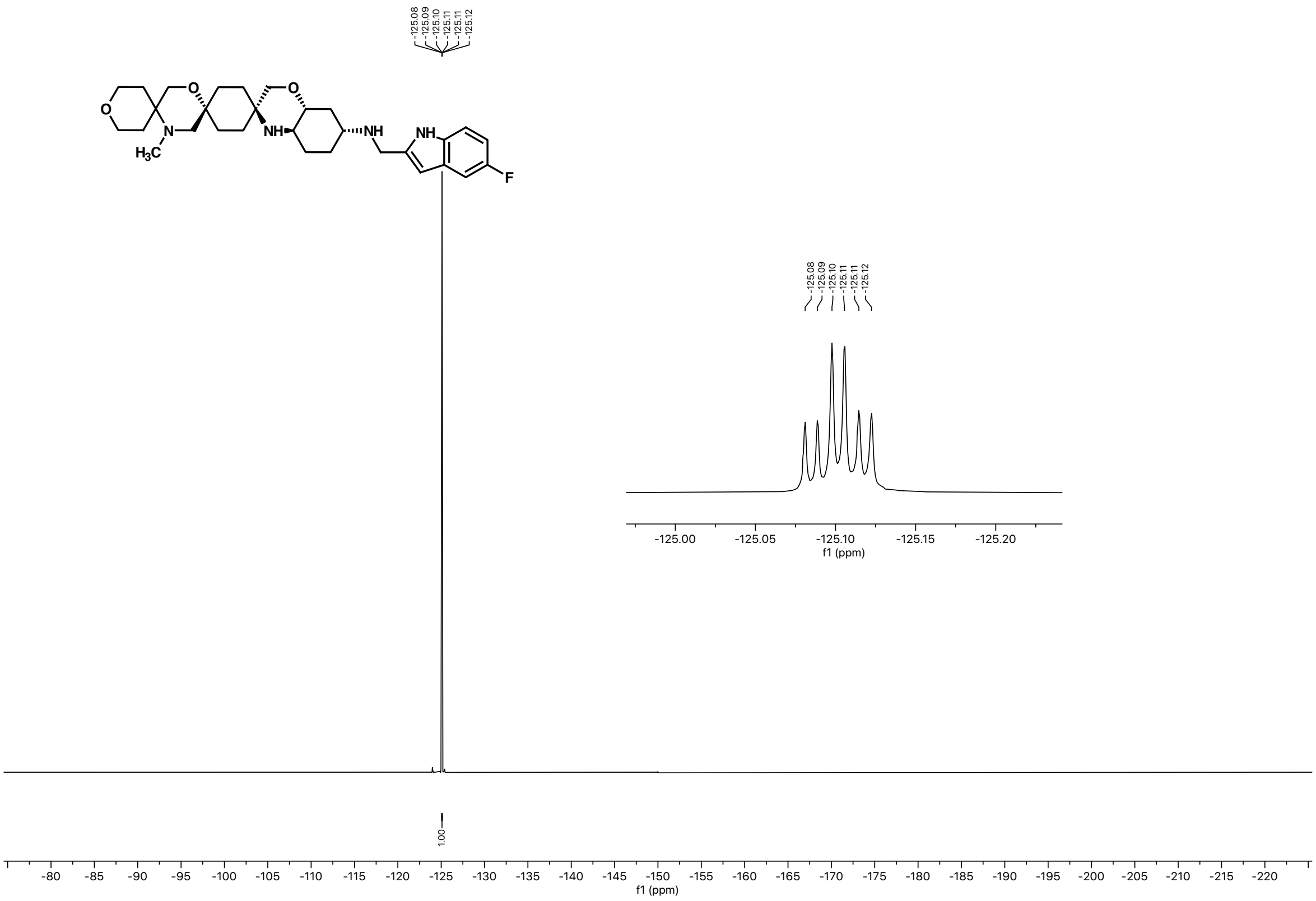

Compound 58d: ^{1}H NMR ($CDCl_3$) top, ^{13}C NMR ($CDCl_3$) bottom

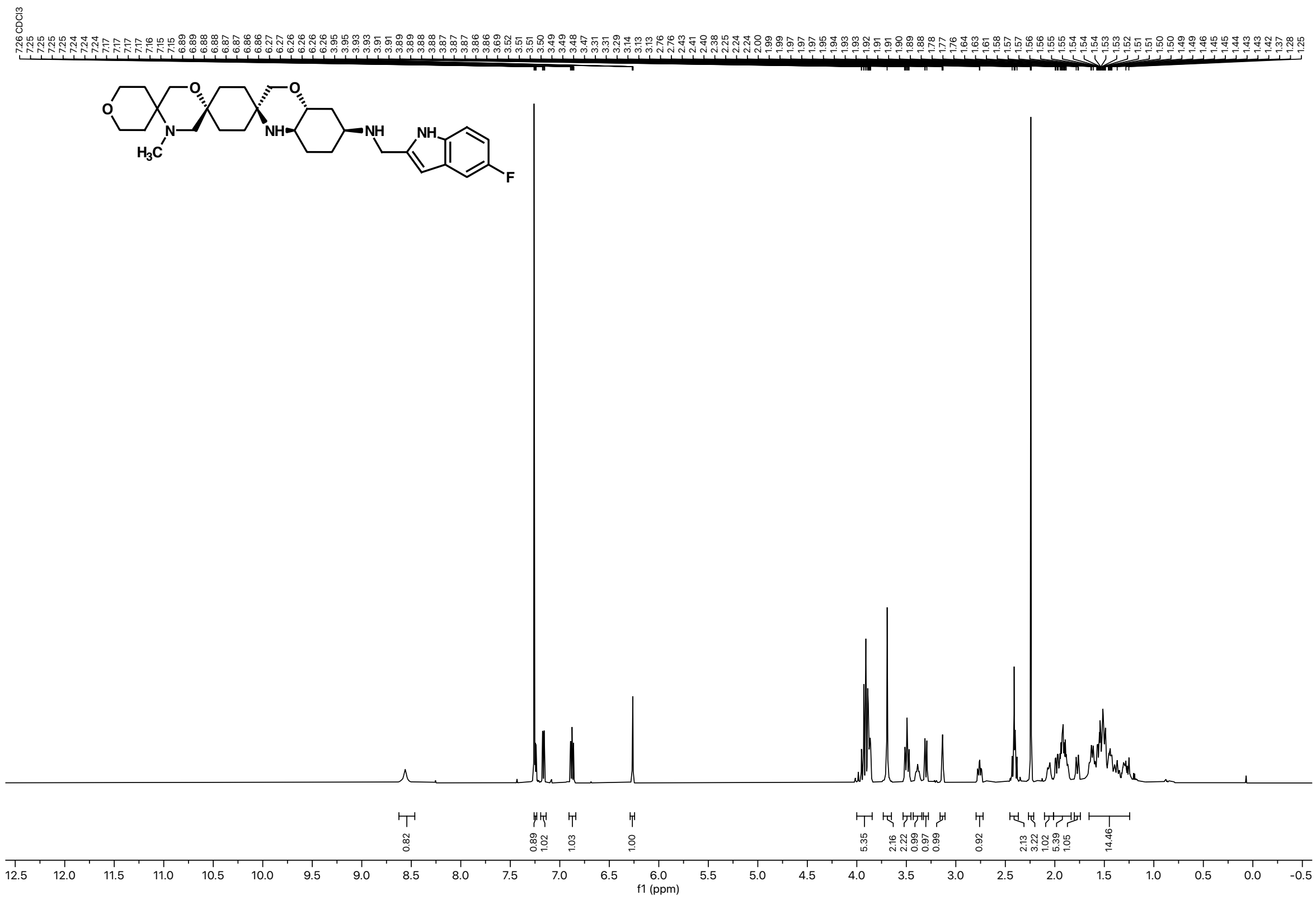

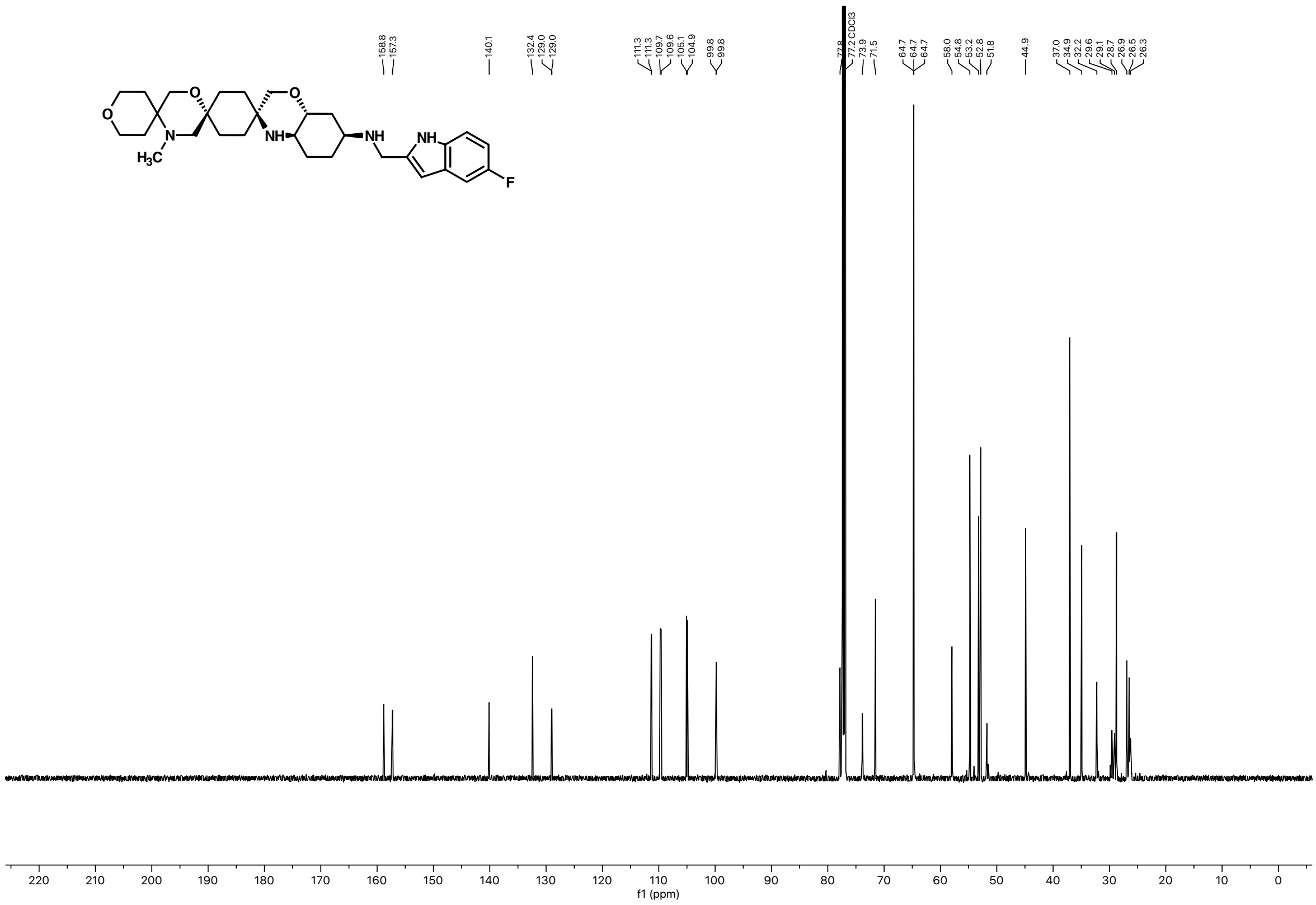

Compound 58d: ^{19}F NMR ($CDCl_3$)

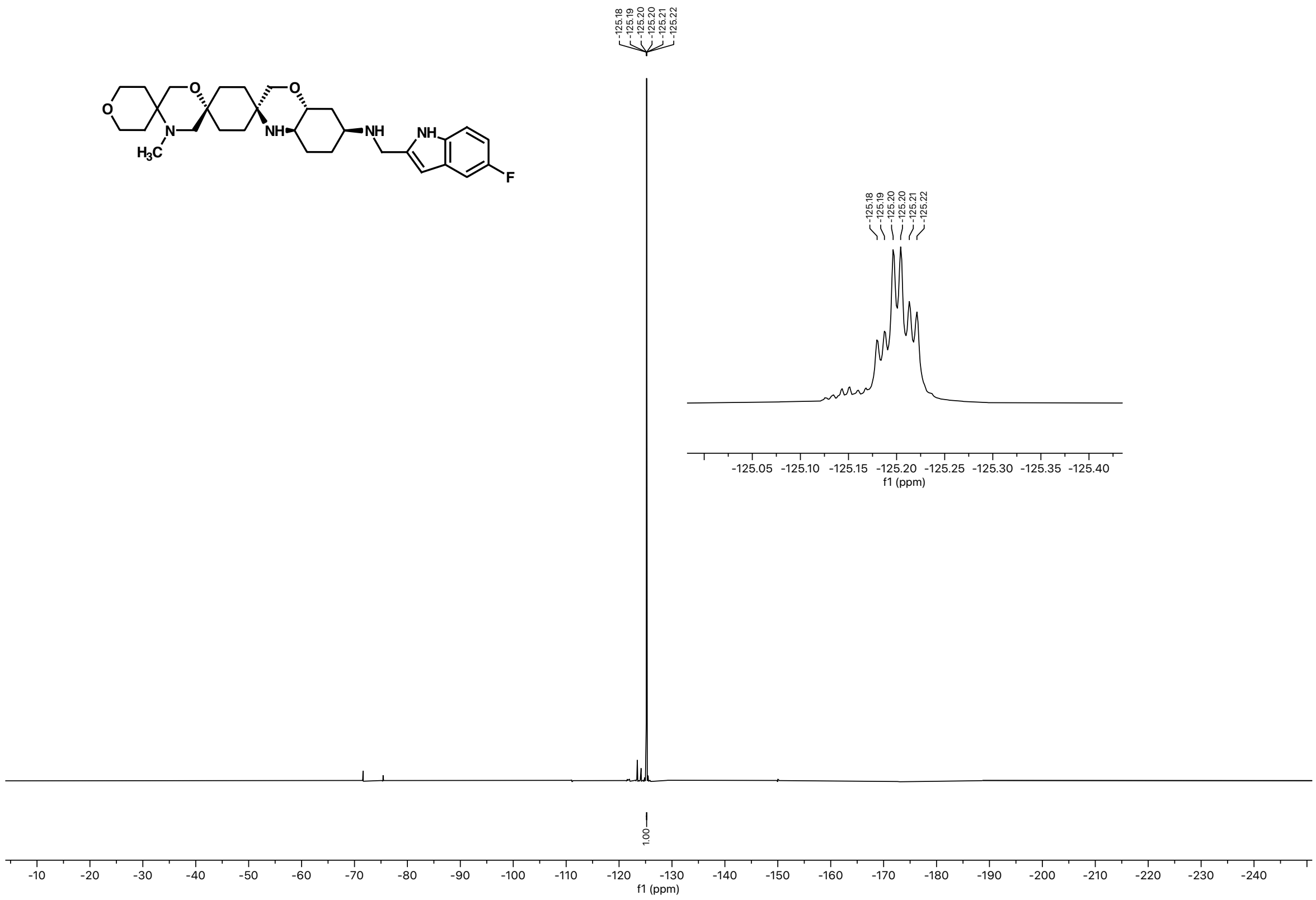

Compound 59a: ^{1}H NMR ($CDCl_3$) top, ^{13}C NMR ($CDCl_3$) bottom

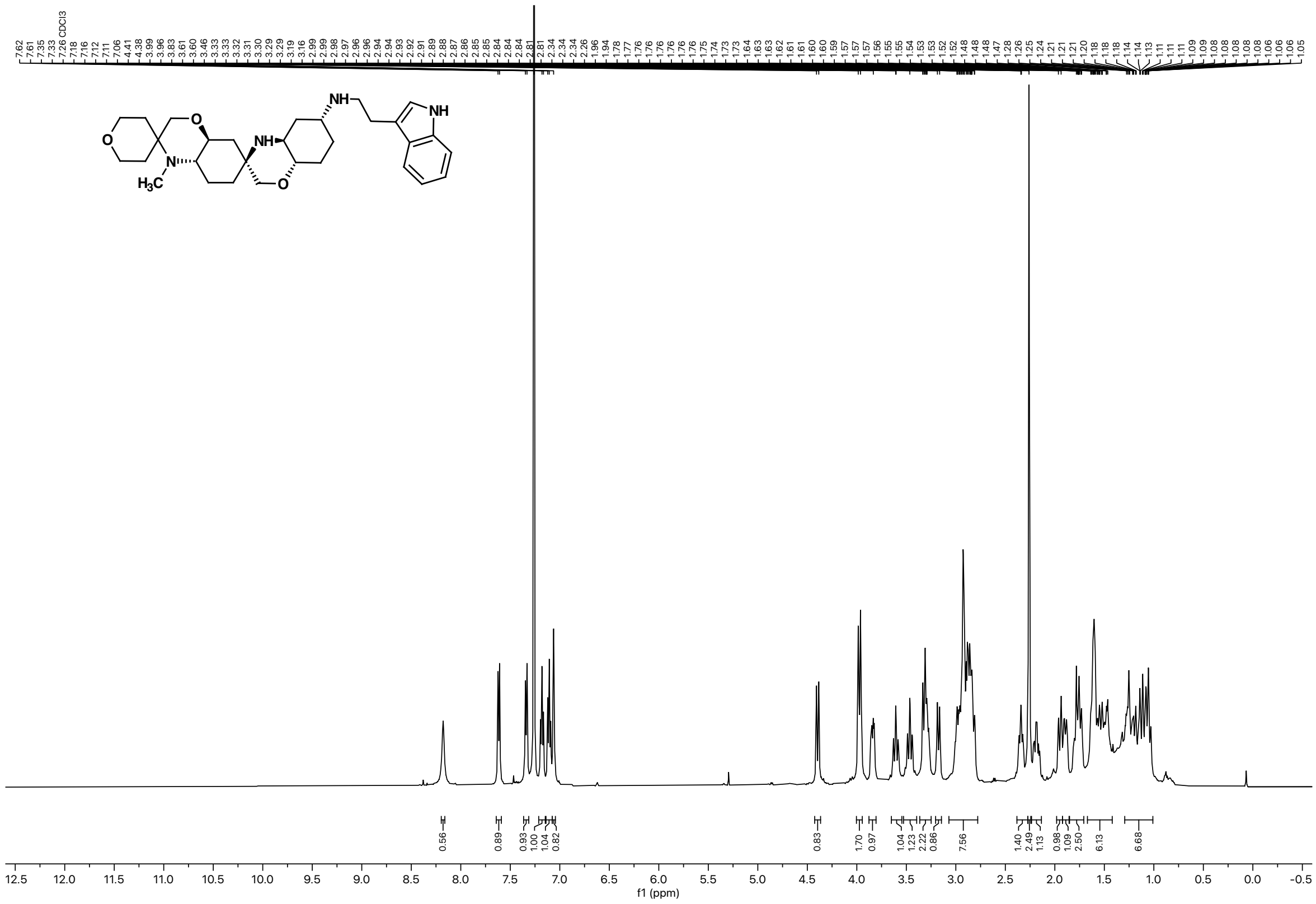

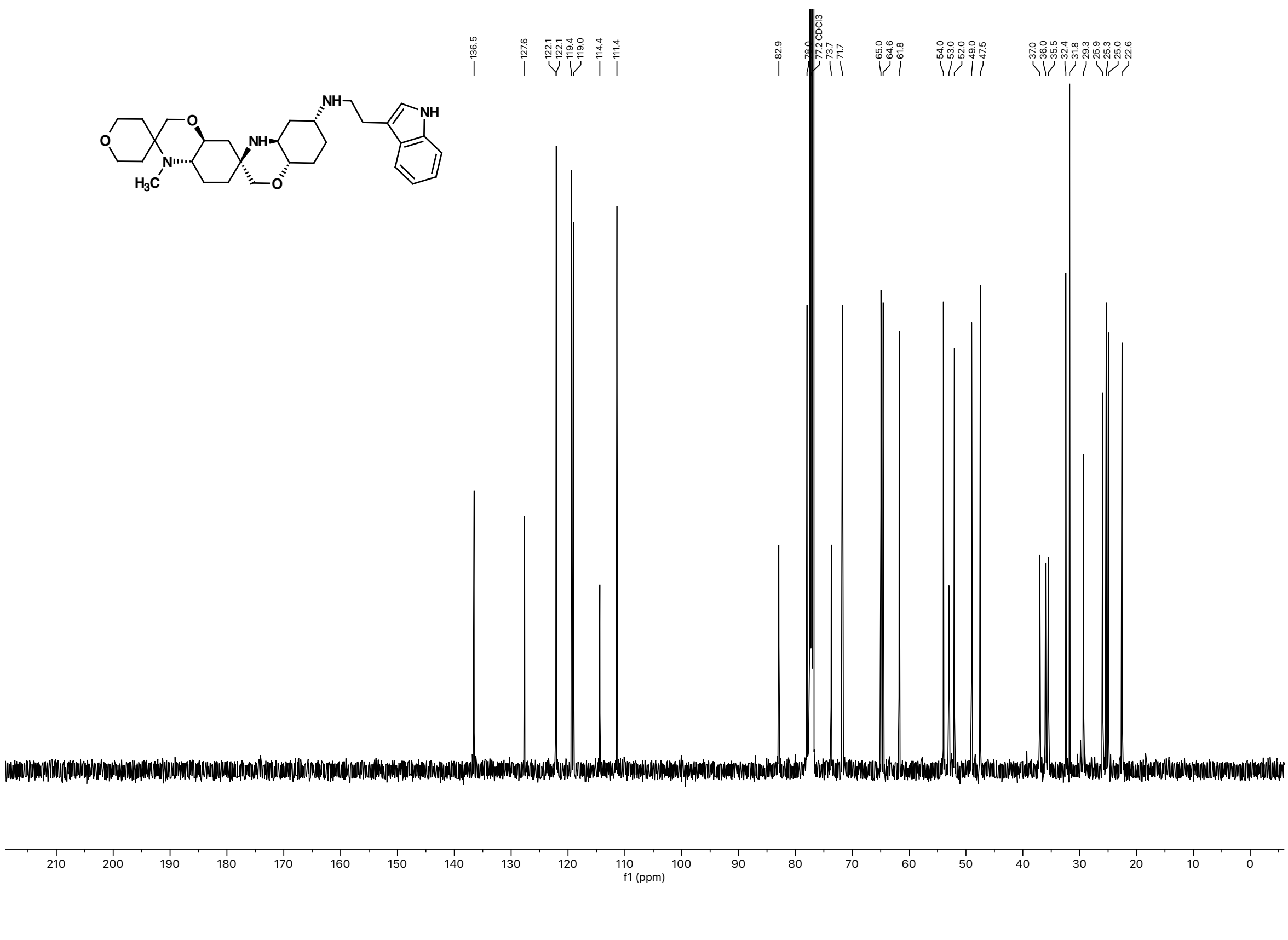

Compound 59b: ^{1}H NMR ($CDCl_3$) top, ^{13}C NMR ($CDCl_3$) bottom

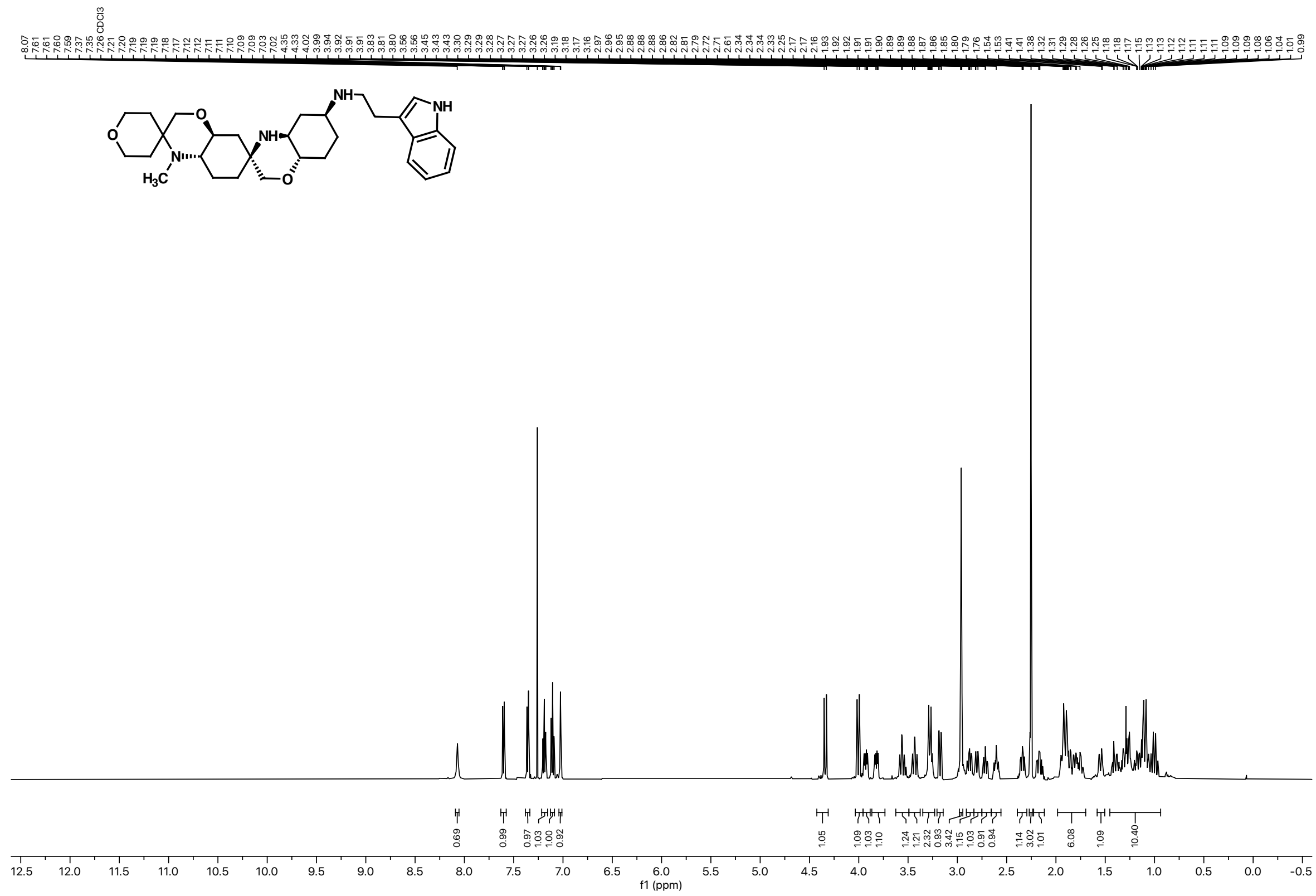

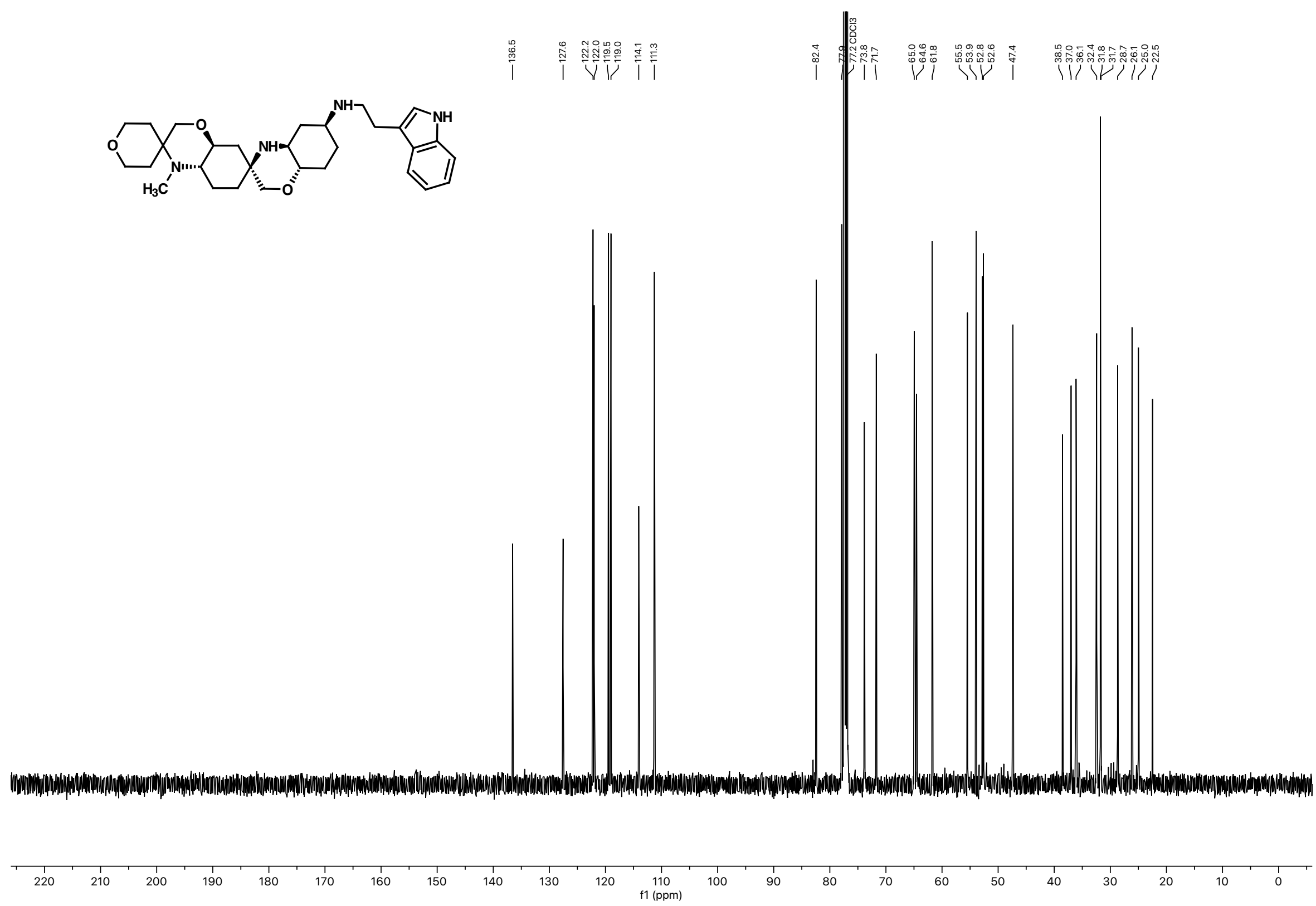

Compound 59c: ^{1}H NMR ($CDCl_3$) top, ^{13}C NMR ($CDCl_3$) bottom

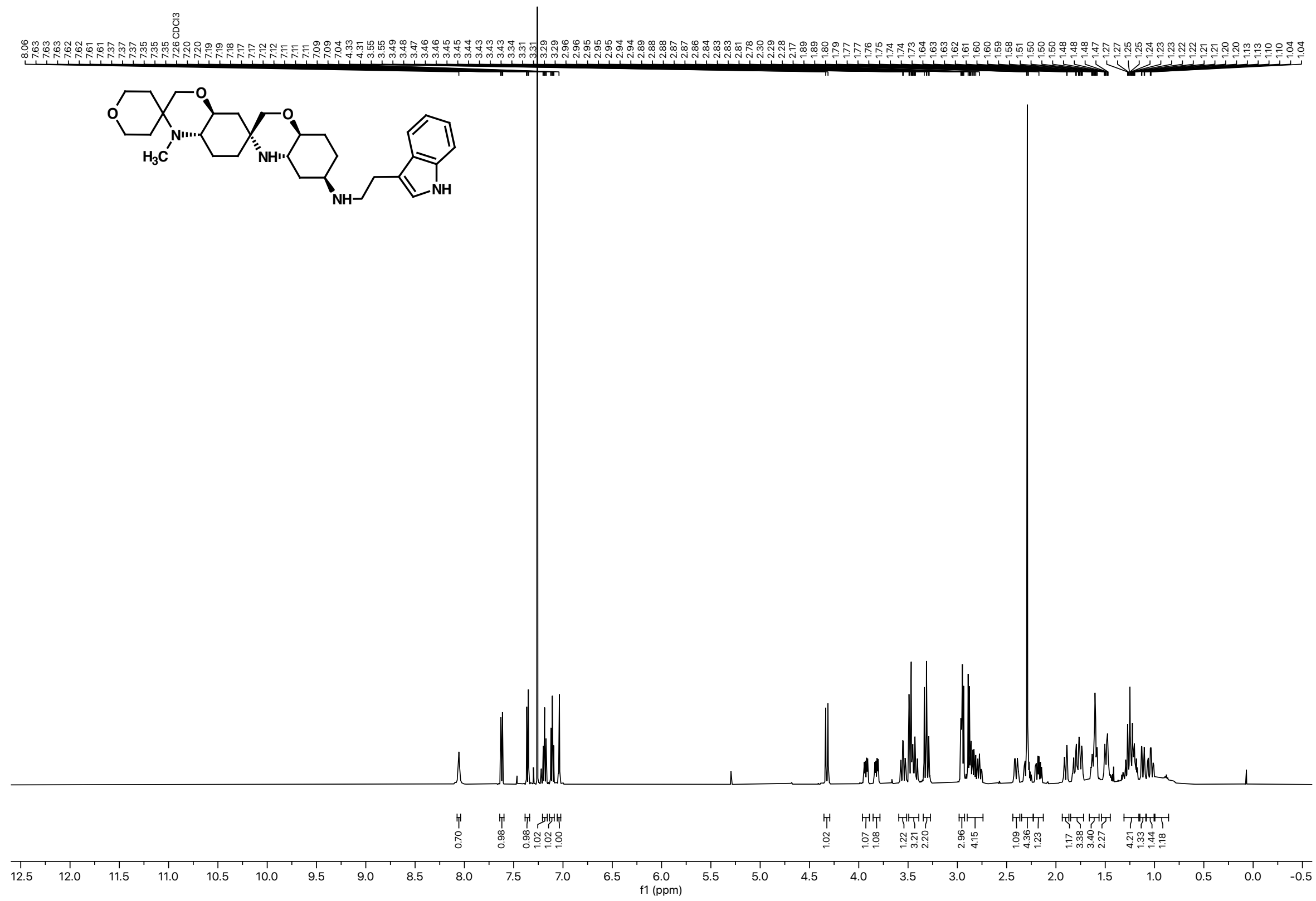

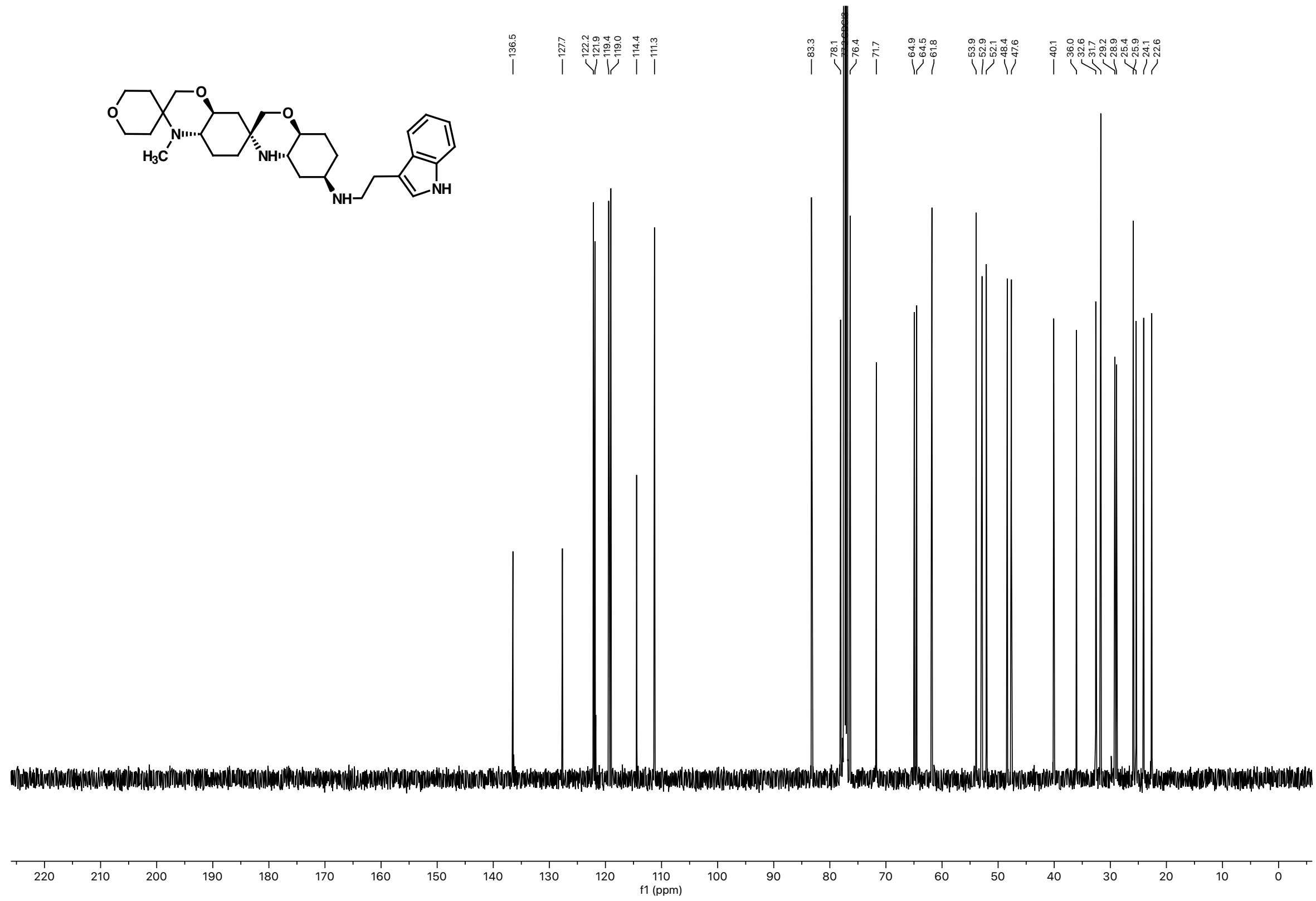

Compound 59d: ^{1}H NMR ($CDCl_3$) top, ^{13}C NMR ($CDCl_3$) bottom

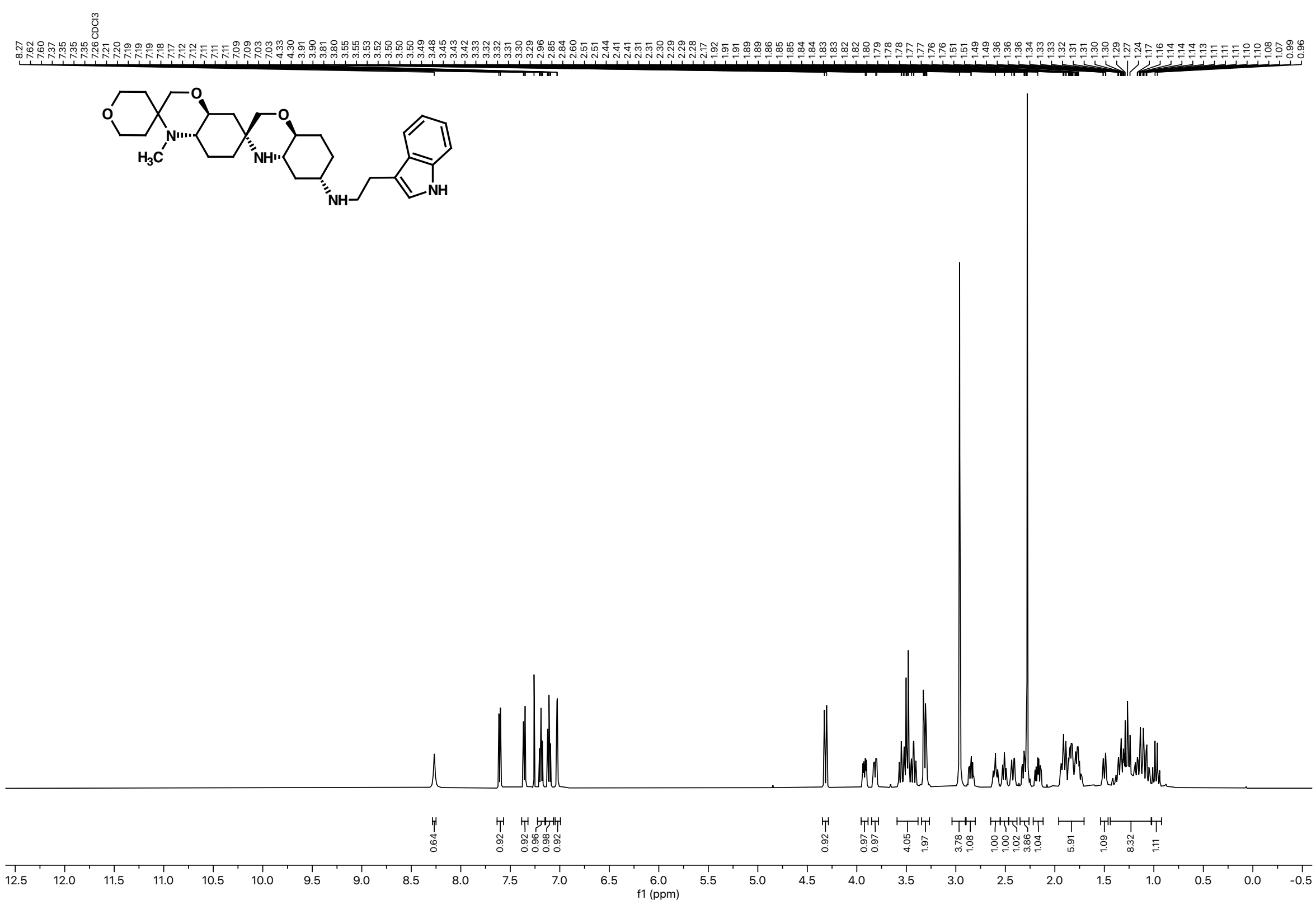

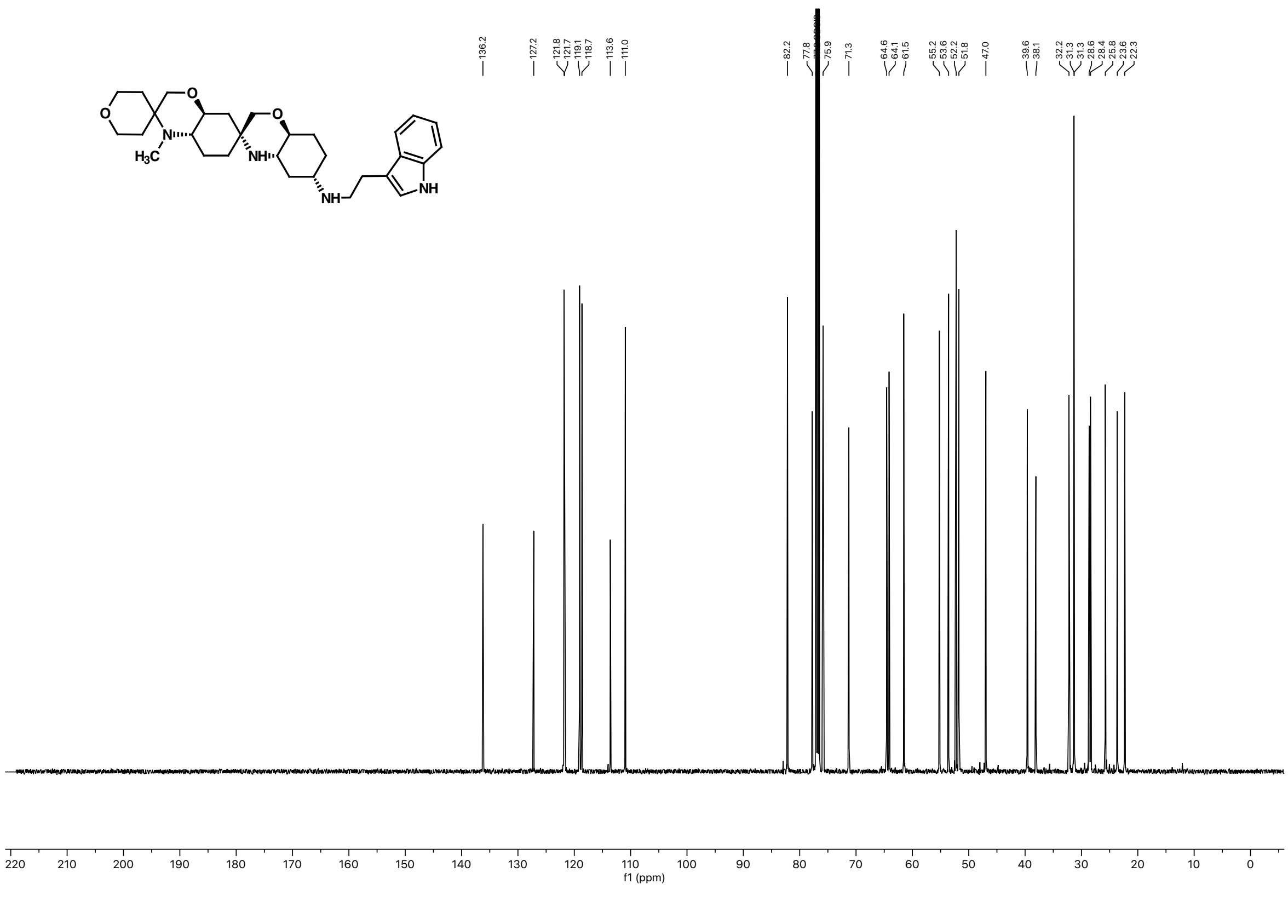

Supplement: Supplementary file 1 [file SC-017-D6SC05613J-s001.pdf]
